# Deer Skin Collagen Peptides Bound to Calcium: In Vitro Gastrointestinal Simulation of Digestion, Cellular Uptake and Analysis of Antioxidant Activity

**DOI:** 10.3390/nu16162585

**Published:** 2024-08-06

**Authors:** Rui Du, Li Sun, Jinze Liu, Fusheng Gao, Xiangjuan Guo, Meiling Shi, Pengli Guo, Weijia Chen, Ying Zong, Jianan Geng, Yan Zhao, Zhongmei He

**Affiliations:** 1 College of Chinese Medicinal Materials, Jilin Agricultural University, Changchun 130118, China; durui@jlau.edu.cn (R.D.); 20221595@mails.jlau.edu.cn (L.S.); liujinze0602@126.com (J.L.); m13180289293@126.com (F.G.); 18239227197@163.com (X.G.); anxia143341@163.com (M.S.); 18073579409@163.com (P.G.); chenweijia_jlau@163.com (W.C.); zongying7699@126.com (Y.Z.); gengjianan@jiau.edu.cn (J.G.); zhaoyan@jlau.edu.cn (Y.Z.); 2 Jilin Provincial Engineering Research Center for Efficient Breeding and Product Development of Sika Deer, Changchun 130118, China

**Keywords:** structural characterization, stability, Caco-2 cells, antioxidant activity, molecular docking

## Abstract

The by-product of deer skin, which has mostly been used as a decorative material, is rich in collagen and amino acids that could bind to Ca^2+^. Therefore, the preparation process, stability, antioxidant activity and calcium transport capacity of deer skin collagen peptide calcium chelate (Ca-DSCP) were investigated. In addition, the structure of the new chelate was characterized. The preparation process of Ca-DSCP was optimized using one-way experiments and response surface methodology. The ideal conditions were pH 9, 48 °C, and a peptide-to-calcium mass ratio of 5:1. The chelation rate was (60.73 ± 1.54)%. Zeta potential, XRD, UV–vis and FTIR analyses yielded that deer skin collagen peptides (DSCP) underwent a chelating reaction with calcium ions to form new structures. The stability of Ca-DSCP and the fraction of bioavailability of calcium ions were determined using in vitro gastrointestinal digestion and a Caco-2 cell monolayer model. The results showed that fraction of bioavailability and stability of DSCP were improved by influencing the structural characterization. The antioxidant activities of DSCP and Ca-DSCP were evaluated by measuring relevant oxidative stress indicators, DPPH radical scavenging capacity and hydroxyl radical scavenging capacity. Finally, bioinformatics and molecular docking techniques were utilized to screen and study the antioxidant mechanism of DSCP.

## 1. Introduction

Calcium is a trace metal element that is necessary to sustain the body’s vital activities. The body needs calcium to build and maintain strong bones [1]. The heart, muscles and nerves also need calcium to function properly. Adequate absorption of calcium in the body can reduce the risk of developing osteoporosis and rickets [2]. Diet is by far the best way to obtain calcium, but if diet is insufficient, calcium supplements are also an option. Most calcium supplements only provide adequate levels of calcium without taking into account the body’s role in absorbing calcium, resulting in low absorption and bioavailability [3].

Calcium supplements range from inorganic calcium to organic calcium, and then from organic calcium to chelate. The first two types of calcium products are ionized calcium, which tends to form calcium deposits in the gastrointestinal tract and reduce their absorption efficiency. In contrast to these two, calcium peptide in chelated form is a new class of chelated system and an effective mineral supplement [4]. It can enter the organism directly in the form of inorganic salt, and is rapidly digested and absorbed in the small intestine using peptide as a carrier. It has a high absorption rate and is safe; thus, it has good application prospects. In addition, metal chelating peptide can also combine with unpaired copper ions, iron ions and other metal ions in the organism, thus reducing the generation of free radicals and exerting antioxidant activity. It has been demonstrated that magnesium-chelated bovine bone collagen peptides showed better DPPH and ABTS radical scavenging activity than bovine bone collagen peptides [5]. However, currently prepared peptides are chelated with calcium ions under certain reaction conditions, such as casein phosphopeptide–calcium chelate [6] and egg white peptide–calcium chelate [7]. Although these peptide–calcium chelates have certain calcium supplementation functions, most of the peptides only act as carriers of calcium ions and lack corresponding biological activities [8].

Deer are being introduced and farmed in an increasing number of countries around the world [9,10]. Deer resources have become abundant, but the utilization of deer skin as a by-product is inefficient and is often used in the tanning industry [11]. Compared with other sources of skin gelatin hydrolysis products, the application and development of deer skin collagen peptides are far from adequate [12]. However, deer skin contains a lot of protein, of which collagen is abundant [13]. The peptides obtained from the hydrolysis of collagen are known as collagen peptides. Some studies have reported that a large number of peptides with antioxidant activity have been identified from various sources of collagen and validated in different antioxidant experiments [14]. Therefore, the aim of this study was to optimize the process conditions for the preparation of deer skin collagen peptide calcium chelate (Ca-DSCP) by single-factor and response surface tests. The structural characterization was studied to analyze whether new substances were formed. It was also used to demonstrate whether calcium could be successfully chelated with deer skin collagen peptides (DSCP). The stability and antioxidant activity of deer skin collagen peptides chelated with calcium were tested. In addition, the calcium transporting effect of Ca-DSCP was evaluated by Caco-2 cell monolayers in this paper. Finally, DSCP were selected for molecular docking to verify the antioxidant basis of DSCP and Ca-DSCP. It was expected to provide a basis for the development of products with calcium supplement function and antioxidant activity.

## 2. Materials and Methods

### 2.1. Materials and Reagents

Deer skin collagen was digested with alkaline protease, papain and trypsin for 4 h and then filtered through a 0.45 μm membrane. Separation and purification were performed using a Sephadex G-25 column. The prepared deer skin collagen peptide (DSCP) was characterized by LC-MS for its sequence. Dulbecco’s modified Eagle’s medium (DMEM) and fetal bovine serum (FBS) were bought from Gibco (Grand Island, NY, USA). Caco-2 cells were bought from Procell Life Science & Technology Co., Ltd. (Wuhan, China). All other reagents were of analytical reagent grade.

### 2.2. Preparation of Ca-DSCP

#### 2.2.1. Single-Factor Experiments

The effects of Ca-DSCP on calcium chelation rate were investigated by three factors: mass ratio of peptide to calcium (1:1, 3:1, 5:1, 7:1, 9:1 and 11:1), pH (6, 7, 8, 9, 10 and 11) and chelation temperature (5, 20, 35, 50, 65 and 80 °C). After 40 min of a water bath, DSCP was precipitated by the addition of 9 times the solution volume of anhydrous ethanol. This precipitate required centrifugation at 4000× *g* for 20 min, collection and lyophilization.

#### 2.2.2. Response Surface Experiments

According to the single-factor experiments, A-Mass ratio of peptide to calcium, B-pH and C-Temperature were selected. The 3 factors and 3 level-experiments were conducted to explore the best optimization conditions using the calcium chelating rate as the response value. The appropriate content was entered into Design–Expert 13 and set the objective function. The experiment was completed according to the content generated by the software, and the experimental design is shown in Table 1. Design–Expert 13 software was used to design experiments and the obtained data were processed and analyzed [15].

#### 2.2.3. Determination of Calcium Chelation Rate

DSCP (10 mg/mL) was dissolved in deionized water, and then CaCl_2_ was added at different mass ratios and water baths were performed for 40 min at different temperatures and pH conditions. At the end of the calcium chelation reaction, DSCP was precipitated by the addition of 9 times the solution volume of anhydrous ethanol. This precipitate required centrifugation at 4000× *g* for 20 min, collection and lyophilization. The amount of calcium in the supernatant and total calcium in the reactant solution was then determined by an atomic spectrophotometer (AA-6300F/G, Shimadzu Co., Kyoto, Japan). The calculation formula is as follows:

(1) $$\mathrm{Calcium}\;\mathrm{chelation}\;\mathrm{rate}\;\left(\%\right)=\frac{A1-A2}{A1}\times 100$$

where A1 is the total calcium content in the solution (g) and A2 is the calcium content in the supernatant (g).

### 2.3. Amino Acid Analysis

The amino acid composition of DSCP was determined using an amino acid analyzer (L-8900, Hitachi Co., Tokyo, Japan) according to previously reported methods [16]. The sample was filtered through a membrane filter with a pore size of 0.22 µm and injected directly into the analyzer for measurement. Hydrolysis was performed for 24 h at 110 °C, with a 6 mol/L HCl solution and vacuum.

### 2.4. Zeta Potential and XRD of DSCP and Ca-DSCP

DSCP and Ca-DSCP (1 mg/mL) potentials were determined using a zeta potential analyzer (90Plus zeta, Brookhaven Instruments Co., New York, NY, USA). The test sample was mixed for 5 s first and then transferred to a Malvern polystyrene U-shaped cell. The measurements were conducted at 25 °C. The X-ray diffraction (XRD) data of CaCl_2_, DSCP and Ca-DSCP were determined for a voltage of 40 kV and a current of 40 mA (Rigaku Ultima IV, Rigaku Corporation, Akishima, Japan) with a scan angle (2*θ*) range of 5–90° and a speed of 5 deg/min.

### 2.5. UV–vis and FTIR of DSCP and Ca-DSCP

Ultraviolet spectrophotometer (UV-6100, MAPADA, Shanghai, China) was used to determine the ultraviolet spectra of DSCP and Ca-DSCP in the wavelength range of 190~400 nm. Appropriate amounts of DSPs and DSPs-Ca were prepared with ultrapure water to 1 mg mL^−1^, respectively, and scanned at 190–400 nm. The UV–visible spectrophotometer was blank-corrected with deionized water prior to the measurement of the samples. The dried DSCP and Ca-DSCP were evenly mixed with an appropriate amount of KBr, tableted until transparent, and the sample was scanned by infrared spectrometer (iS50R, Thermo Fisher Scientific, Waltham, MA, USA), with a scanning range of 400~4000 cm^−1^ [17].

### 2.6. Stability of Ca-DSCP

#### 2.6.1. Acid–Base Stability and Thermal Stability

Ca-DSCP was prepared as a 5 mg/mL solution. The pH (2, 4, 6, 8, 10) was adjusted with 1 mol/L HCl and 1 mol/L NaOH, and then the solution was placed in a water bath at 37 °C for 2 h. The thermal stability of the solutions was measured in a water bath at different temperatures (50, 60, 70, 80, 90 °C) for 1 h. After cooling and centrifugation, the supernatant was assayed for calcium content by atomic absorption spectrometry [18].

#### 2.6.2. In Vitro Gastrointestinal Digestion

In vitro gastrointestinal digestion was carried out in two stages. Stomach stage: Ca-DSCP was prepared into a 5 mg/mL solution, which was adjusted to pH 2.0, 2% pepsin was added, and a water bath at 37 °C was used. In the small intestine stage, the gastric digested solution was adjusted to pH 7.5, trypsin was added and the water bath was at 37 °C. A portion of the digested solution was taken out every half hour, and the enzyme was inactivated at high temperature for 8 min, precipitated by anhydrous ethanol, and centrifuged. The calcium content in chelates was determined by atomic absorption spectrophotometry [19]. The calcium retention rate of Ca-DSCP after digestion in the stomach and small intestine were calculated separately. The calculation formula is as follows:

(2) $$\mathrm{Calcium}\;\mathrm{retention}\;\mathrm{rate}\;\left(\%\right)=\frac{B1}{B2}\times 100$$

where B1 is the calcium content of the treatment preparations (g) and B2 is the calcium content of the control preparations (g).

### 2.7. Cellular Experiments

#### 2.7.1. Caco-2 Cell Culture

**Figure 1:**
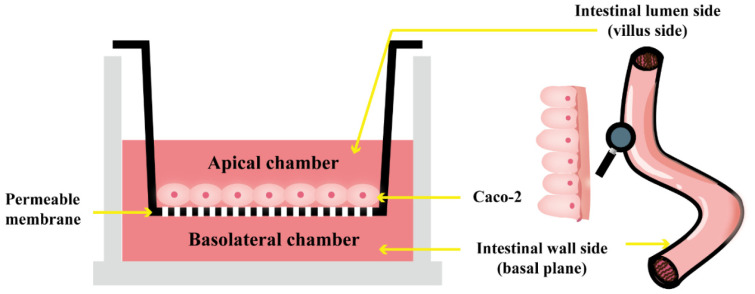
Schematic diagram of the Caco-2 cell model.

Caco-2 cells were cultured in DMEM medium containing 20% fetal bovine serum (FBS), 1% penicillin and streptomycin. They were cultured in a humidified incubator at 37 °C with 5% CO_2_. The medium was changed every 2 days [20]. Caco-2 cells were inoculated in 12-well Transwell™ (0.4 μm pore size, Millipore Corp., Billerica, MA, USA) plates at a density of 1 × 10^5^/mL. The apical chamber of the well plate was added with 0.5 mL of cell culture medium and the basolateral chamber required 1.5 mL of medium. Cells were changed every other day and incubated for 7 days to 21–27 days, at which point they formed a dense monolayer that could be used for subsequent experiments [21]. The Caco-2 cell culture model is shown in Figure 1. The entire process of establishing the Caco-2 cell monolayer model requires monitoring of transepithelial electrical resistance (TEER) values (over 300 Ω·cm^−2^) by means of a resistive device (Millicell ERS-2, Merck KGaA, Darmstadt, Germany).

#### 2.7.2. Cytotoxicity Test of Caco-2 Cells

Caco-2 cells were inoculated in 96-well plates with 100 μL of cell suspension per well at a density of approximately 5 × 10^4^ cells/mL. The cells were incubated for 24 h at 37 °C with 5% CO_2_, and then incubated for 24 h in 100 μL of medium containing different concentrations of Ca-DSCP (25, 50, 100, 200, 400, 800, 1600, 2000 μg/mL). The old medium was aspirated and washed three times with PBS. Then, 100 μL of fresh medium and 10 μL of CCK-8 solution were added and incubated for 1 h. The absorbance at 450 nm was measured [22].

#### 2.7.3. Caco-2 Cells Monolayer Calcium Transport Studies

After 21 days of incubation, the medium was discarded and the Transwell chamber was rinsed 2–3 times with 37 °C pre-warmed HBSS (calcium free) buffer. Next, 1.5 mL of pre-warmed HBSS was added to the basolateral chamber and 0.5 mL of Ca-DSCP solution was added to the apical chamber. For the control group, 1.5 mL of pre-warmed HBSS was added to the basolateral chamber and 0.5 mL of CaCl_2_ solution with equal calcium content was added to the apical chamber. At different transportation time points (30, 60, 90, 120, 150 and 180 min), the chamber solution was removed and the same volume of fresh HBSS was added, keeping the volume constant [23]. The calcium content was determined by atomic absorption spectrometry.

#### 2.7.4. Measurement of Oxidative Stress Indicators

Logarithmically grown cells were inoculated in 6-well plates at a density of 4 × 10^5^ cells/well and cultured in a constant temperature incubator at 37 °C and 5% carbon dioxide for 48 h. Normal, model, DSCP and Ca-DSCP groups were set up. The model group was induced with 200 μmol/L H_2_O_2_ stimulation for 12 h. The Ca-DSCP group was added with Ca-DSCP to continue the culture for 6 h. Staining was performed at 37 °C using DCFH-DA (S0033S, Beyotime Biotechnology, Shanghai, China) to determine the content of reactive oxygen species (ROS). The collected cells were lysed and centrifuged. The activities of superoxide dismutase (SOD), glutathione peroxidase (GSH-Px), catalase (CAT) and the content of malondialdehyde (MDA) were determined with a commercial kit according to the corresponding instructions (NanJing JianCheng Bioengineering Institute, Nanjing, China).

### 2.8. Antioxidant Capacity of DSCP and Ca-DSCP

#### 2.8.1. DPPH Radical Scavenging Ability Assay

A 0.1 mM DPPH solution was prepared in ethanol and stored away from light. Sample solution concentrations were set to 0.3, 0.5, 1.0, 2.0 and 3.0 mg/mL [24]. After mixing, it was protected from light at room temperature for 30 min. The absorbance at 517 nm was measured, averaged and the DPPH clearance at each concentration was calculated.

(3) $$\mathrm{DPPH}\;\mathrm{radical}\;\mathrm{scavenging}\;\mathrm{rate}\;\left(\%\right)=\left(1-\frac{C1-C2}{C3}\right)\;\times \;100$$

where C1 is the absorbance of the sample solution and the DPPH alcohol solution, C2 is the absorbance of the sample solution and ethanol, and C3 is the absorbance of the sample solution and water.

#### 2.8.2. Hydroxyl Radical Scavenging Ability Assay

Sample solution concentrations were set to 0.3, 0.5, 1.0, 2.0 and 3.0 mg/mL. Firstly, 9 mmol/L of FeSO_4_ and 9 mmol/L of ethanolic salicylic acid were added, followed by an appropriate amount of deionized water. Finally, 8.8 mmol/L H_2_O_2_ was added and shaken well. Next, 37 °C water bath was heated for 15 min and then removed. The absorbance was measured at 510 nm [25].

(4) $$\begin{array}{c} \mathrm{Hydroxyl}\;\mathrm{radical}\;\mathrm{scavenging}\;\mathrm{rate}\;\left(\%\right)=\left(1-\frac{D1-D2}{D3}\right) \end{array}$$

where D1 is the absorbance of the added sample, D2 is the absorbance of the sample without H_2_O_2_ and D3 is the absorbance of the blank control.

The data were nonlinearly fitted using Origin 2021 software to obtain the regression equation, and the concentration of the sample was calculated when the free radical scavenging activity reached 50% (IC50 value). The lower the IC50, the higher the free radical scavenging ability [26].

### 2.9. Bioinformatics and Molecular Docking

PeptideRanker (http://distilldeep.ucd.ie/PeptideRanker/, accessed on 24 December 2023) was used to score the biological activity of all the sequences identified by DSCP peptide sequences, and the sequences with scores greater than 0.8 were selected. The potential toxicity of peptides was predicted using ToxinPred (https://webs.iiitd.edu.in/raghava/toxinpred/, accessed on 24 December 2023). Expasy-Compute pI/Mw tool (https://web.expasy.org/compute_pi/, accessed on 24 December 2023) was utilized to predict the molecular weight of peptides. Peptides were mock-digested in ExPASy (https://web.expasy.org/peptide_cutter/, accessed on 24 December 2023) peptide cutter. The hydrophobicity of peptides was calculated by Pepdraw (http://www.pepdraw.com/, accessed on 24 December 2023). These online databases were used to predict the molecular weight, potential toxicity, anti-digestibility and hydrophobicity of the peptides [27]. The three-dimensional structure of Keap1 (PDB ID: 2FLU) was obtained from the PDB (https://webs.iiitd.edu.in/raghava/toxinpred/index.html, accessed on 24 December 2023) as a receptor. AutoDock Vina 4.2 was used to predict the optimal docking conformation of Keap1 binding and was visualized by Pymol 2.4.0 et al. [28]. Finally, the peptides with suitable molecular weight, non-toxicity, anti-digestion, high hydrophobicity and high binding energy to Keap1 were selected.

### 2.10. Statistical Analysis

All experiments were performed in at least three replicates. The data were analyzed using SPSS Statistics 26. Data were analyzed by one-way analysis of variance (ANOVA) with Tukey’s test. Differences were considered to be statistically significant when *p* < 0.05. Meanwhile, Origin 2022 was selected for graphic visualization.

## 3. Results and Discussion

### 3.1. Effect of Different Process Conditions on the Preparation of Ca-DSCP

**Figure 2:**
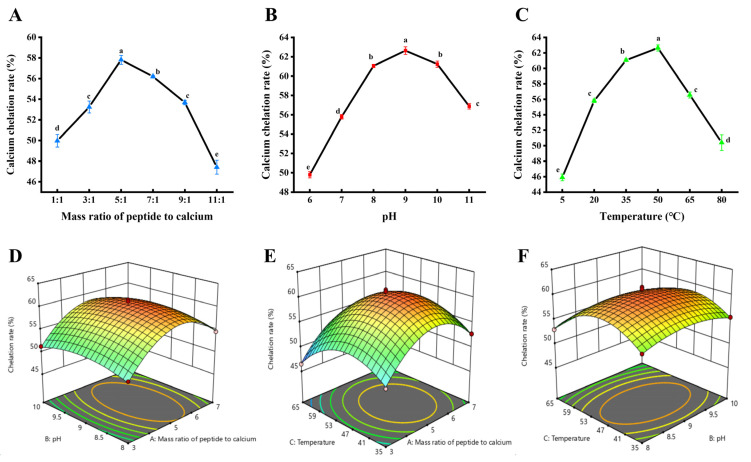
Effects of (**A**) mass ratio of peptide to calcium, (**B**) pH and (**C**) temperature on chelation rate. (**D**–**F**) Response surface plots of the effects of three factors on chelation rate. Different letters indicate significant differences between groups (*p* < 0.05).

As shown in Figure 2A, either a too large or too small mass ratio of peptide to calcium was not conducive to the optimal calcium binding amount. When the mass ratio of peptide to calcium was 1:1, many calcium ions were not involved in chelation, resulting in a low calcium binding amount. With the increase in mass ratio, the rate of calcium chelation increased continuously. This indicated that the increase in peptide can provide sufficient calcium binding sites for chelation reaction, which was conducive to the smooth progress of chelation reaction [29]. When the peptide calcium mass ratio continued to increase to 5:1, the DSCP and calcium ion binding reached saturation. The calcium chelation rate was no longer positively correlated with the mass ratio of peptide to calcium, and even showed a decreasing trend (*p* < 0.05). This result was similar to the binding of pig bone collagen peptides to calcium [18].

As shown in Figure 2B, with the increase in pH value, the calcium binding amount of Ca-DSCP showed a trend of first increasing and then decreasing. When pH value was 9, calcium chelation rate reached the maximum value (*p* < 0.05). This result may be due to the fact that too much H^+^ in the solution inhibits the ionization of H^+^ on the carboxyl group, and also affects the coordination of the amino group and Ca^2+^. Therefore, as the pH increases, the amount of calcium binding also increases. When the pH increased to 9, it was possible that calcium ions could react with the electron-donating group and compete for relative equilibrium. And the coordination ability of carboxyl groups and amino groups was strong. As the pH continued to increase, the amount of calcium binding decreased. This may be because under alkaline conditions, OH^-^ can compete with electron donor groups for metal ions to form hydroxyl complexes, resulting in hydroxide precipitation [29]. This was consistent with the results of collagen peptides derived from sources such as fish and sheep chelated with calcium [30,31].

As shown in Figure 2C, the calcium chelation rate of Ca-DSCP increased first and then decreased with the increase in temperature. At a chelation temperature of 50 °C, the calcium binding amount reached the maximum value (*p* < 0.05). However, when the temperature exceeded 50 °C, the amount of calcium binding dropped dramatically. This may be due to the fact that proper temperature promotes molecular movement, which facilitates chelation. However, too high a temperature may change the conformation of the protein, leading to a decrease in chelation [32]. This variation was similar to the preparation of peptides–calcium chelate derived from cattle bone [33].

According to the analysis of experimental data, the ratio of peptide–calcium mass, pH and chelation temperature had significant effects on calcium binding (*p* < 0.05). Subsequently, the appropriate peptide–calcium mass ratio (3:1, 5:1, 7:1), pH (8, 9, 10) and temperature (35, 50, 65 °C) were selected for subsequent response surface optimization experiments.

### 3.2. Response Surface Analysis for the Preparation of Ca-DSCP

With chelation rate as the dependent variable (response value Y), multiple regression fitting and significance analyses were performed on the data of Table 1, as shown in Table 2 and Figure 2D–F. The regression equation of the model was obtained as follows: Y = 60.35 + 1.66A − 0.1712B − 1.32C − 0.2000AB + 0.2950AC − 0.2325BC − 5.87A^2^ − 1.76B^2^ − 4.58C^2^. The multivariate correlation coefficient of the model R^2^ = 0.9857 indicated that the correlation is good. The Predicted R^2^ of 0.9572 was in reasonable agreement with the Adjusted R^2^ of 0.9674. The difference was less than 0.2. This indicated that the model fits well and the predicted value of the model fits well with the actual value. This can better reflect the real test results and fully explain the process. C.V.% = 1.46 was less than 10%, and the ratio precision of the effective signal to noise (Adeq Precision = 22.4975) exceeded the value of 4, which can be shown to be highly credible and accurate. The *p* value of primary term A was less than 0.001, indicating that the mass ratio of peptide to calcium had a significant effect on the chelation rate. The *p* value of C was less than 0.05, indicating that the effect of temperature on the chelation rate was significant (Figure 2D–F). The *p* values of quadratic terms A^2^ and C^2^ were less than 0.0001, which had extremely high significance. The *p* value of B^2^ was less than 0.05, which was significant. The *p* value of other interaction items was greater than 0.05, and the effect of each interaction on the chelation rate was not significant. The results showed that the change of chelation rate was relatively complex, and the influence of various factors on chelation rate was not a simple linear relationship. And the response surface effect was significant.

The optimal conditions were obtained as follows: mass ratio of peptide to calcium 5.277:1, pH 8.952 and temperature 47.928 °C. At this time, the chelation rate was 60.56%. For suitability of the experiment, the conditions were adjusted to mass ratio of peptide to calcium 5:1, pH 9 and temperature 48 °C. The chelation rate was (60.73 ± 1.54)%, which was not significantly different from the predicted value (*p* < 0.05). This value was higher than grape seed polypeptide calcium chelate (14.42%) and sheep bone collagen peptide-calcium chelate (42.57 ± 0.09%) [34,35]. At the same time, this value was higher than that of salmon ossein oligopeptides (SOOP). The calcium-chelating capacity of SOOP-Ca was 52.47 ± 0.08% [36]. The results showed that the chelation conditions optimized by the response surface methodology were reliable.

### 3.3. Amino Acid Composition

**Figure 3:**
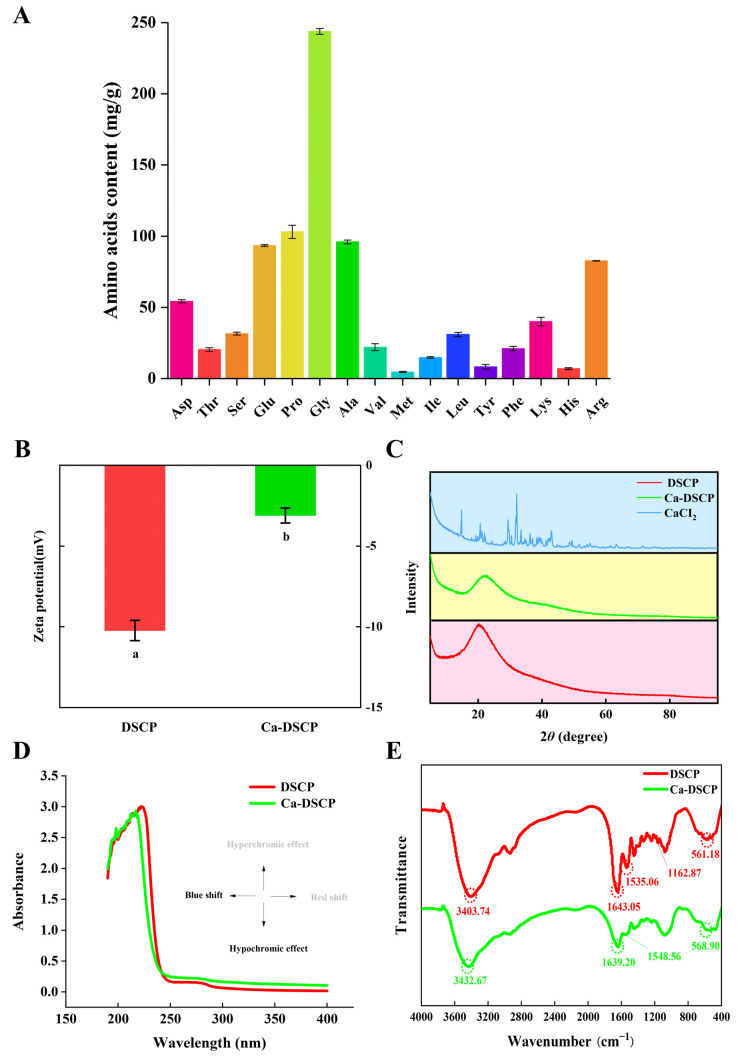
(**A**) Amino acid content of DSCP. (**B**) Zeta potential, (**C**) XRD, (**D**) UV-vis and (**E**) FTIR spectra of DSCP and Ca-DSCP. Different letters indicate significant differences between groups (*p* < 0.05).

The presence of specific amino acids in bioactive peptides has been reported to determine their antioxidant activity and chelating activity with calcium ions. For example, the negative charge and hydrophobic interactions of hydrophobic amino acids facilitate chelation with calcium and enhance their antioxidant activity [37]. Acidic and basic amino acids, for example, provide more active sites for calcium ion binding through electrostatic or ligand interaction. The amino acid composition of DSCP is shown in Figure 3A, with a total amino acid content of 874.07 ± 7.85 mg/g. Of these, the type, quantity and percentage of essential amino acids determine the final nutritional value of the amino acids. In DSCP, the content of essential amino acids was 153.94 ± 4.86 mg/g, accounting for 17.61 ± 0.44% of the total amino acids, making it a high-quality source of protein nutrition. In addition, the total content of acidic amino acids (Asp, Glu), basic amino acids (Lys, His, Arg), Gly, Ser and Leu was 62.23 ± 0.28%. It has been shown that these amino acids have high calcium-chelating activity [38]. The hydrophobic amino acid content of DSCP was 33.47 ± 0.19%. Due to the high hydrophobicity of the hydrophobic amino acid side chains, the peptide chains are folded, thus providing a spatial structure for the binding of the peptide chains to the metal ions, and also exposing more active sites to terminate the lipid chain reaction [39]. Based on this, DSCP appeared to be a potential source of calcium-binding peptides with antioxidant peptides.

### 3.4. Zeta Potential and XRD Analysis

One of the most useful parameters for studying electronic interactions in food systems is the zeta potential [40]. As can be seen in Figure 3B, the potentials of both DSCP and Ca-DSCP were negative. The absolute value of the potential of Ca-DSCP decreased significantly (*p* < 0.05) after DSCP was bound to calcium ions. This may occur by the electron transfer of DSCP to produce new compounds. And calcium ions can be chelated with positively charged groups. These results are consistent with the trend in previous studies [41].

XRD can be used to observe changes in the crystal structure of a substance. It can reflect the formation of complexes between organic ligands and metal ions, and this analytical method has been widely used for the reaction between peptides and calcium [42]. As can be seen in Figure 3C, CaCl_2_ has distinct crystallographic diffraction peaks. After the reaction of DSCP with CaCl_2_, the characteristic diffraction peak of CaCl_2_ disappeared. At the same time, the intensity of the diffraction peak of Ca-DSCP decreased, and the diffraction peak was shifted to the slight right. This indicated that CaCl_2_ was not physically mixed with DSCP, but formed a new compound by chelation, and the chelated calcium was amorphous. DSCP reacted chemically with calcium ions to form a new compound.

### 3.5. Structural Characterization of UV–vis and FTIR Analysis

UV–vis and FTIR analysis are often used to characterize the interactions between peptides and metal ions [43]. Figure 3D shows a significant band shift in the ultraviolet absorption spectra of DSCP and Ca-DSCP. After DSCP was chelated with calcium ions, the absorption peak shifted from 222 nm to 217 nm. DSCP had a weak absorption peak near 274 nm. Ca-DSCP had no absorption peak between 250–280 nm. This may be related to the n→π* electron transition of the carbonyl group (C=O) on the peptide bond and the π→π* transition of the aromatic amino acid residues in the peptide [44].

Figure 3E showed the different FTIR spectral curves of DSCP and Ca-DSCP. After chelating with calcium, the absorption peak of DSCP moved from 3403.74 cm^−1^ to 3432.67 cm^−1^. This change may be due to the formation of coordination bonds between nitrogen atoms and calcium ions by providing electron pairs. The absorption peak of amide I in DSCP was shifted from 1643.05 cm^−1^ to 1639.20 cm^−1^. This may be caused by C=O tensile vibration [45]. The absorption peak of amide II in DSCP was shifted from 1535.06 cm^−1^ to 1548.56 cm^−1^. The original absorption peak of DSCP disappeared at 1162.87 cm^−1^. The change may be caused by a C-N tensile vibration and N-H bending vibration. In addition, the peak at 561.18 cm^−1^ shifted to 569.75 cm^−1^, which may be caused by the tensile vibration of N-Ca. Previous studies have shown that nitrogen atoms can form ligand bonds with calcium ions by providing electron pairs [46]. Therefore, it can be inferred that chelation reactions occurred between DSCP and calcium ions to form chelates.

### 3.6. Acid–Base Stability and Thermal Stability Analysis

**Figure 4:**
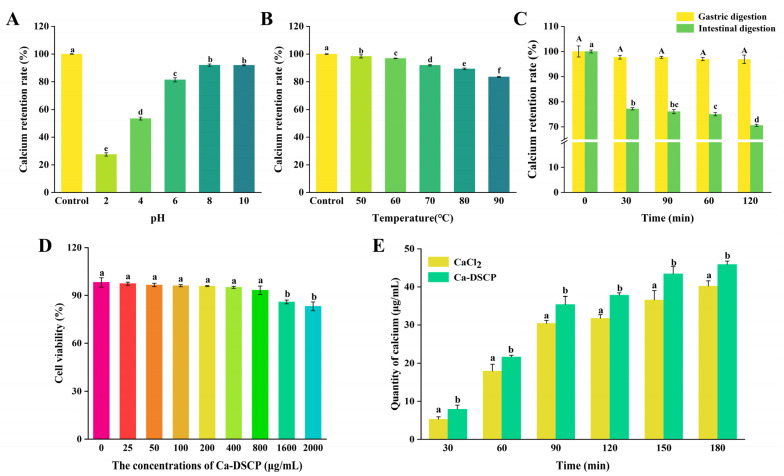
Effect of (**A**) pH, (**B**) temperature and (**C**) in vitro gastrointestinal digestion on the stability of Ca-DSCP. (**D**) Cytotoxicity of Caco-2 cells. (**E**) Calcium transport of CaCl_2_ and Ca-DSCP in Caco-2 cell monolayers. Different letters indicate significant differences between groups (*p* < 0.05).

Stability is an important index to evaluate the performance of calcium chelates [47]. As shown in Figure 4A, Ca-DSCP was relatively stable in the pH range of 6–10, and the calcium retention rate was more than 80%. However, when the pH of the system was 2.4, Ca-DSCP had a lower rate of calcium retention compared to the control group (*p* < 0.05). This may be due to the fact that excess H^+^ competes with Ca^2+^ for negative groups in an acidic environment, which affected the binding of Ca-DSCP [48]. As a result, peptide calcium chelates can generally remain stable in non-highly acidic and non-highly alkaline environments.

As shown in Figure 4B, with the increase in heat treatment temperature (50–90 ℃), calcium retention of Ca-DSCP remained stable for the first time, and then slowly decreased. Compared with the control group, the calcium retention rate was still above 80% (*p* < 0.05). It indicated that Ca-DSCP had a certain thermal stability. This may be because calcium ions interact with DSCP through coordination bonds to form an ordered, tight structure, making its spatial structure more stable [49].

### 3.7. In Vitro Gastrointestinal Digestion

The absorption and utilization of nutrients needs to be digested and absorbed by the gastrointestinal tract, and then digested and absorbed by the intestines. Its absorption and utilization rate are affected by pH and various digestive enzymes [50,51]. Therefore, it was necessary to evaluate the stability of Ca-DSCP in the gastrointestinal simulated digestion process. As shown in Figure 4C, there was no significant difference in calcium retention between 30 min, 60 min, 90 min and 120 min of simulated gastric digestion (*p* < 0.05) compared with the simulated gastric juice hydrolysis for 0 min (control preparations, pH = 2 without pepsin). This may be due to the pH of 2.0 in the simulated gastric digestive system, which, combined with the results of Ca-DSCP acid–base stability analysis, can be inferred to be mainly affected by pH. The results indicated that pepsin had little effect on Ca-DSCP and was only sensitive to pH. With the prolongation of action time, the calcium retention rate decreased significantly compared with the simulated intestinal fluid at 0 min (control preparations, pH = 7.5, without trypsin) (*p* < 0.05). This may be due to certain structural changes in Ca-DSCP in the gastric digestive environment, which affect its ability to bind calcium ions. But overall, after digestion, the calcium retention rate was still above 70%. The results indicated that Ca-DSCP was stable in the gastrointestinal environment and could promote the absorption of calcium by the body, thereby improving the bioavailability of Ca^2+^.

### 3.8. Caco-2 Cytotoxicity Test

As shown in Figure 4D, Caco-2 cell viability was significantly reduced after the Ca-DSCP culture at 1600 and 2000 μg/mL compared with no Ca-DSCP (*p* < 0.05). However, the viability of Caco-2 cells could be maintained at more than 80% after culture with different concentrations (25–2000 μg/mL) of Ca-DSCP. The results showed no toxic effect on Caco-2 cells in the range of 25–2000 μg/mL. Therefore, these concentrations can provide a reference for selecting the appropriate concentration for calcium transport experiments. Then, 200 μg/mL was selected for subsequent calcium transport experiments.

### 3.9. Caco-2 Cells Monolayer Calcium Transport Studies

The Caco-2 monolayer model has been successfully applied to the simulated absorption of proteins, peptides, amino acids and minerals [52]. As shown in Figure 4E, Ca-DSCP significantly increased calcium transport activity compared with the CaCl_2_ group in each time period (*p* < 0.05). In terms of calcium transport, the calcium transport in the Ca-DSCP group was 7.87 ± 1.11, 21.62 ± 0.46, 35.36 ± 2.15, 37.80 ± 0.64, 43.45 ± 1.87 and 45.89 ± 0.92 μg/mL and these were 1.49, 1.30, 1.16, 1.19, 1.19 and 1.14 times higher than those in the CaCl_2_ group, respectively. Overall, Ca-DSCP facilitated a gradual increase in calcium ion transport with increasing time. It indicated that Ca-DSCP was sensitive to the time of calcium ion transport. The results showed that Ca-DSCP effectively promoted calcium transport and calcium absorption increased in a time-dependent manner.

### 3.10. Analysis of Oxidative Stress Indicators

**Figure 5:**
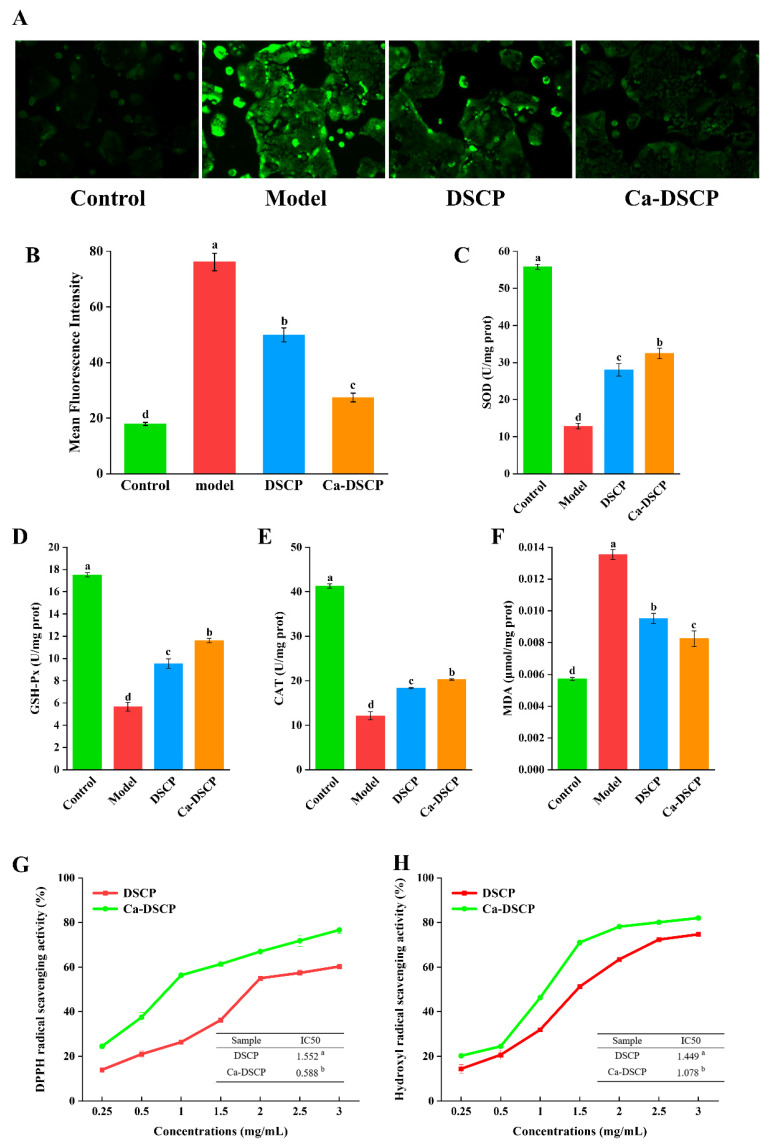
(**A**) Fluorescent images of ROS. (**B**) Mean fluorescence intensity of ROS. (**C**) SOD, (**D**) GSH-Px and (**E**) CAT activity. (**F**) MDA content. (**G**) DPPH radical scavenging capacity and (**H**) hydroxyl radical scavenging activity of DSCP and Ca-DSCP. Different letters indicate significant differences between groups (*p* < 0.05).

Under normal physiological conditions, free radicals in the body are maintained within normal physiological ranges. However, stimulation by external factors can lead to an imbalance between oxidative and antioxidant responses in the body [53]. When excess free radicals are produced in the body, they damage cells and tissues, causing loss of activity of intracellular lipids, metabolic enzymes and functional proteins. Endogenous cellular antioxidant enzymes are important players in the oxidative stress process (SOD, GSH-Px and CAT) [54]. Among them, oxidative stress is a phenomenon of ROS accumulation in cells and MDA is a major product of lipid peroxidation damage. As shown in Figure 5A,B, the mean fluorescence intensity of ROS was significantly increased in the model group compared to the control group (*p* < 0.05). It was significantly attenuated in cells in the DSCP versus Ca-DSCP group compared to the model group (*p* < 0.05). As shown in Figure 5C–E, the enzymatic activities of SOD, GSH-Px and CAT were significantly reduced (*p* < 0.05) in Caco-2 cells after treatment with H_2_O_2_. The activities of SOD, GSH-Px and CAT enzymes in oxidatively damaged cells were increased after treatment with DSCP and Ca-DSCP groups compared to the model group (*p* < 0.05). Thus, it can be hypothesized that DSCP and Ca-DSCP increased the activity of antioxidant enzymes in damaged Caco-2 cells and reduced the accumulation of peroxidation products. The MDA content in Caco-2 cells was elevated after treatment with H_2_O_2_ (*p* < 0.05). Compared with the model group, the intracellular MDA content in oxidatively damaged cells was significantly reduced after treatment with DSCP and Ca-DSCP groups (*p* < 0.05). The results showed that DSCP and Ca-DSCP groups could reduce the intracellular MDA level of oxidative damage (Figure 5F). The results showed that DSCP and Ca-DSCP had antioxidant activity in cellular assays.

### 3.11. DPPH Radical Scavenging Capacity of DSCP and Ca-DSCP

The DPPH method is widely used to quantitatively determine the antioxidant capacity of biological samples, foods, etc. When there is a free radical scavenger, its absorption gradually disappears due to its single-electron pairing, and its fading degree is quantitatively related to the number of electrons it accepts, resulting in a decrease in the absorbance of the reaction system [55]. As can be seen from Figure 5G, the scavenging rate of DPPH free radicals by DSCP and Ca-DSCP was enhanced with the increase in concentration at a mass concentration of 0.25~3 mg/mL. Among them, IC50 is an important index to evaluate the antioxidant capacity, and the smaller the value, the stronger the antioxidant capacity of the tested sample [56]. The IC50 values of DSCP and Ca-DSCP for DPPH radical scavenging were 1.552 and 0.588 mg/mL, respectively. From the IC50, the IC50 value of Ca-DSCP on DPPH radical scavenging ability was significantly lower than that of DSCP (*p* < 0.05). These results indicated that the DPPH free radical scavenging ability of Ca-DSCP was higher than that of DSCP after DSCP was chelated with calcium. This was consistent with the results of Wenjuan Qu et al., which showed that the calcium-chelating zein peptide had a higher scavenging ability of DPPH free radicals than the zein peptide through a Ca^2+^ chelation reaction [8]. It may be that a chelation reaction was conducive to improving the antioxidant capacity of DSCP.

### 3.12. Hydroxyl Radical Scavenging Capacity of DSCP and Ca-DSCP

The hydroxyl radical is the most active and oxidizing free radical. And it has a very strong ability to gain electrons. It is also believed to be the most direct free radical that triggers tissue cell lesions in the body, leading to diseases and accelerated aging. Therefore, it is often used to evaluate the antioxidant capacity of samples [57]. As shown in Figure 5H, both DSCP and Ca-DSCP showed strong hydroxyl scavenging activity. In a certain range of mass concentration, the hydroxyl scavenging rate increases with the increase in mass concentration; IC50 was 1.449 and 1.078 mg/mL, respectively. According to IC50, the IC50 value of hydroxyl radical scavenging by Ca-DSCP was significantly lower than that of DSCP (*p* < 0.05). These results indicated that the ability of DSCP to scavenge hydroxyl radicals was significantly enhanced after chelating with calcium. This may be due to the fact that the coordination of calcium ions with the peptide alters the charge distribution of the peptide so that it provides electrons to the free radicals, thereby eliminating the free radicals.

### 3.13. Bioinformatics and Molecular Docking

**Figure 6:**
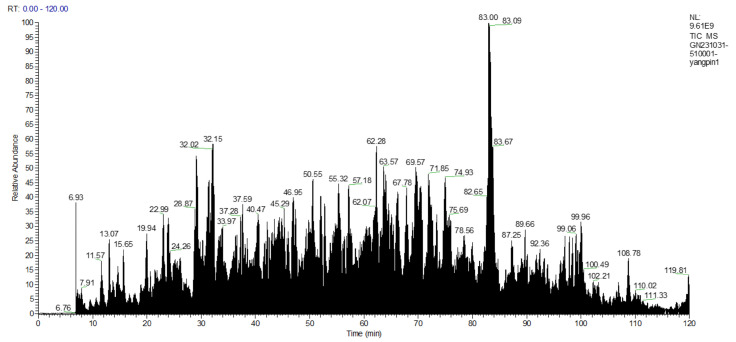
Total ion chromatogram of DSCP.

**Figure 7:**
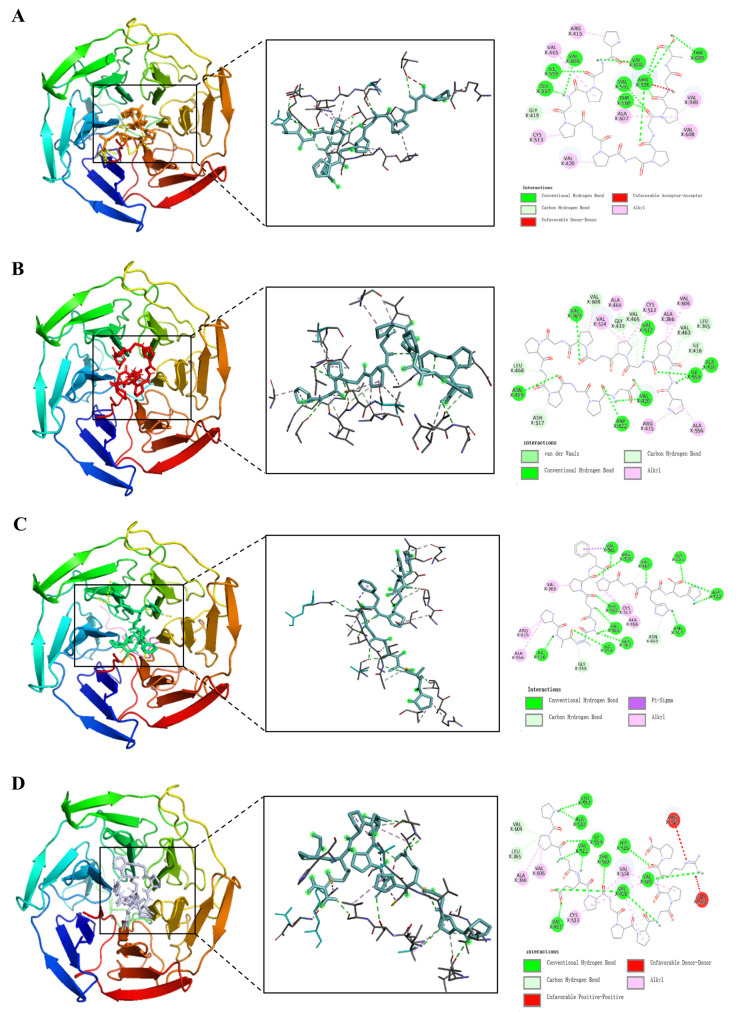
Molecular docking visualization of Keap1 with the top 8 peptides with the highest binding energy (**A**–**D** are shown in this Figure, and E–H are shown in Figure S1).

Virtual screening is a type of screening technology composed of bioinformatics, molecular docking and other technologies [58]. The amino acid sequence of DSCP was determined by high-performance liquid chromatography–mass spectrometry (HPLC-MS). A total of 11,210 peptides were obtained (Table S1). Figure 6 shows the total ion chromatogram. DSCP was virtually screened. Screening of peptide sequences for this study required appropriate molecular weight, non-toxicity, resistance to digestion, high hydrophobicity, a confidence level ≥ 95% and high binding energy to Keap1. Therefore, bioinformatics was used to analyze DSCP, and then molecular docking was used to evaluate its binding ability to Keap1 [59]. Keap1 acts as a receptor for oxidative stress regulation target gene switches, and is a molecular switch that controls the Nrf2 signaling pathway [60]. The Keap1/Nrf2 signaling pathway is the central hub for maintaining redox balance in vivo, and is closely related to inflammation regulation, cell growth and differentiation, and metabolism [61]. The purpose of studying the binding ability of DSCP and Keap1 is to screen out peptides with antioxidant activity in DSCP, and to provide support for verifying the antioxidant activity of Ca-DSCP. This study can also provide a basis for further exploration of the antioxidant mechanism of DSCP and Ca-DSCP. The molecular docking results are expressed as Vina fraction (kcal/mol). The Vina score can reflect the binding affinity of Keap1 to the peptide, which is negative. Therefore, the lowest Vina score represents the highest binding affinity [62]. Table 3, Figure 7 and Figure S1 show the top eight peptides with the highest binding energy (A: PGPGPGPGPGPGA, B: PGPGPGPGPGPGPG, C: PAAGGPFPGHH, D: PPGEPGPPGPRP, E: PGPGPGPGQAPPGG, F: PGPSPGPGPSPG, G: PGPAGPPGAPG, H: MPGPGPGPGPGPGP). These peptides had a certain binding ability to Keap1, and the main interaction forces were the Conventional Hydrogen Bond, Carbon Hydrogen Bond, Unfavorable Positive–Positive and Unfavorable Donor–Donor. Among them, hydrogen bonding was the main force to stabilize the ligand-acceptor complex. Residues of Keap1 such as GLY367, ILE559, VAL606, VAL604, VAL561, ARG326, THR560, THR609, LEU468, ASN469, VAL369, ASP422, ALA510 and ASN517 formed hydrogen bonds with the peptides of DSCP (Table 3 for details). In summary, these peptides can spontaneously interact with the active site of Keap1 protein, which has the potential for further study.

## 4. Conclusions

In this study, the optimum preparation parameters of Ca-DSCP were obtained by single-factor and response surface experiments. The fraction of bioavailability of calcium ions in Ca-DSCP was determined by acid–base stability and simulated gastrointestinal digestion in vitro. The results showed that it had excellent stability and good bioavailability under different conditions. In vitro experiments revealed that DSCP can act as a calcium carrier to increase calcium transport in Caco-2 cells, but the manner in which it promoted calcium transport was unclear. In addition, DSCP and Ca-DSCP were found to have antioxidant activity by measuring oxidative stress-related indices (ROS, SOD, GSH-Px, CAT and MDA) in cellular assays. Based on bioinformatics and molecular docking techniques, DSCP that could directly inhibit Keap1-Nrf2 interaction was screened. We will further screen and characterize peptides with high antioxidant activity, and analyze the mechanism of antioxidant action and the structure–activity relationship. In short, DSCP and Ca-DSCP could have broad development prospects and great application potential in the direction of calcium supplementation and antioxidant functional foods.

## Figures and Tables

**Table 1:** Experimental data for chelation rate from the Box–Behnken Design.

No.	A-Mass Ratio of Peptide to Calcium	B-pH	C-Temperature	Chelation Rate %
1	5:1	9	50	61.52
2	3:1	9	65	46.48
3	7:1	9	65	50.83
4	7:1	8	50	54.58
5	5:1	9	50	59.77
6	7:1	10	50	53.75
7	5:1	9	50	61.19
8	7:1	9	35	52.74
9	3:1	10	50	51.27
10	5:1	10	65	52.27
11	5:1	8	65	52.99
12	3:1	8	50	51.30
13	5:1	8	35	55.30
14	5:1	10	35	55.51
15	5:1	9	50	59.11
16	3:1	9	35	49.57
17	5:1	9	50	60.16

**Table 2:** Variance and significance analysis of response surface regression equations.

Source	Sum of Squares	df	Mean Square	F-Value	*p*-Value	Significant
Model	305.62	9	33.96	53.59	<0.0001	**
A-Mass ratio of peptide to calcium	21.95	1	21.95	34.63	0.0006	**
B-pH	0.2244	1	0.2244	0.3542	0.5705	
C-Temperature	13.97	1	13.97	22.04	0.0022	*
AB	0.1681	1	0.1681	0.2653	0.6224	
AC	0.3782	1	0.3782	0.5968	0.4651	
BC	0.2209	1	0.2209	0.3486	0.5735	
A^2^	144.90	1	144.90	228.64	<0.0001	**
B^2^	12.95	1	12.95	20.43	0.0027	*
C^2^	88.18	1	88.18	139.14	<0.0001	**
Residual	4.44	7	0.6337			
Lack of fit	0.4515	3	0.1505	0.1511	0.9238	not significant
Pure error	3.98	4	0.9962			
Cor total	310.06	16				

* Significant (*p* < 0.05), ** highly significant (*p* < 0.001).

**Table 3:** The first 8 peptides with the highest molecular binding energy of DSCP and Keap1 and partial physical and chemical properties.

Peptide	Mass (Da)	Toxicity	Hydrophobicity (Kcal/mol)	Post-Digestive Fragments	Binding Energy (kcal/mol)	Hydrogen Bonds		Structural Formula
						Quantity	Binding Sites	
A	1014.11	None	16.14	PGPGPGPGPGPGA	−12.1	13	VAL561, VAL604, VAL606, ILE559, GLY367, GLY419, THR,560, THR609, ARG326	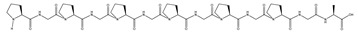
B	1097.2	None	16.93	PGPGPGPGPGPGPG	−11.9	18	LEU365, LEU468, ASN469, ASN517, VAL369, VAL420, VAL463, VAL465, VAL512, VAL608, GLY419, ILE416, ILE559, ASP442, ALA510	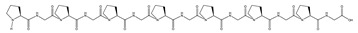
C	1044.14	None	15.72	PAAGGPFPGHH	−11.2	17	ILE416, ILE559, GLY367, GLY423, GLY558, VAL467, VAL561, VAL606, THR560, ASN469, ASN517, ASP422, ARG362	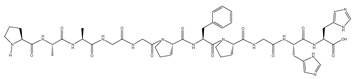
D	1154.29	None	17.77	PPGEPGPPGPRP	−11.0	14	VAL420, VAL467, VAL512, VAL516, VAL604, LEU365, LEU557, ALA510, ILE559, THR560, HIS516,	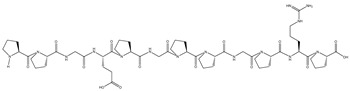
E	1142.24	None	16.91	PGPGPGPGQAPPGG	−10.9	6	ILE559, VAL420, VAL606, GLY367, ARG326	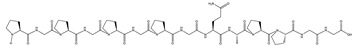
F	1003.08	None	14.26	PGPSPGPGPSPG	−10.9	13	VAL418, VAL420, VAL463, VAL465, VAL467, ASP422, THR560, ARG326	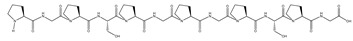
G	873.96	None	14.2	PGPAGPPGAPG	−10.9	8	VAL369, VAL418, VAL420, VAL463, VAL465, ASP422, ASN469, IEU365	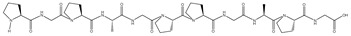
H	1171.34	None	15.11	MPGPGPGPGPGPGP	−10.8	15	ARG326, GLN563, GLY367, GLY419, GLY564, ASN469, VAL512, ILE559, LEU557, ALA510, VAL606	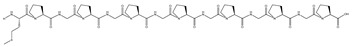

## Data Availability

The original contributions presented in the study are included in the article/Supplementary Materials.

## Supplementary Materials

The following supporting information can be downloaded at: https://www.mdpi.com/article/10.3390/nu16162585/s1, Figure S1: Molecular docking visualization of Keap1 with DSCP with the highest binding energy; Table S1: Peptide sequences of DSCP.
