# Supplementary material for: Deer Skin Collagen Peptides Bound to Calcium: In Vitro Gastrointestinal Simulation of Digestion, Cellular Uptake and Analysis of Antioxidant Activity
Source: Nutrients. 2024 Aug 6;16(16):2585. doi: 10.3390/nu16162585 (PMC11357615; doi:10.3390/nu16162585)
Supplement: Supplementary file 1 [file nutrients-16-02585-s001.zip › nutrients-3115118-supplementary.pdf]

Supplementary Material

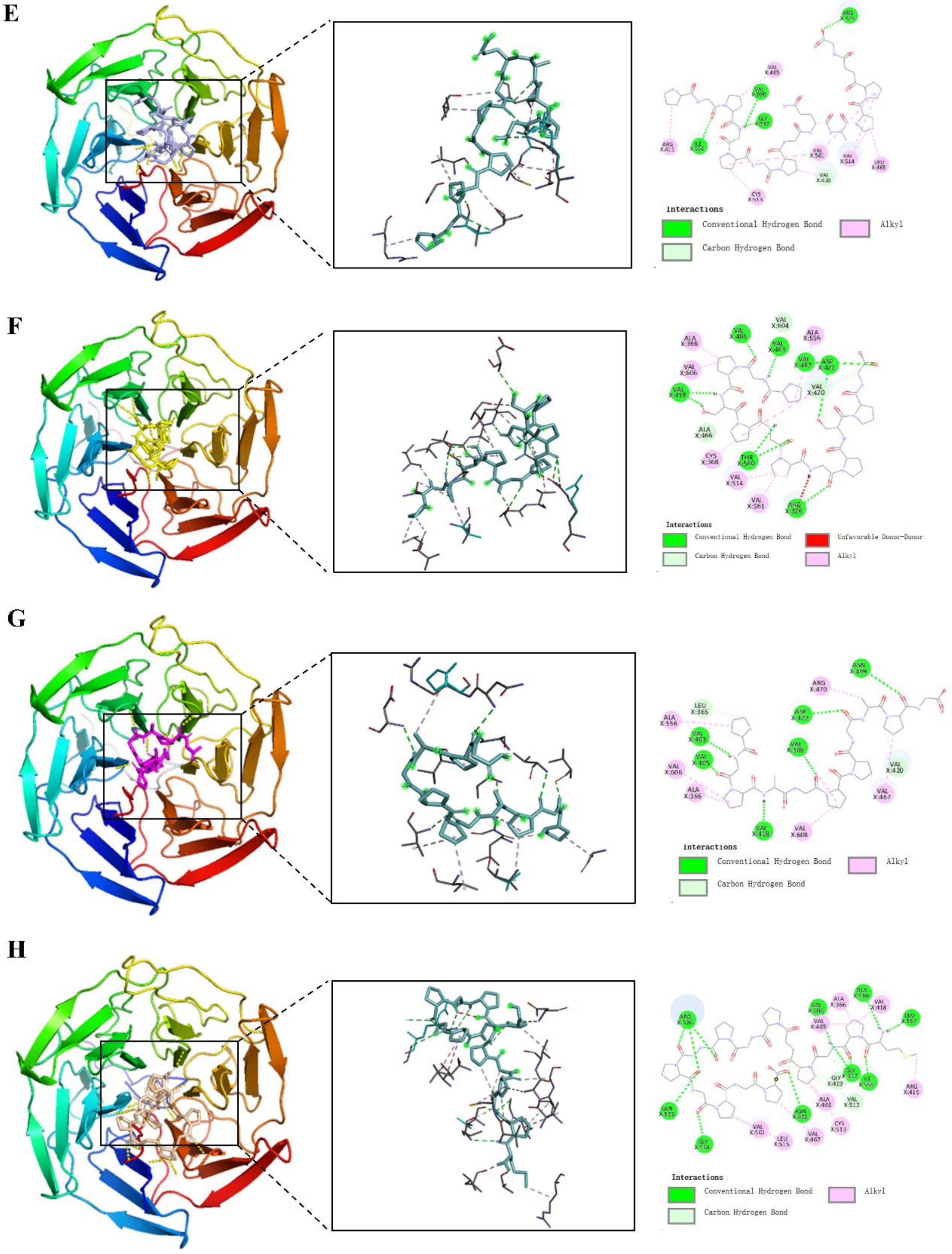

Figure S1. Molecular docking visualization of Keap1 with DSCP with the highest binding energy.

Table S1. Peptide sequences of DSCP.

No.	Annotated Sequence	Mass (Da)
1	[Q].SMVGAD.[V]	579.24429
2	[V].FPSIVG.[R]	619.34499
3	[P].GPAGAVGP.[A]	625.3304
4	[R].GPAWAGP.[E]	655.31984
5	[I].KGAAGRP.[L]	656.38383
6	[S].AAPATSF.[T]	664.33007
7	[K].GPGQGLH.[R]	665.33655
8	[D].GPLDGAH.[V]	666.32056
9	[G].PGAGPAAGA.[A]	668.33621
10	[A].PGAAAPGAG.[P]	668.33621
11	[E].PGAGARGS.[H]	672.34236
12	[M].QPLVMA.[S]	674.35417
13	[A].ADPAPAH.[A]	678.32056
14	[G].SPGPQTP.[G]	683.33588
15	[E].AAGVSGPAG.[G]	686.34678
16	[S].GPASAPST.[C]	687.3308
17	[C].PGARKC.[V]	688.3559
18	[L].SPGPGFQ.[D]	689.32532
19	[A].GVGVDML.[S]	690.34909
20	[G].GPSPAPAP.[R]	693.35662
21	[T].PGPFGGAP.[G]	699.34605
22	[-].MAPGGGGGRA.[L]	699.35326
23	[A].PGSPGTLA.[L]	699.36718
24	[V].GAPGVEGL.[A]	699.36718
25	[E].QEVGALS.[L]	703.3621
26	[D].PGAGAFQG.[Y]	704.33621
27	[-].MGPGSHHL.[S]	704.34745
28	[A].AAAATGGSV.[L]	704.35734
29	[A].AAAAASATA.[F]	704.35734
30	[Q].PGPSGPPP.[R]	705.35662
31	[S].PGGRSHP.[P]	707.35835

32	[T].ATLAGAGF.[T]	707.37227
33	[M].KGHSGAPG.[P]	710.35801
34	[-].MPGPQGAGGA.[P]	711.34203
35	[G].PGGSTHR.[R]	711.35326
36	[A].GPEALAPG.[G]	711.36718
37	[G].PGSGPARA.[P]	712.37366
38	[G].PGPGSGGGAG.[T]	713.32129
39	[P].GPDAPISG.[P]	713.34645
40	[EQ].AAAEPPTG.[LS]	713.34645
41	[A].GPSPASTP.[A]	713.34645
42	[P].AASASGLH.[T]	713.35768
43	[K].QRGQAGP.[Q]	713.36891
44	[G].PAVEQAV.[R]	713.38283
45	[A].PGSPSRGG.[T]	714.35293
46	[C].GAPGARGE.[A]	714.35293
47	[K].PGPAGMKG.[E]	714.36032
48	[Q].GPPGHPGP.[P]	715.3522
49	[A].AAAGAAAASG.[G]	717.35259
50	[S].PGSSRAGS.[K]	718.34784
51	[P].PGSTASLS.[T]	719.35701
52	[A].PGSISTGT.[T]	719.35701
53	[E].GPGTIEF.[V]	720.35628
54	[SQ].PGPGAPGPA.[AR]	720.36752
55	[P].GPGQPPAP.[S]	720.36752
56	[P].GPAGPPGAP.[G]	720.36752
57	[R].PGAPGPPAG.[C]	720.36752
58	[-].MAAAPSHPA.[G]	721.36276
59	[K].GPPSAFF.[L]	722.3508
60	[-].MQPGSHR.[I]	723.35326
61	[S].QASGAGAY.[R]	724.32604
62	[H].GATPNPPA.[A]	724.36243
63	[L].PGSQAPPA.[Q]	724.36243
64	[-].MQAPGGVPG.[EV]	724.36243
65	[KP].GPSPGPGR.[L]	724.37366

66	[P].GPGAGPAAGA.[A]	725.35768
67	[A].AAAPAGGNP.[E]	725.35768
68	[H].VENAGVH.[S]	725.35768
69	[V].GGGPARGGP.[D]	725.36891
70	[P].AAAPTPTP.[P]	725.38283
71	[G].AAAELPGP.[A]	725.38283
72	[E].AAPAGLEP.[K]	725.38283
73	[A].AAAASAQH.[S]	726.35293
74	[K].AAGDHAKG.[L]	726.35293
75	[P].AGPWAQP.[E]	726.35695
76	[E].PGAGAEKP.[S]	726.37808
77	[A].GSPVGDVP.[F]	727.3621
78	[-].MAAAAAAGPGA.[P]	727.37333
79	[L].PSGPGPGC.[R]	728.3032
80	[A].GSPATAAGP.[A]	728.35734
81	[-].MATAPGSQP.[L]	728.35734
82	[G].AAEAAQAP.[A]	728.35734
83	[V].ASAPASGPA.[V]	728.35734
84	[FL].PGSPGEKG.[E]	728.35734
85	[G].GPSGGPTVG.[G]	728.35734
86	[Y].PGSPGLSGG.[I]	728.35734
87	[G].PGKGGPES.[I]	728.35734
88	[S].AVAGASPR.[V]	728.40496
89	[A].KPFSIH.[F]	728.40899
90	[S].QPGGHPH.[N]	729.3427
91	[-].MAAAAAGAGAG.[A]	729.35259
92	[P].GPSGLGGAGG.[L]	729.35259
93	[P].GPSPGAML.[G]	729.35999
94	[A].AAGGRSGPG.[R]	729.36383
95	[W].RGGSGAGPA.[G]	729.36383
96	[A].QRNSGPA.[L]	729.36383
97	[K].PGGGQGRT.[A]	729.36383
98	[G].QPLSSPT.[T]	729.37775
99	[S].PGAGGAFPG.[L]	730.35187

100	[Q].PGSPGMKG.[E]	730.35524
101	[L].GPPSSLSS.[W]	731.35701
102	[C].GAVGTGQAA.[K]	731.36824
103	[A].AAAAAAASGA.[T]	731.36824
104	[L].GPSGPAFV.[Q]	731.37227
105	[W].SPGPGAFT.[T]	733.35153
106	[K].KDDAITA.[Y]	733.37266
107	[I].PGSPLYT.[I]	734.37193
108	[K].QRGSGAC.[I]	735.32025
109	[A].SPGTLSSS.[S]	735.35192
110	[P].PGAGRFM.[A]	735.36066
111	[L].AAADLFAG.[V]	735.36718
112	[G].QVGQAAY.[S]	736.36243
113	[Q].KDMVFP.[N]	736.36982
114	[A].PAQNGIH.[P]	736.37366
115	[G].GPSPGPPE.[H]	737.34645
116	[A].GAGPGPGPGA.[P]	737.35768
117	[G].PGGAGANPP.[W]	737.35768
118	[V].PGATAHSP.[L]	737.35768
119	[G].AGGPAGPGGP.[G]	737.35768
120	[I].GPSIPGIP.[G]	737.41922
121	[Q].PGVGSGPAP.[Q]	738.37808
122	[R].GPSPGPAR.[K]	738.38931
123	[V].GPGAGRGAP.[D]	739.38456
124	[G].PSGPGPGDG.[C]	740.32096
125	[L].PGSPFHV.[P]	740.3726
126	[S].AARGGTAH.[G]	740.37981
127	[A].KEAAGPAP.[S]	740.39373
128	[P].GPGGAGAAPS.[G]	741.35259
129	[D].AGSAGPQGP.[G]	741.35259
130	[P].GQLTHW.[H]	741.36785
131	[Q].GPGRGGGGA.[G]	742.35908
132	[T].PGSTAAAPA.[G]	742.37299
133	[S].AAVSGPSPG.[Q]	742.37299

134	[P].PGSAVAGPS.[S]	742.37299
135	[P].PGSGTLPGG.[S]	742.37299
136	[R].GPSVLAQA.[P]	742.40938
137	[Q].PGAVSPSK.[I]	742.40938
138	[-].MAATAPNAGA.[S]	743.36824
139	[M].AATAPNAGA.[S]	743.36824
140	[E].AAGVSGPAGG.[R]	743.36824
141	[A].KGEQGPAG.[H]	743.36824
142	[E].TAPAAAGGQ.[M]	743.36824
143	[I].PGAGGTQR.[L]	743.37948
144	[P].GPAPGFAAG.[Q]	744.36752
145	[A].GPAGAFPGA.[L]	744.36752
146	[P].AAAPSTTGA.[L]	746.36791
147	[A].TAGSPATAA.[G]	746.36791
148	[F].KYLSVH.[L]	746.41955
149	[V].GPAGGNQM.[L]	747.30901
150	[G].AAGGATAASA.[A]	747.36316
151	[-].MAAAATMAAA.[A]	748.3658
152	[Y].GGGLGAGFGG.[G]	749.35768
153	[L].GPSLTSTS.[G]	749.36757
154	[A].QAISGSTS.[A]	750.36282
155	[T].AGRFGWG.[A]	750.36818
156	[P].GPASPGPAP.[R]	750.37808
157	[Q].PGPSGPPAA.[P]	750.37808
158	[T].PSGPAPAGP.[A]	750.37808
159	[G].PGPSAGPAP.[R]	750.37808
160	[G].GPPSAGPAP.[V]	750.37808
161	[P].PGAGSPPPA.[P]	750.37808
162	[K].KPGAGLAH.[A]	750.4257
163	[F].PSSPGLGH.[K]	751.37333
164	[H].PGQAPPGGA.[S]	751.37333
165	[P].PGQQPPQ.[A]	751.37333
166	[E].PGPGGAGAAP.[S]	751.37333
167	[G].APGGAAPGGP.[G]	751.37333

168	[R].PGPGAGPAAG.[A]	751.37333
169	[P].PAGGGGSIH.[D]	752.36858
170	[F].AARGGHGQ.[Q]	753.37506
171	[P].GPSPTTPP.[Q]	753.37775
172	[L].PANGKMH.[S]	754.36647
173	[-].MQAPGGVPS.[E]	754.37299
174	[A].PGGVSGPSP.[A]	754.37299
175	[P].GELGPGAPG.[H]	754.37299
176	[G].PGSPGVPGS.[P]	754.37299
177	[-].MATAPGPAGI.[A]	754.40938
178	[M].PGSGVQGPG.[E]	755.36824
179	[S].PGAGGGGRAG.[R]	756.37473
180	[G].GARGGAGGGP.[S]	756.37473
181	[V].AGAGWAAGP.[V]	757.36276
182	[A].AAAEAVPE.[S]	757.37266
183	[R].SPGLEASP.[P]	757.37266
184	[R].PGPSDLAT.[A]	757.37266
185	[-].MAAAAASAPQ.[Q]	757.38389
186	[E].RGAGVSGGP.[S]	757.39513
187	[N].IEGPDVK.[I]	757.40905
188	[R].SSPAVGSPG.[N]	758.36791
189	[V].AAGAAAGAAAG.[A]	758.37914
190	[-].MAAAAAGAGAGA.[A]	758.37914
191	[C].PGSGERR.[I]	758.39038
192	[G].AAARGDAGA.[P]	759.37439
193	[K].KGPAHLH.[Y]	759.42603
194	[L].GPAGGGNSGS.[S]	760.32202
195	[Q].QQAAGGND.[S]	760.32202
196	[G].PGGPQHAP.[F]	760.37366
197	[Y].PGAPHPGGA.[P]	760.37366
198	[P].GPGRTSW.[R]	760.37366
199	[P].PGAPPHAGG.[P]	760.37366
200	[S].SVPGATAAS.[A]	760.38356
201	[E].KPGHPQP.[L]	760.41005

202	[D].KVGTDQL.[E]	760.41994
203	[E].GPANSSKT.[C]	761.37881
204	[V].GLAGTGAASG.[LI]	761.37881
205	[A].GPPSGIFS.[Q]	761.38283
206	[-].MPGPSPGLH.[R]	761.39406
207	[R].PGSPGPPGP.[L]	762.37808
208	[R].PGPSPGPGP.[S]	762.37808
209	[T].ATPAQMK.[E]	762.38145
210	[-].MGPPGGQGPP.[G]	763.37333
211	[P].GPGGPGPAGP.[M]	763.37333
212	[R].GPGGPQPGP.[R]	763.37333
213	[V].GPGPGGPGPA.[G]	763.37333
214	[M].GPPGGQGPP.[G]	763.37333
215	[G].PGPGGPGPAG.[P]	763.37333
216	[P].KAGRGQF.[S]	763.42095
217	[S].PGSGPGRH.[P]	764.37981
218	[G].PGATPPGAP.[A]	764.39373
219	[G].PGAPTGPPA.[P]	764.39373
220	[P].PGPSGVPPG.[M]	764.39373
221	[P].KHFHVP.[D]	764.42022
222	[P].GPIGVPGPA.[G]	764.43012
223	[Q].GPVGLPGPA.[G]	764.43012
224	[R].GAAAAASSC.[Q]	765.31958
225	[E].AGGAAPGQH.[R]	765.36383
226	[G].GPAGGHRN.[G]	765.37506
227	[K].PGKSEFT.[R]	765.37775
228	[S].AAPATSFT.[S]	765.37775
229	[A].PGAAAPGAGP.[G]	765.38898
230	[K].GAPGNVGPP.[G]	765.38898
231	[I].PGSPGHTI.[Y]	765.38898
232	[G].APGNVGPPG.[P]	765.38898
233	[A].PAGPSHIS.[Q]	765.38898
234	[G].AAAPGAGPGP.[A]	765.38898
235	[R].RQGGPPGP.[G]	765.40021

236	[P].AGPGEPAAP.[F]	766.37299
237	[K].GAPAEPGAP.[R]	766.37299
238	[W].AGGPEPAAP.[P]	766.37299
239	[H].SPPSGAPGP.[P]	766.37299
240	[S].SPPSPGPQ.[Q]	766.37299
241	[S].SPGGPSPAP.[A]	766.37299
242	[R].PSPASGPGP.[G]	766.37299
243	[G].PGAGAAPEP.[P]	766.37299
244	[V].QGKEPAH.[L]	766.38423
245	[T].GPLAGAGSH.[E]	766.38423
246	[P].GPRGDAPP.[F]	766.38423
247	[G].RGRGSPH.[L]	766.40669
248	[S].KAQPSVH.[T]	766.42061
249	[K].GPGNKSHA.[K]	767.37948
250	[L].PGGFSRF.[Q]	767.3835
251	[P].GAGPMVVH.[C]	767.38687
252	[G].RQGGGGVH.[A]	767.39071
253	[S].SGPSQVPP.[P]	768.38864
254	[G].SRNPTPP.[I]	768.39988
255	[V].PGAGSVPAI.[Q]	768.42503
256	[G].PGGEAGPGGA.[L]	769.34751
257	[M].AAGGHRGSG.[A]	769.36997
258	[I].QPEPVEA.[Q]	769.37266
259	[-].MVGPNGTAGP.[V]	769.38389
260	[Q].GPKGEQGP.[P]	769.38389
261	[T].GVGGPSPGAA.[G]	769.38389
262	[G].GPQNLSGP.[G]	769.38389
263	[G].APGAGAASPA.[E]	769.38389
264	[L].GSPVGQAGP.[D]	769.38389
265	[G].AGPAGAGGAAA.[V]	770.37914
266	[A].AAAAGGAGPGA.[G]	770.37914
267	[A].AAAGGQQAP.[E]	770.37914
268	[L].GPAARDSP.[P]	770.37914
269	[Q].GPAGQGGVQ.[V]	770.37914

270	[P].GPAQTPSL.[L]	770.40429
271	[G].PGPGATATV.[T]	770.40429
272	[P].SAPSAAAPV.[P]	770.40429
273	[E].GTSAPAGGPG.[S]	771.36316
274	[C].SPGSPRGGG.[R]	771.37439
275	[G].PGGPSRSN.[V]	771.37439
276	[V].ARAGEGPGG.[G]	771.37439
277	[G].AGSGVPFH.[W]	771.37841
278	[P].PGKMGPQG.[T]	771.38178
279	[P].PGPSLAET.[G]	771.38831
280	[E].AALSPESP.[N]	771.38831
281	[PT].AASPEISP.[QA]	771.38831
282	[L].LSGTAHSV.[N]	771.39954
283	[Y].DFGFDGD.[F]	772.27843
284	[P].TAGPSAAPT.[E]	772.38356
285	[I].GPGAGGAASK.[T]	772.39479
286	[MV].PGGAGGQDD.[P]	773.30604
287	[S].PGAGGVQST.[S]	773.37881
288	[A].AARGGEAAA.[E]	773.39004
289	[E].KFHNEV.[A]	773.39406
290	[I].GPSRSRGG.[A]	773.40127
291	[E].AAAAAGGGGAT.[A]	774.37406
292	[F].PGAQPHAP.[A]	774.38931
293	[R].PGAAGPAPH.[P]	774.38931
294	[P].PAAPGPGAH.[M]	774.38931
295	[A].APGFFVH.[P]	774.39334
296	[G].GKPADSSL.[E]	774.39921
297	[S].GGGPAGSGSE.[V]	775.32169
298	[V].PGQGGADSS.[Q]	775.32169
299	[P].PGPSSPMS.[R]	775.32908
300	[A].AAAVSGSAAA.[E]	775.39446
301	[K].PGSKSTQA.[V]	775.39446
302	[-].MAATVAGSGAA.[E]	775.39446
303	[G].PGPSPGPAP.[P]	776.39373

304	[P].GPSPGPAPP.[N]	776.39373
305	[N].PGTSGPVY.[R]	777.37775
306	[SP].QGPAGPPGP.[P]	777.38898
307	[EV].QGPPGPAGP.[P]	777.38898
308	[P].PGFPHPQ.[S]	779.3835
309	[T].QGRGGPAH.[P]	779.39071
310	[P].GPPEGPVQ.[V]	780.38864
311	[F].PGDAGLPGP.[P]	780.38864
312	[A].GPSSPPAAP.[A]	780.38864
313	[P].GPSPASPAP.[P]	780.38864
314	[P].PSGAPGPPT.[G]	780.38864
315	[C].PAGGADVPP.[Q]	780.38864
316	[W].AAEAAPPGP.[H]	780.38864
317	[-].MGPPDARAP.[L]	780.39988
318	[P].PGSPGAQPA.[L]	781.38389
319	[T].QPGGPAPSA.[V]	781.38389
320	[V].GAPGAPGTPG.[E]	781.38389
321	[A].QLNNGVH.[L]	781.39513
322	[A].GSRGGPPGP.[V]	781.39513
323	[A].GAGPGAQAGP.[S]	782.37914
324	[Q].GAGPGQAAPG.[G]	782.37914
325	[Q].GPGQGPAGAA.[V]	782.37914
326	[G].GAAGGGPAAGP.[A]	782.37914
327	[P].PGSQGSHI.[H]	782.37914
328	[L].AGGAGGPAAPG.[E]	782.37914
329	[P].ASPAAAGHT.[R]	782.37914
330	[R].AAGGAGQGPP.[P]	782.37914
331	[N].GQAGAIEH.[-]	782.37914
332	[P].GPRGNAGGP.[G]	782.39038
333	[A].APGGGPAGAK.[A]	782.41553
334	[A].ESAPPPAD.[A]	783.35192
335	[S].MSTITSGS.[S]	783.3553
336	[G].GAGGWPAAP.[L]	783.37841
337	[P].GAGARGSHA.[A]	783.38562

338	[Q].APALGGSGGP.[G]	783.39954
339	[M].PGSPGPKGS.[P]	783.39954
340	[G].QGPAGAAVL.[G]	783.43593
341	[L].PGSPSLLI.[K]	783.46108
342	[P].AGPGPALSD.[I]	784.38356
343	[K].PGQGGLGAQ.[P]	784.39479
344	[R].AAAQGAAAPG.[G]	784.39479
345	[H].PGGRGGAAAA.[S]	784.40603
346	[M].SSAPAQGPA.[P]	785.37881
347	[-].MSSAPAQGPA.[P]	785.37881
348	[R].AGAGRGAPE.[G]	785.39004
349	[Q].KGDPGRR.[R]	785.43766
350	[G].GPSPSGRE.[S]	786.37406
351	[P].QSPAAAGGAG.[L]	786.37406
352	[V].GPGQSGGLGG.[V]	786.37406
353	[L].PGAHGPPGP.[T]	786.38931
354	[P].APGTSAVSP.[R]	786.39921
355	[A].GNIGPSQD.[P]	787.35807
356	[P].GPGAESRN.[R]	787.36931
357	[G].AGGARGGAGGG.[P]	787.38054
358	[G].GAGGARGGAGG.[G]	787.38054
359	[W].AAEAAAAAAA.[V]	787.39446
360	[A].SAVPGSGAAA.[G]	787.39446
361	[-].MPGAAAKGSE.[L]	787.39446
362	[P].PGAATASVGG.[A]	787.39446
363	[G].GGAGPGHVH.[L]	788.37981
364	[G].TAPPAAFGG.[A]	788.39373
365	[P].GAPAASPFA.[A]	788.39373
366	[P].GPGPGPGPGP.[GA]	789.38898
367	[F].PGNRGAAF.[G]	789.40021
368	[V].GPVSHHR.[V]	789.41145
369	[S].SPGTASTVA.[A]	790.39412
370	[T].GPSASSLAT.[R]	790.39412
371	[H].PGSGVSSLS.[F]	790.39412

372	[S].AAAAAAASTS.[S]	791.38937
373	[A].GPPSSSSSS.[S]	792.337
374	[C].PGSGVTSTS.[W]	792.37339
375	[E].QGPRMGF.[F]	792.38212
376	[P].GPGPAEPPA.[P]	792.38864
377	[V].GPGAAVHSP.[L]	792.39988
378	[G].GPAGGALHL.[A]	792.43626
379	[S].PGAGSPPGPG.[A]	793.38389
380	[P].GPGPSQPGP.[S]	793.38389
381	[K].PGPGPSQPG.[P]	793.38389
382	[F].PGPGSGPQP.[Q]	793.38389
383	[P].PGSGPGPGPA.[T]	793.38389
384	[D].GPGSPEHI.[K]	793.38389
385	[T].GPASPNPPG.[L]	793.38389
386	[R].AAQAHGPAA.[S]	793.39513
387	[L].PPTVWGH.[G]	793.39915
388	[E].GPAPHPFA.[S]	793.39915
389	[T].PPICGHI.[T]	793.40252
390	[V].LLGIGPSH.[Q]	793.45666
391	[F].GPGGAGANPP.[W]	794.37914
392	[K].GPQNPPAN.[A]	794.37914
393	[P].APGGGGGGPAP.[C]	794.37914
394	[E].FFSGISH.[S]	794.38317
395	[G].SPPGGSRH.[G]	794.39038
396	[L].GPLEPGAPG.[T]	794.40429
397	[S].AAIGGMFK.[Q]	794.42292
398	[H].GAGGHRPSG.[P]	795.38562
399	[L].GPDLEPAP.[A]	795.38831
400	[R].APTPSPEP.[P]	795.38831
401	[T].GPSSVTTF.[P]	795.38831
402	[S].AAPDPVEP.[A]	795.38831
403	[Q].AAGAPSPPAG.[S]	795.39954
404	[P].PGVGSPGAPG.[K]	795.39954
405	[S].GPSVGQGPP.[D]	795.39954

406	[S].GPAATPGQP.[G]	795.39954
407	[H].GAPSAAPGPA.[Q]	795.39954
408	[P].GPSPGPSPT.[P]	796.38356
409	[K].AAAAPAGGNP.[E]	796.39479
410	[Y].AGGPGGPGGLG.[L]	796.39479
411	[S].RNAEAVH.[F]	796.40603
412	[N].RGGPAGGAGP.[D]	796.40603
413	[A].VGQGGVSGH.[R]	797.39004
414	[D].AAAAASAQH.[S]	797.39004
415	[A].ARDGPAGPG.[P]	797.39004
416	[L].APGRDQPG.[S]	797.39004
417	[P].APGGGDPRA.[G]	797.39004
418	[P].AAEDVHR.[L]	797.39004
419	[G].AGGPWGPVG.[G]	797.39406
420	[S].GSPGVGLGPG.[E]	797.41519
421	[P].GSPVGLGGGP.[S]	797.41519
422	[D].KGDPGVPGA.[P]	797.41519
423	[D].ADAGSGSPH.[S]	798.33767
424	[K].NRGPGPGGS.[C]	798.38529
425	[C].AAASPDLGP.[G]	798.39921
426	[V].PGSPTQVL.[F]	798.43559
427	[T].QSRGHSGA.[R]	799.38054
428	[S].QGPGRGGGGG.[A]	799.38054
429	[A].TGVGGPSPGA.[A]	799.39446
430	[A].AGAASASPAP.[R]	799.39446
431	[K].NVPSSAGPA.[A]	799.39446
432	[A].PGSPGKGEA.[E]	799.39446
433	[L].AAGPTGSAAP.[A]	799.39446
434	[D].APGTASAQP.[P]	799.39446
435	[T].PGSTAAAPAG.[L]	799.39446
436	[C].PGSPGAEDA.[A]	800.34209
437	[G].PGEPAGDAS.[G]	800.34209
438	[G].GPGGTLGNAG.[C]	800.38971
439	[K].GPSGPGGKGS.[P]	800.38971

440	[-].MAAAAAGAGAGA.[A]	800.38971
441	[L].AAGSVPNGQ.[E]	800.38971
442	[G].PGSLMQAP.[E]	800.3971
443	[C].LPPAMQK.[V]	800.43349
444	[G].AAGPGGRES.[Q]	801.38496
445	[A].GAPGGGRTE.[W]	801.38496
446	[P].GPAGPAFNA.[T]	801.38898
447	[Q].SVGPWAQG.[Q]	801.38898
448	[D].GPSGDRRG.[D]	801.39619
449	[-].MGKAGDPGSL.[R]	801.41011
450	[A].AASAASAVGP.[V]	801.41011
451	[S].APATSAGGGI.[F]	801.41011
452	[G].ARGGEAAGL.[K]	801.42134
453	[G].QRDTGPK.[A]	801.42134
454	[A].KEQGALR.[E]	801.45773
455	[G].GPAGAEGPM.[A]	802.33998
456	[G].APAASPFAA.[A]	802.40938
457	[T].AAAAAETAAG.[A]	803.38937
458	[A].AAAAETAAGA.[E]	803.38937
459	[A].SPAASSGGLG.[C]	803.38937
460	[K].GPEPSGFL.[S]	803.3934
461	[A].TSPLSPMA.[N]	803.39677
462	[L].GPASSRSAA.[S]	803.40061
463	[K].GPGAATSSR.[P]	803.40061
464	[Q].GPGPGPGPAP.[G]	803.40463
465	[G].PGPGPGPGPA.[S]	803.40463
466	[Q].GPAGPPGPPG.[P]	803.40463
467	[S].LPGMSLVA.[G]	803.43315
468	[D].FGFDGDF.[Y]	804.3199
469	[T].PGSTGRTE.[P]	804.38462
470	[P].PGEPGPPGP.[RP]	804.38864
471	[P].PGPEGPPGP.[P]	804.38864
472	[F].PATPHGQP.[V]	804.39988
473	[N].HTPAGAPGP.[S]	804.39988

474	[A].GAAHPPGTP.[F]	804.39988
475	[S].PGNSRGAF.[L]	805.39513
476	[Q].GPGAAAHPQ.[T]	805.39513
477	[L].PGPGEPVPG.[P]	806.40429
478	[E].GQIAPPSH.[L]	806.41553
479	[-].MPLGTGPPAP.[A]	806.44068
480	[S].GPGPSAGPAP.[R]	807.39954
481	[P].PPGSAGGPAP.[F]	807.39954
482	[P].GPPGSPAPQ.[N]	807.39954
483	[G].PQGPTGPPG.[P]	807.39954
484	[A].GPAGAGLQH.[S]	807.41078
485	[L].RGAGVPGPP.[C]	807.44716
486	[G].APGQGGAPGP.[P]	808.39479
487	[G].GAGFFRPG.[Q]	808.41005
488	[M].KMAHAPPG.[H]	808.41342
489	[A].AGAGSVPGGH.[Y]	809.39004
490	[T].QPNGGLSH.[T]	809.39004
491	[C].PGAWPQPG.[A]	809.39406
492	[E].PGAGARGSH.[A]	809.40127
493	[Q].PGAAAASAPP.[R]	809.41519
494	[L].GPAATPAGPA.[R]	809.41519
495	[P].GPGVSAAPGP.[A]	809.41519
496	[R].GPSGLPGPAG.[P]	809.41519
497	[C].GPSPGAPPM.[P]	810.38145
498	[P].AAGGPFPGH.[H]	810.38931
499	[G].PGAATHAW.[R]	810.38931
500	[E].PGAGSEVPP.[V]	810.39921
501	[K].GPGAGQGAVP.[G]	810.41044
502	[Q].PGAAGAGAAPA.[L]	810.41044
503	[A].AASAVPSAH.[R]	810.41044
504	[L].GAVGPAAGGGP.[N]	810.41044
505	[P].AAGAAPAGGPA.[P]	810.41044
506	[D].PAATPAGLL.[L]	810.47198
507	[G].GPGAGGLEGP.[P]	811.39446

508	[V].APGPGPASAS.[G]	811.39446
509	[S].SAPGLGFY.[S]	811.39848
510	[S].SPGPGRGSP.[D]	811.40569
511	[Q].AARDGPAGP.[G]	811.40569
512	[P].GPRGEPGAA.[G]	811.40569
513	[L].PGAQPREG.[L]	811.40569
514	[L].PAGGADARP.[P]	811.40569
515	[P].AAPAGWAAP.[Q]	811.40971
516	[M].GPQGQVLL.[Q]	811.46723
517	[P].PGAQGGGPGD.[P]	812.35332
518	[P].GAPGMVPAP.[L]	812.3971
519	[I].GPASVGPPM.[E]	812.3971
520	[G].LMGPPGPQ.[G]	812.3971
521	[L].GAGGRGPSPG.[P]	812.40094
522	[Q].GPPGHPGPP.[G]	812.40496
523	[A].PGSPGTLAL.[R]	812.45124
524	[C].AASAHRSGG.[G]	813.39619
525	[A].AAAAGATPPS.[L]	813.41011
526	[A].PGAAASDPK.[Q]	813.41011
527	[L].APGAATASAP.[V]	813.41011
528	[E].PGAGAEKPS.[F]	813.41011
529	[Q].GPAGTSVAGP.[Q]	813.41011
530	[A].AASAVPGSPG.[Y]	813.41011
531	[E].KEGLETH.[H]	813.41011
532	[G].LGPSGASGPA.[L]	813.41011
533	[L].QPGNISPT.[S]	813.41011
534	[E].GTVTGELH.[N]	813.41011
535	[V].AGGGPGGVLE.[P]	813.41011
536	[D].RSGPQIR.[E]	813.46896
537	[Q].GVAGSAGAPGA.[T]	814.40536
538	[L].PGAAAAQAAS.[W]	814.40536
539	[S].VSPGAGGGVN.[E]	814.40536
540	[S].SSPASPAAAG.[H]	815.38937
541	[D].PSGPGIGSSG.[T]	815.38937

542	[A].AAAGAGAGAAQ.[E]	815.40061
543	[G].GSPGRSSPA.[G]	815.40061
544	[A].PGSPSRGGT.[G]	815.40061
545	[G].GPPGRSGST.[F]	815.40061
546	[A].QGPGGGGTGK.[L]	815.40061
547	[P].QRSSPGSP.[W]	815.40061
548	[D].AGPAGAFPGA.[L]	815.40463
549	[G].LPSTMAVP.[E]	815.43315
550	[N].KPPMETI.[A]	815.43315
551	[E].AGPGGGGGSEA.[G]	816.34824
552	[P].SPPGPGGTF.[P]	816.38864
553	[D].PGPSQPHP.[Y]	816.39988
554	[P].PGEPGFRG.[A]	816.39988
555	[A].QPGPSHPP.[P]	816.39988
556	[G].GPGHPTPPG.[H]	816.39988
557	[P].GPAGKDASD.[I]	817.36864
558	[P].GPGGPQHAP.[F]	817.39513
559	[G].GAPGPQPHG.[E]	817.39513
560	[P].PGPGGPQHA.[P]	817.39513
561	[P].ATAGSPATAA.[G]	817.40502
562	[G].GPAASAVSAS.[V]	817.40502
563	[T].PAGAGSALSS.[H]	817.40502
564	[Q].PGASAAGLSS.[L]	817.40502
565	[A].AGAPTGSLGS.[R]	817.40502
566	[G].PGATSSQVA.[R]	817.40502
567	[A].GPAGASTAVS.[K]	817.40502
568	[R].GPSAVSTQA.[P]	817.40502
569	[S].PGAGSDSDL.[S]	818.35265
570	[G].AGPGPPPEP.[P]	818.40429
571	[P].PGPSGPLGH.[P]	818.41553
572	[VE].GPSGPPGPPG.[FK]	819.39954
573	[R].PPGSGPGPGP.[A]	819.39954
574	[E].AAAPGGAAYA.[K]	819.39954
575	[N].GPSPFFPA.[D]	819.40357

576	[R].GPGGHLEPG.[M]	820.39479
577	[V].GPGPGGPGPAG.[P]	820.39479
578	[P].GPSHQPTP.[H]	820.39479
579	[A].APGPDRAH.[R]	820.40603
580	[P].EPGPAGRH.[R]	820.40603
581	[G].GPPGEPGLP.[G]	820.41994
582	[V].PGTASPPPP.[P]	820.41994
583	[E].PSPSPAPPA.[Q]	820.41994
584	[D].ASAAPATSF.[T]	822.39921
585	[S].PGAGSPHLS.[Q]	822.41044
586	[A].PGAAAPGAGPG.[P]	822.41044
587	[V].GPQQQPPA.[G]	822.41044
588	[T].LPPSSGPPA.[L]	822.43559
589	[S].GAGGISPQH.[I]	823.40569
590	[K].SVSHSVAH.[T]	823.40569
591	[S].PGGDRPAPG.[T]	823.40569
592	[E].GGPREGPGP.[P]	823.40569
593	[A].GAGGPGTGHL.[P]	823.40569
594	[A].PGPGADRGP.[W]	823.40569
595	[G].PSGRAQAH.[A]	823.41692
596	[T].KPQASRH.[E]	823.45331
597	[P].PGGSLEPAP.[S]	824.41486
598	[D].GPAAQNPGN.[L]	825.38496
599	[P].AGPQMAPGP.[-]	825.39235
600	[R].GPGGRASSH.[Q]	825.39619
601	[A].PGGVSGPSPA.[Q]	825.41011
602	[G].PSGGPTVGGP.[P]	825.41011
603	[P].QPTSPQPA.[A]	825.41011
604	[A].AASPSAPAPG.[L]	825.41011
605	[S].PGKEGSGPP.[G]	825.41011
606	[L].RAAGGEPAP.[Q]	825.42134
607	[V].QVGTPAPR.[T]	825.45773
608	[G].GPSPGRRV.[L]	825.46896
609	[Y].TAAPGGGPAGA.[K]	826.40536

610	[G].GGPTAAGGAPA.[A]	826.40536
611	[S].PGSPGAGGVQ.[S]	826.40536
612	[P].GGPGAGGGPVT.[P]	826.40536
613	[P].QAASQPAPG.[A]	826.40536
614	[P].QPGGKDGAP.[G]	826.40536
615	[-].MQVGQGGSPP.[M]	826.40536
616	[A].QSPTAVHS.[P]	826.40536
617	[Q].APGGPGGVTGG.[K]	826.40536
618	[R].WLGEGPAP.[H]	826.40938
619	[L].PAGGGSARPG.[S]	826.41659
620	[P].PSGPGAGRAG.[V]	826.41659
621	[I].AGRAESVH.[P]	826.41659
622	[Y].PGPHPAGPP.[V]	826.42061
623	[I].PGPPHAPGP.[G]	826.42061
624	[G].KAAASELH.[P]	826.44174
625	[Q].KSSSHLPA.[N]	826.44174
626	[G].RPDSAPAL.[H]	826.44174
627	[G].PAGDRSAGP.[G]	827.40061
628	[A].AAAAGGAGPGAG.[I]	827.40061
629	[Q].GPQGMLGPA.[Q]	827.408
630	[T].GLDAGGLGPA.[G]	827.42576
631	[T].HATFAPGQ.[S]	828.39988
632	[A].SGPVSDGLP.[S]	828.40977
633	[A].PGAAGASVGAA.[A]	828.42101
634	[G].GPAGGSRAR.[A]	828.44347
635	[V].QSTPLPSV.[E]	828.44616
636	[I].TQSPPYH.[R]	829.38389
637	[K].QPEPGKSS.[C]	829.40502
638	[T].PGAAGGATAAS.[A]	830.40027
639	[G].PGAGIGSGSGA.[G]	830.40027
640	[Q].APGFPPTGS.[S]	830.40429
641	[S].PGAQSASRG.[T]	830.41151
642	[D].GPSTRAGGQ.[A]	830.41151
643	[P].GVGGSGRSPG.[S]	830.41151

644	[L].GARGGEGVQ.[V]	830.41151
645	[V].PPSSSEKV.[C]	830.42542
646	[K].QGVAEAAGK.[T]	830.43666
647	[V].AAAAAEAKK.[K]	830.47304
648	[G].PGAAAGGGNC.[R]	831.34138
649	[R].RGAGSGAGAAG.[R]	831.40675
650	[V].GRASAGAGGAG.[A]	831.40675
651	[P].EGVGAGRW.[A]	831.41078
652	[F].GQVATSTAP.[S]	831.42067
653	[T].AAAADATLAG.[E]	831.42067
654	[M].GPASANTTL.[A]	831.42067
655	[L].GPTGQAAAC.[F]	832.36178
656	[F].NAPGAQQF.[C]	832.39479
657	[G].GPSPPGHSP.[G]	832.39479
658	[A].GPAGGYPTL.[G]	832.41994
659	[G].PGPAEPPAP.[G]	832.41994
660	[P].GDGLFPLN.[N]	832.41994
661	[Q].VGADAAMVV.[T]	832.42332
662	[H].QPQFTVI.[S]	832.45633
663	[G].MVAQPGASG.[M]	833.38218
664	[G].YALMAGAH.[G]	833.39744
665	[A].GDAVAAASAT.[A]	833.39994
666	[T].GPGPSPGPAP.[P]	833.41519
667	[G].PGPSPNPPA.[W]	833.41519
668	[R].GPAGAGPGPGP.[G]	834.41044
669	[A].PGQGGAPGPP.[G]	834.41044
670	[A].GAGPGPGPGAP.[P]	834.41044
671	[S].AAPSSHPPA.[S]	834.41044
672	[P].GRAGGQAAF.[L]	834.42168
673	[N].QPSTGSGDS.[A]	835.34282
674	[A].GAPAQASPH.[C]	835.40569
675	[A].GPAGAHQPT.[A]	835.40569
676	[C].GPGPGAATHA.[W]	835.40569
677	[G].PGARGSPGH.[R]	835.41692

678	[G].GPSRHQGP.[L]	835.41692
679	[L].EAPAAGSPH.[A]	836.38971
680	[G].PSGAAMRF.[D]	836.40833
681	[A].AAAASSPYV.[Y]	836.41486
682	[Q].PGSEGVPPP.[L]	836.41486
683	[A].PTAGALYSG.[S]	836.41486
684	[Q].QPQAPPQA.[P]	836.42609
685	[L].PQQAPPAQ.[A]	836.42609
686	[I].QPQAAPGPA.[T]	836.42609
687	[P].KGNPGLPGP.[K]	836.46248
688	[A].PGAASALHL.[S]	836.46248
689	[E].SGPGTPHGAG.[P]	837.38496
690	[P].GPAGPMGPPG.[L]	837.39235
691	[PI].SPGEISFT.[KN]	837.39887
692	[P].GPSGPPAAPS.[P]	837.41011
693	[S].SPTGQPPPG.[A]	837.41011
694	[A].GPAGPSSPPA.[A]	837.41011
695	[P].GPGPGPTTGP.[G]	837.41011
696	[Q].APAAGEGPAP.[D]	837.41011
697	[T].GQPGPPGLD.[G]	837.41011
698	[P].GDAGLPGPPG.[F]	837.41011
699	[D].PATPMALH.[Q]	837.42874
700	[K].KGDHRAPG.[T]	837.43257
701	[N].PGAPGRWP.[R]	837.4366
702	[L].GAPGGSPPAQ.[P]	838.40536
703	[G].GPTGAPPGGGA.[L]	838.40536
704	[P].AAAPEPPW.[P]	838.40938
705	[P].PGSPGPRGN.[A]	838.41659
706	[Q].GPVFHGPQ.[V]	838.42061
707	[R].LKAQLEH.[L]	838.47813
708	[H].ANPPTLLL.[P]	838.50328
709	[S].APGGGGGTEH.[R]	839.36422
710	[P].GAPGGGAQAGP.[G]	839.40061
711	[I].AAASPSAPAP.[G]	839.42576

712	[Q].PGTAPSGVPG.[A]	839.42576
713	[I].GAGAFRDF.[R]	840.39988
714	[Q].PGAGGGQSLP.[M]	840.42101
715	[A].QPGSVAGAGP.[G]	840.42101
716	[G].PSGPGGKGSP.[S]	840.42101
717	[P].GPAAAANATP.[A]	840.42101
718	[P].PRGLPTEA.[V]	840.45739
719	[F].NPGAGLPTD.[K]	841.40502
720	[T].KGNQGPSGP.[Q]	841.41626
721	[A].AAAAGGQQAP.[E]	841.41626
722	[G].PAGAAGAQAGA.[R]	841.41626
723	[A].GPAQSGILV.[D]	841.47779
724	[I].GAPTSGAGPAG.[P]	842.40027
725	[Y].QPLGDQGQ.[A]	842.40027
726	[P].GPSGLGGAGGL.[A]	842.43666
727	[H].GPQSYHR.[S]	844.40603
728	[K].PGGVGAGSGVS.[S]	844.41592
729	[P].AAGGQPLSGS.[R]	844.41592
730	[A].APGGGSVAAAS.[A]	844.41592
731	[P].GPPGTPFAT.[A]	844.41994
732	[N].QRGSGVAGL.[K]	844.46354
733	[V].PGPSGRDC.[Q]	845.35703
734	[V].GTVGGGAGGVGG.[G]	845.41117
735	[N].GPSREVGSG.[L]	845.41117
736	[-].MQTGGQVAGQ.[G]	845.41117
737	[E].AAAAAGGGGATA.[A]	845.41117
738	[G].AGTGPSRAE.[A]	845.41117
739	[V].ATAGPGRES.[T]	845.41117
740	[V].PGSPGFLNG.[S]	845.41519
741	[A].PGAAAPFGSA.[Y]	845.41519
742	[M].PGSVTHFT.[L]	845.41519
743	[E].PGAAGNVFL.[S]	845.45158
744	[E].SPGPGPPHT.[L]	846.41044
745	[L].PGAQNNVF.[T]	846.41044

746	[P].PGAGMLGFP.[P]	846.41784
747	[G].PNQGRQF.[YH]	846.42168
748	[S].PGAAAYPSL.[T]	846.43559
749	[Q].PGALGSTPF.[L]	846.43559
750	[Q].KDDITAAL.[V]	846.45672
751	[S].GPASAPSTC.[S]	847.36144
752	[E].PSAASPTGC.[P]	847.36144
753	[K].GEGGLPTCG.[P]	847.36144
754	[-].MAAGGLSRSE.[R]	847.42682
755	[Q].SPGGISSDE.[E]	848.36322
756	[S].GPAGGRGGGY.[G]	848.40094
757	[D].GPEPPSPAP.[E]	848.41486
758	[Y].GPEPTPPGP.[A]	848.41486
759	[E].QPNITEF.[A]	848.41486
760	[H].GPGPSPGPSP.[T]	849.41011
761	[P].GPSPGPGPSP.[G]	849.41011
762	[G].AGTPAHAAGP.[K]	849.42134
763	[A].GPPGSGGQPP.[P]	850.40536
764	[F].PGSPGADLH.[G]	850.40536
765	[L].SPGPGHLW.[S]	850.42061
766	[F].GGAPAAGRGH.[P]	850.42782
767	[A].APGQHRGQ.[A]	850.42782
768	[S].PGALGYSSV.[G]	850.43051
769	[C].SVGGPSVTF.[L]	850.43051
770	[T].PSPSTPAPP.[T]	850.43051
771	[Q].GPATTVAFS.[R]	850.43051
772	[P].GPPSGPILL.[Q]	850.50328
773	[P].GPEGPSGPPG.[P]	851.38937
774	[G].GPAGPGGPGGGA.[G]	851.40061
775	[A].GPPAGGMPPA.[P]	851.408
776	[-].MQSPRQGH.[G]	851.41184
777	[P].GSPPGGSRH.[G]	851.41184
778	[G].SPPGGSRHG.[L]	851.41184
779	[A].SPGSGPGRH.[P]	851.41184

780	[D].GSPGPAPAPT.[P]	851.42576
781	[T].GSPAPASPPA.[A]	851.42576
782	[G].PGAGGLEGPP.[Q]	851.42576
783	[V].PSSAGPAAPP.[A]	851.42576
784	[T].PGTPAPSAGP.[P]	851.42576
785	[A].AAAAPGPGSGP.[G]	852.42101
786	[L].PGSVPASTH.[S]	852.42101
787	[P].AAGGYFVPA.[V]	852.42503
788	[G].AGGRGPSPGP.[A]	852.43224
789	[Y].AGSRGGPPGP.[V]	852.43224
790	[G].SPGRQGPPG.[R]	852.43224
791	[-].MAGGGRQPAP.[G]	852.43224
792	[L].KNPFYLA.[L]	852.46142
793	[A].SVPGPEGGGP.[L]	853.40502
794	[K].PGAQGAAAGPG.[D]	853.41626
795	[G].SRGEPGPPG.[L]	853.41626
796	[E].GPAGFPGPPG.[I]	853.42028
797	[E].GPAMVAGPGP.[G]	853.42365
798	[G].GRGPSSRH.[A]	853.43872
799	[T].GPSPGAPGLT.[N]	853.44141
800	[L].ATPAGATEH.[V]	854.40027
801	[G].QPGEPGRN.[D]	854.41151
802	[G].AGGPWGPVGG.[G]	854.41553
803	[K].NGVYPPAH.[R]	854.41553
804	[P].GAGARGSHAA.[D]	854.42274
805	[V].SQGLPPPC.[P]	855.40291
806	[F].GSVGPSPASP.[S]	855.42067
807	[P].AASTPAGPPS.[G]	855.42067
808	[S].PGSPGSPGSI.[P]	855.42067
809	[E].GPPGSEPSE.[P]	856.3683
810	[P].QPGNGSLSP.[A]	856.41592
811	[E].AAGAGPAASSP.[Q]	856.41592
812	[R].GAPGRGSSPA.[S]	856.42716
813	[G].GPEGRGAAAA.[A]	856.42716

814	[I].KGANNQGAP.[G]	856.42716
815	[R].RGGAGTPAAV.[P]	856.46354
816	[V].PGAGGVQAGGS.[Q]	857.41117
817	[H].APGGPGPHPA.[S]	857.42643
818	[Y].PGAPHPGGAP.[S]	857.42643
819	[F].PSSASGPVGV.[T]	857.43632
820	[N].KPGESRAL.[G]	857.48394
821	[A].VSPGGATRL.[T]	857.48394
822	[G].VGSPDPISS.[P]	858.42034
823	[T].GLGGEDLPT.[I]	858.42034
824	[P].PGAATASVGGA.[E]	858.43157
825	[A].QGDVITLL.[I]	858.49311
826	[A].GPSGVSAVDA.[A]	859.41559
827	[P].GPSTGALQE.[R]	859.41559
828	[A].AAAAAAAASGGA.[G]	859.42682
829	[P].KDNGGAAIN.[K]	859.42682
830	[V].AAAAAERAE.[A]	859.42682
831	[P].GPASVGFPAG.[D]	859.43084
832	[A].SPGGFPLEG.[P]	860.41486
833	[D].GPGSPFGLE.[P]	860.41486
834	[F].GARATSSGGP.[P]	860.42207
835	[PYAMV].GPPGPQGPPG.[LYEDK]	860.42609
836	[A].GPGPGPGAPPG.[L]	860.42609
837	[Q].GPGPGPGPAPG.[E]	860.42609
838	[P].GPGPGPGPGPA.[S]	860.42609
839	[G].PGPGGPGPAGP.[M]	860.42609
840	[C].AASPSQVAM.[A]	861.41348
841	[A].PQGPPQTH.[G]	861.42134
842	[R].PGPAAPSGHA.[G]	861.42134
843	[P].PGASGAPPHA.[G]	861.42134
844	[K].GPASTTTLN.[L]	861.43124
845	[V].AAASATAAVE.[P]	861.43124
846	[A].SALSTTGPGA.[S]	861.43124
847	[L].GPPGSVYEG.[G]	862.39412

848	[V].PGSPGIMGF.[Q]	862.41275
849	[A].TGATGTVGAGA.[D]	862.42649
850	[G].APGALSPSY.[D]	862.43051
851	[L].PGGSLTSASS.[V]	863.4105
852	[V].PGKASDTST.[T]	863.4105
853	[G].RGPGGGEHP.[D]	863.41184
854	[E].PAGPGEPAAP.[F]	863.42576
855	[G].PGAGAAPEPP.[A]	863.42576
856	[A].QPATAAAYA.[S]	863.42576
857	[W].AGGPEPAAPP.[A]	863.42576
858	[K].GEVGPPGPPG.[P]	863.42576
859	[G].PGSGPQPQP.[L]	864.42101
860	[L].GPGGPPSAGPA.[P]	864.42101
861	[E].QPGGPGSPPA.[Q]	864.42101
862	[A].AASAHVHAT.[L]	864.43224
863	[A].AAGIGHPGAGG.[H]	864.43224
864	[E].AAHGGTTIH.[I]	864.43224
865	[P].PAAGGLQNH.[T]	864.43224
866	[P].PAAGPHPFA.[S]	864.43626
867	[M].PGAGPMVVH.[C]	864.43963
868	[P].SPGPTPPVL.[P]	864.48254
869	[L].GPLSYSKI.[K]	864.48254
870	[L].PGAPGQGGAPG.[P]	865.41626
871	[K].GPQNPPANA.[M]	865.41626
872	[P].GAPGQGGAPGP.[P]	865.41626
873	[P].PSGARSHPG.[S]	865.42749
874	[Q].QREAPHAG.[G]	865.42749
875	[E].QPGRPSSH.[G]	865.42749
876	[G].NWIAGPAH.[T]	865.43151
877	[S].GPGPWGAAGP.[D]	866.41553
878	[P].PQDPAEIP.[G]	866.42542
879	[Q].PGSVAGAGPGP.[T]	866.43666
880	[C].GPQNGSPGGP.[G]	867.39552
881	[P].GPGAATHAW.[R]	867.41078

882	[V].GAPFGPQGH.[R]	867.41078
883	[P].AAAQQAGPGP.[V]	867.43191
884	[G].AGAAGAPAGGAP.[E]	867.43191
885	[N].QPGNPQVQ.[S]	867.43191
886	[G].AAAGPAPQGGA.[F]	867.43191
887	[A].VNGGGLHASG.[A]	868.42716
888	[S].GPPGGSPGRS.[S]	868.42716
889	[Q].AARDGPAGPG.[P]	868.42716
890	[V].RDAAGGPGAP.[A]	868.42716
891	[Q].GPPGKMGPQ.[G]	868.43455
892	[C].RPGFAPSH.[E]	868.44241
893	[A].ANVTGPGGVP.[V]	868.45231
894	[V].PGAGGVAAVLG.[P]	868.48869
895	[Q].PGAGRGAGLL.[L]	868.49993
896	[S].AASQGAGGGPP.[P]	869.41117
897	[V].GSPGNRQGP.[A]	869.4224
898	[A].AAAAAPETAP.[S]	869.43632
899	[F].LGGEGPVSGP.[Q]	869.43632
900	[P].PVGSQADPV.[S]	869.43632
901	[T].QRQMAHV.[Q]	869.44103
902	[L].QDGGSGGRH.[S]	870.38127
903	[S].PNTPAHFS.[F]	870.41044
904	[S].QGPGRGGGGGA.[G]	870.41765
905	[F].PGAKGDAGTP.[G]	870.43157
906	[C].PGAPGSTVGQ.[Q]	870.43157
907	[Y].AEAAAAQAPA.[A]	870.43157
908	[N].GAPPDPGFL.[R]	870.43559
909	[L].GPASASGRAP.[R]	870.44281
910	[P].AAGAAGGGDGAP.[G]	871.39044
911	[-].MAAAAAGAGAGAA.[Q]	871.42682
912	[Y].APGGAAATANA.[V]	871.42682
913	[G].PGAPADTSR.[P]	871.42682
914	[W].SGRGGTSHL.[S]	871.43805
915	[G].PGAGGPYPR.[G]	871.44208

916	[R].PGSPGAARC.[P]	872.40431
917	[S].GPAGQTDLN.[K]	872.41084
918	[S].PGGSTAAPSGA.[L]	872.41084
919	[A].ATAAAHSFP.[L]	872.42609
920	[D].SVGGFRYS.[E]	872.42609
921	[P].GPAPGFAAGQ.[Q]	872.42609
922	[A].GAGAAVCIPG.[E]	872.42946
923	[E].AGPGGGGGSEAG.[P]	873.3697
924	[E].SPPNPGAPH.[A]	873.42134
925	[G].PGSPLSSGTA.[W]	873.43124
926	[A].GPPSITSSGA.[D]	873.43124
927	[T].SLGQAQAQA.[A]	873.44247
928	[R].GAGAGGRSAGGG.[P]	874.41257
929	[D].GGAPGPQPHG.[E]	874.41659
930	[T].AAAAAETAAGA.[E]	874.42649
931	[P].ATAGSPATAAG.[P]	874.42649
932	[P].PGPAGPPGAPG.[K]	874.44174
933	[A].QPPPGPGPAG.[V]	874.44174
934	[P].AGPPGPQGPP.[G]	874.44174
935	[L].QPNGAIFE.[P]	875.42576
936	[V].PGPEPGPQP.[A]	875.42576
937	[D].GPPGHPGKE.[G]	875.43699
938	[G].PGQPTAPAH.[Q]	875.43699
939	[G].GPSVRVTC.[K]	875.44036
940	[L].GPGAFPSED.[R]	876.37339
941	[R].PAEEVTAC.[Q]	876.37676
942	[R].PGGPPNPSGP.[S]	876.42101
943	[G].PGGAAAAAPGH.[P]	876.43224
944	[K].PGGPGLPGQP.[G]	876.45739
945	[Y].PGPSAGGPHT.[S]	877.41626
946	[G].FGAPPGGAGF.[P]	877.42028
947	[A].PAGSVTATSS.[P]	877.42615
948	[G].PAGERGHPG.[S]	877.42749
949	[A].ASAPRTSTS.[S]	877.43739

950	[G].PGEPVPGPAG.[P]	877.44141
951	[G].VQGAEVGAF.[G]	877.44141
952	[D].PPSGTPPQP.[C]	877.44141
953	[W].VPERETF.[S]	877.44141
954	[P].GPSPASPAPP.[E]	877.44141
955	[A].GAGVEGGAFL.[G]	877.44141
956	[V].SGPGPPPGLV.[R]	877.47779
957	[P].QPGAAPGPPS.[G]	878.43666
958	[-].MAAAGAGPGPGPG.[A]	879.43191
959	[L].PAGSVQPSH.[R]	879.43191
960	[C].PGAASVSGPH.[T]	879.43191
961	[Q].RSTQWTT.[D]	879.43191
962	[R].QGPGGPAQAP.[P]	879.43191
963	[-].MPGPQGAGGAPA.[M]	879.43191
964	[L].PGAPWGPPT.[P]	879.43593
965	[S].PGPRGNAGGP.[G]	879.44314
966	[V].PGSPGLTGPP.[G]	879.45706
967	[E].PGADGSETF.[A]	880.3683
968	[H].GAPAAGQATH.[T]	880.42716
969	[M].PGAGPMVVH.[C]	880.43455
970	[E].PGAGARGSHA.[A]	880.43839
971	[A].GPVGTAGAPGP.[Q]	880.45231
972	[P].GPGVSAAPGPA.[A]	880.45231
973	[G].PGAGGGPVTPA.[E]	880.45231
974	[L].GPEGSGSGSF.[S]	881.36355
975	[Y].GSPHLHFS.[P]	881.42643
976	[N].AAGVGEPSPP.[T]	881.43632
977	[E].PGPGTTPSAP.[L]	881.43632
978	[K].LDPHTGEL.[T]	881.43632
979	[K].PGQGGLGAQP.[A]	881.44756
980	[E].PGRSDPGVP.[G]	881.44756
981	[Y].PAGAAGAQAAP.[Q]	881.44756
982	[E].QVANGPAQP.[P]	881.44756
983	[G].PAVAQGNGAP.[A]	881.44756

984	[F].CPGGGPSPGP.[P]	882.37743
985	[P].VQGGAHPGC.[V]	882.38866
986	[L].APTSGAPGGAP.[Q]	882.43157
987	[G].APTSGAGPAGP.[Y]	882.43157
988	[F].ASAGAPASGPP.[P]	882.43157
989	[S].GPAGQELGPG.[E]	882.43157
990	[A].PGAAGTPNTP.[R]	882.43157
991	[A].AEGVWPGPA.[P]	883.43084
992	[P].GPSPGAMLGP.[S]	883.43421
993	[P].GPGPSPGAML.[G]	883.43421
994	[G].PGPSPGAMLG.[P]	883.43421
995	[S].PGAMLGPSPG.[P]	883.43421
996	[-].MSGGGRPQPGA.[A]	883.43805
997	[T].PSGGGRAGAGP.[H]	883.43805
998	[I].PGPPHAPGPG.[G]	883.44208
999	[A].GPGSPATLSP.[S]	883.45197
1000	[G].ESVVGAPGAP.[G]	883.45197
1001	[S].PGAGGGVRGGV.[G]	883.47444
1002	[G].PGAGRGAPDS.[A]	884.42207
1003	[A].LCGVGGAGPP.[G]	884.42946
1004	[G].PGGAGGARGGAG.[G]	884.4333
1005	[C].KSDPAPGAAA.[P]	884.44722
1006	[L].PGAGADLAVD.[P]	885.43124
1007	[L].PGAGNEVLE.[L]	885.43124
1008	[D].PAGGGGLLED.[L]	885.43124
1009	[S].GPSPGDRTV.[G]	885.44247
1010	[Y].PQGAGTRAK.[Y]	885.49009
1011	[M].AAAEAAAVVL.[S]	885.50401
1012	[-].MAAAEAAAVVL.[S]	885.50401
1013	[E].FFGHGGPPA.[A]	886.42061
1014	[Q].GTQAGQLDP.[S]	886.42649
1015	[R].AGAAAAEPGTA.[S]	886.42649
1016	[F].AKDSVNPGV.[V]	886.46287
1017	[L].RGAAGMAPR.[R]	886.46758

1018	[S].GPASTGGAQAA.[S]	887.42174
1019	[K].GPALWEAGS.[P]	887.42576
1020	[A].GAPTQYPPG.[R]	887.42576
1021	[G].SAGAAGGGAAGAA.[G]	888.41698
1022	[G].PGSLGLEES.[G]	888.4309
1023	[Q].PGGAAPPGHQ.[M]	888.43224
1024	[S].QPGGAAPPGH.[Q]	888.43224
1025	[P].GAPHATPHT.[G]	888.43224
1026	[Q].AQASGALSSP.[P]	888.44214
1027	[-].MAASGSAGVPAT.[V]	888.44214
1028	[T].IGPTETSIA.[P]	888.46729
1029	[A].TAAGPATATE.[E]	889.42615
1030	[P].GPGPAEPPAP.[G]	889.44141
1031	[S].GPAPEPGPAP.[P]	889.44141
1032	[E].GPGPSPNPPA.[W]	890.43666
1033	[P].GPSPGPAPPN.[Y]	890.43666
1034	[V].PAGGGGTFLGG.[F]	890.43666
1035	[C].QPGGGPPSPP.[P]	890.43666
1036	[P].PAGGAPAVPGP.[S]	890.47304
1037	[V].GAPGLPGPAGP.[K]	890.47304
1038	[A].PGQGGAPGPPG.[L]	891.43191
1039	[R].GPAGAGPGPGPG.[S]	891.43191
1040	[S].NPQLMAAF.[I]	891.4393
1041	[S].VAGGGGRGGFG.[G]	891.44314
1042	[I].GPRSPGPSH.[P]	891.44314
1043	[R].GPAPREAPP.[G]	891.46829
1044	[G].PGATSPPEH.[C]	892.41592
1045	[-].MQSKMQGR.[V]	892.43053
1046	[Q].PGSPGPAGVPG.[L]	892.45231
1047	[P].PGQGLGGPLP.[R]	892.48869
1048	[V].PGAAAGVPRP.[Q]	892.49993
1049	[P].PQQGMVPH.[G]	893.4298
1050	[A].GAAGSSAKASS.[N]	893.4323
1051	[P].GSPGPPGPLD.[A]	893.43632

1052	[S].GPSPSPPASP.[S]	893.43632
1053	[P].GPSPGPSPTP.[R]	893.43632
1054	[T].PGAPGMVPAP.[L]	893.45495
1055	[S].QRAMPPPP.[P]	893.46618
1056	[G].PAGSSPPALP.[T]	893.47271
1057	[R].APGPGADRGP.[W]	894.44281
1058	[V].APDGGRPAGP.[P]	894.44281
1059	[N].PGSGGSGVYD.[S]	895.3792
1060	[R].GARAAGAPGPA.[R]	895.47444
1061	[Y].PSSAAPVPAV.[A]	895.48836
1062	[P].GPSPGPALSL.[D]	895.48836
1063	[K].LPPSNSALP.[N]	895.48836
1064	[A].EAAAAFLGM.[G]	896.41823
1065	[S].NGQAGAIEH.[-]	896.42207
1066	[D].GIDGGGGGGIH.[V]	896.42207
1067	[P].QPQPAMQP.[L]	896.42946
1068	[I].AAASPSAPAPG.[L]	896.44722
1069	[P].SPGPGPGLQS.[V]	896.44722
1070	[V].GPSSALPGDP.[Y]	897.43124
1071	[G].NASGLGAGPGP.[S]	897.44247
1072	[S].QVGAEVGHT.[A]	897.44247
1073	[A].GGIGGGTGPPAG.[P]	897.44247
1074	[V].QSPGIPQGN.[S]	897.44247
1075	[L].GPSAPAFPGP.[Y]	897.44649
1076	[E].GPAGAAGAQAGA.[R]	898.43772
1077	[K].GPSRDPGGVG.[T]	898.43772
1078	[D].LSFLPQPP.[Q]	898.50328
1079	[C].GAGVHFSAGP.[S]	899.43699
1080	[T].TASPTTGPPA.[V]	899.44689
1081	[R].AAPVGTGAGAGA.[R]	899.45812
1082	[G].SGPPPPPPGP.[P]	899.46214
1083	[T].PGSPGKIMP.[G]	899.46551
1084	[F].PSGASVPAASG.[Y]	900.44214
1085	[A].AAQPSTPAGT.[P]	900.44214

1086	[S].PGAGSLGSPAS.[Q]	900.44214
1087	[V].AAGAAAGAAAGAA.[A]	900.45337
1088	[V].AGPEKGGGSAA.[A]	901.43739
1089	[W].QPNLNSGAT.[P]	901.43739
1090	[T].PGAAGGATAASA.[A]	901.43739
1091	[L].VAGAGRGQW.[R]	901.46388
1092	[R].KRQGYIH.[E]	901.50026
1093	[Q].TAPAAASPGC.[Q]	902.40364
1094	[R].GAPGHRGHGG.[G]	902.43397
1095	[A].SPTLDAELG.[A]	902.44655
1096	[P].GAAGPAPHPQ.[W]	902.44789
1097	[T].AAPGPAQPGH.[V]	902.44789
1098	[S].GPAAACSRL.[R]	902.45126
1099	[G].KQADTAAVV.[E]	902.49417
1100	[Q].QPSATAKVT.[S]	902.49417
1101	[S].PGSSSRNLS.[T]	904.44828
1102	[G].PGGPPSAGPAP.[V]	904.45231
1103	[G].GPQAYVSPS.[E]	905.43632
1104	[G].PQGPQGPPQ.[G]	905.44756
1105	[M].PGGALEPHAG.[L]	905.44756
1106	[-].MGPAPARAEH.[R]	905.45879
1107	[Q].GPGAAAHPQT.[H]	906.44281
1108	[P].GPPWPPAQG.[P]	906.44683
1109	[L].AAAAAHTFF.[V]	906.44683
1110	[R].GPSGLPGPAGP.[P]	906.46796
1111	[G].QQGRAGHGP.[D]	907.44929
1112	[G].PGAGQGAVPGP.[P]	907.46321
1113	[A].AGVGGEAGPPP.[E]	908.44722
1114	[E].GPGSPGVPGSP.[P]	908.44722
1115	[G].GPGAGGLEGPP.[Q]	908.44722
1116	[Q].PTPASTTPH.[K]	908.44722
1117	[I].LDGGAPGPQP.[H]	908.44722
1118	[P].PGPSGPATQP.[P]	908.44722
1119	[S].PGGSPQTPPA.[F]	908.44722

1120	[P].GPSGPATQPP.[V]	908.44722
1121	[A].PGVPSGNGAPG.[P]	909.44247
1122	[P].GPGPGQVSGPG.[Q]	909.44247
1123	[G].APGGSGAPAPQ.[Y]	909.44247
1124	[Q].PSAQQPGQP.[E]	909.44247
1125	[R].GLGGEVPGSH.[Q]	909.44247
1126	[P].SGPPGPPGFP.[G]	909.44649
1127	[R].PGSPGLPGMP.[G]	909.44986
1128	[K].QPSGRGPGPG.[R]	909.4537
1129	[V].GPRGPSGPQG.[I]	909.4537
1130	[P].PGSPGPRGNA.[G]	909.4537
1131	[L].PGSGPGPGGRA.[A]	909.4537
1132	[L].PSGPSAGSGPP.[P]	910.42649
1133	[C].PSGPGTFTF.[H]	910.43051
1134	[Y].GPSPGGRGPE.[D]	910.43772
1135	[A].GSPPGPGADR.[A]	910.43772
1136	[E].GPSTAPPHF.[G]	910.44174
1137	[V].LGLSERHV.[A]	910.51049
1138	[P].AGGIGGGTGPPA.[G]	911.45812
1139	[A].GPAATPAQAQ.[A]	911.45812
1140	[-].MAAAAAAAPGPGS.[G]	911.45812
1141	[D].VGGAAAAPGGGAG.[G]	912.45337
1142	[E].VGAGAGPGAQAG.[P]	912.45337
1143	[L].PGFPGVNPQ.[A]	912.45739
1144	[V].GASTPAAGGPQ.[E]	913.43739
1145	[E].TAAQTPGGPGG.[P]	913.43739
1146	[A].GPGSPQGKEG.[T]	913.43739
1147	[S].PGSPGAGGVQS.[T]	913.43739
1148	[N].QPAELMPQ.[F]	913.44478
1149	[S].PGAGGGVNER.[R]	913.44862
1150	[L].PAGGGSARPGS.[Q]	913.44862
1151	[P].AGAPTDTLPA.[T]	913.46254
1152	[P].GSATPAVGSAP.[A]	914.45779
1153	[N].TASATAQAPP.[A]	914.45779

1154	[D].PGVGGTGLEQ.[G]	914.45779
1155	[P].ATTTGAAPPQ.[P]	914.45779
1156	[K].SATAVPSGPGA.[A]	914.45779
1157	[L].GMGNGAGTPGP.[G]	915.39889
1158	[T].HAGLSTAMQ.[P]	915.43528
1159	[R].GPSSTVSPPS.[C]	915.4418
1160	[P].QPGAYRSH.[P]	915.44314
1161	[E].GPAGNLARC.[A]	915.44651
1162	[V].PGSQSVGVQG.[E]	915.45304
1163	[G].QAVEAAAGAAG.[Q]	915.45304
1164	[G].DPAPGPPPAP.[S]	915.45706
1165	[M].VWEQGIAI.[I]	915.49344
1166	[Q].PGGGPPSPPPG.[I]	916.45231
1167	[G].QFSRPGPQ.[S]	916.46354
1168	[V].GPGPGGPGPAGP.[M]	917.44756
1169	[P].PGASGAPPHAG.[L]	918.44281
1170	[-].MSPGGPPRGGH.[R]	918.45404
1171	[E].GPAGGLYGID.[S]	919.45197
1172	[G].QAGLGPGSHP.[T]	920.45846
1173	[H].PQAAASYRG.[Q]	920.45846
1174	[G].PGGGAGVRSY.[P]	920.45846
1175	[G].PPGHGGTAQV.[T]	920.45846
1176	[P].QPQPSAVGH.[W]	920.45846
1177	[Q].PGSTALDLF.[K]	920.47237
1178	[F].APEKPGPQP.[S]	920.48361
1179	[Q].AATSHHLGQ.[N]	921.4537
1180	[K].APAFHAGGPP.[G]	921.45773
1181	[L].PGSEGPPGPAG.[S]	922.42649
1182	[Q].QREAPHAGG.[F]	922.44895
1183	[D].LGGPGEGAPAP.[G]	922.46287
1184	[S].ATPPPGASGAP.[P]	922.46287
1185	[A].GPSSPQRPP.[T]	922.47411
1186	[A].AASGGAAGAAGY.[P]	923.42174
1187	[T].PGPESSVPGP.[R]	923.44689

1188	[Q].GPASVPPSPD.[K]	923.44689
1189	[L].PGSPPSTPSP.[P]	923.44689
1190	[P].GPSGSPAIHT.[L]	923.45812
1191	[A].PGAGAASPAEP.[E]	924.44214
1192	[L].GPAGADRSPP.[S]	924.45337
1193	[T].PGGMALPGQP.[G]	924.46076
1194	[R].PGSPGLPGMP.[G]	925.44478
1195	[P].RAGPEGAGGGP.[G]	925.44862
1196	[S].KGQCPAPAP.[L]	925.45601
1197	[V].GTGAGAGARAH.[G]	925.45985
1198	[K].AAPEASPSVP.[C]	925.46254
1199	[S].AAPAAGSAPAAA.[E]	925.47377
1200	[V].LGEWGAVPP.[P]	925.47779
1201	[P].VPACALTPP.[R]	925.48116
1202	[L].SSPAAPTAQP.[K]	926.45779
1203	[R].SPPGSAVAGPS.[S]	926.45779
1204	[E].RSPADAGPVG.[R]	926.46902
1205	[H].AIAWSPGPGA.[F]	926.47304
1206	[Q].GLPPSLTEL.[H]	926.51932
1207	[E].LGNGTVPVGI.[A]	926.53056
1208	[M].GPSALGQSGPG.[S]	927.45304
1209	[F].PGAKGDAGTPG.[P]	927.45304
1210	[A].GIGGEPAAAGAG.[C]	927.45304
1211	[-].MAAAGSGPGPGVS.[A]	927.45304
1212	[R].ATQTVSPSH.[S]	927.45304
1213	[L].PGSPGAKGEQ.[G]	927.45304
1214	[P].QPGNGSLSPA.[Q]	927.45304
1215	[G].PSGPGGKGSPS.[E]	927.45304
1216	[I].KDPGSVPMP.[Q]	927.46043
1217	[R].HDCDLLR.[E]	928.43053
1218	[S].APGGAFQPSP.[W]	928.45231
1219	[Y].FPGGTAPGAPG.[P]	928.45231
1220	[Q].QPSTELGTP.[T]	929.45745
1221	[Y].GPVSGAVSGAK.[P]	929.50507

1222	[E].SAPSAVSQPS.[S]	930.4527
1223	[P].PGAAMSVQVA.[A]	930.47133
1224	[L].GPTGSGKTLL.[A]	930.52547
1225	[P].PGSGSQAAAVS.[V]	931.44795
1226	[G].GPSAKQASGE.[A]	931.44795
1227	[R].GPAGPPGPQGP.[P]	931.46321
1228	[G].AAAFSGAVTH.[S]	931.46321
1229	[-].MQGLGCGVLQ.[Q]	931.46658
1230	[V].PGRAGGQAAF.[L]	931.47444
1231	[T].INAWFGPQ.[G]	932.46248
1232	[G].AATASVGGAEV.[P]	932.46835
1233	[G].GPATSQASTL.[D]	932.46835
1234	[T].KYMPQPPG.[W]	933.44986
1235	[V].GPAGPRGPAGP.[S]	933.49009
1236	[P].GPSGPPAAPSP.[V]	934.46287
1237	[Q].PGPSGPPAAPS.[P]	934.46287
1238	[G].PGAGAAPEPPA.[S]	934.46287
1239	[A].SPGPASPGPAP.[R]	934.46287
1240	[Y].PGMLYIPQ.[D]	934.47027
1241	[G].GVGTGPVPGPP.[F]	934.49926
1242	[S].LSPPAAQPGP.[P]	934.49926
1243	[K].PGAPGGSGAPAP.[Q]	935.45812
1244	[M].KGHSGAPGPAG.[L]	935.46935
1245	[N].PGSPGGHACP.[A]	936.39923
1246	[L].LLGGPTGAPPG.[G]	936.51491
1247	[P].GPGSPPRIR.[G]	936.53737
1248	[A].PNVGASWAH.[D]	938.44789
1249	[Q].GPEPGLSGGPA.[G]	938.45779
1250	[V].GPADQTVPPG.[S]	938.45779
1251	[G].GGLGSGVSTGF.[L]	938.45779
1252	[Q].GPSPGPGLQE.[A]	938.45779
1253	[A].PGAPGTPGER.[G]	938.46902
1254	[R].SDPGQVSSY.[F]	939.40542
1255	[P].GPSSPQVPGGG.[C]	939.45304

1256	[T].AGVQEPGAPGG.[G]	939.45304
1257	[A].GPGQGAEGLPG.[T]	939.45304
1258	[A].PGGATGQAPPS.[S]	939.45304
1259	[G].GAAGVGGEAGPP.[P]	939.45304
1260	[A].GPAPYSPPGP.[G]	939.45706
1261	[E].APGGGPSSRGP.[C]	939.46427
1262	[P].APGRQEGPAG.[A]	939.46427
1263	[P].GPAAHVYPAG.[S]	939.46829
1264	[P].AAPAGWAAPQ.[D]	939.46829
1265	[E].GPRGDRGPQ.[G]	939.4755
1266	[P].GPGPSPGAMLG.[P]	940.45568
1267	[W].RGGSGAGPAGGP.[P]	940.45952
1268	[A].AAAAAAPETAP.[S]	940.47344
1269	[L].AAQEALPLAG.[R]	940.50982
1270	[T].TAPSGGLPQL.[L]	940.50982
1271	[N].NIENLPLQ.[L]	940.50982
1272	[G].PGGAGGARGGAGG.[G]	941.45477
1273	[G].PGSPETASPV.[A]	941.45745
1274	[L].AAEQRMVH.[R]	941.46216
1275	[F].ADVAVGAPLE.[D]	941.49384
1276	[F].SGTPALGEPL.[T]	941.49384
1277	[E].GVSLGDIAPL.[P]	941.53022
1278	[L].QPPGGTAGEE.[V]	942.41632
1279	[G].AAAAEPGTASP.[A]	942.4527
1280	[V].GPGQSGGLGGVG.[A]	942.46393
1281	[S].GVSAAGGGPAGAA.[G]	942.46393
1282	[G].PGSVGPREGS.[W]	942.46393
1283	[A].TGGGAAQVGQP.[A]	942.46393
1284	[T].TAGANVHATT.[A]	942.46393
1285	[T].GLGTAAGGGGPGA.[L]	942.46393
1286	[C].AASPSGLWPG.[G]	942.46796
1287	[G].PGSGASEGPGGA.[F]	943.41156
1288	[A].GGGQGVLGGAGGG.[N]	943.45918
1289	[E].QVGGERSGGP.[E]	943.45918

1290	[M].PGPGPGPGPGPG.[P]	943.46321
1291	[P].GPGPGPGPGPGP.[G]	943.46321
1292	[P].PGAGMLGFPP.[S]	943.4706
1293	[P].PQPEVSVTS.[T]	943.4731
1294	[L].TAGPASRFH.[A]	943.47444
1295	[N].RGAGVGCVPA.[V]	943.47781
1296	[L].KPPSNSSVK.[R]	943.52072
1297	[P].GPSPSSKPTS.[D]	944.46835
1298	[SA].QPATGTTATP.[K]	944.46835
1299	[V].NVGGQAQAVT.[G]	944.47958
1300	[H].VNGPTGGSKK.[P]	944.51597
1301	[E].GGAVGSEIAGGA.[G]	945.4636
1302	[L].KDGNPVSTAG.[T]	945.4636
1303	[Q].GAGDIWLDV.[Y]	945.46762
1304	[L].PGAGAAGTSTR.[L]	945.47483
1305	[R].PGPSPGPGPSP.[G]	946.46287
1306	[Y].SGPPPGPGPSP.[V]	946.46287
1307	[L].GAPDPPGAAPP.[G]	946.46287
1308	[T].GPSVTNPFQ.[P]	946.46287
1309	[P].GPGPAEPPAPG.[E]	946.46287
1310	[H].GPSAPHPTSP.[T]	947.45812
1311	[M].AQAPAQSQF.[L]	947.45812
1312	[P].GPGPGPGPGPAS.[L]	947.45812
1313	[A].GPPGSGGQPPP.[R]	947.45812
1314	[G].GAAAARAWSS.[P]	947.46935
1315	[L].QRQGQYAP.[P]	947.46935
1316	[T].RQAGHHEL.[A]	947.48059
1317	[M].AAAPGRPGPPG.[K]	947.50574
1318	[A].AAEAGAGEDAS.[S]	948.39049
1319	[R].PGSGPDQYQ.[D]	948.40575
1320	[L].RAGEHQPGP.[A]	948.4646
1321	[P].APAPAEAPGAP.[G]	948.47852
1322	[G].PGSPGVPGSPP.[E]	948.47852
1323	[A].PGGAGGGLLYS.[R]	948.47852

1324	[D].GSPGPAPAPTP.[G]	948.47852
1325	[R].GPAPREAPPG.[E]	948.48976
1326	[L].PGSGPGPGGLPG.[S]	949.47377
1327	[G].GAPAPQPSPQ.[G]	949.47377
1328	[S].PGAGSPHLSQ.[G]	950.46902
1329	[P].APGSTVQPGH.[H]	950.46902
1330	[I].PGMPGLPGAPG.[K]	950.47641
1331	[L].GAVGEPHRQ.[R]	950.48025
1332	[K].PGLDGLPGAPG.[D]	950.49417
1333	[G].TAPSGVPGAPP.[L]	950.49417
1334	[E].ELVGPGPGPGA.[T]	950.49417
1335	[T].AAAAASAPAPGP.[A]	951.48942
1336	[P].GPSGVPPGMPG.[Q]	952.45568
1337	[-].MQAPGGVPGVE.[A]	952.47344
1338	[E].GPTTGPTGPPA.[A]	952.47344
1339	[T].PAGSTGQVVH.[G]	952.48467
1340	[Q].EAAQRPSPP.[S]	952.48467
1341	[P].PGPSGGGYDF.[G]	953.39994
1342	[Q].PGSPGLSGQPG.[L]	953.46869
1343	[-].MAAAAAAAPGPGS.[G]	953.46869
1344	[H].LEPASTAGAH.[K]	953.46869
1345	[-].MSSAPAQGPAPA.[S]	953.46869
1346	[K].GLNGPTGPPGS.[P]	953.46869
1347	[Q].GSPQPLGSGGP.[G]	953.46869
1348	[L].APNTRGSPGP.[P]	953.47992
1349	[V].PGPGSGGPGGSAG.[R]	954.42755
1350	[P].PGAPPHAGGPP.[P]	954.47919
1351	[V].APTAQPEEL.[L]	955.4731
1352	[D].AAPASQQPSV.[I]	955.48434
1353	[S].FLPQPPQE.[K]	955.48836
1354	[R].NKGGGALGGGPA.[L]	955.49557
1355	[S].EGNALCRH.[V]	956.43667
1356	[H].QVGGHSCVL.[R]	956.46183
1357	[A].GPSPASTPATA.[D]	956.46835

1358	[K].AAAATAQAQPG.[P]	956.47958
1359	[G].AAAAAAGQPGTA.[P]	956.47958
1360	[L].QAPAASAGGVQ.[L]	956.47958
1361	[H].AAAEAGQLLI.[L]	956.54112
1362	[L].PGSTLQGSPN.[A]	957.4636
1363	[S].PGAGSRAENV.[C]	957.47483
1364	[P].GAPFLGMAPP.[V]	957.48625
1365	[K].KPFLGMPAP.[L]	957.52264
1366	[V].AATPDSNSVP.[A]	958.44762
1367	[P].PGALGSPNMV.[N]	958.46624
1368	[A].TAPGPAGIAMG.[S]	958.46624
1369	[P].QPGARASGQS.[T]	958.47008
1370	[P].GPSPMYPLP.[T]	958.47027
1371	[F].QPTLEEIE.[E]	958.47277
1372	[P].AAAGEIPGTAT.[R]	958.484
1373	[P].PNASGVSVASA.[A]	959.47925
1374	[P].GPATSHPHR.[D]	959.48059
1375	[L].PGLMDIQAV.[D]	959.48664
1376	[G].QPLCTLEV.[G]	959.48664
1377	[T].PGSPSSKGSR.[G]	959.49048
1378	[G].PGGQATNKTS.[N]	960.4745
1379	[-].GPVASTASALS.[V]	960.49965
1380	[C].PGSGEAGLGGC.[S]	961.40437
1381	[P].QPPDPSQPP.[P]	962.45779
1382	[L].PGAPGQGGAPGP.[P]	962.46902
1383	[Q].TAAPGSISTGT.[T]	962.47892
1384	[G].AAAPGPDRAH.[R]	962.48025
1385	[E].GPAGAAAHSQP.[V]	963.46427
1386	[V].PQPFPSGPH.[L]	963.46829
1387	[L].GPIMAHPQP.[E]	963.47166
1388	[A].APATAEGTVF.[R]	963.47819
1389	[I].QPAPTAQPPG.[P]	963.48942
1390	[M].PGANGGDFET.[Y]	964.40067
1391	[D].PGGGQGPGGGQP.[R]	965.44353

1392	[P].AAAPDPAAAGGP.[P]	965.46869
1393	[S].TGLGSPEAPH.[P]	965.46869
1394	[Q].AARDGPAGPGP.[E]	965.47992
1395	[P].PGRPGSPGSPG.[L]	965.47992
1396	[P].RGPGQGSGHL.[A]	965.49115
1397	[H].PSGPQAPQGAG.[R]	966.46393
1398	[P].PGSPGPRGNAG.[G]	966.47517
1399	[D].FGFDGDFY.[R]	967.38322
1400	[ND].SESMRGDW.[G]	967.39381
1401	[Q].DDQGPQGPPG.[T]	967.41156
1402	[L].GPAGEGVVGAPG.[F]	967.48434
1403	[D].PGGLSPGSAPQ.[D]	967.48434
1404	[A].AAAAEVPLPE.[S]	967.50949
1405	[P].GPSGVPPGMPG.[Q]	968.45059
1406	[E].SGPGISPGAEP.[P]	968.46835
1407	[A].SGPETPLGGPG.[G]	968.46835
1408	[-].MAAAAAAAPGPGSG.[P]	968.47958
1409	[Q].NTPAPGVGAAGG.[S]	968.47958
1410	[I].QAAAGTQPGPA.[A]	968.47958
1411	[G].QVSGGPAQPQ.[A]	968.47958
1412	[Y].PGNAGPVGTAGA.[P]	968.47958
1413	[V].PGSPGFPGVPG.[S]	968.48361
1414	[L].SAAPAWAGAAP.[V]	969.47886
1415	[Q].PAGPEGTLVE.[M]	969.48875
1416	[G].RPGATMIAH.[E]	969.49346
1417	[A].SGPGLNASGIP.[A]	969.49999
1418	[N].GPSVGEIPQS.[E]	970.484
1419	[S].AASSPEALAPG.[W]	970.484
1420	[I].VGEAGGSPSPL.[L]	970.484
1421	[Q].IMGPGQAPGQ.[R]	971.46149
1422	[A].GSPATAAGPATA.[T]	971.47925
1423	[R].GPSAVSTQAPG.[T]	971.47925
1424	[P].PGPSGPLGHPG.[L]	972.48976
1425	[G].PHFGHPGPGA.[D]	973.46388

1426	[-].MAGLGKMGPGQ.[C]	973.47714
1427	[S].RGAGAGGRSAGG.[G]	973.49221
1428	[W].AAEAAAAAAAVS.[G]	973.4949
1429	[A].VSPAQQHAH.[L]	974.48025
1430	[S].QPPSPPSPAP.[S]	974.49417
1431	[P].PGSGVAGLGFL.[L]	974.53056
1432	[V].PGAGTGVASSAT.[E]	975.47416
1433	[A].GPRPPGSGPGP.[G]	975.50065
1434	[F].GFDGDFYR.[A]	976.41592
1435	[P].GPSPGPGPSPGA.[M]	977.46869
1436	[S].PGRGQASPAH.[Q]	977.49115
1437	[Q].EPPAGGGGSIH.[D]	978.46393
1438	[G].PAGAWHPRS.[Y]	978.49042
1439	[G].AAPAAAEGPAPG.[S]	979.48434
1440	[G].GPSGGPTVGGPP.[P]	979.48434
1441	[H].PGAALPPGDGQ.[Q]	979.48434
1442	[P].APGAGPPREAG.[S]	979.49557
1443	[G].APGVPSGNGAPG.[P]	980.47958
1444	[M].GPSVADLAPPG.[E]	980.50474
1445	[S].GPSGTGKTYL.[A]	980.50474
1446	[R].GPAGDSPVPGAG.[T]	981.4636
1447	[G].AAGGAAGGGPAAGP.[A]	981.47483
1448	[R].PGSPGLPGMPG.[R]	982.46624
1449	[G].PGQRGDAGGAP.[P]	982.47008
1450	[L].PGAGADLAVDP.[D]	982.484
1451	[A].AAAAAAGGQQAP.[E]	983.49048
1452	[G].GPTSLGGGGAGGP.[L]	984.4745
1453	[T].LESHLMSPA.[E]	984.48189
1454	[N].GVDLGPICGP.[P]	984.48189
1455	[R].GVARAGEGPGGG.[Q]	984.48573
1456	[Q].PASSATLSPGP.[T]	984.49965
1457	[Y].LDSHISDSL.[S]	986.47892
1458	[S].PGAEPGPPPPA.[V]	986.49417
1459	[G].GAGQAGARGWG.[P]	987.4755

1460	[A].AAAAERAEAAG.[F]	987.4854
1461	[F].PNSGLQAGSAS.[L]	988.46941
1462	[K].PGAATGPATMV.[L]	988.47681
1463	[A].GAGPGPGPGAPPG.[L]	988.48467
1464	[P].GPGPGPGQAPPG.[G]	988.48467
1465	[A].QPPELPGPPG.[F]	988.50982
1466	[P].QPQAQGEHP.[D]	991.45918
1467	[A].PGGSPPAQPPS.[T]	991.48434
1468	[S].PGAGAAGISPGH.[S]	991.49557
1469	[V].QRTAHANPP.[T]	991.5068
1470	[P].SPGPQQAPPGG.[T]	992.47958
1471	[D].GPWPGGEPPV.[T]	992.48361
1472	[N].PGWPGTPGAPG.[P]	993.47886
1473	[P].GSPGPAGSPGLP.[G]	993.49999
1474	[L].APGAAEVPGQP.[R]	993.49999
1475	[L].DGIPAAAPGQP.[V]	993.49999
1476	[L].GPSGRADPTH.[L]	994.47008
1477	[G].APGGEEVAAPP.[R]	994.484
1478	[T].GPSPLDSAPPG.[G]	994.484
1479	[A].PGGAQTQAPAP.[A]	994.49523
1480	[C].PGSALGGPGGPE.[Q]	995.47925
1481	[V].AQAAAGAPQNP.[R]	995.49048
1482	[A].GAAGAQAAPQGP.[A]	995.49048
1483	[S].SPRAMPPSPG.[P]	996.49313
1484	[P].RPCTPAPTP.[P]	996.49313
1485	[A].GPATTTTPPGP.[P]	996.49965
1486	[Y].PGSPVPTSSAP.[L]	996.49965
1487	[E].AGPDVGVSTPP.[A]	996.49965
1488	[P].VDAEALELH.[D]	996.49965
1489	[H].GLGHPHAGPPG.[S]	996.50099
1490	[D].PTDEFFLK.[D]	996.50367
1491	[V].QGPPGHPGPPG.[E]	997.485
1492	[G].PGTGGPGVASPT.[I]	997.4949
1493	[E].PGAAGQAELSP.[G]	997.4949

1494	[L].PGAPGIDGEKG.[L]	997.4949
1495	[Q].PGGPAPSAVSSA.[Q]	997.4949
1496	[A].AAQPSTPAGTP.[R]	997.4949
1497	[S].AAPGSAAPAAGSA.[P]	998.49015
1498	[S].PAGSPPSSLW.[E]	998.49417
1499	[A].GAPGVQGYPGP.[P]	999.48942
1500	[L].QPITYGPSH.[S]	999.48942
1501	[L].GGPGGAGWIDL.[S]	999.48942
1502	[Q].PGAGQPVVMQ.[P]	999.49279
1503	[K].PGYPGEPGLN.[G]	1000.47344
1504	[K].GPGSPPASMLA.[L]	1000.47681
1505	[A].AGPGAESRAGGA.[A]	1000.48065
1506	[G].PSPGPPSAGHP.[I]	1000.48467
1507	[P].GPGPGPGPGPGPG.[P]	1000.48467
1508	[A].GPSGVSAVDAAA.[S]	1001.48981
1509	[S].GVGGRFGQEP.[S]	1003.49557
1510	[F].WVTTFVNH.[P]	1003.49959
1511	[E].PGSPGEPPPGL.[A]	1004.50474
1512	[P].PGPSGPATQPP.[V]	1005.49999
1513	[P].GPSPASPAPPE.[G]	1006.484
1514	[S].GPGPGGLPGSGPG.[P]	1006.49523
1515	[V].PGAVGGVSPEH.[A]	1006.49523
1516	[S].PAGGGGLAFYP.[G]	1006.49926
1517	[R].AATLMQLCT.[S]	1008.48526
1518	[Y].KNYFTAGAH.[W]	1008.48976
1519	[C].GPPADAVTSPP.[R]	1008.49965
1520	[P].GPSPGPALSLD.[A]	1010.5153
1521	[P].GPSGNMGPQGP.[K]	1014.43092
1522	[D].GPAEETVNTP.[L]	1014.47383
1523	[S].PGSPGAGGVQST.[S]	1014.48506
1524	[T].NTATTAGPAPGG.[P]	1014.48506
1525	[R].AANSSSRPPAG.[L]	1014.4963
1526	[G].AAAGPAPQGGAF.[S]	1014.50032
1527	[R].AVNEGVQQAV.[A]	1014.52145

1528	[A].GPRGHPGPSGP.[P]	1015.5068
1529	[F].EAVNQVHGY.[M]	1016.47958
1530	[A].TPAGPARSPY.[Q]	1016.51597
1531	[Q].GPAGPTTSPAY.[S]	1018.484
1532	[-].MAATPQSLFPS.[G]	1018.52039
1533	[S].PGAPEVGAAPPG.[R]	1019.51564
1534	[G].AGAAGISPGHSP.[L]	1021.50613
1535	[Q].EPAAAPGAQGPG.[S]	1022.49015
1536	[M].PGAQGAPAAGPE.[P]	1022.49015
1537	[G].TPGQPGSPGPAG.[V]	1022.49015
1538	[E].AAAAEPNPNAP.[G]	1022.49015
1539	[Q].LSWTPGGPAH.[L]	1022.50541
1540	[L].GPSGRAQAHAA.[I]	1022.51262
1541	[S].QAAPTSTFSL.[P]	1022.5153
1542	[A].WSPVRPGAPG.[Q]	1023.53704
1543	[P].AVNPSGSPAGPA.[G]	1024.5058
1544	[R].PGVSGASQPPQ.[A]	1024.5058
1545	[H].PGASSLAASPAP.[P]	1025.5262
1546	[K].QVLSDPAGAAP.[R]	1025.5262
1547	[L].GTSPALGRGLP.[R]	1025.57382
1548	[D].LSFLPQPPQ.[E]	1026.56186
1549	[G].GPSVGPADQTV.[P]	1027.50546
1550	[P].GPAPGEVTAASA.[G]	1027.50546
1551	[R].GPAGPPGPQGPP.[G]	1028.51597
1552	[L].PAGAAAAAESLT.[L]	1029.52111
1553	[I].PGVGSSGSRGGL.[L]	1030.5276
1554	[G].PGATSSQVARG.[L]	1030.5276
1555	[L].PGPGEPVPGPAG.[P]	1031.51564
1556	[N].SPSAAPAFTSP.[P]	1032.49965
1557	[G].SKEATTMLH.[M]	1033.49827
1558	[G].GPAAGPADHGLA.[G]	1033.50613
1559	[P].GPAAAFSVSVE.[R]	1034.5153
1560	[Q].AAGTHLGGTNH.[D]	1035.49663
1561	[Q].SSPAGGPTPAPP.[T]	1035.51055

1562	[E].GPSPPQAPESA.[G]	1037.48981
1563	[A].QPQTMAPPPA.[A]	1037.50844
1564	[W].RGGSGAGPAGGPP.[-]	1037.51228
1565	[G].PGVQGWPPSGG.[L]	1038.50032
1566	[S].KEQGNEKAH.[G]	1040.51195
1567	[Q].PGAGGGQSLPMA.[W]	1042.49861
1568	[Q].GPAGTSVAGPQT.[S]	1042.51636
1569	[S].LDSPGQPSKGG.[F]	1042.51636
1570	[L].SFLPQPPQE.[K]	1042.52039
1571	[H].FSPPPSTSAPG.[G]	1044.49965
1572	[Q].KDPSAHYEV.[K]	1045.4949
1573	[I].LDGGAPGPQPH.[G]	1045.50613
1574	[L].PGSTAYARSH.[G]	1046.50138
1575	[Q].GPQNKQPFM.[V]	1046.50878
1576	[F].GFDGDFYRA.[D]	1047.45304
1577	[A].PGPSTGRTTSS.[E]	1047.50653
1578	[L].PGIPGPMGPPGA.[I]	1047.52918
1579	[S].APGQAGLGPGSH.[P]	1048.51703
1580	[L].ASGAQPVHGGPA.[L]	1048.51703
1581	[T].AAAAAATTTSAAA.[T]	1048.52693
1582	[P].AAAASFPAWSA.[F]	1049.50507
1583	[A].ELPAPAGPGPGS.[G]	1049.5262
1584	[A].SPASAPAPAPSP.[A]	1049.5262
1585	[F].QGLANLGETF.[M]	1049.5262
1586	[T].GPSPLDSAPPGG.[T]	1051.50546
1587	[-].MQAVNHSSPLG.[L]	1051.5167
1588	[P].TYAETLSTAP.[L]	1053.50988
1589	[N].GGAWAAEAAPPG.[P]	1054.49523
1590	[G].PGGGQPRTGMP.[A]	1054.50984
1591	[A].AAAAGQPGTAPSG.[V]	1055.51161
1592	[Q].SGPPLHHSGAP.[P]	1056.52212
1593	[A].PGAGVSSAVSQP.[A]	1056.53201
1594	[F].ATAALEPGDAAA.[A]	1057.51603
1595	[Q].EARSSLGNAPG.[P]	1058.52251

1596	[L].PGPQGPTGPPGP.[P]	1058.52653
1597	[R].AGTAPPAAFGGAA.[C]	1058.52653
1598	[K].KASYSGVSLF.[S]	1058.55169
1599	[E].VSGGPGHPTPPG.[H]	1059.52178
1600	[L].PGAPGQGGAPGPP.[G]	1059.52178
1601	[A].AGAGPGPGPGAPPG.[L]	1059.52178
1602	[E].PQSTPAFNAQ.[P]	1060.5058
1603	[P].AREGGGSCRL.[L]	1062.5109
1604	[P].AGPATPPPNSGP.[Q]	1062.52145
1605	[H].GPASQPPSPGPA.[V]	1062.52145
1606	[H].QPKDFTQAQ.[R]	1062.52145
1607	[M].GVAGSYGGAPGLG.[G]	1062.52145
1608	[M].GPAGPPGPAGER.[G]	1062.53268
1609	[L].APGSAGLAPGHQ.[V]	1062.53268
1610	[A].GPAAEIPASGGH.[G]	1063.5167
1611	[Q].AVDQALRGMS.[D]	1063.52007
1612	[P].GIDQAVFQW.[E]	1063.52072
1613	[V].RQYFIEAH.[E]	1063.53195
1614	[-].MPAVMQMLRS.[G]	1064.52271
1615	[S].PGSAALSTYTP.[E]	1064.52587
1616	[T].PGPGHPTHPAP.[P]	1064.5272
1617	[L].QEVGTPAGVAH.[T]	1065.53235
1618	[G].AGGPAPAASAAPAG.[G]	1065.53235
1619	[Q].GPAGVPGRDGSP.[G]	1066.5276
1620	[R].GPAGLYTHPGP.[V]	1066.53162
1621	[D].GRTGQPGAVGPA.[G]	1067.55923
1622	[D].LPPDTALLDL.[Q]	1067.5983
1623	[S].PATAGSPATAAGP.[A]	1068.53201
1624	[F].RSDSAVPLSH.[W]	1068.54325
1625	[V].AAAAAGGAGPGAGIG.[S]	1068.54325
1626	[E].SGPATLHLCN.[D]	1069.5095
1627	[H].EVTSQAAHLN.[L]	1069.52726
1628	[L].GGLPEGGRVDL.[P]	1069.56365
1629	[S].PGAGGAFPGLLL.[L]	1069.60406

1630	[S].QPSTGQYPPP.[T]	1071.51055
1631	[A].QDPFPTVGPN.[S]	1071.51055
1632	[P].QNPGAFVQNP.[S]	1071.52178
1633	[P].LGGGGAGGPQMGL.[P]	1071.52515
1634	[Q].PGAVQEIAASE.[I]	1071.53168
1635	[E].QPPGMMPNGQ.[D]	1072.45503
1636	[R].PGPSPGPGPSPGA.[M]	1074.52145
1637	[K].PGPGPSQPGPSP.[L]	1074.52145
1638	[P].SPPNKPGSPPP.[P]	1074.55783
1639	[K].GPQGPGQPSGPP.[P]	1075.5167
1640	[A].GPAGPYTREAG.[F]	1075.5167
1641	[A].PGATSVSPGRM.[F]	1075.52007
1642	[S].PGSPPPWPSPG.[S]	1075.52072
1643	[G].PGGPQHAPFAP.[P]	1075.53195
1644	[G].PGSPGVPGSPPE.[A]	1077.52111
1645	[E].FSGFLTHTPA.[K]	1077.53637
1646	[G].SPGAGAAGISPGH.[S]	1078.5276
1647	[S].PGAGAAGISPGHS.[P]	1078.5276
1648	[E].ATPAANLTFSS.[Y]	1079.53677
1649	[W].PGSPQVSGPSPA.[T]	1080.53201
1650	[P].QAVGQSSGPPPG.[G]	1081.52726
1651	[R].PGTAQRGSPGGP.[E]	1081.5385
1652	[L].GAPATVSLAASH.[P]	1081.56365
1653	[P].AAEAAPESSLH.[T]	1082.51128
1654	[P].GPVGQMGPVGAP.[G]	1082.52991
1655	[P].GPSPGAMLGPSP.[G]	1083.51392
1656	[Q].QPATAEQTAAP.[A]	1084.52693
1657	[V].PGAAFPEKPGN.[A]	1084.54218
1658	[L].PSSAAAAPQQAS.[P]	1085.52218
1659	[A].AAQFSAPEPAP.[R]	1085.5262
1660	[P].AGAAGAQAGAREG.[V]	1086.52866
1661	[P].GPSPGHQPVGPG.[E]	1086.53268
1662	[P].PGAATASVGGAEV.[P]	1086.54258
1663	[S].GPAAASGPLATSS.[P]	1086.54258

1664	[D].GTISGGLCVSH.[S]	1087.52007
1665	[E].PGFGSGLPMAPG.[G]	1087.52409
1666	[A].KGETHRTVC.[L]	1087.5313
1667	[K].GPGAATSSRPST.[T]	1088.53308
1668	[Q].SPVNTPPSPGH.[A]	1089.53235
1669	[T].VSNSSEGRASP.[H]	1090.51234
1670	[A].PQPSPDPQPAG.[S]	1090.51636
1671	[R].LVREIAQDF.[K]	1090.58914
1672	[H].AASSMSVTIQP.[S]	1091.54014
1673	[L].PQAASQPAPGAP.[H]	1091.548
1674	[S].PGPGLQGSAPNP.[R]	1091.548
1675	[R].AAAGAGAGQLPAH.[H]	1091.55923
1676	[A].PGPGSGPARAPQ.[P]	1091.55923
1677	[A].GPPSGGASPTPPA.[A]	1092.53201
1678	[A].AATPPEGAPGAGP.[P]	1092.53201
1679	[S].ANPSPERPPGA.[Q]	1092.54325
1680	[S].AGGLGGGLGGSSGF.[G]	1093.52726
1681	[P].GPSGPATQPPVS.[S]	1094.54766
1682	[G].AGGAGGMGPGLPGP.[V]	1095.52515
1683	[A].QPAHHPPQSP.[A]	1095.53302
1684	[Q].VGQAAYSASKGG.[I]	1095.54291
1685	[Y].AEAAAAQAPAAGP.[G]	1095.54291
1686	[P].PGTKGNQGPSGP.[Q]	1096.53816
1687	[P].GEARGERPGPA.[C]	1096.5494
1688	[L].SVGHVSFHGLG.[P]	1096.55342
1689	[G].PGGGARSGGGRPA.[A]	1096.56063
1690	[W].KNSPQKNPQG.[K]	1097.5698
1691	[Q].SPGQAPYPGPQ.[Q]	1098.52145
1692	[P].QGAAPGPFAWP.[H]	1098.53671
1693	[G].AGQAGARGWGPA.[P]	1098.54392
1694	[P].GGPAPSAVSSAQA.[L]	1099.53783
1695	[G].SGRGGAPGEAGVS.[G]	1101.52833
1696	[S].GDGGKAGGQGAAGV.[D]	1101.52833
1697	[I].LDGGAPGPQPHG.[E]	1102.5276

1698	[G].PPGAMGPPGPPGA.[P]	1102.53499
1699	[S].GPGPGGLPGSGPGP.[G]	1103.548
1700	[P].QPGAHAHPHPG.[G]	1105.5286
1701	[I].SPQNVDVNVH.[P]	1108.53816
1702	[D].GPQNLPCVPQ.[W]	1109.5408
1703	[K].GPKMPLGFTF.[S]	1110.56523
1704	[S].GLGGGGVMLVHD.[I]	1111.55645
1705	[N].ISNNAGNTPGPA.[K]	1112.53308
1706	[A].AAGPAPSQAGAGSA.[P]	1112.53308
1707	[G].PRGHPGPSGPPG.[K]	1112.55957
1708	[E].PSVGAGGATYAY.[P]	1113.52111
1709	[-].MQPGNETRQPS.[H]	1113.52833
1710	[T].GPGAGGEKAVSQG.[F]	1114.54873
1711	[D].SRGAGGGGSGALPA.[G]	1114.55996
1712	[P].GPQGPTGPPGPPG.[V]	1115.548
1713	[N].GPNQLMSLQQ.[F]	1115.55137
1714	[V].NPPPPEVSNPA.[K]	1118.54766
1715	[P].PQEPAPGAPQQ.[T]	1119.54291
1716	[P].KGSGHPSGPQAP.[Q]	1119.55415
1717	[P].PGSPGPRGNAGGP.[G]	1120.5494
1718	[G].QRGPVGLPGSPG.[A]	1121.60618
1719	[L].QPSPGTTLGPPA.[A]	1122.57896
1720	[S].GPAGVDASALPPA.[V]	1122.57896
1721	[H].PGASSLAASPAPP.[P]	1122.57896
1722	[D].FGFDGDFYR.[A]	1123.48434
1723	[P].GPSPGAMLGPSPG.[P]	1124.54047
1724	[L].PGSPEGAGKSPAA.[R]	1125.55348
1725	[I].PNSPAPVTETN.[V]	1126.53749
1726	[R].RPHPAPGPGAGN.[I]	1127.57047
1727	[T].QREFMLSFA.[R]	1128.55064
1728	[T].NKDSTGSLPGPG.[S]	1129.54839
1729	[V].SQTPVATASGPN.[F]	1129.54839
1730	[G].PGSGPARAPQPP.[D]	1131.59053
1731	[P].RGNAPSPAAPPP.[P]	1131.59053

1732	[P].GPPGSPAPQNVN.[K]	1134.55381
1733	[A].AAAAERAEAAGF.[A]	1134.55381
1734	[S].PGAGSPPGPGADR.[A]	1135.54906
1735	[L].PGVSGPAGAPGGGR.[T]	1136.5807
1736	[Q].PGMVQPDIGQP.[D]	1138.55612
1737	[Y].FPGGTAPGAPGPGG.[P]	1139.548
1738	[R].QNPGGPSSSVPL.[L]	1139.56913
1739	[K].SLVDLQLTHN.[K]	1139.60551
1740	[L].PGATPPSHAPGGP.[Q]	1142.5589
1741	[T].GPGKWEGARNA.[I]	1142.57013
1742	[E].DKGGGPPRCSI.[A]	1143.55752
1743	[M].VPIEEAAQEY.[Q]	1148.547
1744	[A].GPGPSLGDEAIH.[C]	1149.55348
1745	[A].KGGDGKLFKTV.[L]	1149.66263
1746	[L].PQGEKVQAMY.[I]	1150.55612
1747	[L].PGSPGAKGEQGPA.[G]	1152.56438
1748	[V].PGSPGFPGVPGSP.[G]	1152.5684
1749	[A].RSGNLTFMVGG.[V]	1154.56227
1750	[Q].NLNLEESAPAP.[F]	1154.56879
1751	[P].LSPQGGGGGVAPGT.[A]	1154.58003
1752	[D].LSFLPQPPQE.[K]	1155.60445
1753	[K].GTEDELDKYS.[E]	1156.50044
1754	[L].LEQAMIGPSPN.[P]	1156.56668
1755	[L].PGSLGRAMGGQQ.[E]	1158.56842
1756	[S].GPAQRLHMGHG.[A]	1160.57417
1757	[A].PAGSVTATSSPSV.[V]	1160.57936
1758	[P].GPGPPSQPSLPQ.[D]	1161.58986
1759	[S].GPSPGDRTVGYG.[Y]	1162.54873
1760	[N].PGLMAPSQFAAG.[K]	1162.55612
1761	[S].PGLSQPSGVYAS.[S]	1162.57388
1762	[T].PGSTAAAPAGLHN.[S]	1163.58036
1763	[M].QPPVTQAGAAAGP.[C]	1164.60076
1764	[G].AGAAGSQGPAGGPAP.[D]	1165.55963
1765	[V].TAPGAPSLQASAP.[G]	1167.60043

1766	[G].GPQNLSGPGGRE.[R]	1168.57052
1767	[V].KDLIDEGHAAT.[Q]	1169.57969
1768	[A].EGRADPPGQPF.[K]	1170.55381
1769	[A].GPQERGVWGQG.[R]	1170.56505
1770	[H].DKENKDVPNGG.[V]	1172.55421
1771	[D].GPGSPFGLEPQS.[G]	1172.55823
1772	[T].PNPSPAPHNTAA.[G]	1173.56471
1773	[P].GPATSHPHRDV.[Q]	1173.57594
1774	[E].PQEKTPKSCV.[E]	1173.59323
1775	[R].GPGSPSVDSSSVV.[T]	1174.55862
1776	[H].PGSPSEKVESAS.[P]	1174.55862
1777	[S].PGAGAAGISPGHSP.[L]	1175.58036
1778	[L].SRLGYQGTFF.[P]	1175.58438
1779	[S].FTESKSPTAGPG.[N]	1178.56879
1780	[R].TGQPGAVGPAGIR.[G]	1180.6433
1781	[P].GPASGTEPAHFL.[R]	1183.57421
1782	[G].PGQGGPRPPGQH.[Y]	1184.59193
1783	[N].PSGSVVMVSGLH.[Q]	1185.59323
1784	[E].AAAAEPYGARGPG.[S]	1187.58036
1785	[R].AHQTPQQQGPP.[I]	1188.57561
1786	[-].MSGGGRPQPGAAYA.[L]	1188.57561
1787	[G].SRYSGAHIGSGP.[S]	1188.57561
1788	[G].GPGAPPTSSAYAL.[P]	1188.58953
1789	[I].GLGEGAGPSPFLS.[G]	1188.58953
1790	[S].LLLLCLPSFL.[V]	1188.70608
1791	[G].PPGESVVGAPGAPG.[T]	1191.60043
1792	[P].GPLGAGAAGAPAGGAP.[E]	1191.61166
1793	[G].ERGPSGLPGPAGP.[P]	1191.61166
1794	[D].FGFDGDFYRA.[D]	1194.52145
1795	[P].AAAAPLSQDGPQA.[E]	1196.59059
1796	[S].PQNGGGLSIAEPG.[G]	1196.59059
1797	[G].TFWTVGSLSAE.[G]	1197.57863
1798	[A].ASYGLGSDLYR.[E]	1201.58478
1799	[K].PQSTTSLSNGLP.[S]	1201.60591

1800	[M].GPGPSAEAPRAGH.[A]	1203.58651
1801	[-].MGPGPSAEAPRAGH.[A]	1203.58651
1802	[Y].YPPGPYPPGPY.[A]	1204.56734
1803	[A].PGAGPPREAGSGPG.[R]	1206.58617
1804	[R].GPAGAGPGPGPGSGTA.[P]	1207.57019
1805	[K].AVGHLDDLPGTL.[S]	1207.63173
1806	[P].STPQEGPATPHS.[N]	1208.55421
1807	[Q].PSTQENLPPPE.[A]	1208.57936
1808	[K].PEAATSGQALAPP.[A]	1209.61099
1809	[S].VGEAGLPWNLGP.[L]	1209.62625
1810	[V].PGSPGLPGSRSAE.[R]	1211.60149
1811	[N].QPNGQSAVGTGAQ.[Q]	1214.576
1812	[L].GPAGNAASTAGPFP.[F]	1214.58003
1813	[P].GAATFGSQPGPPQ.[P]	1214.58003
1814	[K].PGLDTHGMRHP.[T]	1217.5844
1815	[A].DKGGGISKSQED.[P]	1220.57534
1816	[E].NPPEPVSTGVSH.[Y]	1220.59059
1817	[E].AANYQDTIGRL.[Q]	1221.62223
1818	[G].EVGADCPQGYK.[R]	1223.53611
1819	[G].PSGPQGPLGYPGP.[R]	1223.60551
1820	[M].TKLPSGLPVSLL.[T]	1224.7562
1821	[S].KESATGQPFFN.[L]	1225.58478
1822	[P].KGEMGPVGPAGNP.[G]	1226.5834
1823	[A].PNATAAAAAAWTN.[I]	1229.59093
1824	[F].PGSPGEKGEKGST.[G]	1230.59607
1825	[Q].GPQNKQPFMVA.[F]	1232.60922
1826	[L].GSPGPGLQGSAPNP.[R]	1235.60149
1827	[Y].DFGFDGDFYR.[A]	1238.51128
1828	[I].DLWGAGCIMAE.[M]	1238.51802
1829	[E].PGSTATWPLDPP.[K]	1238.60518
1830	[A].PGAGGGSGSVPGPSR.[I]	1239.60764
1831	[L].LHPAAFHGAPSH.[L]	1241.61742
1832	[V].PGAGTADALGPGMI.[F]	1243.59871
1833	[K].GPQGPGQPSGPPPA.[K]	1243.60658

1834	[T].PGAGGAGRARGSSF.[A]	1247.62396
1835	[Y].AREPGGPEQATH.[F]	1249.59199
1836	[P].AQPSEAPALAAEP.[A]	1251.62156
1837	[S].CLAALSQPPPNS.[P]	1254.6147
1838	[V].GPAGNPGAKGERGS.[S]	1254.61854
1839	[E].ERAGRGASEGPPA.[P]	1254.61854
1840	[R].VGQGYVFEAPPP.[E]	1260.62591
1841	[R].LGDGAQGAHLPGPA.[P]	1260.63312
1842	[G].QDGLPGLPGPKGE.[P]	1264.65319
1843	[V].PRGCLSPRAGPP.[A]	1264.6579
1844	[C].DQINKELEGPQ.[I]	1270.62737
1845	[Y].DLSFLPQPPQE.[K]	1270.63139
1846	[I].QVGEQSTVQEPA.[T]	1272.60664
1847	[P].GPAGERGHPGSPGP.[A]	1272.60797
1848	[V].GPATSLTARTDTS.[V]	1277.63318
1849	[P].GPGPSPGAMLGPSPG.[P]	1278.6147
1850	[D].TASLQSHNGSPLA.[S]	1282.6386
1851	[Q].RGPAGAGPGPGPGSGT.[A]	1292.63419
1852	[G].TAGGAGAGPAGAGLQH.[S]	1292.63419
1853	[A].AGREGAQPGAQPGP.[E]	1292.63419
1854	[L].GPATPTVSSPQPPG.[D]	1292.64811
1855	[F].LENVIRDAVTY.[T]	1292.68449
1856	[Q].STFPQQVGQFTG.[S]	1296.62189
1857	[T].VSDCKVVNATGF.[C]	1296.62526
1858	[E].FVFDVWGAIGDA.[K]	1296.62591
1859	[H].AEHCQLLTTPH.[V]	1306.62085
1860	[P].QPVESEAGPAAPGP.[A]	1306.62737
1861	[P].GPPSGGYDLSFLP.[Q]	1306.63139
1862	[Y].DFGFDGDFYRA.[D]	1309.54839
1863	[S].PQNGGGLSIAEPGGG.[A]	1310.63352
1864	[Y].PGAATFGSQPGPPQ.[P]	1311.63279
1865	[T].ATAPEPTSPDAIAA.[S]	1311.64269
1866	[P].PVGDTLGGPGGTLGN.[A]	1311.65392
1867	[E].WSRGGRCGPGPGA.[A]	1314.61201

1868	[A].GPRGPAGPSGPAGKD.[G]	1320.66549
1869	[Q].AIELEDLLRYS.[K]	1321.69981
1870	[G].PGATSHNPHQPAN.[L]	1327.61379
1871	[S].NIPDEYFKRF.[S]	1328.66336
1872	[A].AGEQTKTFSLSY.[T]	1331.64777
1873	[S].SPASAQVSPQHQP.[H]	1333.6495
1874	[G].PGAGSASRDPGPPAP.[L]	1333.6495
1875	[G].PGAGQNKPALMAY.[L]	1333.6569
1876	[E].KSIDDLEDELY.[A]	1339.62637
1877	[L].VGLERVANLEQL.[M]	1340.75324
1878	[P].GPAGERGHPGSPGPA.[G]	1343.64509
1879	[S].FPENPLGQYQR.[Q]	1348.66443
1880	[E].AVPKDLPPDTALL.[D]	1349.76749
1881	[M].APTAEERTTIHE.[M]	1354.65973
1882	[-].MAPTAEERTTIHE.[M]	1354.65973
1883	[G].PGANGMPLAGLAWS.[S]	1357.6569
1884	[T].TIPQGLPPSLTEL.[H]	1365.76241
1885	[A].VAGNGGAGAAVGEPPGP.[E]	1377.67572
1886	[M].KGEAAAAGGPTADPAP.[T]	1380.67538
1887	[L].GPSTPRSATSHSIS.[E]	1384.68153
1888	[P].ARGSQPAEPGASEK.[E]	1384.68153
1889	[P].PQPTAQSAQPAPHG.[L]	1386.67605
1890	[E].GPLCPVGPGPGGPGPA.[G]	1386.68345
1891	[E].TAAPTANGSIGDPSK.[E]	1386.68595
1892	[T].ITSREIQTAVRL.[L]	1386.80634
1893	[F].PQGAAPGPFAWPHG.[A]	1389.66984
1894	[D].GRTGQPGAVGPAGIR.[G]	1393.76587
1895	[R].RAGGGGGGGVQNGPPAS.[P]	1395.67236
1896	[M].GTGAPQPNLMPSNP.[D]	1396.65254
1897	[L].GPSAPAFPGPYGAAAA.[P]	1401.67974
1898	[M].APGSPSRGGTGDMAE.[V]	1405.60123
1899	[K].SPDLPNATSAPPAAP.[P]	1405.69578
1900	[A].IGDGANDVSMIKGI.[I]	1405.69916
1901	[D].PGSSLSPTGGAEDHP.[L]	1408.63391

1902	[Q].GRGEVGAGAGPGAQAGP.[S]	1408.69277
1903	[S].GPGGGARSGGGRPAAAN.[A]	1409.69925
1904	[P].VDGLAGSAAGPGAESR.[A]	1414.6921
1905	[S].DKISNGSSINWPP.[E]	1414.69612
1906	[P].GLQAAEAASPSPMKG.[R]	1414.69949
1907	[E].PGWQSLGGSVFPSP.[E]	1415.69539
1908	[D].PGAETKAGNGTVDIS.[G]	1416.69651
1909	[P].PGSAPGPGPLSGSQGPG.[Q]	1419.68628
1910	[S].GAPVSSPGSPPPWPS.[P]	1419.69031
1911	[L].PGSPPSTPSPPGTLQ.[L]	1419.71143
1912	[S].LTGPSPDAAAAPAPTS.[G]	1423.70635
1913	[A].GPLETASGPPALGGNS.[N]	1424.7016
1914	[W].PGPSGGWLSQGQASP.[D]	1425.67572
1915	[L].DMLQRAAGGAGQGPP.[P]	1425.69032
1916	[G].NASGLGAGPGPSVGMGV.[V]	1427.69474
1917	[A].QAQAQAQAQAQASQ.[A]	1428.68259
1918	[G].AGPATTTTPPGPPAGH.[T]	1429.70702
1919	[G].YDLSFLPQPPQE.[K]	1433.69472
1920	[P].PGLDGLPGTSGLPGPV.[G]	1433.76347
1921	[L].QGTRQGPEPGLSGGP.[A]	1437.70808
1922	[A].AGRQSAGPQPSPEGV.[T]	1437.70808
1923	[K].KEQGAQQAFVFGQ.[N]	1437.7121
1924	[R].RADAGSPPAAADGRQ.[P]	1439.69858
1925	[P].PSPGLDSGSSSALAPP.[E]	1439.70126
1926	[R].QSTVSEKGTSALMT.[V]	1439.70463
1927	[P].LWGQGQGPSWGPVA.[W]	1439.70662
1928	[A].RYPWPSAAPSGPPG.[G]	1439.70662
1929	[Q].EKETAAAAGAVGPPGN.[W]	1439.7125
1930	[P].GPSPLSATQGATPQQ.[P]	1439.7125
1931	[S].SGPPGPPGPKGDQGPP.[G]	1441.70702
1932	[M].GPRGDATGKSQKKL.[Q]	1442.8074
1933	[L].PGSPGAKGEQGPAGHP.[G]	1443.69752
1934	[Q].GPRAGPEGAGGGPGLPP.[A]	1443.7339
1935	[F].QTASPAPGVYPKCA.[S]	1446.70458

1936	[P].GPSVSAPSVSTSSSSI.[G]	1449.70674
1937	[T].GGRPGSGAAASAVPSAH.[R]	1449.71931
1938	[A].VGPRGPSGPQGIRGD.[K]	1449.7557
1939	[A].EPAAAATTGPPAEATP.[G]	1451.70126
1940	[T].AATAAASPSPPMAPVP.[A]	1451.71989
1941	[E].VGAGAGPGAQAGPSAKR.[A]	1451.77135
1942	[S].WLSTFTSSTPQSL.[T]	1454.71619
1943	[H].SNPAKGSAHGACDSP.[P]	1455.62812
1944	[N].PGGQLGAGSGGAYHAR.[H]	1455.70875
1945	[A].AAIGWMPVASGPMPA.[P]	1455.7123
1946	[A].EAVAANPGAMLELGP.[P]	1455.71481
1947	[G].DPGVGVQGPPGPAGPPG.[L]	1455.72267
1948	[F].SGIGRSGQQPQPLM.[S]	1455.73727
1949	[P].GPAAAANATPAEEGET.[K]	1456.65504
1950	[S].SAVTTTSAHQGVTTP.[P]	1457.72306
1951	[E].AASPTAALMNGVRAQ.[L]	1457.75292
1952	[S].PNPMLATAAPAAPVH.[A]	1457.75695
1953	[G].GYDFGFDGDFYR.[A]	1458.59607
1954	[I].QQPQTAVTAGQTQT.[Q]	1458.71831
1955	[A].GPATTTTPPGPPAGHT.[L]	1459.71758
1956	[D].GSPGPAPAPTPGPGSGR.[R]	1459.72882
1957	[P].GPSPGPGPSPGAMLGPS.[P]	1462.69949
1958	[P].GPSPGAMLGPSPGPSPG.[S]	1462.69949
1959	[A].QAPAATPETSVASPH.[S]	1463.7125
1960	[A].QLSGGQGAAEPPQPQ.[P]	1464.70775
1961	[S].PGAGSPHLSQGSSGVAG.[G]	1465.703
1962	[S].GPRSSSSAPPANPPSG.[L]	1465.703
1963	[T].HTTTATPTGPHTPF.[T]	1465.70702
1964	[P].VMPPQTQSPGQPAQ.[P]	1465.71039
1965	[A].PGRQEGPAGAAGAQAGA.[R]	1465.71423
1966	[E].GPTRPSGGGGARSAHT.[V]	1465.72546
1967	[E].AAAAAQPAGSPGETPAV.[A]	1465.72815
1968	[A].GSPGLPGPVGPAGAKGM.[P]	1465.74677
1969	[A].EAAAAAAAAAAPGIRL.[E]	1465.81215

1970	[E].APGAAAPGAGPGPAEEM.[E]	1466.65802
1971	[K].TPASGPQTPTSTPAPG.[S]	1466.71216
1972	[E].AGPQAPPPPGTPSRH.[E]	1466.74989
1973	[K].KNSGSEVAPLEEHA.[S]	1467.70741
1974	[L].AVSQEPAQTSDVHV.[Y]	1467.70741
1975	[E].KHFFTSFGARDR.[C]	1468.74441
1976	[V].PGSPTVNPSSIGNKD.[P]	1469.72306
1977	[A].EKQNQAAGPPPPNQ.[A]	1475.72373
1978	[E].AGSTGPSRPGSPGPPGP.[L]	1475.72373
1979	[S].AAQPAPFPSTHSAPP.[P]	1475.72775
1980	[G].AASATRGGGAQSQRGT.[P]	1475.73094
1981	[S].GPAATPGQPGICPGVP.[D]	1475.73112
1982	[D].RQTDPPPTPPPLC.[S]	1475.73112
1983	[V].STPPTGLGGGSASGKTT.[V]	1475.73363
1984	[S].PGSVGPESLPLEFF.[T]	1475.74167
1985	[L].ATAHAPQASGTQPQL.[R]	1477.73938
1986	[R].PGPSGSFVTPGLHPQ.[S]	1477.7434
1987	[V].VAAQERNPGAPRPD.[T]	1477.75061
1988	[P].GPSPGAMLGPSPGPSPG.[S]	1478.6944
1989	[P].GPGPSPGAMLGPSPGPS.[P]	1478.6944
1990	[P].PGSAVSGPSVGQGPPDA.[V]	1479.70741
1991	[S].SFVFGTGPSAPSASPA.[F]	1479.71143
1992	[P].GEPGRPGPPGPPGPGPG.[G]	1479.7339
1993	[G].SPAPAAGASGQASELAP.[S]	1481.72306
1994	[V].GPGATPPGAPARPAPAP.[R]	1481.78594
1995	[A].ATAATTAATATATATST.[E]	1482.72821
1996	[A].PSGRGEPGPPSEAVF.[A]	1483.71758
1997	[T].GPSPLDSAPPGGTPHP.[L]	1483.71758
1998	[G].GPAGFPARGGNESLPG.[S]	1483.72882
1999	[W].PGSPQVSGPSPATRM.[P]	1484.7162
2000	[S].GPRGQGTASPGSVSDL.[A]	1485.72921
2001	[A].GAGPATTTTPPGPPAGH.[T]	1486.72848
2002	[C].PGADPGRTPRHPET.[P]	1487.73496
2003	[N].SGQLEPGPAGAPSPAPG.[L]	1489.72815

2004	[M].AAAAAGAGAGAAQEKQF.[P]	1489.73938
2005	[-].MAAAAAGAGAGAAQEKQF.[P]	1489.73938
2006	[P].GPAPAPTPGPGSGRRD.[S]	1489.75061
2007	[E].IDYRPHGGAGDADF.[Y]	1490.66588
2008	[G].GYDLSFLPQPPQE.[K]	1490.71619
2009	[L].LHFSQTGAPGHPEL.[Q]	1490.73865
2010	[G].PPSGGASPTPPAASPSGG.[S]	1491.70741
2011	[D].SGHATISTAITASSST.[S]	1491.72854
2012	[G].PGSPEGNTPLDPPSQ.[Q]	1492.69143
2013	[L].GPGAPERGSGLDPWP.[A]	1492.71792
2014	[G].GSAAAAAAAAASGGSSDNS.[I]	1493.64627
2015	[P].AGPAAPSSAPASSSPAAP.[A]	1493.72306
2016	[P].GSGGGAGTGAGAGGPGTGHL.[P]	1495.68841
2017	[Q].QNLQMQPPSMPPQ.[P]	1495.7032
2018	[S].GEAQLSPQAGRMNH.[H]	1495.70703
2019	[P].SGPRSSSSAPPANPPS.[G]	1495.71356
2020	[L].PGPSTQVTAGSNHTAA.[L]	1495.71356
2021	[Q].ERQQQAPGGPGGVTGG.[K]	1495.72479
2022	[E].AASAPSPTAPEPSVDV.[E]	1495.72748
2023	[L].PGAMKNGQAAGPAPQT.[P]	1495.73219
2024	[D].TEVPGATAHSPLSTQ.[L]	1495.73871
2025	[L].RQGKGLTAASAGPPGGA.[F]	1495.79756
2026	[R].FEGPIGQAGGGGFRF.[E]	1496.72809
2027	[P].GPATSPGPARLSEEQ.[R]	1496.73396
2028	[K].ERGAGVSGGPSADPLAG.[L]	1497.72921
2029	[A].QKGSTSAAPQEKHE.[S]	1497.72921
2030	[C].GPQGLGSPGTSAAMLGP.[L]	1497.7366
2031	[I].QELGFGVAPGFQTF.[V]	1497.73726
2032	[Y].TAPIMSGQGLAGFFT.[S]	1497.74063
2033	[F].PGSPGEKGEKGSTGIP.[G]	1497.75436
2034	[N].NGNKGPPAGSRISMP.[T]	1498.74309
2035	[D].GVEQRGPASGPSLPF.[V]	1498.76487
2036	[M].GGPQYSQQQAPPNQ.[T]	1499.68735
2037	[P].GPALDSSLGPTDGAGAN.[S]	1499.69724

2038	[S].APAAAGSGQATAQPKSS.[T]	1499.74486
2039	[A].PGSPLGHSPTASPPPT.[A]	1499.74888
2040	[A].APNATAAAAAAWTNIS.[L]	1500.74413
2041	[L].APAGPAPYSPPGPGPAP.[P]	1500.74815
2042	[Y].PGNAGPVGTAGAPGPQGP.[V]	1501.73938
2043	[E].NPPSWAKSAGAKSSGG.[R]	1501.73938
2044	[A].PGAAPASGPAASKFLC.[L]	1501.74677
2045	[G].PSGPGGKGSPSEMKST.[T]	1503.71078
2046	[S].PSTTPPHPYQQGPP.[S]	1503.72267
2047	[G].AAPQFHAAEAGGLGAH.[L]	1504.72915
2048	[S].QSPQVTSPPTQTPH.[S]	1504.73905
2049	[P].PSGPRSSSSAPPANPP.[S]	1505.7343
2050	[S].GAPGVPSGNGAPGPKGEG.[E]	1505.7343
2051	[A].AVQQEAPSAQSPGVH.[T]	1505.7343
2052	[P].GPRGEPGAAGIPGEPGS.[P]	1505.7343
2053	[G].QAHLASSPPSSQAPGA.[L]	1505.7343
2054	[A].GPKGETGPQGYKGMV.[G]	1505.74169
2055	[G].PKGETGPQGYKGMVG.[S]	1505.74169
2056	[S].LGYHPYAAPLGSYP.[Y]	1505.74234
2057	[P].AAASSPATAPAPAPAPAS.[A]	1505.75945
2058	[T].GSPGPKGSPGFPGIPGP.[P]	1505.7747
2059	[N].GSPSRAACLVPAFSS.[S]	1506.73694
2060	[T].SPSLTGPSPDAAAAPAP.[T]	1506.74346
2061	[A].ATAAPGQTPASAPAPAQ.[T]	1506.7547
2062	[G].TPTQQPSTPQTPQP.[P]	1507.73871
2063	[S].PQHQVQPSPLGHPS.[S]	1508.76045
2064	[G].APGAGAASPAEPEDGVD.[A]	1510.66561
2065	[A].GLPGSDGAPGPPGTSLM.[L]	1510.72062
2066	[P].GPSHPPPNPASPRAE.[A]	1510.73972
2067	[P].SAPVFNSPGHSLNNA.[R]	1511.72373
2068	[E].PVDGLAGSAAGPGAESR.[A]	1511.74486
2069	[P].TGPKGEPGFTGRPGGP.[G]	1511.76012
2070	[T].AAAAEPSSDVEVETH.[R]	1512.68126
2071	[Y].TPQSPTYTPSSPSY.[S]	1512.68528

2072	[R].GSPTVASGTGPGSHTLS.[P]	1512.72888
2073	[S].SADPTALGGPAGAEGPM.[A]	1514.67915
2074	[G].PGGSSGGAGGGRVLECP.[S]	1514.70162
2075	[A].QGVAGSAGAPGATLMVQ.[L]	1514.76315
2076	[G].GGYDFGFDGDFYR.[A]	1515.61753
2077	[V].TQGPQQPQPPSQQP.[L]	1517.7343
2078	[C].REVGGGASAASSMLPQ.[S]	1517.73767
2079	[G].GVVGAGMAAAALAAEAGM.[V]	1517.74506
2080	[V].GPQLAGGGGSGAPGEPPL.[L]	1518.7547
2081	[P].QPPGSAVSGPSVGQGPP.[D]	1518.7547
2082	[T].QPNGGLSHTGTPKPAG.[A]	1518.76593
2083	[M].PPMPGPGPGPGPGPGPGP.[G]	1519.73621
2084	[K].FGGAPAGPAGTGKTETT.[K]	1519.73871
2085	[P].AASTPAGPPSGGASPTPP.[A]	1519.73871
2086	[G].SGPGPGVSAAPGPAAAANA.[T]	1519.74995
2087	[T].MIYTVTGGLAALMY.[T]	1519.7535
2088	[H].RGQQPAASTAGGQPPP.[P]	1519.76118
2089	[L].APSVGQGGSATASCSPS.[P]	1520.66456
2090	[A].YFTLGAGGPGWEPAV.[E]	1521.73726
2091	[P].PGAEGNRTAGPPQRN.[E]	1521.75168
2092	[P].PGKMGPQGTPGIPGMP.[G]	1521.75523
2093	[L].PGAGADLAVDPDQPLS.[A]	1522.73838
2094	[L].PGASPATGVTNPQGTAP.[P]	1522.74961
2095	[P].SPGPGPVPPRPPAAEP.[P]	1522.80125
2096	[G].SQGPPGKMGPPGNIGN.[P]	1523.7271
2097	[K].GPPPFPGAPLMSSPVG.[G]	1523.75628
2098	[A].PGTSGLQQVAFHPGQ.[K]	1523.76012
2099	[D].RGAATEGGPGGPSRASP.[L]	1524.75134
2100	[L].SGPGLAPAASSAGGAAPSV.[Q]	1524.76526
2101	[V].PGSHSCFGGSILCF.[C]	1525.65625
2102	[S].GLPSGPSAGSGPPPPPPG.[P]	1525.76453
2103	[P].PGSPGAQPALARGQYG.[F]	1526.77102
2104	[T].AVAGGGHGSHRAGAPEP.[P]	1527.74111
2105	[L].PSGAPATGPSVTNPFQ.[P]	1527.7438

2106	[A].GAPGPQGPVGPTGKHGN.[R]	1527.76626
2107	[F].KNMVPQQALVVRE.[G]	1527.83117
2108	[I].ALWESGVSAAGGGPAGAA.[G]	1528.73905
2109	[G].GYDFGFDGDFYRA.[D]	1529.63318
2110	[A].QPGSVAGAGPGPTEGFT.[E]	1529.72306
2111	[A].GSPATAAGPATATEEAK.[G]	1529.74419
2112	[E].QHVQQPSAQQPGQP.[E]	1529.74553
2113	[N].GPTGPPGSPGPRGNAGGP.[G]	1529.74553
2114	[M].QGPPGPRENQGPAPQ.[G]	1529.74553
2115	[A].AASAVPGSGAAAGALASGGS.[K]	1529.75542
2116	[P].GAPAASPFAAATALGGAE.[A]	1529.75945
2117	[F].GSPATPPPPSPPSFPP.[H]	1529.76347
2118	[A].GAAGGAAGGGPAAGPADHGL.[A]	1531.72479
2119	[F].PNSGLQAGSASLEGFP.[Q]	1531.73871
2120	[P].GLPGDPPSASLPTADH.[R]	1531.73871
2121	[A].PSGSTRPSAASSRPSS.[R]	1531.74592
2122	[P].GAKGEQGPAGHPGEAGL.[P]	1532.74519
2123	[Q].GPSPPLNGHILSCSP.[D]	1532.75259
2124	[P].TQPQVGADGLYSSLP.[N]	1532.75911
2125	[L].GAPPYPASAPGASPRH.[R]	1532.76045
2126	[A].SAFAMLSLGARGNTH.[T]	1532.76382
2127	[R].TKDPPTGSPPASPGPQ.[S]	1533.75436
2128	[T].PGATQYVPGPGQPPAP.[S]	1533.76962
2129	[R].GPGGGRAAAEAQGQPLP.[L]	1533.77683
2130	[A].PGSGIAEYLFDKHT.[V]	1534.75363
2131	[L].PQGVVMAASPGSLHSP.[Q]	1534.76824
2132	[F].SVAITPDHLEPRLS.[I]	1534.82238
2133	[A].VQQEAPSAQSPGVHT.[D]	1535.74486
2134	[G].NLGAGNGNLQGPRHM.[Q]	1535.74957
2135	[K].GPALWEAGSPVAFYA.[S]	1535.75291
2136	[E].RGGAAQASEGVRETF.[R]	1535.75609
2137	[G].QARNGAGGGPRGQTPN.[H]	1537.75782
2138	[P].GPSTGALQERSPGSPP.[G]	1537.76051
2139	[A].SRGGQAGPGAGAPTLAGL.[S]	1537.80813

2140	[Q].AAAGTQPGPAAAGEPSVS.[E]	1538.74453
2141	[A].LAQPDSTAPGGATGQAP.[P]	1538.74453
2142	[T].PAGGRSSQQPTTPQQ.[V]	1539.75101
2143	[M].GPSGEARGAPALPCTV.[S]	1539.7584
2144	[A].AAAAPSPAPARDPGPGH.[L]	1539.76626
2145	[A].APGAAGASVGAAAAAATPGT.[E]	1539.77616
2146	[H].GPQGVPGGAGGHDGSGHP.[A]	1540.68874
2147	[F].PGGLGDAAFFAVNGFT.[V]	1540.74307
2148	[Q].GSPQPLGSGGPGAPPHQ.[L]	1540.75028
2149	[N].PQSQNLGPSPQRMT.[P]	1540.75365
2150	[E].GPLCPVGPGPGGPGPAGP.[M]	1540.75767
2151	[A].VNPSGSPAGPAGAAAACS.[P]	1541.70128
2152	[G].LNLCTGTGSKAWSF.[N]	1541.74169
2153	[P].PGSPGPRGNAGGPGLPGP.[P]	1541.78191
2154	[G].QPGPPPGAGAPPTERN.[E]	1542.76593
2155	[R].SSLHAPFSPNSESLA.[S]	1543.73871
2156	[M].GPPFAEPPTAEMGVK.[G]	1543.74611
2157	[P].GTSITTSAQSESIVY.[T]	1543.74861
2158	[R].PPAYGPPGPGGFGLPY.[E]	1543.75799
2159	[-].MAGLGKMGPGQCLSPL.[I]	1543.76071
2160	[P].PGAGMLGFPPSATSSPA.[L]	1544.74135
2161	[Y].GAEHTAVAPTSSTKST.[S]	1544.75509
2162	[P].PVWDVSASAKGVSMP.[P]	1546.75701
2163	[Q].QPATTTATSTAAAAPST.[A]	1547.75476
2164	[T].GPGPSPGPAPPNYSRP.[H]	1547.76012
2165	[G].GPPSGTRGPGASVHDR.[N]	1547.76733
2166	[R].KSPPTGSTASLCVSR.[V]	1547.78462
2167	[P].QGPGGFLGPPGPQGPKG.[Q]	1547.7965
2168	[A].AAQPSTPAGTPRSGGGH.[S]	1548.75134
2169	[M].EIPEAGSAGNISIYE.[R]	1549.73804
2170	[S].PGKGGGGGAGTAPEKPDP.[A]	1549.76051
2171	[I].PGRASPGAAGRDVGWP.[E]	1550.78225
2172	[A].AAAAASAASAVGPVHNSV.[P]	1550.79214
2173	[L].GPAGGDEEVQAPSSPPG.[E]	1551.69216

2174	[G].VSAAGGGPAGAAGGAAGGGPAA.[G]	1551.75101
2175	[D].GEAPARGGGADPQQLAG.[L]	1551.75101
2176	[F].QEDLISSAVAELNY.[G]	1551.75369
2177	[G].PGQSGGLGGVGAGPAPSW.[W]	1551.75503
2178	[P].GRCRTPAGEGPHTR.[V]	1551.75572
2179	[V].LPWGWGAGPSAPTGTP.[P]	1551.75905
2180	[A].GSPGTVPAGSATATPSPP.[R]	1551.76493
2181	[S].PQISYHWSYLPY.[S]	1553.74234
2182	[Q].QPSASQAGVQQPPATS.[T]	1553.75542
2183	[N].AATAATTAATATATATST.[E]	1553.76532
2184	[M].GPSPGPPSAGHPIPTQG.[P]	1553.77068
2185	[S].MSPPRPGAPAALTDTG.[A]	1554.75807
2186	[H].KFMAFKSFADLPH.[R]	1554.77735
2187	[A].GPVGGGGGRPALPAPHSA.[E]	1554.81355
2188	[G].GPGPHPASAPAATQEAP.[S]	1555.74995
2189	[A].QRGGPGGGPHPHSPAF.[R]	1555.75128
2190	[D].RSTTTSPASGRPSNH.[A]	1555.75716
2191	[V].AAGGQVPHPGQAPPGGAS.[S]	1555.76118
2192	[F].PGERSGAHPPSPAAGAP.[T]	1555.76118
2193	[L].GPSAGRSEVSADLPGGV.[S]	1555.77107
2194	[L].NLGGAPEGPAGTGKTET.[T]	1556.75509
2195	[G].GPVGAGHGNPPGGGGSGPK.[A]	1556.75643
2196	[L].AAASAAPQPQPPPQPE.[P]	1556.77035
2197	[E].ALERMFLSFPTTK.[T]	1556.81413
2198	[E].QEAAAASGAVGVGPSQAS.[P]	1557.75034
2199	[G].FAQGPGASASTAATPGPA.[G]	1558.74961
2200	[G].GPGSPDLARHYKSSS.[P]	1558.76084
2201	[A].KGEQGPAGHPGEAGLPG.[P]	1558.76084
2202	[P].GPSPGPGPSPGAMLGPSP.[G]	1559.75225
2203	[G].PGPSPGAMLGPSPGPSPG.[S]	1559.75225
2204	[P].GPGPSPGAMLGPSPGPSP.[G]	1559.75225
2205	[L].QRQQGQAAGGRGPHGG.[P]	1561.76906
2206	[A].PAGADKNVNRSQSFA.[V]	1561.77174
2207	[P].GPQRPDQPATAAAAGPG.[D]	1561.77174

2208	[Q].KDYSRGFGGKYGID.[K]	1562.75978
2209	[G].SGPAATPGQPGICPGVP.[D]	1562.76315
2210	[P].VMPPQTQSPGQPAQP.[A]	1562.76315
2211	[R].PGSPSTVPLAPSEAVPG.[P]	1562.80606
2212	[D].KENQQAANGPNQPPA.[R]	1563.75101
2213	[I].TSSARSPSASPTARGM.[V]	1563.75438
2214	[L].GPGAPERGSGLDPWPA.[A]	1563.75503
2215	[P].QPANPPHGAHPLSSGP.[Q]	1563.76626
2216	[L].PGAAVSASQAPGFVSFA.[G]	1563.78018
2217	[E].PLGEATGSGPAATPGQPG.[I]	1564.76018
2218	[V].PGTARGAEGGSTAPAPAP.[A]	1564.77141
2219	[P].QGSLGPPNGLPADTLQ.[G]	1564.79656
2220	[A].SPSRGPGTAGTLHLVD.[L]	1564.80779
2221	[L].QGTRQGPEPGLSGGPAG.[G]	1565.76666
2222	[W].PGSPQVSGPSPATRMP.[G]	1565.77405
2223	[D].LTGEVSNGAVAMAKTT.[L]	1565.78395
2224	[V].QAMKPPGAQGSQSTY.[T]	1566.72168
2225	[K].KGGGAAGGGGVASGGAGGPQP.[P]	1566.76191
2226	[T].SSPRAMPPSPGPTER.[H]	1566.7693
2227	[L].GRAGQSGAGNNWAKGH.[Y]	1567.74726
2228	[H].PGPFPSSAGPGPHYLS.[S]	1567.75397
2229	[V].AAAEPAAAATTGPPAEAT.[P]	1567.75984
2230	[M].GARYPWPSAAPSGPPG.[G]	1567.7652
2231	[D].VGEERRGGGGTELGPP.[A]	1567.78231
2232	[H].EDLVVSASPELSPPQ.[P]	1567.78499
2233	[G].GSGAAAATAATAGGQHRN.[V]	1568.75241
2234	[P].ANRGSAQGPSKACAPP.[W]	1568.7598
2235	[M].GAAWPPGGHPGLSDHL.[W]	1568.76045
2236	[R].GAGAGGRSAGGGPPDSSLV.[T]	1569.76157
2237	[L].SGHAVPFASPSMVPGL.[D]	1569.77299
2238	[E].GHYAIPGAPARIEAM.[P]	1569.78422
2239	[H].RQQQEAPAGGGCSPQ.[S]	1570.70268
2240	[E].QVGGERSGGPEWSAPG.[R]	1570.72446
2241	[G].YVHQQAPTYGHGLT.[S]	1571.76012

2242	[V].LAASEGAQAAGGSSQPLG.[A]	1571.76599
2243	[P].SGPGPVTQGPQQPQPP.[S]	1571.78125
2244	[S].PGAGSRAENVCKVLN.[H]	1571.79585
2245	[L].GTIQQVISERVTQL.[S]	1571.87515
2246	[S].GGGYDFGFDGDFYR.[A]	1572.639
2247	[V].PVGDSVASAATENQRA.[W]	1572.76124
2248	[A].APGAAPASGPAASKFLC.[L]	1572.78389
2249	[T].APASSSFAAAGLGVACH.[L]	1573.74275
2250	[P].QGPPGTPGTPGTTRYS.[R]	1573.76051
2251	[S].PGGSPVSPSTSPLYDL.[S]	1573.77443
2252	[G].AAGAGAAPALDFTVENV.[E]	1573.78566
2253	[G].PQGPKGDKGDPGVPGAP.[G]	1573.7969
2254	[P].GPAPGFAAGQQTPATGF.[R]	1574.75978
2255	[G].PAPYSPPGPGPAPPAAM.[A]	1574.76718
2256	[G].GPGAGGGPVTPAEPRVR.[E]	1574.83976
2257	[G].NGLSTPPGPGGGPHPPH.[T]	1575.76626
2258	[P].FASTFVGGELYTGLN.[A]	1575.76895
2259	[S].GPRGPQTEAPPEGVPS.[P]	1575.77616
2260	[H].QPTANPGLGGPYLYQ.[W]	1575.78018
2261	[I].QPGAGQAGVVQPGAGQPG.[L]	1575.78739
2262	[L].GARRKPQGAGPGQAAPG.[G]	1575.84625
2263	[L].AAPAAEAASAAPDPPAAGA.[A]	1576.76018
2264	[C].SRDLAGSGGAAGMDLSL.[G]	1577.7588
2265	[A].GPNRPEAPSMLSPGPA.[L]	1577.77405
2266	[A].GAVGPRGPSGPQGIRGD.[K]	1577.81428
2267	[I].QAAAGTQPGPAAAGEPSV.[S]	1579.77107
2268	[G].PPSSQVSGGAIDSQLH.[P]	1579.77107
2269	[Q].QQAQQQVAQPAPASQ.[Q]	1579.78231
2270	[S].TGIPGMPGSPGPKGSPGS.[V]	1580.77372
2271	[L].GPQPGLSGGSAGVGPELT.[L]	1580.79148
2272	[K].QLGGGTLTGAALDFML.[P]	1580.79887
2273	[E].PGRREGSGGGGGNRPGL.[G]	1580.80002
2274	[V].KNGHNLFMAAAAAPPA.[G]	1580.80021
2275	[R].SPGRSAGASPTNPGPTQ.[S]	1581.76157

2276	[S].RGATNQGATEPTEHI.[E]	1581.76157
2277	[D].TQAPSAAPQAPHDPPP.[V]	1581.7656
2278	[W].PGSPQVSGPSPATRMP.[G]	1581.76897
2279	[A].EPGPPGPPGPPGPMGLQ.[G]	1581.77299
2280	[T].KGDPAGAGPETSLEPGV.[D]	1581.77549
2281	[L].QISNQPTSPTILVAL.[R]	1581.88465
2282	[G].GPAPSAVSSAQALLRSA.[G]	1582.85474
2283	[K].QELSEPVASGGKPRK.[G]	1582.85474
2284	[S].SPGQTPQSPSLLSKR.[K]	1582.85474
2285	[R].KHNFPTSSPTLLIK.[S]	1582.89515
2286	[P].GPSLAETGSGTGDLAPPG.[T]	1583.75476
2287	[P].GVGGSGRSPGSARSAGSH.[T]	1583.7633
2288	[I].PAKAMEAAQAHKMAP.[D]	1583.76686
2289	[Y].AAQQHPQAAASYRGQ.[P]	1583.76733
2290	[L].GAPGGSPPAQPPSTYSL.[P]	1583.77001
2291	[G].REGTAGRGGSRAGSQH.[L]	1583.77454
2292	[G].PQGPPGTPGTPGTTRY.[S]	1583.78125
2293	[S].PQAQPQPGHRIDSPG.[F]	1584.78773
2294	[P].PQSAFPPAAFMPPVQ.[T]	1584.78791
2295	[L].ATPSSITWTQVSGLH.[P]	1584.80165
2296	[G].AASAGPQVSLYQGAPPA.[A]	1584.80165
2297	[N].GFPRAQHGPSPTVHP.[I]	1584.80298
2298	[A].MNRPIQGGMIRNPT.[A]	1584.80973
2299	[G].NRGVEAQAGHGIEFT.[P]	1585.77174
2300	[G].GALGSGAAGGGGKGSWGAAP.[A]	1585.77174
2301	[E].GPASTQASLATSGSTHL.[A]	1585.78164
2302	[-].MGARSAAAAAGGGWEGVR.[P]	1585.78298
2303	[G].GGYDFGFDGDFYRA.[D]	1586.65465
2304	[E].ATLGTYSVAVGDGKTF.[G]	1586.80606
2305	[P].GPSPGPAPPNYSRPHG.[M]	1587.76626
2306	[D].QSAPGPPAGGHHQPLH.[Q]	1587.7775
2307	[V].APGPEGLSTPRSQGPH.[V]	1587.78739
2308	[G].GPAGAKAPPPGPSTAALE.[V]	1588.83295
2309	[A].AAAAAAAAGSLSRSPFLG.[S]	1588.84418

2310	[S].PSGAKVSPARGFKEAS.[L]	1588.84418
2311	[H].QVAVRPTGEAAPAPQP.[P]	1588.84418
2312	[A].AGREGAQPGAQPGPEPA.[A]	1589.76666
2313	[G].DPSIPTQPTPTVAEH.[G]	1589.78058
2314	[E].ELNGYSRKKGGFSF.[R]	1589.80707
2315	[S].AAGGGPAGAAGGAAGGGPAAGPA.[D]	1590.76191
2316	[L].PCRIQASQRLMQS.[Q]	1590.78391
2317	[P].RTEPPSAGATARTEF.[S]	1590.78706
2318	[T].PQKVKQQTAVSETF.[S]	1590.8486
2319	[S].SGPAAASGPLATSSPAYS.[P]	1591.75984
2320	[M].YGAHIPAMPSAGVPHS.[L]	1591.76857
2321	[L].PGSWPKEAKDPGILP.[R]	1591.84787
2322	[N].PGGQLGAGSGGAYHARH.[A]	1592.76766
2323	[R].RAGGGGGGGVQNGPPASPT.[M]	1593.77281
2324	[A].AAEPAAAATTGPPAEATP.[G]	1593.77549
2325	[G].PGSSTSSLVPGPEPGPQ.[P]	1593.77549
2326	[Q].PGSPSPHPLGHYPGPP.[D]	1593.78085
2327	[K].GSGGVLGGIETGGPGGPGAP.[G]	1593.78672
2328	[T].AAAAAAASAASAVGPVHNS.[V]	1593.79796
2329	[G].GLNGVDVYSLVTEDI.[Q]	1593.80064
2330	[G].AAGVGGEAGPPPEREGSG.[P]	1594.74559
2331	[P].QCRVAPGGPEGPCPL.[R]	1594.74646
2332	[S].EAAAAAQPAGSPGETPAV.[A]	1594.77074
2333	[P].GESVVGAPGAPGTPGERG.[E]	1594.78197
2334	[R].GEARSGPAGGSGGYSGGGD.[H]	1595.66807
2335	[Q].AGIGGEPAAAGAGCSPRP.[K]	1595.75946
2336	[V].PGSPGIMGFQGFTGSR.[G]	1595.76349
2337	[D].GSSLSPAQSPSQGQPPA.[A]	1595.76599
2338	[Q].SALPSSPSSPNWTAPP.[E]	1595.77001
2339	[A].GSPLMPEVGSPQDPGK.[S]	1595.77338
2340	[P].PGENLASPGTPLSVPC.[W]	1595.77338
2341	[K].RSPGGGSPAAAAVPQGSE.[S]	1595.77722
2342	[S].SGPPGPPGPKGDQGPPGP.[R]	1595.78125
2343	[S].SWRPTGAPSPSGPELG.[R]	1595.78125

2344	[A].WNPGLPTPSSLGSPW.[R]	1595.78527
2345	[N].RGAQEPSTRSGGAPQP.[P]	1595.78846
2346	[V].PASQRAVMSSGHALSP.[I]	1595.79585
2347	[V].VQGGAATGQLLCQHR.[D]	1595.80708
2348	[R].QQQAPGGPGGVTGGKEE.[E]	1596.76124
2349	[Q].PGAGGGQSLPMAWQQL.[V]	1597.77914
2350	[A].PGAAGASVGAAAAAATPGTE.[D]	1597.78164
2351	[W].KGEGAPGQPAEDSVKQ.[E]	1597.78164
2352	[E].PGGADKQEVKGPSAQE.[P]	1597.78164
2353	[S].PAAREETASPGAKDTP.[L]	1597.78164
2354	[T].HGRATPSHTPTDALH.[A]	1597.78298
2355	[G].GSGALSPSAPGPPDQLF.[A]	1597.78566
2356	[L].SPTGQATTPATTGPPSQ.[P]	1598.76566
2357	[A].AAAPGEAELTSGSGRPQ.[L]	1598.77689
2358	[I].PGLPGSEGPPGPAGSAGPP.[G]	1598.78091
2359	[Q].GQPGPPPGAGAPPTERN.[E]	1599.78739
2360	[E].ASVFPQGAAPGPFAWP.[H]	1599.79544
2361	[P].QGPGTVGSGSPAEGPRF.[S]	1600.77141
2362	[K].APAWAGTAPPWDLTF.[T]	1600.77945
2363	[P].AAASAVPGSGAAAGALASGGS.[K]	1600.79254
2364	[P].LGGQGGGGPSPSPGGEPPP.[E]	1601.75542
2365	[G].KGAGGGPTAGSGTGLPTDT.[S]	1601.77655
2366	[L].GGPTTPASGPAPAPAEPAG.[S]	1602.77583
2367	[G].APGGAAPGGPGFRAFLC.[P]	1602.78456
2368	[S].GPWRSAPTQAGSSTTV.[C]	1602.78706
2369	[L].PGNPMIVATGPRFTE.[N]	1602.79445
2370	[I].QGPPGPPGPPGPSGPLGH.[P]	1602.80232
2371	[S].YFSIVAIVATRPHE.[L]	1602.86385
2372	[S].GPQGANSVPEPLVPIK.[V]	1602.88498
2373	[Q].RDQAPAPQPSLDTAH.[L]	1603.78231
2374	[G].PAGAGPGPGPGSGTAPEAR.[S]	1603.78231
2375	[L].APNTRGSPGPPPAKPC.[S]	1603.80094
2376	[G].FSAVEGAAAGPAPQGGAF.[S]	1604.77035
2377	[H].QHSPKFGSPGNHKSP.[S]	1604.79281

2378	[V].GRDPAAAPATGNRNTH.[L]	1605.78404
2379	[N].SSPVPSPGSPSGIGVGSH.[F]	1605.78672
2380	[Q].GPAGPPGPPGPMGPPGLPG.[P]	1606.80462
2381	[R].AQGRGEVGAGAGPGAQAGP.[S]	1607.78846
2382	[S].ASTPATSAPTPPVSSTH.[T]	1608.78639
2383	[T].GPAEGAAAATAPAEKPSN.[H]	1609.78164
2384	[F].PALSTPSSSAPSVPPGTG.[T]	1609.80679
2385	[E].PGPGLSSTSPVGEPSAGL.[G]	1609.80679
2386	[L].LDGGARGQNACSEIY.[I]	1610.72274
2387	[S].QAAAQPSTPAGTPRSGGG.[H]	1610.78812
2388	[K].AGPGERLDQAQFPPE.[A]	1611.77616
2389	[AV].QAQAQAQAQAQAQAQA.[Q]	1611.78337
2390	[P].PEPPGPLGAGAAGAPAGGAP.[E]	1611.81255
2391	[P].PGTPSVGSPGPLAPIPPA.[L]	1611.87408
2392	[R].GPGSPLSSGTAWLRLL.[Q]	1611.88532
2393	[S].TGGPPGPGPPPGPPGLSSS.[S]	1612.79656
2394	[G].FGAPPGGAGFPGYPQPP.[T]	1613.7747
2395	[G].PGSPGVPGSPPEAAAEPP.[T]	1613.78058
2396	[L].GSWRASPPAHGPAPPE.[Q]	1613.78191
2397	[K].EKAHTGPPSPSGSPAPP.[H]	1613.79181
2398	[S].GPPGPPGGPGMPPGGRGR.[G]	1613.79652
2399	[Y].QSHQPLPQAASQPAPG.[A]	1613.80304
2400	[L].PGSGPGPGGLPGSGPGPGGR.[A]	1614.79829
2401	[P].APGPPPAPSGGGAQGPRSG.[L]	1614.79829
2402	[A].PQPPGSAVSGPSVGQGPP.[D]	1615.80746
2403	[L].ATAWANLAAGGEKLQD.[A]	1615.80746
2404	[R].TPSGGGRAGAGPHLIRL.[Q]	1616.89795
2405	[P].HMSKTAPPSGARSHPG.[S]	1617.79143
2406	[P].GPPGSTASLSTASLTPSS.[P]	1617.79662
2407	[S].QGPAGPPGPPGPPGPPGPS.[G]	1617.80198
2408	[A].THSPQVPGPAPGFAAGQ.[Q]	1618.79723
2409	[S].QPSSLAARLGGPSSPPP.[H]	1618.85474
2410	[K].AMGIMNSFVNDIFE.[R]	1619.70801
2411	[G].GSTAPLSSSTPSPVSTSG.[S]	1619.77589

2412	[Q].AAAQPSTPAGTPRSGGGH.[S]	1619.78846
2413	[P].GPPGSLSSSGLEIQQY.[I]	1619.79114
2414	[P].AAGAAPAGGPAPATTAAPAE.[E]	1619.80237
2415	[G].AAGAQAAPQGPAGPTTSPA.[Y]	1620.79762
2416	[H].QPQPATTPTGSQPPSQ.[H]	1621.78164
2417	[G].SGPGPGPATGAKTEPGSGP.[R]	1621.78164
2418	[E].KREPDPSGGGPTAAGGAP.[A]	1621.79287
2419	[P].PGPSGPLGHPGLPGPMGP.[P]	1621.81552
2420	[W].PGSPQVSGPSPATRMPG.[M]	1622.79552
2421	[Q].QAAVTSSIMQAMRSAA.[G]	1622.79889
2422	[S].SEPGTPRPGRSAVRVG.[G]	1622.87213
2423	[A].QGRGEVGAGAGPGAQAGPS.[A]	1623.78337
2424	[P].PGPSLAETGSGTGDLAPP.[G]	1623.78606
2425	[S].ASQVSPTEPRSSPSPP.[P]	1623.79729
2426	[E].DGQLGPSLGAGPQAVEQ.[P]	1623.79729
2427	[T].LSSQSPAANLPGSPGSPG.[S]	1623.79729
2428	[R].VKACNPLDAGPMVVH.[C]	1623.79816
2429	[P].HPHGIQGGPGSQGIQGP.[V]	1623.79863
2430	[A].GQPAVGQGGVSGHRYPG.[A]	1623.79863
2431	[E].KEAQSPSPPYPTPAGP.[S]	1623.80131
2432	[L].SPCPPLVGTSAGVALVV.[V]	1623.87745
2433	[D].GGSPPRSGTTQVRILV.[L]	1624.91293
2434	[A].TQQGVPGSPTVNPSSIG.[N]	1625.81294
2435	[R].DGRGGRAGGAALAVVVLS.[L]	1625.90818
2436	[P].PGAATASVGGAEVPGSWN.[F]	1627.77107
2437	[Q].RLDGGSGGGPSAAGPGFPA.[A]	1627.78231
2438	[G].GPVGAGHGNPPGGGGSGPKA.[R]	1627.79354
2439	[K].MPGLAGQAAGSGDRPRS.[A]	1627.79691
2440	[G].QAVEAAAGAAGQLARED.[P]	1627.80344
2441	[P].PGADPARGAAGGGRFDR.[Q]	1627.80478
2442	[L].QEAPASLAGSAALGTFH.[G]	1627.80746
2443	[C].NVGAGGPAPAAGAAPAGGPAP.[A]	1627.81869
2444	[A].QRGWGCGQAWTPPAG.[G]	1628.73867
2445	[A].GAAPNATAAAAAAWTNIS.[L]	1628.80271

2446	[K].DGVKNTGAAGAGAAVCIP.[G]	1628.80608
2447	[M].PGPGPGPGPGPGPGPGPGHS.[M]	1629.77683
2448	[T].QENLPPPEANAEAIH.[F]	1629.78672
2449	[S].GPSGPPGPKGDDGIPGQP.[G]	1629.78672
2450	[G].GGSAAGARGAGAGAAASQEL.[N]	1629.79394
2451	[S].PGAKGEQGPAGHPGEAGL.[P]	1629.79796
2452	[A].AGAAAGAAAGAAAGLAAGSSW.[R]	1629.79796
2453	[S].GAAKDAAATAGPPSITSSG.[A]	1629.80785
2454	[A].DLASPVSSASSRSPTPA.[Q]	1629.80785
2455	[I].AKQGGGGGGGSVPGIERM.[G]	1630.79658
2456	[Q].QPSPPSGQVSPPPGDAL.[V]	1630.80713
2457	[R].PPGSGPGPGPATGAKTEPG.[S]	1631.80237
2458	[E].PGAPAPASSTTTHAAPAQ.[P]	1632.79762
2459	[G].AQLSGGQGAAEPPQPQP.[Q]	1632.79762
2460	[C].APSLEAGNPAPAAPCPL.[G]	1632.80502
2461	[G].DAPAEKERGPGGQQPP.[Q]	1633.79287
2462	[Q].SRPPESSPSPQPPDR.[G]	1633.79287
2463	[K].AAAGREPDPVAADGSHL.[Y]	1633.79287
2464	[K].PALGEVAGGQRDQAPAP.[Q]	1633.82926
2465	[F].PASKAQAAEGVWPGPAP.[A]	1633.83328
2466	[P].GPLSQTPPMQRPVEP.[Q]	1633.83665
2467	[L].QAAQQAVMGTGEPMDT.[S]	1634.71488
2468	[-].MGAQLSGGQGAAEPPQPQ.[P]	1634.77689
2469	[T].QSPELTPSGPAPAGPASA.[P]	1634.80204
2470	[A].AAQPSTPAGTPRSGGGHS.[P]	1635.78337
2471	[A].LAQPDSTAPGGATGQAPP.[S]	1635.79729
2472	[G].ESVWVAPSAPSTPGPGP.[A]	1635.80131
2473	[P].SPGKGGGGGAGTAPEKPDP.[A]	1636.79254
2474	[Q].GPQPQAQPHQVQPPQ.[P]	1636.81903
2475	[S].AAADSRPGPTAGDPQRA.[E]	1637.79902
2476	[R].PSGAATTTAAAAASAPAPGP.[A]	1637.81294
2477	[E].VGGGEAGTVIGGSAGPSPPA.[T]	1637.81294
2478	[G].EAAPGAAGASVGAAAAAATPG.[T]	1638.80819
2479	[A].GPSPASTPATADSSEQH.[F]	1639.71943

2480	[V].DNGNATANGNLGEAGPAP.[P]	1639.73067
2481	[V].SRAPASSGAPPTSTAQGP.[C]	1639.80344
2482	[A].SRRMQAGAVSPFGML.[G]	1639.80431
2483	[T].GREGTAGRGGSRAGSQH.[L]	1640.796
2484	[V].PGTSITTSAQSESIVY.[T]	1640.80137
2485	[S].VSPGTGGPSGPGGTMPIR.[I]	1640.80608
2486	[P].RPNSEAQGAPTLGGTEG.[W]	1641.7827
2487	[Q].GQAAPPAAAMQQHAVGH.[H]	1641.79143
2488	[A].AARGGEAAAEVTGWPAGA.[P]	1641.79796
2489	[E].KPMEIAPSAGFGGNLH.[P]	1641.80535
2490	[Q].DKASPFSAAALAGHMAP.[V]	1641.80535
2491	[K].QSSAGRAQGAAVGFPTH.[L]	1641.80919
2492	[R].GPEPAATAAYGSGDAPGGP.[C]	1642.73435
2493	[W].AGERGPGSPLSSGTAWL.[R]	1642.81836
2494	[P].RQHERQSQFQLW.[C]	1642.8197
2495	[V].PGGAAAAAAATVAAASATTAA.[S]	1642.83949
2496	[P].VPGSSGVGPGGVIRVPAH.[R]	1642.90236
2497	[S].GGGYDFGFDGDFYRA.[D]	1643.67611
2498	[D].AGPAGAFPGALDSGARTAG.[Q]	1643.81361
2499	[D].GAPAGGKSSFGHPYTIP.[E]	1643.81763
2500	[S].AAQPAPFPSTHSAPPPA.[C]	1643.81763
2501	[G].AAPGAAPASGPAASKFLC.[L]	1643.821
2502	[T].GPPAGAAGAAGGRGKSAYQ.[D]	1643.82484
2503	[G].VTNPQGTAPPELVAPPG.[G]	1644.85916
2504	[L].VNAGANVNQPNDKGFT.[P]	1645.79287
2505	[R].SPSPISNQPSPRNQH.[S]	1645.80411
2506	[R].KGPGPGGPGGAGGARGGAGGGP.[S]	1645.81534
2507	[H].QQVPGQWGPGQGGPRP.[P]	1645.81936
2508	[Q].KDHAVFTPRGEELF.[M]	1645.83328
2509	[P].SPGPGPSPGAMLGPSPGPS.[P]	1646.78428
2510	[P].AWKDGGPLDPSIYVE.[P]	1646.80606
2511	[L].GLPAKCSQASPPALSY.[V]	1646.82067
2512	[E].TGAPRPGGASGPGLDSHL.[Q]	1646.82451
2513	[K].PGAQGAAAGPGDVGPGSGAGP.[V]	1647.77214

2514	[P].PSAAASLGGSGAPPPPSMP.[P]	1648.79993
2515	[L].GRTQEPPAGGGGSIHDL.[P]	1648.80377
2516	[S].PGRCRTPAGEGPHTR.[V]	1648.80848
2517	[G].APQEPTPPGAAPRNVM.[D]	1648.81117
2518	[V].LPWGWGAGPSAPTGTPP.[Y]	1648.81182
2519	[P].GPRTDVGAPFGPQGHR.[D]	1648.83026
2520	[A].PATGPSVTNPFQPAPPA.[T]	1648.83295
2521	[R].AVPEPNTASATAQAPPAG.[P]	1649.81294
2522	[C].PGVPMAGVTTGYNGSLL.[P]	1649.82033
2523	[A].AKEPTPWAGDKGGAAPP.[A]	1649.8282
2524	[G].SPGEKGEKGETGQPGPP.[G]	1651.7922
2525	[P].FEELPAQFGAEQASK.[S]	1651.79623
2526	[N].PGPFNQGPPGAPPHAGGP.[P]	1651.79756
2527	[T].GPTGDKGSRGDPGTPGVP.[G]	1651.80344
2528	[A].RPACAGAAGRQSAGPQP.[S]	1651.80815
2529	[S].PRGGGPPSASAPAASESR.[P]	1651.81467
2530	[P].RGGGPPSASAPAASESRP.[L]	1651.81467
2531	[R].MAPYKGAGAPAGALDYV.[A]	1651.81485
2532	[S].GAAAATAATAGGQHRNVQ.[P]	1651.8259
2533	[S].PVADQVTGQPSSQLQP.[I]	1651.82859
2534	[S].FAPKSAVFSASWSAVP.[A]	1651.84787
2535	[V].PGAGGVQAGGSQRPGRTV.[S]	1651.86229
2536	[I].AEPGPPGPPGPPGPMGLQ.[G]	1652.8101
2537	[S].AAPGSATPAVGSAPAIADE.[K]	1652.81261
2538	[A].VAAAPAGEGTSAAPVSAEP.[G]	1652.81261
2539	[L].ADPRQAGIDSLLRKI.[Y]	1652.94423
2540	[L].NTTPPSAAHGSSKDNLG.[N]	1653.7827
2541	[T].GAKGESGVDGLMGPPGPQ.[G]	1653.7901
2542	[A].RGGEAAAEVTGWPAGAPG.[P]	1653.79796
2543	[G].RQEGPAGAAGAQAGAREG.[V]	1653.80517
2544	[T].QPGLPSSGQGGAASPGSSL.[G]	1654.8031
2545	[A].QPNSLSPSAPSSLCLP.[E]	1654.8105
2546	[P].AATSPPASAPQTAMQLP.[A]	1654.8105
2547	[P].AAGSASARGSTPAATNPPA.[P]	1654.81434

2548	[S].VSTGSRAGGAAGVGGEAGPP.[P]	1654.81434
2549	[T].PGLDSELSLEGLDDVP.[P]	1655.80104
2550	[I].PGLPGSEGPPGPAGSAGPPG.[Y]	1655.80237
2551	[E].PATPTATQAGHALPLLP.[Q]	1655.91153
2552	[Y].PGTSVPGQPTQDGCQQ.[Q]	1656.72822
2553	[V].SPQSPQKSDCQPNSP.[T]	1656.72822
2554	[A].AGSAARGGTAHGAGAGGDDT.[V]	1656.73206
2555	[R].PGPSPGPGPSPGAMLGPSP.[G]	1656.80502
2556	[G].PGSPFGLEPQSGWLW.[V]	1657.80092
2557	[T].AGSPATAAGPATATEEAKG.[R]	1657.80277
2558	[V].PGSPGFPGVPGSPGIMGF.[Q]	1657.80429
2559	[G].GPRLCPSATGGARSPSS.[P]	1657.80748
2560	[-].MAAGVEAAAEVAATEPKM.[E]	1657.81016
2561	[K].GNPRTTSQPATGTTATP.[K]	1657.814
2562	[E].GVSGPEKGGGSAAAAAAAAS.[G]	1657.814
2563	[P].RNHFGVGGGGVTCNII.[I]	1657.82273
2564	[N].TPAPVAMPASSPPGPPPA.[P]	1657.82542
2565	[P].GSDYFPGGTAPGAPGPGGP.[S]	1658.74453
2566	[E].AGAGPATTTTPPGPPAGHT.[L]	1658.81327
2567	[P].SGPGPVTQGPQQPQPPS.[Q]	1658.81327
2568	[S].AAPGGGSVAAASAAMGAALAS.[M]	1658.81664
2569	[P].SGPPGPPGFPGDRGLPGP.[A]	1658.82853
2570	[V].GPSGPPGPPGFPGDRGLP.[G]	1658.82853
2571	[Y].TAAPGGGPAGAKAPPPGPST.[A]	1658.84966
2572	[S].QPDKPSAPAAAAAAAQPP.[A]	1658.84966
2573	[P].QERREGTAGSSGGSAPL.[G]	1659.8045
2574	[P].GPRGEPGAAGIPGEPGSPG.[K]	1659.80852
2575	[D].AGGLGPAGNAASTAGPFPF.[H]	1659.81255
2576	[Q].PSQSRNPGPAAARTYS.[T]	1659.81976
2577	[D].VTQQPSPPSGQVSPPPG.[D]	1659.83368
2578	[K].EGPPGTKGNQGPSGPQGP.[L]	1661.78779
2579	[A].AQPSTPAGTPRSGGGHSP.[A]	1661.79902
2580	[P].AASTPAGPPSGGASPTPPAA.[S]	1661.81294
2581	[V].AAHTKQGPSVAGGSGTVH.[T]	1661.83541

2582	[P].SVPTPTGEVGTAPSAPPP.[R]	1661.83809
2583	[V].QPGADQQGLVQPVIDP.[R]	1661.84933
2584	[P].EEKPGPGAAPQAEPRE.[Q]	1662.80819
2585	[G].RLRNGIGGASVSPGPGAP.[L]	1662.90343
2586	[D].PGGEPPSPSKEMHGSGP.[L]	1663.73806
2587	[H].NLADNVQPSSTENTSS.[P]	1663.74056
2588	[C].GGELPTQAGAGPGPSPDR.[L]	1663.80344
2589	[N].PGGQLGAGSGGAYHARHA.[A]	1663.80478
2590	[Y].APGGAAATANAVAHGLNGGS.[P]	1663.81467
2591	[K].KGGGAAGGGGVASGGAGGPQPP.[Q]	1663.81467
2592	[T].PPSPSFAATGASSANRF.[V]	1664.80271
2593	[G].YPGNAGPVGTAGAPGPQGP.[V]	1664.80271
2594	[G].KDGDRGAPGAPGEAGRPG.[L]	1664.80992
2595	[V].AAAEPAAAATTGPPAEATP.[G]	1664.81261
2596	[P].PATPSEAPAAASSPATAPA.[P]	1664.81261
2597	[G].RSDPGVPGAAGGEGPVEL.[A]	1664.82384
2598	[A].AAIGWMPVASGPMPAPP.[R]	1665.81275
2599	[N].LYPPSNAPGEGLSHGGL.[R]	1665.82311
2600	[P].GTSAPRGTAPQEPSRGP.[G]	1665.83032
2601	[L].RSIMSSATAYLCGHL.[H]	1666.80397
2602	[S].PPYPTPAGPSALWPES.[E]	1666.81115
2603	[E].GATPPPQASSPAHSFSK.[A]	1666.81836
2604	[G].GPGSQGIQGPVSQGPLMG.[L]	1666.82173
2605	[V].THTTTATPTGPHTPFT.[T]	1667.80237
2606	[L].QGQVDKQYAGLKDMA.[E]	1667.80575
2607	[P].GAPSMAGTVAPGGVSGPSPA.[Q]	1667.80575
2608	[A].ARGGEAAAEVTGWPAGAP.[G]	1667.81361
2609	[S].QGSPQPLGSGGPGAPPHQ.[L]	1668.80886
2610	[H].VWEQKAGGASPEEPLA.[P]	1668.82278
2611	[V].PGVDVSSSLGGGAVEGQGP.[S]	1669.80277
2612	[D].SVSTMVVHDVEEIAGP.[Q]	1669.81016
2613	[E].QGPGGAGVSGGLMVPISAD.[P]	1669.8214
2614	[P].PGIDGKDGTPGMPGVKGS.[A]	1669.8214
2615	[G].GVANTNRAGGGVATEAQP.[R]	1669.82524

2616	[G].STPHVSGLGSPGRYSPA.[N]	1669.82926
2617	[Q].AAEPNKGGPGSRGCRR.[S]	1669.82994
2618	[K].AGPAFGGGVTQQGITSSH.[V]	1671.80852
2619	[P].SRHSLSGSSPGMKDIP.[R]	1671.81189
2620	[L].AAGPTGSAAPAATEGADEE.[R]	1672.72966
2621	[A].APAAEAASAAPDPPAAGAAP.[A]	1673.81294
2622	[P].PGAPPHAGGPPPHQYPP.[Q]	1673.8183
2623	[E].GLPQGTSSAPQAPAHPTG.[A]	1673.82417
2624	[-].MDSPPRASATALSAPWF.[R]	1673.8282
2625	[G].GPQPAPSPAGTGTRLGPL.[T]	1673.89694
2626	[G].AGPEKTAGVSTACSPLE.[D]	1674.80033
2627	[E].QASTAPPEPAAHAAAPTS.[L]	1674.80819
2628	[S].QGPAGPPGPPGPPGPPGPSG.[G]	1674.82344
2629	[L].QPMQRPSTLPASAAGY.[Q]	1674.82682
2630	[V].GQPGPQGRQGPKGEQGP.[P]	1674.83066
2631	[M].GPSALGQSGPGSMAPWC.[S]	1675.7203
2632	[T].VGGGAGGVGGGAGSEDSGDRG.[G]	1675.72664
2633	[H].PGSPGPAGSPGLPGVPGSMG.[D]	1675.81083
2634	[V].KMASPPPSGPPSATHTP.[F]	1675.81083
2635	[Q].PPGSAVSGPSVGQGPPDAV.[R]	1675.82859
2636	[G].TPTQQPSTPQTPQPPA.[Q]	1675.82859
2637	[P].PGKMGPQGTPGIPGMPGP.[I]	1675.82946
2638	[L].EHPGDTTGTSAGLEAFS.[P]	1676.73983
2639	[G].AGGVGGGAGSEDSGDRGGTL.[G]	1676.74704
2640	[R].GVDSPLSSPSPPGPQASP.[V]	1676.81261
2641	[S].PGALSNASAPVDFFLNG.[R]	1676.82786
2642	[V].SPNRPTPSDLAIVMY.[T]	1676.83123
2643	[P].GPAGPRGHPGPSGPPGKPG.[T]	1676.86156
2644	[D].QPDPGAVAATAILRAIL.[E]	1676.96938
2645	[V].AAVQQEAPSAQSPGVHT.[D]	1677.81909
2646	[A].GSGPGPGVSAAPGPAAAANAT.[P]	1677.81909
2647	[A].GAAGAQAAPQGPAGPTTSPA.[Y]	1677.81909
2648	[R].PGQAINGKDGDRGAPGAP.[G]	1677.83032
2649	[A].ASPSGLWPGGELRDVH.[T]	1677.83434

2650	[R].PGAGAGAGENWEPRVLP.[Y]	1677.83434
2651	[V].RQGLLDTSLTASMATL.[L]	1677.884
2652	[A].SPSRGPGTAGTLHLVDL.[A]	1677.89186
2653	[P].QPAPSPAGTGTRLGPLTG.[E]	1677.89186
2654	[H].QGTSPVPGPSQPTRRL.[I]	1677.90309
2655	[G].GPTWTGPLAGAAGRAVPV.[Y]	1677.90711
2656	[P].ADLEPMLPPGSLVILP.[T]	1677.91317
2657	[T].GAEVEPVAQAKPEKKP.[G]	1677.91701
2658	[L].PQGVVMAASPGSLHSPQ.[Q]	1678.82173
2659	[V].ANVNPGGWAPASVLRAV.[A]	1678.90236
2660	[G].MPGQKGQPGSPGLSGQPG.[L]	1679.81698
2661	[M].PGMPGQKGQPGSPGLSGQ.[P]	1679.81698
2662	[S].PTGQATTPATTGPPSQPA.[N]	1679.8235
2663	[G].DREPRGPPGGSRRQD.[E]	1679.83205
2664	[S].PGSPPSPGLDSGSSSALAP.[P]	1680.80752
2665	[G].AQQSLGGGWAGGGRAGPGP.[P]	1680.82009
2666	[A].PSGREVSAGARWLSHA.[R]	1680.85648
2667	[P].AATAAPGAGFGFASKTKK.[K]	1680.90678
2668	[A].AAAQPAGSPGETPAVAAES.[P]	1681.80277
2669	[A].AAIGWMPVASGPMPAPP.[R]	1681.80766
2670	[G].AADASKGLGGSGGAGGPPGTP.[Y]	1681.814
2671	[A].AAAPSPGSARSAGSHTTR.[D]	1681.83647
2672	[G].PGSPLSSGTAWLRLLQ.[G]	1682.92243
2673	[Q].QRQAGSPGGGGGSAGTPEL.[S]	1683.8045
2674	[K].DVTPSPAPGSTVQPGHH.[I]	1683.80852
2675	[G].PSAGAAPNATAAAAAAWTN.[I]	1683.80852
2676	[S].PGPSWPAAYGAPLRED.[W]	1683.81255
2677	[-].MAAGGGGPAPLSSAASSPLSS.[S]	1683.81842
2678	[Y].YASQGISPGPPRPCTP.[A]	1684.81117
2679	[G].AAPNATAAAAAAWTNISL.[P]	1684.86531
2680	[R].PGSTRWGWAADAATAPA.[A]	1685.80304
2681	[K].DGVKNTGAAGAGAAVCIPG.[E]	1685.82754
2682	[L].PGSGPGPGGLPGSGPGPGGRA.[A]	1685.83541
2683	[S].GPSGPPGPKGDDGIPGQPG.[L]	1686.80819

2684	[P].QGPGTVGSGSPAEGPRFS.[L]	1687.80344
2685	[T].TGPTGPPAAGPTGPPTAGPS.[A]	1687.82859
2686	[P].PRTEPPSAGATARTEF.[S]	1687.83982
2687	[A].EAVAANPGAMLELGPPH.[G]	1689.82648
2688	[S].RRPDSSGPGAGAAPEPPA.[S]	1689.83032
2689	[A].PGGAAATANAVAHGLNGGSP.[A]	1689.83032
2690	[I].ATHSPQVPGPAPGFAAGQ.[Q]	1689.83434
2691	[D].KGNPGWPGTPGAPGPKGD.[P]	1689.83434
2692	[L].KGDKGNPGWPGTPGAPGP.[K]	1689.83434
2693	[A].AAQAQVPPGSAGPLASNPG.[S]	1689.85547
2694	[H].GLAMAPASVAPAPAGSGAPP.[G]	1689.86287
2695	[Y].PGAKGGFQVPMIPDYL.[F]	1689.86689
2696	[N].PQQFPGQPAMMQPMA.[H]	1690.7386
2697	[M].GLGPQEPQMVSQGEMS.[F]	1690.7411
2698	[G].TAPQTAQQAGPEAAGHR.[A]	1690.82557
2699	[G].PGSSTSSLVPGPEPGPQP.[A]	1690.82826
2700	[A].EAAAAQAPAAGPGQMSFT.[F]	1691.76936
2701	[V].GPADQTVPPGSKQESPP.[A]	1691.8235
2702	[N].QPSSAVHSSTVISTGAY.[G]	1691.8235
2703	[P].ASSPPQPASPSPCPRR.[C]	1691.82821
2704	[P].GPGPSTGALQERSPGSPP.[G]	1691.83474
2705	[P].PGESVVGAPGAPGTPGERG.[E]	1691.83474
2706	[N].AGDLAPAGGAAPAPREEAA.[D]	1691.83474
2707	[A].PPTNQATAAASAPNASPQ.[S]	1693.814
2708	[P].SGPATQPPVSSATMHLP.[L]	1693.8214
2709	[P].GEIQGPASPAGGNQSPRA.[R]	1693.82524
2710	[F].GGRDGPLSPQGGGGGVAPGT.[A]	1693.82524
2711	[P].PGGSPGRSSPAGGSPGKPGS.[T]	1693.82524
2712	[Q].PPPQHPSQPSAQSAPAP.[A]	1693.82926
2713	[P].RAHSAGSPASGAGKESPGA.[A]	1694.82048
2714	[R].AQGRGEVGAGAGPGAQAGPS.[A]	1694.82048
2715	[V].PGMPGTKGGPGDKGEPGR.[Q]	1694.82788
2716	[Q].AGQPAVGQGGVSGHRYPG.[A]	1694.83574
2717	[P].GPGVSFSPGPTPTPAPTAG.[S]	1694.83843

2718	[A].GGGGSGALPAGTANSGTARH.[W]	1695.81573
2719	[P].PGPSGVPPGMPGQPPGGPP.[K]	1695.81592
2720	[L].SPTGQATTPATTGPPSQP.[A]	1695.81842
2721	[A].GPTGPPGVPGSPGLTGPPGP.[P]	1695.87006
2722	[E].PGSKGDRGEPGQRGQNG.[I]	1696.81098
2723	[T].PTAQSGQGAVPSGDLGAGGA.[G]	1697.80892
2724	[A].STSDPLPAGGGSARPGSQG.[L]	1698.80417
2725	[L].PSQPPGPMPPQQHLMG.[K]	1698.80906
2726	[A].QAQAQAQAQAQAQASQA.[S]	1698.8154
2727	[A].QAQAQAQAQAQAQAQAS.[Q]	1698.8154
2728	[D].VGEEGRVASGGPPGLETS.[E]	1698.82932
2729	[L].GAWAGLGPGQGEQTVTVA.[V]	1698.84457
2730	[L].PLVLGPLGGAPTVEGPGAP.[P]	1698.9425
2731	[V].PGKDGQAGHPGQPGPKGD.[P]	1699.81467
2732	[I].PDGRNFPPGQGIFSGPG.[R]	1699.81869
2733	[G].PGKDRDAAGGGASGGRDR.[E]	1699.82188
2734	[F].KPETMFAATDLYIAE.[H]	1699.82475
2735	[L].TQKADYAAPQAPSSPAP.[P]	1699.82859
2736	[S].PDGKGSATRGGQQGRPM.[V]	1699.82928
2737	[P].TASGNRASPAAASAVPGSGA.[A]	1699.8358
2738	[T].QPGAAAASAPPRAVPEPN.[T]	1700.87146
2739	[P].GSPGEKGEKGSTGIPGMP.[G]	1701.81123
2740	[P].TTGPTGPPAAGPTGPPTAGP.[S]	1701.84424
2741	[S].TPAGPPSGGASPTPPAASPS.[G]	1703.8235
2742	[T].SSPRAMPPSPGPTERH.[A]	1703.82821
2743	[-].AGTTHTSLGPAPSAHDAL.[R]	1703.83474
2744	[E].QNRSAATPPSQPPQQP.[S]	1703.84597
2745	[A].EQQISPPNTNAKSYE.[E]	1705.80277
2746	[G].VSAAGGGPAGAAGGAAGGGPAAGP.[A]	1705.82524
2747	[T].AGPGAVSAGALEPGATTAAH.[R]	1705.85039
2748	[P].ALAQPDSTAPGGATGQAPP.[S]	1706.8344
2749	[H].QAAPPTSSPAASFPPPPS.[S]	1706.83843
2750	[T].GPDTQLPSGPTQNPAID.[F]	1707.81842
2751	[L].PGSPGEKGEKGETGQPGP.[P]	1708.81367

2752	[S].GTPTGGPSPATSSPVPPMA.[S]	1708.82106
2753	[T].GPTGDKGSRGDPGTPGVPG.[K]	1708.8249
2754	[L].AAPSSPSLSHRQGMGPL.[G]	1708.84353
2755	[Q].MKGEAAAAGGPTADPAPTP.[S]	1709.81631
2756	[P].PPGAGPDPPSPPGADPARG.[A]	1709.82417
2757	[I].AEPGPPGPPGPPGPMGLQG.[M]	1709.83157
2758	[V].LDPTQGFFHSAGTPVH.[F]	1710.82344
2759	[L].AAAATTPAAAFGTSTTTIS.[T]	1710.85447
2760	[P].GMRGPPGPGPPGPVGDPGP.[I]	1711.82206
2761	[T].QPGLPSSGQGGAASPGSSLG.[L]	1711.82457
2762	[P].GLSSSGVSAASQGAGGGPPPA.[P]	1711.82457
2763	[E].EPVDGLAGSAAGPGAESRA.[G]	1711.82457
2764	[T].TLSSFQATSASVTQFH.[P]	1711.82859
2765	[L].TPGGMALPGQPGGPFLNT.[T]	1711.84722
2766	[A].GTPTSGSPTAGTAATAEHV.[V]	1712.80858
2767	[L].PQAASQPAPGAPHLQPM.[Q]	1713.83772
2768	[A].AAAATTPATPSGSAAFQPP.[R]	1713.84424
2769	[R].QGPKGEQGPPGIPGPQGL.[P]	1713.89186
2770	[G].QDVALPWSAAGGLSVSR.[A]	1713.89186
2771	[L].GPAGEGVVGAPGFLRRSS.[S]	1713.90309
2772	[S].AAGAAHPPGTPFGPPPHH.[S]	1715.8401
2773	[L].LSMPGAQGAPAAGPEPPPA.[T]	1715.84213
2774	[G].MPGIGGSPGITGAKGDMGL.[P]	1715.8455
2775	[G].GPQNLSGPGGRERDYI.[A]	1715.84597
2776	[V].GPSGPPGPPGFPGDRGLPG.[P]	1715.84999
2777	[L].SPGPDAPLAPASSAGPGPGL.[S]	1715.85989
2778	[G].EREPQVPWGRLDHP.[G]	1715.86123
2779	[L].GPSPGPSPGSAHSIMGPSP.[G]	1716.80099
2780	[P].GPSPGSAHSIMGPSPGPPS.[A]	1716.80099
2781	[V].ENGKPGADVVDLTLDSS.[S]	1716.82865
2782	[Q].QQAPGQAPINSSGFAFP.[P]	1716.83401
2783	[-].MAGGGRQPAPGSTRIPRP.[P]	1716.92522
2784	[V].PAGPVSSSSTATSVTASNP.[S]	1717.8239
2785	[F].GSISSSGALFSAGSQPAPP.[T]	1717.83915

2786	[S].GAQFFTLEGRHPDRS.[V]	1717.84049
2787	[T].ARPSEGPTTGPTGPPAAGP.[T]	1717.85039
2788	[G].SGPGPGVSAAPGPAAAANATP.[A]	1717.85039
2789	[L].GTAAWPSLQQGLQQSF.[L]	1718.84966
2790	[S].TEAQGVAGPAAEIPASGGH.[G]	1719.82965
2791	[A].PGTSAGLGQGQGQLVGMY.[Q]	1720.83229
2792	[P].GPPSAFNLNSDTDEEE.[S]	1721.71368
2793	[V].AAAEPAAAATTGPPAEATPG.[K]	1721.83407
2794	[A].QASPSPTVSWTYPLST.[W]	1721.83809
2795	[K].NVAAEERAAPSPAPAEAA.[P]	1721.8453
2796	[P].GRSDPGVPGAAGGEGPVEL.[A]	1721.8453
2797	[V].PGHHVTPGHFLPSQNP.[P]	1721.85066
2798	[T].GPGVSNLEGPVIEMAVH.[A]	1721.8527
2799	[G].PGPGPGPGPGPGPGHSMRL.[P]	1721.85403
2800	[E].KPDNSAAGTVPSGQKQH.[D]	1721.85654
2801	[S].LICNVGAGGPAPAASAAPAG.[G]	1721.86393
2802	[F].PSDAGVPLSSATLLAPLL.[S]	1721.96838
2803	[A].AGLEEASAAVAVGAGGAPAGP.[A]	1722.8657
2804	[V].RAGMRARPAAAPDPAAAG.[G]	1722.88165
2805	[G].ADPSALAAPSAGGLQLRK.[H]	1722.94971
2806	[L].PGKGGSSPSQSPCSDFE.[E]	1723.7228
2807	[S].RGSFSHGGGGLGSGVSTGF.[L]	1723.81467
2808	[M].SPQTQPGLPSSGQGGAASP.[G]	1723.82457
2809	[A].QPLEWAQGPGQLTSDP.[P]	1723.82859
2810	[Q].EGLDLTGTATTATSFAAP.[P]	1723.83849
2811	[T].RQEGCPLVSPTPSPAQ.[P]	1723.84319
2812	[W].GQAASSLSATPGPETPRP.[P]	1723.86095
2813	[E].PGERMRIQAEGPGRGP.[E]	1723.86566
2814	[A].EAGPQAPPPPGTPSRHE.[K]	1724.83507
2815	[S].LTPAMSVSASASTSQASI.[C]	1724.83711
2816	[E].GPAGAAGAQAGAREGVDRN.[S]	1724.84228
2817	[T].TLSSQSPAANLPGSPGSPG.[S]	1724.84497
2818	[A].QPPRQNTGSGLCGKPQ.[A]	1724.84968
2819	[T].NPATGKGAGGGPTAGSGTGLP.[T]	1724.8562

2820	[L].GPFSELQNRGSRHSGP.[F]	1725.84155
2821	[-].MAAAAAGAGAGAAQEKQFPP.[A]	1725.85547
2822	[R].LEQELGHLKADLSSW.[Q]	1725.88063
2823	[M].PSVWDRDSGLAENPPS.[E]	1726.8031
2824	[-].MPSVWDRDSGLAENPPS.[E]	1726.8031
2825	[R].PRGGGAGGSSVGTVGGGAGGVGG.[G]	1727.84195
2826	[S].VSSPAGSPGPPGSTASLSTA.[S]	1727.84463
2827	[P].PGIMAPPPGMRPPWAH.[H]	1727.85086
2828	[K].QQGQAATPSVGRASSSPV.[E]	1727.8671
2829	[V].AGQGTGAVAIGAGGVAVTSSP.[F]	1727.89225
2830	[P].GPKGERGPAGPPGRSGPPG.[P]	1727.89359
2831	[P].QAASQPAPGAPHLQPMQ.[R]	1728.84861
2832	[S].PGPGPSPGAMLGPSPGPSPG.[S]	1729.8214
2833	[Q].MKATSAASTQPEHAGAR.[G]	1729.82861
2834	[G].KSCARSEAAAAAQPAGSP.[G]	1729.82861
2835	[P].GADPARGAAGGGRFDRQA.[S]	1729.8477
2836	[A].QPQFRPHMPAAQPQP.[S]	1729.85912
2837	[G].QRDQAPAPQPSLDTAH.[L]	1731.84089
2838	[Y].NRAGTGPASPSANATTMK.[P]	1731.84426
2839	[G].PGAPQYPPGPQGEPLEV.[P]	1732.85408
2840	[P].PGPKGDRGEQGDPGLPGV.[C]	1732.86129
2841	[R].PATASQRSPSKHGGPSAP.[G]	1732.87252
2842	[R].GPDGLNRGFAPDGHRAP.[L]	1733.84664
2843	[P].GPPGPSGPATQPPVSSATM.[H]	1735.83196
2844	[A].REEGVPGGGARAGEKEH.[P]	1735.84703
2845	[E].AAAAAQPAGSPGETPAVAAE.[S]	1736.84497
2846	[E].NPAGLRCASPRSRHSA.[Y]	1736.87214
2847	[P].PGLSSSGVSAASQGAGGGPPP.[A]	1737.84022
2848	[G].GAQQSLGGGWAGGGRAGPGP.[P]	1737.84155
2849	[V].AAGSPLMPEVGSPQDPGK.[S]	1737.84761
2850	[G].PGPGPGPGPGPGPGHSMRL.[P]	1737.84895
2851	[G].PADVGGAAAAPGGGAGGSREL.[E]	1737.85145
2852	[P].SPSLNPHPSPSPSPISH.[C]	1737.85547
2853	[H].VSSSSRQPASPGGDHLR.[R]	1737.86268

2854	[A].PGPFAGPQAQQAAREVN.[T]	1737.86671
2855	[AV].QAQAQAQAQAQAQAQAQ.[A]	1739.84195
2856	[E].MLQPYPSAPSSGPAVTH.[L]	1739.84213
2857	[I].SANPGGVSREGPPPPPSH.[P]	1739.84597
2858	[T].SGPNQALPGTTSQQTVPG.[H]	1739.85587
2859	[E].KPDSSTITQGVPTPGPSA.[N]	1739.88102
2860	[P].APAPGDFAPVFARRLR.[A]	1740.96563
2861	[I].PSQISYPASQGAYYIP.[G]	1741.84318
2862	[G].WLKADDSVPPGGSHIY.[N]	1741.85441
2863	[S].NFQSVGQVFPSLGFGTG.[G]	1741.85441
2864	[A].GARGGAEAASAGATGAAKGGPG.[R]	1741.8576
2865	[P].AVDPPTPGPGHPTHPAPP.[L]	1741.86564
2866	[S].GAGKESPGAASTPRGGQSQ.[Q]	1742.84161
2867	[A].VGEPASGTPATVGSLPSSE.[S]	1742.8443
2868	[V].WIGFSAVEGAAAGPAPQGG.[A]	1742.84966
2869	[G].REAGEGGVAAAAAALAPGGF.[L]	1742.88202
2870	[A].AGAPAGGAPERQSVIQFS.[P]	1742.88202
2871	[P].SPGPGPSPGAMLGPSPGPSP.[G]	1743.83705
2872	[A].PPASATSTGAPTSTGRPST.[T]	1743.85078
2873	[G].QPADKASASGSGAPVGGSIS.[S]	1743.85078
2874	[-].MTHLGISGGLGLGPGYDV.[P]	1743.87343
2875	[L].PAGGGGQVTGCGDPGDVPF.[L]	1744.75952
2876	[P].GAPGGPFPSSLPGSLLPPP.[P]	1744.92685
2877	[-].AGTTHTSLGPAPSAHDAL.[R]	1745.8453
2878	[F].ATAPSGSPPVFGNTPAFGA.[V]	1745.84933
2879	[L].QRQQGQAAGGRGPHGGPS.[Q]	1745.85385
2880	[L].GRTQEPPAGGGGSIHDLP.[L]	1745.85654
2881	[P].GPSGLGGAGGLAGLTFPWM.[D]	1745.86795
2882	[T].VQGRIIVQGSPVSTAPH.[C]	1745.96569
2883	[G].KSGSMGPAGPPGPAGERGH.[P]	1746.83403
2884	[S].PVMPPQTQSPGQPAQPA.[P]	1746.84795
2885	[Q].SPKQEAGGAAPGQHRGQA.[T]	1746.86302
2886	[C].PGVPMAGVTTGYNGSLLP.[E]	1746.8731
2887	[R].QRASNQGPGAVSGMEDQ.[G]	1747.7664

2888	[P].APGGYPAPGGYPGAPHPGGA.[P]	1747.81869
2889	[P].QQQAPGQAPINSSGFAF.[P]	1747.83982
2890	[S].QAAAQPSTPAGTPRSGGGH.[S]	1747.84703
2891	[H].GPGSSTSSLVPGPEPGPQP.[A]	1747.84972
2892	[P].LGAGAAGSQGPAGGPAPDTPV.[P]	1747.86095
2893	[A].KSEPAQPSPGSPRGQPQ.[D]	1747.87219
2894	[G].LGASAEQPAGGAEGFHLH.[G]	1748.83507
2895	[P].AASTPAGPPSGGASPTPPAAS.[P]	1748.84497
2896	[R].ARGMSASSIGSSYGGSAFG.[F]	1749.78607
2897	[Q].QSSASLAAATATSEAVPST.[T]	1749.85011
2898	[P].GPRGPEGAMGIPGMRGPP.[G]	1749.85232
2899	[R].LNGGVWGPWYKEESK.[Q]	1749.8595
2900	[A].GPKGDPGSRGPMGMRGPP.[G]	1750.84757
2901	[T].PGMPGVKGSAGQAGRPGNP.[G]	1750.86533
2902	[N].TTGDPGNSPLAISSFAGC.[W]	1751.79049
2903	[V].RQAPEGGESSPPAESRP.[D]	1751.83071
2904	[L].NPGGGLAASGAPWYPIHS.[R]	1751.84999
2905	[V].AAMTAKGQGQVSASTISSG.[V]	1751.85924
2906	[W].PNSVMAPGRGPERGGGGGV.[S]	1751.86058
2907	[-].MTSDVPPLGPAIASGNAGP.[G]	1751.86326
2908	[S].PGRNPMVQQGNVPPNF.[M]	1751.8646
2909	[L].GSPGPGSRRGGSPQTAVSP.[A]	1751.87833
2910	[A].GPARCSLAGSPAPGGGAAAGA.[K]	1752.84459
2911	[D].NSPRNGSVMGPPFAEPP.[T]	1753.83263
2912	[M].VNPQSQNLGPSPQRMT.[P]	1753.86499
2913	[P].KPDASPSVSSAPATRDAP.[E]	1753.87152
2914	[V].PTAGSVPSPSGSVPGAAAPF.[R]	1753.87554
2915	[G].NASGLGAGPGPSVGMGVVPD.[P]	1754.83777
2916	[Q].EKPMEIAPSAGFGGNLH.[P]	1754.85303
2917	[F].PGLHEQAAGHASPNVVNG.[Q]	1754.85687
2918	[G].WVGEAGQVAAAAKKRRG.[S]	1754.97726
2919	[P].PGADPARGAAGGGRFDRQ.[A]	1755.86335
2920	[R].PSGPGPVTQGPQQPQPPS.[Q]	1755.86604
2921	[S].PGSSNSRPPSPVDPYAK.[M]	1755.86604

2922	[S].LGAAGEGPPGAPSHASEVGP.[S]	1757.8453
2923	[I].ASAPDPLGGALGGEAPPGHS.[T]	1757.8453
2924	[A].SPGPPSPAASRSGMSTAAI.[K]	1757.84867
2925	[D].RSHQTGSVSTQPREST.[C]	1757.85251
2926	[Y].QVANNQAAGFGVQGQTPA.[Q]	1757.85654
2927	[L].PGAHGPPGPTGPKGEPGFT.[G]	1757.86056
2928	[C].PGGQGALLCPPGTFRTE.[P]	1757.86393
2929	[A].QSQRGTPGAGGAGRARGSS.[F]	1757.87498
2930	[P].PGSPGQAGAVGIPGERGPPG.[P]	1757.89292
2931	[N].RNSGQLEPGPAGAPSPAPG.[L]	1759.87219
2932	[R].VVSHSGSAGLPQVRVVAP.[P]	1759.98134
2933	[S].EPVSPDAALGSSSVAQVM.[P]	1760.83711
2934	[Y].APGGAAATANAVAHGLNGGSP.[A]	1760.86743
2935	[P].GPRGSMGPVGPSPDLSHI.[K]	1760.87483
2936	[E].RAGRGASEGPPAPALPCP.[G]	1760.88606
2937	[F].SVAITPDHLEPRLSII.[A]	1760.99051
2938	[T].QPTGQPAPDALYPNGVH.[P]	1761.85547
2939	[H].QPTANPGLGGPYLYQW.[N]	1761.8595
2940	[G].TAPQTAQQAGPEAAGHRA.[S]	1761.86268
2941	[G].PGSSTSSLVPGPEPGPQPA.[L]	1761.86537
2942	[P].QALGAEAGASLQAYQQR.[L]	1761.88784
2943	[M].GPSALGQSGPGSMAPWCS.[V]	1762.75233
2944	[S].GVSAAGGGPAGAAGGAAGGGPAAGP.[A]	1762.8467
2945	[N].TRSASFSQGTRASFLM.[R]	1762.85409
2946	[P].QAPDRWSPGLENGHSL.[S]	1763.84597
2947	[G].QATTPATTGPPSQPANPQ.[E]	1763.85587
2948	[A].GSGAPPGSLGPSEQLGQAGP.[T]	1763.85587
2949	[P].SPGALSNASAPVDFFLNG.[R]	1763.85989
2950	[S].GNEVPSQLASSGHIVRL.[E]	1763.93987
2951	[S].SPGGVYATRSSAVRLRS.[G]	1763.9511
2952	[I].TNVQVSGGGPGVSMVMKT.[L]	1764.86188
2953	[S].PVADQVTGQPSSQLQPI.[T]	1764.91265
2954	[D].GAVGEELQGQQHLMPR.[V]	1765.86499
2955	[M].RTGWGNRGVEAQAGHGI.[E]	1765.88409

2956	[-].MRTGWGNRGVEAQAGHGI.[E]	1765.88409
2957	[A].PGAATASAPVNPFQVNQP.[Q]	1765.88677
2958	[G].GPLGSASASTPARLLPRD.[C]	1765.95552
2959	[E].KELLKVQSWAGGAGAGPP.[R]	1765.95954
2960	[L].PSEEDSGAGPPLEGDGVPG.[G]	1766.77153
2961	[L].SPTGQATTPATTGPPSQPA.[N]	1766.85553
2962	[A].ADLDVGVNGQIEYVFGA.[A]	1766.85956
2963	[W].FPAPSPWPGPSGGWLSQ.[G]	1767.84893
2964	[P].PKDGSAPGPGEGALLSNGGS.[G]	1767.85078
2965	[G].SSPHLTGPTGHRHSAPE.[Q]	1767.85212
2966	[V].AAMTAKGQGQVSASTISSG.[V]	1767.85415
2967	[E].PVDGLAGSAAGPGAESRAGGA.[A]	1767.86201
2968	[L].QRNMTPQGLQVMVEH.[L]	1767.86288
2969	[G].GPPGEPGLPGIPGPMGPPGA.[I]	1767.87343
2970	[S].PGGAGGTVLGEAPDVLNML.[G]	1767.89456
2971	[K].GPALWEAGSPVAFYASF.[S]	1769.85335
2972	[R].VNINNGMPPGRTGMVTP.[V]	1770.86255
2973	[D].AALCKPGGPGGPDSFAVLG.[N]	1770.88433
2974	[A].AAEAAAQAAATEEAQALAI.[Q]	1770.88683
2975	[D].PSGPGIGSSGTCEAQVAVV.[T]	1772.84834
2976	[P].TAPPTYPPYASQTGGPAP.[P]	1772.84899
2977	[P].PGSGTPSRGGRSGSNWGR.[G]	1772.85352
2978	[N].NGIGAPTSGAGPAGPYTRE.[A]	1772.8562
2979	[P].AGPAPYSPPGPGPAPPAAMA.[L]	1773.86287
2980	[R].QVGEAVGGSGSLEWLDLG.[G]	1773.86537
2981	[G].NGLSTPPGPGGGPHPPHTP.[S]	1773.86671
2982	[A].PPSSPGGQAPLGPPSAGAAAS.[D]	1773.8766
2983	[N].QVGEAPSAVPEVHSQAPA.[H]	1773.8766
2984	[Q].KYSSRSNRGEVVTSFG.[S]	1773.88784
2985	[V].SPSAASPLAAAPTAPAPEAP.[Q]	1773.90175
2986	[H].NPGPPGLHAPNLGGPPGPQ.[L]	1773.90309
2987	[P].KNVSATNATMTWKVHS.[I]	1774.89048
2988	[S].SAHRVCAGAHSTSARAH.[K]	1775.84666
2989	[P].PGSGPGPGPATGAKTEPGSGP.[R]	1775.85587

2990	[A].KPGAQGAAAGPGDVGPGSGAGP.[V]	1775.8671
2991	[K].GPSPQANKELGNFFRN.[L]	1775.88236
2992	[A].AATAAPGQTPASAPAPAQTP.[A]	1775.89225
2993	[P].GPSGPPAAPSPVFLGLRR.[G]	1775.99151
2994	[G].VSAAGGGPAGAAGGAAGGGPAAGPA.[D]	1776.86235
2995	[L].GASVPGKEEGGPGPVGAPDT.[R]	1778.85553
2996	[H].QPTAVTVPGSALELCHV.[L]	1778.91055
2997	[Q].PGSPGLSGQPGLPGPPGLHG.[F]	1778.91841
2998	[T].KPLPVPPPVGKDGQVGHG.[L]	1778.99118
2999	[P].GWPGTPGAPGPKGDPGFQG.[M]	1779.84491
3000	[W].GPPSSLMSEIADLTFNT.[V]	1779.84694
3001	[R].PGGGGGGTRGANGGRVPGNGAG.[L]	1779.85933
3002	[S].GPGSGGKMALNSPQPGPVE.[S]	1779.86941
3003	[A].TADGSKTSRASVDTTPSV.[I]	1779.87191
3004	[L].HTVSASAGAASRDREPAP.[R]	1779.87325
3005	[S].PGTKDASAAPATSFTSLSA.[K]	1779.87593
3006	[P].PGSAVAGPSSSLAPSATEPP.[S]	1779.87593
3007	[P].QRTQPPGASHVASSRNS.[V]	1779.88448
3008	[S].TTASATTTATAAALGEVED.[E]	1780.84469
3009	[V].SAAPGSAAPAAGSAPAAAEEK.[K]	1781.86643
3010	[V].PGSLMVSGLTEAFVMVQ.[S]	1781.88122
3011	[D].GPSTRAGGQAEPDQEEGP.[L]	1782.78891
3012	[L].QPSEADDAETLDELHI.[E]	1782.80283
3013	[F].PGSPGEKGEKGSTGIPGMP.[G]	1782.86907
3014	[E].RDSSLPAAGAHPAPAVEH.[S]	1782.88817
3015	[K].SPEARGGGGRGWADPRTG.[R]	1783.85827
3016	[V].PGSQSVGVQGEAACVQIP.[H]	1783.86432
3017	[K].QHGVNVSVNASATPFQQ.[P]	1783.87219
3018	[P].GAKGEQGPAGHPGEAGLPGP.[S]	1783.87219
3019	[G].YTPYVSHVGLQQHTGP.[A]	1783.87621
3020	[G].SAAPAAGSAPAAAEEKKEE.[K]	1784.8661
3021	[A].SPAAASAVPGSGAAAGALASGGS.[K]	1784.87733
3022	[P].PGPQGPKGDKGDPGVPGAPG.[I]	1784.89259
3023	[P].GTPKANGSQPPGAGSPPPAP.[-]	1784.89259

3024	[R].ARGGNAAGGPAPRVLVKPP.[T]	1785.02421
3025	[P].WNKALCSQALSEAGPPG.[R]	1785.85884
3026	[T].LQAAEGEAAAAAGAGAGETAV.[K]	1785.86135
3027	[P].KGEPGHPGTDGAAGQRGPP.[G]	1785.86268
3028	[V].PGSPGFPGVPGSPGIMGFQ.[G]	1785.86287
3029	[S].GPRGQGTASPGSVSDLAQT.[V]	1785.87258
3030	[L].QEAPASLAGSAALGTFHGT.[L]	1785.8766
3031	[Y].KNYFTAGAHWLTMVF.[L]	1785.87812
3032	[Q].GTRQGPEPGLSGGPAGGHR.[N]	1787.88957
3033	[M].PGEPGVKGDTGAQGLPGPPG.[E]	1787.89225
3034	[G].VGGGVEATGPILMSPHLH.[P]	1787.91088
3035	[F].PPGPYATPPGYGAAFSAAP.[V]	1788.85916
3036	[L].QGPAGTSVAGPQTSEAFAI.[T]	1788.87627
3037	[A].QPPQPANPPHGAHPLSSG.[P]	1788.87761
3038	[P].PAGPAAPSSAPASSSPAAPAGA.[L]	1789.87152
3039	[L].REASSPPPASLPWPGPGS.[P]	1789.88677
3040	[E].GAEDRALGAQTSVGSRSE.[G]	1790.86274
3041	[P].PGEIQGPASPAGGNQSPRA.[R]	1790.878
3042	[G].PPGGSPGRSSPAGGSPGKPGS.[T]	1790.878
3043	[G].NATGGPLPASAAASQAHQAA.[H]	1790.878
3044	[L].PQGVVMAASPGSLHSPQQ.[L]	1790.88539
3045	[P].APGTSAGLGQGQGQLVGMY.[Q]	1791.86941
3046	[L].QAPAASAGGVQLPRVQLE.[V]	1791.97117
3047	[P].EPAAAPTSPATAGSPATAAGP.[A]	1792.87118
3048	[P].GRSDPGVPGAAGGEGPVELA.[H]	1792.88242
3049	[G].PGAARDSQAEPAQPEQAA.[E]	1793.84128
3050	[I].ASSAALNSAASAAAGMTVGSV.[S]	1793.8698
3051	[A].AGLEEASAAVAVGAGGAPAGPA.[V]	1793.90282
3052	[P].AAQAEVLSGDGQPDEVLP.[A]	1795.87085
3053	[N].PGIGNVSASSPAQQGLGGQA.[Q]	1795.89332
3054	[P].PGPPGGPGPDAPGLPLKKGS.[P]	1795.97011
3055	[P].MLPSQGAPQRLLSTLSP.[A]	1795.97348
3056	[E].PGGFPDIQPAGGGLSEGALG.[P]	1796.88135
3057	[L].GQAQAQAAAATTATGTQPPG.[K]	1797.87258

3058	[Q].QGQAAGGRGPHGGPSQPAAP.[R]	1797.87392
3059	[T].GLDAGGLGPAGNAASTAGPFP.[F]	1797.8766
3060	[A].GPAGAFPGALDSGARTAGQP.[E]	1797.88784
3061	[V].SWISSIMLAVMYGGGPI.[S]	1797.89139
3062	[A].KGEQGPAGHPGEAGLPGPSG.[N]	1799.8671
3063	[P].GPKGDDGIPGQPGLSGPPGP.[K]	1799.89225
3064	[A].GLNTHTSAVPPTAMKQF.[Q]	1799.91088
3065	[L].RCPPAELPWAPRRGH.[R]	1799.92345
3066	[T].GPQGNNLPSPGAGSWPPPA.[F]	1800.86637
3067	[K].GPVGDSHLGEIWVNSPH.[T]	1800.86637
3068	[P].GHSLGPTSTVSGTSEDLR.[P]	1800.87225
3069	[D].AAEPKEPAPPNGSAAEPPA.[T]	1800.87627
3070	[I].PGGPTISYTFRSVGTFN.[I]	1800.89152
3071	[L].QPSPGTTLGPPAASTPAGPP.[S]	1800.91265
3072	[K].FTVDTISAGQGDVMVFV.[E]	1801.86768
3073	[L].HGAAPFPTGPAGFPPLMH.[H]	1801.88427
3074	[G].VGEPGPLGGGGAGGPQMGLPP.[P]	1801.89014
3075	[L].PAGNAQQRTSGPAPAPPQG.[A]	1801.89398
3076	[R].AGMRARPAAAPDPAAAGGPP.[P]	1801.91261
3077	[T].AAQPRASASALQHTAQPP.[Q]	1801.93037
3078	[G].IETGGPGGPGAPGGGLPQVAL.[K]	1801.94429
3079	[M].PGGSTPVSSANMMDFFSA.[N]	1802.7724
3080	[P].SPGAMLGPSPGPSPGSAHSI.[M]	1803.86941
3081	[A].AAQPSTPAGTPRSGGGHSPA.[Q]	1803.87325
3082	[P].QPPGSAVSGPSVGQGPPDAV.[R]	1803.88717
3083	[E].PALAQPDSTAPGGATGQAPP.[S]	1803.88717
3084	[D].PGIAGSPGLPGPVGPAGAKGM.[P]	1803.94218
3085	[L].AGPNGERPLSSTGPSQHL.[Q]	1804.89365
3086	[N].PPMPGALGASGSGGHELSAL.[G]	1805.88506
3087	[P].AMQPLPAQPPLPAPPQH.[T]	1805.9367
3088	[D].PWTPPRSSTSSREALH.[T]	1808.90382
3089	[R].KQPSSTPGNATGGPLPASAA.[A]	1808.91372
3090	[D].QVHTASGTAPGTLKTLNL.[D]	1808.98649
3091	[R].VGAQSPSEGTLDDDGSFQ.[T]	1809.77734

3092	[R].PGPSPGPGPSPGAMLGPSPGP.[S]	1810.87925
3093	[G].PEKGGGSAAAAAAAAASGGGVSP.[D]	1811.88823
3094	[S].GPEGPAVPGWRHLVLAR.[S]	1812.00275
3095	[S].ASVSTGSRAGGAAGVGGEAGPP.[P]	1812.88348
3096	[V].AGARGGAEAASAGATGAAKGGPG.[R]	1812.89471
3097	[V].GPSGPPGPPGFPGDRGLPGP.[A]	1812.90276
3098	[A].PNPPASAEISASASVDLTS.[S]	1813.88141
3099	[Q].PSGAAKDAAATAGPPSITSSG.[A]	1813.89265
3100	[K].GLNGPTGPPGSPGPRGNAGGP.[G]	1813.89398
3101	[G].LNGPTGPPGSPGPRGNAGGPG.[L]	1813.89398
3102	[Q].VNVEMDAAPGVDLTRVL.[S]	1814.93167
3103	[D].VNVEMDAAPGVDLSRIL.[N]	1814.93167
3104	[Q].TGSPAPASPPAAHWPVCL.[R]	1815.88467
3105	[D].GARAAGHAGHGPAHGGLAGHG.[A]	1815.88582
3106	[L].AAPAAEAASAAPDPPAAGAAPA.[A]	1815.88717
3107	[S].PATPTPSPQNTAPSQPQP.[M]	1815.88717
3108	[Y].RNRQDAGAAASAAVEELS.[E]	1815.89438
3109	[T].AVTSESKSGFSLGTTDTK.[S]	1815.89706
3110	[P].GPMVAVTTAAPASPAVSMAA.[A]	1815.89793
3111	[K].GSPGFPGIPGPPGQPGPRGS.[M]	1815.91366
3112	[P].SPPGSPTRSPLAASPEPAP.[A]	1815.92355
3113	[G].AGRGAPEGPGPSGGAQGGSIH.[S]	1816.8685
3114	[E].TTHLAATGSGPTVAETTTT.[F]	1816.89231
3115	[A].GEAGGGVLQGPPGHVLWGE.[A]	1816.89767
3116	[E].KQGFHSGTPAASFNLPSA.[L]	1816.89767
3117	[L].PTSGAAAAAAAAAAAAVTAASSS.[Y]	1816.90355
3118	[Q].PSPGTTLGPPAASTPAGPPSG.[G]	1816.90757
3119	[I].QPQPSPHHVSPQTGSPH.[P]	1817.86777
3120	[S].APASSSPAAPAGALDRHQD.[S]	1818.87291
3121	[A].EAGAPSAVPEAATPAPSAAGP.[P]	1818.88683
3122	[A].PGPSGPGGGARSGGGRPAAANA.[A]	1818.89538
3123	[V].TAAGPQKSGSQGSVMATLQ.[L]	1818.90144
3124	[S].TPGAQAAPSTARSPQDPAP.[P]	1819.89332
3125	[S].QGPLMGLNPRGMQGPPGP.[R]	1819.89418

3126	[V].ASAQRPPASSSRASFVSGG.[D]	1819.90455
3127	[L].GQDPQALTPSGPQGANSVP.[E]	1820.87733
3128	[D].GGAGGNGLVGPGGSGAGPGGGLTP.[T]	1820.88856
3129	[A].IMTSLFGAAGAGLTGYKM.[K]	1820.89212
3130	[R].VSPGAGPTEPPLPEAFAPS.[A]	1820.90651
3131	[S].DSPDEEASICASKRPC.[T]	1821.77419
3132	[A].ARGGEAAAEVTGWPAGAPGP.[C]	1821.88784
3133	[S].GSPRTQGRGGPASVPSASPG.[T]	1822.91545
3134	[G].AASASPAPRESREPRGPS.[V]	1822.91545
3135	[D].GSPGANGIPGTPGIPGRDGF.[K]	1823.90349
3136	[I].GAFSLPNQPSPRLMSSH.[L]	1825.90138
3137	[T].NPATGKGAGGGPTAGSGTGLPT.[D]	1825.90388
3138	[E].GVAGPEKGGGSAAAAAAAAASGGA.[G]	1826.89913
3139	[P].PGADPARGAAGGGRFDRQA.[S]	1826.90047
3140	[P].PQGGGKPPNSAQTAEIFQ.[A]	1826.90315
3141	[S].RGAGAGGRSAGGGPPDSSLVT.[Y]	1826.91036
3142	[G].PGGRESQPPRSPAEAPPP.[P]	1826.91438
3143	[G].PSGGGYDFGFDGDFYRA.[D]	1827.7609
3144	[G].GPAGAAGGAAGGGPAAGPADHGLA.[G]	1827.87325
3145	[S].YRRMFGGPGTASRPSST.[R]	1827.89188
3146	[K].AGPAFGGGVTQQGITSSHVG.[A]	1827.8984
3147	[Y].NRAGTGPASPSANATTMKP.[P]	1828.89702
3148	[Q].GPPGKMGPQGTPGIPGMPGP.[I]	1829.90369
3149	[E].TGPQGNNLPSPGAGSWPPP.[A]	1830.87694
3150	[P].QVYTSGKGSSAAGLTASVM.[R]	1830.8902
3151	[G].ASSAPQVAAAYTFYALAE.[D]	1830.89086
3152	[V].GASTPAAGGPQEVTYAQLN.[H]	1831.88208
3153	[S].SPRAMPPSPGPTERHAQ.[P]	1831.88679
3154	[P].GPSGPATQPPVSSATMHLP.[L]	1831.90071
3155	[P].PGPSGPATQPPVSSATMHL.[P]	1831.90071
3156	[G].LVGPCGVGGAAAGSSTGVMAL.[R]	1831.90408
3157	[A].PGGAAATANAVAHGLNGGSPAA.[A]	1831.90455
3158	[L].GPREVGGQGAGSAGGLEPVH.[P]	1831.90455
3159	[A].PTQSPELTPSGPAPAGPASA.[P]	1832.90248

3160	[S].GVSAAGGGPAGAAGGAAGGGPAAGPA.[D]	1833.88381
3161	[S].GAGPGGGLTPTAPPYGAGKHA.[P]	1833.92422
3162	[D].ENGLWAVYATNQNAGNI.[V]	1834.87185
3163	[G].EEAAAVAPAAGAPAPAGDTTP.[G]	1834.88175
3164	[P].PPGLSSSGVSAASQGAGGGPPP.[A]	1834.89298
3165	[V].TAAGPQKSGSQGSVMATLQ.[L]	1834.89635
3166	[P].PGEPGPPGPPGPPGVPGSDGI.[D]	1834.897
3167	[P].GPSPAGEVLMVEVENVAH.[E]	1834.90037
3168	[G].NASGLGAGPGPSVGMGVVPDP.[F]	1835.89562
3169	[L].NPAFFPPPNATVGPPPDT.[Y]	1835.89628
3170	[G].GPSVGPADQTVPPGSKQES.[P]	1837.89265
3171	[V].NVSNLAGFGGGGGAQPLHQG.[A]	1837.89398
3172	[L].VFQNLNPNTLYEVSVT.[A]	1837.93305
3173	[Q].QAPSTELVSGGSDNQVIH.[W]	1838.8879
3174	[L].RSSGASPAGTPSLADSGAPPG.[Q]	1839.88314
3175	[A].KREDSPGPEVQPMDKQ.[F]	1840.88579
3176	[G].PSPGPGPSPGAMLGPSPGPSP.[G]	1840.88981
3177	[P].PPSGPPSATHTPFHQSPV.[E]	1840.89767
3178	[P].LAASGMAPGPFAGPQAQQAA.[R]	1840.90104
3179	[T].PAASSIWSPASISPGSAPAS.[V]	1840.90757
3180	[S].PGGAGGTVLGEAPDVLNMLG.[A]	1840.91094
3181	[W].PGGEPPAAQEDLAGREFT.[C]	1841.86643
3182	[V].PGSPGFPGVPGSPGIMGFQG.[F]	1842.88433
3183	[A].CALLTEGGAVGSEIAGGAGPG.[W]	1843.88545
3184	[A].AAGGGGATAAAARGGEAAAEVTG.[W]	1843.88929
3185	[E].PGAPCVTPRSGSALARCS.[S]	1843.89016
3186	[M].PPQTQSPGQPAQPAPMVP.[L]	1843.90071
3187	[S].HQAVQASGSTAIDGSFRN.[L]	1845.88381
3188	[Q].GPPGKMGPQGTPGIPGMPGP.[I]	1845.8986
3189	[R].GPPGESVVGAPGAPGTPGERG.[E]	1845.90897
3190	[H].QPDKDAPVATAVLHLTGI.[E]	1846.00689
3191	[A].VNGQAPGGPTGLGPAPLASW.[D]	1846.94462
3192	[W].GEPSVPPAAAFQPGHKRT.[P]	1846.95586
3193	[P].GNATGGPLPASAAASQAHQAA.[H]	1847.89946

3194	[S].MMKLKGMAAAARSQGQH.[K]	1847.9037
3195	[L].SFNQMTKLPSGLPVSLL.[T]	1847.99355
3196	[R].SGDPRSRPPSSVPLLLGL.[L]	1848.03377
3197	[M].PGMPGQKGQPGSPGLSGQPG.[L]	1849.88612
3198	[F].GASTPGVFGQPGFGQAPAFG.[Q]	1849.88677
3199	[P].GLPSSGQGGAASPGSSLGLYS.[P]	1849.89265
3200	[P].DLAEAVGATTAPTTATTSAT.[T]	1849.90254
3201	[A].PVAMPASSPPGPPPAPEPGP.[P]	1849.9153
3202	[H].QAAPPTSSPAASFPPPPSSG.[A]	1850.89192
3203	[H].SPPREAASGGPGSSSITFF.[A]	1851.88717
3204	[T].TILPEDGGPYTNSILFD.[S]	1851.90109
3205	[S].SYSSISTRGTPGIPANMGG.[A]	1852.88579
3206	[L].PSGNWIAGPAHTGREVGF.[P]	1852.90891
3207	[H].GPPGREGKMGRPGAEGARG.[L]	1852.91949
3208	[V].PGKDGQAGHPGQPGPKGDPG.[V]	1853.8889
3209	[C].RDPHSSSKASSVDTGTPK.[S]	1856.90969
3210	[A].AGPGGRESQPPRSPAEAPP.[P]	1857.9202
3211	[V].PGPSGLGGAGGLAGLTFPWM.[D]	1858.91563
3212	[F].PGAAAAAAASLEPPAEAEPAP.[G]	1858.91813
3213	[T].TTGTSTGLGTGLGTGLGFGGF.[N]	1858.91813
3214	[I].PPPFPPMGLPPMSQRPP.[A]	1858.93426
3215	[V].QPGVDPANATGLDGREPAP.[H]	1861.90388
3216	[G].GGGLGGAGFRGGNLGLGGFGPS.[C]	1861.93037
3217	[N].PGRMGYPGPAGPMGPPGLPG.[L]	1862.90402
3218	[N].RDSGHRAAGSAARGGTAHGA.[G]	1862.90768
3219	[S].PGPEARSAGDIPVEKLNL.[G]	1862.99705
3220	[G].NAGPGIQGGGAIVQRAIKR.[R]	1863.06714
3221	[H].QGKQNGSAWARSTTTRD.[S]	1863.90561
3222	[P].EPAAAPTSPATAGSPATAAGPA.[T]	1863.9083
3223	[V].RMPTPATAQQPPDRPQG.[A]	1863.91301
3224	[R].GPQGQPGLPGHPGPMGPPGL.[P]	1863.91703
3225	[L].QPLTSGSAGPAQPGSVAGAGPG.[P]	1863.91953
3226	[I].ERGAAAAAAGQPGTAPSGVPGA.[P]	1863.93076
3227	[Q].QADASKQLWNPPQVQGP.[L]	1863.93479

3228	[V].VGKDDIDNSKPGPGPSQPG.[P]	1864.90355
3229	[Q].GVPGTQGFPSGRHLAGPAC.[L]	1865.90753
3230	[E].AGGAERGAPATQPPGMGEVL.[L]	1865.91742
3231	[L].QGPSGSLSAPPAASVTSAPPS.[S]	1865.92395
3232	[P].AASPQRSPSPLSGHGAQAF.[P]	1865.92528
3233	[D].PAWSADVAAVVMQEGLAH.[I]	1867.90071
3234	[P].TAPPTYPPYASQTGGPAPP.[P]	1869.90175
3235	[R].PSPAPSSPASAQVSPQHQP.[H]	1869.90897
3236	[P].SGPPGKPGTGSPGPQGQPGLP.[G]	1869.94535
3237	[R].AGPVSSGPWGSSEEEGPGSP.[R]	1870.80898
3238	[P].AAEAAAAAGGGGATAAAARGGEAA.[A]	1870.90019
3239	[L].PGSPGAKGEQGPAGHPGEAGL.[P]	1870.90421
3240	[D].PAGSDGRSVPGSAGSGSGGRR.[R]	1871.90667
3241	[G].PGVSFSPGPTPTPAPTAGSF.[A]	1871.9174
3242	[R].NPPAVAMASPASAPAPAPSPA.[P]	1871.93201
3243	[Q].GPPAGPAAPSSAPASSSPAAPAG.[A]	1872.90863
3244	[P].GPPGEIQGPASPAGGNQSPR.[A]	1873.91511
3245	[T].SGPNQALPGTTSQQTVPGH.[H]	1876.91478
3246	[D].KGGGWAPHHGHPGGQAGRN.[C]	1877.90147
3247	[A].ATPGQTTAAPAASPEWPQP.[H]	1877.90282
3248	[P].GCRPLPSAGSPTPTTHEI.[V]	1877.91742
3249	[G].SLASGPSPSPPASPSQGPKE.[T]	1877.92395
3250	[E].GPSACLLRDQRPPEGQP.[S]	1877.92866
3251	[P].AAGEPGRAATAPTAGGEPLSP.[P]	1877.93518
3252	[T].GSRAGGAAGVGGEAGPPPERE.[G]	1878.90528
3253	[S].PAHRDSAYISGSPLGSHQ.[V]	1879.90455
3254	[R].ASSPQPQPSAVGHWRSST.[V]	1879.90455
3255	[H].WPQGPAGPPGPPGPMGPPGL.[P]	1879.91597
3256	[Q].GPGPGPGPAPGEVTAASAGYLG.[D]	1879.91847
3257	[S].PGAKGEQGPAGHPGEAGLPGP.[S]	1880.92495
3258	[L].GPASSRSAASPTERLEPAP.[S]	1880.94608
3259	[Y].VPMQNPVSASPVPSGTNSP.[A]	1881.9011
3260	[A].NLQGFQAGPGGQGSPPEAAV.[P]	1881.90897
3261	[Q].GPSAGAASAGPQVSLYQGAPP.[A]	1882.92937

3262	[G].GSPPMLEGAGGLGISVWTPG.[S]	1882.93676
3263	[V].QVGEGAAGTVSTLLPEEPAG.[A]	1882.93926
3264	[G].QAGPTVGPQQQPPAGAPQPG.[A]	1882.9406
3265	[P].DKPSAPAAAAAAAQPPASHGP.[E]	1882.9406
3266	[R].PSGPGPVTQGPQQPQPPSQ.[Q]	1883.92462
3267	[T].GSPGPKGSPGFPGIPGPPGQP.[G]	1884.96027
3268	[V].APAVVVKQEAVGPEPEPAP.[A]	1885.00655
3269	[T].TPPSTAPSGPGSVQKYIVV.[S]	1885.00655
3270	[E].PGTASPADLLRPVLDILH.[T]	1885.05417
3271	[G].PSSPSRTPSSHDSDTRDG.[P]	1885.82709
3272	[Q].GRDSPPPEGPGGHQSEPKG.[S]	1885.87873
3273	[H].GGAGGPGLSASEGWAPPHSPP.[P]	1885.88275
3274	[C].GVGGAGPPGHGGTAQVTHGGAGS.[A]	1885.88996
3275	[I].KGDPGPPGPMGPPGGMPGLPG.[R]	1885.89352
3276	[P].EEARPAPGTGPGAYNNTAL.[F]	1885.90388
3277	[A].PGAPAASPFAAATALGGAEATS.[S]	1885.92903
3278	[-].MQAPGGVPGVEATLYWVEA.[V]	1885.93305
3279	[A].QPSTPAGTPRSGGGHSPAQP.[P]	1886.91036
3280	[D].GARAAGHAGHGPAHGGLAGHGA.[A]	1886.92293
3281	[P].SGPPGPKGDDGIPGQPGLSGP.[P]	1886.92428
3282	[P].GADPARGAAGGGRFDRQASA.[E]	1887.91684
3283	[G].LDAGGLGPAGNAASTAGPFPF.[H]	1887.92355
3284	[P].SPQAKPSNPSNPRVFFD.[V]	1887.93479
3285	[N].IPPQMSAAAAAAAAAYGRSP.[M]	1887.93816
3286	[T].TGEEVLAQYLQQQQPR.[V]	1887.95592
3287	[P].QPGPLTATGPQPSGPPRTE.[Q]	1887.95592
3288	[Q].ALMQQQAALVAAHSAYLS.[P]	1888.95856
3289	[Q].PNVNSSKASKAGPSILMAT.[A]	1888.97969
3290	[G].PGSPGRCRTPAGEGPHTR.[V]	1889.91474
3291	[L].QGPAGTSVAGPQTSEAFAIT.[V]	1889.92395
3292	[S].WPNRTANHPAPSLENSV.[Q]	1889.92528
3293	[N].VSYMKQQAGASPSSLPSH.[A]	1890.90144
3294	[R].AVPEPNTASATAQAPPAGPGS.[G]	1890.9192
3295	[A].KDGGSPALSTTASVSVEVAD.[V]	1890.92909

3296	[V].GPGQSGGLGGVGAGPAPSWWP.[L]	1891.90857
3297	[Q].GPAGPPGPPGPMGPPGLPGPMG.[I]	1891.91934
3298	[F].GQPGGLGSNIYQHRFNF.[F]	1891.9198
3299	[G].PPGGSPGRSSPAGGSPGKPGST.[P]	1891.92568
3300	[P].SQPTGLPAAPLPSPFPMAP.[A]	1891.96225
3301	[G].GLGSSPGPASSTGQASTTSKD.[S]	1892.8832
3302	[E].SLGELEAPAQGGAPSPGEQV.[L]	1893.91886
3303	[S].TGCVLAGGVAGAGAMLLCSL.[T]	1893.9231
3304	[P].PGVAGEASGPQQVAFPSPAE.[L]	1895.91338
3305	[F].PQNSNTLTPPGAGMLGFPP.[S]	1895.93201
3306	[A].PAQGQPGPPPGAGAPPTERN.[E]	1895.93585
3307	[L].SSEPLSTPSGAPEPTAGTPD.[D]	1897.86616
3308	[P].PQGGGKPPNSAQTAEIFQA.[L]	1897.94027
3309	[S].GAQSGTPPTGLYGHSAVYH.[E]	1899.8984
3310	[A].QPAAEAAAPGWAQARGHPGG.[E]	1899.92087
3311	[V].QVGNAPGPLGDAASGHHTTI.[V]	1899.93076
3312	[F].QLSHQPQPSTASPHISGQ.[T]	1899.93076
3313	[F].GPGGQLIKVIPNLPSEGQP.[A]	1901.04909
3314	[P].GPGAKEEAAGVSGPAGGRGGGY.[G]	1901.91003
3315	[A].GAGPATTTTPPGPPAGHTLSN.[A]	1901.93518
3316	[E].AASGPRGPQTEAPPEGVPSP.[G]	1901.93518
3317	[A].RPSEGPTTGPTGPPAAGPTGP.[P]	1901.93518
3318	[A].SSPGPITDAAKSPSAETSAK.[S]	1901.94508
3319	[A].VGPAGAVGPRGPSGPQGIRGD.[K]	1901.99403
3320	[W].SQGVPFPHGSSWVEDCN.[S]	1902.80754
3321	[V].VGGPEAAAAATGGYGPVSGAVSG.[A]	1902.9192
3322	[T].SSPRAMPPSPGPTERHAQ.[P]	1902.9239
3323	[A].PGGAAATANAVAHGLNGGSPAAA.[M]	1902.94166
3324	[K].GPALERGGDVGYRGNQEK.[H]	1902.94166
3325	[V].GPRGNLGAGNGNLQGPRHM.[Q]	1902.94637
3326	[V].QRSGPSGPPGPKGDDGIPGQ.[P]	1903.92568
3327	[P].GPRGPEGAMGIPGMRGPPGP.[G]	1903.92655
3328	[N].AREEGVPGGGARAGEKEHP.[E]	1903.93691
3329	[K].GEPGQGLRGSQGPPGKMGPP.[G]	1903.9443

3330	[A].TAAPGQTPASAPAPAQTPAQA.[L]	1903.95083
3331	[A].GGAGPGAGIGSGSGAGGSSEPSAC.[S]	1904.80391
3332	[S].KDPGALMFPIYTYTGKS.[H]	1904.94626
3333	[V].APGQGRAGAMGSRTPGSPLH.[A]	1904.95079
3334	[A].PGTSAGLGQGQGQLVGMYQG.[A]	1905.91234
3335	[L].GTAPQTAQQAGPEAAGHRAS.[L]	1905.91618
3336	[D].GAPPKDGSAPGPGEGALLSNGG.[S]	1905.93009
3337	[P].PGLSSSGVSAASQGAGGGPPPAP.[P]	1905.93009
3338	[A].AVEAVEQGVPEKEETPPP.[E]	1905.94401
3339	[T].SPRPAESASAGATLRPLLL.[P]	1907.07089
3340	[R].PEEVKSGSTAQPGSEEME.[M]	1907.81749
3341	[E].KPDNSAAGTVPSGQKQHDA.[P]	1907.92059
3342	[N].QVGEAPSAVPEVHSQAPAH.[A]	1910.93551
3343	[A].TPASVSSPAGSPGPPGSTASLS.[T]	1911.92943
3344	[K].PGSRETPPNPHPMALGSPA.[Q]	1912.93341
3345	[L].GPPAASTPAGPPSGGASPTPPAA.[S]	1912.93993
3346	[G].GAGPAGAGGAAAVATGPQALFSGG.[A]	1912.95116
3347	[T].QQPSTPQTPQPPAQPQPS.[P]	1913.93518
3348	[C].AQPAAEAAAPGWAQARGHPG.[G]	1913.93652
3349	[V].LGQSYIFPSAISAMEATI.[T]	1914.95174
3350	[L].GGPTTPASGPAPAPAEPAGSEP.[G]	1915.90321
3351	[G].LPGSEGPPGPAGSAGPPGYPGP.[R]	1915.91847
3352	[Q].SFGGPPVSQPNHVSSPPPQ.[A]	1915.9297
3353	[L].GGPLTQMPPQTASSHPAAPA.[T]	1915.93307
3354	[A].GPGERLDQAQFPPEAAHP.[Q]	1916.92495
3355	[P].GPGPSPTTVPGPASGKPSTEP.[P]	1917.95525
3356	[K].ETPTAATPGPPGTTRSSGHP.[S]	1918.92534
3357	[V].PRSSTPSHGQTTAPEPTPA.[Q]	1918.92534
3358	[H].QQQVYPSPVSAAAEETTL.[E]	1918.93926
3359	[L].PSPFPMAPARPAQHATTAA.[R]	1918.95923
3360	[P].APGTSAGLGQGQGQLVGMYQ.[G]	1919.92799
3361	[D].GPSAVAQAEHPASAMPGISAA.[A]	1919.92799
3362	[K].KGGGAAGGGGVASGGAGGPQPPQQ.[Q]	1919.93183
3363	[K].PGPLSWDPASVTPEPVSSP.[T]	1919.93853

3364	[L].APNTRGSPGPPPAKPCSGTA.[P]	1919.93922
3365	[P].PQREGAQPTTGREREAH.[G]	1919.94306
3366	[A].GPGAGIGSGSGAGGSSEPSACSD.[H]	1921.78284
3367	[P].TGQATTPATTGPPSQPANPQ.[E]	1921.92501
3368	[Q].KTSAYTNWAVSASDPNPL.[G]	1921.92903
3369	[V].WVEAAPAALELVCVASVH.[S]	1921.98404
3370	[R].GPAEEQPRPHTAAPSPGGPA.[R]	1923.93076
3371	[P].GPLSWDPASVTPEPVSSPT.[T]	1923.93345
3372	[E].PGRAAASGAAASSADPTALGGPA.[G]	1923.95189
3373	[G].RQGSGPSPQTGGVRVTVQL.[L]	1924.0359
3374	[G].PQGQMGPQGPPLHQGGGGPQ.[G]	1924.90825
3375	[E].KYYMKNGVDLGPICGPP.[N]	1924.92957
3376	[I].MRLHGGRDGAGRLCPAQG.[D]	1924.93409
3377	[-].MAAQGAAAAVAAATSGVAGEGQPG.[P]	1924.93591
3378	[R].QQGQAAGGRGPHGGPSQPAAP.[R]	1925.93249
3379	[S].SRLSHSSGYAQLNTYSR.[A]	1926.94166
3380	[P].GPSGPPGKPGTGSPGPQGQPGL.[P]	1926.96681
3381	[V].TVVEPDTPPSPDTPPAATH.[R]	1928.92361
3382	[K].PGSRETPPNPHPMALGSPA.[Q]	1928.92832
3383	[P].GPGVSFSPGPTPTPAPTAGSF.[A]	1928.93887
3384	[A].AASSPATAPAPAPAPASASAPAP.[V]	1928.97123
3385	[P].AAQPPQPANPPHGAHPLSSG.[P]	1930.95183
3386	[A].SPLGAGAAGSQGPAGGPAPDTPV.[P]	1931.94574
3387	[T].PASSFVSPPPPTASPHSNR.[T]	1932.95625
3388	[G].ARGPCPMVAPGPEGPSTPR.[S]	1933.93711
3389	[L].FSEKFPTLWSGARSTYG.[V]	1933.94429
3390	[G].MPGTLGPVGSPSGAASPASHR.[V]	1933.95487
3391	[P].GPSPLSATQGATPQQPPVNS.[L]	1933.96139
3392	[D].PSGWGARSPPQKMYMLP.[Y]	1934.92515
3393	[L].QITAAHSGEALALDSNHLS.[R]	1934.95664
3394	[V].AAAAGLEEASAAVAVGAGGAPAGP.[A]	1935.97704
3395	[Q].PGSTAAPTPYGAYNGPVPGY.[Q]	1936.90757
3396	[W].GPGRGSPQAQGTGSGLPCVPG.[S]	1936.92938
3397	[A].AAAATTTSAAATSAPPALDMF.[G]	1936.93207

3398	[E].AAGAGPAASSPQRGRMEVSH.[S]	1936.94062
3399	[D].YRPHGGAGDADFYYRGH.[I]	1938.86302
3400	[L].RYSGASCGSSFPSNLVYS.[A]	1938.86505
3401	[Q].GGAASREVGGPQAGGARSPGEA.[D]	1938.93764
3402	[E].QPPSQASTVSPTSATSASVH.[L]	1939.93557
3403	[L].QPTAHSPAGNQVQAGKQSH.[I]	1942.94781
3404	[V].SATGPGPSPGPAPPNYSRPH.[G]	1943.93585
3405	[A].GIGGEPAAAGAGCSPRPKYQ.[A]	1943.93922
3406	[R].QGPPAGPAAPSSAPASSSPAAPA.[G]	1943.94574
3407	[A].TPSAGHLSSSSIYPVCLAP.[Y]	1943.95314
3408	[S].SPPSITHSPQNELKGTNH.[S]	1943.95698
3409	[P].VSPESAPSAVSQPSSPHSPP.[S]	1944.92976
3410	[R].RPSVEGPGSDVGFLQAQNS.[T]	1944.94099
3411	[F].SVPADYAPPPAAFPPREY.[W]	1944.94904
3412	[D].GQKGAEGPPGPTGQAGEPGPR.[G]	1944.95223
3413	[L].YHPGALTGTPPSLPPGPSAQ.[S]	1944.9814
3414	[G].GPAPGTSAGLGQGQGQLVGMY.[Q]	1945.94364
3415	[G].PGASASTAATPGPAGLPRGYM.[A]	1945.94364
3416	[S].QGSQGPAGPPGPPGPPGPPGPSG.[G]	1946.93551
3417	[P].GPGSPGRCRTPAGEGPHTR.[V]	1946.9362
3418	[P].PGEQQPVFWAPKWVDY.[S]	1946.94356
3419	[Q].PGLPSSGQGGAASPGSSLGLYS.[P]	1946.94541
3420	[G].GPGPERTPGSGTGSGLQAPGPA.[L]	1947.95189
3421	[L].DWLRSGGGAVGGEKDPPPPG.[T]	1948.95116
3422	[G].PGANGMPLAGLAWSSASAPPP.[R]	1948.95856
3423	[E].PSGRDAQGRASTPAPPAWE.[R]	1950.94166
3424	[G].PGAARGSPASSSPENLVDQI.[L]	1952.96721
3425	[A].GPQAQQAAREVNTASLCR.[I]	1956.96683
3426	[L].SNLHISSPVINGLIDAGHL.[K]	1957.05015
3427	[E].DQGQRPKLSPNKQPVPPA.[Q]	1957.06138
3428	[L].ELLNQRGLRDPGGPLQPP.[P]	1957.06138
3429	[T].PAAEGVGAAANAAATSSTGTGGVA.[A]	1958.94139
3430	[P].GPRGPEGAMGIPGMRGPPGPG.[P]	1960.94801
3431	[A].QPGPSHPPPNPASPRAEAPG.[S]	1960.9624

3432	[P].GPSGPATQPPVSSATMHLPL.[V]	1960.97969
3433	[V].QQQAQQQVAQPAPASQQQ.[P]	1963.95804
3434	[F].RAPGETSAQGHIPGEARTE.[P]	1963.95804
3435	[R].SPPGSAVAGPSSSLAPSATEPP.[S]	1963.96073
3436	[F].IEAQPEPAGAPTDTLPATGQ.[P]	1963.96073
3437	[E].TTHLAATGSGPTVAETTTTF.[N]	1963.96073
3438	[L].AATPYPAERAPPGKAADPSP.[L]	1963.98722
3439	[W].WGPLGQGAPGSNPIRAYHS.[G]	1964.97257
3440	[S].RGSFSHGGGGLGSGVSTGFLE.[N]	1965.94133
3441	[A].ATAPNAGASAPEAAASAEAPLQ.[Y]	1965.95122
3442	[L].PGSPGAKGEQGPAGHPGEAGLP.[G]	1967.95698
3443	[I].SAQPAHHPPQSPAQIQMQ.[L]	1968.93447
3444	[E].AAAGDAVAAASATAAVEPTELD.[F]	1971.95056
3445	[A].APQPPGSAVSGPSVGQGPPDAV.[R]	1971.97704
3446	[Q].AGLGSPGGWKSVSSAPERSAA.[E]	1971.98828
3447	[C].VSHLTSITIFYATIIFM.[Y]	1973.04525
3448	[P].GPSPGAMLGPSPGPSPGSAHSI.[M]	1973.93855
3449	[P].WAAEAAAAAAAVSGSAAAEAKE.[C]	1973.95631
3450	[L].QGPPGKMGPQGTPGIPGMPGP.[I]	1973.95718
3451	[F].STEPLKNNGRGSPLGFYH.[V]	1973.9828
3452	[S].PGAGAAGISPGHSPLQGLINC.[L]	1973.98617
3453	[A].AATAAPGQTPASAPAPAQTPAQ.[A]	1974.98794
3454	[A].TEFRPAPLQGVLPGLLGPL.[R]	1975.13751
3455	[R].GAARGQETPDPPGSTSLSFT.[V]	1975.93557
3456	[G].MLPRPQDAGGVSGQLGHQQ.[A]	1975.97667
3457	[P].PQGVGYPAPQQQPPPRMQ.[H]	1975.98069
3458	[V].QAPGIMGAIAKESSIQTHH.[T]	1976.00182
3459	[S].QSSPGLNPGQPSSMLSPRH.[R]	1976.96068
3460	[P].TARPSEGPTTGPTGPPAAGPTG.[P]	1976.96721
3461	[P].PRSLDMAIEAPRFESIM.[E]	1978.97249
3462	[R].LAECVSVAPVAVESPPEPGA.[A]	1978.97902
3463	[T].QQGVPGSPTVNPSSIGNKDP.[Q]	1978.98286
3464	[H].NQFQPPQGVGYPAPQQQP.[P]	1980.95625
3465	[L].FREANLEPGYPQPLTSY.[G]	1981.96542

3466	[N].QVGEAPSAVPEVHSQAPAHA.[T]	1981.97263
3467	[L].KKRVRAGLPGGGPGPGGGHRA.[G]	1982.12672
3468	[L].GPGQGTPSLGPGSTPTTIKTE.[V]	1983.00293
3469	[R].ARGGNAAGGPAPRVLVKPPTP.[G]	1983.12465
3470	[R].WLGEGPAPHPFASLPFGFG.[K]	1983.97519
3471	[R].KGGGSAASYRTPSKGAGAAFGS.[R]	1984.98353
3472	[Q].PGAAGAGAAPALDFTVENVEK.[A]	1984.99745
3473	[N].GPPTMKTSPTVYPVDDLPA.[R]	1985.98885
3474	[L].ASTSAPPGPPAAASPCLGPAAAA.[G]	1989.96985
3475	[A].QSGFALFTDDPSGRDLEH.[C]	1991.90936
3476	[A].GGRGAPKDADAAGRPANWAPG.[R]	1991.97944
3477	[S].QGPLMGLNPRGMQGPPGPR.[E]	1991.99021
3478	[V].QPGAGQAGVVQPGAGQPVVMQ.[P]	1991.99673
3479	[S].GPVSSPQINSTVSLPGGGSGPP.[E]	1992.00326
3480	[S].NIHFSLDEKSGRDGQKY.[P]	1993.97263
3481	[S].QDPPPPHSGALPFPSAGPPQ.[P]	1993.97665
3482	[V].SAGPRSGGGRNATTAMPPPVP.[N]	1993.98723
3483	[P].LGDQGQAQAKMGACYRALG.[Q]	1994.95349
3484	[S].QGRGNSLRNISSATHGPRS.[S]	1995.02271
3485	[T].AGLGDPEKQSMVSSIDKSF.[G]	1995.96918
3486	[-].MKGDKPEEPGQGPEPSGPPPP.[T]	1996.96106
3487	[M].KGDKPEEPGQGPEPSGPPPP.[T]	1996.96106
3488	[S].APNPSPGRRLQDPASSPLH.[Q]	1997.03115
3489	[G].PGKSGSMGPAGPPGPAGERGHP.[G]	1997.96102
3490	[P].PGQGIFSGPGRGERFPNPQ.[G]	1997.99403
3491	[S].GAGSTTSGVVSGSLGSREINY.[I]	1998.97269
3492	[G].AGVPAFYTPTAYGTLVQEGG.[A]	1998.98073
3493	[S].TGGPPGPGPPPGPPGLSSSSGSR.[E]	1999.98319
3494	[Q].GPGASASTAATPGPAGLPRGYM.[A]	2002.9651
3495	[S].FLPQPPQEKAHDGGRYY.[R]	2002.97699
3496	[M].QGPPHPHGIQGGPGSQGIQGP.[V]	2002.9842
3497	[G].PGSPQGKEGTTTQDYVPDK.[P]	2004.95089
3498	[T].GVQAQPGEATSGPPGIQPGQE.[P]	2004.96212
3499	[V].GPGPSPAGEVLMVEVENVAH.[E]	2004.96952

3500	[A].GPELAGHSPSEASVFPQGAAP.[G]	2005.96139
3501	[R].HATPGPSPTTPPQPPDPSQP.[P]	2005.96139
3502	[R].PPPGAGPDPPSPPGADPARGAAG.[G]	2005.97263
3503	[K].PGEGQRSSPAVGSPGNRQGPA.[G]	2005.97984
3504	[A].GPRSGGGRNATTAMPPPVPNG.[N]	2006.98248
3505	[G].GPGAGGLEGPPQKRGIVEQC.[C]	2007.00763
3506	[V].AAAAGLEEASAAVAVGAGGAPAGPA.[V]	2007.01416
3507	[T].GPGGAGGGGNRVELLVFGYAC.[K]	2007.97052
3508	[L].RAQDPSEVLTMLTNETGF.[E]	2008.96443
3509	[K].PSATPPPGASGAPPHAGLGGWQ.[S]	2009.9828
3510	[M].PGQKGQPGSPGLSGQPGLPGPP.[G]	2010.04031
3511	[P].GPATGLEAASGQGPSSAAAQTGK.[D]	2013.98359
3512	[I].FPTEQYILLNGTVLRYS.[G]	2014.0644
3513	[G].GPPQNSMMMAPGAPDSLNAP.[C]	2014.86671
3514	[P].SPGQQELPPFQPGQPSPPQ.[G]	2015.98213
3515	[S].PGSAPSAASPATPSPPARLRK.[T]	2016.0985
3516	[C].SQAAAQPSTPAGTPRSGGGHSP.[A]	2018.96385
3517	[G].GLSGHRWQSETLEEPRH.[R]	2018.97911
3518	[G].PGGQPSAFPPARSPGGSDPQI.[P]	2019.98828
3519	[G].TVATDFFTVLSTGQRFTE.[D]	2020.0022
3520	[A].SGPPPASTGTSAWGEPSVPPAA.[A]	2020.96106
3521	[E].SVPSSGPTNVSVLATTSSSML.[V]	2022.00596
3522	[E].VSGGPGHPTPPGHASVWSPAAG.[L]	2022.97805
3523	[G].NPPSRAHSVSASVSPIQSSD.[P]	2022.98392
3524	[S].PGAKGEQGPAGHPGEAGLPGPSG.[N]	2024.97844
3525	[L].PGSPGAKGEQGPAGHPGEAGLPG.[P]	2024.97844
3526	[A].PGTPGQPGIPGMKGHSGAPGPAG.[L]	2024.99707
3527	[F].NSPNLKDGRFVNPSGQPTP.[Y]	2025.01483
3528	[E].PGSPGQPGEPGARGEPGDPGLP.[G]	2025.96246
3529	[K].RQEVTPWMPAALDQQQQ.[A]	2025.98108
3530	[L].GRPEPGPSQSPRAQGPSHSP.[E]	2025.98492
3531	[Q].CRVLGGGGPGGGGGLGGPGGSVPF.[K]	2025.99232
3532	[G].AAGPGGRESQPPRSPAEAPPP.[P]	2026.01008
3533	[Q].PGSPGPAGVPGLPGAKGDHGFPG.[S]	2026.0141

3534	[C].GLGAGSNFSGPLGSVSSRGSFS.[H]	2027.97811
3535	[L].PSGGEAILEGHSVAQEPAAAH.[C]	2027.97811
3536	[K].NAEVLYSIESGNIGNSFTI.[D]	2027.99203
3537	[A].AAAAGGAGPGAGIGSGSGAGGSSEPSA.[C]	2028.92171
3538	[R].AYFTLGAGGPGWEPAVESGY.[G]	2028.93378
3539	[I].RQQGPLPEEPYSPPPGSPP.[P]	2029.99778
3540	[P].PKAPAVPDCAPSLEAGNPAPA.[A]	2030.00115
3541	[P].GPGPGPGPGPGPGPGPGHSMRLP.[V]	2030.00249
3542	[T].YHLQPSALESTSESLSWP.[G]	2031.96581
3543	[V].RGDEFLGGSVSLTAPGSMGPP.[D]	2031.98042
3544	[P].GAPGFSGPKGEPGDILTFPGM.[K]	2031.98444
3545	[Q].GPPGKMGPQGTPGIPGMPGPIG.[Q]	2031.99904
3546	[E].KPEPSTQEQPEASEPSPPV.[E]	2033.96621
3547	[L].GPKGEGGLPTCGPPDKASTAH.[S]	2033.97091
3548	[A].PGENVGRANGGQGVESAVAEH.[E]	2034.95877
3549	[G].LGGPPGEPGLPGIPGPMGPPGAI.[G]	2035.06811
3550	[G].GTAGKEVSLYIVEAVGGSGGAL.[V]	2035.07061
3551	[S].QPSPAAPAPPTSTQDSVHLH.[N]	2037.99884
3552	[E].KEPEGPVEATVASGCLTRH.[A]	2038.00221
3553	[F].PGSPGEKGEKGSTGIPGMPGSP.[G]	2039.97025
3554	[P].AAAPTSPATAGSPATAAGPATATE.[E]	2039.988
3555	[V].GPSAVGGQVGQAGEGVHPHTPT.[-]	2039.98934
3556	[Q].NPSPLGMGIGWAPLMAPPHP.[G]	2040.01938
3557	[G].ESGPAPRTAERGAPGSLSPAH.[P]	2045.01589
3558	[A].TGLDAGGLGPAGNAASTAGPFPF.[H]	2045.99269
3559	[P].APGTSAGLGQGQGQLVGMYQGA.[D]	2047.98656
3560	[D].PGPGSSRAPGAGMAGQFRSYV.[W]	2049.99232
3561	[R].PGPSPGPGPSPGAMLGPSPGPSPG.[S]	2051.9855
3562	[N].NLGSSPSPTALSAAEDLIFY.[A]	2053.01243
3563	[M].AAAAYPAFQSLSLPKLYRS.[V]	2054.10694
3564	[-].MAAAAYPAFQSLSLPKLYRS.[V]	2054.10694
3565	[P].GPGVSFSPGPTPTPAPTAGSFAG.[G]	2056.99745
3566	[P].VMDVAFVQFLASVSGKVSC.[L]	2060.01911
3567	[V].PPVVDERTGSEGATASPSLGH.[H]	2063.99924

3568	[A].ATQQHSRQAAPQMLQQQP.[P]	2064.00395
3569	[L].PSGNWIAGPAHTGREVGFPN.[C]	2064.0046
3570	[A].AAAAAAAAANASASTSASSTVSGTV.[P]	2065.99963
3571	[A].NRETAFVHAISSAGVMYTL.[T]	2067.03279
3572	[H].PGQPEVTTATGLLGQPEAAMV.[L]	2067.04268
3573	[T].QSPELTPSGPAPAGPASAPETN.[A]	2075.988
3574	[M].APAGASPAQPGDAQLAGGPTSASP.[Y]	2075.99924
3575	[S].PPMLEGAGGLGISVWTPGSPH.[Q]	2076.02189
3576	[E].LGEAQGPEALGPDRLSLHSE.[V]	2076.03562
3577	[Q].AQAQARDGGAQLAGPAAEADPL.[G]	2078.02612
3578	[A].NLQGPTGVCGLRTFSEASAL.[L]	2078.03351
3579	[G].QGKGGAGGGGGGGGPGCGADMAPGPP.[P]	2080.89235
3580	[G].PQGVPGGAGGHDGSGHPAGPGDTV.[S]	2080.94312
3581	[L].GQASATSHIYQGPESSLPGPP.[S]	2080.99342
3582	[F].PGSPGEKGEKGSTGIPGMPGSPG.[P]	2080.99679
3583	[K].PGKGHSNAFQDRLGVSQER.[H]	2083.04277
3584	[K].GLGGSGGAGGPPGTPYELAKEDP.[Q]	2083.99309
3585	[N].QGPPGAPPHAGGPPPHQYPPQ.[G]	2084.00968
3586	[G].PQGNAGPQGHLGPQGPPGPQGH.[I]	2085.00091
3587	[M].PDGSIRGDPANVEFTQLCI.[D]	2089.00188
3588	[L].SFLPQPPQEKAHDGGRYY.[R]	2090.00901
3589	[P].VPSSTEAQGVAGPAAEIPASGGH.[G]	2090.01489
3590	[P].GPSTYPTPAFPPGTSYPAEPG.[L]	2090.97056
3591	[L].PGSPGEKGEKGETGQPGPPGLD.[G]	2090.9989
3592	[Q].ATTPPASSLCPPGAAGTPAGSQP.[S]	2092.99679
3593	[H].RRDDTGGRPGSGAAASAVPSAH.[R]	2093.0231
3594	[A].AAAATAAASTAAAAAATTTSAAATSA.[P]	2094.03093
3595	[P].QPSATPTGQQQSQHGGSHPAP.[S]	2097.96967
3596	[R].PPPLTRPMSLAVPGLTGGAGPP.[E]	2099.13177
3597	[K].SRKAKATPMTRGAGAGGRPRG.[Q]	2099.1363
3598	[G].GGSAAGARGAGAGAAASQELNNSR.[P]	2101.01293
3599	[L].GPSEQLGQAGPTVGPQQQPPAG.[A]	2101.03087
3600	[G].GPSVGPADQTVPPGSKQESPPA.[A]	2103.03529
3601	[Q].PGAAEGGQFLGGPPPGVCPPEL.[Q]	2104.0168

3602	[P].EPAAGEPGRAATAPTAGGEPLSP.[P]	2104.03054
3603	[T].QRSPGCRPLPSAGSPTPTTH.[E]	2104.03525
3604	[R].TGQPGAVGPAGIRGSQGSQGPAGP.[P]	2104.053
3605	[E].KGETGQPGPPGLDGPTGEKGEP.[G]	2105.01455
3606	[N].PQGYHPAFPVAQGAAEALGHS.[F]	2105.01991
3607	[L].SPTGQATTPATTGPPSQPANPQ.[E]	2106.0098
3608	[V].PSPGTQRSPAGSPSPCRGRPG.[G]	2106.02574
3609	[E].PSGRDAQGRASTPAPPAWER.[E]	2107.04277
3610	[L].QEKPMEIAPSAGFGGNLHPGA.[R]	2108.02295
3611	[A].PGSSGPGALPPYAPKLSSSAGLP.[L]	2108.10225
3612	[V].AAISDLVGRVASGWLGDAVPGP.[V]	2108.11348
3613	[G].SRQVCGTRSVPGSFLGSAATA.[G]	2109.05056
3614	[V].LGGGGPGGGGGLGGPGGSVPFKLEE.[N]	2110.05636
3615	[L].PATSPPPHEVLDGLAQGLSHS.[L]	2110.05636
3616	[A].PNATAAAAAAWTNISLPEMPL.[F]	2110.06375
3617	[Q].GTPGTQGLPGPQGAIGPHGEKGP.[R]	2110.06759
3618	[L].PPGFHPHPGPNYPPFLPDQ.[M]	2111.01337
3619	[V].VGPGGSRAGGSGWGGSGAGPKGQGN.[L]	2111.99655
3620	[A].RQCLQTSSLPFFSVEAKN.[E]	2112.05425
3621	[G].PQGPPGTPGTPGTTRYSRATL.[V]	2112.08324
3622	[P].STLFSAAGAAHPPGTPFGPPPH.[H]	2114.0454
3623	[T].AAAQSAPGTDTATPGAAGGATAASAA.[A]	2114.99488
3624	[K].PAGEALGSAGGPPTRSLSTTNSS.[S]	2115.03126
3625	[R].GPAGPSGPAGKDGRTGQPGAVGPAG.[I]	2116.053
3626	[T].PEPALAQPDSTAPGGATGQAPPS.[S]	2117.01455
3627	[G].GPGGASLVVNSQVNLVFLMVT.[L]	2118.12635
3628	[N].KPGGMTRDQSAGTPRSSGEVT.[Q]	2119.01965
3629	[T].PGAATHRVSSPLWGPPHSAPP.[E]	2119.08318
3630	[K].AGASPFSSVSSKPPIGRRSSGS.[V]	2119.08905
3631	[P].QPPSASSQTGLSQANLSAGPSH.[N]	2122.01595
3632	[S].PGEPKSSVPDTGTPTPASTPQA.[V]	2122.02987
3633	[L].LLCGPSPGAPPMPVEAGGKEAS.[S]	2122.03074
3634	[L].PGSPGAKGEQGPAGHPGEAGLPGP.[S]	2122.03121
3635	[P].ELAVDTELSQAVSEVGPGPPQ.[H]	2123.05027

3636	[L].GVGAESRAPAQGQPGPPPGAGAPP.[T]	2123.06284
3637	[P].GLSGGIVPGPAPWGTRLAGQHP.[P]	2125.13013
3638	[D].QGAPGAVGPAGPRGPAGPSGPAGKD.[G]	2126.07374
3639	[P].GPSPTTVPGPASGKPSTEPPPAP.[E]	2126.07642
3640	[G].PGVSFSPGPTPTPAPTAGSFAGGA.[G]	2128.03456
3641	[A].GVQQPPATSTGGPAASAVSASVST.[Q]	2128.05167
3642	[E].TGPFGPPGPRGFPGPPGPDGLPG.[S]	2128.06105
3643	[R].GIGGLHGLPGSKGFPGSPGADLH.[G]	2128.09341
3644	[Y].QRAAGGGGAAIGEGEPWVLKPL.[R]	2134.14036
3645	[M].PGSPGPKGSPGSVGYPGSPGLPGE.[K]	2135.04037
3646	[N].GAPGPKGEGERPAQNEKRKE.[K]	2135.0952
3647	[I].PGERGPPGPPGPPGPPGPPAPVGP.[P]	2135.10325
3648	[Q].LSGEPPSTPAGAGSALSSHNGLE.[K]	2136.02037
3649	[E].QPGGLEGEDKSAAPFSSPPPAP.[H]	2136.02439
3650	[T].AESPVEVNIEVTDVNDNPPV.[F]	2137.02953
3651	[L].GKNSLMSLSPHVGELSNLTH.[L]	2137.07063
3652	[A].GSPPPQEPPERPESPQPSGAP.[D]	2138.01489
3653	[Y].PAAPTMPSPDIVYGALPPPAC.[E]	2138.02967
3654	[K].GPEGCVKVPGSAPYRATAHSP.[S]	2138.04475
3655	[K].PGSRETPPNPHPMALGSPAQP.[L]	2138.04475
3656	[D].GSPGANGIPGTPGIPGRDGFKGE.[K]	2138.06251
3657	[G].AVGGFGGLGGFGGGISGAVGGFGGLGG.[F]	2139.06178
3658	[G].GLGGFGGGISGAVGGFGGLGGFGGAVG.[G]	2139.06178
3659	[E].TVPEELQNGRGFGYVVAFR.[P]	2139.09816
3660	[G].MPGQKGQPGSPGLSGQPGLPGPP.[G]	2141.0808
3661	[G].PQGPPGPQGNAGPQGHLGPQGPP.[G]	2142.04752
3662	[A].AGVAADWAAAGLADGARAAGHAGH.[G]	2144.03802
3663	[R].ALGDQGWGPLGGVGVTGNPGTPH.[P]	2144.05194
3664	[Q].QLGPEQTSPTREAPSLTMKG.[G]	2144.06521
3665	[K].KGREGGDVSGAFTRLRKRR.[R]	2146.20642
3666	[D].GPFANPSGSDLINNTFQGTGGP.[I]	2147.99924
3667	[P].KEDTALGNGSPREEESKVDS.[P]	2148.00511
3668	[A].GPPSGGASPTPPAASPSGGSATRPS.[S]	2148.0316
3669	[S].NHVSSVSAHFNVTVEPMHR.[M]	2148.04033

3670	[T].SSLPSFGQAPTSVSIPAGFNPS.[T]	2148.06077
3671	[V].PGQPLYGPGAAGADAAVPSTPAGQ.[D]	2150.05127
3672	[P].QPSLGVSFGAPFGSGIGTGLQSS.[G]	2151.07167
3673	[N].GNGLSTPPGPGGGPHPPHTPSHP.[P]	2152.03187
3674	[R].GPPGEPGPPGPRGPPGEKGDSGR.[P]	2152.053
3675	[L].KTAIMSFINAVLSQGAGVESL.[D]	2152.13183
3676	[S].GAGPAGGYPTLGEHASSFGGPEH.[L]	2152.96827
3677	[K].TPASSVASVGGPSASSSTSAVASTS.[S]	2153.02043
3678	[L].QCRVLGGGGPGGGGGLGGPGGSVPF.[K]	2154.0509
3679	[Q].GQAEARAPAGTALPAAAQQAGPGP.[V]	2158.09995
3680	[-].AGTTHTSLGPAPSAHDALREAG.[H]	2159.04758
3681	[H].PEGTQVPGRAGGQAAFLGGGGFQ.[I]	2159.06284
3682	[Q].NDLNRGGPGAQSTSSRGQATW.[S]	2160.01768
3683	[G].AAEAARGASRAQCHTLQAGFS.[S]	2160.03631
3684	[T].GPGAGGEKAVSQGFVVTGEEKTG.[A]	2162.0724
3685	[N].NGIPEDSKVEGPAFTDAIRM.[Y]	2163.03866
3686	[T].PGQPGNQLYVVGTSSMSLGQQ.[K]	2164.03391
3687	[Q].PQGEQGQAGIQGPPGPPGPPGPSG.[P]	2164.04177
3688	[L].PGSKGFPGSPGADLHGDLGFPGP.[A]	2164.04579
3689	[Q].RQNEELEGLGHNREPHPGP.[P]	2166.0435
3690	[D].KDGELCKTGNAYLTAELSTP.[D]	2168.05397
3691	[Q].QRPSGPGPVTQGPQQPQPPSQ.[Q]	2168.0843
3692	[P].GPAPGEVTAASAGYLGDAPGPSVE.[E]	2170.02987
3693	[G].AGVGGFGGSGGFGGGISGAVGGFGGLGG.[F]	2170.03121
3694	[E].TGAAPQFHAAEAGGLGAHLEQH.[T]	2170.04244
3695	[D].FFAVNQHAGPYVTMIAKME.[P]	2170.04599
3696	[S].PAGGGYYLAVGGAAAQHSWSHI.[S]	2170.04646
3697	[P].AAEAAAAAGGGGATAAAARGGEAAAEV.[T]	2170.04831
3698	[M].QPPPGMSLPPADIGPPPYEPP.[G]	2170.05252
3699	[S].QPPSQHAAPSPVQHQAGQAPH.[L]	2170.05367
3700	[T].LQGDRQTDPPPTPPPLCSPP.[V]	2170.05973
3701	[K].TPAKADPALLNNHSNLKPAPT.[A]	2170.16149
3702	[P].QTPGRLQPTPVMPPVPSAPLA.[P]	2170.16889
3703	[Y].CLLPAGGVCGPGVQAWSGSLW.[A]	2172.03649

3704	[S].ADNIHTLTGFAKPVDIYCH.[R]	2172.05425
3705	[V].CAAVPVPEGAAAASVTVPSPEGH.[V]	2174.05464
3706	[L].PGAPGQGGAPGPPGLPGPAGLGKPGL.[D]	2174.17166
3707	[D].PVISGGEEPSVGAGGATYAYPPV.[Q]	2175.06044
3708	[S].QSPKQEAGGAAPGQHRGQATGAA.[S]	2175.06497
3709	[G].HKGERGYPGNAGPVGTAGAPGPQ.[G]	2175.06899
3710	[P].AASPSAPQSPGYQVSQLMNRS.[P]	2176.04514
3711	[Q].GGAVEGSLWPLTPGLCGALSFS.[P]	2176.07432
3712	[G].SGPLEVTTDSLPNGPALADGPAP.[V]	2176.07682
3713	[E].EDAARPEKAQPSPPAGKMSNP.[F]	2178.06079
3714	[P].EPGRAAASGAAASSADPTALGGPAGA.[E]	2181.05306
3715	[P].SPRMQPQPSPHHVSPQTSSP.[H]	2182.04581
3716	[Q].QSQQGLSPSHVAGSSSQGQALQ.[Q]	2182.04831
3717	[C].PGVPMAGVTTGYNGSLLPEGHL.[E]	2183.08013
3718	[P].GPGPSPTTVPGPASGKPSTEPPPA.[P]	2183.09789
3719	[N].KGNDPNVNSHFQQEFPSLQ.[A]	2186.02612
3720	[E].QPAGAGTGGGPQACESKLGFAPAG.[A]	2186.02949
3721	[G].PGPSPGAMLGPSPGPSPGSAHSIM.[G]	2186.03688
3722	[A].REEREPSISGKFKIAGVLAV.[G]	2186.22918
3723	[S].PGATSPFPVGASTPKMGAIGSLQ.[G]	2187.11143
3724	[V].QRVVGALASQDYRAVAAQRK.[D]	2187.21051
3725	[S].GLPAPSMGLEPPQEVPEPPVM.[A]	2188.06645
3726	[S].TFRMVTGDTLPSGGEAILEGH.[S]	2188.07029
3727	[L].PQSSSGNTMAPPHSGSSPNVHI.[L]	2189.004
3728	[L].SPTASSLSPGPDAPLAPASSAGPGP.[G]	2189.07207
3729	[F].IEAQPEPAGAPTDTLPATGQPQ.[G]	2189.07207
3730	[G].PSGPLGHPGLPGPMGPPGDPGIQG.[Y]	2189.0808
3731	[E].ERAPTQSPELTPSGPAPAGPASA.[P]	2189.0833
3732	[A].WLENPQSVHKSWDNFFR.[K]	2190.05155
3733	[A].PGGPAQPPPPQASASDLQFSQL.[L]	2190.08257
3734	[Q].QPGLQNLNAMQAGGPRPGQQQ.[Q]	2190.08326
3735	[Q].PYGSGKMKMFQEVPAVDAFG.[P]	2191.01984
3736	[H].SDPDLGSVTGRSLCKENVEGA.[K]	2191.02955
3737	[I].QPHGGTSAPLEDTPPLPDAQY.[V]	2191.0302

3738	[V].SAPAAGTPAFWVSGWLGPQQY.[L]	2191.06071
3739	[Y].AGGPGGPGGLGLPSHSARPSTDFT.[Q]	2193.06832
3740	[K].PGAPGTPGQPGIPGMKGHSGAPGPA.[G]	2193.08695
3741	[V].EGKAPGNGAALAPVGVSAEKQNQ.[A]	2193.12583
3742	[R].PPGSSRKGAGSPPRPGPAAPSGHA.[G]	2193.12717
3743	[I].PGLGQQGAQGRIPPLNPGQGPGP.[N]	2193.15232
3744	[F].PGSPGEKGEKGSTGIPGMPGSPGP.[K]	2194.04447
3745	[G].NAGPVGTAGAPGPQGPVGPTGKHGN.[R]	2195.0952
3746	[Q].SPSPGTPSATPAADPALPTLSSSS.[A]	2196.06665
3747	[T].PGSHGLPGRDGRDGIKGDPGPPG.[P]	2196.09045
3748	[R].GVHSSVASATSVATKKTVQGPPS.[S]	2196.16189
3749	[N].IALALGHNSVVLYDPVVGCSL.[Q]	2197.16855
3750	[T].GPPGSPGPRGNAGGPGLPGPPGPPGP.[P]	2198.11012
3751	[S].RGPWSSAPTSRAPSPPPQPQP.[P]	2198.11012
3752	[P].PGSPGPRGNAGGPGLPGPPGPPGPPG.[Q]	2198.11012
3753	[L].RGPGPSSAAEPEAVSHSSAGVLH.[S]	2200.07413
3754	[L].AGRSEEVPAPAATPLPQGPHAF.[L]	2200.11454
3755	[Q].PAAASSLNSYGAQGSSLASYGNQ.[P]	2201.01053
3756	[G].GPSAKQASGEASSLRDYAASTM.[T]	2201.0139
3757	[L].RGASSPQWGPCHAGTIPGTTY.[T]	2201.01926
3758	[Q].ATAPMATTAPHLDGHPPTNTIS.[T]	2201.06554
3759	[Q].IQMQLQHELQQQAAFFQP.[Q]	2201.0808
3760	[K].GPGTPAFPHYLPADPRPFTY.[P]	2201.08145
3761	[L].AGEPGRAGAPTLPRPRRELSL.[G]	2201.22616
3762	[D].LSFLPQPPQEKAHDGGRYY.[R]	2203.09308
3763	[S].KNQLPDAEDIPENKETVYT.[K]	2204.07173
3764	[Q].ARNGAGGGPRGQTPNHSQRDGD.[S]	2205.02523
3765	[I].GALPTAAGSPDSPGGRPGATMIAH.[E]	2205.07169
3766	[A].PAASSAAAAAAADKADDEDDDEE.[E]	2205.8902
3767	[R].APGQPPRRGTVMYVGLTDFK.[P]	2206.14373
3768	[G].AGPAGGYPTLGEHASSFGGPEHL.[L]	2209.03087
3769	[M].PPPPMPPGAGGHGPPSAGTPGAGHP.[G]	2211.04
3770	[S].SGQMGSPLALPSEHLGGGLGMGAA.[S]	2211.05326
3771	[R].PPGGPSGADSYLVQQPVDADALG.[L]	2211.05642

3772	[P].FFWLAGAGSAVFWVLGATLVV.[I]	2211.20011
3773	[E].GPRGEPGFMGNIGPTGSVGDRGP.[K]	2212.05637
3774	[I].SGAAAPAPAPSGLGYGPPTSLASASG.[S]	2212.08805
3775	[R].GPTAMTRFGVPAEGRNPPPFP.[G]	2212.09678
3776	[G].PGDASLPGPAPGSARPPSLPQGAD.[M]	2212.09928
3777	[P].PGGSPGRSSPAGGSPGKPGSTPHVS.[G]	2215.08503
3778	[A].TGPPTLVPQIAVEGAEAVSGAPGP.[S]	2215.16049
3779	[P].GGPEPELSPITEGSEARAGPPAP.[A]	2216.08297
3780	[L].SKPAEGGAQAPPGGSLEPAPSSQP.[A]	2217.07822
3781	[D].GPRSTPGAQAAPSTARSPQDPAP.[P]	2217.10068
3782	[F].GGSPPVTGPGSVLALGKRKKRR.[H]	2218.32548
3783	[C].PQPPSASSQTGLSQANLSAGPSH.[N]	2219.06871
3784	[S].IDPPSSTVQQGQDASFRCLI.[H]	2219.07611
3785	[Q].GPLMGLNPRGMQGPPGPRENQ.[G]	2219.08082
3786	[K].VVSSGGPGSSLEARSPADLHLSP.[L]	2220.1255
3787	[G].EAAAAAGAGAGETAVKVEGPGSPGVP.[G]	2221.10952
3788	[S].RPSMPVASGAALPSASPSGSLSPP.[P]	2221.12814
3789	[T].PSTDPSLGPSFNPSPGLSPSPSP.[P]	2222.06117
3790	[Y].GSPGASGVGTSPGPRDTELAASGPP.[T]	2222.06838
3791	[A].AGGGGPAPLSSAASSPLSSSLGTVGH.[R]	2222.10476
3792	[L].QGFPGITPPSNISGSPGDVGAPGI.[F]	2222.10879
3793	[R].PSGPGPVTQGPQQPQPPSQQPL.[P]	2222.12002
3794	[A].QGVAGSAGAPGATLMVQLQQLPL.[G]	2223.18018
3795	[V].HPLDAFTQGFGEQPAGGMPLGP.[P]	2224.04916
3796	[V].APGPGVSFSPGPTPTPAPTAGSFAG.[G]	2225.08732
3797	[R].PGPSPGPGPSPGAMLGPSPGPSPGSA.[H]	2226.04956
3798	[V].AATPYSSYIPYNPQQFPGQP.[A]	2226.05021
3799	[P].GPMGYTGRPGPLGQPGSPGMKGE.[S]	2228.05868
3800	[V].MTLGAPHNQVGEAPSAVPEVHS.[Q]	2228.07644
3801	[S].QAAAQPSTPAGTPRSGGGHSPAQP.[P]	2228.08028
3802	[Q].DLWSAAGSPAAQPLAQAWMQL.[L]	2228.08046
3803	[L].QGPGGIPPDNGYVEKPTPLYE.[L]	2228.08699
3804	[T].KDSPGKNPMASPSKELPGRES.[I]	2228.09757
3805	[E].PGQVATGEEGNLKPEFVDEVL.[T]	2228.10812

3806	[S].SGPPGPPGSIGPKGPEGLQGQKGE.[R]	2228.13059
3807	[I].PQMPAPPPPRTPPGSPAGYWK.[R]	2229.12735
3808	[G].AQQSLGGGWAGGGRAGPGPPEHSV.[P]	2230.0748
3809	[S].PGRGGRGTQEVASTPAASLPSSF.[C]	2230.12108
3810	[Q].GPPGGQAAGLRLTPGGMALPGQPGG.[P]	2230.13971
3811	[G].SPGRSSPAGGSPGKPGSTPHVSGLG.[S]	2231.11633
3812	[A].LPGMGGPGPVGTPDIPLGTAPSMP.[G]	2232.1039
3813	[T].TPASGPAPAPAEPAGSEPGLGSDLS.[K]	2233.0619
3814	[A].TAAANNNFVNFGVADLNAVQSP.[G]	2234.08363
3815	[I].PYSQQRPSGPGPVTQGPQQPQ.[P]	2234.09487
3816	[-].MGGPSPAGVASGRSTRSQASAFPF.[L]	2234.09487
3817	[G].PVSSGGLMQLPTSFTLMPGGAVA.[Q]	2234.11955
3818	[R].VTPAAGGGGTWGRERFARFGPS.[P]	2234.12136
3819	[G].ESVHRVLTGQAEETEATAAVH.[G]	2235.10001
3820	[E].VGGGEAGTVIGGSAGPSPPATLAPDT.[R]	2236.10918
3821	[R].GPMQLSVGGAAAGVGAVLAAGLLWA.[C]	2237.21108
3822	[L].ETQRSTPTCCSSDTAQSAPGP.[C]	2237.93975
3823	[Q].RQQQQQQQQQQQQQQQQAA.[V]	2238.07184
3824	[P].QNLPCVPQWRNLGPTTTGTV.[I]	2239.12881
3825	[M].IGPNGSSLGAPSPGPPGPGVSPVQL.[A]	2239.17172
3826	[R].QQAARLAQGVGPLAPPHPPPPAA.[P]	2241.22509
3827	[L].PGLDGLKGDKGNPGWPGTPGAPGP.[K]	2242.12511
3828	[R].GPGAPQKEEDLAGYYLTTWF.[G]	2243.06553
3829	[P].GPPTSPETAGTTRPSSSTTSEVP.[R]	2244.06262
3830	[M].HAKNGGGGSGSHRSSPVPGAPAVC.[E]	2244.06867
3831	[C].EVHLVESGEGFVQPGGSLRCS.[C]	2244.07136
3832	[L].QPGGQSGFLPSGAPAQQMLLPM.[V]	2244.07875
3833	[C].GPQGLGSPGTSAAMLGPLGRLSMG.[P]	2244.11111
3834	[L].PGSPGEKGEKGETGQPGPPGLDGP.[T]	2245.07313
3835	[Q].QPMLSGVQMAQAGQPGKMPSGI.[K]	2245.07737
3836	[L].PAVTTSGSVSSRGHSFADPASNL.[G]	2245.08436
3837	[T].PTNGTPGPPLDFPERFPASTF.[F]	2245.09241
3838	[G].SLHSHDRLSYPPHSVSPTDI.[N]	2245.09962
3839	[C].RNSAWVSGGVTQPGGDVHLGPGP.[H]	2245.11085

3840	[Q].GPAGPPGPPGPMGPPGLPGPMGIPGS.[P]	2246.10966
3841	[P].AGPPGPPGPMGPPGLPGPMGIPGSPG.[H]	2246.10966
3842	[T].APGPSGPGGGARSGGGRPAAANAARE.[R]	2246.11331
3843	[K].NATANLAKTQGSQGSPNSVKSSV.[S]	2246.13713
3844	[V].KDEGLTSAPQEPKAPKASPASH.[A]	2246.14115
3845	[P].KGEGGVVGPQGPPGPKGEPGLQGF.[P]	2246.15641
3846	[G].QSAVSESLLVQTPAVPPGPCLP.[P]	2247.16894
3847	[E].QRDGQLTLTPMGRKMAAFPL.[E]	2247.17365
3848	[E].GVGGPTPPGAPPKASEPHLPAAEL.[K]	2247.17681
3849	[Q].QKEETGPQGNNLPSPGAGSWPP.[P]	2248.0629
3850	[Q].APAGGLTEEELMEQLEQCDL.[A]	2248.99481
3851	[E].KGSYTPDPCDVQTEVSTDHL.[L]	2249.00267
3852	[G].RMIAFTMALMGCLLIMYKA.[I]	2250.13396
3853	[Y].KNYFTAGAAWPVIIFLILVN.[I]	2250.26852
3854	[Q].DTTSLPTGATAPSETATDLTPGF.[G]	2251.06123
3855	[N].FHSSVAFMFRNPPAVAMASPA.[S]	2251.07869
3856	[A].AGRQSAGPQPSPEGVTGPRELC.[A]	2251.0884
3857	[Q].GSSSPQRAAGPNRPEAPSMLSPG.[P]	2251.0884
3858	[T].GTPVTHTTTATPTGPHTPFTTQ.[S]	2251.09895
3859	[P].STLFSAAGAAHPPGTPFGPPPHH.[S]	2251.10431
3860	[S].SSPSTPVGSPQGLAGTSQWPRPG.[A]	2251.11018
3861	[E].QRPWGTQQSRAPAWTSHTR.[P]	2251.12275
3862	[D].GVMPVAPTSAVSSSGSPASVMTSI.[R]	2252.07848
3863	[K].GEPPGVGVAGAGWAAGPVDSAFPSV.[V]	2252.09822
3864	[D].GPGAQEPVHLTDVQAAMEFIAA.[A]	2252.10159
3865	[T].GPTSSPTAPPASATSTGAPTSTGRP.[S]	2254.09459
3866	[G].SAGPDASVMNLISALESRGPQPG.[P]	2254.11322
3867	[T].GPKASPDPGTDPGPRGEPGAAGIPG.[E]	2255.1051
3868	[S].GVSAAGGGPAGAAGGAAGGGPAAGPADHGL.[A]	2256.0752
3869	[P].GQSYLRPPGPVVMQTVSQAGAL.[N]	2256.18051
3870	[S].SGTPKAPAAGRGDLSLPPPPTPTA.[P]	2256.19827
3871	[A].AAAPSPGSARSAGSHTTRDSSSLS.[S]	2258.07559
3872	[P].SPAPAAAAATAAASTAAAAAATTTSAAA.[T]	2258.12589
3873	[SE].QAYVIRGETLRTELPQREV.[F]	2258.22515

3874	[D].AGAHVEGSAVNGGEDSYAETPLQ.[L]	2259.01601
3875	[E].GPASTQASLATSGSTHLAQMETL.[L]	2259.09215
3876	[P].PATTTGAAPPQPPGTLSKPMSVH.[L]	2259.14379
3877	[Q].KGERGPPGESVVGAPGAPGTPGER.[G]	2259.14763
3878	[S].LVNCPPTTCARPQSGPGQCC.[P]	2259.97259
3879	[G].GALGSGFVGGYGGGLGAGFGGGFGGGLG.[G]	2260.07816
3880	[S].LGSHAGPNLEEAVNFRSSDLF.[D]	2260.09928
3881	[R].GQRRCAPGLGAAPQAADPADPLA.[E]	2260.12512
3882	[G].GRSISTCSTRFVSGGSAGGFGGGV.[S]	2261.07275
3883	[G].GPGEPGPPRGPYAGYRTYGAEL.[P]	2262.09381
3884	[D].AGPDYIKQRFQEGVDAKENP.[E]	2262.11493
3885	[G].PGGGGINVQEILTSIMGSPNSHP.[S]	2262.11831
3886	[F].PGAAGYPTYRLGYPQAPPSGLE.[R]	2262.11896
3887	[A].PAAAAATAAASTAAAAAATTTSAAATSA.[P]	2262.12081
3888	[P].APAAAAATAAASTAAAAAATTTSAAATS.[A]	2262.12081
3889	[Y].GAELPATPAFSAFSRALGAGHFS.[V]	2262.13019
3890	[S].RGAGGGGSGALPAGTANSGTARHWP.[P]	2263.1075
3891	[P].PAPQMPTAPPAVPQAPPTVMPAP.[L]	2263.16136
3892	[A].STIPLVQEEVGAPSGGTGVSKPGP.[P]	2264.17687
3893	[A].LCPSSSGLTPASCSAASTPGELD.[P]	2265.00095
3894	[R].GEKGEAGRAGEPGDPGEDGQKGAP.[G]	2266.03306
3895	[T].STSRGTGTPVTHTTTATPTGPHT.[P]	2266.10583
3896	[A].GPAPAPHAPGGPPSNADLLSCLLG.[A]	2266.12848
3897	[C].HLAGLSGDGPVPGAEPRSEGRAH.[G]	2267.12757
3898	[D].GPGAQEPVHLTDVQAAMEFIAA.[A]	2268.09651
3899	[-].EAAAAPTAAPGPAQPGHVSPTPATT.[S]	2268.1255
3900	[A].AAAAGGGGATAAAARGGEAAAEVTGWP.[A]	2269.0956
3901	[L].PGADRTAGGIPVPNGNHPREKE.[R]	2269.14322
3902	[V].VFAEHVGTGQAGGHGGVALGAQSY.[Y]	2270.09487
3903	[S].SSPALLLSPPPPPGLPSLHSLGH.[P]	2271.24958
3904	[A].GEPKGGPGPGSGGGAGTGAGAGGPGTGHL.[P]	2272.07011
3905	[-].EAFLGSFVAGGMGPAASSHGSPVP.[L]	2272.07029
3906	[P].PGGSPGRSSPAGGSPGKPGSTPHVSG.[L]	2272.1065
3907	[I].QPQPQPPPQHPSQPSAQSAPAP.[A]	2272.11052

3908	[P].SQPGAYQPRPGFTPPPGSTMSP.[L]	2273.06554
3909	[P].GTDPGPRGEPGAAGIPGEPGSPGKD.[G]	2273.07928
3910	[Q].QEQQGLAGPLGDPLGGDHLAAGGD.[V]	2273.07928
3911	[R].GLGNGHRAMGSLQQALVCFEK.[R]	2273.12777
3912	[C].GPSPGAPPMPVEAGGKEASSQPNI.[C]	2275.10232
3913	[A].APAGTTFLQYQPPQLQPDRM.[Q]	2275.11758
3914	[P].PGRAGAGAAEGQAGSPLFTSAPPAH.[R]	2275.12142
3915	[S].PGAGGGVNERRRHAHSAPSAHPG.[M]	2275.12997
3916	[R].NGIGGASVSPGPGAPLTHLSTPSGGS.[E]	2275.13131
3917	[S].AALEPGGAQQSLGGGWAGGGRAGPGP.[P]	2276.11667
3918	[L].GAHRYAPGSAAMKDCNHCVC.[Q]	2277.93687
3919	[L].FGEFLNSDASAAQPAPFPSTHS.[A]	2278.0411
3920	[K].PAPDQAPAALTNRSGHRDPGPPG.[S]	2279.12757
3921	[L].GAGQASLFGNSQPKLGGSLGTGAFG.[A]	2279.14148
3922	[Q].APSTGAPPGHTASVVQAQAQPHAV.[Y]	2279.15272
3923	[G].GAAGTRSLAGACLRAGQHGASPRG.[R]	2279.1534
3924	[C].ASGPTAQGAVSVALDVQLQHSSGV.[R]	2279.16261
3925	[E].ELQIWVQQHSPKSGEEAVTL.[L]	2279.16664
3926	[A].GPAPLEEAGLSLDSKPTLAAGTPS.[V]	2279.17653
3927	[R].GPPGEPGRPGPPGPPGPGPGGVAPPAG.[Y]	2280.15199
3928	[S].SAAPAPQAAPAPTPAAAAPTPAPSAQ.[A]	2280.16189
3929	[S].PGGSPSGRSVKSESPGVRRKRV.[S]	2280.26433
3930	[A].ASAQEKASSPSETCDSEISEAP.[V]	2280.97724
3931	[L].PLDQSQAGWPPPATSLQPASFS.[G]	2282.10879
3932	[P].GAPGTPGQPGIPGMKGHSGAPGPAGL.[P]	2282.13463
3933	[A].LGPGSSSAGSGDTAAQSQAAPGPSPQ.[S]	2283.04837
3934	[G].AAGGPEEARSWPGVRVMCQPQ.[R]	2283.07573
3935	[E].SGRGAEPGGPQPPGERDPGSLQH.[P]	2283.08609
3936	[G].AMLGPSPGPSPGSAHSIMGPSPGPP.[S]	2283.08965
3937	[S].IHEDTVPTTASFGAKMFNLTS.[Q]	2283.09617
3938	[P].GSGAAAGALASGGSKEEFVATFKGN.[E]	2284.12041
3939	[I].RRMAPYKGAGAPAGALDYVAFS.[S]	2285.14955
3940	[P].AAEAAAPGWAQARGHPGGELAAAAS.[A]	2288.11667
3941	[V].ATGGGAAQVGQPAATADSGASNGSQSA.[S]	2289.03378

3942	[P].GPQGHLGPQGPPGTPGMQGPPGPR.[G]	2290.11456
3943	[L].PGPKGEMGPVGPAGNPGAKGERGSS.[G]	2291.1197
3944	[S].ASLITMFWSLSVSSFAVGGMIA.[S]	2291.14504
3945	[L].PGSKGFPGSPGADLHGDLGFPGPAG.[D]	2292.10437
3946	[G].DPQVEGLTASYRPLSASSQSSL.[R]	2293.13064
3947	[P].WPGSPPGGSRHGLGPGPSPSPSPE.[E]	2294.09487
3948	[E].QSATPAGAVSTPEQSATPAGAVSIP.[E]	2295.14629
3949	[S].PGQGRTLPRSGPRNLRVFGET.[T]	2295.24287
3950	[G].LPGLDGIPGIKGEAGLPGKPGPTGP.[A]	2295.27071
3951	[L].LASVAGSGGGGGGGGPGTATGLDAGGLGPA.[G]	2296.11639
3952	[D].KASASGSGAPVGGSISSGSSASSVTVT.[R]	2296.12629
3953	[E].DAEKNPKAIDTWIESISELH.[R]	2296.14557
3954	[S].AAGGGPAGAAGGAAGGGPAAGPADHGLAGR.[G]	2297.11298
3955	[Q].QPPATSTGGPAASAVSASVSTQVEP.[E]	2297.12556
3956	[K].GPGTPAFPHYLPADPRPFTYP.[P]	2298.13421
3957	[L].LGGSRPGKDGPVRPHFPPADLQ.[T]	2298.21017
3958	[M].GPRGSKGAVGPPGLDGLPGTSGLPGP.[V]	2298.22007
3959	[P].DVVVKGWLYREPRGGGARPW.[L]	2298.22543
3960	[P].PGRVSSIVAPGGTLREGHGGPLPS.[G]	2298.2313
3961	[L].KICYLSYGVWLKRKHAYI.[C]	2298.25798
3962	[G].QSRPPESSPSPQPPDRGTGPPAG.[P]	2299.10616
3963	[T].PGAAGGATAASAAASVLGGSAAPATAGDT.[T]	2299.11606
3964	[Q].RAPGGSPGSAGLVQRLEEYAATL.[A]	2300.19933
3965	[A].ASAPAPGPASSPEASPAPGFPFPPP.[W]	2301.11862
3966	[V].WSPGVAPAQPPAFPTSNPSHGTV.[V]	2302.12511
3967	[P].PGPSGPLGHPGLPGPMGPPGDPGIQ.[G]	2302.12848
3968	[-].MGQPWAVGTAEAAPARLPLVLTAL.[W]	2302.29178
3969	[M].GQPWAVGTAEAAPARLPLVLTAL.[W]	2302.29178
3970	[E].AAAGDAVAAASATAAVEPTELDFGAG.[E]	2304.09901
3971	[A].GLDPRCLGPGAPERGSGLDPWP.[A]	2304.11897
3972	[A].HPRGSMATLGGAAASAHHPITTY.[P]	2304.13021
3973	[I].PQGADSTMLATKTVKHGAPGPSH.[P]	2304.1401
3974	[L].DRASPAQGLPLDTAGGGHERGGVS.[W]	2305.12796
3975	[P].APAAEAGLEKDGGSLSQTPGTPPGP.[T]	2305.13064

3976	[A].PNPSITSSGQPLNVYSQPGFTD.[H]	2306.09353
3977	[F].PGSPGEKGEKGSTGIPGMPGSPGPK.[G]	2306.14452
3978	[T].GIPGMPGSPGPKGSPGSVGYPGSPGL.[P]	2307.14379
3979	[P].APAAGASGQASELAPSTADGSLLPAP.[A]	2307.14629
3980	[K].VSVDAGRGGGESLQEASPRLADH.[G]	2308.12762
3981	[D].GMKLESAHPRGSMATLGGAAASAH.[H]	2308.12849
3982	[L].QATPYTLDTPPTPPPQATPSQT.[T]	2309.12958
3983	[R].AGAGVEGGAFLGVQTSALGGEGPGLH.[Q]	2309.15205
3984	[V].KVWDMATHRAKEVHCVQTI.[A]	2309.16415
3985	[L].EGTANRPPPGSSGPVTGAEIMRK.[L]	2309.16665
3986	[H].PGQPEVTTATGLLGQPEAAMVLE.[L]	2309.16934
3987	[E].GPTSAASSAPKGRRSPSPGGSPSGR.[S]	2309.17049
3988	[L].KQNEAFPGPKLEVCGQLGSLH.[L]	2309.17068
3989	[S].PGGAGGTVLGEAPDVLNMLGADKLG.[R]	2309.18057
3990	[P].AAQPPSTGQSAGRPIPGPAVKKPP.[V]	2309.27244
3991	[T].PNPGPSAGPAAANGSAGGSRGGRTCS.[C]	2311.05923
3992	[T].KETVETQHFNSIEEEKVTY.[S]	2311.10885
3993	[F].PQGATSPQVLSASFSSGGSALHPQ.[A]	2311.13131
3994	[P].PADGDDEEMMATEVAPSAMAEL.[T]	2311.92507
3995	[G].SPGEKGEKGSTGIPGMPGSPGPKGS.[P]	2312.1187
3996	[I].SVMVARSNGTVSPSAFLEPYSP.[A]	2312.12272
3997	[A].GPPGPPGPMGPPGLPGPMGIPGSPGH.[M]	2312.13145
3998	[V].GSPGNRQGPAGSSDAQSEQASGGPGG.[P]	2313.00863
3999	[A].ADNRAVMAQQASVDRRGKRSP.[G]	2313.19527
4000	[L].GSPGPGLQGSAPNPRLRKPSDQP.[S]	2313.20582
4001	[H].SLTVPGSQHAVEIPGLKAGTSYT.[I]	2313.2085
4002	[S].PAASGTPTLSRLLEAGPTQFTTP.[L]	2313.2085
4003	[A].LGWAGAAILVLQTLATAAGAPGHPG.[D]	2313.27138
4004	[T].PPTSGTSTPTFGQNTPAPGVGAAGGS.[L]	2314.09459
4005	[S].TAPDGAALTPPSPSFAATGASSANR.[F]	2315.12623
4006	[L].QPSAEAVAANPGAMLELGPPHGVS.[A]	2316.12887
4007	[E].KSQPLCEPAPLSGASASPSPAPPA.[C]	2317.14927
4008	[P].GPSLGSTPLGMPPQNYALMQVAG.[Q]	2318.11553
4009	[A].SPAAASAVPGSGAAAGALASGGSKEEF.[V]	2318.12589

4010	[P].GPGVSAAPGPAAAANATPAEEGETKP.[A]	2318.12589
4011	[T].PGATQYVPGPGQPPAPSSYPGHR.[L]	2318.13125
4012	[A].AHSPHVMIGPNGSSLGAPSPGPPGP.[G]	2318.13462
4013	[G].KELSQETSSTAPGSEATIKQEP.[V]	2318.13579
4014	[E].GAPQIPGPHASSVTHFPPPSSLH.[Q]	2318.16764
4015	[P].KPGEAQNGSPEIHEAQLPFSSP.[H]	2320.12041
4016	[T].GPGAGGEKAVSQGFVVTGEEKTGAS.[D]	2320.14154
4017	[M].PGSPGPKGSPGSVGYPGSPGLPGEKG.[D]	2320.1568
4018	[A].SAKAAEAGPPTDRGVQVTEHGATA.[A]	2321.14803
4019	[G].SKVGADQSTSVPPLSICRHGGPA.[R]	2321.16665
4020	[V].QRGGAGGGPGKPGMGGTQGRAEKPL.[A]	2321.18912
4021	[R].LGLDGGQKPRAHMGPHSPAQLGP.[A]	2321.19314
4022	[F].PGSPGEKGEKGSTGIPGMPGSPGPK.[G]	2322.13944
4023	[R].AACTKDLTTMAVDVLLENATGE.[D]	2323.11559
4024	[N].PGPNERALPSEDLSQIGFPEGL.[T]	2323.15647
4025	[K].QQVEQQPSASQAGVQQPPATSTG.[G]	2324.11131
4026	[L].GPAAAPPAAAPDAAAGGAQTLADGFTS.[P]	2324.11533
4027	[P].QGVVMAASPGSLHSPQQLAEEAT.[R]	2324.1187
4028	[P].PGFGSISSSGALFSAGSQPAPPTFG.[T]	2324.11935
4029	[S].SPAASPNLSPGASPASSQSNSLTVP.[T]	2324.13646
4030	[G].AQAGGAGSLSPSAGAQSPAIIDSDPV.[D]	2324.13646
4031	[R].QAPIPAMETSGQRPQNSTSARP.[P]	2324.14117
4032	[C].ADRPSLINSGHSDLAPHPSVGPT.[S]	2325.1582
4033	[R].NAVSKYTMALQQKKALSKTSK.[V]	2325.29588
4034	[P].PGSPGPRGNAGGPGLPGPPGPPGPPGQ.[A]	2326.1687
4035	[G].VSAAGGGPAGAAGGAAGGGPAAGPADHGLAG.[R]	2327.11231
4036	[A].QAENGPATLPAGTTPATSEAPKMS.[N]	2327.11837
4037	[P].SSPVPTPSPSTPAPPTSSPSSPAPP.[NT]	2327.14015
4038	[E].GPATSIQNDLNRGGPGAQSTSSRG.[Q]	2328.12869
4039	[Q].PGAAEGGQFLGGPPPGVCPPELQP.[D]	2329.12814
4040	[G].KAHGGNNNKYSTFSGFLLYPD.[-]	2330.12002
4041	[T].SIAGSAEASVPQPSQQQQFLQE.[L]	2330.12589
4042	[S].LSGQPPGNSTVSPFTAASSFHLQ.[Q]	2330.14115
4043	[P].PGAEAGSLVLDDSPAPPAPFEHR.[V]	2330.14115

4044	[L].NTPAPVAMPASSPPGPPPAPEPGPP.[P]	2330.14854
4045	[P].PGSGPLEVTTDSLPNGPALADGPAP.[V]	2330.15105
4046	[G].DNFQKLMQIQYSLNGHHEI.[V]	2331.11864
4047	[P].GPQGHLGPQGPPGTPGMQGPPGPRG.[M]	2331.14111
4048	[S].QGPLMGLNPRGMQGPPGPRENQ.[G]	2331.14448
4049	[S].PPAQAFPAPSPFTFPALGPGPGPGG.[Q]	2332.17608
4050	[E].FYLASSPPTGSFMDDSTMHIP.[P]	2333.01006
4051	[E].AAPDPAGVGRGGGAAGPTSGGGGQPQW.[Q]	2333.10174
4052	[Q].SPPSSPAHRDSAYISGSPLGSHQ.[V]	2335.10616
4053	[S].GPAGKDGRTGQPGAVGPAGIRGSQGS.[Q]	2335.18614
4054	[F].PLGYQGASPAAGPPLQQRFGHVS.[Y]	2335.19419
4055	[A].FEAGIAGGGQGVLGGAGGGNGPGDPAVP.[G]	2336.12656
4056	[T].FIEAQPEPAGAPTDTLPATGQPQ.[G]	2336.14048
4057	[H].YVHITPGGSGSANPPVVSTVYAY.[I]	2336.15574
4058	[S].GPAGAPFGETVTSAPCSGRRHVQ.[T]	2339.13094
4059	[L].AQHVNEVARAQRGNNAHLCPP.[L]	2339.1534
4060	[Q].EQQGLAGPLGDPLGGDHLAAGGDVP.[P]	2341.14188
4061	[F].RAPGETSAQGHIPGEARTEPPGQ.[R]	2343.14361
4062	[P].PGAGPDPPSPPGADPARGAAGGGRFD.[R]	2344.1065
4063	[H].ASQEEVFRELESAVLSCLGGY.[S]	2344.11255
4064	[E].VEERAPTQSPELTPSGPAPAGPAS.[A]	2346.15719
4065	[K].FGSTKMKKGGGAAGGGGVASGGAGGPQP.[P]	2346.1619
4066	[Y].PGAGGYPAPGGYPAPGGYPGAPHPGGA.[P]	2347.08905
4067	[P].TGPSAASSSGPAAASGPLATSSPAYSP.[G]	2347.10482
4068	[S].GAGVGGRMCPGPGFVLCRQELE.[E]	2347.1104
4069	[M].AGPKGETGPQGYKGMVGSIGAAGSPG.[E]	2347.13468
4070	[P].GPQGHLGPQGPPGTPGMQGPPGPRG.[M]	2347.13602
4071	[V].SASPVPSGTNSPAPKKSTGSVDYL.[A]	2347.17759
4072	[S].PAQTLNDTLDDIMAAVSGRASAM.[S]	2348.12207
4073	[E].PQPQGQRGYGREGSSRTSLEGT.[S]	2348.13377
4074	[S].GAATTTAAAAASAPAPGPASSPEASPAP.[G]	2348.13646
4075	[S].PGAASTPRGGQSQQQQRGGGPQAQ.[S]	2349.14026
4076	[S].QAPAPAFSGQPQQLPAQPPQQY.[Q]	2349.16222
4077	[K].RCPTPEVQKRSTGDVPHASVAG.[D]	2349.1728

4078	[R].SAAPGGGSVAAASAAMGAALASMAGLMT.[Y]	2350.11996
4079	[P].DPAGVGRGGGAAGPTSGGGGQPQWQK.[C]	2350.12829
4080	[P].SPAPTPSSAPSPLGGSALCGGKPEAG.[E]	2350.13435
4081	[S].GPGAGGEATLLAPSAEAGGGLTCAPAP.[Q]	2350.13435
4082	[D].GGAGGNGLVGPGGSGAGPGGGLTPTAPPY.[G]	2350.14221
4083	[G].GAGGNGLVGPGGSGAGPGGGLTPTAPPYG.[A]	2350.14221
4084	[Q].PHPPGFGSISSSGALFSAGSQPAPP.[T]	2350.14624
4085	[W].RPGGNLHGSLTEAAPPHADGWLP.[L]	2350.1687
4086	[A].AQGAAAAVAAATSGVAGEGQPGPGENAA.[V]	2351.12221
4087	[K].GGGSAAAAAAAAASGGSSDNSIEHSDY.[R]	2351.99706
4088	[L].GPQGPPGPQGNAGPQGHLGPQGPPGP.[Q]	2353.14322
4089	[G].PGEGAPAPGEKQEGSRAPPEQPPA.[D]	2354.13713
4090	[E].PTPWAGDKGGAAPPAATASDPAGPPP.[L]	2354.14115
4091	[G].PSAGAAPNATAAAAAAWTNISLPEM.[P]	2354.14452
4092	[S].AMGMGHLLASVAGSGGGGGGGGPGTATGL.[D]	2355.11799
4093	[A].PATWLIPETPALPESQVAQPLP.[F]	2355.25947
4094	[S].FVFGTGPSAPSASPAFGANQTPTF.[G]	2356.12444
4095	[P].QGYKGMVGSIGAAGSPGEEGPRGPP.[G]	2356.13502
4096	[G].NHSVLSSTVTASSTDLNHKTQE.[N]	2356.13752
4097	[G].ARAETQGANHTPVPSTHHTRST.[S]	2356.15009
4098	[W].KSSEEWLPGKHPSQVKDGAEF.[Q]	2356.1568
4099	[P].TGAPSPSQAAPPSLPSAQNPTFHP.[G]	2357.15205
4100	[F].FRSHTGTVFNTVEPPDGATLPN.[I]	2357.15205
4101	[T].GPKGTSGHPGEKGERGLQGEPGPQ.[G]	2357.15926
4102	[Q].GPPGSPGQAGAVGIPGERGPPGPPGPP.[G]	2357.19967
4103	[A].PNPSADLAGGENRAGGAAAAAAAPPPS.[P]	2358.14327
4104	[A].QPGPSHPPPNPASPRAEAPGSSQP.[H]	2360.1378
4105	[D].EAAPVPCALGAEAPGGTPVGKEEGP.[S]	2361.1391
4106	[T].APDGAALTPPSPSFAATGASSANRF.[V]	2361.14696
4107	[V].QKSSSPAPADIAQTAQEDLRTF.[S]	2361.16809
4108	[H].PGAGGRERCASPLGAGAAGSQGPAGGP.[A]	2363.12691
4109	[N].TSLPMTSAVQNSTYTTSVITSSS.[L]	2363.12826
4110	[S].QGPLMGLNPRGMQGPPGPRENQ.[G]	2363.13431
4111	[P].GPAGPKGDPGSRGPMGMRGPPGLQG.[P]	2363.13431

4112	[P].PEPAAAPTSPATAGSPATAAGPATATE.[E]	2363.13612
4113	[E].RSGDAPASLHERTQGGRSHSEK.[E]	2363.15591
4114	[P].APGGNRSLSPGSQTSGFSGSLFSPA.[S]	2366.13713
4115	[P].PSAAPSSHPPASSSISIPGMGSRTS.[G]	2366.1405
4116	[G].AHTWTWLSPTGQATTPATTGPPS.[Q]	2366.14115
4117	[Q].KGDPGTPGYPGKNGPMGTPGIPGTP.[G]	2366.14452
4118	[D].PGGAEKEEKSWRPQHNPPPQP.[Q]	2366.16362
4119	[A].GSLPWTGGSAAKPGKPKGKKKLSS.[V]	2367.35069
4120	[T].GTTHTATTVTSNMSSNQDPPPPAG.[D]	2369.06739
4121	[R].APASAEVAVGDSVTLSCNATGVPVP.[A]	2369.16532
4122	[P].PTARPSEGPTTGPTGPPAAGPTGPPT.[A]	2369.17318
4123	[A].GPRGPAGPSGPAGKDGRTGQPGAVGPA.[G]	2369.20688
4124	[G].ASQPHPPGFGSISSSGALFSAGSQP.[A]	2371.13131
4125	[V].GGAKSGPEGLESFASAAAPPPGAGCK.[V]	2371.13468
4126	[S].SPGLRDGSGTPSRHSLSGSSPGMK.[D]	2371.1419
4127	[M].PPMPGPGPGPGPGPGPGPGPGHSMRL.[P]	2371.14342
4128	[A].AASAPAPGPASSPEASPAPGFPFPPP.[W]	2372.15574
4129	[N].FSFKSSSGFATAPSGSPPVFGNTP.[A]	2374.135
4130	[S].GTPTGGPSPATSSPVPPMASGGFLGF.[L]	2374.13837
4131	[P].PGSAPGPGPLSGSQGPGQQCLGQAGL.[P]	2375.14083
4132	[L].VSDQTEKVGEAQAGGVTQTRAEN.[G]	2375.14333
4133	[A].AHSPHVMIGPNGSSLGAPSPGPPGPG.[V]	2375.15609
4134	[T].GGVAASGMAASGVVPGGGFVASAAAEVQ.[T]	2375.16598
4135	[S].LSAASGGPEPPEPRTPGAGLARQC.[S]	2376.17247
4136	[P].GQPAEDSVKQEGLDLTGTATTATS.[F]	2377.13652
4137	[N].SAWVSGGVTQPGGDVHLGPGPHQH.[V]	2377.14322
4138	[T].GSVYNKTQTFDKQGFHAGTPPP.[F]	2377.15713
4139	[G].ARSAAPGGGSVAAASAAMGAALASMAGL.[M]	2377.15985
4140	[P].GHVSPTPATTSPGEKGEAGTPVAAGT.[T]	2377.16301
4141	[P].KRCNQMPLMWCPPRNMNL.[Q]	2378.0807
4142	[M].PNAAAVAMAATLTQQQQPATGPQP.[S]	2378.17688
4143	[L].GPAGPGTGGPGVASPTITVAMPGVPAF.[L]	2379.20131
4144	[Q].PPQPANPPHGAHPLSSGPQPGTAPA.[T]	2380.17927
4145	[P].NSGLYGSYPQGQAPPLGQGHPGAQ.[P]	2381.1269

4146	[E].PTGSASVSTGSRAGGAAGVGGEAGPPPE.[R]	2381.13277
4147	[S].QPHPPGFGSISSSGALFSAGSQPAP.[P]	2381.15205
4148	[S].GRSFSTADSILGCLAGRVVHMM.[G]	2381.15227
4149	[Q].AAAQPSTPAGTPRSGGGHSPAQPPSP.[E]	2381.15926
4150	[A].AGSASRSGPGGSGSSGRGGAGVPGPGSGGP.[G]	2382.1141
4151	[T].FLSSASTALSTHNNSVFGDLKAD.[E]	2382.15719
4152	[L].SPSLNTPAPVAMPASSPPGPPPAPE.[P]	2382.16459
4153	[E].KQPSQEVKMEPKMEVEAPEPA.[D]	2382.16796
4154	[V].AAPAPTERLSMPLLTDCAQPSR.[P]	2382.19043
4155	[C].PEAQLLEAQALEPPSPEPEPQL.[L]	2383.20275
4156	[A].AAAAGQPGTAPSGVPGAPPLPGMAIVK.[E]	2383.28023
4157	[D].VGGAAAAPGGGAGGSRELEMHTISSK.[V]	2384.1623
4158	[V].AASSPALELPDLLLLAGPAKENGH.[L]	2384.282
4159	[G].QQFDQISNRTGKQEAQAGPWP.[L]	2386.15345
4160	[D].SKTASPACRSGRGTGAVGNSEKHA.[D]	2386.16403
4161	[D].GQPGHKGERGYPGNAGPVGTAGAPGP.[Q]	2386.16468
4162	[F].LHMLSSRSSGIQVGEQSTVQEP.[A]	2386.16671
4163	[S].PSGTRFNFSQLASPTTVTQMSL.[S]	2386.17074
4164	[G].ERDLPGWSRYWISGNAASGQH.[D]	2387.12757
4165	[P].PSQHAAPSPVQHQAGQAPHLGSGQ.[P]	2387.15993
4166	[V].LGGGGPGGGGGLGGPGGSVPFKLEENY.[D]	2387.16261
4167	[L].PLSEQQVDWPPQRASSGLTYQ.[D]	2387.16261
4168	[S].SGGRGVMLDSEPRPVERGGAGRM.[T]	2387.16667
4169	[P].APNAGVLSSLASDGGPGSASTFTNPL.[L]	2388.16776
4170	[A].KSVDHSAGIDVGPVADDPSSLPQP.[T]	2388.16776
4171	[D].AARDAPEAAGAGLEAPLLPAGNRLS.[L]	2388.263
4172	[L].AAHSPHVMIGPNGSSLGAPSPGPPGP.[G]	2389.17174
4173	[L].KEQPQALNFGGIGMVIGHEITH.[G]	2392.20779
4174	[P].AGGKASTHVTSSSLTIWPSPAGSPP.[P]	2393.20956
4175	[G].QRQGASEQQPGDRGPGGHPLAPGP.[P]	2394.16574
4176	[I].NAYFVKYRKLEDGVGVVGGWH.[T]	2394.23532
4177	[A].ARLPTSDGSASKGKQQTSEPVHI.[L]	2394.23718
4178	[Q].RQEEPPPGPQRPDQPATAAAAGPG.[D]	2395.17491
4179	[P].PGLPGRNGAPGEQGFPGPRGEPGPP.[G]	2395.19017

4180	[A].LPLDQSQAGWPPPATSLQPASFS.[G]	2395.19285
4181	[P].KGDPGIAGSPGLPGPVGPAGAKGMPGH.[N]	2395.21869
4182	[P].GPGVSFSPGPTPTPAPTAGSFAGGAGGP.[S]	2396.15171
4183	[M].LGGGSSPLPLPAGGSSSVGGSGGFGSLH.[Q]	2396.18408
4184	[P].PGSPGPRGNAGGPGLPGPPGPPGPPGQA.[A]	2397.20582
4185	[W].LNWAFGRECLGVGPASRNSGLH.[N]	2398.18331
4186	[P].RPADSGGKQGRDQKHEPLHSPT.[S]	2398.19704
4187	[T].GVQAQPGEATSGPPGIQPGQEPPVT.[M]	2399.18374
4188	[E].PGLVGMQGPPGRPGPVGQMGPVGAPG.[R]	2399.19585
4189	[L].AEGVAGPEKGGGSAAAAAAAAASGGAGSDN.[S]	2400.1022
4190	[P].QGPPGPQGHLGPQGPPGTPGMQGPPG.[P]	2400.15134
4191	[P].PAASPVMPPQTQSPGQPAQPAPMV.[P]	2400.16863
4192	[F].ASHFLQGGPFPLPYPGPGAYLDV.[G]	2400.20229
4193	[K].QNGGPLTPGTSPTQLAAPVSFATST.[T]	2400.20414
4194	[L].HTVSASAGAASRDREPAPRPAAPQ.[N]	2400.21269
4195	[V].NNGVPGGAAAAAAATVAAASATTAASSSL.[A]	2401.19537
4196	[S].TAAPSGALDAAAAVAAKINAMLMAKG.[K]	2401.25778
4197	[D].NMLLAEGVSGPEKGGGSAAAAAAAAASG.[G]	2402.16163
4198	[P].PGTSDLGFPLDMTNGAALAANSNGI.[A]	2404.14491
4199	[Q].PGAGQPGMVQPDIGQPDLVQSGAGQ.[A]	2404.15615
4200	[G].SPPGGSRHGLGPGPSPSPSPEEPGAP.[G]	2405.14803
4201	[P].KGDDGIPGQPGLSGPPGPKGEPGHPG.[T]	2405.18441
4202	[Q].PGLMAQMATTAAGVAVGSAVGHTLGH.[A]	2405.20641
4203	[E].RGGGAGGGRGGVVISAVWTVRGGAGGH.[E]	2405.26573
4204	[A].VNPSGSPAGPAGAAAACSPPRNDRE.[P]	2406.12149
4205	[G].VGVDMLSLPGGPEAGGFAPLLDFM.[Y]	2406.17198
4206	[A].VGEPASGTPATVGSLPSSESFLGMK.[A]	2406.18572
4207	[Q].QPKCKSHSSRAAAYDLLVEMV.[K]	2406.19043
4208	[P].ASGPGPGPGLGPGPPDEKLEASPAPGP.[S]	2406.19358
4209	[L].PGPKGETGDPGPPGLPAYSPYPSVA.[K]	2408.17687
4210	[K].HMRVMAGALEGDLFIGPKAEEH.[R]	2408.18495
4211	[G].SQSPKPLMVHMRKYGGITSFE.[N]	2409.20535
4212	[Y].AYTGSVAISGALAGSPAPLGPGTEPPA.[L]	2409.22963
4213	[A].PGPASEIKPEPQVQESPSLPAPGP.[P]	2409.22963

4214	[P].QIQELLHSEHLGLNELEAPAPA.[G]	2409.24086
4215	[P].VQTVRGSHAAALRVEVEQPPHQ.[T]	2409.27456
4216	[V].GSPATVTFQQNKNFHQTFATW.[V]	2410.15747
4217	[L].GSPEKEEVAPEIPNPAPDTAPPAP.[D]	2410.17726
4218	[G].HLDPGFLASEKTSAGNAPLNEEI.[N]	2410.18849
4219	[A].ASAVPGSGAAAGALASGGSKEEFVATF.[K]	2410.18849
4220	[S].PASPPSSPRTKDPPTGSPPASPGPQ.[S]	2410.19973
4221	[R].GGERGAPAQSRARSPASGAPTSWR.[R]	2410.20828
4222	[V].QPGVDPANATGLDGREPAPHLLQG.[L]	2410.21096
4223	[A].PGSATPAVGSAPAIADEKKEEKKE.[E]	2410.24601
4224	[P].VLSVTQTLTTGPDSAVSQAHLTPS.[P]	2410.24601
4225	[N].PGAPGPRGPKGERGLPGVQGSPGDIG.[P]	2410.25858
4226	[H].QPYPTPAAANSMDTDYPGSACSI.[Q]	2414.0275
4227	[E].SGVSAAGGGPAGAAGGAAGGGPAAGPADHGLA.[G]	2414.14434
4228	[G].PAAHVYPAGSQVMMIPSQISYPA.[S]	2415.18355
4229	[L].PGAAVSASQAPGFVSFAGRGDIQPQ.[L]	2415.20515
4230	[G].SLICNVGAGGPAPAAGAAPAGGPAPATAA.[A]	2415.20852
4231	[Q].GPAGPPGPPGPMGPPGLPGPMGIPGSPG.[H]	2416.1788
4232	[K].ADTDKTAVGSSVAPGNIATSPSSPTS.[P]	2418.16307
4233	[K].KPSGDGGKAGGQGAAGVDQWLEVFS.[E]	2418.16843
4234	[F].LPQCLYPGAIKKAKGADQLSPY.[Y]	2418.28498
4235	[R].SGGGRPAAANAARERSRVQTLRH.[A]	2418.29334
4236	[Q].QVEQQPSASQAGVQQPPATSTGGPA.[A]	2421.16407
4237	[H].PPGFGSISSSGALFSAGSQPAPPTFG.[T]	2421.17212
4238	[A].ASVQEGLPGSLGRAMGGQQEAPPAP.[E]	2421.1827
4239	[S].RQGCLPQTPGAPRQETSGRMPP.[V]	2421.18741
4240	[T].SAQPAAATPASVSSPAGSPGPPGSTASL.[S]	2421.18922
4241	[S].AQPAAATPASVSSPAGSPGPPGSTASLS.[T]	2421.18922
4242	[-].MIPSGPSTGSTGAPAVAEATEQGPKNP.[R]	2421.18922
4243	[M].IPSGPSTGSTGAPAVAEATEQGPKNP.[R]	2421.18922
4244	[L].EFHRNLGELVEGTGHLLEAHY.[A]	2421.19458
4245	[S].PKMGGEIQAPDLDISSPGINVEGP.[D]	2421.19662
4246	[A].GNQVQAGKQSHIPYSQQRPSGPG.[P]	2421.20179
4247	[L].PGPGTTLSPMGTNAVTSHLNQSPAS.[L]	2422.16671

4248	[L].GGARGPGDPPAPPEPPYPIPEEGAP.[R]	2422.16736
4249	[L].NKAMTIHEAEAFVGAERCIMK.[T]	2422.16758
4250	[Q].EENMILGLPGASPATGVTNPQGTAP.[P]	2422.19186
4251	[A].VQSAFYPQKSFPEKAPANGVEQ.[T]	2422.20375
4252	[P].EAGPGTPKSVRSSGMTPGTPRSPAP.[S]	2422.21433
4253	[R].PAAEAAAAAGGGGATAAAARGGEAAAEVTG.[W]	2425.17022
4254	[P].AAAPTSPATAGSPATAAGPATATEEAKG.[R]	2425.18414
4255	[R].KGGPSPASGRPSESKETTGAGLWAP.[D]	2425.21063
4256	[A].VSSSQLNTIVTMNHHHPHPHH.[A]	2426.15307
4257	[P].GPEAALMPDPGPGEVPAAADDAPLPA.[L]	2426.15442
4258	[E].AEEARRQLAALGHTEPPLVAEAP.[W]	2426.27865
4259	[S].KYFGSIDSSEAEAGAAQARAEPGD.[Q]	2427.10589
4260	[C].SPGAASVSPSAGWAESAMPVSPPGMV.[D]	2427.13191
4261	[L].TGLGGVMAQAGFNGGNLTNFFSLPG.[S]	2427.17616
4262	[A].GRAAESGRLNFGIPGSWPEAGGGLA.[N]	2427.21638
4263	[R].TPSGGGRAGAGPHLIRLQSLMFTS.[T]	2427.25614
4264	[I].QEREVVAASHELGQDYEHVTM.[L]	2428.11976
4265	[D].EEPWGPIGKDPTPSMLGLCGSLA.[S]	2428.15231
4266	[P].NGQLDYEIINGNKEHSFSINH.[A]	2429.14803
4267	[K].PATSQSPQVTSPPTQTPHSAPDPT.[V]	2429.15792
4268	[S].PGSTSSVTSINSRAPEVPSESGSPV.[Y]	2429.17905
4269	[P].GPSPSLAPSPLKLFPSQAAHQLGE.[G]	2429.28233
4270	[E].QERQHNYLKDGPYITAEEAVA.[V]	2432.18408
4271	[M].PSSMFLPAAVPDRDGSSSVEEAGK.[Q]	2434.15548
4272	[K].AEGGDGKAEPEKTLGFPSTDSLEV.[S]	2434.162
4273	[H].PGAGGRERCASPLGAGAAGSQGPAGGPA.[P]	2434.16403
4274	[V].GARSAAPGGGSVAAASAAMGAALASMAGL.[M]	2434.18132
4275	[L].RQSMVGADVGVWTGETIPVRTC.[F]	2435.18059
4276	[K].WHEGLYRGPPPGGQCIWKPNS.[M]	2436.16659
4277	[G].SKSSAGTGVSAAAGSAGGGGAAATTSGGVGAG.[P]	2437.15496
4278	[P].QREGAQPTTGREREAHGNRATD.[S]	2437.16753
4279	[K].DQKTPASSVASVGGPSASSSTSAVAST.[S]	2437.16888
4280	[S].TPPLAASGMAPGPFAGPQAQQAARE.[V]	2437.19287
4281	[I].LSGMGNGTIASSAALNSAASAAAGMTVG.[S]	2438.165

4282	[Q].AGDPGLVSAYGPGLEGGTTGVSSEFI.[V]	2438.17217
4283	[G].PKGEMGPVGPAGNPGAKGERGSSGLD.[G]	2438.17286
4284	[Y].RNRQDAGAAASAAVEELSESVHGL.[C]	2438.20185
4285	[Q].VGWQEAQKSPSVTTPPSCPAPLT.[P]	2438.20204
4286	[H].PGARAGGASSPSPVVFTVGSPPSGTTP.[P]	2438.23103
4287	[G].RLCNKSSAGPDGCGSMCCGRGH.[N]	2439.97915
4288	[Y].PYPSPVSPTGAKMGSHHGVPGTAGH.[V]	2442.1619
4289	[G].SREAAAADATTTAPGADRPWEGVAA.[G]	2442.1644
4290	[N].GFSSPGSYKTNAAAAAAAAAAAATVNM.[G]	2442.1718
4291	[T].GPTSSPTAPPASATSTGAPTSTGRPST.[T]	2442.1743
4292	[G].PTGDKGSRGDPGTPGVPGKDGQAGHP.[G]	2442.17564
4293	[A].PTQSPELTPSGPAPAGPASAPETNAP.[N]	2442.17832
4294	[S].QVSMDTGALHVVIVGGGFGGIAAASQ.[L]	2442.24457
4295	[S].QRSPSKHGGPSAPGALQPLTSGSAGP.[A]	2442.24841
4296	[A].QLVEATEELRCGRARGGVVGRE.[I]	2442.26301
4297	[P].GPSPGAMLGPSPGPSPGSAHSIMGPSP.[G]	2443.13806
4298	[S].SFVFGTGPSAPSASPAFGANQTPTF.[G]	2443.15647
4299	[A].AEAAAAAGGGGATAAAARGGEAAAEVTGW.[P]	2443.15965
4300	[E].GSPGLRFEGSAGGLRFEGPGGQPVGG.[L]	2443.21129
4301	[Y].PGAGGYPAPGGYPAPGGYPGAPHPGGAP.[S]	2444.14182
4302	[P].KGDPGSRGPMGMRGPPGLQGPPGSPG.[Q]	2444.19216
4303	[M].PSSNSRPPACLAPVALFLALLLH.[R]	2444.34825
4304	[L].QGPGPGPGPAPGEVTAASAGYLGDAPGP.[S]	2445.16809
4305	[M].PGAVPGSGHPGVADPGTPLPPDPTAPS.[P]	2445.20448
4306	[I].SGAAAPAPAPSGLGYGPPTSLASASGSF.[P]	2446.18849
4307	[K].PGSLSAEVGLETGDQIVEVNGIDF.[S]	2446.19839
4308	[L].AGGVVGAGMAAAALAAEAGMVAAGAAVGAT.[G]	2446.20647
4309	[T].PAAEGVGAAANAAATSSTGTGGVAASGMAA.[S]	2447.14671
4310	[D].ARPACAGAAGRQSAGPQPSPEGVTGP.[R]	2447.18443
4311	[P].GPAPGFAAGQQTPATGFRVGHFIY.[S]	2447.22549
4312	[R].PGSASSQKPAPDTGASGNAAPHSDIQ.[L]	2448.13858
4313	[V].PGSPGFPGVPGSPGIMGFQGFTGSRG.[D]	2448.17649
4314	[S].LNGLMYGAAQPGGCMDNRTFPY.[P]	2449.07335
4315	[G].PGGRRPSLGPMPPLSSRVSFSGLP.[L]	2449.31326

4316	[I].LDGGAPGPQPHGEPPGVPDAVDGVTN.[G]	2450.15826
4317	[M].LQPPADTSQSPVNTPPSPGHAPASP.[G]	2450.19464
4318	[F].PGSPGEKGEKGSTGIPGMPGSPGPKGS.[P]	2450.19801
4319	[P].PGPPGGPGMPPGGRGRGRGQGNWGPP.[G]	2450.20069
4320	[K].KQENFNKRIAKIQALWRGYS.[W]	2450.34152
4321	[L].AGAATQTFTRAGKKKKVGSEKSGAA.[S]	2450.34739
4322	[A].EGVGAAANAAATSSTGTGGVAASGMAASGV.[V]	2451.14162
4323	[Y].KNYFTAGAHWLTMVFLILVNI.[T]	2451.32572
4324	[I].LGGYGTTSTAGGKPMEISGTHTEIS.[L]	2452.16604
4325	[I].FYKGICNVSSSWRPTGAPSPSGP.[E]	2452.1714
4326	[P].GPLPAAEHYGAHQSLSLKNMMAE.[N]	2452.17477
4327	[H].SIPTSPSHGSIAAYQGFSPQRTY.[R]	2452.18916
4328	[Q].QPGEAEPLTPTYNISANGLTELH.[D]	2452.19906
4329	[L].GPGPAAAFSVSVERSLAAESGLDTY.[R]	2452.19906
4330	[D].GPSGALLLRLVQLAGAPEPAEPAQP.[G]	2452.35583
4331	[E].KDAAAEGKEAGGDSEEDGENVFEV.[E]	2453.05866
4332	[P].GTAGPRPEAAGPGTASSAVPPTEDFL.[P]	2453.19431
4333	[L].QPAGALMEPQPSPRSLAEGFLQE.[E]	2453.21293
4334	[L].PQVNQQMAGLSLGGVASAAGFGPPPS.[T]	2454.20818
4335	[E].PGPGLSSTSPVGEPSAGLGPGPEDVPP.[F]	2455.19872
4336	[G].QPGHKGERGYPGNAGPVGTAGAPGPQ.[G]	2457.20179
4337	[K].GPVGAPGLGGPPGEPGLPGIPGPMGPPGA.[I]	2457.25949
4338	[T].IALMAWDSPMLGPLALGGQPPPQP.[P]	2457.26688
4339	[G].PGDASLPGPAPGSARPPSLPQGADMD.[F]	2458.16671
4340	[Q].KDQPDKIAEAPASEAVAAPKDAHV.[S]	2458.25724
4341	[L].RSPAGGGTAGAGATGDGGSLLPASNFAAA.[P]	2459.19095
4342	[W].GTGPQPSAPFPDPPGWRNIEPEL.[P]	2459.199
4343	[R].RAGGGGGGGVQNGPPASPTMAHEATPL.[P]	2460.16844
4344	[T].APDGAALTPPSPSFAATGASSANRFV.[S]	2460.21538
4345	[S].PGGSLPGAASASSLLQGLSFSLQDIS.[S]	2460.26166
4346	[P].SPGGSLPGAASASSLLQGLSFSLQDI.[S]	2460.26166
4347	[P].LPTGEAGGPPSTREAELKLRLRL.[V]	2461.38853
4348	[S].GPAAASGPLATSSPAYSPGLSSPGQAY.[S]	2462.18341
4349	[P].AASTPAGPPSGGASPTPPAASPSGGSATR.[P]	2462.19062

4350	[Q].NTPAPGVGAAGGSLSFGASSTPAQGFVG.[V]	2462.19464
4351	[P].GPWPPGAPASEALVAEFLQDQNAP.[L]	2462.19866
4352	[A].LFSAGSQPAPPTFGTVSSSGQPPVF.[G]	2463.21907
4353	[S].AAGSQQPAGPPAVQPQAQAQPPAQPA.[P]	2463.23751
4354	[E].AFEAGIAGGGQGVLGGAGGGNGPGDPAVPG.[D]	2464.18514
4355	[G].PGPHPASAPAATQEAPSALPSPQMPG.[D]	2464.19253
4356	[L].TSTSRGTGTPVTHTTTATPTGPHTP.[F]	2464.20627
4357	[G].PFGGAVSAAGLTQMPAGNVFTTAEGL.[F]	2464.21769
4358	[P].QGVVMAASPGSLHSPQQLAEEATR.[K]	2464.2249
4359	[V].DLPEVASGGLEGKLKGPKIKAPEM.[I]	2464.34797
4360	[S].PGGSPSGRSVKSESPGVRRKRVSP.[V]	2464.34913
4361	[G].TPGQPGFPGPKGEMGVMGTPGQPGSP.[G]	2465.15879
4362	[N].SYVHPQAPHLYPGPSPMYPLPT.[Q]	2465.19583
4363	[T].GQPNITPSSSPSPVPAATNQVPTAM.[S]	2465.19768
4364	[G].GLGPAGNAASTAGPFPFHLSQHMLA.[S]	2465.20304
4365	[L].PGQQPLSHEPPPAMLPSPTPLGSN.[I]	2465.21293
4366	[Y].RAAQSPEPPAASRSADLDSSLPEL.[A]	2465.22667
4367	[E].AGGPPSPPGSPTRSPLAASPEPAPAQA.[A]	2465.24193
4368	[P].RAGAGVEGGAFLGVQTSALGGEGPGLH.[Q]	2465.25316
4369	[L].QPAALTDQPVTPELTSRATRGRT.[Q]	2466.30592
4370	[L].MPVAAVSVHLLAGNGTEVPLSGPIH.[L]	2466.31734
4371	[C].RADHGLLLERAAAGSPRSPGPLPGA.[R]	2466.33241
4372	[V].KLEAGSFSGTLGAIINILDLFSTV.[P]	2466.34902
4373	[G].PSAGAAPNATAAAAAAWTNISLPEMP.[L]	2467.1922
4374	[R].QRPSAGEAGTLEGVEAALFYQCL.[E]	2467.1922
4375	[A].PGPEGPSTPRSQGPQVTLGGPEGHGA.[Q]	2467.19604
4376	[G].AAGEGISAAPASPRSPKAGTSEGPVDS.[V]	2467.20593
4377	[V].PSLSAPGQGTSSTNAVGGAVSSQAAPAP.[P]	2467.20593
4378	[G].PPGGAFGEAFLPFPPHQEAAYGLP.[Y]	2467.20811
4379	[T].GEPGPRGFPGEKGMQGVPGLNGQKG.[E]	2467.21467
4380	[I].AGAPTQYPPGRAGPPPPMGRGAPPPG.[M]	2467.22992
4381	[T].GGVPGCSRLSNREGWAGRTWQH.[Q]	2468.17487
4382	[G].SASVSTGSRAGGAAGVGGEAGPPPEREG.[S]	2468.17603
4383	[E].AAAAAGGGGATAAAARGGEAAAEVTGWPAG.[A]	2468.19129

4384	[P].PGNVHAEATNSTTIRFTWNAPSP.[Q]	2468.19531
4385	[M].PPMPGPGPGPGPGPGPGPGPGHSMRLP.[V]	2468.19618
4386	[D].PPNGGQARNGAGGGPRGQTPNHSQR.[D]	2468.19984
4387	[F].CGHLAAVGGAVGAGLMGLAGGVVGAGMAA.[A]	2468.22068
4388	[A].GPRSGGGRNATTAMPPPVPNGNLHP.[H]	2468.22115
4389	[S].PASSQPGTVTSYGPTSSVALGFTSLG.[P]	2469.21437
4390	[D].KGTQSEGSTPLTTKGRSEKASMTS.[L]	2469.22496
4391	[A].ARGTGAAGNAARMSASASGGGGGDSDSSS.[S]	2470.06074
4392	[S].EEENCEKKEQGAQQAFVFGQN.[L]	2470.09394
4393	[T].GSPGPKGSPGFPGIPGPPGQPGPRGSM.[G]	2470.22959
4394	[H].LSPVMSRSHGSVPPGSPAYGGHPVP.[A]	2470.22959
4395	[D].RDVEFLIYYSAVHTPSVVVEM.[G]	2470.23227
4396	[R].PVSGSGPPGRLVSGPGRSISGPAPAGR.[P]	2470.32733
4397	[E].SGVSAAGGGPAGAAGGAAGGGPAAGPADHGLAG.[R]	2471.1658
4398	[S].AEIPAEASASVEALAQAEAPAQYPS.[E]	2471.19364
4399	[T].ATPGAAGGATAASAAASVLGGSAAPATAGDT.[T]	2471.20085
4400	[S].VSPTQHGTLGSGRSSDKGPSWSSR.[S]	2471.20219
4401	[L].AGRPEPGYERMDQFTVSVEHVA.[E]	2475.17213
4402	[Q].DPGAVPHPGPPPHWQPESVGQAFA.[R]	2475.18402
4403	[L].QDLQTLAMPGTFLLMAPTGLGHM.[P]	2475.20805
4404	[E].PGTTQTPEQAKPVATPGTTSPGPTPG.[D]	2475.23617
4405	[P].PGSKVGADQSTSVPPLSICRHGGPA.[R]	2475.24088
4406	[V].SASPAPGTSAVSPRIEAVSVTPEHAG.[V]	2475.24741
4407	[L].PGPGGLAEVAGTCVPHAHVSPGPSGAP.[R]	2476.20377
4408	[A].GPAPHPQWAGQPSVLDSINPDRH.[F]	2476.21163
4409	[K].KESFAPGTMYKPFGKEAAGTMTL.[S]	2478.20434
4410	[P].AAPTQPSTPASSSGQTPTPTPGSVPSA.[S]	2479.1947
4411	[S].EHFIGLAGSGAASGFAVGAWEFNAL.[L]	2479.20408
4412	[M].IMEIPEAGSAGNISIYERIPGDF.[G]	2479.21735
4413	[S].REGTALNNSNSSLLLMNGPGSLFA.[S]	2479.22456
4414	[G].RQSAFQYLQSTAAQPAPESSVRG.[R]	2479.23242
4415	[S].QPDKPSAPAAAAAAAQPPASHGPERS.[Q]	2480.22767
4416	[K].SVPHFSAGAPRANVTSLSLVSNRI.[H]	2480.33683
4417	[G].SPGFPGIPGPPGQPGPRGSMGPVGPSP.[D]	2481.23434

4418	[E].LGTDSRPALPGAPARGAHAVARAQAA.[L]	2482.33856
4419	[P].GPGVSFSPGPTPTPAPTAGSFAGGAGGPS.[P]	2483.18374
4420	[L].LSMPGAQGAPAAGPEPPPATASPEGQP.[K]	2483.18711
4421	[P].SPGPGPSPGAMLGPSPGPSPGSAHSIMG.[P]	2484.1646
4422	[P].GPSPGAMLGPSPGPSPGSAHSIMGPSPG.[P]	2484.1646
4423	[T].QDGLADLAVGAQGHALLLRTRPVL.[R]	2484.40452
4424	[V].AATGRSCSSRVATVTATSADPFNTG.[K]	2485.17359
4425	[G].PEAAAAAEGVSGKQPPGLLLLGQSLL.[L]	2487.38171
4426	[A].ASTPAGPPSGGASPTPPAASPSGGSATRP.[S]	2488.20627
4427	[-].MGTAGLTHASPPSAITPGMSPSPPPL.[S]	2488.22106
4428	[K].MVQRSGPSGPPGPKGDDGIPGQPGLS.[G]	2488.2249
4429	[L].AAAERDLAPAFLEAFATAALEPGDA.[A]	2488.23544
4430	[G].SPGTPGSKGPQAIPGESVWIALLLL.[G]	2488.38099
4431	[R].RSLRLGAVFPRTPAATTPPAESPP.[A]	2489.36232
4432	[L].FRRSSGGGGGGSAAGARGAGAGAAASQEL.[N]	2491.21448
4433	[Q].AGKEEDRLQQNVHNGVNQAGKEA.[N]	2492.22365
4434	[K].EPPPAHVDAAAGAVPPSPPAAALGATC.[A]	2492.22383
4435	[V].RAGSPGAPLFSAEAASGASHPAPSLW.[A]	2492.2317
4436	[V].SAAPGPPSLAPVGASPSAFGFNSIPVP.[I]	2492.282
4437	[P].GLQGPAGPIGPQGLPGMKGEPGLPGPP.[G]	2492.2966
4438	[G].TLGPVMYGKLPRLEADSGPGHSLP.[P]	2492.2966
4439	[H].PPTSAAAPVTPLRPPGLGSASLHSGGP.[A]	2492.3256
4440	[E].PGAGAGDLQGLASDLVASGSQAGGGRGAP.[G]	2494.22807
4441	[L].QRGTMASQTAVLGSPGPGLQGSAPNP.[R]	2495.23071
4442	[A].GPAGAGGAAAVATGPQALFSGGADLLGLQ.[A]	2495.28888
4443	[E].FGISPSDIPFSQGSGSRPDLSPSY.[E]	2498.18341
4444	[H].PGSAVSAAPGTPFEGGNKFQTLDNH.[Q]	2499.18989
4445	[P].GPFNQGPPGAPPHAGGPPPHQYPPQ.[G]	2499.19525
4446	[T].KFTMDCVVPTIHVYEHHENK.[W]	2500.17478
4447	[R].GGAPNVAVVMVDGWPTDKVEEASR.[L]	2500.21366
4448	[N].VNNGVPGGAAAAAAATVAAASATTAASSSL.[A]	2500.26378
4449	[V].GHLPPFSHSGHILPTPTPIHPSSS.[L]	2500.27317
4450	[Q].VPAGGAGGGAGGSGPGLGRAGALSTGALPPL.[A]	2500.32666
4451	[A].APWAAATSSPPPSADEEGMLLAGGAM.[D]	2501.1323

4452	[P].GPRGMQGPPHPHGIQGGPGSQGIQGP.[V]	2501.22148
4453	[Q].GQKGERGPPGESVVGAPGAPGTPGERG.[E]	2501.24914
4454	[Q].GAPGAMGKTAGPGEKLAVLRNSPGQH.[G]	2501.30415
4455	[P].PTVCVTGPPTARPSEGPTTGPTGPPA.[A]	2502.22931
4456	[G].GGGERTPAPGALEPDAAATRAAPNPAS.[L]	2502.23315
4457	[A].GAHFRAGTGGGPVASQNSLIQTVDY.[L]	2503.23242
4458	[Y].WGSITASEARQHLQKMPEGTFL.[V]	2503.23982
4459	[I].PSVGSPAGSTPLPPDSTGPNSTPNNR.[A]	2504.20118
4460	[T].PTFGQNTPAPGVGAAGGSLSFGASSTPA.[Q]	2504.20521
4461	[H].KGTSSGATMAPASKATPSSVPSSETAP.[S]	2505.21372
4462	[S].QPNHVSSPPPQALPPGTQMTGPPGP.[P]	2505.21908
4463	[S].RSPAASGAAASPPPISNTTTQGNASPP.[L]	2505.23282
4464	[A].KSQGKGSSGSEIYERLTPGQPGNQ.[L]	2505.23282
4465	[T].WDILNLAEALLEQAMIGPSPNPL.[I]	2505.30577
4466	[T].SAQPAAATPASVSSPAGSPGPPGSTASLS.[T]	2508.22125
4467	[L].GTKTPTEGTGPPARGSQPAEPGASEK.[E]	2508.23248
4468	[-].EAAAAPTAAPGPAQPGHVSPTPATTSPG.[E]	2509.23176
4469	[P].PGLPPPPPPGMLMPPMPGPGPGPGPGP.[G]	2509.24404
4470	[R].GEEARAPPTGEPGSALFPGPAMGTAAA.[V]	2510.19801
4471	[T].GPVQTTTSSTHAAPSPTHATSHSHI.[T]	2510.20185
4472	[A].GEKAPTGADKGGGLALGSGAGGLAEGAGAL.[A]	2510.28452
4473	[T].QPGLPSSGQGGAASPGSSLGLYSPIEP.[G]	2511.23617
4474	[P].GPAPHHHPPPHPSSGLQGLQAQHQ.[H]	2512.2341
4475	[P].GPSPLSATQGATPQQPPVNSLPSSHG.[H]	2512.24265
4476	[D].PGVGGTGLEQGPSAGAASAGPQVSLYQG.[A]	2513.22667
4477	[G].TAPQTAQQAGPEAAGHRASLGRAPGD.[C]	2515.23963
4478	[G].VYHVPHGSTTSLKSTEGGAAGTTSGL.[A]	2515.24232
4479	[P].SPGVTSAGAQPTKTPAAPSGFSFPSPA.[V]	2515.24634
4480	[Q].PWGPPAPGPRPQPEPEPAAGEPGRA.[A]	2515.24768
4481	[R].MPLAAGGGAALDQDLERARSCLRS.[A]	2515.2504
4482	[P].RNTYQSAMGKQAMGVYITNFHV.[R]	2516.21731
4483	[G].DAGLQPSPGTTLGPPAASTPAGPPSGGAS.[P]	2516.22634
4484	[R].PGTSAAARAATSASARADMSATARPGP.[S]	2516.22702
4485	[P].PGSGPLEVTTDSLPNGPALADGPAPVS.[L]	2516.25149

4486	[P].GPPGPPGPGPGGVAPPAGYVPRIAFYA.[G]	2516.30849
4487	[T].EPNAAKNQTSSASHLLDSTVVCST.[I]	2517.18857
4488	[G].ERLDQAQFPPEAAHPQYTPQPP.[C]	2517.21571
4489	[V].PGTSAPRGTAPQEPSRGPGVAAMDIP.[P]	2517.25145
4490	[A].PGARPRCCVPTAPPRAAFQDFF.[F]	2518.22306
4491	[A].HVYPAGSQVMMIPSQISYPASQGA.[Y]	2519.20574
4492	[S].QAPGENSRNSILASSGFGASLPGSSQ.[A]	2519.21208
4493	[K].VGSAALEPGGAQQSLGGGWAGGGRAGPGP.[P]	2519.23857
4494	[G].TVSYSLFQASEEIKQTFSINEV.[T]	2520.25043
4495	[Q].EVESLMPTATLEGKTAHTAPPPMP.[R]	2521.23129
4496	[-].MIQGGPNQREGYAHMLPGTPERAQ.[G]	2523.21573
4497	[M].IQGGPNQREGYAHMLPGTPERAQ.[G]	2523.21573
4498	[S].PGAMLGPSPGPSPGSAHSIMGPSPGPPS.[A]	2524.1959
4499	[G].PGPSPGAMLGPSPGPSPGSAHSIMGPSP.[G]	2524.1959
4500	[P].TPPPPYSAFQLQQQQQQLPAPC.[G]	2524.22892
4501	[V].SEPVELSAAVEAVEQGVPEKEETP.[P]	2524.23008
4502	[V].AFMFRNPPAVAMASPASAPAPAPSPA.[P]	2524.24755
4503	[A].GQAGRPGNPGHQGLAGVPGMPGTKGGPG.[D]	2525.24261
4504	[W].AAAGLADGARAAGHAGHGPAHGGLAGHGAA.[A]	2527.24097
4505	[R].ADRNVKEASLQVGQHPRKTQPAP.[A]	2527.34879
4506	[F].RQLLTPSSQPAAGGPSRAAGSPAPPR.[A]	2527.34879
4507	[K].KAQEEAPQQPEAAAAATTPVTPAGHG.[H]	2528.23757
4508	[E].SEALQPSAEAVAANPGAMLELGPPHG.[V]	2530.22423
4509	[L].NTPAPVAMPASSPPGPPPAPEPGPPPS.[V]	2530.22825
4510	[Q].QSLPSPTSAPPGTPTQQPSTPQTPQ.[P]	2530.24199
4511	[T].PGSGVTGTPMRQRPDLGSAQKCLT.[S]	2530.25007
4512	[H].PGAGGRERCASPLGAGAAGSQGPAGGPAP.[D]	2531.21679
4513	[G].KLQDSSVGGQGAQGSQPKPAAAGGPHT.[L]	2531.2597
4514	[S].CVRFGGAEVIEGLMRVAREHAGT.[Y]	2531.26057
4515	[P].RNTYQSAMGKQAMGVYITNFHV.[R]	2532.21222
4516	[V].APSGRGEPGPPSEAVFARDPMRPPG.[P]	2532.24121
4517	[R].SPGPSPFRPASGTPEAGRLEEPPAAG.[P]	2532.24774
4518	[S].LADNVESPQGALPGQATPENPTAQQ.[I]	2533.2165
4519	[E].PGDVSAGPRSGGGRNATTAMPPPVPNG.[N]	2533.22121

4520	[T].PGSGKCLAETPGAATHRVSSPLWGP.[P]	2533.26162
4521	[R].AGVGVDMLSLPGGPEAGGFAPLLDFM.[Y]	2534.23056
4522	[Q].ATTPNPVPSSTEAQGVAGPAAEIPASGG.[H]	2534.2369
4523	[V].SAEKQNQAAGPPPPNQASRSYPAAP.[Q]	2534.23824
4524	[T].GQVPGQETGLSDLAWAVGLQAYHH.[W]	2534.24226
4525	[V].PGTAPPAGAPSRAKPTATPPRPSGTPP.[A]	2534.34739
4526	[P].SSPQRPPTPEETKPASPGAQEDND.[D]	2535.15938
4527	[F].FHPNGPRFGQSPSCACEDPSAAF.[T]	2536.07685
4528	[Q].GEKTEAPGGPQAYVSPSEFFPFPP.[G]	2536.20308
4529	[M].GPVGPAGNPGAKGERGSSGLDGKPGYPG.[E]	2536.25389
4530	[S].KMWDAVLYRMSVAASRGGQAGPGAG.[A]	2536.25476
4531	[E].EVGAAGAAFATTALGVSNAAMEELLTA.[T]	2536.25994
4532	[G].AGEPKGGPGPGSGGGAGTGAGAGGPGTGHLPP.[G]	2537.21275
4533	[P].APAAEAGLEKDGGSLSQTPGTPPGPTM.[A]	2537.21881
4534	[A].EHTAVAPTSSTKSTSVNFGSLSMTP.[F]	2537.21881
4535	[P].GAPAALTDTGASDLGSPGPGSRRGGSPQ.[T]	2537.23388
4536	[E].PTGSASVSTGSRAGGAAGVGGEAGPPPER.[E]	2537.23388
4537	[P].GPQGNAGPQGHLGPQGPPGPQGHIGPQ.[G]	2537.23924
4538	[Q].GPAGPPGPPGPMGPPGLPGPMGIPGSPGH.[M]	2537.24279
4539	[E].PGGAQQSLGGGWAGGGRAGPGPPEHSVP.[P]	2538.22326
4540	[P].SSPVPTPSPSTPAPPTSSPSSPAPPNP.[S]	2538.23584
4541	[A].AEAAAAAGGGGATAAAARGGEAAAEVTGWP.[A]	2540.21242
4542	[R].YPEPAHLSANARRLIARLLAPNP.[A]	2540.42083
4543	[S].KMHAQQQQQQPQQQQQQHGV.[F]	2541.21237
4544	[Q].VSGGGPGVSMVMKTLEDAENEAILH.[P]	2541.23235
4545	[A].AAPAGQAELSQERQNLFTGYFRS.[L]	2541.24807
4546	[T].NTVPNSSPTAASVSTDVYGQAIYEA.[C]	2542.19437
4547	[M].PGAVPGSGHPGVADPGTPLPPDPTAPSP.[G]	2542.25724
4548	[G].PGLAPPTQPGAPSMAGTVAPGGVSGPSPA.[Q]	2542.26061
4549	[G].MQISGAAAPAPAPSGLGYGPPTSLASAS.[G]	2543.24463
4550	[Q].QQQPATGPQPSLGVSFGAPFGSGIGTG.[L]	2543.25249
4551	[P].PAGSPLQEESPSLSPRGEAQGQQPP.[Q]	2544.23248
4552	[H].VSPQTGSPHPGLAVTMASSIDQGHLG.[N]	2544.25111
4553	[T].PGSSTPGPGTPVPTAGSVPSPSGSVPGAAA.[P]	2544.25764

4554	[R].GPLGLPGASGLDGRPGPPGTPGPIGVPGP.[A]	2544.3569
4555	[I].GPQKSAPEHKAKKSPPHRSHSRP.[G]	2544.36544
4556	[D].SDPVGTGDWRPPRPSTAAEPPTGTP.[V]	2546.227
4557	[R].QRGAGGAPGGEADARPLAWSQPWGGP.[L]	2546.22834
4558	[A].REEGVPGGGARAGEKEHPEGTQQGL.[R]	2546.23422
4559	[P].YSQQRPSGPGPVTQGPQQPQPPSQ.[Q]	2546.23824
4560	[M].PTPATAQQPPDRPQGAAPASDTAITS.[R]	2546.24813
4561	[F].PPAAFMPTQTVMPLPAAMFQGPLT.[P]	2546.24919
4562	[A].GPASTPVGRVSSREPAPGAPGRESGLG.[R]	2546.30699
4563	[R].PGPSPGPGPSPGAMLGPSPGPSPGSAHSI.[M]	2547.22965
4564	[A].GPAGGKASTHVTSSSLTIWPSPAGSPP.[P]	2547.28379
4565	[E].AVTGGTASGPAPDPIRAPDPPAPDSAY.[C]	2548.23142
4566	[S].QREAASGAHAGLGHIPWGSAFGHHP.[H]	2548.2341
4567	[Q].LQQMRMQQPPAPAPTTTAATQQH.[S]	2548.2395
4568	[G].GKAGGQGAAGVDQWLEVFSEREEK.[W]	2548.24265
4569	[N].TVNMPPLSTISPSGTQSKSMPIKD.[N]	2548.26332
4570	[P].GPQGQPGLPGTPGHAVEGPKGDRGPQG.[Q]	2548.26512
4571	[Y].PAAGDAARAREAAAGAMAAPSPGPREI.[L]	2548.26849
4572	[A].VPTPEQSATLAGAVSTPEEPATPAGAV.[S]	2548.2777
4573	[R].VEANVPMVPAPAEEPGARNGVERC.[L]	2549.22352
4574	[S].PDQPPSPAQSPAPRPDAQAEEVAPP.[P]	2549.22667
4575	[V].GARSAAPGGGSVAAASAAMGAALASMAGLM.[T]	2549.22689
4576	[S].GLSPAGPELGAFSQSPAPAMGGRAGLH.[C]	2549.25653
4577	[P].KGPASGPGSGGKMALNSPQPGPVESEL.[G]	2549.26643
4578	[V].EGFVLPSSTTREMTPGEIKFSVH.[V]	2549.27045
4579	[T].PGQPGFPGPKGEMGVMGTPGQPGSPGP.[A]	2550.17517
4580	[L].PGAPPQQVQYGQPAPAVAPPMAPSHG.[T]	2550.2558
4581	[N].TTQIPMNVAKETQLKGGQHSQAAP.[V]	2551.29331
4582	[P].LGEEPEVPGAASAELGTSEGSVQQPL.[L]	2552.23623
4583	[H].TQLSNPSSLANIDFYAQVSDITPA.[G]	2552.25149
4584	[L].QPSPGTTLGPPAASTPAGPPSGGASPTPP.[A]	2552.26272
4585	[K].PGPSPLSATQGATPQQPPVNSLPSSH.[G]	2552.27395
4586	[A].GPVTMTSVHPPIRSPSASSVGSRGSS.[G]	2554.26782
4587	[K].QMKFAASGSFLHHMAGVSSSKLSM.[S]	2555.22035

4588	[S].PGAEKADDPRGPEGNLKTTTLECV.[N]	2555.24061
4589	[S].PGAGPTEPPLPEAFAPSACIVEYGK.[A]	2555.24865
4590	[R].MQLWLSDQLGQAVGQQPSASQVSP.[T]	2555.25586
4591	[A].QQPMVPQQPMVPVPGQHSMTPIQ.[HP]	2555.25673
4592	[G].RYGGEAVTGLRGEVGDVGKWKANH.[Y]	2556.30659
4593	[K].VGEPGVAGPTGPPGVPGSPGLTGPPGPPGP.[P]	2556.30928
4594	[W].PGSPQVSGPSPATRMPGMSPANPSLH.[S]	2557.2286
4595	[G].EAAAAAGAGAGETAVKVEGPGSPGVPGSPP.[E]	2559.26853
4596	[G].GPGPSPRSFHAAAYVPAGRGAMYLL.[G]	2559.29252
4597	[D].INDNAPRFGVEEVELKISETTTP.[G]	2559.29369
4598	[R].HTLAMTNPTAEIPPDLQRQLGQQ.[P]	2559.2984
4599	[A].QGPTVQAPMPTPRTVDDASQRPTP.[T]	2563.25692
4600	[G].INAHAATTQYANGVVPSGQTANAVAH.[R]	2563.26479
4601	[P].NPPQSPATPFAPAASPSAPQSPGYQV.[S]	2564.24159
4602	[N].NPLELGEQPEQPPLEAPGAAAPGAGPG.[P]	2564.26272
4603	[A].AAAAGGGGATAAAARGGEAAAEVTGWPAGAP.[G]	2565.24405
4604	[A].EVESRPGNGQCCDCGAADPTWLS.[T]	2566.03915
4605	[Q].QGPGLAHPSSRHSPTCIKSGEGPPL.[L]	2567.27833
4606	[C].SFVQNLNSVNVSGASSQPTFPSSLT.[N]	2568.25764
4607	[S].QPPPGPEPPQPPQKDSQQPAQQPP.[P]	2570.26339
4608	[T].PSSAPSPLGGSALCGGKPEAGESPPPAP.[G]	2572.23479
4609	[T].ATPGAAGGATAASAAASVLGGSAAPATAGDTT.[K]	2572.24853
4610	[P].PLVENEEAEPGRGGLGVGEPGPLGGGA.[G]	2572.26378
4611	[K].REYPPPPPEPAAAPTSPATAGSPATAA.[G]	2572.26781
4612	[G].AQVGPGQGPVPQDGGAAPSEPRGTAALS.[E]	2572.27502
4613	[E].PAGSAASEESAPYCSGGAAAFSDREK.[L]	2573.12088
4614	[S].QPGFSSVPAFGQSVSSTPTSTSGNVF.[G]	2573.21544
4615	[W].PGSPQVSGPSPATRMPGMSPANPSLH.[S]	2573.22352
4616	[G].APQKEEDLAGYYLTTWFGALYH.[I]	2573.23472
4617	[P].PGKMGPQGTPGIPGMPGPIGQKGDPGE.[N]	2573.24867
4618	[L].PANASLAAMAAAAGLNPGLMAPSQFAAG.[K]	2573.24867
4619	[V].NNGVPGGAAAAAAATVAAASATTAASSSLAT.[P]	2573.28016
4620	[G].AGQAGVVQPGAGQAGVVQPGAGQPVVMQ.[P]	2574.30929
4621	[V].QVSASPAPGTSAVSPRIEAVSVTPEH.[A]	2574.31582

4622	[E].GVGAAANAAATSSTGTGGVAASGMAASGVVPG.[G]	2575.24167
4623	[S].PGTSAFVGTPSPMRFTFPQAVGEPP.[L]	2575.26497
4624	[D].RPSPSSNQPWGLAALLLPSVANNVS.[L]	2575.36271
4625	[L].ISQNFTTAEAAPLEAPDVWRNVM.[F]	2576.24496
4626	[G].WGSLQELSSPRQPVSPPENSRAGP.[R]	2576.28519
4627	[G].KGTTSSSASFLPDISCWDMDQED.[D]	2577.07557
4628	[R].ACGFAQPGCSCPGEAGAGPATTTTPPG.[P]	2577.08028
4629	[S].LSSYNPFEDEDDTGSTVSEKEDI.[K]	2577.09986
4630	[L].IGGPMTGDTVAATGATTTAEIGEKPTM.[T]	2578.23749
4631	[P].GMPGTKGGPGDKGEPGRQGFPGVSGPPG.[K]	2578.24669
4632	[L].PVSPSGAQASPSSSSRPGVSGASQPPQA.[S]	2578.2492
4633	[G].AVMVPSAMLMGQVVTAYPTFAPQH.[P]	2578.25025
4634	[P].GPAAHSQGLPTASPSLSYSTGHSPALS.[G]	2578.25322
4635	[L].AAQAQVQAQAQAQAQAQAQAQAQAQA.[Q]	2578.27166
4636	[Q].PADKASASGSGAPVGGSISSGSSASSVTVT.[R]	2579.24311
4637	[H].PGPFPSSAGPGPHYLSSALPPGTYAGP.[T]	2579.25651
4638	[R].LSGLPGDPSLPRGLAPPLPPTTNQSP.[R]	2579.38278
4639	[D].PWGPPSSLMSEIADLTFNTVAFAE.[V]	2580.23267
4640	[F].ARGGRESYSIAGSEGSISASAASGLAAP.[S]	2580.26485
4641	[M].FGQPGALGSTPFLGQHGFNFFPSGI.[D]	2580.26702
4642	[G].QPSSQLQPITYGPSHSGTATTASPAP.[S]	2581.25288
4643	[C].NVELPTNHTNAVFAVMHAQRTSGS.[G]	2581.25759
4644	[Q].QQPGQPPPHSTWNRHSLPLYSGP.[K]	2581.26948
4645	[Q].GPPGKMGPQGTPGIPGMPGPIGQKGDPG.[E]	2582.28539
4646	[M].PPPPMPPGAGGHGPPSAGTPGAGHPGHGH.[S]	2583.20583
4647	[R].AARDGTEGARHPEARPSAPEQGLPGG.[R]	2584.2611
4648	[D].PGVGGTGLEQGPSAGAASAGPQVSLYQGA.[P]	2584.26378
4649	[C].AQPAAEAAAPGWAQARGHPGGELAAAAS.[A]	2584.26512
4650	[P].GPQKTMERGGPMGCADCSADSHGY.[S]	2585.0272
4651	[-].MGPHPNAIKSCAQPASAAAAAAFADKY.[M]	2585.25653
4652	[M].GPHPNAIKSCAQPASAAAAAAFADKY.[M]	2585.25653
4653	[V].AVGQEQAFSVNTRGAGGQGHLDVRM.[T]	2585.26374
4654	[A].ERVSVGPCPPAAPQPPGSAVSGPSVGQ.[G]	2585.27766
4655	[G].SLICNAGAGGPAPAAGAAPAGGPAPATTAAP.[A]	2585.27766

4656	[-].MAGPAMLEAAALALCLLLASPGLAWY.[K]	2585.35061
4657	[G].TSAPLEDTPPLPDAQYVMVQERE.[H]	2586.23921
4658	[A].MTKVMTGTGVPQSIQAQGPSSPSSPP.[V]	2586.25381
4659	[L].GSTTSAPAPSAPPAPPAFHGMLERAPA.[E]	2586.27693
4660	[S].QPQFPLAPGVQQCAPVGLYGSPFGA.[R]	2586.28095
4661	[A].AAAGQPGTAPSGVPGAPPLPGMAIVKEE.[E]	2586.32321
4662	[S].LNGLMYGAAQPGGCMDNRTFPYPG.[V]	2587.15266
4663	[S].GQRIDTGSSKDYEPCSQSLEMAK.[A]	2587.17629
4664	[Q].VQYGQPAPAVAPPMAPSHGTSVTPNP.[A]	2587.26095
4665	[I].PSAVVPGSMAGRMTTTVAPGSIAGGMAP.[S]	2587.26769
4666	[T].TAGPAPGGPAQPPPPQASASDLQFSQL.[L]	2587.27871
4667	[L].QAGVMASPPPPGLPTGSGPLAGPHHAW.[D]	2587.28744
4668	[S].GDIQGRNTSPSVSVQKSNPMRITD.[S]	2587.28929
4669	[L].PNQNGTVLPSESTGLATASCPITVSS.[V]	2588.25084
4670	[S].PQEAGTVDVWRIPEAGAAHSGLTPE.[S]	2588.27395
4671	[D].TTGGRQSGAVLYNTFGIMGKANVTE.[R]	2588.27733
4672	[-].MSAGGAVEPGLPAAAAAPSPAPARDPGPGH.[L]	2588.28519
4673	[D].RQETPAGPGPALSDILRSVQRARI.[P]	2588.43794
4674	[I].QGPVSQGPLMGLNPRGMQGPPGPRE.[N]	2589.26605
4675	[R].GGPSPPGHSPGPPRTFPSAPPRASGSH.[G]	2589.27054
4676	[A].AAAAASVGGTIPGPGPGGGQGPGEGGERRT.[A]	2589.27641
4677	[S].PAGGSPGKPGSTPHVSGLGSPGRYSPAN.[G]	2589.28044
4678	[A].SRGSIHRTSTSPSLTGPSPDAAAAPAP.[T]	2589.30157
4679	[P].HGINDILSRPSMPVASGAALPSASPSG.[S]	2589.30896
4680	[E].PGTTQTPEQAKPVATPGTTSPGPTPGD.[S]	2590.26312
4681	[G].PQGNNLPSPGAGSWPPPAFPALPSSF.[L]	2590.2725
4682	[A].TAPTAGGEPLSPPPPQEPAPGAPQQTP.[W]	2590.27837
4683	[A].PPNPSPSNPTPASFAPVPAPEASPPAP.[D]	2591.27764
4684	[P].SGPPGKPGTGSPGPQGQPGLPGPPGPSATG.[K]	2591.28485
4685	[A].VAGNGGAGAAVGEPPGPEPLTLDSYQAP.[G]	2592.25764
4686	[A].ANPVSQSSPAPPTPIPGLQILNIALP.[T]	2592.43956
4687	[A].LGPGSSTQPSATALSQLPLLKAVVKE.[V]	2592.46069
4688	[E].SAQSKMLSGVGGFVLGLIFLGLGLIV.[R]	2592.48334
4689	[P].GNAGPVGTAGAPGPQGPVGPTGKHGNRGE.[P]	2594.28183

4690	[G].PQRPDQPATAAAAGPGDPKRKGGPGPT.[L]	2595.33862
4691	[A].EAAAAAGGGGATAAAARGGEAAAEVTGWPAG.[A]	2597.23388
4692	[P].GVGAAGGSLSFGASSTPAQGFVGVGPFGSA.[A]	2597.26306
4693	[P].PWGPGSRGFPGEKGELGEIGLDGLDG.[E]	2597.26306
4694	[A].PGDGPRERTATTVTDSRGAGGGGSGALP.[A]	2597.26624
4695	[E].TGSEKHPAPDVVGTASLQDSSNTLSV.[A]	2597.26893
4696	[P].SANLDFPSPIPSVSSSFMISSSFPP.[M]	2598.24323
4697	[G].DRRPCPMVAPGPEGLSTPRSQGPH.[V]	2599.26163
4698	[P].KGDPGFQGMPGIGGSPGITGAKGDMGLP.[G]	2599.26432
4699	[R].AGRPTRECGVAAASKMQASSSRSVH.[L]	2601.27326
4700	[M].PSPWAPSADCAMESPPPAPLPCPGP.[A]	2602.14109
4701	[G].VAEPPEASAPEVPMEPLEPRSPEQ.[S]	2602.23412
4702	[P].RSGAEAANVTGPDGVPVEGSRYAADR.[R]	2602.26043
4703	[R].GQPWAPPSLHLCHFGLPEDHPPL.[R]	2602.26597
4704	[Q].QESGLLGDPGARVYSSHSMGARVDL.[E]	2602.26782
4705	[A].GPSPQARATKCPAEEPVTAWAPSPP.[P]	2602.27185
4706	[E].KEPEGPVEATVASGCLTRHAAREH.[R]	2602.27906
4707	[E].PSGRDAQGRASTPAPPAWEREKTH.[H]	2602.28692
4708	[T].TSQPATGTTATPKRKKRVMATRQS.[P]	2602.42058
4709	[A].STGQTFQITGNPVTMAGKVITKLPL.[P]	2602.42728
4710	[E].EGRAGSPAVGEEAPASKEPEQPGPQAG.[T]	2603.23321
4711	[E].EPATPAGAVSTPEEPATPAVSTPEEPA.[T]	2603.2359
4712	[A].VMKDPHMSKTAPPSGARSHPGSSQP.[S]	2603.24531
4713	[A].PGGGPSPSGRESHRLQVTQYSYRS.[H]	2603.27093
4714	[R].GEVFIGSRESGYTVLDGLPPSTQGH.[H]	2603.27362
4715	[G].GAVGAGLMGLAGGVVGAGMAAAALAAEAGMV.[A]	2603.29899
4716	[S].SAPSNIAPSDVVSNMTLINFTPSQL.[P]	2603.30214
4717	[T].TMGQLQAGVHVVAEPGLDVPEGTALN.[L]	2603.31338
4718	[S].LTSPSTPSSLGPSLTSTSGIGTSPSQR.[S]	2603.31588
4719	[P].PDSGSLTPPGHRPPHPAPRPPLLLP.[R]	2603.4205
4720	[S].ESPARGPSGSPRTQGRGGPASVPSASPG.[T]	2604.28731
4721	[G].WPGTPGAPGPKGDPGFQGMPGIGGSPGI.[T]	2605.25038
4722	[M].VMMQEEPVIVKPMPGRPNTQNPP.[R]	2605.29351
4723	[-].MVMMQEEPVIVKPMPGRPNTQNPP.[R]	2605.29351

4724	[G].QPDAGARAEQDLGGRPVEQVQALEA.[R]	2605.29648
4725	[L].KMPQQARDAESIMLNLAGQLIMM.[Q]	2605.29688
4726	[L].AGSAALGTFHGTLACVLGSTLELLDM.[G]	2605.30004
4727	[E].VALGQCNELLGANPEAEGLLQGKHS.[T]	2605.30387
4728	[G].PGEGAPAPGEKQEGSRAPPEQPPADH.[G]	2606.22298
4729	[P].SGPPGPKGDDGIPGQPGLSGPPGPKGEPG.[H]	2606.28452
4730	[G].DVFKGGGRSGGGLTGPPLGGGGPTPPAGAD.[S]	2606.29575
4731	[P].SPDKQRMPMPVNTPLGSASRKMM.[Y]	2607.25099
4732	[V].PGYQQTPPPGVSRAPASSGAPPTSTAQ.[G]	2607.27977
4733	[S].GPQTPTSTPAPGSATRTPLSQDPACI.[T]	2608.26715
4734	[P].PASSATASSTATATLQLQQQPDLSSF.[L]	2608.27368
4735	[S].FPSNPSTPVGSPSPLTGTSQWPRPGG.[Q]	2608.27904
4736	[I].VDSTAALAGFSEAVSSAPRAPGPYGPH.[R]	2612.27395
4737	[S].QASTVSPTSATSASVHLDSSLTLSQH.[Q]	2612.27983
4738	[P].GPTGPKGTSGHPGEKGERGLQGEPGPQ.[G]	2612.28117
4739	[D].GSVIRELDGSLSGAVNGMDITVEGVH.[F]	2612.29845
4740	[H].VAENNPPGASIAQVSASDPDLGPNGHV.[S]	2613.25395
4741	[S].ASQAGVQQPPATSTGGPAASAVSASVSTQ.[V]	2613.27508
4742	[A].PGPEQPPTPTGAGATSGQGALPASQGVSP.[R]	2614.27435
4743	[E].AALSPESPNEAESFPTQQETQGLSP.[V]	2615.21075
4744	[T].PGAAGGATAASAAASVLGGSAAPATAGDTTKS.[E]	2615.29073
4745	[E].PEELPTPMAQALPSPASTATPPPTPT.[H]	2615.29091
4746	[L].DPKATATNWSEADMEPCVDGWVH.[N]	2616.11296
4747	[D].PAATKTPGMEPSGSVAGLGEVDPTAFL.[D]	2616.28616
4748	[Q].PPQPPHSSSPGPSPVLSPSQEQVLAP.[A]	2617.32566
4749	[P].AAAGEIPGTATRGPRSPPAPAGYPQAR.[N]	2617.35936
4750	[G].PQGPPGPQGNAGPQGHLGPQGPPGPQGH.[I]	2618.2607
4751	[P].QAGGGRGAGDLVQTPRGLSDLEIGMY.[A]	2618.29912
4752	[T].GPEGKPGAATGPATMVLGREDGLQRH.[S]	2618.31036
4753	[S].PGTKDASAAPATSFTSLSAKNVIKKK.[G]	2618.45119
4754	[F].PATGSVGTGVQMLAVSHKGIKLLRVV.[K]	2618.51744
4755	[N].QNVTPFGMLGGLVPVTMPFQFPLE.[L]	2619.33496
4756	[G].AAPAGGPAPATTAAPAEEKKVEAKKEE.[S]	2619.36244
4757	[R].EIINMQPPQKPEAPREEMLAPGE.[K]	2620.27455

4758	[L].PQDNELAIDLRQTAVVVMAHLDR.[L]	2620.35116
4759	[P].SPGPSALSPGSSGRTEKNSLSFKSDQ.[V]	2621.28016
4760	[L].PPQPHQQVPGQWGPGQGGPRPPGQH.[Y]	2621.28686
4761	[S].APQAEVGMLPSQRVASVQSEPGQQN.[L]	2624.2733
4762	[G].PMLGGGSSPLPLPAGGSSSVGGSGGFGSLH.[Q]	2624.27733
4763	[A].RSPGTSAFVGTPSPMRFTFPQAVGE.[P]	2624.29258
4764	[Q].GPSAGAASAGPQVSLYQGAPPAAEQGVVS.[R]	2624.29508
4765	[S].PAVMQPPPGMSLPPADIGPPPYEPPG.[H]	2625.27276
4766	[W].PQGPAGPPGPPGPMGPPGLPGPMGIPGSPG.[H]	2625.29522
4767	[T].PGADRQAARATGPQPIRVAGSLPPGW.[T]	2626.39608
4768	[A].GPWLGPWTPHWLSLAATALLLTLL.[P]	2627.47483
4769	[V].PGARAEEPGASSSHHGSMGLDKNTVH.[D]	2629.21718
4770	[A].GHHGDQGAPGAVGPAGPRGPAGPSGPAGKD.[G]	2629.26143
4771	[T].VQGDGVIVSTPTGSTAYAAAAGASMIHP.[N]	2629.29264
4772	[S].PGAASTPRGGQSQQQQRGGGPQAQSHG.[E]	2630.25266
4773	[D].KGMPPLSMEMFISLNVADINDNPP.[A]	2630.26629
4774	[L].TSPSAYAGLHNIPPQMSAAAAAAAAAYG.[R]	2630.26676
4775	[S].GTTNTATTAGPAPGGPAQPPPPQASASDL.[Q]	2630.26926
4776	[Q].GPWRPGPGGSTMDSKLTLTDCVAQV.[E]	2630.27013
4777	[G].AGGPQQAIGEMAVPPSVPSSQKDYLS.[N]	2630.27666
4778	[T].VCVTGPPTARPSEGPTTGPTGPPAAGPT.[G]	2630.28789
4779	[Q].LSGGQGAAEPPQPQPQPQPQPQPQPA.[A]	2630.29575
4780	[V].NIGSLICNAGAGGPAPAAGAAPAGGPAPATT.[A]	2630.29912
4781	[A].AAAGAGAGETAVKVEGPGSPGVPGSPPEAAA.[E]	2630.30565
4782	[G].PSGPPGKPGTGSPGPQGQPGLPGPPGPSAT.[G]	2631.31615
4783	[R].PGQAPIGNPPVGPIGGMMPPQPGIPQQ.[Q]	2632.33742
4784	[A].GANRPTNPAAPSVMGAGPAGPSSQAPGTV.[I]	2633.27364
4785	[G].GPGSQGIQGPVSQGPLMGLNPRGMQGP.[P]	2633.29226
4786	[P].PGSGLGTFPAVAQSPYADARDKNPAF.[N]	2634.29469
4787	[T].PQPTGLSGVFDTSVKSASTNTKEPSV.[M]	2634.32572
4788	[G].GSPEALRGGGRGGGVGSSGLCRALRSY.[A]	2634.32774
4789	[L].ARDLYYLLPSTDVLGPAPYDPGAGL.[V]	2634.34499
4790	[Q].AQVQAQAQAQAQAQAQAQAQAQAQAQ.[A]	2635.29313
4791	[M].ELTEQPVTTTELEQPVGMTAVEHP.[G]	2636.27599

4792	[V].RAAGGGPAASSTQAAFNGTSRSNTTQLG.[A]	2636.27714
4793	[G].QKGDPGTPGYPGKNGPMGTPGIPGTPGT.[M]	2636.27733
4794	[G].EPDALTPPSAASASASAKAAEAGPPTDR.[G]	2636.27983
4795	[E].AAAAAGGGGATAAAARGGEAAAEVTGWPAGAP.[G]	2636.28117
4796	[P].SGTPPQPCVLSAPQPGPPTSSVTTATT.[D]	2636.28722
4797	[L].PSGNWIAGPAHTGREVGFPNCSLLV.[Q]	2636.30382
4798	[I].AAMAVPTSIYQTSTGQYSATIVQYA.[A]	2638.27051
4799	[E].PGAGAGDLQGLASDLVASGSQAGGGRGAPGS.[P]	2638.28156
4800	[P].LPDEVTQEGQALASARTGGKAEEPPS.[Q]	2638.29548
4801	[V].QPGAGQPGVVQPGAGQAGVVQPGAGQAGVV.[Q]	2638.36959
4802	[N].DTKTPKVDDGSSSEIKLAIPVFFF.[G]	2641.37596
4803	[L].KDLGEIGRGAYGSVNKMVHKPSGQI.[M]	2641.38788
4804	[D].LKDLGEIGRGAYGSVNKMVHKPSGQ.[I]	2641.38788
4805	[E].ASPSVPSSTAALMGGPTTVQTVAGETVQ.[T]	2644.31344
4806	[V].EERAPTQSPELTPSGPAPAGPASAPET.[N]	2645.26893
4807	[P].GPLSGSQGPGQQCLGQAGLPGSVPASTH.[S]	2645.27364
4808	[T].SPGAAYAGLHNISPQMSAAAAAAAAAAAY.[G]	2645.27766
4809	[D].PGPGPEGRAPHSAIEEKVMKGIEEN.[M]	2645.29879
4810	[V].GPAPGMRPPMGGHMPMMPGPPMMRP.[P]	2647.1715
4811	[S].QAAIDTQAGASPNSPGVDFGEMRGASP.[T]	2647.20528
4812	[D].QPSFVSPPESLVGQHIENVSSSHGK.[G]	2647.31107
4813	[I].KGLGQGAGESPGSGERKAPPAGREEAGP.[E]	2647.31828
4814	[Q].PGSPGLSGQPGLPGPPGLHGFPGAPGREG.[P]	2647.33756
4815	[G].PEGVAAQAAPSVASAGPADSEMEEVFD.[N]	2648.16683
4816	[S].ASAASGLAAPSGPSSGLSSGPCSPGPPRPA.[S]	2648.2733
4817	[G].GSLSQTPGTPPGPTMASREVNKICF.[P]	2648.2807
4818	[A].AAAGGGGATAAAARGGEAAAEVTGWPAGAPGP.[C]	2648.28117
4819	[G].EGPETLSSALSKGAAVYSPSRYSYQ.[L]	2648.28385
4820	[G].GPSGGRATSWRHGVTSPQNSALCKH.[G]	2648.28587
4821	[P].PSSVLASGHHTTSAQALHHPPHQGPP.[L]	2648.30765
4822	[P].PGPQGQQNPARGPHPSQGPIPFQQQ.[K]	2648.30765
4823	[K].KGHRNNATNSKPEFSIASLNGLMY.[G]	2649.32019
4824	[V].PGAPGMPPGIPPLMPGVPPLMPGMPPV.[M]	2649.33114
4825	[V].GPAGAKGMPGHNGEAGPRGVPGIPGTRGP.[I]	2649.34266

4826	[T].SLPMTSAVQNSTYTTSVITSSSLTSS.[S]	2650.27638
4827	[H].VAITSTVMLEFPLTSHWWAALGFG.[L]	2650.33741
4828	[P].GPAQEAVPTRAGARGAQQSRGVQTEQ.[P]	2650.34041
4829	[P].TPFSAPPPAGAMIPPPPSLPGPPRPGM.[M]	2650.35201
4830	[L].KLTCNEHLPTASNPAVSAPQMQKE.[P]	2651.2916
4831	[T].AGVQEPGAPGGGAQAGPGGDGWGTDTKEQ.[R]	2652.19208
4832	[H].EVPGHPPGGDVGQMNVMMQRLGQDS.[L]	2652.19632
4833	[Q].WKGTSGFGRSQTMLGEDSAAGDSKY.[Q]	2652.19947
4834	[Q].PGDAQLAGGPTSASPYEFSPPGGPVPLS.[V]	2653.27804
4835	[D].SFLAELPGSLSLSSAEPQPTSPQPAAA.[A]	2653.33555
4836	[K].KSPEGSISRTASSLASSPGMVPDHVR.[K]	2653.33624
4837	[G].GSAPIDPPPVHESPHPPLPATEPASR.[L]	2653.33689
4838	[R].PSHPGGPPVSGALPGPGLGTNASLAQMVS.[G]	2653.34026
4839	[R].ASYVAPLTAQPATYRAQPSVSLGAAY.[R]	2653.36204
4840	[G].RGAGGVGGGRWLTSRSLADPGSARQPS.[L]	2653.36657
4841	[A].TVGVSFSFFWKTQGEQSTSIPSAY.[G]	2654.27731
4842	[K].PQGAGPGQAAPGGASRMAEGRAGGAAGLFA.[K]	2654.28521
4843	[E].VDALNSSHPVSTPVENPAQIREMF.[D]	2654.28789
4844	[E].QSEFSLTLMALDGGSPPRSSTSMVR.[I]	2654.29126
4845	[F].PSSAGPGPHYLSSALPPGTYAGPTQML.[Q]	2654.29191
4846	[P].MINTPPPPPPGGFGSPATPPPPSPPSF.[P]	2654.29594
4847	[M].QQSPLYSPQNNMPGIQGATSSPQPQ.[A]	2655.24675
4848	[G].PKGERGFPGDAGLPGPPGFRGPPGLPGA.[P]	2655.37903
4849	[A].SGNRASPAAASAVPGSGAAAGALASGGSKEE.[F]	2656.29212
4850	[A].VAMAATLTQQQQPATGPQPSLGVSFGA.[P]	2656.33993
4851	[S].PGNTTQAPGPEQPPTPTGAGATSGQGALP.[A]	2657.28016
4852	[E].PALHPGPGPGQVSGPGQAPVPAHVPAPAP.[G]	2657.39468
4853	[I].PGADAATLQGSRASRPGGSHGDSGSPPPA.[L]	2658.26149
4854	[R].RGSFVNSSGVMNQGVAPMVGTPAPGGSP.[Y]	2658.27628
4855	[E].QGVFSQSFLGTFWTVGSLSAEGQPQ.[T]	2658.28346
4856	[L].VEEGSERARPSPAPSSPASAQVSPQH.[Q]	2658.28664
4857	[Y].QAPGPSPQFQSPPAKQTSAFSKQMP.[H]	2658.29806
4858	[T].QLGASGGQGAPTPTPAPASTSQEPPLPSG.[P]	2658.30056
4859	[P].ASPSSASKEVGIGFAQGPGASASTAATPGP.[A]	2658.30056

4860	[L].FPGAAAAAAASLEPPAEAEPAPGPSSPQV.[P]	2658.30459
4861	[Q].QGPPPAEEKGPTAPAHGLRGAWPEAH.[S]	2658.31716
4862	[L].SASLQPTPQAVHPPRQLQPSLTASF.[T]	2658.39982
4863	[P].QTSPMLGSSIQTFAPSSQEVGSGIHP.[D]	2659.26682
4864	[L].SRAMAPATTSVTSPAVITEMVPEPAE.[C]	2659.29534
4865	[L].PGSPGEKGEKGETGQPGPPGLDGPTGEK.[G]	2660.27983
4866	[G].PGSPATLSPSAGVPQPVGMEALDQAEGP.[A]	2660.28722
4867	[P].GPKGDDGIPGQPGLSGPPGPKGEPGHPGT.[D]	2660.30632
4868	[Y].GPPPTSAQVTAQLAGMQISGAAAPAPAPS.[G]	2660.33484
4869	[P].AMGPVPAMVVPPQPQPMLPSVDARQ.[L]	2661.31973
4870	[C].GQPADKASASGSGAPVGGSISSGSSASSVTV.[T]	2663.27547
4871	[A].AAAASAASAVGPVHNSVPSNPVAAPGFFV.[H]	2663.35762
4872	[G].MAASGVVPGGGFVASAAAEVQTGRNNFV.[I]	2664.31986
4873	[H].TWTWLSPTGQATTPATTGPPSQPANP.[Q]	2665.28927
4874	[K].KAQEEAPQQPEAAAAATTPVTPAGHGH.[L]	2665.29648
4875	[P].QGPAGPPGPPGPMGPPGLPGPMGIPGSPGH.[M]	2665.30137
4876	[E].AVCVGPAPTGKSYLNMDAIMDAVRN.[S]	2666.2735
4877	[E].PTGSASVSTGSRAGGAAGVGGEAGPPPERE.[G]	2666.27647
4878	[S].GVSAAGGGPAGAAGGAAGGGPAAGPADHGLAGRGA.[A]	2668.29346
4879	[D].GQPGHKGERGYPGNAGPVGTAGAPGPQGP.[V]	2668.29748
4880	[G].PSSLPGFPQNSNTLTPPGAGMLGFPPS.[A]	2668.30756
4881	[G].PGGGRAAAEAQGQPLPLPEAHAAYNQP.[F]	2668.32264
4882	[H].GGPSAPGALQPLTSGSAGPAQPGSVAGAGPGP.[T]	2668.33253
4883	[S].PVSSVHSHPGQSVRSVNSPSVPALEN.[S]	2668.34377
4884	[S].KVWEPGTPVTSPESLPMSILHPLH.[P]	2668.38033
4885	[G].GPTSHPSPAPTPSSAPSPLGGSALCGGKP.[E]	2669.29879
4886	[W].VNEVRYGGFSLGGRDPGLPSGLEVGH.[S]	2669.34304
4887	[G].NGLVGPGGSGAGPGGGLTPTAPPYGAGKHAP.[P]	2669.34304
4888	[H].KQSYYSIAKCVAALTRACPKEGPA.[V]	2669.3538
4889	[R].TQPGPPEGVTASSVGAPRSTPPARSPAP.[S]	2669.36417
4890	[L].AQRTAEPSSPRSPSLAQPATPPAAPPS.[A]	2669.36417
4891	[Q].QPGSAAAAAAAAAAAAAAAAALGHPQHHAPV.[C]	2669.3655
4892	[T].GQLLGGPLTQMPPQTASSHPAAPATVAA.[S]	2669.37156
4893	[E].QPQQETPAAEGVGAAANAAATSSTGTGGVA.[A]	2670.26015

4894	[G].GPAATSPSRPSPPPPRSASAGETPSPTI.[Q]	2670.34818
4895	[A].NSPLGNPFQGNVFPAPAVSTQTPSML.[S]	2671.31846
4896	[E].SPPTPQTSLTPPQASPAASKDQSPPPS.[P]	2671.32096
4897	[T].PGAGGAGRARGSSFAKFGNRNVFMKD.[H]	2671.32701
4898	[G].SSTITVPGPPGPPGAMGPPGPPGAPGPVGPA.[G]	2671.35485
4899	[P].PPGTAGPPAVHGLAMAPASVAPAPAGSGAPP.[G]	2671.36608
4900	[V].VSRSGSLTPHVNFPLDSHPVSPEVI.[V]	2671.38384
4901	[P].ATTTGAAPPQPPGTLSKPMSVHLLNQG.[S]	2671.38721
4902	[R].PGPWPPGAPASEALVAEFLQDQNAPL.[I]	2672.33549
4903	[A].AAPAWPGLAEGRRRAGAMAEAGPQAPP.[P]	2672.34741
4904	[S].GQTHYLSLSGFELYGTVNGVCEDQ.[L]	2674.20897
4905	[L].GPQGPPGPQGNAGPQGHLGPQGPPGPQGH.[I]	2675.28217
4906	[A].PGGPGPHPASAPAATQEAPSALPSPQMPG.[D]	2675.28822
4907	[P].VEHGPASQPPSPGPAVSPGPADLDPEAV.[R]	2675.29475
4908	[A].IDARPACAGAAGRQSAGPQPSPEGVTGP.[R]	2675.29544
4909	[E].AQEAGLAETNISGPGALTRCQLPSPH.[P]	2675.32059
4910	[G].PGPGPGPGPGPGHSMRLPVPQGHGQPPP.[S]	2675.32595
4911	[R].GPAGLYTHPGPVGSPGMMMSMQGMMGP.[Q]	2676.15696
4912	[P].GPGVSFSPGPTPTPAPTAGSFAGGAGGPSPP.[L]	2677.28927
4913	[G].GAGLCCLGTPDPALPRGAGSSRQGPQP.[P]	2677.29333
4914	[P].GPSPGAMLGPSPGPSPGSAHSIMGPSPGPP.[S]	2678.27013
4915	[G].SAPVSMLTTVTMLDPGSSAPGGTTPISS.[K]	2678.28992
4916	[E].QQAHPNPPQSPATPFAPAASPSAPQSP.[G]	2678.29575
4917	[A].RGAPTAEGPEPRGPPPQYPHVMLAH.[E]	2678.32561
4918	[F].NAVMQNLAQSHILTELMNEIKEH.[G]	2679.3229
4919	[P].GSPGQAGAVGIPGERGPPGPPGPPGPPGPPA.[P]	2679.36377
4920	[S].AQPAAATPASVSSPAGSPGPPGSTASLSTAS.[L]	2680.30604
4921	[V].NILSGMGNGTIASSAALNSAASAAAGMTVG.[S]	2681.2869
4922	[P].QGPAGPPGPPGPMGPPGLPGPMGIPGSPGH.[M]	2681.29629
4923	[L].SPASPPSSPRTKDPPTGSPPASPGPQSP.[S]	2681.31655
4924	[D].PGVGGTGLEQGPSAGAASAGPQVSLYQGAP.[P]	2681.31655
4925	[Q].APTTSSQQAALESTQVVSQVAPSEPAP.[G]	2681.32644
4926	[T].GSQGSSKSCRNLKRGSPGAGAAGISPGH.[S]	2681.32847
4927	[P].GPPGPRGFQGTAGARGSHGERGPPGAVGP.[T]	2681.34035

4928	[G].GLGGGFGGGFGGGDGLLAGSEKVTMQNLN.[D]	2683.27805
4929	[S].QAGVQQPPATSTGGPAASAVSASVSTQVE.[P]	2683.31694
4930	[T].PPVAASDPAGPSYAAATLQASSAAASASPV.[S]	2683.32096
4931	[R].PGSPPRSGQAAVNKGSSNNRKMAEDK.[K]	2683.33288
4932	[Y].RAQPSASLGVGYRTQPMTAQAASYR.[A]	2683.33691
4933	[R].EPGGSRPVSAQRRVCPRGTKSLCQ.[K]	2683.36274
4934	[T].PGKLQDSSVGGQGAQGSQPKPAAAGGPHT.[L]	2685.33393
4935	[P].AGPATQPTGPLPQPACPPPAPAAGPAAPQ.[T]	2685.34535
4936	[A].TASIAGAPTQYPPGRAGPPPPMGRGAPP.[P]	2685.35658
4937	[L].QQPPPAPSQALPQQLQPPQHHPAPP.[Q]	2686.38484
4938	[D].GEGPRGPAEPFLAQARLAWEAQQAH.[L]	2687.34371
4939	[S].NRAVQSPNSSVPSPGLAGPVTMTSVHP.[P]	2687.35697
4940	[V].PPPGTAGPPAVHGLAMAPASVAPAPAGSGAP.[P]	2687.361
4941	[P].PAVCPPVVFMGTQVPKGAVMFVVPQ.[P]	2687.37578
4942	[S].ASSVSPLASAAPVADSSLTLSPAFSAVSP.[G]	2687.37742
4943	[P].RGPPPVPCQQVTASLRFGGGATPHLS.[R]	2687.38346
4944	[T].PGVPGKDGQAGHPGQPGPKGDPGVSGIPGA.[P]	2688.34885
4945	[S].GPAGKDGRTGQPGAVGPAGIRGSQGSQGPA.[G]	2688.35606
4946	[G].PAGGKASTHVTSSSLTIWPSPAGSPPPT.[G]	2688.36277
4947	[V].PGQETGLSDLAWAVGLQAYHHWRP.[R]	2689.32699
4948	[A].SSSSLSSKSSVTPSASGRAAQGSPSPVPS.[M]	2690.32276
4949	[G].PGPGPGPGPGPGPGPGHSMRLPVPQGHGQ.[P]	2692.31611
4950	[P].GSIPAGPPESVPAVPMASPLSLGAAGHGAP.[Q]	2692.37631
4951	[D].GEPDALTPPSAASASASAKAAEAGPPTDR.[G]	2693.30129
4952	[G].EPDALTPPSAASASASAKAAEAGPPTDRG.[V]	2693.30129
4953	[G].PTPAPAAPSQGSPLASQPPTQPQAPSAAA.[P]	2693.35293
4954	[P].GVSAYGNATSVQIGNISGYIDTPDPPT.[I]	2694.28933
4955	[A].AGRGAGGVGGGRWLTSRSLADPGSARQP.[S]	2694.39312
4956	[S].QPDKPSAPAAAAAAAQPPASHGPERSQS.[P]	2695.31828
4957	[G].ASPSPPAPSPRNGAAGQREGGGAGGPAGPGL.[V]	2695.32951
4958	[R].SPSKHGGPSAPGALQPLTSGSAGPAQPGSV.[A]	2697.35908
4959	[L].GPATPTVSSPQPPGDAAPELPLLEVEQ.[V]	2697.36177
4960	[T].QNPSPLGMGIGWAPLMAPPHPGFAGTP.[T]	2698.32686
4961	[P].GPPTSSVTTATTDPGASSLGKAPSNSGRP.[P]	2698.32784

4962	[L].PARLCGFAVPTPMVSITGVMSISFQ.[S]	2698.34013
4963	[S].DNLTHKASLTPAPGVPGGDTGESQVLH.[V]	2698.3431
4964	[V].GAGMAAAALAAEAGMVAAGAAVGATGAAVVGGGV.[G]	2698.36509
4965	[Y].SAAAPAGQAELSQERQNLFTGYFRS.[L]	2699.31722
4966	[G].GPGEPGPPRGPYAGYRTYGAELPATPA.[F]	2699.32124
4967	[A].GPAPGGPAQPPPPQASASDLQFSQLLGN.[L]	2699.34237
4968	[D].PLQEAAASEATATKEAARRANDGRGTG.[L]	2699.34556
4969	[T].GPSPGAPGLTNSSLLHQVGQGLGAPWGGS.[W]	2699.3536
4970	[P].QGHLGPQGPPGPQGHIGPQGPPGPQGHL.[G]	2699.35494
4971	[V].NIGSLICNVGAGGPAPAAGAAPAGGPAPATAA.[A]	2699.35697
4972	[G].APGATLMVQLQQLPLGGDGEEGGHPRA.[I]	2699.35697
4973	[-].MAGEQKPSSNLLEQFILLAKGTSGSAL.[T]	2701.44069
4974	[R].SRDPGAARSPQNQYPPELMRRFE.[L]	2702.32159
4975	[E].AKSAEEPAGRRDESGKGSWEARTLGG.[V]	2702.32409
4976	[P].ATPAFSAFSRALGAGHFSVPADYAPPP.[A]	2702.33616
4977	[Q].QPATGPQPSLGVSFGAPFGSGIGTGLQSS.[G]	2702.34203
4978	[A].GGIPSSIFGMAGQVPTLQSATTGGGGSPGL.[A]	2702.3454
4979	[I].PGPCPTVQLTVEPAPEEQASQDKQP.[S]	2703.29304
4980	[P].VSQGPLMGLNPRGMQGPPGPRENQGP.[A]	2703.30898
4981	[Q].GPVSQGPLMGLNPRGMQGPPGPRENQ.[G]	2703.30898
4982	[S].GPSGPPGPKGDDGIPGQPGLSGPPGPKGEP.[G]	2703.33728
4983	[D].FPKGMPPQLGPGRELEFGMVPSGMK.[G]	2703.34555
4984	[E].PGKAGTDGPDGKPGIDGLTGAKGEPGPSGI.[P]	2703.35841
4985	[G].KAGTDGPDGKPGIDGLTGAKGEPGPSGIPG.[V]	2703.35841
4986	[T].PSTAPGPHVPLMPPSPPSQVTPASEPK.[R]	2703.38106
4987	[L].RVSARMTQNLPGPLSTGPQGAQRQH.[V]	2703.38559
4988	[N].QGVGKDPTTPTVHLHDVQLEDPGPPA.[P]	2705.35293
4989	[S].SEPAVHAPGTPGTPASLSANSSLSSSGEL.[V]	2708.30096
4990	[A].LPDSGETRGPAEEQPRPHTAAPSPGGP.[A]	2708.30229
4991	[G].NVTLDHFGEVPGGLAGGGQGREVQWQ.[V]	2708.31755
4992	[P].SPAPAAAAATAAASTAAAAAATTTSAAATSAPP.[A]	2711.34824
4993	[Q].IPLADSEVEPSVIGHMSPITTSPHSP.[G]	2714.33417
4994	[E].GAQAAGGSSQPLGARPGSLGAPDAPSSPRP.[L]	2714.36048
4995	[I].RQAVMSAQRFVCSLPAELPAPGQW.[V]	2715.34939

4996	[L].MGLAGGVVGAGMAAAALAAEAGMVAAGAAVGAT.[G]	2715.36265
4997	[V].GGAVGAGLMGLAGGVVGAGMAAAALAAEAGMVA.[A]	2715.36265
4998	[S].VSSSLSSGSSLGLSLGSNSTVTASTRSSV.[A]	2715.36429
4999	[A].AAAGQPGTAPSGVPGAPPLPGMAIVKEEE.[T]	2715.36581
5000	[I].PGPQGPIGTPGEKGPPGNPGIPGLPGSEGP.[P]	2715.37367
5001	[G].ELPECARLAYGAGRDEVRPEEIAD.[Q]	2716.29952
5002	[G].PGPGPGPGPGPGPGHSMRLPVPQGHGQPP.[P]	2716.3525
5003	[G].GIPSSIFGMAGQVPTLQSATTGGGGSPGLA.[F]	2716.36106
5004	[E].PGRAATAPTAGGEPLSPPPPQEPAPGAPQ.[Q]	2716.36892
5005	[S].RQGPSGQPSGSIFQPQLVDEGPGLPPA.[Q]	2716.36892
5006	[G].EPGARGATGAKGESGVDGLMGPPGPQGPPG.[D]	2717.29477
5007	[V].SSAGRPASASPAPNATADGSKTSRASVDT.[T]	2717.3085
5008	[K].PEAGESPPPAPGTPKANGSQPPGAGSPPPA.[P]	2717.31655
5009	[K].GEPGKAGTDGPDGKPGIDGLTGAKGEPGPS.[G]	2719.31694
5010	[F].RGQAEDRQPVTVADYISRAESQSR.[Q]	2719.35064
5011	[A].EGVGAAANAAATSSTGTGGVAASGMAASGVVPG.[G]	2720.27918
5012	[E].PKGGPGPGSGGGAGTGAGAGGPGTGHLPPGAGTGP.[G]	2720.31353
5013	[N].AVGMSRPSPASQPFMPIGPPSEPTHL.[A]	2720.31708
5014	[D].FLGPQGIRGYPGMAGPKGETGPQGYKG.[M]	2720.36133
5015	[G].KPDTGTPRSHLPTAGKMTGDAAAAVVNG.[K]	2720.37844
5016	[G].APVSGTPASDPWAPAPAFSDPWGGSPAK.[P]	2721.29436
5017	[P].GPADSLPGACAANPEITITSAELPPGSQ.[S]	2721.3036
5018	[-].MSGPGTRSATPGAFTTGTPPAPRYHHSA.[V]	2721.3128
5019	[P].QNNPLPQGFQQPVSSPGRNPMVQQG.[N]	2721.31617
5020	[G].FGGGISGAVGGFGGLGGFGGAVGGGDAGILPAD.[E]	2721.32672
5021	[T].PGEGSLGAQPLWGQGQGPSWGPVAWGF.[M]	2723.30011
5022	[Q].EALEAVEGAAFFVSLDSEPAGLTRED.[P]	2723.30465
5023	[A].APGGGSVAAASAAMGAALASMAGLMTYGRR.[Q]	2724.33783
5024	[S].GLPAPSMGLEPPQEVPEPPVMAQELP.[G]	2726.34157
5025	[A].QPGPSGPPAAPSPVFLGLRRGPGLGPTM.[S]	2727.43991
5026	[N].GARTPSHMSASHSFPQLARNQQGPPA.[R]	2730.32774
5027	[P].TVCVTGPPTARPSEGPTTGPTGPPAAGPT.[G]	2731.33557
5028	[P].KPNGENAISARSDLNPANGSYPFQAL.[H]	2731.34343
5029	[G].PQGNAGPQGHLGPQGPPGPQGHIGPQGPP.[G]	2731.34477

5030	[G].GNLHGSLTEAAPPHADGWLPLLSSGPH.[S]	2731.35869
5031	[S].AAGSPAAQPLAQAWMQLLDPARESVH.[V]	2731.36206
5032	[D].GRDGPKGEKGEPGQGLRGSQGPPGKMGP.[P]	2731.36927
5033	[D].ARPACAGAAGRQSAGPQPSPEGVTGPRE.[L]	2732.32813
5034	[Q].QQPPAGAPQPGAVPPGVPPPGPHGPSPFP.[N]	2732.39434
5035	[P].GPGPGPGPGPGPGPGPGHSMRLPVPQGHGQ.[P]	2733.34266
5036	[P].GPSPRHGSARSSPTPEASGALGAALDSSL.[R]	2733.35506
5037	[G].RGASEGPPAPALPCPGAPTPSPLPEAAAP.[P]	2733.36648
5038	[E].PEGQRGPAVPGSTPLPAVNGQAPGGPTGLG.[P]	2736.40637
5039	[A].APEGPGAPDDPIGLFVMRPQDGEVTVG.[G]	2737.31377
5040	[G].PSGGPTVGGPPPGDEACSLGATQLAASSSL.[Q]	2738.29376
5041	[Q].PGSPSPHPLGHYPGPPDGRGPWEHPL.[I]	2738.32224
5042	[S].AAPQPSTSCSVLPATPDLTHNQLFW.[L]	2738.32428
5043	[Q].APTTSSQQAALESTQVVSQVAPSEPAPG.[A]	2738.34791
5044	[A].AAAGATPPSLAGHPLYPYGFMLPNDPL.[P]	2738.36468
5045	[G].EIGEPGLVGMQGPPGRPGPVGQMGPVGAP.[G]	2738.37527
5046	[P].AAEAAAAAGGGGATAAAARGGEAAAEVTGWPAG.[A]	2739.30811
5047	[F].PAYGHSFTLRDASNNGIGAPTSGAGPAGP.[Y]	2740.30738
5048	[E].GEAAPGAAGASVGAAAAAATPGTEDWKKGAE.[S]	2740.31728
5049	[S].GSPGASGGARDVRTEAAAEVAGAPGARAAC.[A]	2740.31796
5050	[C].PPNFTATPPASEHSRFSLEALTGPDT.[E]	2740.3213
5051	[G].GPTALCGVGGAGPPGHGGTAQVTHGGAGSALA.[Y]	2740.32198
5052	[M].KDPHMSKTAPPSGARSHPGSSQPSGAAP.[G]	2740.32198
5053	[W].PGSPQVSGPSPATRMPGMSPANPSLHSP.[V]	2741.31339
5054	[Q].PGSTAAPTPYGAYNGPVPGYQQTPPPGV.[S]	2741.32057
5055	[A].AAAAQPAGSPGETPAVAAESPELANYSSK.[C]	2742.32169
5056	[K].AVPSQSTFPSKTGGMEGGTAVATSSSLTA.[D]	2743.30908
5057	[L].GTSDANGLLSTPSVNGGGGSVLGSAGSGGGPVG.[S]	2743.31292
5058	[Q].GPPGKMGPQGTPGIPGMPGPIGQKGDPGE.[N]	2743.31781
5059	[F].QRGPTSTSVDNIDGTPVRDERSGTPT.[Q]	2743.32415
5060	[P].PAASTPAGPPSGGASPTPPAASPSGGSATRPS.[S]	2743.32817
5061	[G].QPWAVGTAEAAPARLPLVLTALWAAAV.[G]	2743.52938
5062	[K].GVNKYYPPDFNPEKHGSLNRYHN.[S]	2746.31207
5063	[N].GARTPSHMSASHSFPQLARNQQGPPA.[R]	2746.32265

5064	[D].RQKFCYEISAEPLACPVQNIWH.[L]	2746.32284
5065	[L].TSPGAAYAGLHNISPQMSAAAAAAAAAAY.[G]	2746.32534
5066	[T].QQLQASLMSPGQEELSQPPAQPPTAP.[G]	2746.33523
5067	[L].QRNGYDLAKAMSTLVPQGGPVLCRD.[E]	2746.37633
5068	[G].GPPSGTRGPGASVHDRNANSYVMVGTF.[N]	2747.29544
5069	[Q].AARDGPAGPGPEPGWEPLPAAEETLVY.[K]	2747.33114
5070	[A].PAAASPPAAGPPAAAPAVVCPAAAAQSAGSPP.[A]	2748.37737
5071	[G].WSRGDIIEKMLTDRRSADLNESR.[R]	2748.38458
5072	[L].APAASSAGGAAPSVQTHRPFLGTFAPGPQ.[F]	2748.38524
5073	[V].NDKAPAQARPTVFRWTGGGKEVYLS.[G]	2748.42162
5074	[I].EPPEELLANDFNLPQVEPVDLSFH.[K]	2749.33555
5075	[S].KNSSLSASPTSQQSSASLAAATATSEAVP.[S]	2749.34864
5076	[S].RVPCAGWPADQPAPAPPPCLGSLAAPP.[T]	2750.35414
5077	[G].GPSGVARLESWEVRDGGLGPAGDRSAGP.[G]	2750.36048
5078	[F].GSPATPPPPSPPSFPPHPDFAAPPPPPP.[P]	2750.36131
5079	[H].RAGEPETQKLLKFSITGVGGFMPGLT.[A]	2750.45456
5080	[S].LNMMKTYSHKAVILKPAAVETYIE.[I]	2750.46196
5081	[T].VMAAAAQISGHPEAGLGVWQGAGSWQR.[P]	2751.34199
5082	[E].WQRSGAPPPSGSAVSTAPQPKPADKMS.[K]	2751.35189
5083	[T].SGPAGPPGPPATQDPATLRYWQLLTC.[M]	2751.35591
5084	[Q].VYHSQQVGTPGSAISPDLLVDSSGSHL.[Y]	2751.35841
5085	[P].AAQPPQPANPPHGAHPLSSGPQPGTAPAT.[Q]	2751.35975
5086	[C].IFHMPCIQKWAKDSQFLVSSLTD.[D]	2751.3633
5087	[A].PPGTASNAAATAAVAFAKEEARLPHTDS.[N]	2751.36965
5088	[P].PQHQRGGFGAAAMGPYMPPGFPHPQS.[R]	2752.25073
5089	[Q].FLPQSQFPSSSGALSVSSVGMGQPAAQA.[G]	2752.32467
5090	[T].VHTETTIETKETSTTNKQTSTSAKE.[K]	2752.3483
5091	[D].GVNQAAPSFGFGNRQTVTFGSPGFPVN.[N]	2753.34304
5092	[K].ARGAGKSGESTGDTATPASRPPEQPEAK.[G]	2753.34489
5093	[P].KDLFHTAGHPQSNLSFKSCPTRQP.[Q]	2753.35764
5094	[A].QRQPSESGRHLLSEPNTPLSPPGPGD.[V]	2753.36014
5095	[I].VATASTPHSEGPLSTGPLPAATATTSPHP.[E]	2753.37406
5096	[R].AGPGESRGGFPRSLDQVFETLENHGP.[G]	2754.32303
5097	[A].ALEPGGAQQSLGGGWAGGGRAGPGPPEHSV.[P]	2754.33426

5098	[T].AGPFPFHLSQHMLASQGIPMPTFGGL.[F]	2754.35307
5099	[T].QDPSIYGREPKNSIKSYKKIQMK.[S]	2754.46071
5100	[L].AGVGSFMHSQAAVLSQLGSAENRPEQS.[L]	2758.32132
5101	[L].KCHITANPFKVDLVSEEEVVMSIN.[S]	2759.37426
5102	[G].RGAASRFACIQSGEAGTGARPGPARVC.[G]	2760.35291
5103	[E].ERSPASPIAETGPSAEPGGPAATSPSRPS.[P]	2761.33874
5104	[E].SPTSSLQASMDKTQGTWASLPREGLD.[P]	2763.3254
5105	[P].SAASVTSAGATSASSVHLPVSAPHGAGLMAA.[A]	2763.37302
5106	[V].AAEPPPGLLCGPLQVSDGGTAWSKVWA.[A]	2764.37631
5107	[I].QAMHPTLAGKITGMLLEIDNSELLH.[M]	2764.40081
5108	[L].EGPVPPGAAMGGTLLSLPTPDQLEPALH.[L]	2765.41784
5109	[C].PAASGTGGPLRPPGEISALANRTTKAMGS.[R]	2765.43629
5110	[P].VAPSASNAYPNTPYIPSAPSYSGQSQL.[F]	2767.32096
5111	[D].QGLSGFPGSPGEKGEKGSTGIPGMPGSPGP.[K]	2767.33557
5112	[T].SAQPAAATPASVSSPAGSPGPPGSTASLSTAS.[L]	2767.33807
5113	[R].QGEESKISAVDASPRNASPGLPNGEKE.[D]	2767.3493
5114	[L].ELWVGMPAWYVAACRANVKSGAIMS.[A]	2767.35169
5115	[C].PSTAITTSTPTSVTGLSSTATSERVSMP.[T]	2767.36659
5116	[F].PAQTSMTQQQNGYSFGDLGSAQLGQGP.[L]	2768.25805
5117	[S].TGPASPAVSTAAASPVLPTSPTSSPVGPAPP.[P]	2769.43051
5118	[Q].AGMFKGNSRQTVWRGYLTTDKEAPG.[L]	2770.37296
5119	[L].EPRVADGTPCSPDSSSVCVQGRCIH.[A]	2771.22941
5120	[R].APTTCKQLNGDHPGVMRRNGDGNFL.[G]	2771.31004
5121	[D].YTGVQEDRGSASGAGQAGSAASPGSGPGRH.[P]	2772.26803
5122	[P].GPGPGPGPGPGPGPGHSMRLPVPQGHGQPP.[P]	2773.37396
5123	[P].PGEPGPPGPRGPPGEKGDSGRPGLPGLQL.[T]	2773.438
5124	[I].AGMSNVLNPPSSAAFPTASAGSGSVKSQPG.[L]	2774.34138
5125	[F].SSPPSSLTSPSTPSSLGPSLTSTSGIGTSP.[S]	2774.3578
5126	[G].KDEMLQMIRHGATHVFASKESEIT.[D]	2774.36001
5127	[M].AAAPSHPAGLPGSPGPGSPSPSGGLELQSPP.[P]	2774.3744
5128	[-].MAAAPSHPAGLPGSPGPGSPSPSGGLELQSPP.[P]	2774.3744
5129	[G].AAPAAAEGPAPGSIFLAGAAPPAPCPASSSI.[L]	2774.38179
5130	[A].GGPSGGRPEPGRAAASGAAASSADPTALGGPAG.[A]	2775.34047
5131	[Q].GPEGIGKPGAPGTPGQPGIPGMKGHSGAPGP.[A]	2776.38352

5132	[-].MSGPGTRSATPGAFTTGTPPAPRYHHSAV.[V]	2778.37065
5133	[M].SGPGTRSATPGAFTTGTPPAPRYHHSAV.[V]	2778.37065
5134	[Q].GSVSSGQAHSLASLAKTWSVGGSRPQEP.[N]	2781.39144
5135	[L].SLTERGQCRVQHLRFPSVVDMLH.[H]	2781.40355
5136	[C].QGLDLETTTSLQSDQQELVVNYVSD.[V]	2782.3265
5137	[Y].KTYSQGAPEAPLSPSLNTPAPVAMPASS.[P]	2785.37129
5138	[P].PPEPAAAPTSPATAGSPATAAGPATATEEAK.[G]	2788.36356
5139	[T].PAAEGVGAAANAAATSSTGTGGVAASGMAASGVV.[P]	2789.33703
5140	[N].FYPTVGLSADMVAMLPKSGTPASPAHQ.[S]	2789.3637
5141	[K].PQFRFGQPSLFGQSNTLSGKSSGFSQ.[V]	2789.36417
5142	[F].GPSPGVEPVASMTSVASHPALGASSSSLPP.[L]	2790.36145
5143	[-].MEGPECDLGKSCARSEAAAAAQPAGSPG.[E]	2791.208
5144	[G].AGRTSRPGRTSDSPWFLSGSETLGRL.[A]	2791.42341
5145	[Y].VQGINDLVTPFFVVFLSEYVGTHPI.[T]	2791.47053
5146	[L].NLAGGPGHGDADGPISLDVPDGAPDPQRT.[K]	2796.31834
5147	[K].PSHRSSPVGPAPSSSQSEPPVSPAGGSVPG.[G]	2796.35472
5148	[P].HLMIAGGSSLQSGQLAGDPAPHPHPAHP.[P]	2796.36346
5149	[K].KEGEAAPGAAGASVGAAAAAATPGTEDWKKG.[A]	2797.37513
5150	[V].PTAGSVPSPSGSVPGAAAPFRPLFNDFGP.[P]	2797.3944
5151	[A].GDQERLHAQVQEAADVGFSHFQETV.[R]	2798.31286
5152	[I].QAAAGTQPGPAAAGEPSVSEDTLPCSADSG.[L]	2799.23737
5153	[A].TSQALQAPQVPPAAQEPEENQDLGGGPA.[S]	2800.33841
5154	[Q].QPLQDAYVQQYQHAVQQQQMLQQ.[Q]	2800.34714
5155	[A].TAAANNNFVNFGVADLNAVQSPGIPQGN.[S]	2800.36489
5156	[A].YSREAYHLPLPMAAEPLPSSVSGEEA.[R]	2801.34507
5157	[Q].SNVMPGPAQIMRGPTPNLQGNMVQFT.[G]	2801.35315
5158	[N].SVVYGSERTMLSQQVGSVKWPNSVM.[A]	2801.35968
5159	[G].EPGLPGPPGEGKVGEPGVAGPTGPPGVPGSPG.[L]	2801.41045
5160	[N].QPGQLSGEPPSTPAGAGSALSSHNGLEKQ.[N]	2802.36529
5161	[P].GPPFRSSQGAPGVPGPEEPETSARIGPE.[T]	2803.36456
5162	[N].LPQTGFSHLSRQGETLNLLETGYSR.[C]	2804.43258
5163	[A].AAGDAVAAASATAAVEPTELDFGAGEGHHL.[Q]	2806.32784
5164	[C].GLGAGSNFSGPLGSVSSRGSFSHGGGGLGSGV.[S]	2806.35031
5165	[S].QVAPGPAGKDASDIPGSDVGPWMSPLAW.[L]	2806.35049

5166	[H].GAAFGPPQGGFHPPYWQPGPPGPPAPPQ.[N]	2806.35248
5167	[G].LGAAPQAADPADPLAEAWGAQHSPRPDP.[S]	2806.35433
5168	[S].PASGAGKESPGAASTPRGGQSQQQRGGGP.[Q]	2806.35752
5169	[F].PGSPGEKGEKGSTGIPGMPGSPGPKGSPGSV.[G]	2806.3676
5170	[H].PLSDVATSVIQAEVTVAPGTGVMPPSGLP.[V]	2806.45429
5171	[L].YIVQVFDNTPAALDGTVAAGDEITGVNG.[R]	2807.37339
5172	[S].PASSQPGTVTSYGPTSSVALGFTSLGPSGP.[A]	2807.37339
5173	[H].GVTPCKLAVAEMRSEGKDSRALTFSP.[G]	2807.41786
5174	[A].TAPSGSPPVFGNTPAFGAVPAASSAIPAATP.[T]	2808.42028
5175	[G].PGAQLQQTPSVPPASAVGGACAGVPSPAHT.[H]	2810.389
5176	[K].VTDAPESTPPAPAPTSGIVGALMEVMQK.[R]	2810.39506
5177	[P].GSYKTNAAAAAAAAAAAATVNMGRPFQKN.[R]	2810.40023
5178	[N].GPSSLPGFPQNSNTLTPPGAGMLGFPPSA.[T]	2812.36106
5179	[A].AAAAAAYGRSPMVGFDPHPPMRATGLPS.[S]	2812.36576
5180	[K].MVQRSGPSGPPGPKGDDGIPGQPGLSGPPG.[P]	2812.36827
5181	[D].PWGAPVSMAAALPTAAPASDPWGGPPVPQ.[A]	2812.37631
5182	[S].LAQQYAHPNATLHPHTPHPQPSATPT.[G]	2812.39138
5183	[G].LAGHHGDQGAPGAVGPAGPRGPAGPSGPAGKD.[G]	2813.38261
5184	[G].QPGNRGLGFYGEKGEKGDMGLQGPGGIP.[P]	2816.37844
5185	[L].TPGLKETSMTLGFSSSTLTNTPSSALST.[H]	2816.38699
5186	[L].GEGNLSQPAVRLLGDVMSKDGFFYLS.[F]	2816.39236
5187	[N].HPFRAGETGDPAENWEAKNHSGKPAN.[S]	2817.30878
5188	[V].LGGAGGGNGPGDPAVPGDAVSRGVPGGSGDQAN.[P]	2817.31465
5189	[G].NAFGGLGNPSVTPNSVFGHKDGPSVQSF.[S]	2817.35908
5190	[P].PAGGGGSIHDLPLFTETSPTSAPWDPLP.[G]	2817.373
5191	[P].AAAPATDPWGAPVSMAAALPTAAPASDPWG.[G]	2818.35049
5192	[P].GPEGPAGFPGPPGIQGNPGPVGDPGERGPPG.[R]	2818.35433
5193	[A].GAVSTPEEPATPAGAVSTPEEPATPAVSTP.[E]	2818.36289
5194	[I].QGGPGSQGIQGPVSQGPLMGLNPRGMQGP.[P]	2818.37231
5195	[V].ADAYLGQARLSQHQSFPANNPLYKE.[A]	2818.39072
5196	[D].TGGLGRSISSPTTLYDRYSSPTASSTR.[R]	2818.39659
5197	[N].PGALTKDIQENGMKAGLAIKPGTTVEY.[L]	2818.46552
5198	[E].EEVPRDEGLGTASPHQATQVPEASKW.[G]	2819.35948
5199	[R].IGLFGEEGASALAAGSESPPATPESPAIPP.[E]	2820.39379

5200	[D].AAAAAPTVEDAVCAAVPVPEGAAAASVTVPS.[P]	2820.4084
5201	[L].RNPDICLLLSFQALRLSSSSRGKTT.[T]	2820.51487
5202	[H].KGTSSGATMAPASKATPSSVPSSETAPSAAS.[H]	2821.35201
5203	[S].QGASQPHPPGFGSISSSGALFSAGSQPAPP.[T]	2821.354
5204	[T].QPGAAAASAPPRAVPEPNTASATAQAPPAGP.[G]	2821.42274
5205	[G].AAAQRGPSSSATLPRPPHHAPPGPAAGAPP.[P]	2821.46047
5206	[Y].LPATSPPPHEVLDGLAQGLSHSLSVGLE.[N]	2821.47305
5207	[K].QEGARPDPSPAPGLPAASAPQQPSGGTPPS.[E]	2822.37037
5208	[S].SARAPGESSAISMGIVSVSSPDVSSVSEL.[T]	2822.37241
5209	[P].ARVCGSGRCGRRGVGLASSPPGSPGAQPA.[L]	2822.40092
5210	[Q].AWNSTIPSVAVGPRGSTVAAGGAWEPARG.[T]	2822.43325
5211	[P].AAPPSPQNEVLPQPDPVFLEALSSRY.[L]	2822.43593
5212	[N].DVLAVNTPKDAAQQDAKAEENKKEPL.[C]	2822.45304
5213	[P].QPQALGQMVTKTMVNTVVANPAFTYL.[T]	2822.45793
5214	[M].AAAALAAEAGMVAAGAAVGATGAAVVGGGVGAGLAA.[T]	2822.4829
5215	[K].PNLSPPHGSQPVGGPGVQGWPPSGGLLSM.[D]	2823.38827
5216	[A].AAAPTAAPGPAQPGHVSPTPATTSPGEKGEA.[G]	2823.39078
5217	[S].SIPPPSAGSAKTTAPSPTPRSHSPAAQQP.[G]	2823.43839
5218	[E].PGPEAGRAADSGERPLAASPPGAVKAEEH.[Q]	2824.39726
5219	[A].AGPETKEQLFIDDLPDLEDVNAGEPL.[G]	2825.37273
5220	[R].GEIGEPGLVGMQGPPGRPGPVGQMGPVGAP.[G]	2827.38656
5221	[T].GGIGDSRPPSFHPNVAGSRDGMDNETGT.[E]	2828.26526
5222	[F].PALSTQGPGGLTWAISAASTSPGEDASLSV.[T]	2828.39486
5223	[G].EAAAAAGAGAGETAVKVEGPGSPGVPGSPPEAA.[A]	2830.38536
5224	[K].KGADHTAAPPADGDDEEMMATEVAPSAM.[A]	2831.18045
5225	[C].PGAALAAEAPSTLPGGPGRLPGDEAFLTAQ.[I]	2832.45265
5226	[P].GGVAVDGGVALAGERAEAASVGALAGGTALGAH.[V]	2832.45986
5227	[S].QPGTPSPLDGPRPFPMAQGRHSSSLSN.[V]	2834.36385
5228	[D].AVAEAFEAGIAGGGQGVLGGAGGGNGPGDPAVPG.[D]	2834.37037
5229	[G].VPSSQPMQLSQQQQGVQPTAPSPQAAQ.[Y]	2834.37375
5230	[A].PQGGGLRWGISSTLPQSFARVTTSMTV.[A]	2834.46177
5231	[A].VGNDPGVITWRIEKMELALVPLCAH.[G]	2834.46917
5232	[V].DLPEVASGGLEGKLKGPKIKAPEMIIQ.[K]	2834.56959
5233	[S].VQDADLLSPGSMVNTAHGSNLESSRHL.[S]	2835.36899

5234	[P].APGALEPDAAATRAAPNPASLPNTLGSGYS.[P]	2837.40643
5235	[L].QRGSRRPASPVVSVSCLPREVAATGLS.[A]	2837.51627
5236	[A].PGAGSVGKLRSWTPPGRAAAPGPAGPPQLS.[N]	2837.51692
5237	[R].RPEELGLLQALGAGSGLQSPRAAPTPPGP.[E]	2837.52682
5238	[T].PLNPFLGSAIFITSYVRPVKFWER.[D]	2837.55012
5239	[P].AATVAATNPAKPVLSSGPPSQAPRTLPAAP.[A]	2838.54722
5240	[A].QRGGPGGGPHPHSPAFRVQVPYVGASAR.[Q]	2839.46114
5241	[S].QRSKSEPRDLRSSEKGQTASPRELP.[E]	2839.4769
5242	[E].QPPAQPLPGSPPRRAYYIYSGGEKIP.[L]	2839.48897
5243	[I].GPRGSPGPLGRPGIPGPQGPIGTPGEKGPPG.[N]	2841.51183
5244	[N].KMGQLGLGNQTDAVPSPAQIMYNGQPI.[T]	2844.40187
5245	[A].SHFLQGGPFPLPYPGPGAYLDVGSKPM.[Y]	2845.4018
5246	[P].GQHGLPGFPGFKGAPGNVGPPGPKGMKGDS.[R]	2845.42024
5247	[P].GPRGPEGAMGIPGMRGPPGPGPPGPVGDPGP.[I]	2847.36649
5248	[M].PGDMGPPKQGGTRYGSISSPPSPGPQQAP.[P]	2848.36827
5249	[R].PGPSASAHAGHPASARPGPSASARPGPSASAS.[S]	2848.38334
5250	[L].TGVPSSQPMQLSQQQQGVQPTAPSPQAA.[Q]	2848.38939
5251	[I].QGKHFEQIKEFMLAVIGMFSIGPDK.[V]	2850.4681
5252	[S].PDSGLPPSPSPSHWALAAAGGGGGERTPAPG.[A]	2851.37579
5253	[Y].NIGSLSSGTGAGAITMAAAQAVQATAQMKE.[G]	2851.39243
5254	[V].RSWGSAIAAWNIIAKTMLPLPHCLE.[G]	2851.47459
5255	[G].VEAAAEVAATEPKMEEESGAPGVPSGNGAP.[G]	2852.32546
5256	[Q].QPPATSTGGPAASAVSASVSTQVEPEEPEA.[D]	2852.34322
5257	[G].PGLAMETALKSPDVMKDKQTELGETF.[G]	2852.40562
5258	[G].HLAISSSATLGPGGLTRGREPGGVGHVAAQ.[V]	2853.50781
5259	[P].PPPPPPLPGGMGPPPPPPLPPGGPPPPPGPP.[P]	2853.51604
5260	[H].QGLAFQQKSYAGLPVDKRPAGDPISIP.[F]	2853.52575
5261	[P].PLLPVRAPGFSPMSSQPPAPPVSFPGAH.[P]	2854.47088
5262	[Q].VPAGGAGGGAGGSGPGLGRAGALSTGALPPLAPEG.[E]	2854.48059
5263	[L].NLVPDIEEIRPGSVVSKKGYLHFKE.[P]	2854.54615
5264	[P].QGPPGPQGHLGPQGPPGTPGMQGPPGPRGM.[Q]	2857.36208
5265	[A].KQAQETPQAPGGLGGACWGCSCGSEPPA.[G]	2858.22907
5266	[G].QPAPVGTMASAEPLTALSRWYLYAIH.[G]	2859.44981
5267	[P].EQRMSSVGSEVKTITEAGPSGGELPNSP.[R]	2860.36291

5268	[V].ELDSPPKNYLPKDNDDLFLSLSLMP.[N]	2861.42774
5269	[V].QGVHHGVNQAGKEAEKFGKDVHYAAGQ.[A]	2862.40301
5270	[A].KGTNVSMPNKLEQNGSGGKNMNLESVL.[S]	2862.40842
5271	[P].GRSSPAGGSPGKPGSTPHVSGLGSPGRYSPA.[N]	2862.42414
5272	[A].EPEELPTPMAQALPSPASTATPPPTPTH.[L]	2865.3975
5273	[P].PRGAASATRGGGAQSQRGTPGAGGAGRARGSS.[F]	2867.44398
5274	[D].ILVSSMRTNRNMATWRNSKVTDGTI.[D]	2867.46145
5275	[T].PPSGPGAGRAGVIGAAGDRARPPEDLPSEI.[V]	2867.47584
5276	[G].PQISAPDVDLNLEGPKIKGSLGASGEMK.[G]	2867.4819
5277	[K].QAPDIGDLGTVNLFKRPLPKSKPGSPH.[W]	2869.56829
5278	[P].QGPPGTPGTPGTTRYSRATLVKKVRFP.[V]	2869.57952
5279	[G].HALSSSGSVVSESSRITHPASSLDGTTLS.[T]	2870.41263
5280	[P].PSAQAQPSLAQGAAGPGGRESQPPRSPAEA.[P]	2870.41397
5281	[P].SLPPGPSAQSPQSSFPQPAAVYAIHAHQ.[Q]	2870.42202
5282	[T].TALCPSSSHRASPPETPTPGLDWGRPP.[P]	2871.38425
5283	[T].IDNPDQKTFVMKDLQPESVYEFLV.[T]	2871.41209
5284	[A].GPRGQASQAAPALGSLSSGRGQGAAAAARHL.[V]	2871.50446
5285	[P].KPEFMSKSLEELQIGTYANIAMVRT.[T]	2872.45833
5286	[L].QGPPTQPQVGADGLYSSLPNGLGGPSEHL.[A]	2873.40643
5287	[S].ASSPAQQGLGGQAQGQPSSANMASLGAMGKS.[P]	2874.34688
5288	[L].LEGHVHMSVVDLFIGGTETTASTLSWA.[V]	2874.39783
5289	[L].LGSGGPPASSSSSSDLQSILATMSVPAGPGSG.[Q]	2875.36257
5290	[G].PPGGMPGLPGRDGMTGAPGLTGERGEKGEP.[G]	2875.38254
5291	[A].QMGPPGPQGQFRPPGPQGQMGPQGPPLH.[Q]	2875.3879
5292	[G].PGKSGSMGPAGPPGPAGERGHPGSPGPAGSPGL.[P]	2875.3904
5293	[R].VAGDGPAGDSAARLAKQRSGTHKSAFSVH.[P]	2878.46667
5294	[A].TAQPSAPHEKLPVVIRVPKLAYFSVM.[S]	2878.60117
5295	[V].RGSVHASGSDFKAKTSCPPHVFGIAEH.[A]	2879.40057
5296	[K].GTPSLQFPRAASASAVLYPNLAELENY.[M]	2879.4574
5297	[F].ATAPSGSPPVFGNTPAFGAVPAASSAIPAATP.[T]	2879.4574
5298	[S].QLVDLLTDRFQQELEELLQSSFLD.[E]	2879.46729
5299	[E].GKMGRPGAEGARGLPGATGPKGDRGFDGLP.[G]	2879.46932
5300	[A].GKENVGSDLVPAQDQDIGIEVEAGVGQGV.[E]	2880.42213
5301	[S].SWTSRPVAPGFQYHPNLPMHAVIME.[K]	2881.39125

5302	[F].GDDIFKDPKVLQSYYYAVSDFSVKP.[P]	2881.42945
5303	[S].PGQQVHTPQSMPPPPQPSPQPGPPSSQP.[N]	2882.389
5304	[S].PVGNSTLSHTGGTVSHQTGFGTNIPNVHA.[L]	2887.40816
5305	[G].GPAGAAGGAAGGGPAAGPADHGLAGRGAAGDGPAAAL.[L]	2892.40955
5306	[E].PGAAGIPGEPGSPGKDGIPGVRGDKGDVGFM.[G]	2892.43087
5307	[P].GPPSPAASRSGMSTAAIKREVPTPGQGER.[F]	2892.47446
5308	[T].RASPSSHGQGPKENRSGPAHGPMALSPPS.[L]	2894.40745
5309	[P].TLATSAQPAAATPASVSSPAGSPGPPGSTASLS.[T]	2894.43778
5310	[G].NGLVGPGGSGAGPGGGLTPTAPPYGAGKHAPPQ.[A]	2894.45438
5311	[V].KGPSAQEPLEVVREVVSEDGTVVTIKQ.[V]	2894.54694
5312	[F].PSPASVPPAEGPVYLGKPAAAKAPGTGGPPR.[P]	2894.5523
5313	[G].GKVPDPGDGAPAAATEAALAAPAAEAASAAPDP.[P]	2898.41157
5314	[P].GPEGPPGPPGEAGLDGAKGEKGVQGEKGDRG.[P]	2900.4133
5315	[V].GPGPGGPGPAGPMGPFNPGPFNQGPPGAPPHA.[G]	2901.35256
5316	[K].GTPGSPSSKGSRGRQGHRGAQGALGEPGTPG.[P]	2901.44225
5317	[E].AGSPFREPLIKFLTRHPSQTVELFM.[M]	2901.54438
5318	[N].PDPRFPASKAQAAEGVWPGPAPADAMRL.[S]	2903.46211
5319	[N].RAPSPHVVQENLHSEVVEVCTSSTLK.[T]	2903.46798
5320	[G].PGLDSAPRTARTAPASGSAPRESRTAPEP.[T]	2903.47182
5321	[Q].ATIVMMPALPAPSSAPAVSTPESVAPVSQP.[D]	2903.48929
5322	[Q].APGGAAPAPSSALPGTAQLPTPGPTPAPAAPSQ.[G]	2903.48976
5323	[K].SLPGKPDTGTPRSHLPTAGKMTGDAAAAVV.[N]	2903.50437
5324	[F].PANTLHSQTPVKDVRAETASSHSPNLF.[P]	2904.45986
5325	[N].SRVVCPAGSLFPLPSTHISRNHVLEM.[N]	2904.49711
5326	[A].LQQGLERVHFGTSLQPGPGALAEWLEA.[L]	2904.50027
5327	[E].PGQGLRGSQGPPGKMGPPGNIGNPGLPGPRG.[H]	2904.50095
5328	[Q].AENPAISDCSCSKSDGSGPTTHGSADLPS.[P]	2905.22107
5329	[S].ADFLGPQGIRGYPGMAGPKGETGPQGYKG.[M]	2906.42539
5330	[C].PSPGALSNASAPVDFFLNGRVYADEAAVA.[E]	2906.43191
5331	[T].PGYGAPAPPAVQFIAQGGPGSGAAAGSGAGAGSGP.[N]	2907.40201
5332	[P].NLSPGASPASSQSNSLTVPTSPGEVQAPDI.[A]	2908.41705
5333	[V].WTVRGGAGGHERSRATAPRACGGLWGGQ.[V]	2908.42443
5334	[A].AADAARRVLVLLLLGVLSAGPGPGALATEH.[Y]	2908.67309
5335	[R].NGVAVQELGKEAVLLGEAAEATGFQLKPA.[E]	2910.55711

5336	[A].GPGGACKAEPRPVAASGGGPGGDEEEDEEE.[E]	2911.22826
5337	[G].LPPGMSVEGIRPGMEMNRMMPGSQRH.[V]	2911.36162
5338	[Q].KHSHDSGVPPVVDERTGSEGATASPSLGH.[H]	2911.3929
5339	[E].APGSTEPPARHPDTKGKGGDSATTGHERP.[E]	2911.40413
5340	[P].PGSLGSIYQHMGMPSAAPAGVPLPRFCP.[S]	2911.40519
5341	[Q].GPPGEPGFRGAQGKAGPQGRGGMSANPGFR.[G]	2911.41287
5342	[P].GMPGVKGSAGQAGRPGNPGHQGLAGVPGMPGT.[K]	2913.42065
5343	[V].PQASPGPPGPLSQTPPMQRPVEPQEGPH.[K]	2913.4312
5344	[A].ITMATTTPSPQMTSDCSSTSASPEPSLP.[V]	2914.26385
5345	[P].PGPQGQFRPPGPQGQMGPQGPPLHQGGGGP.[Q]	2915.4118
5346	[E].EAGLPVSADVGVDSGTSPSSSLPSQVPFET.[R]	2917.39492
5347	[T].GPGAGGEKAVSQGFVVTGEEKTGASDLPGQS.[A]	2917.41738
5348	[P].SGGRPEPGRAAASGAAASSADPTALGGPAGAEGP.[M]	2918.39871
5349	[V].RGHNCPKPVLNFYEANFPANVMDVI.[A]	2918.40763
5350	[G].SALVGTEVNRDSGHRAAGSAARGGTAHGAGAG.[G]	2918.43241
5351	[V].APAEPPSLVPQFYVSSQGQPPAGVAQPQA.[L]	2918.4683
5352	[T].GVAKMNTINAAEPHIVTVTMNGVAGRNH.[G]	2918.47235
5353	[G].NVATTPGSPSLGRHPGAHQGTLASNLHGNA.[V]	2919.45684
5354	[C].RQSKRSESTDSLGGLSPAEVTAIQCKN.[I]	2919.45887
5355	[F].PGFPGLDMPGPKGDKGSQGLPGLTGQSGLPG.[L]	2919.46692
5356	[T].SPTLSTTGPAPSAHLGSANKTINSSSPELP.[T]	2919.46942
5357	[E].RQVGRQSAFQYLQSTAAQPAPESSVRG.[R]	2919.48199
5358	[Q].ATIVMMPALPAPSSAPAVSTPESVAPVSQP.[D]	2919.48421
5359	[P].PPGMRPPMGPPIGLPPTRGTPIGMPPPGM.[R]	2919.48641
5360	[K].GWHKRYFVLEDGILHYATTRQDIT.[K]	2919.49004
5361	[T].VPTTASFGAKMFNLTSQVLEPALNGTQP.[E]	2919.49207
5362	[Y].EMRMMMDFNGNNRGYAFVTFSNKQ.[E]	2920.26335
5363	[-].MLNQQHPSQPAPRGRNRGTPPTPSLSVS.[A]	2921.50887
5364	[L].LNTQSPREQVWWQSGGGVATGEPGPQR.[L]	2922.42414
5365	[P].QNRSAPPPFPQGSPRSFPATGDLQELQ.[G]	2922.44929
5366	[P].PGSPGPRGNAGGPGLPGPPGPPGPPGQAALPED.[F]	2922.44929
5367	[M].GPAGPPGPAGERGHPGSPGPAGSPGLPGVPGSMG.[D]	2929.40096
5368	[G].GAGPPEPLSPAMSISPQATYLSKLIPHAV.[L]	2929.54919
5369	[I].PSDGVDLAAACGARAADVLPGPHTGDYAPL.[G]	2934.40505

5370	[G].ARGGFYSYGGGVGAGLGGSLGDGGLFSGGEKQ.[T]	2935.39692
5371	[Y].QLSADLQLEQRAATGPALDNKKCTPPI.[E]	2935.53058
5372	[M].KQRCEKISEMKMATPVDVLCNGFPV.[E]	2937.44534
5373	[-].MKQRCEKISEMKMATPVDVLCNGFPV.[E]	2937.44534
5374	[P].PAGPAAPSSAPASSSPAAPAGALDRHQDSPVT.[S]	2938.42895
5375	[T].TTTITTSSSRMQQPQISVYSGSDRHAV.[Q]	2938.43232
5376	[L].GPRLCSAYGVAAAKDHDIGTTNLHVEVS.[D]	2938.44758
5377	[P].GIVDSTAALAGFSEAVSSAPRAPGPYGPHR.[P]	2938.48059
5378	[H].SCGLPRLGGGTMKNSSSVSNTLTNGCVAN.[G]	2939.3768
5379	[A].AAAPQVSSSGFGSPAFGASTPGVFGQPGFGQA.[P]	2939.39586
5380	[H].PGSPGPAGSPGLPGVPGSMGDMVNYDEIKR.[F]	2939.4026
5381	[L].ATSAQPAAATPASVSSPAGSPGPPGSTASLSTAS.[L]	2939.42286
5382	[P].PGRDGQPGHKGERGYPGNAGPVGTAGAPGPQ.[G]	2939.42554
5383	[S].PGSPPGAEVEALPEAAALEVAEPPAEALGEA.[S]	2939.45204
5384	[F].KDAAVGMVTETRNSGVSVTEAMLGVTTEA.[D]	2940.42888
5385	[G].GPEDVRSRMPTGRMIAFTMALMGCLL.[I]	2942.41774
5386	[P].QAQAAPSPAEDLQRKDTATPDRGFFPGA.[A]	2942.43912
5387	[P].GPQGNAGPQGHLGPQGPPGPQGHIGPQGPPGP.[Q]	2942.44046
5388	[G].PGPLSGSQGPGQQCLGQAGLPGSVPASTHSL.[T]	2942.44249
5389	[G].PGPRCRGGGSGRASRPESRRMERSGRG.[P]	2942.45173
5390	[S].PGSPPPWPSPGSHGVQGSCVCSWGSPQPG.[P]	2943.29372
5391	[D].QRAGGERVEEGSLPEGSQVESLEVDSAK.[G]	2943.42901
5392	[T].LNQSSYQIHVTAHNSVGPSPASTVAVSGH.[P]	2945.45002
5393	[-].MAGRLWGSPGGTPKGNGSSTLLLNASQPAPGG.[G]	2947.50206
5394	[G].QSVAGMRFEGQHNQLGGNLRFEGPHGQ.[P]	2951.40778
5395	[Q].SPIGEGGSSTQLLMPVEPEELGPTRPSGE.[A]	2951.43026
5396	[G].GSMRPSLEAQGPAPSGERSRPPSGSAKEQ.[K]	2951.4388
5397	[V].ATAGPGRESTEGPPPLYNTNHDFKFSY.[S]	2953.37513
5398	[H].KDAEAPWLSKPTYDSAECRHHAAEAL.[R]	2953.38973
5399	[T].TAAATMPMGPSVADLAPPGEAALCLEEVAP.[P]	2953.39916
5400	[V].APGAFERAHPSPRANADPGPTGGTAPDSPR.[A]	2954.4252
5401	[G].VDQRGSSPTSHPRPGAPPSASSAASRAPPE.[S]	2954.44633
5402	[E].AAAAEPNPNAPGYLLVSRGFGADVVGSPKE.[T]	2954.50066
5403	[G].GRGVGPGQTLRQEEDRHGGGQKQGPAKPG.[M]	2954.50519

5404	[Y].VSPAPSAFQSLRTPSASALYTSLGSSISAT.[T]	2954.51056
5405	[K].QPSPNGNLFSSHLATLQGLKVFSEAAQL.[I]	2954.53705
5406	[A].EVAAAPAWPGLAEGRRRAGAMAEAGPQAPP.[P]	2955.50062
5407	[K].ISAVDASPRNASPGLPNGEKEDRFLTTL.[S]	2955.51704
5408	[K].QLGPTTSWSALPPPGLSPPYQPGPVVSPQ.[P]	2955.52508
5409	[V].PLPMPDSKSTSTAPDGAALTPPSPSFAATGA.[S]	2956.42444
5410	[E].PGKAGTDGPDGKPGIDGLTGAKGEPGPSGIPGV.[K]	2956.50105
5411	[D].KGAYSGGVFRSNLLTQDNGILTFSNLSP.[G]	2956.51631
5412	[S].GPQSLAGAKTPELEPLGAASPDLRASASPPA.[P]	2956.53744
5413	[Q].LKDTVENLPGKMNTELAESRLNLSVGQ.[R]	2956.54081
5414	[S].GARPHLLSVPELCRYLAESWLTFQI.[H]	2956.55019
5415	[G].GRASRQGPGDAQQGAAEDPAPSTPQARPPS.[S]	2957.42085
5416	[S].GPQASSTQITPDTPERVEASLQVSDKES.[Q]	2957.43343
5417	[T].PGAAGGATAASAAASVLGGSAAPATAGDTTKSENV.[A]	2957.44466
5418	[S].SAPGSRSGSRSGSRSGSRSGSRSGSRRGSF.[D]	2957.45052
5419	[P].GAREPPPPAPAPAHHHPEYQGQPVVSHP.[H]	2957.45538
5420	[V].PGAAMTIVHLIGGPMTGDTVAATGATTTAEI.[G]	2957.45945
5421	[H].SAPAGPPSSLGTKTPTEGTGPPARGSQPAEPG.[A]	2957.45992
5422	[P].GARGAGGGRQPQPSGSNRRGWNTTSQRY.[S]	2959.44907
5423	[L].NGIVDPAVMGGFAKYEKHAATNGRGGEGR.[V]	2959.45915
5424	[Q].AEAGGPRGTILMSSHKGPAVAQGNGAPASNR.[A]	2959.49151
5425	[M].RGAASCLEVMLAHGANAMSTDGAGYNALH.[L]	2961.34002
5426	[N].LTLSCPVASGGGNVIGDAHSLSYNFTINP.[R]	2961.4411
5427	[S].SSSLSSKSSVTPSASGRAAQGSPSPVPSMVQ.[K]	2961.4582
5428	[P].AAAPAAASPPAAGPPAAAPAVVCPAAAAQSAGSPP.[A]	2961.48872
5429	[A].AAPAAASPPAAGPPAAAPAVVCPAAAAQSAGSPPA.[L]	2961.48872
5430	[P].GMPGPPGIPGLPGRPGQAINGKDGDRGAPGAP.[G]	2961.51118
5431	[D].PSSKQGDANTIATVFDTVMRVHYPSALG.[H]	2962.47273
5432	[Q].WVRDQVGEQGLWALVNNAGVGLPSGPNE.[W]	2962.48059
5433	[R].QPSQPTTSQSLGQLQAHAASVPGPNPRAH.[G]	2962.4878
5434	[V].AHTRSGSSTPLVETVGFHGSRKRSRDH.[F]	2962.51027
5435	[L].KMPKMKMPKFTMPSLKGEGPELDVNL.[P]	2962.52727
5436	[V].SSPGSPPPWPSPGSHGVQGSCVCSWGSPQ.[P]	2963.28355
5437	[P].AAEAAAAAGGGGATAAAARGGEAAAEVTGWPAGAPG.[P]	2964.41945

5438	[S].PALAPSPYGGMHPPASRASPKQENGTMAL.[L]	2965.42949
5439	[A].ATVDHAGAAETEGVSITPQPGSSSVSRPGSL.[H]	2965.44975
5440	[G].GDAGLQPSPGTTLGPPAASTPAGPPSGGASPTPP.[A]	2965.45377
5441	[L].QPSPGTTLGPPAASTPAGPPSGGASPTPPAASPS.[G]	2965.45377
5442	[A].GPALELHNCMAKLLAHPLQRPCQSHA.[S]	2965.47058
5443	[L].PGLAGHHGDQGAPGAVGPAGPRGPAGPSGPAGKD.[G]	2967.45684
5444	[Q].PSAASMAAVAQRSMPLQTGAAQICARPDP.[F]	2969.43901
5445	[P].MINTPPPPPPGGFGSPATPPPPSPPSFPPH.[P]	2969.46546
5446	[R].RGSAAAPGASEFPPRPPIGQEPGGGGSPSAPL.[A]	2969.48641
5447	[E].FSGVPSLGPSEAMHGLPEGQPPRPGGPFAP.[E]	2970.45669
5448	[I].SSAKVTMASSLSSPVKQMPGHAEVALVNGS.[I]	2970.50232
5449	[T].STREEARGGAAEAAEARDRSASIREGPGGG.[A]	2971.43247
5450	[P].SAAPSSHPPASSSISIPGMGSRTSGPHGLGSP.[L]	2971.43266
5451	[N].KTQTFDKQGFHAGTPPPFSLPSALGSTGP.[L]	2971.49485
5452	[P].GSQPVPPPGTAGPPAVHGLAMAPASVAPAPAGSG.[A]	2971.50945
5453	[V].DMRLREGALPGAARPTPPSAQTPSHLGAT.[L]	2971.51666
5454	[D].RPLYDGPSRPSVARDGPAKMIFEGPNK.[L]	2971.52068
5455	[G].TVTDLAVDWLSGNIYWIGSENAHINIA.[S]	2972.47886
5456	[G].PSPGAMLGPSPGPSPGSAHSIMGPSPGPPSAGH.[P]	2973.39819
5457	[T].KGTAELMQQKEATTEQQLRELFEKH.[K]	2973.50984
5458	[A].GPLCPKAQVEEGVGGPTPPGAPPKASEPHL.[P]	2974.50912
5459	[L].EAVAWLAGGAVYRGETASLLCNISVRGGP.[P]	2974.52035
5460	[M].GPSELEMNMGGPQYSQQQAPPNQTAPW.[P]	2975.29345
5461	[K].TCMSEETSNQKQQQQQQQQQQQHV.[T]	2975.29663
5462	[F].VAGGMGPAASSHGSPVPLPSDLSFRSPTPSN.[L]	2977.44724
5463	[N].GNLGEAGPAPPTAPLPAEPAPASDANDNRLP.[S]	2977.465
5464	[Y].QPTPTQGYQNVASQAPQSLPAISQPPQSG.[T]	2977.465
5465	[T].LNGGGSGAGGNRGGGRDRDRRRGSTPWGPP.[P]	2977.47087
5466	[G].KNKTRSSSLKTLVRAKGTQAPAPGGGDPR.[A]	2978.66062
5467	[I].PPAMPPPPMPPGAGGHGPPSAGTPGAGHPGHGH.[S]	2979.38896
5468	[E].GPPGPAGSAGPPGYPGPRGVKGEDGFPGFKGD.[A]	2979.43839
5469	[P].PGSPQIPLADSEVEPSVIGHMSPITTSPH.[S]	2980.47206
5470	[Q].PWGSLCGSLVGGYGGGLGVGYTGGDGSLLPGN.[E]	2981.4098
5471	[A].PQGGMAALNMSLGMVTPVNDLRGSDSIAY.[D]	2981.41654

5472	[A].RGGRESYSIAGSEGSISASAASGLAAPSGPSSG.[L]	2981.41951
5473	[V].STPEQSATPAGAVPTPEQSATLAGAVSTPEE.[P]	2981.42219
5474	[K].ERGGLTGWGPPPSQGPRWHPPHHSQPE.[G]	2981.43023
5475	[Q].QQQQQQAAVAAVQQSTSQQATQGPSGQTP.[Q]	2981.43074
5476	[E].NVQEQLSADIFQQVSQIQNSVSPGMFS.[S]	2981.43093
5477	[T].SPTGETWTIPVYSAQPRGDPQQQSITH.[I]	2981.43879
5478	[Q].EAAAASGAVGVGPSQASPLSVSTEGGQQGAPSR.[P]	2981.45589
5479	[S].KGGYGGSSQTQNKAAGSGPGKGVSATSSTSGLP.[D]	2981.45589
5480	[S].GEQLLSVSSSVDQVFPSAAGKASGTDSPFI.[D]	2981.47384
5481	[R].GPSPRPGTAPYDAPAAFGSPLLGTGGSAFAPP.[L]	2981.4792
5482	[A].GLSEQKPSHRSSPVGPAPSSSQSEPPVSPA.[G]	2984.47082
5483	[E].AARAGPEGTSPPTPVPSTATGGPLEDSPTVAP.[K]	2985.47998
5484	[D].LGAPASPEGPESLPALAGRQPGPFGDTGGVAL.[V]	2986.52687
5485	[A].AVAMAATLTQQQQPATGPQPSLGVSFGAPF.[G]	2987.49313
5486	[D].LSLGGPDPDHPPRPPRETTAPAPPGSAAAGT.[P]	2987.49697
5487	[S].PLAASANTGESEGKKRTEALYTPAGGEKPG.[A]	2987.50687
5488	[L].PENIRVSSAKKTEEMLSNQMLSGIPEV.[D]	2987.51763
5489	[S].PSLGRHPGAHQGTLASNLHGNAVASPGSPSL.[G]	2987.51944
5490	[M].VAEVAVDSSAGTQQLLQRGYLSPEVLAEG.[S]	2987.53202
5491	[K].VGEVTYVELFKDAEGKSRVSGVVEFKD.[E]	2987.53604
5492	[E].RGVPGGRGPMGSPGLQGFPGITPPSNISGSPG.[D]	2988.51085
5493	[G].LPGMPGSDGPPGHPGKEGPPGTKGNQGPSGPQ.[G]	2989.42209
5494	[V].PGSPGFPGVPGSPGIMGFQGFTGSRGDKGAPG.[T]	2989.42612
5495	[L].GGVLNSASANTANHPEVSIATSSQATTTTTT.[T]	2989.43449
5496	[N].GPMLSPQAPGCIFPKAPGPCGPPADAVTSP.[P]	2990.4209
5497	[A].AEAAAAAGGGGATAAAARGGEAAAEVTGWPAGAPGP.[C]	2990.4351
5498	[S].LAPSNPSGSSEQRPDINLDLSPLTLGSPQ.[N]	2990.50653
5499	[I].KIEPPEELLANDFNLPQVEPVDLSFH.[K]	2990.51458
5500	[M].VQAPPGYVLVGADVDSQELWIAAVLGDAH.[F]	2990.52581
5501	[S].SQTVSPLSNYQRHGQALYGPPVASHPVT.[P]	2991.50714
5502	[G].PTSSKTTPTPGSTTGLPLPSTGPTSSPTAPPA.[S]	2991.5157
5503	[T].PFSQRPSAHTLDLGEGSPQALIASGSSALP.[S]	2991.51704
5504	[K].VKSDSGPQGHQRPGPVHRGRLSSKDVLP.[P]	2991.59836
5505	[S].PSPGGSPSGRSVKSESPGVRRKRVSPVPF.[Q]	2991.62351

5506	[P].GLPGRPGQAINGKDGDRGAPGAPGEAGRPGLP.[G]	2992.54599
5507	[H].KMGAPGSGIAEYLFDKHTVGHSGGSHQLP.[G]	2994.45266
5508	[M].GLSLSSQEVQQSLPQTPEGASAAVSGPSPGQ.[V]	2994.46506
5509	[L].GPAGEVQYVLLDDAHRTFHVNPMTGALS.[L]	2995.47307
5510	[H].PGAMLAMLDLLASVGSVTQPEHAMDLQLA.[V]	2995.49373
5511	[G].AAGASVGAAAAAATPGTEDWKKGAESPEKKPA.[C]	2995.51195
5512	[P].VGVSARGNARSPEDQLGKHGEKQTAGMKS.[P]	2995.51264
5513	[G].AAEPHTTAAFLGGAAAQEVIKIITKQFVI.[F]	2995.66152
5514	[A].ANPGAMLELGPPHGVSAEEAGLGPQMAGQPL.[E]	2996.46045
5515	[T].NAVTSHLNQSPASLSTQGYGASSLGFNSTT.[D]	2997.41845
5516	[P].PTSSAKESAAAPESYRERRDKMAAATSE.[G]	2997.43305
5517	[E].KPEGAETAAPTANGSIGDPSKEYVSLSHPS.[V]	2997.4436
5518	[W].PGSPPGGSRHGLGPGPSPSPSPEEPGAPGPGVQ.[G]	2997.44494
5519	[A].AASEEPLRPTALNYPHPSAAEAAFVSGFP.[A]	2997.47411
5520	[D].PNMPPMPPPGGIPPPMGPPHLQRPPFMP.[P]	2997.47585
5521	[V].PGERGPAGPPGPQGPPGEQGPEGIGKPGAPGTP.[G]	2997.48132
5522	[V].GPPGMVPHMGTAQGKFGLQPAPQSPWNLP.[Q]	2997.48621
5523	[P].GLPSSGQGGAASPGSSLGLYSPIEPGVVASGGQG.[P]	2998.47523
5524	[S].GAGPGGGLTPTAPPYGAGKHAPPQAFPPFPEG.[H]	2998.48462
5525	[P].AAPAGATLSTAPQQPLPPVPQQYQVPGNLS.[A]	2998.56326
5526	[P].GVGAAGGSLSFGASSTPAQGFVGVGPFGSAAPSF.[S]	2999.45338
5527	[S].QRSGYGAAAGAFPSTVPGLYNVNSPLYQN.[P]	2999.46461
5528	[T].QHGTLGSGRSSDKGPSWSSRSLGARCRN.[S]	3001.45177
5529	[G].PFLNTTLAQQQQQQHSGGAGALGGPSGGFF.[P]	3001.45511
5530	[E].DPGGRGPAPSLPAHPREGPWEVGGRSEHV.[M]	3001.47757
5531	[G].AAEALGHSFLDRASPAQGLPLDTAGGGHER.[G]	3001.48747
5532	[P].QPDPNQPKPEGRQMTPVKGEPLGVICN.[W]	3002.48225
5533	[T].VPGVSAYGNATSVQIGNISGYIDTPDPPTI.[I]	3003.49457
5534	[L].KGGDGIPGPPGLDGFHGLPGPPGDGIKGPPGDA.[G]	3003.49591
5535	[V].SSVGMGQPAAQAGVPQGQVPGTALPNPLNML.[G]	3003.50265
5536	[G].KSVAHEDIKYEQACILYNLGALHSML.[G]	3003.50667
5537	[D].KTQGTWASLPREGLDPHSLSAPSGVPEVS.[L]	3003.51704
5538	[A].SKVSGVEPSLAQSPMTTGVAPSLSQPSLTF.[G]	3003.53433
5539	[S].QPTSYTVAPASQPGMAPSQPGAYQPRPGF.[T]	3004.42578

5540	[L].KNGIEDAGLTDEVGNDPRPSQYTNGLPF.[Q]	3004.42828
5541	[Y].GAPSSLNKNTMPVQWNEVSSGTVDALASQ.[V]	3004.43165
5542	[W].GGPAPTPASGDPWRPAAPAGPPADPWGGTQAP.[A]	3004.43364
5543	[G].GPGEPGPPRGPYAGYRTYGAELPATPAFSA.[F]	3004.4588
5544	[L].PSPTSAPPGTPTQQPSTPQTPQPPAQPQPS.[P]	3004.46467
5545	[G].SASVSTGSRAGGAAGVGGEAGPPPEREGSGPAKP.[G]	3005.46713
5546	[L].SQQTVFTSGPNQALPGTTSQQTVPGHHVT.[P]	3005.47115
5547	[I].VQGVHHGVNQAGKEAEKFGQGVHHAAGQAG.[K]	3005.48372
5548	[T].SRSPAASGAAASPPPISNTTTQGNASPPLTSN.[P]	3007.47154
5549	[A].GPQGHLGPQGPPGPQGHIGPQGPPGPQGHLGP.[Q]	3007.50339
5550	[R].CTVGPSRPPTISQPGFSAGPSSSSSLPPPAS.[S]	3010.45747
5551	[G].SSTITVPGPPGPPGAMGPPGPPGAPGPVGPAGLPG.[Q]	3011.52952
5552	[P].GPPSPPSILSASSQGVTLSWTAPRGPGSAHI.[L]	3012.55376
5553	[G].AKGEKGEPGQGLRGLQGPPGKMGPQGTPGIP.[G]	3012.56836
5554	[R].GPSPGPARKEANGQSSKQDTSNHQVSPPR.[G]	3014.4787
5555	[M].PPGRTGMVTPVSQMAPVGLNVPRPGQVSGP.[V]	3015.55027
5556	[K].QGLSMRESPVSAPLEGLICRALPRGSPH.[S]	3015.5615
5557	[G].TAPTPVGRATPPPGIMAPPPGMRPPMGPPIG.[L]	3015.57292
5558	[S].PQTAVSPASEPTFLKFGVNAILSSAPRTE.[T]	3015.57858
5559	[S].ELPSETSVVPPSQPTTGGTEHGLVSREDL.[K]	3019.48546
5560	[T].PPPETFSRAVSSPTAGPAPPPPWPQPAPW.[S]	3019.5101
5561	[V].QPAKEQPVQAMFDHSPVGVGSKGVIPMNA.[K]	3019.51282
5562	[P].AAAPTSPATAGSPATAAGPATATEEAKGRNPRA.[T]	3019.51916
5563	[K].AEGKEAKTANGHGGEVAEGKGAGGALKPGEGKG.[S]	3019.51916
5564	[P].KETLSPPGNGCAIYRSEIISTAPSSWVV.[P]	3019.51935
5565	[R].GPPGPMGYTGRPGPLGQPGSPGMKGESGDLGP.[Q]	3021.41932
5566	[P].KGEPGSIPAGPPESVPAVPMASPLSLGAAGHGA.[P]	3022.53025
5567	[S].QPGDLSSSPLSQLSSSLSSHQSSLASTHTP.[L]	3023.45523
5568	[L].TSGPAERPEDGGQLDLQGKEKPGVDSDEL.[S]	3024.43924
5569	[Q].GPSGSTPRPSTTSSQGPALGQSPSGTTTPTQP.[P]	3024.45047
5570	[G].VMGTPGQPGSPGPAGVPGLPGAKGDHGFPGSSGP.[R]	3024.46323
5571	[-].MRTAHTQNPSPLGMGIGWAPLMAPPHPGFA.[G]	3025.49236
5572	[M].RTAHTQNPSPLGMGIGWAPLMAPPHPGFA.[G]	3025.49236
5573	[Q].GPSNGRQEKAPPAPADPPLMAGASPVHFAAA.[G]	3025.49486

5574	[Q].TQPGLPSSGQGGAASPGSSLGLYSPIEPGVVAS.[G]	3025.51128
5575	[R].SQPGGAAPPGHQMFAIQPGAAEGGQFLGGPPP.[G]	3026.45775
5576	[V].GARSAAPGGGSVAAASAAMGAALASMAGLMTYGR.[R]	3026.46047
5577	[G].ERVAYEVSVQEAAALYGGHTPAGMQTKY.[M]	3026.46765
5578	[P].EPPEPRTPGAGLARQCSGEVSSSSGFIRP.[E]	3026.47486
5579	[A].PAVMGGGRESGLPASRMHRTLSASEGRWG.[E]	3026.47956
5580	[D].QGVPGERGPAGPPGPQGPPGEQGPEGIGKPGAP.[G]	3026.50787
5581	[Q].KEVGPGPGGGGSGINPAYRTEDANEDTIGVL.[V]	3027.4654
5582	[A].GSEVPPVGSQADPVSAETLISEELAAMTLE.[K]	3027.47145
5583	[A].KYASSNPQPLPGQPGLQPPTMPAQQGVHS.[S]	3028.49453
5584	[L].RQGWQYEFRVTAVAPSGRGEPGPPSEAV.[F]	3028.50239
5585	[G].IQGGPGSQGIQGPVSQGPLMGLNPRGMQGPP.[G]	3028.50913
5586	[G].AGRPDALDIGERGTALAGAPPAQEAEGGQRP.[G]	3028.5195
5587	[G].DPGAPGAPGLAGPAGPQGPSGLKGRGASGPSVGSF.[S]	3028.52352
5588	[P].VGQQANNSPPVAQASVGQQTQPLPPPPPQP.[A]	3029.54392
5589	[I].FSPTMGGSSNSSLSLDSGGAEPMPANLSRC.[A]	3030.32376
5590	[N].WPHNLLTDSGGFQMVSLVSLSEVTEEGV.[R]	3031.47173
5591	[S].NPVTVAAAMSMRSPVNVSSAVNITSPMNIG.[H]	3031.50094
5592	[Q].PGQGRAPEAPRKEGESRSQPPRFKTGGPG.[D]	3031.55689
5593	[C].GPRAAASGGAAGAAGYPPVQYVQPMHKGPVGP.[P]	3032.51593
5594	[G].AASASPAPRESREPRGPSVLAQAPGEPFSL.[T]	3032.55482
5595	[S].LEATGGERNASPVGLPAQESLAEGLPLERA.[M]	3032.56472
5596	[T].KSMFQRTTYKYEMINKQNEQMHAL.[L]	3035.45359
5597	[A].VAGPMLPAGNAQQRTSGPAPAPPQGAPQPGLS.[G]	3036.53198
5598	[C].PQTSLSMQISGMSPQLQYIMPSPPSNAF.[A]	3037.44678
5599	[L].PAGGLGGSQPRLCGHPPPGDGGALTWGKPGW.[V]	3037.48497
5600	[P].PGRPGPVGQMGPVGAPGRPGPPGPPGPKGQPGN.[R]	3037.55372
5601	[T].EGPGVSISEERQSLAENSATTVVYNPYAA.[L]	3039.45416
5602	[P].SHIAGTSGSSLSFHSTVINHYRMRGHSP.[F]	3039.46021
5603	[L].QTIEVETQGDDLQSLLFHFLDEWLY.[K]	3039.46221
5604	[S].GPRGWTHPDVTEEFALPQLSWTPGGPAH.[L]	3040.47003
5605	[A].AAQPSTPAGTPRSGGGHSPAQPPSPERGMEE.[R]	3041.41298
5606	[A].PGTDTATPGAAGGATAASAAASVLGGSAAPATAGDTT.[K]	3043.44505
5607	[I].AAGGGGRAYGAKTDTFHPERLENNSSVLGL.[N]	3045.51368

5608	[T].STVTLAPGQEKSCAVEGRADTTTGSAAGPLL.[S]	3045.51572
5609	[I].AALTKAEERHGNIEERMRHLEGQLEE.[K]	3046.5123
5610	[V].KYLYLDENEIKSFKGADSRDMLGLEI.[L]	3047.53941
5611	[P].NPTPSPLDASPRRPPGPATSPTSSSISSSIS.[S]	3048.52325
5612	[-].QLGSAEDPAWLQLLRKDSSPPGPQPTAF.[C]	3048.54253
5613	[L].LGQPEAAMVLELPGQPVATTALELSGQPSGA.[G]	3048.55579
5614	[G].GKAAPGYHMAKLIIKLITSVAEVVNNDPV.[V]	3048.69144
5615	[V].RQPSLHMSAAAASRDITLFHAMDTLHR.[S]	3049.5207
5616	[A].AAPTSPATAGSPATAAGPATATEEAKGRNPRAT.[R]	3049.52973
5617	[S].GRESPSLAARGSISLYPSEFGKVPGEAFAA.[V]	3051.55342
5618	[S].EQRLFGAMELQVQRGEETPSKCIAYP.[P]	3053.48192
5619	[A].IHHPHDLARPSTLFSAAGAAHPPGTPFGPP.[P]	3053.54928
5620	[G].PQGPVGPTGKHGNRGEPGPAGAVGPAGAVGPRGP.[S]	3053.57762
5621	[S].PQATTPNPVPSSTEAQGVAGPAAEIPASGGHGT.[E]	3054.4763
5622	[A].DGRRPDLPGGLAGTRGGLSSSRGLYGGLQSP.[C]	3054.58277
5623	[A].GVAADWAAAGLADGARAAGHAGHGPAHGGLAGHGA.[A]	3055.47422
5624	[R].EVAQQAVDADVHTVGVSTLAAGHKTLVPEL.[I]	3055.60585
5625	[P].AATPSPTLGTPPLPQVTTRAPAPSAPTTPML.[S]	3055.61325
5626	[G].KMKMFQEVPAVDAFGPGIEGQVERFET.[V]	3056.4856
5627	[V].MIHSGSRGLGHQVATDALVAMEKAMKRD.[K]	3057.50267
5628	[S].TPDPALGGTPRPGPSPGPGPSPGAMLGPSPGPSP.[G]	3057.50984
5629	[R].LNGTPGEPSAWGATAGRAAKSMSAEDLLER.[S]	3059.48509
5630	[P].KPDEFGKHSEFLTVPAGSYSLSVPGHHH.[H]	3060.49624
5631	[H].PGPMGPPGLPGLDGLKGDKGNPGWPGTPGAPGP.[K]	3061.52002
5632	[Q].PGTLFGDDQIYNVIVTAHAFVMIFFMV.[M]	3061.5202
5633	[Q].GSRPESTNNFHSLYVKRHQGVSVLYAD.[I]	3061.52385
5634	[T].PGLEAPTAFTSEDLVVQVTLAPGAAEVPGQP.[R]	3061.57282
5635	[A].DQPAPAPPPCLGSLAAPPTFTETPPQYIE.[A]	3062.48156
5636	[E].ELCRRWMQLGAFYPLSRNHNGPGFR.[D]	3062.51008
5637	[G].AVWNTSSKGTISVASNGNPKADLNMTVTQA.[S]	3062.52114
5638	[P].GGAQVTVAGSSPPAVPSHSMVGITMDVGGSPIV.[S]	3062.52853
5639	[S].SAYSRGVFRRDTHKSEIAHRFKDLGE.[D]	3062.56672
5640	[F].FPPQLNGTANDTAGPELPDPLGGLLDEAML.[D]	3063.49794
5641	[S].IGDPTVFGNLPTDPEVTQAMKDALDSGKF.[N]	3063.49794

5642	[P].PEEPLLGELMVTGSSPDSLSLSWTVPQGH.[F]	3063.49794
5643	[G].RPGNPGHQGLAGVPGMPGTKGGPGDKGEPGRQ.[G]	3063.52896
5644	[G].GMIGSFSVGLFVNRFGRRNSMLMMNLL.[A]	3063.55114
5645	[T].QTQSAPALQGLSSLLQSVTGNPVPSSEAAPQ.[S]	3063.5593
5646	[S].QRGLLAAGMGDVVNIWAGQGKASLPSLEQP.[Y]	3063.60441
5647	[P].VISSSMGSPGLPPPAPPGFSGPVSSPQINSTV.[S]	3064.52958
5648	[V].KEFESHLDKLDNEKRDLIQSDIAALH.[H]	3064.5698
5649	[G].AGSPTDGVTLSPGGPVETPSLEAASGDLTTPPS.[T]	3065.47971
5650	[W].QRVGMVEAGSDTGTRGSWLRTGGPRAAYE.[G]	3065.49699
5651	[S].PAGGLSQDVASGRLDEKMPGLAGQAAGSGDRP.[R]	3065.50688
5652	[E].DKEKGEAMVETVVAKGGLNENSLQAEFR.[K]	3065.5208
5653	[L].SATVHAAAAAATAAAHSFPLSFAGAFPMLPPN.[A]	3065.53019
5654	[L].KAGSAQTGGREVRGGGGAAGGPEEARSWPGVR.[V]	3065.53721
5655	[T].RYSQLTILDGGFNSISKLEPELCQSLP.[W]	3065.56121
5656	[S].QAVMGRTRESVPSSGPTNVSVLATTSSSML.[V]	3066.51942
5657	[N].GGAPSPGLPAEALGSGPESPRLDSLEAGSPRH.[P]	3066.52391
5658	[L].PQPSAAGSQQPAGPPAVQPQAQAQPPAQPAPP.[P]	3066.53917
5659	[W].KRSPWCEAIIGAFSLPNQPSPRLMSSH.[L]	3066.54004
5660	[G].EPGSPGKDGIPGVRGDKGDVGFMGPRGLKGE.[R]	3066.54254
5661	[G].LPVVDSEILEMSPEKSDGIVEGIDVNGPK.[A]	3066.55512
5662	[E].GAKAQPSKHTGLGAISRGLGAEAGKQPASAHL.[V]	3066.65554
5663	[L].AGGPSAVLGLDVAPSTLLPIYDQDTGLVLLT.[G]	3066.66091
5664	[I].SAPLPVSASGPTLLTNVTPTLAPVVSAAPGPPS.[L]	3066.67214
5665	[A].IPALLCTILIFMDQQITAVIINRKEH.[K]	3066.68424
5666	[A].EPRPPMSSHLQSPPHAPSSAAFGFPRGAGP.[S]	3067.49553
5667	[Q].GSNQAIRFFVMTSLRNWYRGDNPNKP.[M]	3068.52717
5668	[Y].EELPFQGLTGTELQLSNGKSEPVVAPSSGP.[S]	3068.54225
5669	[P].AAAPATDPWGAPVSMAAALPTAAPASDPWGGPP.[V]	3069.47748
5670	[D].PGQEPAPSLPKGSESVSAEGPPAPLPTGSCAP.[C]	3069.48336
5671	[M].GPPGPQGQFRPPGPQGQMGPQGPPLHQGGGGP.[Q]	3069.48603
5672	[I].QGGPGSQGIQGPVSQGPLMGLNPRGMQGPPGP.[R]	3069.4993
5673	[K].RRPSVDYPGWGLGSRVWADADFSALPPP.[P]	3069.53296
5674	[M].IGAQTDQTVQEHLIEKYMLLPNQVWD.[S]	3069.535
5675	[S].YSSIYQSLTKDKKDMRNMPVSKIASF.[I]	3069.53837

5676	[E].VADEVTRPRPAGDEVLQDIQVMLKSEAS.[P]	3069.5521
5677	[E].NLLAEPWTRDGFLTETGKTRASTIFSTG.[T]	3069.56399
5678	[H].AQLGPREARADVGCQEKPLNLSLGAPHGGA.[G]	3069.56467
5679	[G].SQGLPGLTGQSGLPGLPGQQGTPGQPGFPGPKG.[E]	3069.57522
5680	[D].GAPGQKGETGPFGPPGPRGFPGPPGPDGLPGSM.[G]	3070.48396
5681	[P].SPLGGSALCGGKPEAGESPPPAPGTPKANGSQP.[P]	3070.48984
5682	[G].PGAAGADAAVPSTPAGQDLLSSGEPQPLPSSPGA.[E]	3070.49636
5683	[A].AAGSGPGPGVSAAPGPAAAANATPAEEGETKPAAAV.[A]	3070.5076
5684	[S].SQVPPMLQETDKSKSNAKQNSVPPSQTK.[S]	3070.54735
5685	[I].PGPPGQKGEMGIPGPKGERGPAGPPGRSGPPGP.[R]	3070.56395
5686	[I].PAAALDPNIATLGEIPQPPLMGNVDPSKID.[E]	3070.57653
5687	[G].GPPSGTRGPGASVHDRNANSYVMVGTFNLP.[S]	3071.47519
5688	[A].AGADAAVPSTPAGQDLLSSGEPQPLPSSPGAEP.[R]	3071.48038
5689	[G].LNFSGAYGAAAVASTTASTTTTTTVTTTTTTT.[T]	3071.49027
5690	[L].LGNEVLQVNPVRMEPKEIQDHVGNVEK.[L]	3071.59424
5691	[S].SPTQPGPVLYMPSAAGDSVPVSPSSPHAPDL.[S]	3074.47754
5692	[P].APNAGVLSSLASDGGPGSASTFTNPLLPLMSE.[Q]	3074.49867
5693	[L].PDGGGGRRPGASVVSSASMSALHTSSLRDYT.[P]	3076.48648
5694	[P].PGPPGAMGPPGPPGAPGPVGPAGLPGQQGPRGEPG.[L]	3078.52141
5695	[P].GGAQVTVAGSSPPAVPSHSMVGITMDVGGSPIV.[S]	3078.52345
5696	[G].PARFDGRPSSRLSHSSGYAQLNTYSRAP.[A]	3078.52525
5697	[P].VNTLQNWLAEFNMWLPAPEALPADNKP.[E]	3079.5346
5698	[P].SQLSQPPSSKMPPVSQEAKGTQTGVEQPR.[L]	3079.54769
5699	[P].LQPGGQSGFLPSGAPAQQMLLPMVDSQLPV.[V]	3079.5591
5700	[V].SPVCQAIPSPACFPQALGRGPSAPSGREVS.[A]	3080.50405
5701	[G].KVPDPGDGAPAAATEAALAAPAAEAASAAPDPPAA.[G]	3080.5171
5702	[L].AALSPPGVEGPASTQASLATSGSTHLAQMETL.[L]	3080.52047
5703	[G].PGASASTAATPGPAGLPRGYMAPTSPAASERSP.[S]	3081.50582
5704	[R].RGEPSGRDAQGRASTPAPPAWEREKTHH.[V]	3081.511
5705	[K].PPSGGTPKSEAAVWEWQAALGLICELGWA.[T]	3081.51387
5706	[E].PAAHAAAPTSLLPCRGGEGAGAAATAGVQEPGAP.[G]	3081.51706
5707	[S].GVSAASQGAGGGPPPAPPLPTAQGPSGGGTGAPSLAS.[A]	3081.52358
5708	[R].QSLPQNEGSLPPSSPPMPVIDNVFSLAPY.[R]	3081.52376
5709	[Q].KRYHEDIFGAVFPYEVKKDEAAGLPSD.[P]	3081.53163

5710	[Q].PTNVTLSSGFVADSGVKHHNGGGKPFQSQK.[E]	3081.55007
5711	[I].SRPASAPEIYSNLETLFCQLLKLQTSD.[E]	3081.55613
5712	[D].TPSGGVPAKETPTAATPGPPGTTRSSGHPSLAP.[R]	3081.55997
5713	[V].LNPPSSAAFPTASAGSGSVKSQPGLLGMPLNQ.[I]	3081.56736
5714	[L].HVEAGPAPAPHAPGGPPSNADLLSCLLGAPDP.[A]	3082.50509
5715	[H].PPGSAPATTFPGAAGSTKPALDWLMREGGEL.[P]	3082.53025
5716	[L].ALMGPPGLPGQIGPPGAPGIPGEKGEIGLPGPPG.[L]	3082.6394
5717	[A].VGGAVGAGLMGLAGGVVGAGMAAAALAAEAGMVAAGAA.[V]	3084.56387
5718	[A].SAVPGSPGYPGCSEGALRMDAEVAPDGGRPAG.[P]	3085.41021
5719	[G].PGASAGGIPSSIFGMAGQVPTLQSATTGGGGSPGL.[A]	3085.52589
5720	[H].GIQGGPGSQGIQGPVSQGPLMGLNPRGMQGPP.[G]	3085.5306
5721	[E].QRAEYNITITGHFPGHLVDVSGTGTLSQS.[Y]	3085.53375
5722	[V].FAVMHAQRTSGSGAPALYLPHSVSQSCLL.[F]	3085.53462
5723	[I].RLATAEENAGSVQLEVIEDLINSICNNI.[L]	3085.54702
5724	[S].QGEVFENTLVQNEPPAATELNVGNVQTTS.[V]	3086.49128
5725	[S].PPPPYPVTAGYPEPALHPGPGPGQVSGPGQAP.[V]	3086.53705
5726	[T].GARELDPTQKQPQEPAAAPGAQGPGSARPGF.[M]	3086.54023
5727	[I].RQMALSLEDTELQRKLDHEIRMREG.[A]	3086.54698
5728	[E].EKPAANPSGAPSSSSLQRPKKSDPALIPEQ.[E]	3087.60692
5729	[E].PAMGIPSAVVPGSMAGRMTTTVAPGSIAGGMAP.[S]	3088.49341
5730	[Q].GPAGPPGPPGPMGPPGLPGPMGIPGSPGHMGPPGP.[T]	3089.47941
5731	[Q].ADYSDKIKQMLGNPQGQPPGLGEGTTSGLGG.[A]	3089.48442
5732	[P].PGGSPGRSSPAGGSPGKPGSTPHVSGLGSPGRYS.[P]	3089.51475
5733	[T].YPFVTSSNCTVGGVCTGLGMPPQNVGEVY.[G]	3090.40055
5734	[M].GPPMGIPPGRGTPMGMPPPGMRPPPPGMRGP.[P]	3090.50789
5735	[G].PRATAEFSTQTPSPTPASDTPRSPGAPAPTP.[T]	3090.51268
5736	[G].LGAGFGGGFGGGLGGGFGGGFGGGDGLLAGSEKVTM.[Q]	3091.45781
5737	[K].PNYVQQATFKSSVYASWCISSCNPNPS.[G]	3092.38768
5738	[A].GRDFSLDLRSTGVGGSFMELLAHPHHGLA.[W]	3093.53231
5739	[T].PEGSKADSAVSSFYLDIQPSPDQSGLDING.[I]	3094.44874
5740	[A].GPVTMTSVHPPIRSPSASSVGSRGSSGSSSKP.[A]	3097.5331
5741	[G].GGNGPGDPAVPGDAVSRGVPGGSGDQANPRGPSAA.[G]	3098.46344
5742	[G].GGCALPVSGAAQWAPVLDFAPPGASAYGSLGGP.[A]	3098.50403
5743	[P].PGSAPATTFPGAAGSTKPALDWLMREGGELP.[E]	3098.52516

5744	[V].AVNFTLEEWALLNSAQKELYRDVMLE.[T]	3098.55031
5745	[A].LGAELNVLPFCSQFIPMEVISAPRHGSI.[I]	3098.58017
5746	[K].KKEQHSSHPQQTTLLDLMDALPSAGPAAQ.[K]	3099.55277
5747	[P].GPWPPGAPASEALVAEFLQDQNAPLISRAP.[Q]	3099.58981
5748	[A].LTFVDEVHAVGLYGSRGAGIGERDGIMHK.[I]	3100.56328
5749	[V].QRGGAGGGPGKPGMGGTQGRAEKPLAAGPPMAPG.[T]	3102.53199
5750	[P].PLFHCSDALTPPPLPPSNNLPGPPGPSGPAT.[Q]	3102.53533
5751	[R].ITYVQSAGGHALPLGTSPASSQPGTVTSYGPT.[S]	3102.53783
5752	[Q].GAGPLDYGPKGLPDPAEPVSYLNSGGKYVPS.[G]	3102.54186
5753	[F].SLSRWDSELGLPHLSASGCPRGLGPEGLPG.[R]	3102.54254
5754	[D].SSRPAPSLPEPHPGPDFFIPLGMPSPLVM.[S]	3102.54273
5755	[I].YTAFASNMKGNAICSGKLSISPRSVSRSP.[I]	3102.54591
5756	[A].AAEAAAAVVAAAAAAAAAAAAAANASASTSASSTVSGTV.[P]	3103.55019
5757	[L].HHTPPPASTAAGMPSLQHPAAPGVTPPQPAAP.[T]	3104.53706
5758	[K].ATSERSVPDPGSPLQPCPSPAPEHSLSAFS.[A]	3105.4582
5759	[S].GPSPATRMPGMSPANPSLHSPVPDASHSPRA.[G]	3106.49455
5760	[L].QRSPSDAGKSSGDEGKKPPSGIGRSTATGSFG.[F]	3106.51481
5761	[P].GPAPRASHTHSALMETLLDYQWDRPEI.[T]	3107.50034
5762	[F].PGSPGEKGEKGSTGIPGMPGSPGPKGSPGSVGYP.[G]	3107.51024
5763	[G].RQEGPAGAAGAQAGAREGVDRNSVPRRGDAM.[P]	3107.526
5764	[Q].ASPSPGQRPSRSGGSSPARGQPPLSGHPAQQA.[A]	3107.54778
5765	[R].SPSKHGGPSAPGALQPLTSGSAGPAQPGSVAGAGPG.[P]	3107.55046
5766	[V].TSAVSIMHSSLQQGKFDTKGIESTDEAKL.[S]	3108.55177
5767	[P].KSSSEPAVHAPGTPGTPASLSANSSLSSSGELV.[E]	3109.52839
5768	[L].GPQGPPGPQGHIGPQGPPGPQGHLGPQGPPGTPG.[M]	3109.53509
5769	[A].NAAVPDAAALEASSVHSYLPGASRGGEEVRE.[G]	3110.51374
5770	[K].QPQEPAAAPGAQGPGSARPGFMRRLLEPLP.[H]	3112.6109
5771	[P].LNESNGHAVPANSSLLSSLMNKMSQGSPNL.[G]	3113.49902
5772	[R].RGSGGGSASALGAAGTGVGSSAPSAEDFPPPSLLQ.[P]	3113.51341
5773	[S].GPPAPFPGEPGPGTTPSAPLGAPQAGPAPDHSAL.[T]	3113.53269
5774	[Q].ADVISQEPAMGIPSAVVPGSMAGRMTTTVAPG.[S]	3114.52682
5775	[S].GTVGEASTALSSAAQVALQSLSHAMASAEQQL.[Q]	3114.53718
5776	[V].QVSDKERHTQLEQMFRDIATIVADKC.[V]	3118.54083
5777	[R].QRRLHGLPEQFLYGTATKHLTYNDFI.[N]	3118.62211

5778	[L].PPYGDIHDPLNILDDGSRKQTSSFYADT.[P]	3122.47015
5779	[Q].QPAASTAGGQPPPPAAGGEQTAPHGRDASQALE.[L]	3122.48859
5780	[S].GSTPRPSTTSSQGPALGQSPSGTTTPTQPPNE.[T]	3123.4825
5781	[I].KMEPAEPEQGISGAEIVSGVVPTTNMEPPE.[L]	3123.48606
5782	[A].PALSLGPDHQSDLTERPEEFGFILDNVQ.[S]	3124.52218
5783	[H].CLVHGPAGMQLQTKCLTLSQKLACTRH.[S]	3124.56349
5784	[V].PGQQPAQPQTHQQMRSLNPLGNNPMNIP.[A]	3125.50036
5785	[G].PQGAPPQLVYWLEPSADSAAFGVHPFTGW.[I]	3125.51558
5786	[T].PGAAGGATAASAAASVLGGSAAPATAGDTTKSENVAP.[A]	3125.53454
5787	[R].KRGSIVSKLLASMMCKAGLTHLITMDLH.[Q]	3127.66108
5788	[P].GKMGPQGTPGIPGMPGPIGQKGDPGENMDDY.[I]	3130.39145
5789	[A].QPAASELTVSEGAVVTITAPVSMNVDSLQSL.[S]	3130.5824
5790	[G].AHGLREEPEFVTARAGESVVLRCDVVHP.[V]	3130.58508
5791	[V].GPSRPPTISQPGFSAGPSSSSSLPPPASSKHK.[T]	3130.5916
5792	[A].GEPGRAATAPTAGGEPLSPPPPQEPAPGAPQQT.[P]	3131.53923
5793	[N].TPAPGVGAAGGSLSFGASSTPAQGFVGVGPFGSAAP.[S]	3131.54325
5794	[L].PGSPGAKGEQGPAGHPGEAGLPGPSGNMGPQGPKG.[I]	3132.49157
5795	[N].IQQPGEDAVLEEALSTGGLDLIFMPGLGFD.[K]	3132.54456
5796	[K].STLSMLPMVLPGMAAVPQMFGVGGLLNAPMA.[T]	3133.56267
5797	[G].TLSFSLNGVSLGTAFENLSRGLGMAYFPAI.[S]	3133.60268
5798	[S].GNRASPAAASAVPGSGAAAGALASGGSKEEFVATF.[K]	3134.55013
5799	[S].QAASERPLWDISVRAPGCLEALRDPQAQ.[G]	3134.57999
5800	[G].APGRPLAPPQGHSQHSVEEPEGAAGRAPAAPP.[G]	3136.56712
5801	[P].QPPTAPTVPHPPPASAFGLGGALEAAESEGLGL.[G]	3136.59495
5802	[P].GVGGTGLEQGPSAGAASAGPQVSLYQGAPPAAEQG.[V]	3137.51341
5803	[Y].STARMTPPPGPQYGVGSVLRSSNGVVYSSVA.[T]	3137.56842
5804	[K].SRPQPGLESPATINEVVQEPAAEGVSSPYK.[I]	3137.57495
5805	[I].KQGWTPLRMFKEADNFFTSLGLLPMPP.[E]	3137.59509
5806	[Y].GHGLTSTQRFSHQTLQQTPMIGTMTPLGP.[Q]	3138.54591
5807	[K].LNGEAPRPAPAGPSSWADCVLHGAPGSPGKGE.[A]	3139.50141
5808	[C].GGGGGLTSLSNNPPLAQPTPENTAGAGDQPLPPG.[P]	3139.52906
5809	[S].SPQATTPNPVPSSTEAQGVAGPAAEIPASGGHGT.[E]	3141.50832
5810	[D].ADGREVPGSQQPGRPVACLSFCQLQKQQ.[I]	3141.53166
5811	[L].QGASQLPANASLAAMAAAAGLNPGLMAPSQFAAG.[K]	3141.54558

5812	[Q].PWGPPAPGPRPQPEPEPAAGEPGRAATAPTAGG.[E]	3141.55007
5813	[M].QQQPQPQPQMLQMRPGEIPMGMGVSPYG.[Q]	3142.45769
5814	[L].APGLGNGSGNLSEPVPAAPSSDLDVNTDIYSK.[V]	3142.51749
5815	[L].GAGPAGLQMAYFLQRAGRDYMVFERAPGP.[G]	3142.53495
5816	[Y].DPLQFQGPIYTHGDSPAPLPPQGMIVQPE.[M]	3145.52991
5817	[P].GIQGNPGPVGDPGERGPPGRAGLPGSDGAPGPPGT.[S]	3145.54096
5818	[L].GPRATAEFSTQTPSPTPASDTPRSPGAPAPTP.[T]	3147.53414
5819	[S].GSGISGSSGLLGSAGGGGGGGIGLGSLGGGGGLSSSLGGTA.[T]	3147.55125
5820	[Q].SGGTVHHLGPQSPAAAGGAGLQPLSSPGHITTTS.[L]	3148.57701
5821	[H].QLPGPGTTLSPMGTNAVTSHLNQSPASLSTQG.[Y]	3149.55317
5822	[P].ATQPTGPLPQPACPPPAPAAGPAAPQTTTASDL.[L]	3149.55719
5823	[L].LEAGADGTIVNNAGRTPLETARYHNNPEVA.[L]	3150.55628
5824	[Q].RAAEKSSKHGAEDRTQNIIMAMKDRMK.[I]	3150.55649
5825	[N].GAPTAAQVLIMGPDDFIVAMVSSLNRPFGSG.[L]	3150.55983
5826	[R].GQAPVVPYMRYGHSTVLIDDTVFLWGGR.[N]	3150.58295
5827	[Q].EVRGQARPPRDHLGAGVSPGAPLPAASAAEES.[P]	3150.6039
5828	[A].VAPASPGDRGSFRQLLTPSSQPAAGGPSRAAGS.[P]	3150.6039
5829	[T].PWTAHLGMYVQGNAKFVSRVRRIVVHE.[C]	3150.68942
5830	[Q].GNNLPSPGAGSWPPPAFPALPSSFLGTPDPAH.[L]	3153.54286
5831	[R].DGENLGRPLAGGRGPGGALQAEFPLGPGTPAHS.[G]	3153.58243
5832	[T].APSLFGQQTGSNVSTAAAAPQVSSSGFGSPAFGA.[S]	3154.5076
5833	[S].HWLGPYAATAEHLQEAPPRDEAEALEGPV.[A]	3154.52285
5834	[K].TPSSLDIEPGSFSSGRVSVEGSRIQGLDYN.[Q]	3154.52872
5835	[R].GPAGPSGPAGKDGRTGQPGAVGPAGIRGSQGSQGPA.[G]	3154.57366
5836	[R].ASSPQPQPSAVGHWRSSTVGNVSAMGSSDLC.[Y]	3157.44257
5837	[V].SQGSNSSSADPKAPPPPPVSSGEPPTLGENPDG.[L]	3157.45562
5838	[N].DAASRVAPDAAPGSEAPGPGPSTGALQERSPGSP.[P]	3157.51447
5839	[P].GPPGVRGMDGPHGPKGSLGPQGEPGPPGQQGTPG.[T]	3157.5232
5840	[N].FYPTVGLSADMVAMLPKSGTPASPAHQSPAP.[P]	3157.53328
5841	[L].QGASQLPANASLAAMAAAAGLNPGLMAPSQFAAG.[K]	3157.54049
5842	[L].PGAAVSASQAPGFVSFAGRGDIQPQLDSALQD.[V]	3157.55488
5843	[G].QVGHQDPHAAPARVGGQFPWVLQDYVSPT.[L]	3157.56024
5844	[E].ITQKMNFFTLHLYGDPRENYPFLPTV.[V]	3157.58155
5845	[P].PALSSAPGAPAAAAAASMSAPAPSHPLSDVATSVI.[Q]	3157.5834

5846	[R].HGQALYGPPVASHPVTPSLHSGPSPQMPLPT.[S]	3157.58876
5847	[G].GPSVGIKGLGQGAGESPGSGERKAPPAGREEAGP.[E]	3157.59848
5848	[M].PGQPTLMSNPAAAVGMIPGKDRGPAGLYTHPG.[P]	3158.58738
5849	[P].GPPGPAGPRGHPGPSGPPGKPGTGSPGPQGQPGLPG.[P]	3158.58785
5850	[I].PGEALPTAQLRGETGSVPQSSNSKNSGKLTF.[K]	3158.60764
5851	[L].YAEVQKHRMQQQQRDHQQQPGEAEPL.[T]	3159.5137
5852	[P].TAEERSPASPIAETGPSAEPGGPAATSPSRPSP.[P]	3159.51889
5853	[G].KPGLPGMPGSDGPPGHPGKEGPPGTKGNQGPSGP.[Q]	3159.52762
5854	[P].HSTPRPVSDGGKMVNAAVNTYGSAPSGSRSR.[T]	3159.53483
5855	[H].HGDQGAPGAVGPAGPRGPAGPSGPAGKDGRTGQPGA.[V]	3159.54269
5856	[E].TTHLAATGSGPTVAETTTTFNTLAGSPFAHVT.[T]	3159.5593
5857	[V].PGNRGLNQGGEGHYAIPGAPARIEAMPWAR.[A]	3159.56534
5858	[L].SGPGLAPAASSAGGAAPSVQTHRPFLGTFAPGPQ.[F]	3159.59702
5859	[P].GPSPQFQSPPAKQTSAFSKQMPHHPFPSP.[A]	3161.52616
5860	[P].TPTNSTAKTTTLPSTTTTSTTATTSGTTNTTL.[T]	3161.55433
5861	[P].QGAGRSGQNLLGQASATSHIYQGPESSLPGPP.[S]	3162.55628
5862	[Q].LSQSPHSVPGGPQAQATMTPPPNLTPPPMNL.[P]	3162.57106
5863	[E].AASSLVQTAESVAVGMTSLEAARAPAKNSAYP.[L]	3164.58922
5864	[E].TSDNISPVASPVHTGFLVSFMVDARGGSMR.[G]	3167.52484
5865	[Q].PGAGQPVVMQPRMYPRGLVQPGMYPRGLV.[Q]	3167.64273
5866	[S].GPSQGPSGSTPRPSTTSSQGPALGQSPSGTTTPT.[Q]	3168.50397
5867	[L].PQDSLPEPSMLLTSDQTLGSNLQNEGPQR.[G]	3168.51136
5868	[G].GPGDKGEPGRQGFPGVSGPPGKEGDHGERGPVG.[Q]	3168.52056
5869	[S].LDNGRTWQPYQFYAEDCMEAFGMPAR.[R]	3169.36009
5870	[V].AADWAAAGLADGARAAGHAGHGPAHGGLAGHGAAAAG.[V]	3169.51715
5871	[D].PALASPEMPTLESLLEPGVPAEGLSRSYSR.[P]	3170.6038
5872	[S].GAQPPELPGPPGFPRAPSAGADGPLAVYGWGAL.[P]	3170.60579
5873	[C].KPGDSPPPQEQLENDIATSFEAFQRHSF.[D]	3172.49703
5874	[T].EAGAGAGRRGSAAAPGASEFPPRPPIGQEPGGGGS.[P]	3173.54711
5875	[P].SPPPPYPVTAGYPEPALHPGPGPGQVSGPGQAP.[V]	3173.56907
5876	[E].AKSAEEPAGRRDESGKGSWEARTLGGVPQF.[K]	3173.57226
5877	[V].LQSPGPSPETPSPPSDAHPRPQSSTLGAHNQ.[A]	3174.51989
5878	[S].PGAKGEQGPAGHPGEAGLPGPSGNMGPQGPKGIPG.[N]	3174.53852
5879	[D].QGAPGAVGPAGPRGPAGPSGPAGKDGRTGQPGAVGPA.[G]	3174.61513

5880	[N].QLPGLLGGVGPMHSVVQLFQGFRDLLWLP.[I]	3174.72849
5881	[A].QTSPSPAPKAPRGSSSQGPSQTSTPTDVTAIH.[L]	3175.56142
5882	[Q].SLGTYLQGMASTLSPSQEPPPPPGSQVPPASP.[S]	3175.56161
5883	[V].TGPPTARPSEGPTTGPTGPPAAGPTGPPTAGPSAAP.[T]	3175.56544
5884	[I].AVQLEEQQTLEEAISVATAAMQQGAVTLET.[A]	3175.56748
5885	[Q].PAPSAGKAADPWAAASAAKPVSSSGSFDLFSNL.[N]	3175.56947
5886	[S].GLNTGAALSYVHANHFTEAGGSRIRDHVPQ.[L]	3175.57802
5887	[A].WPGRLTSEVDLKTAYPESGHTVFSAAGSVL.[S]	3175.60585
5888	[S].FHVTPLKLMVSPTSMAAVPVGNSPALSSSHP.[V]	3175.62785
5889	[P].NQPPRSAWGSAAREEGLGRCASLESSDCE.[S]	3177.40725
5890	[P].GPSGNMGPQGPKGIPGNPGLPGPKGEMGPVGPAGN.[P]	3177.55681
5891	[K].GSAAAVAAAAAAAAAAVAEQVSAAVSSATPIATSGPPA.[L]	3177.63861
5892	[E].GPRGPPGRAGEKGDVGSQGVRGPQGITGPKGGPP.[G]	3177.66241
5893	[P].QPPPRTLFAPASAAAAAAAAAAAAAKGALEGAAGFA.[L]	3177.68036
5894	[N].MKQVKDMYLIPLGATDKIPHPLVPFDGPG.[L]	3177.68391
5895	[Q].PRAPPSTSRPLSGSPRKGGMIRDTQIQVST.[A]	3177.69094
5896	[F].TYVFNKAKHLNSNISLIMPFHALRLYS.[H]	3177.70301
5897	[T].AAAAPQVSSSGFGSPAFGASTPGVFGQPGFGQAPA.[F]	3178.52285
5898	[L].QPAPQSPWNLPQVNQQMAGLSLGGVASAAGFG.[P]	3178.57384
5899	[P].VTQPGTLQPPMVLLDFPPLACFLNNILVA.[F]	3178.70431
5900	[M].GQGLPQGSVIDSVAGAVDSRIPPCPVNSSVDT.[A]	3179.56373
5901	[G].VNVNILSGMGNGTIASSAALNSAASAAAGMTVGSV.[S]	3179.5671
5902	[A].TSAQPAAATPASVSSPAGSPGPPGSTASLSTASLTP.[S]	3179.57026
5903	[W].GEGVSSGVGLHVRAGGQDENRTLPATSSSTPR.[R]	3179.5788
5904	[R].QSTVSFRSGGSRSFSTASAITPSVSRTSFTS.[V]	3183.56651
5905	[T].SKPSSFETTAFETKKLSHLTPGLKETSMT.[L]	3183.62421
5906	[P].GQPGEPGARGEPGDPGLPGRPGTSIGDEDEKR.[G]	3185.52062
5907	[Q].QTELDVVPGRDGLTSYNHSQVSVQPVTTTG.[P]	3185.57092
5908	[P].GAPGMPPGIPPLMPGVPPLMPGMPPVMPGMPPG.[L]	3187.57074
5909	[A].AAPHFHFVNNSRSATQVGNKPEAVSSSVNH.[K]	3189.55728
5910	[Q].PGPVLYMPSAAGDSVPVSPSSPHAPDLSALLC.[R]	3189.5595
5911	[P].PGGGAGGGGLHVAIPDSVLTPPGADGTSSSATATTR.[K]	3189.57707
5912	[A].GRPLAELCDHNAKVARELGRNQVAQTWT.[M]	3190.62867
5913	[D].QLEGTPILSEVSLESFSTLAPEPVSGGLYGI.[D]	3190.64057

5914	[L].PGAAAAQAASWYLNHSGDLNHLSGHTFAAQQ.[Q]	3191.50418
5915	[E].EQKPEAAEAAEERLDCCQDGCSDSMIGH.[N]	3193.29254
5916	[A].DLELPRSRSLSWSPGHTPRVEGGPAPAPAGV.[V]	3193.65012
5917	[L].KAAYNAYKAWLRKHGEEQQLPAVGLTNH.[Q]	3193.66537
5918	[A].KTHENEIIAVSALVHHSFALDKAPPQPPF.[Q]	3193.67929
5919	[P].QAAPGPATVQQKITAQQIAAQGQPQKVTYAT.[Q]	3193.6964
5920	[F].QSIHDVLKSQAGPFVSTGKTVTFRPDPALP.[S]	3193.70042
5921	[T].AEAAAAALGTVAAAAAVAGLATTGPAKELEAERSL.[M]	3193.7063
5922	[Q].AAAAGQAAQGKTTLPSQGPVQRPSRLVFTDVA.[N]	3193.70763
5923	[T].NGLPFQSSASSLNPSKNETSQPTTSGSFPIN.[E]	3194.52364
5924	[P].AVHGLAMAPASVAPAPAGSGAPPGSLGPSEQLGQAG.[P]	3194.58989
5925	[P].VTQPGTLQPPMVLLDFPPLACFLNNILVA.[F]	3194.69923
5926	[G].ATGPPSTVVLGPQQSQGLIVTERVYAPASTLG.[N]	3194.70557
5927	[L].PVSQGKVSVHGRIVYVSQQPWVFPGTVRS.[N]	3194.72216
5928	[S].APAQLVGGVGQDGVAELGVRPGGGPVSVELVGPR.[S]	3194.72803
5929	[Q].GNSSRGPTPGSPNQADVLMVYAAAEGCVAYR.[D]	3195.49461
5930	[P].SRLSPPMLEEMAYSAYVPQDGTMLTALHS.[Y]	3195.51592
5931	[H].PGLHPYGPPPGMALPYNPMMVVMMPPPPPP.[V]	3195.53607
5932	[A].AAAAAAVAEQVSAAVSSATPIATSGPPALPPPPAAD.[I]	3195.6532
5933	[A].GHLHASSLMANTSLHGSAPMDGLTPLHCGAR.[S]	3196.51971
5934	[N].AGPVGTAGAPGPQGPVGPTGKHGNRGEPGPAGAVGPA.[G]	3197.61988
5935	[M].QVQPQQANAGVGQPASGESSLIKQLLLPKRG.[P]	3199.75458
5936	[H].ANVPSAGGSASPGLPPFALKPDATRVLPGVLSR.[L]	3199.75861
5937	[V].RDPSQAPPVDVHQPPAFLHKLLQLAGVRL.[H]	3199.7851
5938	[D].DGPETGISSWPETEVSSETSGVPSGENSSRH.[P]	3202.40431
5939	[E].PQGGPGGGERVGSSMWPEPRVPLDLGLDCPS.[T]	3204.52009
5940	[L].SVAEDSFLVKVCCRYTYGKPMQGVAHVS.[V]	3204.52749
5941	[P].GPEGRAPHSAIEEKVMKGIEENMLRLQGQ.[E]	3204.62523
5942	[P].SPLGMGIGWAPLMAPPHPGFAGTPTMWPLW.[P]	3205.54604
5943	[W].KGEGAPGQPAEDSVKQEGLDLTGTATTATSFAA.[P]	3205.54952
5944	[K].PGAMGMPGAKGEIGPKGEIGPMGIPGPQGPPGPH.[G]	3205.55912
5945	[P].GPRGPEGAMGIPGMRGPPGPGPPGPVGDPGPIGFG.[P]	3205.56698
5946	[G].PGSRTPPSAPSQSRVTSERAPSPASRMVQAP.[S]	3205.61308
5947	[Q].PPSMPPQPNLQPPPPQPHLGVGSAASGHLGRS.[F]	3205.63236

5948	[R].PGPLGQPGSPGMKGESGDLGPQGPRGPQGLMGPP.[G]	3207.56738
5949	[S].PSPPVSLSSHFLQQPQGHLSQSENTFLGTS.[A]	3207.57053
5950	[E].AAASGEAGFVLEAVKAEPFEPKVDPEVPPAEG.[V]	3208.60485
5951	[H].TGLSGSGVAFVCAFSEALAEGAVKMGMPSGLAH.[R]	3209.5428
5952	[T].PTSLPAAMPVCVMCRTPALLWTEPSTGIH.[H]	3209.56143
5953	[E].SLSSYLQSAGLSSIPGPPGPPGPPGPRGPPGISGA.[L]	3209.65895
5954	[D].MDFLTRQKKLQAEAKMALAMAKPMAKMQ.[V]	3209.68519
5955	[G].ARGCSHIGVLKALEEAGVPVDLVGGTSIGSFI.[G]	3209.69871
5956	[T].QLQPVAMQHKSPVLQGPHNRAISVSQSRT.[P]	3210.69127
5957	[F].CPLSLSAALGMVRLGARSGSARQIDQVLHF.[N]	3210.69867
5958	[K].NHMKVHASKLGPLRAPGPGSGPARAPQPPDLG.[L]	3210.70653
5959	[S].QVGLRTQDTINRIQDLLAEGTLTGVIDDR.[G]	3210.70769
5960	[P].TVCVTGPPTARPSEGPTTGPTGPPAAGPTGPPTAG.[P]	3211.56882
5961	[S].AVSSSGSPASVMTSIRAPSTTGSLGINSVTGTNT.[M]	3211.57469
5962	[N].AQMRLGGLTQAPGNPVLAVQINQDKNFAFL.[E]	3211.70446
5963	[Q].PGADSSFPPALAEGYRYPDLDTPKLDCFL.[S]	3212.52449
5964	[G].PAGNAASTAGPFPFHLSQHMLASQGIPMPTF.[G]	3212.5292
5965	[V].AANPGAMLELGPPHGVSAEEAGLGPQMAGQPLE.[A]	3212.53507
5966	[P].GPQGHLGPQGPPGTPGMQGPPGPRGMQGPPHPH.[G]	3212.53775
5967	[G].GSGLEATYSITALGEEPVQGGAHPGCVGWVGR.[L]	3212.54294
5968	[G].QAGFGPMLGGGSSPLPLPAGGSSSVGGSGGFGSLHQ.[H]	3212.54294
5969	[A].VGQQPSASQVSPTEPRSSPSPPPSPMEPPEK.[S]	3212.55283
5970	[P].SSRQEPPTANGKGLVSRKTSPLEPGTGRSMA.[A]	3212.64405
5971	[S].KAGGEDQLWRPYYSYKPKRKAGAAARASS.[G]	3212.67119
5972	[G].AGPAGLQMAYFLQRAGRDYMVFERAPGPGS.[F]	3213.57207
5973	[P].PAPPGFSGPVSSPQINSTVSLPGGGSGPPEDVKP.[P]	3213.60625
5974	[T].PVYSQVTPRAWRHQAPSEDARGAQPGRVP.[V]	3213.64128
5975	[T].GAPGQKGERGVPGGRGPMGSPGLQGFPGITPPSN.[I]	3214.61744
5976	[S].FSPSATPPQKYSSRSNRGEVVTSFGSAQGVS.[W]	3215.57159
5977	[H].RIHSSSEIQNNISLNGKMDNTTFGKLSSH.[L]	3215.5862
5978	[G].PAPRNSSTRPCLPEIHRSSAPGALELLCE.[V]	3215.60482
5979	[L].LDSAQINLYQDVILENFRNLVSVEMLY.[F]	3215.62929
5980	[N].PNLQASLSGPQPQLQGSHSHPSRLPSSSLAH.[H]	3215.63045
5981	[G].GPPSNADLLSCLLGAPDPAPEGAAGGLLFSSPAP.[T]	3216.58815

5982	[E].VSGAGSSPVSGGVNLFANDGSFLELFKRKME.[E]	3216.59939
5983	[L].GQQVKDGLIVGGQGDASVDAIYKAVVDAAGKGM.[Q]	3217.65215
5984	[P].EKTQSQVTSGFFTFSHPVSSGPGGLAPFSFS.[Q]	3218.54292
5985	[F].DASVSVPSEGLPQGTSSAPQAPAHPTGASESIVS.[Q]	3218.54477
5986	[R].PGAQTPTAVYQANQHIMMVNHLPMPYPVP.[Q]	3218.55839
5987	[R].HYSPPPFSLLGTTPAYSFGIAGAGSGVPFHW.[H]	3218.57343
5988	[D].RLPSGGEGPEVSASGVEDISGLPSGGEVHLEIS.[A]	3218.58115
5989	[E].PGAAGIPGEPGSPGKDGIPGVRGDKGDVGFMGPR.[G]	3218.60112
5990	[L].EEVPEPLASSQGQSLPGSSREHMAQWEVR.[N]	3221.52801
5991	[P].VAAGGGSGEGRKRCPSQSSSRPTTSQPPTPPAG.[Q]	3222.56686
5992	[Q].GARPGPGWADSPKADKEKGSSWRNWPGEAK.[A]	3222.58277
5993	[P].PGPQGNAGPQGHLGPQGPPGPQGHIGPQGPPGPQG.[H]	3224.57326
5994	[L].GPQGPPGPQGNAGPQGHLGPQGPPGPQGHIGPQGP.[P]	3224.57326
5995	[L].QTKNPEGRFPDLTSELNRWHTTFGHEK.[A]	3225.58243
5996	[E].KPGASVASVHSEAGPKGAEKPAAATGKGWPEAKG.[Q]	3228.676
5997	[K].GASAPLVPIEELENPELIGKGGFGSVFRAHH.[R]	3228.68002
5998	[P].AVSAAGVPPMPVICQMVPLPANNPVVTTVVPS.[T]	3228.68292
5999	[H].PGPSSLGTPTSLLPCSSGFQTPSSPPAHDPGVP.[V]	3229.54702
6000	[A].AAAASAPAPGPASSPEASPAPGFPFPPPWMGMPL.[P]	3229.54854
6001	[L].KRKGYHCTAQKEVGPGPGGGGSGINPAYRTE.[D]	3229.59195
6002	[S].VPNSSELSPAISTILMSGYPFLGALCFAITG.[T]	3229.61595
6003	[A].LYGGHTPAGMQTKYMDVGWGLGSVTHELAPG.[I]	3230.53976
6004	[D].LNSSHTSPGHAMRGTLAGTEGHTKGLEMALH.[G]	3230.54295
6005	[E].PGDVSAGPRSGGGRNATTAMPPPVPNGNLHPHD.[P]	3230.55081
6006	[G].SSPLNQASAELAKTSFGSSPHLTGPTGHRHSA.[P]	3230.59373
6007	[H].GPPIQCVPWGLAGGRAGPQPGQGVSMAASSRAP.[V]	3230.59459
6008	[E].PAGETQPGASAAGLSSLAHGVPHPPGTGRDRGEP.[E]	3231.58897
6009	[Q].ESISSAISALDDPPLAGPKDASTPDGPPLAAEAA.[V]	3231.59032
6010	[P].GAPGPKGDPGFQGMPGIGGSPGITGAKGDMGLPGVP.[G]	3231.59253
6011	[T].VSSAPHSPSPSAPLTVSQTASLSTSPPSPATSTP.[I]	3231.60156
6012	[A].PGEYFFSDGIRLKKYRGMGSLDAMDKHL.[S]	3231.60778
6013	[D].MGLGIEGAKPPHAYGAKKAKNGGGPAYEMPAF.[T]	3231.60778
6014	[D].SGPPTRESASRQEDLKTEAPVSPGAAPWRPG.[L]	3231.61413
6015	[Q].NPVSASPVPSGTNSPAPKKSTGSVDYLALDFQ.[P]	3231.61681

6016	[G].KPDTGTPRSHLPTAGKMTGDAAAAVVNGKHTE.[E]	3231.6175
6017	[L].VLPSANSPSHTPTLNMPATLVPLDADAKSVGD.[G]	3231.62018
6018	[P].RPREGGEGGGSRRSRSAPAQGGSAPAPPPPPTH.[T]	3231.62267
6019	[G].APAAATEAALAAPAAEAASAAPDPPAAGAAPAAPAAPAA.[P]	3231.62805
6020	[M].ATPENLASLMKGMAGSPSRGGISWQSSIVHY.[M]	3232.58778
6021	[P].LMAGASPVHFAAAGTVEPKAGSSKNAPNPPASAE.[I]	3232.60554
6022	[G].PQGHLGPQGPPGPQGHIGPQGPPGPQGHLGPQGP.[P]	3232.61474
6023	[T].PADHLGVSLTPADSLGASLTPADSLGVSLTPADS.[L]	3232.62196
6024	[V].PQQKAMAPIHAHPAGMRINVVNNHQAKQN.[L]	3232.63271
6025	[Q].PQGGGLARGAEAESQPPRPPKHGLAVSPAPVGM.[P]	3232.66439
6026	[L].PGSLGFGSSNFQSVGQVFPSLGFGTGGFQSVSP.[N]	3233.55382
6027	[C].DKVNNIHPAVHTKTDNSVASSPSSAISTATPS.[P]	3233.60329
6028	[S].AGGQLNSSGPSASQLQQLQMQLQLERQHAQ.[A]	3233.608
6029	[P].DLLHHANPGSISHLSYRQSSIGLYTQNQP.[L]	3233.60865
6030	[K].PGALGPQGQPGLPGPPGPPGPPGPPAVMPPTPPPH.[G]	3233.65645
6031	[A].PGGAAPGGPGFRAFLCPLICHNGGVCVKPDR.[C]	3234.58701
6032	[P].GPPGTPFATAISKDSMVIQWHEPINNGGSPI.[I]	3234.58882
6033	[Y].SIGDSTKTIEPMEGLKSEFKPSSSRSEAHV.[F]	3234.5947
6034	[S].VRSSGMTPGTPRSPAPSTRSVTSVSSRERGF.[E]	3234.63963
6035	[D].GRGESATVAEGAEGQARGGPAQPRGPETGESQAA.[E]	3236.5275
6036	[Q].VFESGGSKKCIQVGGEFYTPSKFEDPTGGK.[N]	3236.55685
6037	[Q].GPPGPRGMQGPPHPHGIQGGPGSQGIQGPVSQGP.[L]	3236.57664
6038	[T].EGPGVSISEERQSLAENSATTVVYNPYAALS.[I]	3239.57026
6039	[R].DRRAGGGGGGGVQNGPPASPTMAHEATPLPTGRP.[R]	3239.57228
6040	[L].TYMVKQFAVSSASEKLFSSKSNAQFKMY.[K]	3239.57515
6041	[D].VGRCWSPGAAVAPEMCPIRATGKSPSICPQ.[S]	3240.55332
6042	[T].TGRKYYCLLPAGGVCGPGVQAWSGSLWASAA.[L]	3240.57173
6043	[G].PGKSGSMGPAGPPGPAGERGHPGSPGPAGSPGLPGVP.[G]	3241.58072
6044	[S].TLGQPSTNTMGLFGVTQASQPGGLFGTATNTST.[G]	3242.5634
6045	[P].AVMQPPPGMSLPPADIGPPPYEPPGHPTPQPG.[F]	3242.56492
6046	[S].AGDVDANKQSLPYPQPGLESAGIESPTSSVLD.[K]	3242.56992
6047	[A].DVGGAAAAPGGGAGGSRELEMHTISSKVFGDILD.[F]	3242.57463
6048	[P].HSTPRPVSDGGKMVNAAVNTYGSAPSGSRSRT.[P]	3244.58759
6049	[T].PPSAASASASAKAAEAGPPTDRGVQVTEHGATAAL.[V]	3244.61927

6050	[K].AGPRAQPQQGIHHWTLYSSFPKLGDSLRP.[M]	3244.67627
6051	[G].PGSHRMEMLNRLPFPPGAPEWQGGSQGAPGA.[M]	3245.53675
6052	[R].LNGTPGEPSAWGATAGRAAKSMSAEDLLERSD.[V]	3245.54914
6053	[L].PGSPGAKGEQGPAGHPGEAGLPGPSGNMGPQGPKGI.[P]	3245.57563
6054	[T].AGRPGTGAPAEGAGEGALRRGVLHARCVSSVKT.[I]	3245.70323
6055	[L].PGRGEVWGAGYRSHREPGPGAKEEAAGVSGPAG.[G]	3246.57874
6056	[G].DPPKSIELDGTFVGAEVPGEVGGLGPGPAEARE.[L]	3247.61173
6057	[I].SVLESAPAGMLLIQLNASDPDLGPSGNVTFSF.[S]	3247.61912
6058	[Q].NLIMASLPGVMAIGPGEPTSLGPTFTNTGASTL.[V]	3247.62249
6059	[V].EGRTFPVDIFYLQSPVPDYIKSTVETVM.[K]	3247.62314
6060	[G].PQGYPGIGKPGMPGMPGKPGAMGMPGAKGEIGPK.[G]	3247.6247
6061	[G].EGLSPLPQLTESSSFLSSVTSVSRDSPVGNLG.[K]	3247.63286
6062	[V].VFAEHVGTGQAGGHGGVALGAQSYYGKSLPFGE.[R]	3248.57595
6063	[Y].FAITMERSFQGPVLIGSSHGGVNIEDVAAET.[P]	3248.58922
6064	[E].QRAEYNITITGHFPGHLVDVSGTGTLSQSY.[Q]	3248.59708
6065	[G].SPGSAGPRATWRWQPAHQMGRPGLPSPQTH.[L]	3248.60313
6066	[S].PTDGVTLSPGGPVETPSLEAASGDLTTPPSTLSP.[P]	3248.60564
6067	[T].NDLFSQALQHALQASGQPSLQSQWQPQLQ.[Q]	3248.60831
6068	[Q].AGRPGNPGHQGLAGVPGMPGTKGGPGDKGEPGRQG.[F]	3248.609
6069	[E].AAAAPTAAPGPAQPGHVSPTPATTSPGEKGEAGTPV.[A]	3248.61821
6070	[F].GGRGATDRHFAGIGGGFRDLVPETPAPENSPI.[Y]	3248.61955
6071	[K].GPAPRETKDTDIVDEAIYYFKANVFFKN.[Y]	3248.62625
6072	[P].PGDIGLDGNPGAPGPRGPKGERGLPGVQGSPGDIG.[P]	3248.64068
6073	[A].PRGGGMGEDEEDAEGVAEPPEASAPEVPMEPL.[E]	3249.41983
6074	[L].QGLTSPEPPSITSSEPPTMTPAEPSITASLEP.[T]	3249.57189
6075	[G].VAEASQPDKPSAPAAAAAAAQPPASHGPERSQSP.[A]	3249.58831
6076	[C].PGPLADSSVTLSPVDSLDSPRAFGGPPASPGGFP.[L]	3249.60625
6077	[L].RANLNGSNVEEVVSTGLESPGGLAVDWVHDK.[L]	3249.61346
6078	[A].QISRHSNPTQGAAPAWTPSTRPGFSAQQVAT.[Q]	3249.6148
6079	[H].PGGREPGLQCGSPAPPRGRSPGPRGAGRGGEAGP.[G]	3249.61548
6080	[R].AASLANTVRSFHHEGEARGRSLGAETGAGSRP.[A]	3249.62201
6081	[D].QNQFISSEPTALHSSEPQHSLINSTVENR.[V]	3250.57232
6082	[Q].QPSASQAGVQQPPATSTGGPAASAVSASVSTQVEP.[E]	3250.58222
6083	[R].SGGVGGLSPPCITTVSVNESLLTPLNLEIDPN.[A]	3250.65115

6084	[E].AAQGAGAVPTAVTKVTEHPAGADPGAAPMIPAVGPG.[L]	3250.65249
6085	[A].RSHVSSNGGGGGSSEHPLEMPICAFQLPDLT.[V]	3253.50008
6086	[P].AGPPTTDFWAQNSPHHKLPNNGDNPWGNLA.[E]	3253.51983
6087	[L].AQTIYPSEGGVTEQGASGHRGQGRGPHMLSTA.[V]	3253.54031
6088	[R].ARGFQVHERQFRGGAGLGAGSGASDYDLCIT.[C]	3253.55557
6089	[L].GPAGNAASTAGPFPFHLSQHMLASQGIPMPTF.[G]	3253.55575
6090	[M].QPLHFLDPLPLSQQPGDSLGEVNDPYTFE.[D]	3253.5688
6091	[D].GPEPPSPAPEPAPSRAQAAEGPHLTPEASPDPV.[P]	3253.57601
6092	[P].LGDGGVSLDTGPTGWQESRGERHAVEKEGTK.[P]	3253.58322
6093	[N].SRGHGPAQAGHHHPEKSQPLCEPAPLSGASAS.[P]	3255.54606
6094	[W].QPSRADGPPATPTQPSGGRSLGEDGPPARGASSP.[T]	3255.57372
6095	[V].RGEFMTSRVNWVVQSSAVDYLHLMLVAM.[K]	3255.61116
6096	[L].GPQGPPGPQGHIGPQGPPGPQGHLGPQGPPGTPGM.[Q]	3256.57049
6097	[I].PSAVVPGSMAGRMTTTVAPGSIAGGMAPSLPPGSM.[I]	3256.58329
6098	[P].GVGAAGGSLSFGASSTPAQGFVGVGPFGSAAPSFSIG.[A]	3256.59093
6099	[L].QPASVTQTAGSHSAPGHPATAANSATTQVLIGNN.[I]	3256.59412
6100	[N].PPMPGALGASGSGGHELSALGGEGGLQSLLGNMSH.[S]	3259.54703
6101	[Q].PLEWAQGPGQLTSDPPGLTEPWSSLSSDLPP.[K]	3259.57936
6102	[I].GHRGAPMLAPENTLMSLRKTAECGAVVFET.[D]	3259.60205
6103	[R].SGAPPPSGSAVSTAPQPKPADKMSKNKKKKLK.[K]	3260.81474
6104	[L].PGSPGAKGEQGPAGHPGEAGLPGPSGNMGPQGPKGI.[P]	3261.57055
6105	[H].FGQAPNKGTSSDGVSLSNLAQLSLTAADQQQQ.[E]	3261.5982
6106	[K].ARDGDTKSIPEAQRSPSLESGVGPAPPEPGLM.[E]	3262.60084
6107	[Y].SPTPVTSGIGIGMSAMGSATRYHTYLPPPYPG.[S]	3263.58638
6108	[D].NAPSPSIGGSSRLDSTTPTQPMTPLHVVTQNG.[A]	3263.59609
6109	[K].KGNSPGSEPPPPKTAWAETSRPPETEPGPPAP.[K]	3263.59675
6110	[L].SRPSMPVASGAALPSASPSGSLSPPPPPPGLYFS.[P]	3263.64052
6111	[A].APGGGSVAAASAAMGAALASMAGLMTYGRRQFEH.[L]	3265.56633
6112	[P].GPMGPPGLPGPMGIPGSPGHMGPPGPTGPKGTSGHP.[G]	3265.57035
6113	[P].GPGADLETNWAQNPGRGKVTPGPDLIYQNLS.[G]	3265.62363
6114	[R].AAPGEAPRERHTSTGNIQVGLPEPASVSNHVS.[A]	3265.63084
6115	[G].AGRSLYGGPELVFPEAMNHHALTVHPAHQL.[G]	3265.63236
6116	[P].GPLADSSVTLSPVDSLDSPRAFGGPPASPGGFPL.[E]	3265.63755
6117	[Y].SVDARGFLDQPGRVDEQRRPLGEPGVGDSR.[P]	3265.64207

6118	[Q].AAGVAADWAAAGLADGARAAGHAGHGPAHGGLAGHGAA.[A]	3268.58556
6119	[R].HTVSTFNVTAGEYDLRYVEPGEQTLTIET.[I]	3270.58009
6120	[G].VDPAVSHSPFEPHHRGGTPGEVYRSHLPAH.[L]	3270.594
6121	[A].PGASGHGGTVGGVKAAAEPVEAGEIPGPIASCQEL.[K]	3270.60593
6122	[G].VGAAGGSLSFGASSTPAQGFVGVGPFGSAAPSFSIGA.[G]	3270.60658
6123	[V].EAGPAPAPHAPGGPPSNADLLSCLLGAPDPAPEGA.[A]	3271.56882
6124	[G].PPGGSRKCPPGSPTDPNATLSKDEAAVHQDGK.[P]	3271.57603
6125	[V].YPLMGVSGKDDVFAGAWIAIFCGFSFFVVA.[S]	3271.59951
6126	[L].SGPPGPTGRSFTVHTHRENPAEPGAVTGSATVT.[T]	3272.60429
6127	[G].TDPPRPAAEAAAAAGGGGATAAAARGGEAAAEVTGWP.[A]	3274.58355
6128	[T].AQLAGMQISGAAAPAPAPSGLGYGPPTSLASASGSF.[P]	3274.60487
6129	[S].DGAISVPSLSAPGQGTSSTNAVGGAVSSQAAPAPPPA.[V]	3274.6186
6130	[A].SPSSRPVASPAVTSMPPTSSGVREASLTSAMTK.[V]	3274.6406
6131	[Y].MEGVNPFIKSNKHRMIMFLDELGNVPEL.[P]	3274.64212
6132	[P].GQPGRQGVAGRDASDQHIEDVVLKMLQEQL.[A]	3274.6597
6133	[Q].GPPGPRGHQGERGLPGLSGSGSSSLGLNLQGPPGP.[P]	3274.66756
6134	[P].SIVICFLVAALSSMLAGLCYAEFGARVPRS.[G]	3274.67851
6135	[G].LNFPWMESSRRFVKDRFTAAAALTPFTV.[T]	3274.683
6136	[P].WKSVRLDVTLSPGDVAGIGWERTEGTPPPPG.[Q]	3274.6855
6137	[F].KTQPAEAIIWQMGTGMKPNLSQIGMGKEIS.[D]	3276.64252
6138	[V].AASVQEGLPGSLGRAMGGQQEAPPAPEGRTPFI.[G]	3276.64298
6139	[C].AIVVNAAGAWSGQIAELAGVGNGPPGTMQGTRLP.[V]	3276.67937
6140	[K].PAASVDPWGAPTGAGTHSAPKGSDPWAAPQQPAP.[S]	3277.56611
6141	[A].KPVCVCMRDLYALFKAPQVPSPGAPGQGPH.[P]	3277.64312
6142	[L].ANPRAFYRPGARGDHYARNLLWFDFGLP.[F]	3277.65548
6143	[Q].GPASPAGGNQSPRARAGARPQSAPEFKGSLASLS.[D]	3277.67846
6144	[A].KPMAKMQVEVEKQNRKKSPVADLLPHMP.[H]	3277.72177
6145	[H].ASEEDVYLATTQQLVEGVVSGYNATVFAYGP.[S]	3278.57394
6146	[V].VQTALSETQDPEEVSVTVKAFMTADLPNEL.[I]	3278.59844
6147	[F].RAPGAARGCSLDGLAGGGVDEGSLLCAGGPAAPLGA.[Q]	3278.60047
6148	[E].RGEKGEPGERGPPGFPAYLDEELQGTLHEI.[R]	3278.60764
6149	[G].LAGQDGVSTSLSLSSHGGGSPPAPSQAAIDTQAGAS.[P]	3279.57238
6150	[A].PLPASLDTGDHLFGSMSVGNSVGNLPAAMTHLG.[I]	3279.57727
6151	[E].DLPEGNKTISENASATAAPKMPESAPVSAPVPS.[H]	3279.60493

6152	[V].PGERGPAGPPGPQGPPGEQGPEGIGKPGAPGTPGQP.[G]	3279.61413
6153	[A].QGSLWSVEGGNKLVCSGLLKLTKANVIHATV.[T]	3279.78819
6154	[I].QSTISIDSNVSPQGSSSRVATTPGLNPMTPVH.[K]	3281.60666
6155	[G].PKGDPGFQGMPGIGGSPGITGAKGDMGLPGVPGFQ.[G]	3281.60818
6156	[A].NYEVVVTARDGGSPSLSTMASVSVEVADVNDN.[A]	3282.54305
6157	[M].MHPPPSAPPGPEAALMPDPGPGEVPAAADDAPLP.[A]	3282.54458
6158	[G].PGGAGGGGSAGGARTALSDAELGRWAELLSPLDES.[A]	3282.59854
6159	[Q].QRARPSTTSSGPSQGPSGSTPRPSTTSSQGPALG.[Q]	3282.60575
6160	[V].RSPEPGSTGPASPAVSTAAASPVLPTSPTSSPVGPA.[P]	3285.65974
6161	[Q].QPQERHPPPHLAALSPPGVEGPASTQASLATSG.[S]	3285.66108
6162	[L].NGEEKAEASKGVPAPPHSSSPAQGRAERQENA.[P]	3286.57953
6163	[K].KSEEKHQEQKNSSINTVKHDSKKTTNTC.[F]	3286.60805
6164	[W].TFPNARAASGSSDPFLCPPRQLEGLPRTPM.[R]	3286.60958
6165	[H].HQLPGPGTTLSPMGTNAVTSHLNQSPASLSTQG.[Y]	3286.61208
6166	[R].VYPERWSPAGIGMRLEVLGCDWTGLAHTV.[S]	3286.6136
6167	[R].PGAPPSASSAASRAPPESSAHRLSGGPSTSRPSSS.[T]	3288.59518
6168	[E].PGGAGGSPDGAGGSKLPPVLANLMGSMGAGKSPQGPGG.[G]	3288.60997
6169	[T].SGPNQALPGTTSQQTVPGHHVTPGHFLPSQNP.[P]	3288.61446
6170	[E].GPAGFPGPPGIQGNPGPVGDPGERGPPGRAGLPGSD.[G]	3288.61446
6171	[A].PAGSGAPPGSLGPSEQLGQAGPTVGPQQQPPAGAPQ.[P]	3288.62436
6172	[P].PGGTSPPNGGLPGPLASTSAPPGPPAAASPCLGPAAAA.[G]	3288.63175
6173	[I].GVPGPAGPKGERGSKGDPGMTGPTGAAGLPGLHGPPG.[D]	3288.65422
6174	[G].DQGAPGAVGPAGPRGPAGPSGPAGKDGRTGQPGAVGPA.[G]	3289.64207
6175	[P].GIMAPPPGMRPPMGPPIGLPPTRGTPIGMPPPG.[M]	3289.67165
6176	[A].GVTPAEPEELPTPMAQALPSPASTATPPPTPTH.[L]	3290.62493
6177	[L].PASVSVEFAVAATDCIAKDVVDPTKCNLLAE.[K]	3290.62831
6178	[P].PAGPTSPSGAHPGEKPLVDLPGEAPTGPTDAAGKN.[M]	3290.62877
6179	[L].EERDWLPGKTLFENLWASVYSSRKTMF.[V]	3290.63029
6180	[F].PQDLLPPAREEPLMGGPRSPALVSSLSPESM.[S]	3290.63954
6181	[A].RGAEGGSTAPAPAPAASPPPEGPVLTFQSEKMKG.[M]	3290.6474
6182	[Q].GPGEEETRFLSPRPAEGAALHPTPHPPTGTAL.[Q]	3290.65526
6183	[P].GPGASRPPLGNVEGVGCKASPGRFVVGPARSQE.[Q]	3290.6811
6184	[V].KLHSAPASPSSASKEVGIGFAQGPGASASTAATPGP.[A]	3291.66041
6185	[A].LSENAPGGKPGINQTYRSPLGSTTSAPAPSAPPA.[P]	3291.66041

6186	[A].SGSGAPVGGSISSGSSASSVTVTRSYRSVGGSGGGSF.[G]	3293.56288
6187	[Q].QVYGEKRDNMVIPVPEAESNIAYYESIY.[P]	3293.56708
6188	[A].AEGVGAAANAAATSSTGTGGVAASGMAASGVVPGGGFVAS.[A]	3293.57027
6189	[S].ASQAGVQQPPATSTGGPAASAVSASVSTQVEPEEP.[E]	3293.5768
6190	[T].PLDMNRFQSKAFRTVMSQHSGQASVSPTPG.[Q]	3293.579
6191	[L].QAAGSGIQNQNGHPTLPSNSVTQGAALNHLSSH.[T]	3293.6006
6192	[L].KNAQDLDVGNNNIQNYSISPNSYFHVSTR.[N]	3295.57266
6193	[D].KNKSIVWDEKKNRWVDVNEPEEEKKAP.[P]	3295.70696
6194	[L].PGANTPLPGLSHRQGWPRPLTPPAAGGLQNHT.[V]	3295.71954
6195	[E].KDAWDVKMLLEQFSFDIAEEASKVCLAH.[L]	3296.59661
6196	[V].LGGSASFGPSPGVEPVASMTSVASHPALGASSSSLP.[P]	3296.61035
6197	[E].SPQSLGATNSSPTPVGRGAQVGPGQGPVPQDGGAAP.[S]	3296.62542
6198	[L].PGRDGMTGAPGLTGERGEKGEPGERGPPGFPAY.[L]	3297.57055
6199	[K].PGLPGMPGSDGPPGHPGKEGPPGTKGNQGPSGPQGP.[L]	3297.57055
6200	[S].QRQFPGQAYGSPGASGVGTSPGPRDTELAASGPP.[T]	3297.58831
6201	[E].VWGAGYRSHREPGPGAKEEAAGVSGPAGGRGGGY.[G]	3297.58964
6202	[G].TTHTSLGPAPSAHDALREAGHAGVVPCHWPAP.[A]	3297.59704
6203	[S].ITPNTWSANSPLGNPFQGNVFPAPAVSTQTPS.[M]	3297.61748
6204	[T].ATPGAAGGATAASAAASVLGGSAAPATAGDTTKSENVAP.[A]	3297.61933
6205	[G].PGLSSTSPVGEPSAGLGPGPEDVPPFPPMLLNAP.[G]	3297.63477
6206	[T].SNKELATSNGNGRNDSKGALTQTLEMRENF.[Q]	3298.57167
6207	[P].GPSADVLSQDPRGSAASVAHQEPPSSVLDMVHG.[E]	3298.57569
6208	[Q].GQVPSTTATTPGNSGAPQLQANQNVQHAGGQGAGP.[P]	3298.57953
6209	[P].LSPSPQQSQQGLSPSHVAGSSSQGQALQQPPQG.[S]	3298.60468
6210	[P].ASASPAPNATADGSKTSRASVDTTPSVIQHRAM.[M]	3298.60806
6211	[T].PGKLQDSSVGGQGAQGSQPKPAAAGGPHTLNLSEG.[C]	3298.64107
6212	[R].QEVNPVRQGNGSEIVSEEKIQEQKSFEPL.[Q]	3298.65499
6213	[-].AGTTHTSLGPAPSAHDALREAGHAGVVPCHWP.[A]	3299.5763
6214	[S].TPAGPPSGGASPTPPAASPSGGSATRPSSGPTSEAPR.[P]	3299.5887
6215	[R].AQLEQGGPGMRRGRSSGTGVGGGVEATGPILMSP.[H]	3299.62193
6216	[L].NGIVDPAVMGGFAKYEKHAATNGRGGEGRVQP.[F]	3299.63382
6217	[A].GPGSPATLSPSAGVPQPVGMEALDQAEGPAASQRA.[M]	3301.61174
6218	[P].PNHKAPLTMASPAMLASVESGGPPPPTASQSASV.[S]	3301.61914
6219	[H].VDQLQAFNVPQAGGWGPWGPWGDCSRSCGGG.[V]	3303.44832

6220	[C].PDCVLEKQVFVNGERFSHPQDPCQECQ.[C]	3303.46159
6221	[A].GPATQPTGPLPQPACPPPAPAAGPAAPQTTTASDL.[L]	3303.63142
6222	[S].GAGPGGGLTPTAPPYGAGKHAPPQAFPPFPEGHPA.[V]	3303.63341
6223	[S].GPGVEPHGVLREVTTEFTVDARSLTATGGNHV.[T]	3303.67164
6224	[H].HATQLHAHQPQPATTPTGSQPPSQHAAPSPVQ.[H]	3304.62061
6225	[A].WPGLAEGRRRAGAMAEAGPQAPPPPGTPSRHE.[K]	3304.65047
6226	[P].GPEGAPQIPGPHASSVTHFPPPSSLHQRPGHE.[Q]	3305.61988
6227	[A].KSPSRQEAHEALSPGEAAGGQAEARREFLEP.[V]	3305.62575
6228	[V].GEPGVAGPTGPPGVPGSPGLTGPPGPPGPPGPPGAPGAF.[D]	3305.65895
6229	[R].AGRAPGGQHVPAPAAPGSSSGTGVPKWPVAPAGAGTS.[P]	3305.6774
6230	[H].PGGREPGLQCGSPAPPRGRSPGPRGAGRGGEAGPG.[P]	3306.63694
6231	[N].QPQTQLQPQVPGQQPAQPQTHQQMRSLNP.[L]	3306.63963
6232	[A].RGAEGGSTAPAPAPAASPPPEGPVLTFQSEKMKG.[M]	3306.64232
6233	[H].PGARAGGASSPSPVVFTVGSPPSGTTPPQGPRTTM.[F]	3306.65355
6234	[R].GEPGPAGAVGPAGAVGPRGPSGPQGIRGDKGEPGDKG.[P]	3306.65739
6235	[P].PGSTASLSTASLTPSSPRVPNVSAQGPTVQAPMP.[T]	3306.66344
6236	[K].PGPTGPAGQKGEPGSDGIPGSVGEKGEPGLPGRGLP.[G]	3306.67131
6237	[A].AGAGPGPGPGAPPGLEAALQKLALRRKKVLSAEE.[M]	3306.86447
6238	[A].NPSKQNQSAPAAAPFTSSSAANGLESSVATDSSK.[L]	3307.5673
6239	[P].AAQETRASPSSHGQGPKENRSGPAHGPMALSPP.[S]	3307.59849
6240	[A].PPEPAAHAAAPTSLLPCRGGEGAGAAATAGVQEPGA.[P]	3307.61241
6241	[H].TQSPGNLHAASSPSGALRAPSPASFVPTPPPSSH.[G]	3307.64543
6242	[M].ASKSTAFQNPMGIVPSSPKNAGYKNSLERNN.[I]	3307.6488
6243	[S].NRAFGAVWNTSSKGTISVASNGNPKADLNMTV.[T]	3307.6488
6244	[Q].PGGAKDSVNGTLARSSLEDTYGAGDGLKRGALSS.[S]	3307.6513
6245	[P].PAGDGEGAVLSPSQKPHGAPGAEPGSSGSHGSVGSLA.[V]	3309.57305
6246	[L].GPAGNAASTAGPFPFHLSQHMLASQGIPMPTFG.[G]	3310.57721
6247	[G].WGSLQELSSPRQPVSPPENSRAGPREFSKS.[Q]	3310.65633
6248	[V].VPSAGASAAQLSSSPLETPPSVHYESIKFEPE.[N]	3312.62704
6249	[K].GQVCVMIHSGSRGLGHQVATDALVAMEKAMK.[R]	3313.62722
6250	[N].FFHFVLALYDRQGQPVEIERTAFVGFVE.[K]	3314.69969
6251	[P].PAAAPPSAVGSPAAAPRQPGLMAQMATTAAGVAVGSA.[V]	3316.67766
6252	[Q].PNITPSSSPSPVPAATNQVPTAMSSSSTPQPQGP.[P]	3317.59542
6253	[G].SPGRCRTPAGEGPHTRVSRVFCFLDLMKA.[R]	3318.6405

6254	[L].SLQQAFSELRHARMAEGPSTAPPHFGQTGPV.[F]	3320.62292
6255	[A].PKDEWAAAYGPGPTAPTASPAPLAFGPPPDFGAV.[P]	3320.62625
6256	[R].TLDPSPAGWFVQQHRELELMSSFRERFG.[C]	3320.62694
6257	[T].AASPESPPTPQTSLTPPQASPAASKDQSPPPSPP.[P]	3320.6281
6258	[R].GEQISGTVGWWGPAPVFVTSLEACVTQSFLP.[G]	3320.62963
6259	[S].PGQGLTARREHVYGMFRGGDRSGSLSSTAGGR.[S]	3320.64136
6260	[P].GARVIRGLDWKWRDQDGSPQGEGTVTGELH.[N]	3320.65191
6261	[A].AGASGGASPVAFTPRGGPSPPGHSPGPPRTFPSAPP.[R]	3320.65593
6262	[L].FNCGGGRRLALSSSTGRGVSALGPCAATGGGRRG.[G]	3320.65597
6263	[S].LGLTSTNTNFKGPLICNMNYVVKVEVGAPPS.[A]	3320.70174
6264	[T].GTGGVAASGMAASGVVPGGGFVASAAAEVQTGRNNFV.[I]	3321.62806
6265	[P].VHAAYNIEPNKSTKNEMSPALLQASLEQHT.[M]	3321.65321
6266	[L].ASPVSVYLFPGERSGAHPPSPAAGAPTGSLGSRSG.[A]	3321.66108
6267	[H].PPQAAGTPHLVYSQAPPPPMTSAPPPITPPPGH.[I]	3321.67249
6268	[G].AFPPPATPSAPGGQARPRPNGAPWPPEPAPAPAP.[E]	3321.69159
6269	[S].VFQGTNGTSVITPLDPSAQLRIMPLPAGGPSIS.[Q]	3321.75114
6270	[L].EGALSTMVQLYSRTQEASPQVLEAFQNFY.[P]	3323.58888
6271	[R].ERTATTVTDSRGAGGGGSGALPAGTANSGTARHWP.[P]	3324.60642
6272	[P].QPATTPTGSQPPSQHAAPSPVQHQAGQAPHLGSG.[Q]	3324.61044
6273	[M].GPLGTGFHGNTVSSPQSSAATTPGSPSLGRHPGAH.[Q]	3324.61044
6274	[Q].PGEPGARGEPGDPGLPGRPGTSIGDEDEKRGLPG.[E]	3324.62033
6275	[G].PGGAGTPPRTGAGLPLPTHGGGFGSGCGRPAPPAASS.[P]	3324.62907
6276	[P].LANSQYATIKEEKGQCYLYMKVIERAAF.[P]	3324.67553
6277	[I].KGSGFAVASAGAQVLMGHFPCKVLSVNYTAIE.[C]	3325.67078
6278	[L].GPPAASTPAGPPSGGASPTPPAASPSGGSATRPSSGPTS.[E]	3326.58837
6279	[I].EMATGRPPFHELGSPQAAMFQVGMYKVHPP.[M]	3326.59075
6280	[G].GPALGTRGKTKGDRGKQDPGDPELPSSTVQALP.[V]	3330.74006
6281	[S].GSPEKVQTHSPQLLTAMISGLDDGDDPHSLVA.[L]	3331.61107
6282	[P].GAVGSGPGLDSAPRTARTAPASGSAPRESRTAPEP.[T]	3331.67377
6283	[P].GPPGTPFATAISKDSMVIQWHEPINNGGSPII.[G]	3331.67797
6284	[F].CATVPKDGRSYSPTLFAQTVRVLKKINKPG.[N]	3331.83073
6285	[T].VSGYFLAGRSMTWVAIGASLFVSNIGSEHFI.[G]	3332.67724
6286	[A].TDGGIPPLSTETHFTLQVADINDNPPTFSHI.[S]	3334.62263
6287	[P].TAPQHFSGRLSSSPLSPGGPTHLTKPTATSSTE.[R]	3334.66622

6288	[S].RSAAVTSEFHLVPSRSMNGQPLTCVVSHPGL.[L]	3334.67832
6289	[K].GARSVEGPAGLEPGLEEYRRGPPGTPAFLQEP.[P]	3334.68148
6290	[G].PQPQLQGSHSHPSRLPSSSLAHHALPATSLVP.[G]	3334.74033
6291	[R].PGKTPNTTPYVNLPHQASAPAWWPDPSRHI.[F]	3335.67085
6292	[L].LEELNAEAGHLDPGFLASEKTSAGNAPLNEEI.[N]	3336.62302
6293	[G].RMSAPRNYSRSGGFREGRTGFRPVEAGGQH.[A]	3336.62638
6294	[P].GINQTYRSPLGSTTSAPAPSAPPAPPAFHGMLE.[R]	3336.63175
6295	[P].QVPGQQPAQPQTHQQMRSLNPLGNNPMNIP.[A]	3336.63244
6296	[D].GGVALAGERAEAASVGALAGGTALGAHVVAEDGAQHP.[A]	3337.68835
6297	[P].PPNLASTTMNLTSPLLQCNMSATNIGIPHTQ.[R]	3338.61776
6298	[T].PGTPASLSANSSLSSSGELVEPSVDQTPQASPGLA.[P]	3338.62341
6299	[P].PGLSSSGVSAASQGAGGGPPPAPPLPTAQGPSGGGTGAPS.[L]	3338.62475
6300	[A].PGTSAGLGQGQGQLVGMYQGADTFMQQLLNRV.[D]	3338.62562
6301	[R].GSPPIQYQFYHEDVALGNSSSLSGGGTSFHLS.[L]	3339.55527
6302	[P].NIPPHGVPMGPGLMSHNPIMGHGSQEPPMVPQ.[G]	3340.58462
6303	[A].VSQPSSPHSPPSLGRSSEVSPVPTPSRGGADGGGGG.[G]	3341.6105
6304	[S].KGLPSPYNMSSAPGSRSGSRSGSRSGSRSGSRSG.[S]	3341.61118
6305	[Q].THQQMRSLNPLGNNPMNIPAGGITTDQQPPN.[L]	3341.61137
6306	[I].KAEADKIYSFTDNAPSPSIGGSSRLDSTTPTQ.[P]	3341.61318
6307	[L].AGAWTHKMGTAHVSVLGEDGSAVAATSTINTPFG.[A]	3341.62191
6308	[V].EREASQLHRQAGNSSGKEVLMPSHDPPPASL.[E]	3341.62912
6309	[Q].KYSSRSNRGEVVTSFGSAQGVSWSGRGGTSHL.[S]	3341.63699
6310	[P].HSTPRPVSDGGKMVNAAVNTYGSAPSGSRSRTP.[T]	3341.64036
6311	[D].KEPNPASMRLLGAFPSPSGPASPCSLVNETTL.[I]	3341.65044
6312	[Q].KGDPGTPGYPGKNGPMGTPGIPGTPGTMGPPGEPGV.[E]	3345.58784
6313	[P].AGAVPTPEQSAAPACAVSTPEQSATPAGAVPTPEQ.[S]	3345.59034
6314	[P].TPAPEPHTPAPFSWGTAEEEGVVAAVQEGAAEL.[E]	3345.59099
6315	[Q].QADVISQEPAMGIPSAVVPGSMAGRMTTTVAPGS.[I]	3345.61234
6316	[E].PTAQPGEGHTIPANVETSLPETDPQAVTAAGAAF.[S]	3345.62335
6317	[G].PSGGRPEPGRAAASGAAASSADPTALGGPAGAEGPMAK.[K]	3345.62404
6318	[E].GPAGFPGPPGIQGNPGPVGDPGERGPPGRAGLPGSDG.[A]	3345.63592
6319	[G].HLRTEAEGGGGESRTQAQGAGVGRSWPQSRSH.[S]	3346.61323
6320	[H].GDQGAPGAVGPAGPRGPAGPSGPAGKDGRTGQPGAVGPA.[G]	3346.66354
6321	[P].PGEPGPPGPPGPPGVPGSDGIDGDKGPPGKAGPPGPKG.[E]	3347.66549

6322	[E].APTAGPTTPNGNLVDECDDDQANCHSGTGDDF.[Q]	3348.32878
6323	[Y].RSYTTQLTMNVPFQAIHFMTYEFLQEH.[F]	3348.58163
6324	[G].PGQGGPRPPGQHYWPGPEGAPQIPGPHASSVTH.[F]	3350.62021
6325	[D].GPEPPSPAPEPAPSRAQAAEGPHLTPEASPDPVP.[E]	3350.62877
6326	[P].GASAGGIPSSIFGMAGQVPTLQSATTGGGGSPGLAFGA.[F]	3350.63214
6327	[F].EGPPGPVGTPLRFEGPIGQAGGGGFRFEGSPGLR.[F]	3350.70288
6328	[Q].VQSPMLGSPSGNLKSPQTPSQLAGMLAGPAAAASI.[K]	3350.70829
6329	[H].PANQVNGASAPTSNGSAAAGAKAREEATKEAAEPQ.[T]	3351.61598
6330	[Y].KPIAPAPSSTPGSSTPGPGTPVPTAGSVPSPSGSVPGA.[A]	3351.70669
6331	[Q].PAAEAAAPGWAQARGHPGGELAAAASAAGDAGWPNK.[H]	3352.62061
6332	[G].LSTTTILKTTTVETTHLAATGSGPTVAETTTTF.[N]	3352.73699
6333	[N].NNLLTIVGALIGSSGAILSYIMCVAMNRSLAN.[V]	3352.74256
6334	[M].NGPSSLPGFPQNSNTLTPPGAGMLGFPPSATSSPA.[L]	3353.61068
6335	[F].QTASPAPGVYPKCASPASGLPAAFSTFEWMKV.[K]	3353.63333
6336	[T].SPAVMQPPPGMSLPPADIGPPPYEPPGHPTPQP.[G]	3353.63333
6337	[V].AVSPTICRRGGAWFASFGRERNSGTKLFNI.[S]	3355.72291
6338	[I].GAPGASKAAIPSGGPLSDPELQRRVMEVELSVH.[G]	3355.7427
6339	[D].GARAAGHAGHGPAHGGLAGHGAAAAGVAVETGLEAASAT.[A]	3356.65912
6340	[F].GPGPTMGKPQSTNYAVATGNFHPSGSPLGPTSGST.[G]	3357.58044
6341	[G].AAGEGISAAPASPRSPKAGTSEGPVDSVPYLDRM.[P]	3357.63796
6342	[K].VQDGSVQVTVPGYQPGGTVEKESPDKLSVGDGQ.[V]	3357.64448
6343	[G].GPGMRRGRSSGTGVGGGVEATGPILMSPHLHPSE.[Y]	3357.6539
6344	[K].LHQTTAAAAAAASAASAVGPVHNSVPSNPVAAPGFF.[V]	3357.69746
6345	[P].AAAPRQPGLMAQMATTAAGVAVGSAVGHTLGHALTG.[G]	3357.71544
6346	[P].PGAGGHGPPSAGTPGAGHPGHGHSHPHPFPPGGMPH.[P]	3358.52085
6347	[S].ASSPAQQGLGGQAQGQPSSANMASLGAMGKSPLNQ.[G]	3358.57504
6348	[E].QGVFSQSFLGTFWTVGSLSAEGQPQTKEENV.[P]	3358.62263
6349	[T].RASPSSHGQGPKENRSGPAHGPMALSPPSLYTS.[V]	3358.63454
6350	[R].RQGGASGGQGGSSLRAPESPVPSPGTQRSPAGSPSP.[C]	3358.64828
6351	[Y].QEKANLYPPSNAPGEGLSHGGLRPNGQTKPMP.[A]	3358.6597
6352	[T].PLRAGEEGSHSRKSLCRSREEPGPGDRGAPL.[S]	3358.67814
6353	[A].INDVLWACALSHSLGKNELAAIIPLVVKSVK.[C]	3358.89193
6354	[F].RQAGDAPGPPSALLLEGKSPLRSPVRLLPLPR.[L]	3358.94339
6355	[A].ELGEAWPGAATPPLQATGWEGVRAEPPAGLTET.[L]	3359.65426

6356	[V].GLFMAGETGAVWTEACGGELPTQAGAGPGPSPDR.[L]	3360.52597
6357	[T].GASPSSSKGLENGHGHQYICLGGSVVPSPTDGAH.[G]	3360.56619
6358	[V].DSAAFRAPGAARGCSLDGLAGGGVDEGSLLCAGGPA.[A]	3360.56956
6359	[L].QPDMLPAEWTAGCAPSLGDILGSTWSETLEK.[R]	3360.57627
6360	[L].KPLQDEGQSAVPPLMTSPEAVMAMGQKHSLPA.[D]	3361.65889
6361	[P].ASPSSRPVASPAVTSMPPTSSGVREASLTSAMTK.[V]	3361.67263
6362	[I].YKPQTPSMIMQQGLTSPSPGASEPFPFGHTK.[P]	3362.61841
6363	[L].APEVGGAENKEAGKTLDQVGQCMVASAAVVTTAS.[S]	3362.62026
6364	[T].LARQGSLESPSSGTGSLGSAGGLSGGSSPLFNKPSD.[L]	3362.64588
6365	[A].AAGTQPGPAAAGEPSVSEDTLPCSADSGLCCESD.[S]	3364.3886
6366	[H].KNSSLDELEEGEIISDSEKPEPQRSFDKSA.[K]	3364.60268
6367	[G].RPGNPGHQGLAGVPGMPGTKGGPGDKGEPGRQGFP.[G]	3364.6716
6368	[R].GQLASPSSQSAAASSLGPYGGAQPSASALSSYGGQPA.[A]	3365.58803
6369	[A].FQGPDRAAATVPAQPPDPEFSFMDEEEEDE.[I]	3367.42194
6370	[S].ERSRAMASGTPGRSSACSNDSDFESGRAMHH.[F]	3367.43454
6371	[R].PAQEPRCILQSSPGPDPICVLGPSPAPVMNPA.[Q]	3368.64358
6372	[S].RSPEDLERGAAPAAAEGPAPGSIFLAGAAPPAPCP.[A]	3368.6692
6373	[V].PGAAMTIVHLIGGPMTGDTVAATGATTTAEIGEKP.[T]	3368.67123
6374	[I].QTSSSKGSVPSGHVAIKRAEESAALCSEKNPVG.[N]	3368.68631
6375	[V].PSSTAALMGGPTTVQTVAGETVQTVVQLSPTNPE.[I]	3368.68899
6376	[V].DTPVHHGVFSTLIAASVVEISHELRKVSDVE.[E]	3371.75939
6377	[V].STGSRAGGAAGVGGEAGPPPEREGSGPAKPGTPGNSPT.[S]	3372.61631
6378	[S].PMSTPDPALGGTPRPGPSPGPGPSPGAMLGPSPGPSP.[G]	3372.63512
6379	[S].SGPPAPFPGEPGPGTTPSAPLGAPQAGPAPDHSALTA.[A]	3372.64951
6380	[G].APTSSSGSPSPISSPTATPPTKPPPFNLHPAPHL.[L]	3372.72228
6381	[A].AGGKMADEEKLPPGWEKRMSRSSGAAGVGAGTGP.[R]	3374.6216
6382	[R].ERGGVGSSPDTKDQSSALAKPSQDEELMEVVE.[K]	3375.58565
6383	[G].RDGQASPARPPGSRARAPIPGPPRGGGGSGKAAAAE.[R]	3377.76459
6384	[D].GAPGQKGETGPFGPPGPRGFPGPPGPDGLPGSMGPPG.[T]	3378.63242
6385	[Q].GPPGPQGNAGPQGHLGPQGPPGPQGHIGPQGPPGPQG.[H]	3378.64749
6386	[M].LEAGQGPHASPSPQVTFRNPVIERIPRLRR.[Q]	3378.86178
6387	[L].KYHQAHAHTDDDSKPEADGDSEYGEEPTLH.[A]	3379.43701
6388	[G].EKGQTGPTGDKGSRGDPGTPGVPGKDGQAGHPGQPG.[P]	3381.61665
6389	[-].MSESKRDLSTSTSREGTALNNSNSSLLLMNGPG.[S]	3381.61868

6390	[L].EALREQMVGVQPPQAPRDLVFRTQFLDQP.[S]	3381.73722
6391	[A].GASGLQQVQMAGAPSQQQPMLSGVQMAQAGQPGK.[M]	3382.63005
6392	[S].ANPDAKAAAAPFQTSQASTSAPRHQPASTFSTAP.[S]	3382.64107
6393	[S].LGGGGGCALPVSGAAQWAPVLDFAPPGASAYGSLGGP.[A]	3382.65249
6394	[R].ASPAAASAVPGSGAAAGALASGGSKEEFVATFKGNEF.[F]	3382.65499
6395	[A].MLSLGARGNTHTEILEGLGFNLTELAETEIH.[K]	3382.69474
6396	[Q].QPGQQKNVHAHSHPKKRPRHRCSSPPPPH.[E]	3382.74235
6397	[P].VPGQTLVTMATATVTANNGQTVTIPVQGIANENG.[G]	3383.71112
6398	[Q].SPQSSFPQPAAVYAIHAHQQLPHGFTNMAHV.[T]	3384.63309
6399	[P].GVGAAGGSLSFGASSTPAQGFVGVGPFGSAAPSFSIGAG.[S]	3384.64951
6400	[I].QVQEPVDMLSSVPGATAASAGRGVSISPNTSQVQ.[M]	3384.66999
6401	[L].HLNHLEPPSSGSPLLSQLGQPSIFDTQKGQTA.[G]	3384.71826
6402	[K].PLAPEKPDPGPSESAGSIASGKVAPVALDKTDSVP.[P]	3384.75331
6403	[A].PDSGRPAPYSAAFLELQPGPAGSGYPAAAPPASFA.[S]	3385.64878
6404	[F].PSGSPTLPAQAQAGQMMPLSSARPTSGSVGVMLAA.[G]	3385.65487
6405	[R].QPLTYMAQRQPSESGRHLLSEPNTPLSPPGP.[G]	3385.69575
6406	[A].TSSSQGVKAEPSGNPISFPAPQTPPKEASQAHPG.[L]	3386.66114
6407	[L].SVDAVGLPAPPSLIKAMGGSQPGELQISWEAPAP.[E]	3386.73007
6408	[S].GPHISLPGQQGSEARIIRVSIDNDHGNLYRS.[I]	3386.73122
6409	[E].ARGVPMPDITWFKDGDPLVPSAEVVYTRGGR.[Q]	3386.7314
6410	[P].PSAPASHARTLPPPPYTTFPGSKPKFDWAPAP.[A]	3386.73206
6411	[P].GPSHPISAPQAAAAAALRRQMASQAPGKSTGTGAR.[K]	3386.74583
6412	[D].GPSAVAQAEHPASAMPGISAAASAGSSASASAHARATS.[S]	3390.60912
6413	[H].HPVATHQASGGDTQPLTSAAQAPAATPETSVASPH.[S]	3390.6309
6414	[A].THGPETLGSPPPPGGGPLDRSTTTSPASGRPSNHA.[L]	3390.64213
6415	[P].VSMPGIYAAVFALWEKVVDSTDALPIGPCGQV.[P]	3390.71125
6416	[P].RPGPGGGNGGPVGAGHGNPPGGGGSGPKARAAVVPRPPA.[P]	3391.75911
6417	[A].PLFGLAGQPPRGTSGPAPEPGPAPPTPGPRSAHGPA.[S]	3393.74508
6418	[G].GIAEAAQGAGAVPTAVTKVTEHPAGADPGAAPMIPAV.[G]	3393.74711
6419	[E].KPVMSPPEASPIKPALTCHTSTKGPLQMVYK.[M]	3393.77314
6420	[T].TAAEPQPTAPPTVCVTGPPTARPSEGPTTGPTGPP.[A]	3394.65836
6421	[L].APASQPNSLADVGSLLGPGASAGGIPSSIFGMAGQVP.[T]	3394.69474
6422	[L].FRSTGYLGDKQEIWTEHTVCSANVDIPTGN.[L]	3395.59609
6423	[K].ETKAVSEMSTEIGTMISVSSTEYGTNVKESVT.[D]	3395.60802

6424	[E].AHGLWKEPGSRVTMEVDPECVPVVRDFIR.[-]	3395.69873
6425	[E].KAVGDDVAASSSAKKATGRKKSSSLDSGTDIASVT.[L]	3395.76124
6426	[S].QGAPEAPLSPSLNTPAPVAMPASSPPGPPPAPEPGP.[P]	3396.67803
6427	[I].PGGSQSVPAASRLPGVNKEEETESRDKKEDSL.[R]	3397.68299
6428	[K].VMTLSSTAPATEQTLAPTVGTVTTFNHGIAQTH.[T]	3398.68966
6429	[G].AKGERGSSGLDGKPGYPGEPGLNGPKGNPGLPGPKG.[D]	3399.74039
6430	[K].SFRGSNFTVAPSVVNSDNRRVSSRVGGSVSQF.[K]	3400.71049
6431	[H].DAAALAAQSKSSEDIIKFSKFPAAQAPDPSEIP.[K]	3400.72709
6432	[T].RSSKEPNKLDIPNMPESKPRITNTTVPATY.[T]	3400.75293
6433	[E].KVVQGSEESTVSSPSQKEVQDPGASNVPSSTQK.[V]	3401.66667
6434	[R].RQQQQQARRKMAGWLPACVAHGVSWLELP.[V]	3401.75825
6435	[H].VTITGSPVSIALAHLETAKSTSGGTPGSAPTDLPAP.[F]	3401.77985
6436	[G].GPSGGRPEPGRAAASGAAASSADPTALGGPAGAEGPMAK.[K]	3402.6455
6437	[A].PGSISTGTTPISTVFPSTQRSTTGTWISTSFVT.[T]	3402.70635
6438	[A].GVIQAGAGQAGVIEPGAGQAGVIQPGQAGVIQPGAGQV.[G]	3407.80299
6439	[E].LTQMVSGPTSYAGPKPSTQYGAPGPFAAPGEGGTL.[A]	3408.64165
6440	[S].VSSSVSTLSHYTESVGSGEMGTLAAPPVQPQPPP.[T]	3410.64204
6441	[S].SGVNVNILSGMGNGTIASSAALNSAASAAAGMTVGSVS.[S]	3410.65262
6442	[G].GPGGAGGGGSAGGARTALSDAELGRWAELLSPLDESA.[H]	3410.65711
6443	[P].KDGVVDSTVLSSMPCLLMELRRDSSESQLAS.[T]	3410.66002
6444	[G].TTELNTELATPSFPLLETSNETSFLIGINEE.[S]	3410.67372
6445	[I].AKQGGGGGGGSVPGIERMGPGIDRIGGAGMERMGAGL.[G]	3410.68382
6446	[I].KGTAPMGTLMGSPVHLEPSNQVGVIQTKSWEM.[E]	3410.69053
6447	[G].TAAEDPLSAHQAPWTLSQSLAAKEPSSSEPSAF.[A]	3411.63392
6448	[P].KGETGPQGYKGMVGSIGAAGSPGEEGPRGPPGRAGE.[K]	3411.6346
6449	[G].GQGVLGGAGGGNGPGDPAVPGDAVSRGVPGGSGDQANPR.[G]	3411.63844
6450	[G].SPGRSSPAGGSPGKPGSTPHVSGLGSPGRYSPANGGH.[L]	3411.6537
6451	[H].GIQGGPGSQGIQGPVSQGPLMGLNPRGMQGPPGPR.[E]	3411.70085
6452	[S].HFTPSRQSQISGILHSDEEEEDEEEEEEP.[R]	3412.45714
6453	[G].SQVHFAAPPNSLVSFFDASLPGTLPVRLLFNA.[V]	3412.80522
6454	[W].KPGDSKAPCTYTLERRVDGESSWHPVSSGIP.[D]	3413.65428
6455	[S].AGEVDAGLASPPGPVSATVEVTSPTGFVHAHVLED.[V]	3413.68595
6456	[A].GEPGRAATAPTAGGEPLSPPPPQEPAPGAPQQTPW.[S]	3414.67131
6457	[K].ADTDKTAVGSSVAPGNIATSPSSPTSPTLDATASLE.[M]	3416.65511

6458	[H].GPQTVGNHFQRTPITNQSSSLTATQMSFPVQG.[V]	3416.66518
6459	[L].TSSGPPGPPGPKGDQGPPGPRGHQGERGLPGLSGSGS.[S]	3416.66902
6460	[P].GPSPGPGPSPGAMLGPSPGPSPGSAHSIMGPSPGPPSAG.[H]	3417.58381
6461	[S].SFASAGAPASGPPPASTGTSAWGEPSVPPAAAFQPGH.[K]	3417.61346
6462	[P].PAASSVPASGVGSPAGWAEDTAPVDAQPAFPADGPGL.[P]	3417.62335
6463	[V].KEDPDGEHARRAMQKVMATTGGMGMGPGGPGMI.[N]	3418.54289
6464	[V].PDSDVEELLQILDAMDICARDLSSGTMVDIP.[A]	3418.60625
6465	[R].VAEEGAAAHLENGIALSGLESCVMSAPPGSGPLEV.[T]	3419.64575
6466	[T].EPVMAPAGTTGADARSAASEEKDDAVTSAGSEGKC.[D]	3423.52748
6467	[G].VGSPGAPGKYLSSVLASAPFLAPPGASSYAAGAGGYK.[G]	3423.75833
6468	[S].REEIVEDVAQNILIKVPEPVNLQWVMAKY.[P]	3423.83447
6469	[E].ATVTAPVALGVMGPEPWAVSPVLAVAAPPGPMVAVT.[T]	3423.84187
6470	[S].GSAPTPLRCDSRASTVSPGGYMVPKGTTASATSAA.[S]	3425.64239
6471	[R].PSLEPDTSLPPNLSSSSLETPVTTETKAMGPSR.[N]	3426.69447
6472	[Q].AAAPSHTSSLPLTYSVASSVGTLQGAGPVSGPTTGAE.[E]	3426.70233
6473	[G].SPGIMGFQGFTGSRGDKGAPGTAGLFGEVGPTGDFG.[D]	3429.61683
6474	[S].PMSTPDPALGGTPRPGPSPGPGPSPGAMLGPSPGPSPG.[S]	3429.65658
6475	[S].VGVQSDEIDLSDVLSGNGKVSSCTAAEGSFTSLT.[G]	3430.61661
6476	[S].RTPSSASAGPSDPCGLKPLQQEGPPAAPSGELRD.[L]	3430.66557
6477	[G].FGAVGSTLFGNNKLTTFGTSTTSAPSFGTTSGGLF.[G]	3430.68014
6478	[T].QPGLPSSGQGGAASPGSSLGLYSPIEPGVVASGGQGPL.[S]	3433.7234
6479	[R].KTQMLQGSRGGGRRPSASGGGPFVNVSNLAGFGGGG.[G]	3434.70944
6480	[D].QMLRCLMTPLDTLTITRCLLTDSDLTHLS.[Q]	3434.71503
6481	[A].PSNGLSVRSAAEAVATSVLTQMASQRTELSMPI.[Q]	3434.72539
6482	[E].DLVQICGAADGIRLYNSLKSRSVRPRLTLY.[V]	3434.8689
6483	[N].GLQGLPGLAGHHGDQGAPGAVGPAGPRGPAGPSGPAGKD.[G]	3435.72647
6484	[K].DGKSRGIGTVTFEQSIEAVQAIYGLGGIGMGLGPG.[G]	3435.75768
6485	[V].RTLTFNSSAQGQSLGAPILEMTDPSISRSEVQ.[T]	3436.70129
6486	[A].RQAGAGAGPPNPAINGSAPRDLFDMKPFEDALR.[M]	3436.71788
6487	[F].GMKIPSGLFIPSMAVGAMAGRMVGIGVEQLAYH.[H]	3436.72481
6488	[A].QPRETPRETPRPPMPPAKPSPAPETSSAEVAGA.[S]	3436.72778
6489	[R].VQYFFAASIIANGMCHLAAPLSSTYVGLCIY.[A]	3437.67309
6490	[G].AGGLSPSSLPASSFALGGGLAADLSLHSFSDGASLSH.[K]	3441.6921
6491	[S].GPAGKDGRTGQPGAVGPAGIRGSQGSQGPAGPPGPPGPP.[G]	3441.73704

6492	[L].GPQGIRGYPGMAGPKGETGPQGYKGMVGSIGAAGSPG.[E]	3444.67872
6493	[G].SPNSVKSSVSSRQSDDNVTKLDHSVTTDKQTP.[K]	3444.68372
6494	[P].GPGANTPDRFYQQVMKEAQDQPRAERAQQE.[L]	3445.63019
6495	[S].GHSDPPTPPPPLPLPGDEGGSPASGSRGGFVLAGPGD.[G]	3446.66114
6496	[L].KQQLLEESEEPQFPGPEPSPGQVGQGGSALASPA.[P]	3446.67103
6497	[P].YAVQVGGRTISSNSFSPEAFVLPVDVEKENAH.[F]	3447.71792
6498	[Q].GKRPMPGMQPQMPALPPPSVSATGPGPSPGPAPPN.[Y]	3448.68103
6499	[A].TSAQPAAATPASVSSPAGSPGPPGSTASLSTASLTPSSP.[R]	3450.68708
6500	[A].EGVGAAANAAATSSTGTGGVAASGMAASGVVPGGGFVASAAA.[E]	3451.63941
6501	[A].GPSVTSVPQLSQELSGLPAPSMGLEPPQEVPEPP.[V]	3451.73013
6502	[Q].QERKEAARPAYEAVDGTREANNRLVTYVGTS.[E]	3451.73128
6503	[P].QSPRDMPPSLVSMLQPPADTSQSPVNTPPSPGH.[A]	3452.65731
6504	[M].GPLGTGFHGNTVSSPQSSAATTPGSPSLGRHPGAHQ.[V]	3452.66902
6505	[G].PGANGMPLAGLAWSSASAPPPRGFSAISCTVEGAPA.[S]	3452.67257
6506	[G].QPGEPGARGEPGDPGLPGRPGTSIGDEDEKRGLPG.[E]	3452.67891
6507	[P].RTNFHSSVAFMFRNPPAVAMASPASAPAPAPSPA.[P]	3452.69906
6508	[C].YVSDITRTWPVNGRFTAPQAELYEAVLEVQ.[R]	3452.74849
6509	[G].IQDGKYPGQFPRGTMPDRLSKRVTFALTAW.[M]	3452.78959
6510	[T].DIFPEPQGQAFPGSAGPALQYPPPAYPGAKGGFQ.[V]	3457.68517
6511	[E].FVFSKDSEASGQRVDGLAFVNEDVVASKGSGPGT.[I]	3457.68702
6512	[I].KQTEGEGTDVRDHTVLEMKAGFSLPDAGDLLE.[E]	3458.6744
6513	[C].GQLSTGPHRGHLHPGAGGRERCASPLGAGAAGSQGP.[A]	3458.69552
6514	[Q].QQPATGPQPSLGVSFGAPFGSGIGTGLQSSGLGSSNL.[G]	3458.71865
6515	[G].GDVPPAPLAPAGPAPYSPPGPGPAPPAAMALRNDLGS.[N]	3459.73655
6516	[A].ARGTGAAGNAARMSASASGGGGGDSDSSSSSQASCGPES.[S]	3460.43202
6517	[G].VGGAGPPGHGGTAQVTHGGAGSALAYRTPMDSSPTIL.[M]	3461.68664
6518	[F].LNGRDYRSGEPVGSGDPCSHCHCANGSVQCE.[P]	3462.40628
6519	[R].APGRGTESPFEGKDGKEASPDQISPVSDMTSTAL.[F]	3462.63293
6520	[G].INQTGDQASQNKPSSDLSVPGNPERSAQSLSSY.[N]	3462.63677
6521	[M].AAGSQKHSHDSGVPPVVDERTGSEGATASPSLGHH.[S]	3462.63811
6522	[T].GKKGPMGPEGEKGEVGPPGPPGPKGDRGEQGDPGLP.[G]	3462.70704
6523	[R].GPAGPSGPAGKDGRTGQPGAVGPAGIRGSQGSQGPAGPPG.[P]	3462.72211
6524	[D].HISQPYGSGKMKMFQEVPAVDAFGPGIEGQVE.[R]	3465.64535
6525	[N].TTQLGASGGQGAPTPTPAPASTSQEPPLPSGPATAPAP.[R]	3465.71323

6526	[G].QGAPSRDRNLGGPGQDTPGVSLHPLSGDSPDREP.[E]	3466.66941
6527	[L].PQSTGLGSPEAPHPVPGGGEGPPKTGTAPSPGPPCPP.[V]	3466.66959
6528	[T].EIRFSELPTQMFPAGATPAEITRHSMDLSYA.[L]	3466.67699
6529	[A].GQVRLTYSTGESNTVVSPTVPGGLSDGQWHTVH.[L]	3466.69858
6530	[S].QGSINSPVYSRHSYTPTTSRSPQHFHRPEL.[V]	3466.69992
6531	[G].TPGMPGVKGSAGQAGRPGNPGHQGLAGVPGMPGTKGGP.[G]	3466.70666
6532	[D].QGVPGERGPAGPPGPQGPPGEQGPEGIGKPGAPGTPGQ.[P]	3466.70982
6533	[A].EARAEAGREAGEGGVAAAAAALAPGGFLGLPAPFSEE.[D]	3467.71899
6534	[Q].QGLAGPLGDPLGGDHLAAGGDVPPAPLAPAGPAPYSPP.[G]	3467.75939
6535	[Q].PEHAGARGHPGIHSGPATSVDKNLSSHPCSAHLS.[Q]	3468.65741
6536	[T].SVWSSVPMMSPPLASPSRAASQATTPPASSLCPP.[G]	3468.65962
6537	[P].PQMSAAAAAAAAAYGRSPMVGFDPHPPMRATGLPS.[S]	3468.66096
6538	[G].PGANGMPLAGLAWSSASAPPPRGFSAISCTVEGAPA.[S]	3468.66748
6539	[L].GQAVGQQPSASQVSPTEPRSSPSPPPSPMEPPEK.[S]	3468.66999
6540	[I].QGGPGSQGIQGPVSQGPLMGLNPRGMQGPPGPREN.[Q]	3468.68593
6541	[S].SVGMGQPAAQAGVPQGQVPGTALPNPLNMLGPQASQ.[L]	3468.73623
6542	[V].TADLTLEDNRRMIIMEKGPLPGPATGLEAASGQ.[G]	3468.74613
6543	[V].DIHTGLSGSGVAFVCAFSEALAEGAVKMGMPSGLA.[H]	3469.64364
6544	[P].PAASTPAGPPSGGASPTPPAASPSGGSATRPSSGPTSEAP.[R]	3469.64661
6545	[K].GPPETRSSLELDTELSWISGDLLPTDSQEEAP.[S]	3469.64929
6546	[G].NGLSTPPGPGGGPHPPHTPSHPPSTRITRSQPNH.[T]	3469.72205
6547	[D].QGLQAAYTLAQELGHVLGMPHDDSKLCTRLF.[G]	3469.7355
6548	[H].GAPQAPAASTRTLLGLSPSSSLSHTVGPTGHLQCL.[V]	3469.78563
6549	[P].AREPGDVSAGPRSGGGRNATTAMPPPVPNGNLHPH.[D]	3471.70469
6550	[L].RNVMRISPDQGQQFAQMLVQDEEPLADITQ.[I]	3473.67878
6551	[R].VGAAGVSETQDALGTQGALEPPAGEVAENPLEPGLE.[A]	3473.69183
6552	[P].GPAAGAPPPGCATLPRMPPDPYLQETRFEGPLP.[P]	3473.69806
6553	[L].PGTPGHAVEGPKGDRGPQGQPGLPGHPGPMGPPGLPG.[L]	3473.71313
6554	[L].QDVQPSPINPFRAFFEEQERQAQNSSVAGTP.[G]	3474.66728
6555	[V].PSPSGSVPGAAAPFRPLFNDFGPPSMGYVQAMKP.[P]	3474.69733
6556	[P].GAREPPPPAPAPAHHHPEYQGQPVVSHPHHIM.[P]	3475.69775
6557	[L].GPQGPPGPQGNAGPQGHLGPQGPPGPQGHIGPQGPPGP.[Q]	3475.70026
6558	[G].INGKNDVVAPEMQSGSPTLMKAAAAVEKDSSRAS.[P]	3475.71556
6559	[Y].GYPPSSLGRAITDGQAGFGNDTLSKVPGISSIEQG.[M]	3476.72922

6560	[G].RQELGNSTKAGFLDLNDRPSGFGQKPSSGATQL.[N]	3476.75168
6561	[G].NPRGDQSPPVSAPGPAPSLQLSSYGQPLQPGSGPH.[D]	3477.71457
6562	[V].QMVESIGGLLGAPLSGYFRDVTGNYTASFVVAGT.[F]	3477.73588
6563	[S].AGGSGGCSSSGGGGGGSSGGAGGTSGAQNPAGPGGISQHLTY.[T]	3478.49085
6564	[S].GQISYDEVGERIKDFLQGSSCIAGIYNETTK.[Q]	3478.67949
6565	[S].PVTLSAMTSPSPESPTLPGRSPSSPTPVTSLFTH.[G]	3479.73628
6566	[D].NNTNTLGRNVMSTATSPLMGAQSFPNLTTPGTTS.[T]	3481.66861
6567	[D].QSQAGWPPPATSLQPASFSGRSSSPPAQWEGTLA.[R]	3481.67712
6568	[C].GPSPGAPPMPVEAGGKEASSQPNICILTLAMMIAG.[I]	3481.68339
6569	[S].RPSSPQATTPNPVPSSTEAQGVAGPAAEIPASGGHGT.[E]	3481.69423
6570	[G].GVGAGLGGSLGDGGLFSGGEKQTMQNLNDRLANYL.[D]	3482.69687
6571	[G].QGVHHAAGQAGKEAEKFGQGAGKEAEKFGQGVHH.[A]	3482.70607
6572	[S].PSPRMQPQPSPHHVSPQTSSPHPGLVAAQGNPM.[E]	3483.66446
6573	[L].SSDPKPGRNANSDLLASPDHRSGLSPDTDPVPSP.[D]	3483.67349
6574	[F].TPPPGSTMSPLPSGSNPYARSRPPFGQGYTQPGP.[G]	3483.67501
6575	[L].HMLEPVVDHERGNIIIHGELVAVSTCGEGDVT.[G]	3483.69951
6576	[R].RGLLSRDAGSPEHSAASPPSPGAARPQTPTAVEDT.[A]	3483.72111
6577	[E].KEDRFLTTLSSQSSTSSPHLQLPTSPEGVPEQ.[A]	3483.7238
6578	[L].GPAGPGTGGPGVASPTITVAMPGVPAFLQGMTDFLQA.[T]	3483.72869
6579	[S].AASSPLSSSLGTVGHRANSPSLFGTEGKPKMEPVA.[S]	3483.75366
6580	[R].STLQAIGSAAAAGMVFYSISDVTDKLLSTSGDPIP.[T]	3483.75634
6581	[P].ASSALGPASPGLPWAAQGAREASLGPQPGLSGGSAGVGP.[E]	3483.76152
6582	[V].PGAAGGAGDRRLEEALGALVAALDDYRGQFPELQ.[G]	3483.76152
6583	[G].PGTASSAVPPTEDFLPLPTGFLQMPRGLTDLEI.[G]	3483.7716
6584	[R].QRLDGGSGGGPSAAGPGFPAALHDFEMSGNMSDDM.[K]	3484.44746
6585	[P].GPVSTTAASPESPPTPQTSLTPPQASPAASKDQSPP.[P]	3484.70781
6586	[H].VVLDEDAGQEAVYQASVQPLLEAFFEGFNVTV.[F]	3484.71586
6587	[Q].AQAAPSPAEDLQRKDTATPDRGFFPGAASGLRGE.[L]	3484.72038
6588	[V].RATAFPAVENRTGEATRPWAFPGESTPGLEAPT.[A]	3484.72441
6589	[Y].GKAWAAGDIVSCLIDLDEGTLSFSLNGVSLGTAF.[E]	3484.73046
6590	[L].QATSVPTLGGAGPQPSSAPAAFLPCTFHPPAPVQGA.[P]	3484.7318
6591	[T].SRPPATNSGVFAATTGPIQAAFDASVSVPSEGLPQG.[T]	3484.7343
6592	[E].HEFKKAADEDEKKARSGTLAASGPLDMSLPSTP.[D]	3484.73767
6593	[V].TADLTLEDNRRMIIMEKGPLPGPATGLEAASGQ.[G]	3484.74104

6594	[S].TVEGSGRSEPGQVGLLIPEMKDTSMERVGQPLS.[K]	3484.74104
6595	[G].GRPQGFQGMPSAICQSGPRPALRHLAPTGNAPAS.[R]	3484.74372
6596	[S].EDARARKAEGAEAAARERAAVSGKDASSPAATSQV.[S]	3484.74872
6597	[P].QGPRAGPEGAGGGPGLPPALPLQMFELGEGVTGTVGP.[T]	3484.75293
6598	[R].PPPDPLSPPGTPMVPEMPRLLLTSDLDSFAVTP.[Q]	3484.77424
6599	[N].TNTLGRNVMSTATSPLMGAQSFPNLTTPGTTSTV.[T]	3485.68867
6600	[Q].LAHHGPHGLGHPHAGPPGSGGQPPPRPPPGMPHPGP.[P]	3486.72497
6601	[L].PPMSQRPPAIPPMPPGIMPPMLPPMGAPPPLTQ.[I]	3486.74785
6602	[D].AVHSGSLSRSSPAVPHSTPRPVSDGGKMVNAAVNT.[Y]	3486.75064
6603	[S].KPGNGQFSPRASCGLGGATALPLFQSEVEALAEQ.[L]	3487.72744
6604	[L].PPGSQPVPPPGTAGPPAVHGLAMAPASVAPAPAGSGAPPG.[S]	3487.77908
6605	[G].PGSPATLSPSAGVPQPVGMEALDQAEGPAASQRAMP.[P]	3488.67844
6606	[A].PPPYASPTAPSPSSPVPTPSPSTPAPPTSSPSSPAPP.[NT]	3489.70602
6607	[L].PGLSGSGSSSLGLNLQGPPGPPGPQGPKGDKGDPGVPGA.[P]	3490.7561
6608	[G].QGVHHAAGQAGKEEDRLQQNVHNGVNQAGKEAN.[Q]	3491.68713
6609	[G].PMGLQGMQGPKGLDGAKGEKGSSGERGPSGLPGPAGP.[P]	3491.70058
6610	[M].PGSGIGTGPGVIQDRFSPTMGRHRSNQLFNGHGG.[H]	3491.70977
6611	[E].PAMGIPSAVVPGSMAGRMTTTVAPGSIAGGMAPSLPPG.[S]	3491.75175
6612	[G].GPAGPGGPGGGAGVRSYPVIPVPSKGFGLLQKLPPPL.[F]	3491.95255
6613	[L].APSPVTDLHHQAHLQGTPPAGHLHASSLMANTSL.[H]	3493.73934
6614	[G].SPGFPGVPGSPGIMGFQGFTGSRGDKGAPGTAGLFGE.[V]	3494.67976
6615	[L].PGQPGGPFLNTTLAQQQQQQHSGGAGALGGPSGGFF.[P]	3494.6836
6616	[T].ESVQDQLPYSVTHISMPATTEGRRGFSVSVES.[A]	3494.68564
6617	[N].MHVVDVELSGPPGPTGRSFTVHTHRENPAEPGA.[V]	3494.68697
6618	[M].GGGRESGLPASRMHRTLSASEGRWGEVTHEMR.[S]	3494.68766
6619	[G].GEARPPEHPQSGQTAEALSLSRRAQMTHPGQGP.[Q]	3494.69418
6620	[Y].AVDLSEPQKAGAGDGSARREPYAPYPAGYPRTF.[E]	3494.70875
6621	[M].GNVTLDHFGEVPGGLAGGGQGREVQWQVFVPSAE.[S]	3494.70875
6622	[E].GPELVADSNVVLDTTMRGGRLGVFCFSQENII.[W]	3494.74065
6623	[G].MQGPPGPRGMQGPPHPHGIQGGPGSQGIQGPVSQGP.[L]	3495.6757
6624	[P].QGPPGKPGPAGMKGEDGLPGSPGEKGEKGETGQPGPP.[G]	3495.68089
6625	[H].SSPTSPALSENAPGGKPGINQTYRSPLGSTTSAPAP.[S]	3495.73503
6626	[Y].KTYSQGAPEAPLSPSLNTPAPVAMPASSPPGPPPAP.[E]	3495.74645
6627	[D].EARGAGPPGTAGPRPEAAGPGTASSAVPPTEDFLPLP.[T]	3495.75029

6628	[V].VQVVSAMAHHGYLEQPGGKAMVEYVVQQCALP.[P]	3496.71741
6629	[W].GRETPPSPQAEAGGPRGTILMSSHKGPAVAQGNGAP.[A]	3496.73499
6630	[A].YDLAGSCKGVKVNGFRSYVDLYVKDKLDETG.[V]	3496.74169
6631	[A].PAAASPGCQARPGGKPASSALGPASPGLPWAAQGARE.[A]	3496.75024
6632	[S].NSLNVNNGVPGGAAAAAAATVAAASATTAASSSLATPELG.[S]	3496.75141
6633	[P].PQYVTTSTSNIVSATSVQNFQVATGQMVTIAGVP.[S]	3496.76282
6634	[A].ATEAALAAPAAEAASAAPDPPAAGAAPAAPAAPAAPAAPAAP.[A]	3496.77069
6635	[Q].MVTGLAANLVNALANYLFLYQMHLGVMGSALAN.[T]	3496.77895
6636	[K].GEIAFDPRSAYYLWFVMDFCDGGDMNEYL.[L]	3497.47993
6637	[V].NGNRGGAPNVAVVMVDGWPTDKVEEASRLARES.[G]	3497.719
6638	[L].LDGRGEGGRSRDLGRSAGGSDTSEGLKPRNHFGV.[G]	3497.73408
6639	[P].FIPQKKNPDTFMIPMALPNDNGNVSGVEPTPI.[P]	3497.74434
6640	[Q].TGPPPSYRPGLRMFPETGGTTGCARPPPVSFLP.[R]	3497.74567
6641	[-].MAGWIQAQQLQGDALRQMQVLYGQHFPIEV.[R]	3497.74568
6642	[M].HLSTSNLSMGEMTAGQICNLVAIDTNQLMWF.[F]	3498.61604
6643	[S].ASAAAATPHSAASRSNSLVSSFPMEKRGFYESLA.[K]	3498.70704
6644	[K].ESKPSGNTSIVPEISRHAGETPNSTASVEGLPNH.[V]	3499.70479
6645	[G].QMPGSGIGTGPGVIQDRFSPTMGRHRSNQLFNG.[H]	3499.707
6646	[P].SSPAPSGTAPSGPAASRAHLTPPCSPGAPAEATPHNP.[R]	3500.66115
6647	[C].QPSAASMAAVAQRSMPLQTGAAQICARPDPFQQ.[A]	3500.68315
6648	[G].SPGASGGARDVRTEAAAEVAGAPGARAACAPANPAGAPA.[R]	3500.70475
6649	[P].QAPSPLGSPFLWPGVEGPDSPSSPKPGAPHATPHT.[G]	3500.72334
6650	[K].SPLNQGDSSAPSLPKQAASTSGPTPPASQALNPQAQ.[K]	3500.72519
6651	[P].RSSSSAPPANPPSGLVNPSLPFTSSPDPTPSQNPL.[S]	3500.72922
6652	[K].VGDVTAKRMSCHAVASGNKLYVVGGYFGIQRC.[K]	3500.73479
6653	[G].RGENPGFQVHKNNGVIFNNGPTWRDTRRFS.[L]	3500.74312
6654	[S].MVPSAATAPGTVPGPGALQGLGGGPPGLAPAAVSSGAVSC.[P]	3500.75121
6655	[P].QPTSPQRSPSPLLGHSLGNSKMAQAFPSKMHSP.[P]	3500.75255
6656	[A].AGRPGTEAEVAAAPAWPGLAEGRRRAGAMAEAGPQA.[P]	3500.75639
6657	[T].AGPPAVHGLAMAPASVAPAPAGSGAPPGSLGPSEQLGQAG.[P]	3500.75908
6658	[Y].QGQLVPQDHQGPPGPPGLPGPKGEQGLQGPPGPPGQ.[I]	3500.76694
6659	[G].QGPLSQPAGISTNPFVTGSSSSPFAAKPPTTNPFL.[-]	3500.76962
6660	[A].AFPPSLMMMQRPLGSSTAFSIDSLIGSPPQPSPG.[H]	3501.72149
6661	[D].SATQWFTGIITHHDLFTRTMIVMNDQVLEP.[Q]	3501.72936

6662	[A].VYFTRTEWAGLSPAQRALYRSVMLEICGNL.[M]	3501.77698
6663	[E].PSLAQSPMTTGVAPSLSQPSLTFGVSPCQTQPSF.[T]	3505.69778
6664	[M].NRSPSPISNQPSPRNQHSLYTATTPPSSSPSRG.[I]	3505.71669
6665	[A].AEGVGAAANAAATSSTGTGGVAASGMAASGVVPGGGFVASAAA.[E]	3506.68161
6666	[L].ESMLQVLGPGVGDLYADATDKSPGRTSWASPRE.[G]	3506.68564
6667	[A].QARLSAMAGSNGSKHASRQDAAGKDSPNRHSKGE.[P]	3506.70139
6668	[Y].NEAKTNRSPARGSGFSGKSDIPNTSLDSTSQPVT.[E]	3506.71061
6669	[S].PGFPGVPGSPGIMGFQGFTGSRGDKGAPGTAGLFGEV.[G]	3506.71615
6670	[E].AGGPDLGVGRNSGSLWPGDQAPEDRRLAPNQRY.[N]	3506.7272
6671	[P].PAGQAPFQAQPAPPASRMLTGSHSFAASGMAGVPVV.[P]	3506.73075
6672	[A].GPGLGNVAMGPRQHYPYGGPYDRVRTEPGLGPE.[G]	3510.69714
6673	[N].QGVAPMVGTPAPGGSPYGQQVGVLGPPGQQAPPPYPG.[P]	3510.74745
6674	[P].ASAPPATPSEAPAAASSPATAPAPAPAPASASAPAPVPAPA.[P]	3511.77035
6675	[Q].GQAGIQGPPGPPGPPGPSGPLGHPGLPGPMGPPGDPGIQ.[G]	3512.73795
6676	[G].GSQAPGRATGPARERGGPSAPEGARAAPDACGRARP.[A]	3512.73845
6677	[V].GSPLTSSISSSITSSLAATPPSPAGTSSVPGMNANALP.[F]	3512.74248
6678	[D].AVQSAFYPQKSFPEKAPANGVEQTQKMVTPAY.[N]	3512.75187
6679	[A].GTPPPPPAPDPAPLELEPAAEEGAVGPPELSRGTAQ.[P]	3512.75437
6680	[V].GGAVGAGLMGLAGGVVGAGMAAAALAAEAGMVAAGAAVGATGAA.[V]	3512.76582
6681	[R].IYLDMLNVYKCLSENISAAIQANGEMVTKQP.[L]	3513.74262
6682	[V].RISDTGLATGTGPEKQKGSWSQAPGENSRNSILA.[S]	3513.76806
6683	[F].AAMPPGEEVKSTLSMLPMVLPGMAAVPQMFGVGGL.[L]	3513.76864
6684	[Y].IEGYVPSQADVAVFEAVSGPPPADLCHALRWY.[N]	3514.71
6685	[P].PGPSGPLGHPGLPGPMGPPGDPGIQGYHGRKGERGM.[P]	3514.72192
6686	[P].KLAHSMAHPCPVNRTSKYTMMKTHLFQASAA.[Y]	3514.73268
6687	[M].PGQPTLMSNPAAAVGMIPGKDRGPAGLYTHPGPVGS.[P]	3514.75697
6688	[Q].TQQRPTDMSALNNLFGPQKPKVSMNQLSQQK.[P]	3514.78933
6689	[N].AQMLNGMIKQEPGTVTALPPHPARAPSPPWPPQ.[G]	3514.80861
6690	[I].PGFPGAKGDAGTPGPPGPAGIAVKGLNGPTGPPGSPGPRG.[N]	3514.81897
6691	[R].AAAPSLGEGEGVVSVSVSPGPLPTRKAMSLDVHVPS.[L]	3514.82101
6692	[R].PGAQTPTAVYQANQHIMMVNHLPMPYPVPQGP.[Q]	3516.68611
6693	[G].LFGTATNTSTGTAFGTGASLFGQTNTGFGAVGSTLFG.[N]	3516.69177
6694	[L].PNRSDLSGVDITMLNMLNRRDSSTSTVSSAYL.[S]	3516.70572
6695	[A].AINVSTSHAVPAMHQVSRASNSITVSWPQPDQT.[N]	3516.72884

6696	[L].QTLPQAAVCPTDLPQLWKGEGAPGQPAEDSVKQ.[E]	3516.74276
6697	[G].NQPAAPFSPSRNVTSPIADFPAPPPYSAVTPPPE.[T]	3516.74341
6698	[G].AGVGGFGGSGGFGGGISGAVGGFGGLGGFGGGISGAVGGFGGLGG.[F]	3517.68835
6699	[L].SHDLQISSGVTQDVWLNSPVGNSTLSHTGGTVSH.[Q]	3517.69423
6700	[I].AVGGFRAGSCSRSFGYRSGGVGGLSPPCITTVSVN.[E]	3517.70633
6701	[A].AAPGPGGERGQAGLLFGGGDERVAQLDWQGPGKDR.[D]	3519.7476
6702	[S].PQDPIHPEVYSLPSSSGLAGQDGVSTSLSLSSHGGG.[S]	3520.68266
6703	[K].GEKGETGQPGPPGLDGPTGEKGEPGDPGRPGATGLPGP.[I]	3520.69389
6704	[S].QALQELTQMVSGPTSYAGPKPSTQYGAPGPFAAPG.[E]	3520.70531
6705	[A].AAAASAPAPGPASSPEASPAPGFPFPPPWMGMPLPPP.[F]	3520.70683
6706	[G].PGGQELSAGDTGVNTAYLATTAAVSSLPSRITGEQT.[G]	3520.74017
6707	[I].SGVGISTPQYSTARMTPPPGPQYGVGSVLRSSNGV.[V]	3520.74891
6708	[L].EHLQLLSNQLLAPPLPDGTISSSSILLAQSLQH.[C]	3520.90097
6709	[E].TFHGVIYGFNVNAGNVIQQLAAKKGVRIKLHK.[V]	3521.00157
6710	[N].MNLSRSSSDNNTNTLGRNVMSTATSPLMGAQSF.[P]	3521.60535
6711	[P].KQQPSRQPFTVNSMSGFGMNRNQAFGMNNSL.[S]	3521.61072
6712	[P].GPAGPMGPFNPGPFNQGPPGAPPHAGGPPPHQYPPQ.[G]	3521.65964
6713	[E].RNGTLQDNTAQTSAYIQYEPHLLFSSESTLH.[R]	3521.69316
6714	[I].PLGSGSGMIQASSARAERGAVATDMPVGSQSKEEK.[C]	3521.69588
6715	[IS].DPASLGAEHLQVLDSEAYYLHLDVTDGHFVPN.[I]	3522.6812
6716	[S].SPVADQVTGQPSSQLQPITYGPSHSGTATTASPAPS.[H]	3522.69831
6717	[P].PGLSSSGVSAASQGAGGGPPPAPPLPTAQGPSGGGTGAPSLA.[S]	3522.74593
6718	[V].MMSIRTKLQNKEPVTEALTGPSASSLATRRSM.[S]	3523.80293
6719	[R].SPPPPPPPPTLLSSGHPVPTPSPLPFTQPGPAFSQ.[Q]	3523.82602
6720	[G].PGPSPGPAPPNYSRPHGMGGPNMPPPGPSGVPPGMPG.[Q]	3528.62457
6721	[T].PGSTAAAPAGLHNSMPSYSLEQSYLHQNAREDL.[P]	3528.64483
6722	[G].GPQSPLVDGQPAEGRASGDQNLQDAPAETAAPALAE.[V]	3528.68372
6723	[P].AASTPAGPPSGGASPTPPAASPSGGSATRPSSGPTSEAPR.[P]	3528.69496
6724	[T].GTPLQNSLMELWSLMHFLMPHVFQSHREF.[K]	3528.70137
6725	[P].PMGPPMGIPPGRGTPMGMPPPGMRPPPPGMRGPPP.[P]	3528.70158
6726	[E].EGTPPNIAAMAVPTSIYQTSTGQYSATIVQYAAQ.[S]	3529.71554
6727	[P].PGPEPSTSSLGTSLGPTTTTTTTSSVATLSSPKTHTSG.[T]	3530.73442
6728	[E].RPTSVFYQRADMAIGSLTINEERSEIVDFSV.[P]	3530.75841
6729	[D].SEDVIFPNSPDNEEHNMHSHDVSPDIQSEPI.[L]	3531.52411

6730	[K].YFLTQSTASMLLMMTIIINLMFSGQWTVMK.[L]	3531.74685
6731	[P].PGGSLEPAPSSQPAKPTASSVTPKRPPPAPPAAMASP.[P]	3531.82643
6732	[G].AGGPAPAASAAPAGGLAPSTTAAPAEEKKVKAKKEESD.[D]	3531.82893
6733	[D].GQVGASTPAAGGPQEVTYAQLNHKPLTQRAARAVS.[P]	3531.8415
6734	[P].TCRGRGQRRCAPGLGAAPQAADPADPLAEAWGAQ.[H]	3532.70331
6735	[T].PGSGQLAGTNDLASTPGSGSSSVSAGLQKMVIENDLS.[G]	3532.70716
6736	[-].MQSLELQPSLSATFFVSGRSGGDAVEAALVCHTGV.[K]	3532.73767
6737	[L].QDAVGKLDDPLHLRCTSSPGRAPACPPSVPTPH.[P]	3533.73763
6738	[K].AAAEPVEAGEIPGPIASCQELKGSADSFVDPPETP.[G]	3536.67373
6739	[L].KGPSGPGGKGSPSEMKSTTAGFPMYPGKDGPGSGPSK.[G]	3536.67844
6740	[P].EAQVDIHTGLSGSGVAFVCAFSEALAEGAVKMGMP.[S]	3536.68584
6741	[S].PNSLHYISPSGVNEYLTAIWSVGLVIQDYDAD.[K]	3536.722
6742	[P].GAPGAPGFSGPKGEPGDILTFPGMKGDKGDLGSPGVPG.[L]	3537.73186
6743	[L].QAAGVAADWAAAGLADGARAAGHAGHGPAHGGLAGHGAAAA.[G]	3538.71837
6744	[A].GKTSTFRMVTGDTLPSGGEAILEGHSVAQEPAAAH.[C]	3538.72308
6745	[A].TAAANNNFVNFGVADLNAVQSPGIPQGNSGVSTLHG.[I]	3538.73095
6746	[P].PGAKGEPGTRGPPGLIGPTGYGMPGLPGPKGDRGPAGV.[P]	3538.82235
6747	[T].LDPTSLSTRPSSGCAQVLSHPGHAPVTSLAWAPSG.[G]	3541.74924
6748	[G].RSPMVGFDPHPPMRATGLPSSLASIPGGKPAYSF.[H]	3541.77189
6749	[A].FEKMKGQMLGSRSLEDDISLAHSVYASSLQSQ.[V]	3542.72539
6750	[D].KNSEELVEAQTPTSTPEKPQELVSAEATAPSTSS.[S]	3543.71844
6751	[V].FCGPARLASSALQEPSSFRADGIPNDSSDSEME.[D]	3544.55912
6752	[S].RRGSFVNSSGVMNQGVAPMVGTPAPGGSPYGQQVGV.[L]	3545.73763
6753	[P].AGPRGHPGPSGPPGKPGTGSPGPQGQPGLPGPPGPSATGK.[P]	3545.79964
6754	[T].IDAMFGAPRFGGSRAGPLSGKKFGNPGEKLVKKK.[W]	3545.95257
6755	[C].APVFQAGNPGEPGLRGPEGSRGLPGEEGPRGPPGPR.[G]	3546.79488
6756	[V].GPAVSSGVNVNILSGMGNGTIASSAALNSAASAAAGMTVG.[S]	3548.73193
6757	[V].QGSKGPPQMGLSALTWAGSQRAGLASNGHAQQRGE.[L]	3549.73638
6758	[G].GPGSQGIQGPVSQGPLMGLNPRGMQGPPGPRENQGP.[A]	3549.74378
6759	[V].PGAPGMPPGIPPLMPGVPPLMPGMPPVMPGMPPGLH.[H]	3550.76139
6760	[A].ASVDRPGSNGVLYAVFSTDGRGGGGPRTGLCLFPL.[D]	3550.78596
6761	[G].FLDLREDSRGSIQIAGITEVSTSNAQEIMQLL.[T]	3550.80575
6762	[G].PLGGGGAGGPQMGLPPPPPALRPRLVFHTQLAHGSP.[T]	3550.88522
6763	[T].EPVTIFSHQSETTAPPPAPTQALPEFTSIFSSH.[Q]	3551.7329

6764	[P].PRTEDPGDAVTRDPLGPSRSETLRCPLCSRVG.[S]	3551.74417
6765	[P].TQATPAPGVAFKSAFSPYQTPVPPFPPPPEEPTT.[T]	3551.77331
6766	[R].SPSRSPNRSPGVPPAPEMALPRPSTQGAGPGERLS.[P]	3553.79284
6767	[A].ASTPAGPPSGGASPTPPAASPSGGSATRPSSGPTSEAPRP.[P]	3554.71061
6768	[T].PGAAGGATAASAAASVLGGSAAPATAGDTTKSENVAPADRS.[A]	3554.73173
6769	[G].KDPTPSMLGLCGSLASIPSCKSLASFKSNECLV.[S]	3554.73616
6770	[A].PDQAPAALTNRSGHRDPGPPGSSNHGGRTPPATPAL.[A]	3554.75956
6771	[G].TAQLPTPGPTPAPAAPSQGSPLASQPPTQPQAPSAAAP.[H]	3555.8078
6772	[I].LQSLCDQGCCGDDSGLMEVEGAHSARTMSINAA.[E]	3556.48656
6773	[R].KPSDDKHFGQAPNKGTSSDGVSLSNLAQLSLTAAD.[Q]	3556.75141
6774	[V].SPTSGRSTPNRQKTGSPGSVGVSLGGTQKSSVSSALP.[-]	3556.83139
6775	[P].GPKGETGDPGPPGLPAYSPYPSVAKGVRGEPGSPGQP.[G]	3557.76594
6776	[T].SLKSTEGGAAGTTSGLAIAGTTTSPGGGVAVAGVGAGSVGGAV.[S]	3558.82457
6777	[-].MLQDIGEAIQFEVSIGNYGNKFDTTCKPLASTT.[Q]	3559.7261
6778	[N].DTVVFACVFGFTMKGSKAVRCNAQGTWEPSIP.[V]	3560.71233
6779	[L].KDKEPGSFIVRDSHSFRGAYGLAMKVATPPPSV.[L]	3560.83185
6780	[H].VWPMYQKYKREMEANGVEVVYLDGMKPRE.[E]	3561.73273
6781	[E].GGKVPDPGDGAPAAATEAALAAPAAEAASAAPDPPAAGAAPA.[A]	3561.74559
6782	[S].HLQSPPHAPSSAAFGFPRGAGPSHPPAPPAAPEPLGG.[I]	3561.77744
6783	[Q].GLHKGQSSHLAGPNGERPLSSTGPSQHLQAAGSGIQ.[N]	3561.79053
6784	[L].PPRPGEAPGHSGPAAGAQARSTATQEENGLLVGGARP.[E]	3561.79053
6785	[P].RGPAGPSGPAGKDGRTGQPGAVGPAGIRGSQGSQGPAGPP.[G]	3561.80176
6786	[G].PETKPVLMALGEGPGAEGPRLASPSGSTSSGLEVVAP.[E]	3561.8105
6787	[R].IMKALMGSRPGRPSSLPEVSHSEGRRLSHSAQA.[L]	3561.81253
6788	[V].QVGPEAGMQKVRAWGPGLHGGIVGRSADFVVESIG.[S]	3561.83833
6789	[T].QPRLEEAASSAAVTEVESAVRPEVASPREEAAEP.[G]	3562.76197
6790	[T].AATGPLFGAPVTSAGMFGGTSLRFGLNFSGAYGAAAVA.[S]	3562.77875
6791	[R].QMLPPVEGVHLLSSGGQQSFFDSRTLGSLTLSSS.[Q]	3562.78462
6792	[P].PAGGPPPPPGPPPPPGPPPPPGLSSSGVSAASQGAGGGPPPA.[P]	3563.79176
6793	[F].ASRVHSSQDLHQGSGNKHSVLFSMRSHQRGSF.[M]	3564.73739
6794	[A].PGQQRARPSTTSSGPSQGPSGSTPRPSTTSSQGPALG.[Q]	3564.73855
6795	[V].HTQSRVEPSAPWCLRARDSGSLAPQCGSVLEL.[S]	3564.74344
6796	[R].RPCPMVAPGPEGLSTPRSQGPHVTLGGSKGHGAQSG.[S]	3564.75468
6797	[T].TPTPGSTTGLPLPSTGPTSSPTAPPASATSTGAPTSTGR.[P]	3564.76639

6798	[A].GPPGPPGPMGPPGLPGPMGIPGSPGHMGPPGPTGPKGTSG.[H]	3565.70249
6799	[G].ANQLNAPARLGIMSSEEMGGGRGGPVAYGAIFPGFG.[G]	3565.73148
6800	[V].LETGQTPEGKGFVEMLCKGLSQPTSNLVAGCLQ.[L]	3565.73352
6801	[L].PQSTGLGSPEAPHPVPGGGEGPPKTGTAPSPGPPCPPV.[D]	3565.73801
6802	[G].GPGSQGIQGPVSQGPLMGLNPRGMQGPPGPRENQGP.[A]	3565.73869
6803	[Q].AGRPGNPGHQGLAGVPGMPGTKGGPGDKGEPGRQGFPG.[V]	3565.74655
6804	[V].RGPDSAPFLLGLLTNELPLPGPAVGEASTSARAGYA.[H]	3565.86492
6805	[R].PASPARSGSPAPETTNESVPFAQHSSLDSRIEML.[L]	3566.718
6806	[V].PSLGGGGGCALPVSGAAQWAPVLDFAPPGASAYGSLGGP.[A]	3566.73728
6807	[V].PGAPGMPPGIPPLMPGVPPLMPGMPPVMPGMPPGLH.[H]	3566.75631
6808	[P].GPAQTPSLLPGLEVVTGSAHPAEAALEEGSLEEAAAP.[S]	3566.78606
6809	[L].GQPEPPSSKMPPSPGLLSTPAQDSPANSSRAPGPLD.[P]	3567.7384
6810	[V].ASPEPAGSSPLLEASLPAVSYQSQPSGTPDPPPAPPA.[P]	3567.74895
6811	[A].AAAVAAATSGVAGEGQPGPGENAAVEGTAPSPGRISPPTP.[A]	3567.76739
6812	[L].AQWPSQVFSGDPVLTTVAVLMLLLITGVTVIIW.[R]	3567.98991
6813	[T].SPPTQTPHSAPDPTVTPVGSSGDHLTPMAHPLDQP.[P]	3569.69654
6814	[Q].QPSASQVSPTEPRSSPSPPPSPMEPPEKSSQEPL.[S]	3569.70643
6815	[I].PEEAPGVHVVPIPEEAPGGHEVPIPEEAPGGHEIP.[N]	3569.7547
6816	[S].VPVAQMGNYQEYLKTLASPLREIDPDQPKRL.[H]	3569.87846
6817	[G].PYPSIPAAAADPSMVSAYMYPAGAAGAQAAPQGPAGPT.[T]	3572.68247
6818	[G].PGKSGSMGPAGPPGPAGERGHPGSPGPAGSPGLPGVPGSMG.[D]	3573.69616
6819	[P].ATPAVSTPEEPATPEEPATPAGAVSTPEQSATPAGAVS.[T]	3573.70787
6820	[A].GAVSTPEQSATPAGAVSTPEQSATPAGAVSIPEEPDAP.[A]	3574.70312
6821	[P].GPEGPRGLAGEVGNKGAKASGHGWVVTEATEIAVCL.[D]	3574.80709
6822	[F].GPSPGVEPVASMTSVASHPALGASSSSLPPLGPAAMNM.[V]	3575.71786
6823	[L].PSGEGHLEISASGVEDLSRLPSRGEDHLETSASGV.[G]	3575.72084
6824	[E].AKSAEEPAGRRDESGKGSWEARTLGGVPQFKQM.[L]	3576.7612
6825	[S].GPWSQAVMGRTRESVPSSGPTNVSVLATTSSSMLV.[R]	3576.77849
6826	[V].KSAGVTPAEPEELPTPMAQALPSPASTATPPPTPTH.[L]	3576.78904
6827	[Q].PGGYYALKDLGLEDTVEGIDAQVVNGYVIHDQE.[S]	3577.7333
6828	[N].KLTTFGTSTTSAPSFGTTSGGLFGNKPTLTLGTNTN.[T]	3577.80205
6829	[E].KNPQMGDPGSLQPKLAETMNNIDRLRMEIHK.[N]	3577.8036
6830	[P].GQAPQLPLSESSAPGPPHGPPPGLRPDAPGGGGGSVPGK.[P]	3577.81462
6831	[Q].KPGAMGVNIITSDFVDLVDFATTVIELNDLLQE.[D]	3577.83459

6832	[E].TPAAEGVGAAANAAATSSTGTGGVAASGMAASGVVPGGGFVAS.[A]	3578.70274
6833	[T].GVGPLLSGHDGGPGLPGYPSPGETAAGKERAQETTAF.[L]	3578.75101
6834	[S].QQPGPVSVGRRDHPGDPRASLMSLNGSGQLKMSS.[H]	3578.75507
6835	[R].KGGGSAASYRTPSKGAGAAFGSRSLYSLCRGDLCI.[P]	3578.75909
6836	[Q].RQTFAASHQLPGYAATPQPTGLSGVFDTSVKSAST.[N]	3578.7874
6837	[I].QRGGAPPLPGAMKNGQAAGPAPQTPSTSSLSQLGVYS.[D]	3578.802
6838	[L].GLAMSSSIFIGGSFILKKKGLLRLARKGSMRAGQ.[G]	3579.05018
6839	[P].ENVAGRHSFAFIPFSAGPRNCIGQQFAMNEVK.[V]	3579.73724
6840	[P].AVSSGVNVNILSGMGNGTIASSAALNSAASAAAGMTVGSV.[S]	3580.75815
6841	[P].KYSPTSPTYSPTTPKYSPTSPTYSPTSPVYTPT.[S]	3581.721
6842	[E].MAAWFITNVQVSGGGPGVSMVMKTLEDAENEAIL.[H]	3581.73245
6843	[K].SSPGQGLTARREHVYGMFRGGDRSGSLSSTAGGRS.[G]	3581.73745
6844	[T].TPTPPKMTTSAMPGPTPTSLPEAMLQTTTSPTPTP.[N]	3581.74235
6845	[P].QGPPGEQGPEGIGKPGAPGTPGQPGIPGMKGHSGAPGPAG.[L]	3581.75539
6846	[K].GEPGRRGDPGTKGSPGGDGPKGEKGDPGPEGPRGLAGE.[V]	3582.72799
6847	[A].SGPTETASSLPPTGSRRPDSSGPGAGAAPEPPASLPEP.[S]	3582.73067
6848	[S].VTGIMGHAVQTVEVVNEGDHSVREKLMHLFMS.[G]	3582.75017
6849	[T].AENMKNSVVISNPHATLSQQGNLESPSGSGVLSSGS.[S]	3583.72929
6850	[T].IEISNNNSTMVMTGMRIQIGTQAIERAPSYIE.[I]	3583.75531
6851	[I].MAMIALNTVVLMMKDVYFSSLALDDHLSFYI.[M]	3583.75952
6852	[L].NGIVDPAVMGGFAKYEKHAATNGRGGEGRVQPFH.[D]	3583.76114
6853	[W].PGTPGAPGPKGDPGFQGMPGIGGSPGITGAKGDMGLPGVP.[G]	3583.7672
6854	[L].RELPEPLMTFAQYGDFLRAVGPRCGPASLPHT.[L]	3583.79369
6855	[G].QGPSPQRPLSEASKPSGMKRSPSATVQSSLRCAT.[L]	3583.80677
6856	[E].DRAPESKSGSGSESEPSSRGGSLRRGGEAGGTGDGGPP.[P]	3585.65086
6857	[A].GPELGAFSQSPAPAMGGRAGLHCAQAYPVRTTGQE.[L]	3585.69616
6858	[P].ELDRAPGRGMSPQPSAVPEPSSSPDRSTGPHPTTA.[P]	3585.69866
6859	[Q].QPSASQVSPTEPRSSPSPPPSPMEPPEKSSQEPL.[S]	3585.70135
6860	[D].SVSSNLAAETVGHGGVMPMHGNGLELPVVMETDHI.[A]	3585.71345
6861	[V].FLMTFLDALETGYGKYKNPYHNQIHAADVTQ.[T]	3585.74711
6862	[L].HHRGNASGLGAGPGPSVGMGVVPDPFVGREVTSAKGD.[D]	3585.76154
6863	[G].AAGEGISAAPASPRSPKAGTSEGPVDSVPYLDRMPF.[L]	3585.76422
6864	[Q].GAGPLDYGPKGLPDPAEPVSYLNSGGKYVPSGPESL.[R]	3585.77477
6865	[Q].AAQQIQAPEIPEEHIPHQQIQAQLVAGQSLAGGQ.[Q]	3585.84083

6866	[P].GLPGTPGHAVEGPKGDRGPQGQPGLPGHPGPMGPPGLP.[G]	3586.79719
6867	[L].KAPDEDGETEGQDSSVDLGSEGDQKPGVGFFFKD.[E]	3587.59324
6868	[P].TPVASPMTPSAASFSFGSSGFKPTLESTPMPSVSAP.[N]	3587.69203
6869	[G].MALPASPAVWGQPGEGQPVCPLQAQVQGGGSAATSSP.[T]	3588.72098
6870	[P].RQCPPGRPYPHQDSIPSLEPGSHSKDGAHRGAP.[L]	3588.72615
6871	[K].KPEGPIQAMMVQSQSLGKGPGPRTDVGAPFGPQGH.[R]	3588.7686
6872	[E].AGLGGREAEARAEKVELGGDVGRGNAAAAPTSEAVVGP.[W]	3589.83172
6873	[A].ELWSAPGQAGFGPMLGGGSSPLPLPAGGSSSVGGSGGFGS.[L]	3590.68564
6874	[Q].PAAEAAAPGWAQARGHPGGELAAAASAAGDAGWPNKHT.[L]	3590.7272
6875	[R].SVSEDEKPKGVSSDSPRPVSEETGYKPVSSNSPK.[P]	3590.74565
6876	[P].ASPSSASKEVGIGFAQGPGASASTAATPGPAGLPRGYMA.[P]	3590.75438
6877	[P].GPLGGGGAGGPQMGLPPPPPALRPRLVFHTQLAHGSP.[T]	3591.91177
6878	[P].GPGPSPGAMLGPSPGPSPGSAHSIMGPSPGPPSAGHPIPT.[Q]	3592.73115
6879	[E].VFEVSGGPGHPTPPGHASVWSPAAGLSCPWGLGPVE.[P]	3593.72705
6880	[Q].GGPRPPGQHYWPGPEGAPQIPGPHASSVTHFPPPS.[S]	3593.74614
6881	[A].PGAPAASPFAAATALGGAEATSSTGPGTPEASAVSLVPEGA.[A]	3593.76058
6882	[G].KEPPGAMASATSQKPGGNQGRPDGSLGGTAPLIFPDS.[K]	3593.76528
6883	[P].FSTMRPASSQVPRVMSTQRVANTSTQTMGPRPA.[A]	3593.77336
6884	[I].PLQVPPEAVNMSLGLSMAATTNPFQLLACGPTVH.[H]	3593.78007
6885	[G].QDGHDRGAKTPREVAQQAVDADVHTVGVSTLAAGH.[K]	3593.78036
6886	[G].QPGLPGHPGPMGPPGLPGLDGLKGDKGNPGWPGTPGAP.[G]	3593.7958
6887	[L].CLPVVSSLADVYAGVDVQAAICLLANMAVDRSIS.[S]	3593.8012
6888	[Q].TSLGQERPVDQEVGDLARRSSVTSMESTVSSGTQ.[T]	3594.73002
6889	[G].PRGPEGAMGIPGMRGPPGPGPPGPVGDPGPIGFGPGYL.[S]	3594.76205
6890	[T].VEGGSSTTGPSSLESTPGCECPGDSPQSVSMDLPSV.[A]	3595.53566
6891	[P].IGPQGLPGMKGEPGLPGPPGEGKVGEPGVAGPTGPPGVPG.[S]	3595.85773
6892	[T].GPGPRCRGGGSGRASRPESRRMERSGRGPAGSGGGGG.[P]	3596.72388
6893	[F].AVGAQTLEGWDFLYSKYQSSLSSTEKNQIEFA.[L]	3597.73838
6894	[R].IRDPNQGGKDITEEIMSGARTASTPTPPQTGGGLE.[P]	3597.74494
6895	[R].RSLDTPQSLASLSSRSSLSSLSPPSSPLDTPFLPA.[S]	3598.8599
6896	[P].SVLPSPSAAAPASVETPLNSVLGDSSAPEPGLQAASQP.[A]	3599.80753
6897	[Q].GPLMGLNPRGMQGPPGPRENQGPAPQGMMLGHPPQ.[E]	3601.73956
6898	[P].ASASAPAPVPAPAPAPAPSPAPASSSDPAAAATAAPGQTPAS.[A]	3601.77689
6899	[Q].PMQLSQQQQGVQPTAPSPQAAQYPLPQASAPSEAA.[I]	3602.75438

6900	[M].GATDTVVTNVSGSVSSAGRPASASPAPNATADGSKTSRA.[S]	3602.7641
6901	[L].PVCVKELSVSGSCTVRNGSFSSNCPCANDANDD.[T]	3603.5203
6902	[T].GLDAGGLGPAGNAASTAGPFPFHLSQHMLASQGIPMP.[T]	3604.73115
6903	[T].YVLQSPGPSPETPSPPSDAHPRPQSSTLGAHNQAP.[R]	3604.74151
6904	[D].SPDSGLPPSPSPSHWALAAAGGGGGERTPAPGALEPDAA.[A]	3605.72553
6905	[S].SGQMGSPLALPSEHLGGGLGMGAASRELSSQASGSLAH.[P]	3605.7435
6906	[K].RCRGSCKKGKCESSRRGAAAAEPGPHAREELH.[H]	3605.74553
6907	[D].NAPSPSIGGSSRLDSTTPTQPMTPLHVVTQNGAEAS.[S]	3605.75003
6908	[C].QPMVSHLEALGAPRACSTSPLDLGGLCQVPMEAL.[G]	3605.75829
6909	[P].GPAGPKGDPGSRGPMGMRGPPGLQGPPGSPGQAGAVGIPG.[E]	3605.76999
6910	[K].PENLSVSPNRPTPSDLAIVMYTSGSTGRPKGVMM.[H]	3605.77605
6911	[P].GPAGPKGERGSKGDPGMTGPTGAAGLPGLHGPPGDKGNR.[G]	3605.79898
6912	[I].ATAQAQNQQQTEGVKTEESEPLPSCPGSPPLPDD.[L]	3606.65004
6913	[P].AGPPPTQSATAAANNNFVNFGVADLNAVQSPGIPQGN.[S]	3606.75716
6914	[S].LNQSISQMAGQSCVQLSVSQPVHPQTAANSQITP.[A]	3606.7639
6915	[L].SHGASQRAGITSPVEKREDPGAGTGSSLAAPELSGTQ.[D]	3606.77427
6916	[E].VPTADPTGVDRDDGPRIGVSYIFSNDDEDMEPQ.[P]	3607.61292
6917	[I].VGFGSGLSTDAGFNGGPSSSAGYGNGLNNAAGFGGGATSLG.[A]	3607.63202
6918	[S].PGAAYPEPSKTPHVVSSEPSPLAFTETPKTDLQE.[T]	3607.78025
6919	[E].RPCPAPYHVTEGTRGALRGTSTGNTQKPSEAPLT.[G]	3607.8034
6920	[A].EPGPPGPPGPPGPMGLQGMQGPKGLDGAKGEKGSSGER.[G]	3608.75842
6921	[Q].AARGPPESGSPKVGNWTPEDKRLSANHSHGQLDL.[L]	3608.79528
6922	[Q].GVLGGAGGGNGPGDPAVPGDAVSRGVPGGSGDQANPRGPSAA.[G]	3609.73889
6923	[L].RAQASGSTHSIPSLGHPEDSSLGASVPGKEEGGPGPVG.[A]	3609.7528
6924	[P].RGAEPAAAPTPSPTQQREPASSGDRPDPSSVEAPVP.[S]	3609.7528
6925	[D].VGGAAAAPGGGAGGSRELEMHTISSKVFGDILDFAYT.[S]	3609.76422
6926	[G].KQQEKPEGAETAAPTANGSIGDPSKEYVSLSHPSV.[R]	3609.76672
6927	[M].GSPGLQGFPGITPPSNISGSPGDVGAPGIFGLEGYRGP.[P]	3609.79724
6928	[P].GLGGPPGEPGLPGIPGPMGPPGAIGFPGPKGEGGVVGPQGP.[P]	3609.85225
6929	[P].GISSIEQGMTGLKIGGDLTAAVTKAVGTALSSSGMTSI.[A]	3609.87139
6930	[L].HLQAPEAEPQKVDVILEGLGSDLASMSQGKGHVPG.[T]	3610.81699
6931	[S].SGPPGPPGSIGPKGPEGLQGQKGERGPPGESVVGAPGAPG.[T]	3613.83575
6932	[G].GPADVGGAAAAPGGGAGGSRELEMHTISSKVFGDILDF.[A]	3614.75438
6933	[G].TESSLYAHGYLKGAKGPGITGTKGDPAGAGPETSLEP.[G]	3614.7973

6934	[C].PGAFLDVCRKEAPHTGPCLPRRCSGLGPGRPAQ.[D]	3614.80019
6935	[K].PGGRFLCLEFSQVNNPLVSRLYDVYSFQVIP.[V]	3614.84643
6936	[A].QGALGEPGTPGPQGESGAEGLQGSQGLSGPPGKKGEKGD.[E]	3616.74738
6937	[L].QSEAGKLESGASAPAPAAEAGLEKDGGSLSQTPGTPPGP.[T]	3617.75655
6938	[S].GEAAAPPSTSAAALSRPSGAATTTAAAAASAPAPGPASSPEA.[S]	3617.76779
6939	[H].QRPAPPSAAPSSHPPASSSISIPGMGSRTSGPHGLGSP.[L]	3617.78775
6940	[N].PGAGAAKMDKQEKVKLSFDMTASPKVLMSKPMLS.[G]	3621.88751
6941	[P].PGGSPGRSSPAGGSPGKPGSTPHVSGLGSPGRYSPANGGH.[L]	3622.74939
6942	[A].SPDPVPEPPPPSVEAPDKPTGSPDQPPSPAQSPAPR.[P]	3622.76599
6943	[G].QVGFAQGTQAGQLDPSQPQTPQQTQRGPKNVMPS.[L]	3622.76668
6944	[G].ISQDTTSLPTGATAPSETATDLTPGFGSAPVSMLTTV.[T]	3622.76803
6945	[P].QGIPGAPGAPGFSGPKGEPGDILTFPGMKGDKGDLGSP.[G]	3622.78462
6946	[L].LSEPIDGGAGGNGLVGPGGSGAGPGGGLTPTAPPYGAGKHAP.[P]	3622.78846
6947	[V].AGPTGPPGVPGSPGLTGPPGPPGPPGPPGAPGAFDETGIAGL.[H]	3622.81764
6948	[S].QPHPPGFGSISSSGALFSAGSQPAPPTFGTVSSSGQPP.[V]	3623.74011
6949	[R].QELNTRFLASQSADRGASLGPPPYLRTEFHQH.[Q]	3623.8102
6950	[A].QPTKTPAAPSGFSFPSPAVLGKPAEPPETSSAATSAAA.[P]	3623.82278
6951	[G].GVYIFGDGRGLIEGNDIYGNALAGIQIRTNSCPI.[V]	3623.82749
6952	[G].GVADLNNPRGPGAAGESDGTVGVIPQFPGAPQVPGPGGD.[A]	3624.76773
6953	[W].APDGSEDEPPKDSDGEDSETVAAGGQVPHPGQAPPGGA.[S]	3625.57971
6954	[P].AASTPAGPPSGGASPTPPAASPSGGSATRPSSGPTSEAPRP.[P]	3625.74772
6955	[G].QPGTGKTAIAMGMAQALGPDTPFTAIAGSEIFSLEM.[S]	3625.75867
6956	[T].PGAAGGATAASAAASVLGGSAAPATAGDTTKSENVAPADRSA.[T]	3625.76885
6957	[S].TPDPALGGTPRPGPSPGPGPSPGAMLGPSPGPSPGSAHSI.[M]	3625.77037
6958	[L].QHVGGGRRWMLVGAPWDGPSGDRRGDVYRCLV.[G]	3625.77642
6959	[I].GPPGIPGFPGAKGDAGTPGPPGPAGIAVKGLNGPTGPPGSPG.[P]	3625.87615
6960	[E].PAMGIPSAVVPGSMAGRMTTTVAPGSIAGGMAPSLPPGS.[M]	3626.76852
6961	[G].LNPMTPVHKGASPYGTPVTPRMNLNSNFGMATIP.[S]	3626.79164
6962	[P].PQPQPQPQPQPQPQPAAPEGPAQPRPEPSPWGPL.[D]	3626.81389
6963	[V].KPMNTTAPKVTNSTSGIAAIMSENLINEAGMKKY.[K]	3626.82266
6964	[K].SKPGSTGPEPPVPQASPGPPGPLSQTPPMQRPVEPQ.[E]	3626.82716
6965	[P].PGPSGVPPGMPGQPPGGPPKPWPEGPMANAAAPTSTPQ.[K]	3627.7359
6966	[V].KSAPPEAALECTQPPAPASEEEKVPVEPPEGEEK.[V]	3627.73706
6967	[L].GPAGNAASTAGPFPFHLSQHMLASQGIPMPTFGGLF.[P]	3627.75115

6968	[G].TDPPRPAAEAAAAAGGGGATAAAARGGEAAAEVTGWPAGAPG.[P]	3627.75347
6969	[L].SPADAAPDFPAGGPPPAAAAAAAASYGPDARPGQSPGRLE.[A]	3628.74151
6970	[L].QGAPAVNPSGSPAGPAGAAAACSPPRNDREPVEVVQF.[H]	3628.75612
6971	[A].LHKMGPGGGKAKTLGGAGCGGKGSVGSGSKRRLSSEDS.[S]	3628.83947
6972	[I].NSQPNINGKPSSDPIVGWGPSGGYVFQKGENITNA.[P]	3629.76191
6973	[E].FPGHTGPVNVVEFHPNEYLLASGSSDRTIRFW.[D]	3629.79243
6974	[I].SGPALMWTPQSVSREMELPTVLVMPTVSRETSP.[G]	3629.8012
6975	[G].GGLMAAALPVLGFAGTGIAANSVAASLMSWSAVANGGGVP.[A]	3629.84545
6976	[K].AAFQSQYKSHFVAASLSNQKAGSSAAGASGWTSAGSL.[N]	3630.75716
6977	[R].PSATTAQAENGPATLPAGTTPATSEAPKMSNADFAKL.[L]	3630.7592
6978	[R].PPPPRDSGGHSQSRSPGRQTQGALGEQKDLSNTTS.[K]	3630.76035
6979	[Q].GPAGPPGPPGPMGPPGLPGPMGIPGSPGHMGPPGPTGPKGT.[S]	3630.76542
6980	[L].PGRGEVWGAGYRSHREPGPGAKEEAAGVSGPAGGRGGG.[Y]	3630.76571
6981	[L].GLPSGGDGFLTGLATGTTLCQHANAVTEAARAMAAAR.[P]	3630.77514
6982	[M].QLAHHGPHGLGHPHAGPPGSGGQPPPRPPPGMPHPGP.[P]	3630.77846
6983	[T].APDGAALTPPSPSFAATGASSANRFVSIGPRDGNFLN.[I]	3630.79355
6984	[H].GPQGIQRHPGPHGPLGPQGPPGPQGNAGPQGHLGPQGP.[P]	3630.81735
6985	[G].ALNPFSGPAYPTGPSAASSSGPAAASGPLATSSPAYSPGL.[S]	3631.7551
6986	[R].PSDLTISINQMGSPGMGHLKSPTLSQVHSPMVTSP.[S]	3631.7917
6987	[R].ASAQGKPGNRNSNSYGVPEPAHAYAQPQTMAPPPAA.[G]	3632.7299
6988	[P].PATPTLATSAQPAAATPASVSSPAGSPGPPGSTASLSTASL.[T]	3632.82899
6989	[A].APAAAEGPAPGSIFLAGAAPPAPCPASSSILVNGSFLAAG.[S]	3632.84174
6990	[L].PGSLLRRPDLAAVLEVLGTYGPAAFYAGGNLTLEM.[V]	3632.91451
6991	[K].PGDVGPPGPQGPPGKPGPAGMKGEDGLPGSPGEKGEKGE.[T]	3634.74421
6992	[P].TGHLTDPFSRSSTFGGLGSLGSNAFGGLGSHTLTPSGG.[G]	3634.75208
6993	[D].SALASSMGEATNSKFLQGVGFNPFQERGNNIVTY.[E]	3634.75947
6994	[L].WLSMLPSGPGALWLETQMVGLGPCVLQQHFLH.[S]	3634.80075
6995	[V].APGVAIFATQFNMEVEIITSGMVISTFVSAPIMY.[V]	3634.82456
6996	[A].TTAGPAPGGPAQPPPPQASASDLQFSQLLGNLLGPAGPG.[T]	3634.85
6997	[P].GPRGPEGAMGIPGMRGPPGPGPPGPVGDPGPIGFGPGYL.[S]	3635.7886
6998	[G].GPAQELSALRAAQSLVHFQLRHGNDLLAMDAIHG.[C]	3636.88159
6999	[R].QPGTAQAQALGLAQLAAAVPTSRGMPGTVPPGQAHLAS.[S]	3636.89149
7000	[E].DHVGEVLPGELSYLSRVAALPAGSRPWPTGPSAHL.[G]	3636.89214
7001	[Q].QVEQQPSASQAGVQQPPATSTGGPAASAVSASVSTQVE.[P]	3637.75761

7002	[K].DGRTGQPGAVGPAGIRGSQGSQGPAGPPGPPGPPGPPGPSGG.[G]	3637.78544
7003	[D].MIPEIKPSAAILILPELRTETVVPNKDVSPVMH.[F]	3638.00597
7004	[S].GQPPRDPELETRMARPSTSGQNISGSATPRSEGR.[L]	3638.76881
7005	[N].QPSSAVHSSTVISTGAYGQVAHSMASKYQSSQGDIG.[V]	3639.69799
7006	[W].SGPWRSAPTQAGSSTTVCTGKTPASSAPMMNMKLQ.[E]	3639.70223
7007	[G].DVQAAAPSHTSSLPLTYSVASSVGTLQGAGPVSGPTTGA.[E]	3639.81367
7008	[P].PFHELGSPQAAMFQVGMYKVHPPMPSSLSAEAQA.[F]	3640.73854
7009	[E].SAPSAVSQPSSPHSPPSLGRSSEVSPVPTPSRGGADGGG.[G]	3640.75862
7010	[R].PRMTPQNRGSQEPRPEGAADGPAVPAAERRTEDP.[N]	3640.76333
7011	[W].INRSIQGSSTSSSASSTLSHGEVKGTSGSLADVFANT.[R]	3640.76851
7012	[T].PNVRMLTPEEPAAPAGAGSTPEELATPAGAVPTPEQS.[A]	3640.77993
7013	[V].SAQGPTVQAPMPTPRTVDDASQRPTPTLLNTAPEP.[A]	3641.8228
7014	[R].RQEAVGQQGPPPAEEKGPTAPAHGLRGAWPEAHSK.[T]	3641.832
7015	[P].PGRAGPPPPMGRGAPPPGMMGPPPGMRPPMGPPMGIP.[P]	3642.74451
7016	[A].GPFHAGQPAGAPLDCAAVAAGAHFRAGTGGGPVASQNSL.[I]	3642.76187
7017	[D].SRVASSSKGADSASVTMVVTPSVPGGGMTTMPVSTLSS.[N]	3642.76594
7018	[G].GPRPGQQQQQQQQQGGAGMAGGMAGHGQFQQPPGPT.[G]	3643.66257
7019	[Q].PAAAAAAAAVAAAAATATATATATVAALQETQNKDINQY.[G]	3643.85621
7020	[P].PAAAAAAAASYGPDARPGQSPGRLEALGGRLGRRKGSGP.[K]	3643.92763
7021	[V].AHLHMGKQAEEQQKFGEQVAYFQSALDKLNEA.[I]	3645.77545
7022	[V].PNSDPSRAVASPAGSRASSTRAARDGTEGARHPEAR.[P]	3645.79372
7023	[S].PFLGQHPFPLHSSSHRTCLNPGTHHPALTPAPH.[L]	3645.80328
7024	[L].AGPLGDPLGGDHLAAGGDVPPAPLAPAGPAPYSPPGPGPAP.[P]	3645.83362
7025	[S].GLDGKPGYPGEPGLNGPKGNPGLPGPKGDPGIAGSPGLPG.[P]	3645.86598
7026	[V].RGVQDSSALPSAVHRDAPVTSGPARAPPPDAPTSLAP.[P]	3645.87319
7027	[P].GTASAQPPPPPPPPPAPKESPFSIKNLLNGDHHRP.[P]	3645.89247
7028	[R].LHAGSKDSTMPRTGRSPGRSAGASPTNPGPTQSAGASP.[K]	3646.77389
7029	[E].CWDPRTRGRVGVLDCALSSVTAESEINSLPTIS.[A]	3646.7952
7030	[V].QRFAQERGHDASQITLALGTAASYPRACQALGAM.[L]	3646.79654
7031	[A].YPQGVHPAFLGAQYPYSVTPPSLAATAVSFPVPSM.[A]	3647.8243
7032	[G].RPGPPGPPGPPGENGFPGQMGLRGLPGMKGPPGALGVM.[Q]	3647.83959
7033	[L].AGRGAAGDGPAAALLQAAGVAADWAAAGLADGARAAGHAGHG.[P]	3648.81266
7034	[T].QPGLPSSGQGGAASPGSSLGLYSPIEPGVVASGGQGPLSQ.[K]	3648.81401
7035	[I].RGYPGMAGPKGETGPQGYKGMVGSIGAAGSPGEEGPRG.[P]	3649.7122

7036	[L].NAAEQRGAREAAGSASRSGPGGSGSSGRGGAGVPGPGSGGPGG.[S]	3649.71586
7037	[A].RGHCTTLISSGGYVSSDYVGRANLTNFPESGTFV.[V]	3649.73398
7038	[T].PGMQGPPGPRGMQGPPHPHGIQGGPGSQGIQGPVSQGP.[L]	3649.74993
7039	[Q].TPPPGPSADVLSQDPRGSAASVAHQEPPSSVLDMVH.[G]	3649.75511
7040	[P].GPQGPPGKPGPAGMKGEDGLPGSPGEKGEKGETGQPGPP.[G]	3649.75511
7041	[R].VGQTIERMGSGVERMGPAIERMGLGMERMVPAGM.[G]	3649.7562
7042	[P].EPPDREELSQAEATKPEGKEPEGRAPPSPSPVHT.[C]	3649.77287
7043	[A].AAAPTAAPGPAQPGHVSPTPATTSPGEKGEAGTPVAAGTTA.[A]	3649.80926
7044	[M].GVAGPASLYHSGLTVGMISGGGVVCVQEARAGYVGSLG.[C]	3649.81013
7045	[N].ASPSLPESLSSLAATPVGGSSPGSVDVASSPGVERPGSGV.[S]	3649.81915
7046	[A].SQVTTVGSGKASEPEVPDKHSSASYVSSLLKSSVNS.[H]	3649.81915
7047	[S].PAGNQVQAGKQSHIPYSQQRPSGPGPVTQGPQQPQ.[P]	3649.82183
7048	[P].GQPGPKGDPGVSGIPGAPGLPGPKGSTGGMGLPGMPGPKGV.[A]	3649.84651
7049	[P].GPQGPVGPTGKHGNRGEPGPAGAVGPAGAVGPRGPSGPQGI.[R]	3649.86945
7050	[L].PGSPGEKGEKGETGQPGPPGLDGPTGEKGEPGDPGRPGA.[T]	3650.73173
7051	[P].QLSSPMYFFLSHLSFVDVWFSSNVTPKMLEN.[L]	3650.76982
7052	[P].GPMGPPGLPGPMGIPGSPGHMGPPGPTGPKGTSGHPGEKG.[E]	3652.74575
7053	[T].GAEEAAVAPGAFERAHPSPRANADPGPTGGTAPDSPRA.[F]	3652.74872
7054	[Q].STIREHRDGGNAGGIFNRYNVIRIQKVVNKKL.[R]	3653.02589
7055	[P].IGTPGEKGPPGNPGIPGLPGSEGPPGPAGSAGPPGYPGPRG.[V]	3654.82993
7056	[V].VGAGMAAAALAAEAGMVAAGAAVGATGAAVVGGGVGAGLAATVGC.[M]	3654.84005
7057	[P].PGEGKVGEPGVAGPTGPPGVPGSPGLTGPPGPPGPPGPPGAPG.[A]	3654.85508
7058	[A].AEPPQPQPQPQPQPQPQPAAPEGPAQPRPEPSPW.[G]	3656.78807
7059	[G].AAHPPGTPFGPPPHHSNFLNPAAHLEPFNRPSTF.[T]	3656.79343
7060	[N].TKLLKALRVRKKTGGEKMPVHMIGDILAAELSH.[M]	3657.08187
7061	[A].LGGPAGAEGPMAKKHAGERDKKLAAKKKTDKKRAL.[R]	3657.08571
7062	[R].TSFTSVSRSGGGGGGGFGRVSLGGAYGAGGFGSRSLYNL.[G]	3657.77929
7063	[P].QPSTPWLAASRDGLETELPGVGPECVPQTAPAAASP.[G]	3657.78535
7064	[G].QQNAVTATRPEASKMSYTLDSLGNPSAYRRVTE.[T]	3657.79256
7065	[S].SPDSPIASATKGIPGFGSTGNLSSAPVTYPSAAAPGVNN.[T]	3657.80311
7066	[Y].FVAFDGDLVQEFDVGSATHVLGGGDRNPEAQQVSP.[E]	3658.74084
7067	[L].GPASDTGILNPEGYTLNYNEFIVYNPNQVRMR.[Y]	3658.75947
7068	[I].KEEPVSDITFPVSEELEADLASGDQSLPMGVLGAQ.[S]	3658.76803
7069	[Q].SPAPAMGGRAGLHCAQAYPVRTTGQELPFAYSGQP.[G]	3659.74819

7070	[I].VGWMPLMAAQKDFFWEALDMLQRAAGGAGQGPPP.[P]	3659.75961
7071	[P].PGGQGPPGSSGPPGVKGEKGFPGFPGLDMPGPKGDKGSQ.[G]	3659.7911
7072	[E].FGYIPQQVEPVMPGGQQQPAFDPFLGTAPEIAVM.[P]	3659.79129
7073	[S].VPMSQAALGEIVPPGEDQVGHPSPTVHQDFVREH.[H]	3660.78635
7074	[P].GPVGEAEKGVLAQGQEDGAVSKGGRGPSSRHAKEAED.[K]	3660.79607
7075	[T].APPTVCVTGPPTARPSEGPTTGPTGPPAAGPTGPPTAGPS.[A]	3660.79625
7076	[G].AGMAAAALAAEAGMVAAGAAVGATGAAVVGGGVGAGLAATVGCM.[E]	3661.78048
7077	[L].RRDMGLWSAVSLTAGSMIGSGIFMAPQGVLVYMGS.[P]	3661.79976
7078	[W].KAASAQGWWPVWPHASASGACRRRTGGSAWRPR.[W]	3661.8319
7079	[M].FLNYIMELLKELQSATVNDMSPYIKVAPGEFT.[I]	3661.85322
7080	[Q].DGKDLVPQESTVVPHGDAPGLWGGPEQIPTPPTSPS.[S]	3662.7973
7081	[P].GEPGLRGPEGSRGLPGEEGPRGPPGPRGVQGEQGATGL.[P]	3662.83821
7082	[Q].GFGAGTDPPRPAAEAAAAAGGGGATAAAARGGEAAAEVTGWP.[A]	3663.75347
7083	[G].HLLASVAGSGGGGGGGGPGTATGLDAGGLGPAGNAASTAGPFPF.[H]	3663.77863
7084	[I].PGGVADLNNPRGPGAAGESDGTVGVIPQFPGAPQVPGPGG.[D]	3663.81501
7085	[P].GPSVSAPSVSTSSSSIGLTSAGGAGDQAEGTICSSGAGVPN.[H]	3664.68788
7086	[V].RSSEQVASASLGPAGSLGQEGLVETVLAMEPGAMRF.[I]	3664.79454
7087	[L].HQTTAAAAAAASAASAVGPVHNSVPSNPVAAPGFFVHPS.[D]	3664.82552
7088	[A].QAQAQAQAQAQASQASQQPQQQPQPPHFQSPGAA.[P]	3666.73922
7089	[K].IWCSTIKESESTGINGVAFVSFAGMESVLDERF.[L]	3666.74546
7090	[S].NSTSLNSTFGSTNLSGLFFPPQLNGTANDTAGPELP.[D]	3666.75582
7091	[V].QNIGQQPTQGSPQPVGQQANNSPPVAQASVGQQTQP.[L]	3666.7855
7092	[G].VPSLGPSEAMHGLPEGQPPRPGGPFAPEQDTGAKNK.[T]	3666.79692
7093	[P].YVNLPHQASAPAWWPDPSRHIFAVVASAYSLSE.[S]	3666.81283
7094	[G].PQGLPGMKGEPGLPGPPGEGKVGEPGVAGPTGPPGVPGSPG.[L]	3666.85846
7095	[G].KRVAMVGDGVNDSPALAQADVGIAIGTGTDVAIEAADV.[V]	3666.86433
7096	[S].VRALQEMLANTVEAGVEALNLDKWSSQGGGHRTL.[L]	3666.86567
7097	[D].VGVGIAGEIGLPGPPGPQGPPGYGKMGATGPMGQQGIPGI.[P]	3666.87708
7098	[A].LGATCAAAFPSAASVTSAGATSASSVHLPVSAPHGAGLMA.[A]	3667.78431
7099	[L].LMLEQLLMNMKVDWATNDLSTFSFPADSVTHL.[Q]	3667.78449
7100	[E].HPVGRWGGAGNPAPTRQCPSPGVLPPSLPAMAHAAR.[L]	3667.85975
7101	[Q].PAAGGPSRAAGSPAPPRAPPVPDYVTHPERWTRYS.[L]	3668.84692
7102	[D].GARAAGHAGHGPAHGGLAGHGAAAAGVAVETGLEAASATAQGG.[D]	3669.79774
7103	[P].SRGEDHLETSASGVGDLSGLPSGREGLEISASGAGDLS.[G]	3670.74269

7104	[G].TPGTPGSHGLPGRDGRDGIKGDPGPPGPMGPPGGMPGLP.[G]	3672.76457
7105	[G].RGDASSPAPATTLAQPQQNQTQTHHTTQQTFLNP.[A]	3672.77494
7106	[P].SPSAAAPASVETPLNSVLGDSSAPEPGLQAASQPAETPA.[Q]	3672.78752
7107	[F].PGSPGEKGEKGSTGIPGMPGSPGPKGSPGSVGYPGSPGLPG.[E]	3672.79625
7108	[P].YRYPTPDGPSRFPRVAGPRGSGPPMRLVEPVGAH.[E]	3673.8921
7109	[D].DASLVPQGPPKVKRRVRIPDKPNYSLNLWSIM.[K]	3675.03155
7110	[A].KSQAEESASGKAEKKTSGEAKNQVNGTRTNKSNNP.[H]	3675.82809
7111	[P].GSPGQGEFSHVDLAVLFSDPPADGSAAPGAPDEALGAGI.[L]	3676.74017
7112	[A].AAPATDPWGAPVSMAAALPTAAPASDPWGGPPVPQAADP.[W]	3676.77406
7113	[C].QQPEKIECRAENYPEVSIDQIGQVLSCSLETG.[L]	3677.74217
7114	[P].GSPQVSGPSPATRMPGMSPANPSLHSPVPDASHSPRA.[G]	3677.75474
7115	[G].SPQVSGPSPATRMPGMSPANPSLHSPVPDASHSPRAG.[T]	3677.75474
7116	[A].GHHGDQGAPGAVGPAGPRGPAGPSGPAGKDGRTGQPGAVGPA.[G]	3677.80282
7117	[P].KLMTPDAFMTPSASLQQIAASPSSSSSSSSSLTAVS.[A]	3678.73608
7118	[I].GPKGPEGLQGQKGERGPPGESVVGAPGAPGTPGERGEQG.[R]	3678.82189
7119	[L].PCRCCFCGEDHPQWGSLPCHVPPGAKPTDWA.[R]	3679.55446
7120	[G].PGDPAVPGDAVSRGVPGGSGDQANPRGPSAAGESGGAAGAIP.[Q]	3680.76477
7121	[L].SAAGDRSQRGGNQWDARALSPPHPAPRNCPAFVH.[E]	3680.77483
7122	[Q].GPDTATTAAPHTEGRLAGQGPQGQQSMLPGSPPSTPSP.[P]	3681.75618
7123	[P].RQHYPYGGPYDRVRTEPGLGPEGNMGTGAPQPNL.[M]	3681.76154
7124	[Y].LPDTCGEPLTIRQLGGTPGQPGRMWELGVASAGED.[V]	3681.76357
7125	[E].ANESEEVRRFRQQFVQLAGPDMQVGATDLMNI.[I]	3681.7748
7126	[L].SQTPSSAVTPVLNESSTSPTTANHSRCVAPGISLNN.[S]	3681.7773
7127	[Q].QRARPSTTSSGPSQGPSGSTPRPSTTSSQGPALGQSPS.[G]	3681.78114
7128	[Q].GHIGPQGPPGPQGHLGPQGPPGTPGMQGPPGPRGMQGPP.[H]	3681.7914
7129	[N].SFRGYPSEIQMMTLPPGQFVITDSGVATPVTPGQG.[H]	3681.79274
7130	[A].VGGAAAQHSWSHISAALQDRRFRCQLIDSSEDL.[G]	3681.7939
7131	[L].AQPGPYEASKQPPPQPALPYNHIYQYPYPSPVS.[P]	3681.80126
7132	[A].VGQEQAFSVNTRGAGGQGHLDVRMTSPSRRPIPC.[K]	3681.8085
7133	[F].APNSQRSGYGAAAGAFPSTVPGLYNVNSPLYQNPFA.[S]	3683.78773
7134	[L].YFQVLFLTAQFEAAIAFLFRMERLRCHAVH.[V]	3684.90826
7135	[T].HTAEGADPLPQPSGTPTPTQPSEAMAVTDSIRGEAPG.[A]	3686.72387
7136	[Y].QSHQPLPQAASQPAPGAPHLQPMQRPSTLPASAAGY.[Q]	3687.84487
7137	[C].SPMELVSVRGESPQSLGATNSSPTPVGRGAQVGPGQGP.[V]	3688.83476

7138	[Q].QMRSLNPLGNNPMNIPAGGITTDQQPPNLISESAL.[P]	3688.84215
7139	[L].NAAPPPEPPGPLGAGAAGAPAGGAPERQSVIQFSPPFPN.[S]	3688.85067
7140	[A].GPRPPGSGPGPGPATGAKTEPGSGPRAQARTGGTTSPKHG.[R]	3688.86509
7141	[P].PYASPTAPSPSSPVPTPSPSTPAPPTSSPSSPAPPNPSP.[S]	3690.78098
7142	[R].TNPRTLMSTPFTHGRATPSHTPTDALHAHGTRAH.[L]	3690.80547
7143	[S].GPGAGGEATLLAPSAEAGGGLTCAPAPQGSLRIFEGPQE.[S]	3691.80206
7144	[T].TAAAAASAPAPGPASSPEASPAPGFPFPPPWMGMPLPPP.[F]	3692.79162
7145	[A].LSSSVPSSSTPSGPHTTATPSVTASALGPSTPRSATSHS.[I]	3692.79981
7146	[T].EIHGGAGGPSGGRPEPGRAAASGAAASSADPTALGGPAGAEGP.[M]	3693.76002
7147	[P].TSAPPGTPTQQPSTPQTPQPPAQPQPSPVSMSPAGFP.[R]	3693.78535
7148	[P].PTTTAAEPQPTAPPTVCVTGPPTARPSEGPTTGPTGPP.[A]	3693.80648
7149	[L].PGQSFGGPPVSQPNHVSSPPPQALPPGTQMTGPPGPPP.[P]	3693.81184
7150	[S].PGAKGEQGPAGHPGEAGLPGPSGNMGPQGPKGIPGNPGLPG.[P]	3693.81905
7151	[P].PTQTPHSAPDPTVTPVGSSGDHLTPMAHPLDQPPPD.[H]	3694.74421
7152	[M].RGMQGPPPQGSMLGPPQELRGPPGSQGQQGPPQGSLG.[P]	3694.78129
7153	[Y].KNYFTAGAHWLTMVFLILVNITAQVAYALQDW.[W]	3697.92396
7154	[T].EGRPGYVEISTFRNIEDVRSTMATFLLLRIPT.[L]	3697.93704
7155	[T].DPRGSRGGGGGPQPEGPSARQFLARLEARPLAARAAA.[D]	3697.94943
7156	[Q].PQQVQMQVQPQQANAGVGQPASGESSLIKQLLLPK.[R]	3697.9694
7157	[A].AAQPSTPAGTPRSGGGHSPAQPPSPERGMEERAGMRA.[S]	3699.7463
7158	[T].NTTGLTPSTGMTTISEVPYPDETSLHATNVSQPVGP.[T]	3699.76943
7159	[T].LATGMFLSMSQVSPGRSGSVGQRSVASALGPSQSQMS.[R]	3699.78874
7160	[G].PAAAVAEPVPGQSDTATGLGPCLPPAAASPSEAGSTGPSR.[P]	3699.79189
7161	[P].GRTSPAVMQPPPGMSLPPADIGPPPYEPPGHPTPQP.[G]	3699.79341
7162	[A].GAPLDCAAVAAGAHFRAGTGGGPVASQNSLIQTVDYLS.[G]	3699.81838
7163	[R].HSNVSQASRASRVLPILPVNGKMHSAVDCNGVVSL.[V]	3699.91699
7164	[Q].AAAAGQAAQGKTTLPSQGPVQRPSRLVFTDVANAIHA.[-]	3699.96776
7165	[K].GPEGLQGQKGERGPPGESVVGAPGAPGTPGERGEQGRPG.[P]	3706.82804
7166	[D].GQISTEVSEVPMANDKPKTLVVKVQKKAADLPDR.[D]	3708.00003
7167	[S].QVGQYSQPEVPVRSPMQFHQNFSPISNPSPAASV.[V]	3710.802
7168	[S].APEPGLSPAQPTQAQGQPDPVPSLTSIQVLENSMPI.[T]	3710.85818
7169	[P].GQQVHTPQSMPPPPQPSPQPGPPSSQPNSNVSSGPAP.[S]	3711.74561
7170	[I].KDTQNVAPLNKMEDGELECAPEDAVVVQPAEAET.[P]	3713.71568
7171	[W].KGTSGFGRSQTMLGEDSAAGDSKYQNLNLEESAPAP.[F]	3713.73477

7172	[H].QETHTREKSPTPEDPLPEPATLSTSQEGEGEAPAP.[T]	3713.7413
7173	[W].NCLVAAAAPPGASPLFGYELSSGTKETAFIYAVMAAG.[L]	3718.81315
7174	[P].RQSNWASAVGRQALQMTSSPLSPRKSFHFEVSL.[L]	3718.88707
7175	[G].PGPSPGPAPPNYSRPHGMGGPNMPPPGPSGVPPGMPGQP.[P]	3721.74609
7176	[I].GPDSSARAPGESSAISMGIVSVSSPDVSSVSELTNKEA.[A]	3721.77088
7177	[P].QPEPEPAAGEPGRAATAPTAGGEPLSPPPPQEPAPGAPQ.[Q]	3721.80926
7178	[G].SRSFSTASAITPSVSRTSFTSVSRSGGGGGGGFGRVSLG.[G]	3721.86409
7179	[P].GASGWSHNPTGGPGPHWGHPMGGPPQAWGHPMQGGPQ.[P]	3722.63014
7180	[G].PPAASTPAGPPSGGASPTPPAASPSGGSATRPSSGPTSEAPR.[P]	3722.80048
7181	[M].KDPHMSKTAPPSGARSHPGSSQPSGAAPGSSGPGALPPY.[A]	3723.79323
7182	[A].GWSYERSAKASLVYGSSRTSHPETDILHRQAYA.[A]	3723.82624
7183	[E].PAMGIPSAVVPGSMAGRMTTTVAPGSIAGGMAPSLPPGSM.[I]	3725.81918
7184	[M].APGGGDLKAYLRGGEPEPARGDAQTMLLQRLACEI.[A]	3726.86904
7185	[-].MAPGGGDLKAYLRGGEPEPARGDAQTMLLQRLACEI.[A]	3726.86904
7186	[P].PAAAPPSAVGSPAAAPRQPGLMAQMATTAAGVAVGSAVGHT.[L]	3726.86904
7187	[I].SGGLALGPGYDAPGLHSPLSHPSLQSSLSNPNLQASLS.[S]	3726.87219
7188	[L].LFSDRMVQGSKGPPQMGLSALTWAGSQRAGLASNGH.[A]	3728.83841
7189	[P].RRGSGGGSASALGAAGTGVGSSAPSAEDFPPPSLLQPPPPA.[A]	3728.86269
7190	[L].RAGAFDDLTELTYLYLDHNKVTELPRGLLSPLV.[N]	3729.98504
7191	[A].PSPLGGSALCGGKPEAGESPPPAPGTPKANGSQPPGAGSPP.[P]	3730.81296
7192	[G].PPYSPLDASIFPQSESRMKNSRSGVDSGIGESVHV.[S]	3730.81296
7193	[L].GTGLGTGLGFGGFNTQQQQQQQTTLGGLFSQPTHAPA.[Q]	3730.82082
7194	[L].WRHVDKGSAEGVVDAMLLCGQDAITVSNGGRIMR.[S]	3730.82104
7195	[L].PGANGPKGEKGESASDKLQESLAQIIAEPGPPGPPGPPG.[P]	3731.88751
7196	[P].GPSPGPGPSPGAMLGPSPGPSPGSAHSIMGPSPGPPSAGHPI.[P]	3732.78972
7197	[Q].QIVSTTHTSSYTLPTCPAGFQTSVQGLGHVPTGVGM.[S]	3732.79962
7198	[C].PGSGVTSTSWIRGAWAPGYPAGPGLAQPSSHREAGGGR.[G]	3732.83781
7199	[V].TGTGPNFSLGELQGHLAYDLNPASTGMRRTLPSTSS.[S]	3733.82386
7200	[A].ASTAAAAAATTTSAAATSAPPALDMFGDLFESAPEVAPAP.[K]	3734.77418
7201	[R].AQGPVSGTMLETLGTNRTDPVCTALARLAEGDTCR.[S]	3734.78947
7202	[E].VGEAASHLASYALGSSSANVEALPQESLDRMMANLL.[K]	3734.80001
7203	[H].APSSAAFGFPRGAGPSHPPAPPAAPEPLGGICEHETSI.[D]	3734.802
7204	[V].PSLGGGGGCALPVSGAAQWAPVLDFAPPGASAYGSLGGPAP.[P]	3734.82716
7205	[P].PAPLGPGMAFGSPAFPPHTVMLHAGPPYTPQRPATH.[F]	3734.83589

7206	[P].QEQVSGGQLQEAQVTCPPQRSSAAWDMTFLEQL.[C]	3735.73775
7207	[M].GANSLERMGPAMGPALGAGIERMGLAMGGGGGASFDRAI.[E]	3735.78221
7208	[P].AAAEGPAPGSIFLAGAAPPAPCPASSSILVNGSFLAAGSSP.[A]	3735.86869
7209	[S].DALTPPPLPPSNNLPGPPGPSGPATQPPVSSATMHLPL.[V]	3735.90507
7210	[I].QVPQQKAMAPIHAHPAGMRINVVNNHQAKQNLY.[D]	3735.9071
7211	[C].FEFNLSFQQSYGIYKIAHEDYCDDDENSAAC.[H]	3736.54788
7212	[T].VASQDGGSVVTFTTPVQINQYGIVQIPNSGANSQFL.[N]	3736.88169
7213	[P].VEARTAAEQIPAHPLVTESMETVPPPEKVQPGPSQ.[P]	3736.88506
7214	[F].PGLRTARDQTPSTPEGFRAAPQVGDRGPAPPAPGQSP.[G]	3736.89024
7215	[K].AVQTSRAPAKDPGALNAQPKPGPLSWDPASVTPEPVS.[S]	3736.92931
7216	[D].RLGQEAYLSLLSDWDLSTAFATASKPYLHLRVD.[I]	3736.93334
7217	[E].GAENKTGDKAGSHRGLGSSSGDGSRSPSKPAGQALEEGV.[R]	3738.80261
7218	[P].NGERPLSSTGPSQHLQAAGSGIQNQNGHPTLPSNSVT.[Q]	3738.81786
7219	[G].LDNTGVSTLDTLEALSVSEDPQTSDNSGVAILRAYS.[P]	3738.81921
7220	[S].AGPSASASAGPPASARPGTSAAARAATSASARADMSATARPG.[P]	3738.83247
7221	[V].GARSAAPGGGSVAAASAAMGAALASMAGLMTYGRRQFEH.[L]	3739.78499
7222	[A].SPAMLASVESGGPPPPTASQSASVSIPGSLPSSTPYTMP.[P]	3740.80337
7223	[S].AVDASPRNASPGLPNGEKEDRFLTTLSSQSSTSSPH.[L]	3740.81105
7224	[C].GQLSTGPHRGHLHPGAGGRERCASPLGAGAAGSQGPAGGP.[A]	3740.82833
7225	[G].VGAAGGSLSFGASSTPAQGFVGVGPFGSAAPSFSIGAGSKTP.[G]	3740.85548
7226	[C].GDPAPKEAVWEALSRMGVYVGREHCVFGEPREL.[L]	3741.82644
7227	[N].KAVPSQSTFPSKTGGMEGGTAVATSSSLTADNDFKPLG.[I]	3741.82761
7228	[P].GRAGEKGDVGSQGVRGPQGITGPKGGPPGIDGKDGTPGMP.[G]	3741.87254
7229	[S].KQMYTGIPIIAANMDTVGTFEMAKVLCKFSLFT.[A]	3741.87628
7230	[S].PDADVPHADAAAPPKAPVCSNGPPAELQSGAPLPGELE.[E]	3742.80173
7231	[E].ANLQGDPCRFALTSRGPEGGIQRYVLQAADPAVSQ.[A]	3742.87181
7232	[A].GPLGDPLGGDHLAAGGDVPPAPLAPAGPAPYSPPGPGPAPPA.[A]	3742.88638
7233	[C].VGLGHISGKYASHRSASGCPLAARRQKEGSLNGSSF.[S]	3742.89428
7234	[P].HYTPEISTKLTLINFTLSPSGLEDQLLGQVVAEE.[R]	3742.94256
7235	[G].AADKPRDVSSVEVLMNYHQGLKTELEARMPELT.[A]	3743.87312
7236	[M].SYKPIAPAPSSTPGSSTPGPGTPVPTAGSVPSPSGSVPGAAA.[P]	3743.87627
7237	[-].MSYKPIAPAPSSTPGSSTPGPGTPVPTAGSVPSPSGSVPGAAA.[P]	3743.87627
7238	[I].FNAPSLQDRLRFTSDLRESIAEVQEMEKYRV.[E]	3743.88098
7239	[A].ARMIYSTAGLYNHFIKGLDSFSGKPRGSGSPAGTAL.[P]	3743.89624

7240	[T].KKTYSTDEKVEEEVIPLADVSTSNLDSKGLALYT.[A]	3743.91132
7241	[K].KEPPAGDLAPALTEDGPPTVAPGPMQAPLPLAPMAGRP.[G]	3743.91353
7242	[P].GPQGHIGPQGPPGPQGHLGPQGPPGTPGMQGPPGPRGMQ.[G]	3744.78704
7243	[A].GPPGGSRKCPPGSPTDPNATLSKDEAAVHQDGKPRY.[S]	3744.81469
7244	[V].SPRFERCGWASQRPSARTPACAPRDLPGPQAPAP.[P]	3744.83466
7245	[-].MTPFALTWMVDMIADVLTGRTAQGIASTMEKLSTM.[V]	3744.83554
7246	[S].SPSPSAVSTSHSIPTVSSAPHSPSPSAPLTVSQTASLST.[S]	3744.85627
7247	[G].GGGERTPAPGALEPDAAATRAAPNPASLPNTLGSGYSPR.[L]	3744.86884
7248	[Q].FRGTPDEVGSLRREPSWAEGSAAVPVDEIMLLYP.[S]	3744.86902
7249	[V].GTPPSAHGAHSVGHSLQSPVPSASQRERQALQDLLE.[L]	3744.88007
7250	[A].IFPGFGGMRPSLGGMPQNPDMGGDFTLEFDSPVAAT.[K]	3745.69713
7251	[H].HVHRLNMSGPFGGAVSAAGLTQMPAGNVFTTAEGLFS.[T]	3745.82136
7252	[C].PSPGALSNASAPVDFFLNGRVYADEAAVAEELLEPE.[E]	3745.82318
7253	[L].QPVGWEMNFVPVYVNDTVLLGGFSDKHISPQSAN.[I]	3745.83191
7254	[G].FPINCILRYSYPFFGSAAPIMTNPPVEVRKNM.[E]	3745.86916
7255	[E].TISDEIHERRRSGAMGPRGRGVGAEGGAVPAEGLAGR.[K]	3745.90116
7256	[A].PAPPQGAPQPGLSGLSPAGPELGAFSQSPAPAMGGRAGLH.[C]	3746.87075
7257	[Q].ANPAYQASSDMNGWAQGSMGANSMFSQQSPPHFAQ.[Q]	3748.54857
7258	[S].IGNGNSQIEENGKLTLTPNYSSSLNITQSQGHFLS.[P]	3748.84129
7259	[F].WRPGTEGPGVSISEERQSLAENSATTVVYNPYAAL.[S]	3749.84056
7260	[W].QQSATQPAGSLSVVTTVWGVGNAAQSQVLGNPMGPAGSP.[P]	3749.85516
7261	[P].PGLPPPPPPGMLMPPMPGPGPGPGPGPGPGPGPGHSMRLP.[V]	3749.85755
7262	[I].QGGPGSQGIQGPVSQGPLMGLNPRGMQGPPGPRENQGP.[A]	3750.81873
7263	[C].GANQRQDARGTSCVCLPGFQMISNNGGPDVICKK.[C]	3751.75198
7264	[L].PASEEPGTMIEWGNNWARAIKYRQENQEAVGGF.[F]	3751.75578
7265	[F].GVQPCGPHDPTQGGMMPHPQSRGPLPTCQLKSELG.[V]	3751.756
7266	[Y].LRPQQEQNQFPSRMKETHGVLPEELSSRERE.[E]	3751.85689
7267	[R].RPGAAGPAPHPQWAGQPSVLDSINPDRHFTVNKSF.[L]	3751.88403
7268	[G].TVPSENLEPEVESSPELAPLPPPACPSESPMPVAPT.[A]	3752.79213
7269	[Q].GGGPGGAGTPPRTGAGLPLPTHGGGFGSGCGRPAPPAASSPY.[P]	3755.80955
7270	[T].VTMNGVAGRNHGINAHAATTQYANGVVPSGQTANAVAH.[R]	3756.83716
7271	[A].TPKCQSLGGPAAAYATGKASGAGGAGGQAYSPGQPQGLLGP.[Q]	3756.83985
7272	[A].SQTPASNQSKRPPAAPENTPSVPSGVKSWAQASVTHG.[A]	3756.86884
7273	[S].PAGSPGPPGSTASLSTASLTPSSPRVPNVSAQGPTVQAPM.[P]	3756.88613

7274	[E].KGEDGFPGFKGDMGVKGDRGEVGVPGSRGEDGPEGPKG.[R]	3758.78272
7275	[V].QQPSSHQPHSVAHVVGPVHAVTPGSHIHSQTAGHHL.[P]	3758.87593
7276	[K].APPAPADPPLMAGASPVHFAAAGTVEPKAGSSKNAPNPPA.[S]	3758.8959
7277	[P].RSTSRPAAEPAGARGGEAAGLKGARSVEGPAGLEPGLEE.[Y]	3758.91685
7278	[P].GPGANGMPLAGLAWSSASAPPPRGFSAISCTVEGAPASF.[G]	3759.78939
7279	[P].LCPVGPGPGGPGPAGPMGPFNPGPFNQGPPGAPPHAGGPPP.[H]	3759.79475
7280	[S].GNSVSNSLPSYLFGMENSHSPYPSPRHSSARSHSA.[R]	3760.71571
7281	[F].GIQNNCSQLLTSGPGTLPDQLMAISPPGQPQNEGQP.[P]	3760.79051
7282	[R].RGSKGHGPGMARKFSAPGQLCVSMTSNLGGSAPGSAASA.[T]	3760.80645
7283	[E].MTITSQTGLPEATSQGTLTLNSVTEASGAGTHPAVTQS.[F]	3760.81817
7284	[T].ATGLDAGGLGPAGNAASTAGPFPFHLSQHMLASQGIPMP.[T]	3760.82102
7285	[P].GHSTPVPEGKNAMSLFSSTKTDVRQDNAAGRAGSSSL.[T]	3760.83074
7286	[E].PGARGATGAKGESGVDGLMGPPGPQGPPGDPGPPGTPGLDGK.[P]	3760.83476
7287	[H].PNPPQSPATPFAPAASPSAPQSPGYQVSQLMNRSPVA.[G]	3760.83878
7288	[L].TQGRLEGPPASPRDGATAWGGREAASWQPPADLSALS.[L]	3760.84262
7289	[R].GRDPGVGGTGLEQGPSAGAASAGPQVSLYQGAPPAAEQGVV.[S]	3760.85252
7290	[Q].QAQLQEFGQSSRKGEFPGGLMGPVRMISSGHELTT.[D]	3760.85339
7291	[A].GAPGEGTTTTLPAGTAAPPATSRSPAASGAAASPPPISNTTT.[Q]	3760.86241
7292	[V].PQGIPGAPGAPGFSGPKGEPGDILTFPGMKGDKGDLGSPG.[V]	3760.86393
7293	[H].STPLPPQGSGQPRGERELPNSHSMICPKAAGAPAAPP.[A]	3760.86462
7294	[E].KGSKGDVGFPGLAGSPGIPGSKGEQGFMGPPGPQGQPGLP.[G]	3760.87517
7295	[S].AKGKGASADIHGYDHRHGGGGGGGSGGALGSGAAGGGGKGSWGA.[A]	3761.76242
7296	[F].TAASSFHLQQAHLKMSSPQFSQAMPSRPMAPMSSA.[A]	3761.7655
7297	[P].GQPGEAEPLPNWGFGAQAGGAGSLSPSAGAQSPAIIDSDP.[V]	3761.76779
7298	[P].SNPSTPVGSPSPLTGTSQWPRPGGQAPSSPSYENSLH.[S]	3761.77902
7299	[N].PGMHRDAGNYAFVRKRGGGGPSSELCVTACLAAWP.[G]	3761.7846
7300	[M].GMGHLLASVAGSGGGGGGGGPGTATGLDAGGLGPAGNAASTAGPFP.[F]	3761.79362
7301	[S].GPHFSPEHKELSNSPPRENSFGSPLEFRNSGGPVA.[E]	3761.80551
7302	[V].KTETHPDTVAAGKEPPGAMASATSQKPGGNQGRPDGSL.[G]	3761.81475
7303	[H].KMGAPGSGIAEYLFDKHTVGHSGGSHQLPGQHASVGF.[L]	3761.82414
7304	[P].AMASGKEREGEPAALRAGEHQPGPADDLAKRSDKEA.[A]	3761.82599
7305	[S].EIDTVGTAPSSPISVTMEPPEPHLIADGPQHHHHL.[H]	3762.81805
7306	[I].PGADAATLQGSRASRPGGSHGDSGSPPPALSSSIRSVMQ.[K]	3762.82124
7307	[A].EPGPPGPPGPPGPMGLQGMQGPKGLDGAKGEKGSSGERGP.[S]	3762.83265

7308	[D].GAPGQKGETGPFGPPGPRGFPGPPGPDGLPGSMGPPGTPSV.[D]	3762.8333
7309	[I].RDLNEVSFLASLTELEQLSIMNNPCVMATPSIPG.[F]	3762.83871
7310	[I].RMQLWLSDQLGQAVGQQPSASQVSPTEPRSSPSPP.[P]	3762.85041
7311	[A].LPGMGGPGPVGTPDIPLGTAPSMPGHNPMRPPAFLQQG.[M]	3762.8553
7312	[T].QITDVPNKSGSNVMVGTLCRKRMCGGRGVGPGQTL.[R]	3762.86186
7313	[Q].AFPGSAGPALQYPPPAYPGAKGGFQVPMIPDYLFPQ.[Q]	3762.8665
7314	[V].PSSLSTPAASSIWSPASISPGSAPASVSMPEPLAAPSNAS.[C]	3764.83236
7315	[P].PTTTAAEPQPTAPPTVCVTGPPTARPSEGPTTGPTGPPA.[A]	3764.84359
7316	[E].KGEKGEKGEPAVVEQGQQFEGPPGAPGPRGVVGPSGPPG.[P]	3764.89907
7317	[P].PGIDGKDGTPGMPGVKGSAGQAGRPGNPGHQGLAGVPGMPG.[T]	3765.8184
7318	[E].SPQSLGATNSSPTPVGRGAQVGPGQGPVPQDGGAAPSEPR.[G]	3765.85392
7319	[S].FYSLGLESTPQLSNENRVLREENHRLQAQLSH.[V]	3765.90556
7320	[S].GIQVGEQSTVQEPATPSPPPPPPPPSTERPRTSAYI.[R]	3765.90824
7321	[A].SLPRPAMGGSLPAMPLRSNSVPGARPAMQQQPQPQP.[Q]	3765.9098
7322	[P].GRAQATWEQPPPLPPKMCRSARPQTLMSKAPVY.[C]	3765.91382
7323	[S].VCTAALSPTAASGPEDVALYVGLIAVAVCLLLLLLVL.[I]	3766.11485
7324	[A].GALGPDLGSDETDLILLVWQVVEPRSHQVGTLHKS.[L]	3766.97626
7325	[S].TSGIAAIMSENLINEAGMKKYKPSDPAFAYAQLTH.[D]	3767.87714
7326	[A].REAAGSASRSGPGGSGSSGRGGAGVPGPGSGGPGGSAGRMSLTP.[K]	3768.7815
7327	[T].NPWGAPAAPASTADPWPSFGAKPAASVDPWGAPTGAGTH.[S]	3768.78298
7328	[F].VSSFNCVFAVYLPEQQAHNRADGSMIVTSDRRN.[Q]	3768.79694
7329	[R].TAHTQNPSPLGMGIGWAPLMAPPHPGFAGTPTMWPL.[W]	3768.81237
7330	[V].ASSPSSAISTATPSPKSTEQTTTNSVTSLNSPHSGLHT.[I]	3768.81586
7331	[T].STGFMKAPASGAKSTPRMRAPASGAMSIPSSTAPISET.[V]	3768.83535
7332	[R].KPASDFSPPGRTVSGTSGLGRPASSSGGPGRPVSGSGGSGR.[P]	3768.87605
7333	[N].SSSSRPATAIPPATPTLATSAQPAAATPASVSSPAGSPGPPG.[S]	3768.90389
7334	[Y].QMHLGVMGSALANTVSQFTLALLLFLYILAKRLH.[Q]	3770.10121
7335	[S].KPDGDVAGTAQSLTEQMNKIALESGEQMESDNCSGG.[D]	3770.64259
7336	[R].PGPPGPPGPKGQPGNRGLGFYGEKGEKGDMGLQGPGGIP.[P]	3770.87075
7337	[A].AASLGGSGAPPPPSMPPPPLGSPFPVISSSMGSPGLPPPAP.[P]	3773.89173
7338	[L].QSGSRTQRETGLPFPISLPTPSSIGAMSEREERR.[F]	3773.89876
7339	[T].LNAQMLNGMIKQEPGTVTALPPHPARAPSPPWPPQ.[G]	3773.92543
7340	[R].VDRTLVKVIPQGSCQRASVNSMLHEYLVNHLPL.[A]	3773.99418
7341	[S].IGDEDEKRGLPGEMGPKGYTGERGFPAVYPGPPGTE.[G]	3776.78608

7342	[T].LTSTSDISIAEMDFANLTLEEKRENEAKSCFQV.[S]	3776.79935
7343	[L].IEGSATEVYAGEWRADRRSGYGVSQRSNGLRYEG.[E]	3776.81239
7344	[V].SYVVSSQRQQGEQSRSAGEEVPVHPRSREQAGNH.[A]	3776.8196
7345	[K].KLQHCLTGGEPLNPEVLEQWKMQTGLELYEGY.[G]	3776.82986
7346	[V].PGQVGFAQGTQAGQLDPSQPQTPQQTQRGPKNVMPS.[L]	3776.84091
7347	[L].AAAVPTSRGMPGTVPPGQAHLASSPPSSQAPGALQECPP.[T]	3776.8483
7348	[Q].QQPATGPQPSLGVSFGAPFGSGIGTGLQSSGLGSSNLGGFG.[T]	3776.85146
7349	[N].AAVPDAAALEASSVHSYLPGASRGGEEVREGTRHTLD.[P]	3776.85867
7350	[H].STPLPPQGSGQPRGERELPNSHSMICPKAAGAPAAPP.[A]	3776.85954
7351	[A].ASSPKEEAPEKHVADRQATEKQEKHEAMAGKAKGS.[R]	3776.86204
7352	[V].EQAGKEAGKVAQGVHDGVNQAGKEAEKLGHGVNHAAGQ.[A]	3776.88113
7353	[A].MMDALPSSTPHQPMQVLSGLAESSSVSPTVSFGPRT.[K]	3777.77684
7354	[A].PSETATDLTPGFGSAPVSMLTTVTMLDPGSSAPGGTTPI.[S]	3777.80851
7355	[G].KMVSGGAGISNARPDLTDPAGYGAELAGPHLQASEKVP.[R]	3777.88646
7356	[M].GPSPTQHRSPSGRMRGEVSWAQMTASLLSVPPPSSS.[C]	3778.8388
7357	[D].AGGLGPAGNAASTAGPFPFHLSQHMLASQGIPMPTFGGL.[F]	3778.84685
7358	[S].RTLHNDAASRVAPDAAPGSEAPGPGPSTGALQERSPGSP.[P]	3778.84916
7359	[G].MPGVKGSAGQAGRPGNPGHQGLAGVPGMPGTKGGPGDKGEP.[G]	3778.85003
7360	[S].PGPASPGPAPRNSSTRPCLPEIHRSSAPGALELLCE.[V]	3778.87519
7361	[L].GPPAASTPAGPPSGGASPTPPAASPSGGSATRPSSGPTSEAPR.[P]	3779.82195
7362	[E].GPSTAPPHFGQTGPVFPAVPPALSSAPGAPAAAAAASMSAPA.[P]	3779.885
7363	[S].PRNGAAGQREGGGAGGPAGPGLVGVVAEEAARQPSSHSSAL.[G]	3779.89203
7364	[L].DLPPLGDARREERRANSNNNNRKPSHAREEKPG.[P]	3780.90975
7365	[V].PGSLMVSGLTEAFVMVQSRVEELVERLSWDFRL.[G]	3780.94516
7366	[S].KGSGGASAEGGPTGLAHGRISCGGGINVDVNQHPDGGPGGK.[A]	3782.80117
7367	[K].RAGQQLTACTAFPEKLRGLPGSGPGPGGLPGSGPGPGGRA.[A]	3783.94598
7368	[A].LSTPSYRLSVLETYFIPKDGSLASYKEYISMLP.[G]	3784.93939
7369	[A].QAQDASRPSSPQATTPNPVPSSTEAQGVAGPAAEIPASGG.[H]	3786.81653
7370	[L].AAGDAVWVRMFQRDRDNAIFGERGDLYITFSGH.[L]	3786.81939
7371	[V].AGRNHGINAHAATTQYANGVVPSGQTANAVAHRAQEM.[L]	3786.82257
7372	[S].QGEVFENTLVQNEPPAATELNVGNVQTTSVQTTSSP.[Q]	3786.83045
7373	[A].AAPTAAASPSPSPSSSSTGVFGNLPLTSAGSSGLFSFGGLF.[F]	3786.84972
7374	[K].EEPERFLHTPALGPASHHRPFPGLSDGGDGKQGHR.[L]	3786.85961
7375	[A].PPPYASPTAPSPSSPVPTPSPSTPAPPTSSPSSPAPPNPS.[P]	3787.83374

7376	[P].PPYASPTAPSPSSPVPTPSPSTPAPPTSSPSSPAPPNPSP.[S]	3787.83374
7377	[A].ICLNPQATWTQTLIKLLRKRNWSISPAPVVKR.[R]	3788.16323
7378	[V].HCFLPVAEGWAGEPRSAASRRVSSSSELDLPPGEH.[C]	3788.81978
7379	[E].PGFGSGLPMAPGGAGPEDPSPSVTLAYIYRELYAFFG.[D]	3788.83051
7380	[Q].PGGAAPPGHQMFAIQPGAAEGGQFLGGPPPGVCPPELQP.[D]	3788.8312
7381	[R].RDELPEVNDLTLDGNPFLDPGALQHQDAPMISGVV.[P]	3788.84359
7382	[R].GGRVAQAAAGAPQNPRGTSRLWHFPRGPSSTWLSQS.[P]	3788.92288
7383	[S].AAPACAVSTPEQSATPAGAVPTPEQSATLAGAVSTPEEPA.[T]	3789.81235
7384	[L].GPQGPPGPQGHIGPQGPPGPQGHLGPQGPPGTPGMQGPPGP.[R]	3789.83028
7385	[A].VSMPQALSYFGRSVDGRLDLDGDDLVDVAVDVTPPA.[I]	3789.86399
7386	[K].EGPPGTKGNQGPSGPQGPLGYPGPRGIKGVDGIRGLKGH.[K]	3789.98956
7387	[S].GAPVGGSISSGSSASSVTVTRSYRSVGGSGGGSFGDSLVTR.[S]	3790.85906
7388	[P].MQGVPRGSSMGVRNSPQALRRTASGGRTEQGMSRST.[S]	3790.87185
7389	[L].GQFLWEVDPGLPAEKAGMQAGDRLVAVAGESVEGLGH.[E]	3790.88573
7390	[L].PGLAGHHGDQGAPGAVGPAGPRGPAGPSGPAGKDGRTGQPGAV.[G]	3790.88689
7391	[P].TAAMMRGLEGPHGSPRSSAPMQQLNRSSSERDGPPV.[E]	3791.81227
7392	[G].GPSTSRVTDPQGKSDIPTDLFDFYEQMDKDEEE.[E]	3792.6705
7393	[Q].ATTPPASSLCPPGAAGTPAGSQPSSPRYRPYTITHPSG.[S]	3792.83985
7394	[G].QPGGPFLNTTLAQQQQQQHSGGAGALGGPSGGFFPGNLA.[L]	3792.84771
7395	[T].PWPLMETLDAQLAAQSPGMGRGPCAPPLEAPLHPMG.[L]	3793.83212
7396	[W].KAPKDCAAPVTPSADDTVSPGGVPSPSASPPRVTSMAST.[Q]	3793.83713
7397	[I].RHSRDKKNEPNPQRFDRIAHTKETMLSDGLNS.[L]	3793.88992
7398	[A].KVDIDAPDVDVHGPDWHLKMPKVKMPKFSMPGF.[K]	3793.89029
7399	[P].PGLSSSGVSAASQGAGGGPPPAPPLPTAQGPSGGGTGAPSLASAI.[A]	3793.89913
7400	[G].RPVSGSGGSGRPMGSSGGPGRPVSSPHELRRPVSGSGPPG.[R]	3793.90116
7401	[P].PAAASSSLAAKASFGGGGGGGGGGGGLFAASGVISYAEVLKQGP.[L]	3793.91439
7402	[A].RESRQSSPSSSAALPGPPAPLVDGSAVPGTALGTEPRHG.[G]	3793.9216
7403	[R].STQSRGHSGARKASLSCSVLGSSPVHRARLQPSSTSG.[Q]	3793.92229
7404	[P].GRWVSSSASDPVPSDGPPPPPPVPSSEGGQLRHNPLH.[I]	3794.86336
7405	[S].AGRPASASPAPNATADGSKTSRASVDTTPSVIQHRAMM.[R]	3794.86608
7406	[L].KDAAGDPNKPLLAFSGSCPPSPPSVISSIAALENQMK.[M]	3794.90917
7407	[A].PGSGSLGRVGPSASEDARARKAEGAEAAARERAAVSGKD.[A]	3794.92406
7408	[G].PAAAVAEPVPGQSDTATGLGPCLPPAAASPSEAGSTGPSRP.[G]	3796.84466
7409	[P].QSLGPLGQPEPPSSKMPPSPGLLSTPAQDSPANSSRAP.[G]	3796.88104

7410	[T].PPSNISGSPGDVGAPGIFGLEGYRGPPGPPGPAALPGSKGD.[E]	3796.89293
7411	[A].PGGVSGPSPAQLGAPALGGQQSVSNKHLAWSGVLEWQE.[K]	3796.90416
7412	[L].GPQGPPGPQGNAGPQGHLGPQGPPGPQGHIGPQGPPGPQGH.[L]	3797.83921
7413	[S].QAPGPSTGRTTSSEPVGQAEATGRLQSLANGPSNGSSSR.[Q]	3797.83972
7414	[K].DIRSEMSTIRQNLGVCPQHNVLFDMLTVEEHI.[W]	3797.84077
7415	[S].NGGGGSQAPGRATGPARERGGPSAPEGARAAPDACGRARP.[A]	3797.84577
7416	[C].AAAPSEEPVAQPAAADTQEALKAAEKPRAHQEPTDTT.[E]	3797.85766
7417	[L].GTPTLDTLATASSSGTHLAVTQGFPHSKMTALTSQGPE.[D]	3797.86506
7418	[L].PSAASMVPVMNTGPNMGQPQPGMTSNGPLPDPSMIRS.[S]	3799.72165
7419	[A].GPPAVHGLAMAPASVAPAPAGSGAPPGSLGPSEQLGQAGPTVG.[P]	3799.9072
7420	[S].FLQGYQTQEAMKPVIPVVPQKRTPTDTEVECLP.[V]	3799.93974
7421	[G].AGGISPQHIAQDSSLDGPPGPPDVATVPLEGLSLPQPAD.[L]	3800.89774
7422	[G].GAGPGAGIGSGSGAGGSSEPSACSDHPSPGCPLKEEEKQH.[S]	3803.66202
7423	[S].FYAVDLSEPQKAGAGDGSARREPYAPYPAGYPRTF.[E]	3804.8405
7424	[P].GSPGFPGVPGSPGIMGFQGFTGSRGDKGAPGTAGLFGEVGP.[T]	3804.84387
7425	[E].DAPGPAAPQEDSVDLLGLHVEAGPAPAPHAPGGPPSNADL.[L]	3804.84637
7426	[A].PTPSSAPSPLGGSALCGGKPEAGESPPPAPGTPKANGSQPP.[G]	3804.84974
7427	[Q].EGQALASARTGGKAEEPPSQGPGVALCTQVALGMEHLS.[N]	3804.86435
7428	[P].AAAAPLSQDGPQAEPQAPGRPPASGLAAAAEESEPPREL.[E]	3804.87873
7429	[M].PPRGPPFGSPMGHPGPMPPHGMRGPPPLMPPHGYTGP.[P]	3805.81233
7430	[A].PGAAREPSAPLTDTSSLGAAGEGPPGAPSHASEVGPSAPPAS.[G]	3805.82636
7431	[T].SGPGSRISSSAFSRVGSGSSFRGGLGTGMGVAGSYGGAPGLG.[G]	3805.83107
7432	[Q].LYSRTQEASPQVLEAFQNFYPTVGLSADMVAMLP.[K]	3805.84517
7433	[P].VSTTAASPESPPTPQTSLTPPQASPAASKDQSPPPSPPP.[P]	3805.87667
7434	[V].PGPRGEPGALGPKGPPGMDGVGAPGLAGLPGPQGPPGAKGEPG.[T]	3805.94425
7435	[P].QQPPPTQQAVARRPPGGTSPPNGGLPGPLASTSAPPGPPA.[A]	3805.97324
7436	[P].GDGPRERTATTVTDSRGAGGGGSGALPAGTANSGTARHWP.[P]	3806.83016
7437	[A].PGDGPRERTATTVTDSRGAGGGGSGALPAGTANSGTARHW.[P]	3806.83016
7438	[R].AVPREDGAPGDGPRERTATTVTDSRGAGGGGSGALPAGTAN.[S]	3806.84006
7439	[L].QREMKKSLMDLANDACQLLSGERYKEDPWLW.[D]	3810.84005
7440	[A].QGVPGTQGFPSGRHLAGPACLVSITEAEGAGGGGNFILM.[E]	3810.86904
7441	[G].MQPSKPQQPSLNTMIQQQNMQQPMNMMTQSFGA.[V]	3812.69914
7442	[L].NAPARLGIMSSEEMGGGRGGPVAYGAIFPGFGGMRPSL.[G]	3812.83054
7443	[S].NQVPRRAGERRCPAMPPRARSASQDRLEEVTAH.[R]	3814.9161

7444	[L].TQQQQPATGPQPSLGVSFGAPFGSGIGTGLQSSGLGSSNL.[G]	3815.88348
7445	[H].PGSRRGDDLPNADGTWYLRVTLDVAAGEAAGLSCRV.[K]	3815.88819
7446	[G].ELPSMQLQPSWQGPPAALQGQPGAPLAGANFPMGSAKS.[L]	3816.88362
7447	[L].AGRGAAGDGPAAALLQAAGVAADWAAAGLADGARAAGHAGHGPA.[H]	3816.90254
7448	[D].PGAAGGRGGRGGSPGGTRRATCPRPLRPGAVADSAASFGGP.[V]	3816.92838
7449	[T].TGPIQAAFDASVSVPSEGLPQGTSSAPQAPAHPTGASESI.[V]	3817.85152
7450	[C].QSLGGPAAAYATGKASGAGGAGGQAYSPGQPQGLLGPQAYGQ.[G]	3817.85285
7451	[V].NLAETFHGVIYGFNVNAGNVIQQLAAKKGVRIKLH.[K]	3820.11331
7452	[G].GAEGPILMAETVMKVNRGNGKTSSRILLLTKGHVII.[T]	3820.12994
7453	[P].KDDTSLHLFHISSGKSPKHSCGLSEKQTTPLKQE.[H]	3820.92866
7454	[V].NIEGPEGKLKGPKFKMPEMNIKAPKISMPDVDLH.[M]	3820.97983
7455	[L].WYLPFQNPNSPRRSKSLKHKNGFSVCTSASNTL.[P]	3821.92927
7456	[P].AGPGLAQPSSHREAGGGRGVAPPRERRAGPGVFEAQSR.[P]	3822.97195
7457	[K].PGGNQGRPDGSLGGTAPLIFPDSKNVPPVGTLAPEANPK.[A]	3822.97733
7458	[L].GAETGAGSRPAKRPAVGRLQASLAGQLGPGAADISLFEF.[I]	3824.02019
7459	[S].SSPQAQPPRPAELSDEEVAELFQRLAEMQQEKW.[M]	3824.85483
7460	[F].PSQPTANICSEINKHYGRISTSPFISMLKPCMK.[I]	3824.85907
7461	[A].LGENNKFANFLMKVLKKRIKRVKKKEVEGDVGL.[Y]	3829.23606
7462	[E].GPGSPGVPGSPPEAAAEPPTGLRFSPEQVACVCEALLQ.[A]	3829.85238
7463	[S].TAAGSLPFSASTPFPSSTLHTTDLSVSSPSHWITSSPA.[T]	3829.85554
7464	[W].CLRARDSGSLAPQCGSVLELSRGHSPGAGSPPGPGADR.[A]	3829.85691
7465	[V].PGAAMTIVHLIGGPMTGDTVAATGATTTAEIGEKPTMTQ.[T]	3829.86565
7466	[P].QRGLEEGSPVSSVEDVVIDLSSSTRQETERVQQGAG.[A]	3829.87986
7467	[A].GSSRQKPAPRSHKGQTACGAATVRGGASGNLQPGPGETV.[G]	3829.92229
7468	[P].VTAPTQSSEEQPGKKAVQAAAEPAHAGPAGGRSVSTIFP.[V]	3829.94675
7469	[R].NQPTNVTLSSGFVADSGVKHHNGGGKPFQSQKESHPG.[T]	3830.85934
7470	[G].AGPPNPAINGSAPRDLFDMKPFEDALRMPPPPQSIA.[M]	3830.89927
7471	[S].PGLSQPSGVYASSSVQDFRHPPPQLLSTSNRAYMR.[N]	3830.90311
7472	[G].AGATGDGGSLLPASNFAAAPSYAHYMRYPHMPGMDPH.[G]	3831.71009
7473	[R].EGNIHVTVSGNGKPVMREMKNTQDGAQGSWFKVTI.[P]	3831.8905
7474	[K].SAAPAPISASCPEPPIGSAVPTSSASIPVTSAVGDPGVGSVS.[P]	3831.89569
7475	[L].GGNPAVAVPSSLSTPAASSIWSPASISPGSAPASVSMPEPL.[A]	3831.91094
7476	[G].NPAVTSPVSTQTPTSVSVEVLGEPSLTSIAMSTWTAVA.[S]	3831.92084
7477	[E].GIPLDMTKMTAPVSRGMSTRSPEMATALKIISSQEP.[S]	3831.94755

7478	[P].QPAAPTQPSTPASSSGQTPTPTPGSVPSASQTQSTPTVQA.[A]	3832.84716
7479	[D].PGGPEMTKTRSASTSSPLQHPRPRMTPQNRGSQEP.[R]	3832.85658
7480	[P].NPPQSPATPFAPAASPSAPQSPGYQVSQLMNRSPVAGQ.[N]	3832.87115
7481	[D].AGLQPSPGTTLGPPAASTPAGPPSGGASPTPPAASPSGGSATRP.[S]	3832.91003
7482	[A].WPGTLRSGMVPRGPTAMTRFGVPAEGRNPPPFPGEP.[W]	3832.91626
7483	[R].PANIDLPPSGIVKGMHKGSNRSSLMDTADGVPVNSRV.[S]	3832.94326
7484	[D].VFTRNNPGFHGAPNSSPIHLNRTPLSPPSVMLHGSP.[V]	3832.94525
7485	[P].KGDPGVSGIPGAPGLPGPKGSTGGMGLPGMPGPKGVAGIPGPQ.[G]	3833.96769
7486	[L].IVCPPGFQGLQASPSKHAGYSVRMENAVPIVTQAPGA.[Q]	3834.94181
7487	[L].YLSYNKYLELTTNSFTSVPSLQRLMLRRVTLK.[N]	3835.09388
7488	[K].ISHESLGSSQCLLEYLLNRLHSGSGRVKLKVLKI.[L]	3835.13747
7489	[L].PGGLGADVSGSLMFNSLSGSTGGIMCNICHKMYSNKG.[T]	3836.7169
7490	[P].APGVFAGLHCPQDLARPLFSSSGATHPAANPFGPSAHP.[G]	3836.87142
7491	[S].PAGGPALSQASSGACFPRKRISSKSLKVGMIPAPKRL.[C]	3837.11021
7492	[D].TAANSPFSSGSSVTSPSGTRFNFSQLASPTTVTQMSLS.[N]	3837.82359
7493	[G].AGGPRPEWGSPEGPAPPARRESLTKKAKRFLANLVP.[R]	3838.09872
7494	[N].PGAGAAKMDKQEKVKLSFDMTASPKVLMSKPMLSGGA.[G]	3838.95738
7495	[F].GPMLGGGSSPLPLPAGGSSSVGGSGGFGSLHQHERMGYQL.[H]	3839.82282
7496	[K].REKECGVTATFDASRTTFTREGSFRVTTATEQAE.[R]	3839.82532
7497	[Q].YTSSSSIKGSGGIGGGSSRMSSVLAGGSCRAPSAYGGLSVS.[S]	3839.82869
7498	[T].LDVSKEIHPEVDSPPGGCDTTLRGLEQAPLNPTCH.[L]	3839.83271
7499	[Q].PTSSGTTLSSNLSGGMPFIDVPTPISSASSETASAVVSPS.[T]	3839.8379
7500	[V].GKTNFPYVRDFVMNLVNSLDVGSDHIRVGLVQFS.[D]	3839.95375
7501	[E].PSPPTSELQAAPEAPPVSSPDPPPALLPAVEAPVTQGEV.[V]	3839.95894
7502	[Y].GPEIEANVTTGPGEGAPGPPGVPIIVRYSSAIAIHWSSG.[D]	3840.95553
7503	[V].FPHVAEAPGWPEEVLGSGYREQLLTDMLELCQGL.[W]	3841.85641
7504	[S].QTVAMQGPARTLTMQRGMNMSVNLMPSPAYNVNSV.[N]	3842.81147
7505	[A].AAAADMSARKMAHPAMFPRRGSGGGSASALGAAGTGVGSSAP.[S]	3842.85955
7506	[P].GWPGTPGAPGPKGDPGFQGMPGIGGSPGITGAKGDMGLPGVP.[G]	3842.86289
7507	[C].PGQTGHYRPVNLSSSENKTVNVSLADLRGGSHPNTGP.[L]	3843.9121
7508	[R].RDRDGQHSWVPRGRGSAGSGRSSPHTPRAHSPAAPR.[V]	3843.92198
7509	[P].GPRGEAPGSGAVCLGPSLEEERGPARGSPTAKARVNKK.[Q]	3843.99947
7510	[S].LPQGDAQPQPPVPKPRTVPVADVLAQEGGGAGWALETP.[E]	3844.00281
7511	[P].GAVGPAGPRGPAGPSGPAGKDGRTGQPGAVGPAGIRGSQGSQGP.[A]	3845.95022

7512	[F].ANPNIFVGENILEESENLHNADQPLRVRGCILTL.[V]	3845.9603
7513	[-].MVIITWDMGGGWKTRKWNLMSKFTKSEKTMGCK.[F]	3846.93144
7514	[S].KPGCPPPAFARSCFDLRGGGGGSGGLTVGDWLDSIRM.[G]	3848.84178
7515	[V].AVPSEAETRPFPETRDPAQDHRPRDPSLGGPATASH.[P]	3849.86515
7516	[P].VSGAAQWAPVLDFAPPGASAYGSLGGPAPPPAPPPPPPPPP.[P]	3849.96391
7517	[V].GAPGLGGPPGEPGLPGIPGPMGPPGAIGFPGPKGEGGVVGPQGP.[P]	3850.9585
7518	[F].QPLPMDADGMTALFALSGQPVIGLELKVTNLPSDPAQ.[P]	3850.96054
7519	[R].GERRGGRAVAPAPMVRYSSGAQGSTLSFPPGVPAPAPVS.[Q]	3850.97695
7520	[L].WYLAAAPSAPAPPAFAYISSIPVLPYPSATVYYAPPA.[P]	3850.9771
7521	[T].LMAIFWTTISYLAISATIGKPPAPSTGGVPGCSRLSN.[R]	3850.98703
7522	[H].APSPSSSAPKVGVHLLEPAARDGAPQPPPPPPPPPPPMP.[L]	3850.99489
7523	[Q].KQGEQQCLIDEDAVCCVCMDGECQNSNAILFC.[D]	3851.55663
7524	[P].RGDRSAAFHPVSFPDEKLGREDKPVIPYQELNSP.[Q]	3851.94636
7525	[T].KAPAQGPPEGEPLQPTQPAQPTQPLQPVQCAQPAQPA.[Q]	3852.93375
7526	[S].TAAVLDRESLYIKNNVYEATFLAADNGIPPASGTGTL.[Q]	3852.96542
7527	[P].GSAVSAAPGTPFEGGNKFQTLDNHQPYPCAEDEDCS.[A]	3853.63407
7528	[V].AAAEDRAKGLAPDVSDAAIISTSTAECLLAPAGLDRQE.[E]	3853.92363
7529	[M].ARGVTNPIMPGGYALAGAAAFSGAVTHSISTALLAFEMT.[G]	3853.92516
7530	[P].SGAAPGSSGPGALPPYAPKLSSSAGLPLGTPGSVLSGISLYD.[P]	3853.98582
7531	[V].PGSTNLQFSQPWPSSDSLLSTFTLLPGYSEMSRHS.[S]	3854.83303
7532	[A].GPDASVMNLISALESRGPQPGPSASSLLSQFRSPSWQ.[T]	3854.91301
7533	[S].GPRSSSSAPPANPPSGLVNPSLPFTSSPDPTPSQNPLSL.[M]	3854.91954
7534	[R].VFPAEEVMIHMFLGDQELSPFLFWEGDTIWANA.[T]	3855.80733
7535	[A].AVAEPVPGQSDTATGLGPCLPPAAASPSEAGSTGPSRPGSPG.[P]	3855.84538
7536	[K].SREFNLMYDGTKEVPMNPVKIYQVCDIPQPQGS.[I]	3856.83429
7537	[Y].PSQPVFAPMLQSNPRMLTSGSHPQAIVSSSTPQYPS.[A]	3856.86328
7538	[L].QPRGSFYSVVPANQGWGDGPLSSRVAADASFTVQSAF.[L]	3856.86777
7539	[L].GPSPTAARPRTPMCPSTPRTPGLNHPASSATTSVPAGGT.[A]	3856.88173
7540	[L].AAPTSVHNHPGGPKTQIFMNGACSPSLLPALPTPMPF.[P]	3856.89717
7541	[S].SSTSVTLHIRDVNDNAPVFHQATYVVHVAENNPPGA.[S]	3856.90014
7542	[S].SSPPQQLSAATPHGINDILSRPSMPVASGAALPSASPSGS.[L]	3856.9134
7543	[V].LGNASASANFNAQATVLVVTPSHTLQEGTGANLTCRVS.[R]	3856.92464
7544	[F].GSPATPPPPSPPSFPPHPDFAAPPPPPPPPAVDYSALPP.[P]	3857.92137
7545	[A].GDPPGPALPQVDETLAERESPPPAREAALRILEPVLG.[Q]	3858.03959

7546	[L].PSGAPATGPSVTNPFQPAPPATLTLNQLQISPVPPVAGAP.[P]	3858.04361
7547	[N].TAAQPRGMQQPPAQPLSSSQPNLRAQVPPPLLSPQVA.[M]	3858.0443
7548	[V].VGGPEAAAAATGGYGPVSGAVSGAKPGKKTRGRVKIKMEF.[I]	3858.08068
7549	[R].TSFTSVSRSGGGGGGGFGRVSLGGAYGAGGFGSRSLYNLGGS.[K]	3858.85425
7550	[I].AAAFSAASWWPLGFTGAAHPLVWPSMRMAGRMWLQ.[M]	3858.89705
7551	[A].GPPGPQGPPGEQGPEGIGKPGAPGTPGQPGIPGMKGHSGAPGP.[A]	3858.89803
7552	[L].AMAPASVAPAPAGSGAPPGSLGPSEQLGQAGPTVGPQQQPPAG.[A]	3858.90792
7553	[W].QGLQAATTKETFEKIREMKTTTSVDVLCDGFPIQ.[F]	3858.92522
7554	[P].PSAAASLGGSGAPPPPSMPPPPLGSPFPVISSSMGSPGLPPP.[A]	3860.92376
7555	[M].PPAKPSPAPETSSAEVAGASGLDQRFGLQLSVTWIPAGA.[S]	3860.98174
7556	[G].LAGHHGDQGAPGAVGPAGPRGPAGPSGPAGKDGRTGQPGAVGPA.[G]	3861.924
7557	[N].GGGVTRAADEDAVAEAFEAGIAGGGQGVLGGAGGGNGPGDPAVPG.[D]	3862.82267
7558	[Q].IGEGQVSLESGAGSGRAQAEQWAAEFIQQQGTSEAWV.[D]	3862.8267
7559	[L].PNPNEKTPSWRDSSVGDKPAVSSWAAGGDPGENVPLSG.[M]	3862.8267
7560	[G].PPPYEPPGHPTPQPGFIPPHVNADGTYMPPGFYPPP.[G]	3862.83626
7561	[Q].DANIPFPRTSGARFCGAGYLVYFTRPMTMHRAVS.[P]	3862.87268
7562	[E].SPTFSPGKLGPRATAEFSTQTPSPTPASDTPRSPGAPAP.[T]	3863.91987
7563	[A].AAAAQMHAKNGGGGSGSHRSSPVPGAPAVCEPLAVPAASPM.[A]	3866.84832
7564	[F].GLDPKTPMEMLYHHVHRLNMSGPFGGAVSAAGLTQM.[P]	3866.85974
7565	[T].FSGEFVDIHPGGASKLMLAAGGPLEPFWAHYAVHNQ.[P]	3866.87477
7566	[W].KEPRDGAQGGGPGGAGTPPRTGAGLPLPTHGGGFGSGCGRPA.[P]	3866.88517
7567	[L].PSLSQPGDLSSSPLSQLSSSLSSHQSSLASTHTPLSAST.[S]	3866.88902
7568	[P].LQADHYAALLGSNSESWGWDIGRGKLYHQSKGPGAP.[Q]	3866.89974
7569	[A].PASASAPAPVPAPAPAPAPSPAPASSSDPAAAATAAPGQTPASAP.[A]	3866.91954
7570	[S].LGATNSSPTPVGRGAQVGPGQGPVPQDGGAAPSEPRGTAALS.[E]	3866.93798
7571	[L].GPPRPATAVNPTEETTPTAVAATSSTSSPSSEATTPSPGAS.[P]	3867.83665
7572	[G].PGQAPQLPLSESSAPGPPHGPPPGLRPDAPGGGGGSVPGKPP.[S]	3868.97291
7573	[L].RSFPQQQFAHQGNPAAYSMVHMNGSSGPMGQMNMN.[S]	3869.6458
7574	[T].RRNSSSPPSPSSMNQRRLGPREVGGQGAGSAGGLEPVH.[P]	3869.92843
7575	[S].PGLQGFPGITPPSNISGSPGDVGAPGIFGLEGYRGPPGPPG.[P]	3870.94496
7576	[G].PQGPPQGLPRPQDMHGPQGIQRHPGPHGPLGPQGPPGP.[Q]	3870.94699
7577	[T].PSVNGGGGSVLGSAGSGGGPVGSVENGKPGADVVDLTLDSSSSS.[E]	3871.8428
7578	[S].SSMVTSAHKGTSSGATMAPASKATPSSVPSSETAPSAASHI.[T]	3871.84367
7579	[A].QVGICRPGDFGSDVSHLNLHKTFCIPHGGGGPGMGPI.[G]	3871.85776

7580	[V].APGMNRQQVSLLAQRLSGGPGSDLQNHVAAGSGQERGAG.[D]	3871.93285
7581	[F].GDIGDTIDLPGSPGLKGERGTTGAPGLKGFFGQKGAEGDV.[G]	3871.94608
7582	[K].VYEELPFQGLTGTELQLSNGKSEPVVAPSSGPSAQKQ.[H]	3872.95525
7583	[P].PAGQAPFQAQPAPPASRMLTGSHSFAASGMAGVPVVPLR.[G]	3872.96869
7584	[L].FLEDELDIFGKNSDIGQAAERRLMMCQTTFLPQ.[M]	3873.86084
7585	[G].PSPGPGPSPGAMLGPSPGPSPGSAHSIMGPSPGPPSAGHPIPT.[Q]	3873.8687
7586	[P].RGAEDREDKSDKGTLRPAKSMDSLSAVAGVSDEPEGL.[V]	3873.88831
7587	[I].GLGEGAGPSPFLSGKPFKCPECKKSFALSSELLLHQ.[K]	3873.96663
7588	[S].GTPPQPCVLSAPQPGPPTSSVTTATTDPGASSLGKAPSNSG.[R]	3874.87635
7589	[E].VENEITKMLNVCFSTLPGYTRSTAHVSRESSVVM.[M]	3874.87722
7590	[Y].QWTWRLPHGDIGELSEFAGAAAMKQEHSNVAANPR.[Q]	3874.88305
7591	[L].PGPSTQVTAGSNHTAALLMDGQVFTFGSFSKGQLGRPI.[L]	3874.95448
7592	[L].QEQLAEMAVSAKREALGATGMMGPPGPPGPPGYPGKQGP.[H]	3875.88772
7593	[P].RWNAGSAAGEVEPGTSRGSFLITESAPTYAHLSISQQ.[S]	3875.89472
7594	[Q].PGQAPGFPGGADDRIREFSLAGGIWHGRADGLQVGQDA.[R]	3875.89606
7595	[-].MSGARGERVGGGSPDTSVDAAAPLPATVEFGLSPCLGLQH.[S]	3875.89809
7596	[S].INGQPLDGLSHADVVNLLKNAYGRIILQVLRSMEL.[A]	3876.1164
7597	[Q].QQPATGPQPSLGVSFGAPFGSGIGTGLQSSGLGSSNLGGFGT.[S]	3877.89913
7598	[I].QAVTETLAHGGAVTDGEGITQRMWNRSFQGVTGYLK.[M]	3878.92424
7599	[A].WKRPMFPSIHEDTVPTTASFGAKMFNLTSQVLEP.[A]	3878.92443
7600	[D].GVQVVDCGLSLNDAAFEKMIISELVHNNLRGGSKAH.[N]	3878.964
7601	[V].FYWYRGARGLSLKTKTQRSMMATCEIPAVRER.[D]	3878.97273
7602	[G].GKSPEARGGGGRGWADPRTGRQEEEDLLRVEEQLGS.[D]	3879.90808
7603	[H].KGQTACGAATVRGGASGNLQPGPGETVGLRCTRPQWSP.[W]	3879.90895
7604	[L].PFRFGSGGGDKGPVMALRGPPGPMGYTGRPGPLGQPGSPG.[M]	3879.91699
7605	[F].VFLSMFVGVMIIHTEDSIKKFERELMLERHMN.[L]	3879.97806
7606	[L].FPETREPTEEEPPMNGEASWEEMGGAVGGEDASLLT.[G]	3881.66403
7607	[G].RGAEPGGPQPPGERDPGSLQHPEGAEPGSPQALGGREPGG.[P]	3882.85023
7608	[K].AAQADVQEKLSCTSKHLAECQAAMLRKDEEGAALH.[Q]	3882.85313
7609	[G].RQGPEPGGCPAALTSLSFSSLQPGPTPSGTNVGSSGRSPS.[K]	3882.86752
7610	[E].LTCPANQELSGEKPLARSSEEWRGGGDDKVKTETH.[P]	3882.86752
7611	[G].APPQQVQYGQPAPAVAPPMAPSHGTSVTPNPASEYVQP.[S]	3882.87556
7612	[Q].PGLMAQMATTAAGVAVGSAVGHTLGHALTGGFSGGSSAEPSR.[P]	3882.88614
7613	[D].AAISEGRGTQREENPLEGNILAGEAASRAGNSGNEAASK.[G]	3882.89249

7614	[S].KAFGTCSSHLIAVGIFFASITFMYFKPPSSNSLEQ.[N]	3882.92336
7615	[Q].NKGFGFGTGFGTTTGTSTGLGTGLGTGLGFGGFNTQQQQQ.[Q]	3884.84743
7616	[P].GAMLGPSPGPSPGSAHSIMGPSPGPPSAGHPIPTQGPGGYPQ.[D]	3884.8483
7617	[T].APAAGTATISQDTSHLTTGPVSGLASGSSVLNVVSMQTTTA.[P]	3886.93386
7618	[G].NTVSNVPGVGDTVSDPRGLQKAVTEAPEPPGVPVTTEAT.[P]	3886.96688
7619	[A].QPAAPAHEAMDCSNPQEENGFGPRPQGISPCLAGAPE.[E]	3887.71703
7620	[L].GPAGNAASTAGPFPFHLSQHMLASQGIPMPTFGGLFPY.[P]	3887.86725
7621	[G].PQGNNLPSPGAGSWPPPAFPALPSSFLGTPDPAHLGLPE.[S]	3887.93915
7622	[A].PASPRSPKAGTSEGPVDSVPYLDRMPFLAKGKQTTGE.[E]	3887.95963
7623	[V].SSPPPQALPPGTQMTGPPGPPPPMHSAQQPGYQLQQNG.[S]	3888.84322
7624	[I].AADEMVIEYVGQNIRQMVADMREKRYVQEGIGSS.[Y]	3888.86772
7625	[M].PGPADQAMASPLQNWLRLYGYLPQPSRHMSTMRS.[A]	3888.87308
7626	[-].MPGPADQAMASPLQNWLRLYGYLPQPSRHMSTMRS.[A]	3888.87308
7627	[S].PSGPGKEPGRPSQHQQPHGPSWLLSPTMGSVYSEYL.[S]	3888.87623
7628	[H].LYSATGTITSPTGETWTIPVYSAQPRGDPQQQSITH.[I]	3888.90389
7629	[G].PQGYPGIGKPGMPGMPGKPGAMGMPGAKGEIGPKGEIGPMG.[I]	3888.90899
7630	[D].MGVKGDRGEVGVPGSRGEDGPEGPKGRTGPTGDPGPPGLM.[G]	3889.85557
7631	[P].SGAATTTAAAAASAPAPGPASSPEASPAPGFPFPPPWMGMPL.[P]	3889.85641
7632	[A].VSMAQPLAGITMSHTTTPMVTYPIASQSMRITAMPH.[-]	3889.87775
7633	[E].DPDAALTGVVETTIEPSPYPHPQFPDVTLWDLPGAGS.[P]	3889.88069
7634	[A].GPGLSYCVWVVSATEVLQPCAPGTLCPPLTLTFSPD.[S]	3889.88493
7635	[V].PVEVPAEAEASAEIPAEASASVEALAQAEAPAQYPSEPL.[I]	3889.88656
7636	[Q].SLPSPTSAPPGTPTQQPSTPQTPQPPAQPQPSPVSMSPA.[G]	3889.89127
7637	[C].RQRCPRDWSVPLRPSQDVSFEEEVTPSFASSLQ.[V]	3890.88786
7638	[Q].GPPGPQGHLGPQGPPGTPGMQGPPGPRGMQGPPHPHGIQGG.[P]	3891.86669
7639	[S].LQSMGTDQLLDLFTLDKDGKAEKADTSTSGKTSMRS.[I]	3891.89504
7640	[P].RSGAPPAGGVSEEQLQGGSVGEAGTGGGMGVGVLSDIYYEP.[L]	3892.8294
7641	[C].GGGFGAGVGGFGGSGGFGGGISGAVGGFGGLGGFGGGISGAVGGFGGLGG.[F]	3892.84262
7642	[Q].QPQQFPGYGQQPTSQAPAPAFSGQPQQLPAQPPQQY.[Q]	3892.86777
7643	[G].PGSPATLSPSAGVPQPVGMEALDQAEGPAASQRAMPPPPP.[A]	3892.88441
7644	[L].PLTAEQSPGGSLGLGSLQDPGENRCEVGKSSPESLLPE.[S]	3892.88691
7645	[H].LASPHFTNNSVEIKSAQENPFIFSTVTQTVEELNT.[D]	3892.92395
7646	[A].STPAGPPSGGASPTPPAASPSGGSATRPSSGPTSEAPRPPEPS.[Q]	3893.85364
7647	[F].HSSLSKYSRLEEFEQIQSQTFSQVQMFLKDSW.[I]	3893.88031

7648	[E].GSEPGPGLSSTSPVGEPSAGLGPGPEDVPPFPPMLLNAPGE.[A]	3894.87422
7649	[G].FPAGFAAMPPGEEVKSTLSMLPMVLPGMAAVPQMFGVG.[G]	3894.90111
7650	[P].GPGVSFSPGPTPTPAPTAGSFAGGAGGPSPPLFARPEAAHEP.[P]	3895.90383
7651	[M].FVQMLLNICGEAQGLEGLLSGNELQSLMIATTCLR.[E]	3895.94246
7652	[A].AGDGPAAALLQAAGVAADWAAAGLADGARAAGHAGHGPAHGGLAG.[H]	3896.92875
7653	[P].EIVHGLAQFPDTLPGPGGLAEVAGTCVPHAHVSPGPSGAP.[R]	3896.93883
7654	[Q].KPEAQPANGENPLNVLKQKAEQQAASSSGQGQQAQPPA.[P]	3896.94854
7655	[G].VAPMVGTPAPGGSPYGQQVGVLGPPGQQAPPPYPGPHPAGP.[P]	3897.9381
7656	[S].IFWQWDRLALTNASSTTLMPNGSLHLAACPLTGASP.[P]	3897.94147
7657	[H].QAAPPTSSPAASFPPPPSSGASFQHGGPGAPPTSSAYALPPG.[T]	3898.86711
7658	[F].WGRWAPPAMTANPGQALSALLLTLLLRLTGGRPSISL.[V]	3899.18403
7659	[N].GPGSLFASENFLGISSHPRNDFGNFFGSAVTKPASSVT.[P]	3899.89874
7660	[R].PGSPPRSGQAAVNKGSSNNRKMAEDKKIVIMPCKCA.[P]	3899.94592
7661	[S].RQSPDHPTVGAGMLHITENGPTEVDYSPSDMQDSSL.[S]	3900.7287
7662	[G].VSDGRGSVSAQSGFGCCPDGVTVAEGPHQAGCASSHRR.[D]	3900.73072
7663	[L].GPAGPGTGGPGVASPTITVAMPGVPAFLQGMTDFLQATQTA.[A]	3900.91465
7664	[A].TGGIPGGADLEESFLFPECVQGFGAGTDPPRPAAEAAAAA.[G]	3901.83376
7665	[C].FYRDTDMASVVYVYVQDYRSPFIASVSDQHEVV.[Y]	3901.83778
7666	[I].YLAYRNFMIDTYRLNPQEYLTSTACRRNLAGD.[V]	3901.87485
7667	[G].APVTSAGMFGGTSLRFGLNFSGAYGAAAVASTTASTTTTTT.[V]	3901.89127
7668	[R].PPGSGPGPGPATGAKTEPGSGPRAQARTGGTTSPKHGRTEH.[Q]	3901.94005
7669	[T].QSCLRPPPSTSTTSTVAPASGSGLGPSPARASPVNRPSLG.[A]	3902.97774
7670	[S].AVPSAAGYALMAGAHGLWMLLLGRMLTGFAGGLTAACIP.[P]	3902.99404
7671	[L].QAMPAAGGVLYQPSGPASFAGTFSPAGSVEGSPMHTMYM.[S]	3903.74851
7672	[Q].KQCSIINSATFSACHAHVRGAPRAPGEGGMEVGPGSPP.[H]	3903.84357
7673	[I].FPGTFVGTAESTSPPLSSTSPTTAAATMPMGPSVADLAPPG.[E]	3903.8667
7674	[P].PPPPPGMLMPPMPGPGPGPGPGPGPGPGPGHSMRLPVPQGH.[G]	3903.90663
7675	[H].PHHIMPPQQHYAPPPPPPPPISHPMPHPPQAAGTPH.[L]	3903.91112
7676	[G].VDQRGSSPTSHPRPGAPPSASSAASRAPPESSAHRLSGGP.[S]	3903.91931
7677	[P].PGEHSLPLDRHPQPAGPGSPQGKEGTTTQDYVPDKPL.[D]	3903.92602
7678	[P].FILSTYCLMRGIMAPKDIMTNTHAKSILNSMNSL.[R]	3903.92979
7679	[R].PGNPGHQGLAGVPGMPGTKGGPGDKGEPGRQGFPGVSGPPGK.[E]	3903.93073
7680	[V].VIQDINDNNPFFPTREMKLEISEAVAPGTRFPLE.[S]	3903.95856
7681	[E].AKGQGTLDPGGPRVRHGSGTPGSVGGLYRDMGAQGGRPSL.[I]	3903.97432

7682	[P].SPKSEVSSNVPERELLSINVHSSFAASPTRSVNSKY.[N]	3903.98353
7683	[S].STPCPAPRSHSQTLPEAPPTTSPEGPARDMQPTMKF.[V]	3904.8415
7684	[T].FGPAFSAVTTITKADGTSTYKQHRRTPSSSSSLAYSP.[R]	3904.94642
7685	[L].PPQSAFPPAAFMPPVQTSIPGLRPYPGLDQMSFLPH.[M]	3904.95533
7686	[P].PGPRGMQGPPHPHGIQGGPGSQGIQGPVSQGPLMGLNPRG.[M]	3904.95584
7687	[S].PHHVGQSGTKVMSEPLSCAARLQAVASMVEKRASPAT.[S]	3904.95787
7688	[S].PTASSLSPGPDAPLAPASSAGPGPGLSVAPGPGVSFSPGPTPTP.[A]	3904.96034
7689	[A].AAAPTAAPGPAQPGHVSPTPATTSPGEKGEAGTPVAAGTTAAAI.[P]	3904.96755
7690	[H].GINDILSRPSMPVASGAALPSASPSGSLSPPPPPPGLYFS.[P]	3904.97896
7691	[E].GPPGPPGEAGLDGAKGEKGVQGEKGDRGPLGLPGASGLDGRP.[G]	3904.99001
7692	[G].TAGPRPEAAGPGTASSAVPPTEDFLPLPTGFLQMPRGLT.[D]	3904.9902
7693	[N].LTHIKAGCILCGYLKLMPMFLMVMPGMISRVLY.[P]	3904.99147
7694	[D].KSYTHPSSLRKHMKIHCKSPPPSPGALGYSSVGTPV.[G]	3904.99491
7695	[R].YVLDPSARPIWGNVTRWFITCVQQPEFRAVLGE.[V]	3904.99556
7696	[Q].IIIQQPQTAVTAGQTQTQQQIAVQGQQVAQTAEGQTI.[V]	3905.0363
7697	[T].QLACVWARTTEAASCRRGLGDRPGLIAILALAVLLL.[A]	3905.17281
7698	[Q].SHGGPPQGAVPQSGVPALSASTPSPYPYIGHPQGEQPGQA.[P]	3905.88415
7699	[Q].SPQNHALQPHHHIPMVPAQQPMVPQQPMVPVPGQH.[S]	3905.92612
7700	[A].QVMPGAQPIAWGQPGLFPATQQPWPAVAGQFPPAAFM.[P]	3905.92945
7701	[R].LGFPGGFVDLRDGSLEDGLNRELGEELGEAAAAFRVE.[R]	3905.93043
7702	[H].VDAAAGAVPPSPPAAALGATCAAAFPSAASVTSAGATSASSVHL.[P]	3905.93381
7703	[G].PAASSHGSPVPLPSDLSFRSPTPSNLPMVQLWATHAH.[E]	3905.93917
7704	[A].KSWASLFHDSKPSSSSSPVVSVETKYSPPATSPLVSE.[K]	3905.94435
7705	[L].KETEKNDISPRGKMPEIIDVTEEMRTDLKETEK.[N]	3905.94707
7706	[H].RMTVSTAAHGTHLSPQLPHRMLSTSSTLTRDYHSL.[T]	3905.94975
7707	[S].SGSLFATVGSRSSTPQHPPLLTQPRACGSASPAPQLSAS.[P]	3905.95627
7708	[L].VQQNTRSQTETFRAGRDTLCSLGRVAHRGMEKSL.[F]	3905.95696
7709	[P].GVYLLQWRMHSPPAHAACSLPGVEDVLTAVHSPGPR.[C]	3905.96903
7710	[S].PLPEPPEKMVPRGCLSPRAGPPAARERGGGGLEEEPV.[D]	3905.9749
7711	[D].SGVKHHNGGGKPFQSQKESHPGTSRQRQTRTTHRP.[L]	3905.98392
7712	[M].DKEYLSLMAELGEAPVPASVGSTSGPATTPLASAPRPAAP.[A]	3906.97936
7713	[I].MLQLSQQLSGINAVFYYSTGIFKDAGVQEPVYATIG.[A]	3908.9779
7714	[F].GANQTPTFGQSQGASQPHPPGFGSISSSGALFSAGSQPAPP.[T]	3909.84268
7715	[F].GAGTDPPRPAAEAAAAAGGGGATAAAARGGEAAAEVTGWPAGAPGP.[C]	3909.88628

7716	[S].KIEYTCPPQKCSFRGVCNSEDHCHCHVGWAPP.[L]	3913.68727
7717	[Q].PHGFLNALDDRISFSPDSVLEPSLSSHSDIDLFSQA.[S]	3913.8879
7718	[S].LNCRGPPVGAHVAPTFVLATPISGPALMWTPQSVSRE.[M]	3914.02039
7719	[N].ANREEPVSLAFPNPYQFVSSVDYNPRDNQLYVW.[N]	3914.87728
7720	[D].MHGPQGIQRHPGPHGPLGPQGPPGPQGNAGPQGHLGPQGP.[P]	3914.91166
7721	[G].RFCGRVPPPPFTSSWHVMSVVFHSDKHVASRGFS.[A]	3914.91185
7722	[S].PQPRGQEAESLDPPSVPVNPALYGNGLELQQLSALDD.[R]	3914.94066
7723	[V].DPKTKNCTTLAGTGNASNIIGSNFTDSTFNEPGGLCVG.[E]	3915.81237
7724	[L].TLSGPPRGDSTCRSLGGPDRSEPPGLQVTCPLSPDPR.[L]	3916.90286
7725	[D].SGSPRAAPGEAPRERHTSTGNIQVGLPEPASVSNHVSAP.[F]	3917.96011
7726	[I].QGGPGSQGIQGPVSQGPLMGLNPRGMQGPPGPRENQGPAP.[Q]	3918.90861
7727	[K].NRSPADAGRGVDEAPSSTSKGKTNGADPVPGAETLIVAEP.[V]	3918.94279
7728	[Q].NEWRYLLSGGSVSVMSENPAPDWLTDRAWRDIQA.[L]	3919.88204
7729	[H].LWDMIILSKMAGSYSGKTFTGVETKPEMIGHCAGR.[F]	3919.88495
7730	[S].PARGSGFSGKSDIPNTSLDSTSQPVTELDPEKDVLSTS.[R]	3919.90434
7731	[P].PGQAHLASSPPSSQAPGALQECPPTLASGMTLAPVQGTAH.[V]	3919.90654
7732	[A].FPQVYTSGKGSSAAGLTASVMRDPSSRNFIMEGGAMVL.[A]	3919.91394
7733	[G].PAPELQGGLAAPTSVHNHPGGPKTQIFMNGACSPSLLPA.[L]	3919.95819
7734	[P].KCQSLGGPAAAYATGKASGAGGAGGQAYSPGQPQGLLGPQAY.[G]	3920.89842
7735	[L].GVNGAGKTSTFRMVTGDTLPSGGEAILEGHSVAQEPAAAH.[C]	3920.91955
7736	[V].KQTKDLTDTLMDNMSSLTSLSVSTPKISASSSFTSVP.[S]	3920.9355
7737	[M].AGTPKDPERGSRGRGSEGRRPGSTRWGWAADAATAPAAG.[A]	3920.93596
7738	[-].MAGTPKDPERGSRGRGSEGRRPGSTRWGWAADAATAPAAG.[A]	3920.93596
7739	[A].QPSLAQWGKESPAPDTAPSWAAAGNREAIQELESSLK.[E]	3920.94133
7740	[S].GSSGLLGSAGGGGGGGIGLGSLGGGGGLSSSLGGTATIGHLRGSSEH.[H]	3920.94452
7741	[L].KNMHVVDVELSGPPGPTGRSFTVHTHRENPAEPGAVT.[G]	3920.94604
7742	[G].QGHPGAQPPPRSVPPQASSFTPSVSGGPRMPSMTGPLLP.[G]	3920.95344
7743	[M].GPVGPAGNPGAKGERGSSGLDGKPGYPGEPGLNGPKGNPGLPG.[P]	3920.9638
7744	[R].GAQPGRVPVDEGLAGPGAYEDVADGAQSGGLGFNLRIGRP.[K]	3920.9638
7745	[A].GPPEHGEARPPPSAVPEGPAALASEATRPAPSEDPPSQPA.[E]	3921.9002
7746	[F].GGGISGAVGGFGGLGGFGGGISGAVGGFGGLGGFGGAVGGGDAGILPAD.[E]	3921.91545
7747	[K].EGSPVSKMSVSRSSSLRSSSSLSSQGSVASSIGSQTSFR.[S]	3921.92067
7748	[F].GGAVSAAGLTQMPAGNVFTTAEGLFSTLPFPVYSNGIHAA.[Q]	3921.948
7749	[E].PGPPADLDACPRIPGLPGVPGPRGPEGAMGIPGMRGPPGPG.[P]	3921.96731

7750	[H].NRAISVSQSRTPSQKGSPRDQEMTATLLTAGFQTLE.[K]	3921.97232
7751	[R].RSMPVDERDLQAALSPGALTKSEAGTGAQGPRLDWPE.[G]	3922.9352
7752	[R].VSLGCRGASPQAAPPAHLTDGRPPFLGPLPDEVTQEGQ.[A]	3922.95046
7753	[S].WPTIALMAWDSPMLGPLALGGQPPPQPPPGMPHSEHL.[P]	3923.94339
7754	[P].NTQMMRNLLCVHVSVSGNELCLMTSHLESTRRH.[A]	3926.86629
7755	[E].KGGLTQDPKMDSSPEPVAPGKAEVTAEDGEKTSSELQP.[I]	3926.88116
7756	[R].PGPSPGPGPSPGAMLGPSPGPSPGSAHSIMGPSPGPPSAGHPIP.[T]	3926.89525
7757	[P].GPRGPEGAMGIPGMRGPPGPGPPGPVGDPGPIGFGPGYLSGF.[L]	3926.91051
7758	[S].PAAAAAAAAAAPETAPSGLSGLTNPFLTSLQTNPFFEELI.[A]	3928.00147
7759	[E].SPRGGGPPSASAPAASESRPLPRVHRARGAPRQQGPGTGT.[R]	3929.04618
7760	[D].PESGAAPTVNSSSRSSSPSKVMDEGKVSMAAKGPPPFPGA.[P]	3931.88006
7761	[P].GGPPSSPGSRKLSAAGSSDGVMPVAPTSAVSSSGSPASVMTSI.[R]	3931.90118
7762	[G].RPTAPRSSMNLSHNMPGSVTHFTLRDDTLADLPPPS.[T]	3931.91778
7763	[K].FSDPPSPSVLPKPPSHWMGTTIENSNQNRELMAVH.[L]	3932.86943
7764	[Q].RCVPGAGTGVASSATEVRPGQGDGGGQLCDGDLNYCGTH.[Q]	3933.72972
7765	[K].VENENLNQLGEQEKKTSSSERNVPDSQNSRNKFQ.[P]	3933.89215
7766	[R].PGMERQNRRPGPSSKSGGGGGSSGGGGGGPGGRAGPGRGDKRS.[W]	3933.90541
7767	[P].GAPGPKGDPGFQGMPGIGGSPGITGAKGDMGLPGVPGFQGQKG.[L]	3933.93745
7768	[D].GAPGDGPRERTATTVTDSRGAGGGGSGALPAGTANSGTARHW.[P]	3934.88874
7769	[G].VGGFGGSGGFGGGISGAVGGFGGLGGFGGGISGAVGGFGGLGGFGGAVGG.[G]	3934.88957
7770	[I].QGGPGSQGIQGPVSQGPLMGLNPRGMQGPPGPRENQGPAP.[Q]	3934.90353
7771	[K].REIYDLYGREGLTGAGTGPSRAEAGSGGPGFTFTFRSP.[E]	3934.9107
7772	[L].PGSPGAKGEQGPAGHPGEAGLPGPSGNMGPQGPKGIPGNPGLPG.[P]	3934.92531
7773	[R].SPTPGPTSSKTTPTPGSTTGLPLPSTGPTSSPTAPPASATSTG.[A]	3934.94039
7774	[I].PSGGLTEICRKPLSPGCVSSVSDWLISIGLPMYASAL.[S]	3934.97514
7775	[K].VSDERTTRDASDNLTVQNLKGSFSNASGLFEIHGATV.[V]	3936.93223
7776	[G].TQFKPMEPPQPTAPAAPPAPPGPALSPQAGDSESLESQL.[A]	3936.93241
7777	[S].HGNSAFQPIASSCKIVPQGQIPNPESPGKSFQPITMS.[C]	3936.93712
7778	[T].AVPMTTSSIFSRGPETTPSLVPSPGVETSTAVPMTTSSI.[F]	3936.94568
7779	[A].EPGGPAATSPSRPSPPPPRSASAGETPSPTIQRARYPPD.[M]	3936.95871
7780	[R].PAAEAAAAAGGGGATAAAARGGEAAAEVTGWPAGAPGPCASTAEC.[W]	3937.7828
7781	[Y].GLGEHVHQQFRHNMTWKTWPIFTRDATPTEGMI.[N]	3937.90134
7782	[P].GPPGPMGLQGMQGPKGLDGAKGEKGSSGERGPSGLPGPAGPPG.[L]	3937.92834
7783	[R].PQEERPPYHRYKKGGSVGGVCYLSMGMVVLLMGLV.[F]	3939.97404

7784	[L].GPQASQLPCPPVTQPPLHHTPPPASTAAGMPSLQHPAAP.[G]	3940.95852
7785	[Q].PAPAASGPYPSIPAAAADPSMVSAYMYPAGAAGAQAAPQGPAG.[P]	3941.8473
7786	[G].LPAPMDFYRFTIESPSTVTSGGGHHDPAGPGQPLHVPG.[S]	3942.8868
7787	[T].PAGAVPTPEQSAAPACAVSTPEQSATPAGAVPTPEQSATLAG.[A]	3942.90256
7788	[R].LAHTQSPMLQQSQANPAYQASSDMNGWAQGSMGANSM.[F]	3943.67385
7789	[G].VQPSPARSSSYSEANEPDLQMANGGKSLSMGPSPVSPNA.[L]	3944.83892
7790	[G].VIYEMRVYAVNAVGMSRPSPASQPFMPIGPPSEPTH.[L]	3944.91321
7791	[R].PSRESAGVGEGLGDQVPGWPQTVGSREEPLARQGELEA.[G]	3945.93256
7792	[E].LTDSPYVVTSMRIMTRMGTAFLEALGDGEFVKCLH.[S]	3945.93698
7793	[E].WNAANLEELQRNRVSHILNMAREIDNFYPERF.[T]	3945.95655
7794	[P].GFKGEGPEVDVNLPKTDIDIAGPKVDVEGPDVNIEGPE.[G]	3945.9604
7795	[G].RQSRRQSVSRFLDGGTDLKGEAGSRQSTPTASSPTQP.[R]	3945.98739
7796	[I].QQPLETQEVIEITTNHLASPHFTNNSVEIKSAQEN.[P]	3946.94173
7797	[D].KIYSFTDNAPSPSIGGSSRLDSTTPTQPMTPLHVVTQ.[N]	3946.94912
7798	[Q].PPPSSGTPLLGPQPFPGQGPMSQIPQGFQQPHPSQQLS.[M]	3946.95448
7799	[K].VMAQGSIGVAPGMNRQQVSLLAQRLSGGPGSDLQNHVAA.[G]	3946.99743
7800	[E].QEIYFKVCRDNSILPPLDKEKGETLLSPLVMCGP.[H]	3947.01153
7801	[S].PSAADVLAARCMSRAAGTPPPPPAPDPAPLELEPAAEEGA.[V]	3947.92661
7802	[L].SASPGGSAPSRRPSSLRNSPSMYDSPFSFLFLALSGDS.[S]	3948.8821
7803	[L].ISYMSSGPVVAMVWEGPNVVCTSRAMIGHTNSAKAAPG.[T]	3948.88634
7804	[A].EGVGAAANAAATSSTGTGGVAASGMAASGVVPGGGFVASAAAEVQTG.[R]	3949.88323
7805	[Y].SPTSPKYSPTSPTYSPTTPKYSPTSPTYSPTSPVYTP.[T]	3949.89059
7806	[K].QGAQIVSLPECFNSPYGTKYFPEYAEKIPGDSTQK.[L]	3949.89529
7807	[P].PMPPGIMPPMLPPMGAPPPLTQIPGMVPPMMPGMLMPA.[V]	3949.91856
7808	[C].AFAHVEQPPLSDDLQPSSAVSSPTQPGPVLYMPSAAGDS.[V]	3950.87529
7809	[P].GPEGPAGFPGPPGIQGNPGPVGDPGERGPPGRAGLPGSDGAPGP.[P]	3950.91685
7810	[P].PGPEGPAGFPGPPGIQGNPGPVGDPGERGPPGRAGLPGSDGAPG.[P]	3950.91685
7811	[M].LDIVDAIYQMVGNTVELPEEENTPEKRVDRIFAM.[M]	3950.95143
7812	[A].RPPGEGSSTGASPGSPPGAEVEALPEAAALEVAEPPAEALGE.[A]	3951.90942
7813	[Q].NTPAPGVGAAGGSLSFGASSTPAQGFVGVGPFGSAAPSFSIGAGS.[K]	3951.91478
7814	[G].QRNFPLYYTEERRRREMEDERLIQEYLMR.[S]	3951.9341
7815	[P].ATSLPVNNSNSGPNEIANFGRGDVLPPVPGQGDKTATML.[S]	3951.95052
7816	[T].PATPSGSAAFQPPRFPPPAPLYPAGSAGPTQNGFAAHQPP.[T]	3951.95653
7817	[S].SDLPATPEASLLGPDAVSVLSPGPSSGLDPDPGSALGSLPNP.[N]	3951.97096

7818	[S].RSYSPAHNRERNHPRVYQNRDFRGHNRGYRR.[P]	3951.98084
7819	[A].MPPPPMPPGAGGHGPPSAGTPGAGHPGHGHSHPHPFPPGGMP.[H]	3952.81181
7820	[S].TDSLEVSTSSSLTPAMSVSASASTSQASICSSQGISQTVS.[D]	3952.81215
7821	[D].GLTPGHWHPAISVSSACTASGGAGQPPGNHVHHLFAVPGS.[H]	3952.90485
7822	[N].DKVMGPGVSYLVRYMGCVEVLQSMRALDFNTRTQ.[V]	3952.91764
7823	[D].GPVGSTWERGGGAESAQLVLVSTVSFQSAWRHQFSFS.[D]	3952.93652
7824	[F].NGGSISYSQPGLSGPARSIPGYPSSPLPGSPTPPMTPGSSI.[P]	3952.93856
7825	[K].APPLVENEEAEPGRGGLGVGEPGPLGGGGAGGPQMGLPPPPPA.[L]	3952.94979
7826	[Q].LGGDLNSTPLHWATRQGHLSMVVQLMKYGADPSLID.[G]	3952.96842
7827	[T].KAERAPDEAPGSNQRLTKAQCGSWLDTEAAKDEPAGV.[D]	3953.90463
7828	[R].PKGVMMHHSNLIAGMTGQCERIPGLGPKDTYIGYLP.[L]	3954.94855
7829	[G].LPGSRQDGDADARQPSQHSARMEIPPTHYPAARAASV.[V]	3956.91687
7830	[R].PQDMHGPQGIQRHPGPHGPLGPQGPPGPQGNAGPQGHLGP.[Q]	3956.92223
7831	[F].RYGGLLDETKDTQWQRALAYLSAIPELNYMTQIV.[I]	3957.0215
7832	[D].RNSERAQSGAPFSQARGGPPRSGPCGMEIVQTMNSDP.[G]	3961.80864
7833	[C].VQGFGAGTDPPRPAAEAAAAAGGGGATAAAARGGEAAAEVTGWPA.[G]	3961.91758
7834	[R].PGQAPIGNPPVGPIGGMMPPQPGIPQQQGMRPPMPPHGQ.[Y]	3961.94447
7835	[S].AGGGGGGGIGLGSLGGGGGLSSSLGGTATIGHLRGSSEHHFSNTL.[G]	3961.94994
7836	[S].QGFPPDRGGLRPGSLDAEIDSLTSMLAELDGGRGHAPR.[R]	3961.95734
7837	[T].PGSTAITPGTPPSYSSRTPGTPGTPSYPRTPHTPGTPKSA.[I]	3961.96788
7838	[K].KAQEGGGSEVFQELKGICIALGMSKPPANITMFQFF.[S]	3962.94893
7839	[M].RQEVMFLTNMNNSGSSTQIYQAVSRIICGHPEGGGL.[K]	3967.88476
7840	[T].VSEGAVVTITAPVSMNVDSLQSLSSDGATLAVQQVMMAE.[Q]	3967.91847
7841	[C].GQPADKASASGSGAPVGGSISSGSSASSVTVTRSYRSVGGSGGGS.[F]	3971.89255
7842	[G].ADAGGRGAPKDADAAGRPANWAPGRKKKDFSCADRLFA.[L]	3971.97941
7843	[R].AGPGRENMQSVPEGRVDAVGPVGSGEEVPSEPTEPSVPSA.[D]	3974.86724
7844	[F].GRLLNAAEQRGAREAAGSASRSGPGGSGSSGRGGAGVPGPGSGGP.[G]	3974.96363
7845	[P].GPATSPGPARLSEEQRRLVENTEVECYDSLTGTAVGE.[S]	3975.89888
7846	[D].PFLDGDPGVALKPEERKCPYCPDRFHNGIGLANHV.[R]	3975.93812
7847	[I].SQQAQLQEFGQSSRKGEFPGGLMGPVRMISSGHELTT.[D]	3975.94399
7848	[Q].ARPSQNLEAPLGSPRGSLDPPVPRASRGQMLLSGGPRGP.[V]	3976.10461
7849	[A].RQMELEQAVENIEKLTETSTPTAFKAAAPDATEGLST.[E]	3977.96483
7850	[G].GQDDPGDPACPADAVSHGVHGGAGDLANPRGPRTAGESGGAAG.[A]	3978.7768
7851	[A].ADLDFAASLLGTEPFQEEIVAAGAGGSSHGGLGDSSEEEAS.[P]	3978.79993

7852	[I].TSPLPANTPSPSFSKLPPSKASKSSKGKDRLEVEAPSR.[K]	3979.12472
7853	[S].YLEVASDTQLKDSESIPVDGHISLEQPLNGNDTPSLV.[E]	3980.96113
7854	[A].RCYIMNKKPRLAWELYLKMETSGESFSLLQLIA.[N]	3989.08497
7855	[K].PEVTDVSRNTVTLSWQPNLNSGATPTSYIIEAFSHAS.[G]	3989.95156
7856	[G].LGQNISEADLQNQFSRFGEVSGVEIITRKDDQGNPQ.[K]	3989.95877
7857	[G].ERGPAGPPGPQGPPGEQGPEGIGKPGAPGTPGQPGIPGMKGHS.[G]	3989.96751
7858	[F].AGTFSPAGSVEGSPMHTMYMSQPAPAASGPYPSIPAAAADP.[S]	3990.7983
7859	[W].ARDAMSLSGRRGSKGHGPGMARKFSAPGQLCVSMTSNL.[G]	3991.95822
7860	[S].LKTSLQPMVSALNISMGGTGTFTVRMALFQSPAYTQP.[Y]	3991.99661
7861	[R].GPPGRAGEKGDVGSQGVRGPQGITGPKGGPPGIDGKDGTPGMP.[G]	3992.99953
7862	[P].AAQAGMCTRSQRVRGGPQPGAEGPRRPGRALTPGGGGSPH.[P]	3993.00156
7863	[Y].IAPWTSETPPEGNPAPSPTSGSTQTPSTSTSSNTVEAHVS.[E]	3994.84247
7864	[P].NGERPLSSTGPSQHLQAAGSGIQNQNGHPTLPSNSVTQGA.[A]	3994.93502
7865	[S].KSHPDPGSPSRDTVSPALPTGESPRWVQEQEALLGPDG.[-]	3994.95296
7866	[V].IITGSQDGMVRGPPGVEERTGPEPADRFVAPAHGMFSI.[L]	3994.95383
7867	[P].KPALAQAQDASRPSSPQATTPNPVPSSTEAQGVAGPAAEIP.[A]	3995.01048
7868	[L].PWLDGGKAALSAAAAHHHNPWTVSPFSKTPLHPSAAGGPG.[G]	3996.00521
7869	[N].PQRRYNPSSHVLSGACAGAVAAAATTPLDVCKTLLNTQ.[E]	3996.01783
7870	[P].GPQGRPGVFGLPGEKGNTGVPGRGRASLGDAGQRWPEAPI.[H]	3996.06994
7871	[L].FHPERVRAVASLNTPFMPSNPKVSSMEIIKANPAFN.[Y]	3997.05751
7872	[D].TATVEVRPGTRWASGPAFVGLACEPGLGGDLPLGLFAGGSG.[T]	3998.0229
7873	[M].DGVAGEHTQIPNGSRSHEPLSVDSVSSNLAAETVGHGGVM.[P]	3998.88971
7874	[C].PFIVCMSYAFHTPDKLSFILDLMNGGDLHYHLSQ.[H]	3998.89141
7875	[G].GLGGPGGSVPFKLEENYDNFFTVVTDRPLDRETQDE.[Y]	3998.90428
7876	[Y].QIVLDAATTGMSYTQLFTIARYMEHRGYPMRAYK.[L]	3998.97139
7877	[L].GPPDPRLAMGSVGSGVAHTQEFAMKSVGTRTGGGGNQGSFP.[G]	3999.91884
7878	[P].AHVDAAAGAVPPSPPAAALGATCAAAFPSAASVTSAGATSASSVH.[L]	4000.94577
7879	[P].GPSGPLGHPGLPGPMGPPGDPGIQGYHGRKGERGMPGMPGK.[H]	4000.94797
7880	[G].PGALWLETQMVGLGPCVLQQHFLHSFMEPAVLFTM.[V]	4000.96208
7881	[P].TAPPTVCVTGPPTARPSEGPTTGPTGPPAAGPTGPPTAGPSAAP.[T]	4000.97092
7882	[L].GTPAHRTEERSAAPPDLRHCFYRGQVNAREDHTAV.[F]	4001.92843
7883	[P].KLGGSLGTGAFGAPGFNTTTATLGFGAPQAPVALTDPNASAAQ.[Q]	4003.01958
7884	[G].PGSRISSSAFSRVGSGSSFRGGLGTGMGVAGSYGGAPGLGGITA.[V]	4003.9679
7885	[C].EKNIIMGYKTLCFGLTCILFAYYIYTPIPENIE.[E]	4003.99342

7886	[A].GHHGDQGAPGAVGPAGPRGPAGPSGPAGKDGRTGQPGAVGPAGIR.[G]	4004.00946
7887	[A].SRDGSLTSNPYSGDVTKFGRGDSASPAPPTTLAQPQQSQ.[S]	4005.9173
7888	[Q].KSKSMPNLGDEMLSPVTLEPQQNGLCPQRRFSIES.[L]	4005.9467
7889	[L].VGAALCITMLGLGCTVDGNHFAAHVRRPVGALLAALCQ.[F]	4006.03943
7890	[E].GTSILEEASPAGMPIQQELDSPAFGVKEVTGTVLHGKVP.[L]	4006.04777
7891	[A].PSMPVAPPATPAPRTCDGSPRGTPTSPAPEGGEGPPSPLSE.[A]	4006.89095
7892	[F].KGMSVSRSQGAIASDTLPDMSRGTLATGMFLSMSQVSPG.[R]	4006.8977
7893	[G].QAPCDLLSPPLSSAPPTIWGSNETSEVAVMEGHPVWF.[L]	4006.899
7894	[K].EMQVIVEDRNDNAPVFQSISFSANVSEGSEYLFRI.[L]	4006.91274
7895	[T].VSMGSAVFAGVAMTNLPGILVLGLAKAQLIQIFFFRLN.[L]	4007.2377
7896	[Q].GPAGHPGEAGLPGPSGNMGPQGPKGIPGNPGLPGPKGEMGPVGP.[A]	4007.94908
7897	[M].GPPGLPGPMGIPGSPGHMGPPGPTGPKGTSGHPGEKGERGLQG.[E]	4007.96031
7898	[Q].QGVQRGLNMSMCHPGQMSLLGRTGVPPQQGMVPHGLH.[Q]	4008.93465
7899	[T].AAYPAGPPPTQSATAAANNNFVNFGVADLNAVQSPGIPQGN.[S]	4008.94748
7900	[A].SYFLSRAQSLCGPERSAVPDSLRWLCHPLGQKFF.[M]	4009.99524
7901	[K].IVQAEEALSKNPGYIKLRKIRAAQNISKTIATSQNR.[I]	4010.26215
7902	[I].QGLGSREDLLSEVSGPSPPLARASSFWGRSSTQAQQHS.[R]	4010.97034
7903	[K].AYGYTLDERYIPIVGAKHADFVNSELKYKETYEK.[L]	4011.01746
7904	[P].GPKGDQGPPGPRGHQGERGLPGLSGSGSSSLGLNLQGPPGPPG.[P]	4011.01796
7905	[-].MSRQSTVSFRSGGSRSFSTASAITPSVSRTSFTSVSRSGG.[G]	4011.98672
7906	[L].ATTASPAPEEQKAQAAPEEEAATVAVPEKGVGNGRAPDVAP.[E]	4011.98941
7907	[Y].NRAGTGPASPSANATTMKPPPRRPPGNISWTFSSSSLSI.[K]	4012.00937
7908	[N].GPILTLGVSPSGSSIQEASNVATQQFSGTDLPNGPLASSLN.[S]	4012.01456
7909	[A].TKSLQQGMVSFGPESPIQAIEPPSSVAAAAATSIVAAGQSQ.[A]	4012.03318
7910	[R].LASAESTVGSKAALERRLLQVQEILLMKGEGEVKLNM.[A]	4012.19333
7911	[A].GARGGAEAASAGATGAAKGGPGRWLLLGTGPIFRNFIIFLI.[F]	4012.20318
7912	[Q].GREAQPVLPGPSEFQGDPAPRGPPGRLGRKALLARKVH.[K]	4012.22163
7913	[K].SKSNNWAPKYNETFHFLLGNEEGPEAYELQICVK.[D]	4012.91743
7914	[H].QPHCPTASGNRASPAAASAVPGSGAAAGALASGGSKEEFVATF.[K]	4012.92062
7915	[F].SGEFLPRGPSTPDLPEVEVAAGGSEAPLQDGGQAVCFSAL.[G]	4012.9233
7916	[E].IESEALQPSAEAVAANPGAMLELGPPHGVSAEEAGLGPQMA.[G]	4012.92667
7917	[K].RCRGSCKKGKCESSRRGAAAAEPGPHAREELHHRD.[A]	4013.93249
7918	[A].ARSPCSVPSASVEPWSLTELSLLMGSGLTPALGSDLSPQ.[R]	4013.98346
7919	[S].PSSMNQRRLGPREVGGQGAGSAGGLEPVHPASLPDSSLAAS.[A]	4013.98461

7920	[L].EGTAGLPGPPGPRGFQGTAGARGSHGERGPPGAVGPTGLPGPKG.[E]	4014.04412
7921	[F].QLHTTLAETPAGSLDALCSAPSSVATTQLGPYAFKIPLS.[I]	4014.05286
7922	[Q].GTARLGNALGMIGVAGGLAATLGGLKPCPELLAQMSGAMALG.[G]	4014.06431
7923	[V].VTNGSQPAAPHNNTHPRPPGSPGSPLLRSLYVVTGLIVL.[A]	4014.16719
7924	[I].PGAPGAPGFSGPKGEPGDILTFPGMKGDKGDLGSPGVPGLPGL.[P]	4015.02698
7925	[Q].SAPGSWRRAHGIHSLLPEPGERMRIQAEGPGRGPEPL.[S]	4015.05799
7926	[I].KHFHEAYPGCVVTRVHFCYDVRNLIDLDDQRR.[Q]	4015.9555
7927	[S].GLNDAKPEPRPDPAERREPPPSQELPGSLGAVELSAAAP.[P]	4016.0472
7928	[T].ALGSGRLGARGGEGVQVGGAAGVTHRSQVAEGRHTGHRAAAP.[I]	4016.08944
7929	[L].YSPKEPPNGNAFPPFHPGTMLDRDVGPTPMYPPTYL.[E]	4016.89861
7930	[D].KGKGPPWADCGGTVAQPMSPAPGPADPGPGPEGRAPHSAIE.[E]	4017.89704
7931	[Y].VACVPDDSSGAVALVAFSAAPEGPLAPEACGALCFAAGQTL.[A]	4017.9031
7932	[N].KIMHLSTSNLSMGEMTAGQICNLVAIDTNQLMWFF.[F]	4017.90397
7933	[G].GGGERTPAPGALEPDAAATRAAPNPASLPNTLGSGYSPRLC.[P]	4017.98355
7934	[R].ARSHSTPLPPQGSGQPRGERELPNSHSMICPKAAGAPAA.[P]	4017.98826
7935	[R].RQPCKEVTAAPAVCNGVLQGILTFADGCVLRADVGIY.[T]	4019.02997
7936	[S].RQPGLAQPLMPTQPPAHALQQRLAPSMAMVSNQGHML.[S]	4019.03468
7937	[A].RQPLWHMLLADALAAVGSFEEAGAHLQKVLHPSPPSE.[A]	4019.05962
7938	[S].QSVHGSLETGSRCKRSVAGATEKAGVEKAHFVPKPKKG.[L]	4019.13557
7939	[G].SPEAPHPVPGGGEGPPKTGTAPSPGPPCPPVDGTSEGKGARH.[P]	4020.9257
7940	[A].GPGSPATLSPSAGVPQPVGMEALDQAEGPAASQRAMPPPPPA.[S]	4020.94299
7941	[M].VRTQTESSTAPGVPSGGSRQGPAMDGTAAQPRASASALQHT.[A]	4020.95404
7942	[-].MVRTQTESSTAPGVPSGGSRQGPAMDGTAAQPRASASALQHT.[A]	4020.95404
7943	[G].SIPDTPKSADASQDSLSSSPLPLEMSSIAALENQMKMI.[N]	4021.92904
7944	[E].LEGGPYSPLGKDYRKAEGEREEEAGVPDHSPRSSQVP.[K]	4023.94312
7945	[Q].AAGVAADWAAAGLADGARAAGHAGHGPAHGGLAGHGAAAAGVAVETG.[L]	4023.96693
7946	[S].WGARHLEPASTAGAHKGWWTLRRECSQPLTHWPGS.[G]	4023.96845
7947	[S].APAAAPFTSSSAANGLESSVATDSSKLATITTPMALNTSGID.[V]	4023.97031
7948	[C].EGAKDGFPDRKSGNTLTKGCHDSPLLLSTGGPGDPESLI.[A]	4023.97165
7949	[L].PAPAEVAARSQALEDDATMRTADLAEKRGPSSSPENLR.[K]	4023.97886
7950	[A].KETSNMLPTAPFKSQNAPAKGPNTAYDFSQAAPTSELT.[A]	4026.93895
7951	[D].PADGPPDLTGWLHSLAGSTLPGLLRQADPQHVMEYSLA.[L]	4027.00183
7952	[G].QSSHLAGPNGERPLSSTGPSQHLQAAGSGIQNQNGHPTLP.[S]	4027.97174
7953	[I].PGGPPSSPGSRKLSAAGSSDGVMPVAPTSAVSSSGSPASVMTSI.[R]	4028.95395

7954	[A].GFPGPPGIQGNPGPVGDPGERGPPGRAGLPGSDGAPGPPGTSLM.[L]	4028.96717
7955	[S].PSSFSPSATPPQKYSSRSNRGEVVTSFGSAQGVSWSGRG.[G]	4029.94379
7956	[Y].QSQHPHVYSPVIQGNARMMAPPTHAQPGLVSSSATQYG.[A]	4029.94466
7957	[Q].GPPGPQGHLGPQGPPGTPGMQGPPGPRGMQGPPHPHGIQGGPG.[S]	4029.946
7958	[E].FVFSKDSEASGQRVDGLAFVNEDVVASKGSGPGTICLW.[S]	4029.96511
7959	[Y].RNHLEASAPSTVSPDALSPGPVPSPPPPEKESPQEVAEE.[V]	4029.96761
7960	[A].VSTPEQSATPAGAVPTPEQSATLAGAVSTPEEPATPAGAVSTP.[E]	4029.9775
7961	[Q].GPRPDGVNGPASLLRMEASCLELALEGERLCKAGDFK.[A]	4029.99431
7962	[R].PPGSGPGPGPATGAKTEPGSGPRAQARTGGTTSPKHGRTEHQ.[A]	4029.99862
7963	[M].LSTHSSPYKTLERRPQAGRSMPTTPVLTRNAYSSSH.[L]	4030.03117
7964	[L].QQPPQGSTVQHTYLPNTWNSFRGYPSEIQMMTLPP.[G]	4030.92147
7965	[T].PAAEGVGAAANAAATSSTGTGGVAASGMAASGVVPGGGFVASAAAEVQ.[T]	4030.94108
7966	[S].AHEGLGSPRGPPNSLSQRQFPGQAYGSPGASGVGTSPGPRD.[T]	4030.95028
7967	[M].LNGEVYPPSVEEAPVLMRYPEGIPPQSQMAVGQEVFG.[L]	4030.95651
7968	[L].PANASLAAMAAAAGLNPGLMAPSQFAAGKDRSPFCPASSPK.[S]	4030.9572
7969	[S].PGSQSKEGSPVSKMSVSRSSSLRSSSSLSSQGSVASSIGSQ.[T]	4030.95818
7970	[I].QGGPGSQGIQGPVSQGPLMGLNPRGMQGPPGPRENQGPAPQ.[G]	4030.97227
7971	[G].RNTTPCKEVDIEGTTVIEVGLDPSNNMTLAVDCVGIL.[K]	4030.97699
7972	[D].ASVSVPSEGLPQGTSSAPQAPAHPTGASESIVSQAEEKAVAT.[P]	4030.98399
7973	[E].AATKYFLTQSTASMLLMMAVPRATEAATKYFLTQSTA.[S]	4031.03266
7974	[P].PAAALGATCAAAFPSAASVTSAGATSASSVHLPVSAPHGAGLMAA.[A]	4032.99061
7975	[R].PHNRNSGQLEPGPAGAPSPAPGLPGPRRRASPADLVYFE.[K]	4033.05395
7976	[S].LGTYLQGMASTLSPSQEPPPPPGSQVPPASPSSQEPESGQ.[A]	4033.89715
7977	[G].GVQEAHFDGLIFVHSGIYTDEWIYIESPITMIGAAPG.[K]	4033.96807
7978	[A].GRAHQTPQQQGPPIPGAPSEPGLQQLSYTPSQTPTPPST.[P]	4034.00025
7979	[P].GLKGDQGVPGERGPAGPPGPQGPPGEQGPEGIGKPGAPGTPGQP.[G]	4034.01148
7980	[V].LFPFSLSRWDSELGLPHLSASGCPRGLGPEGLPGRSSP.[P]	4034.03413
7981	[V].LSSPSCLKNLSTGTSALGSMSPTTSSQILARKRRRGVI.[L]	4034.15974
7982	[P].PGPPGPMGLQGMQGPKGLDGAKGEKGSSGERGPSGLPGPAGPPG.[L]	4034.98111
7983	[F].NAIGSGPWSQAVMGRTRESVPSSGPTNVSVLATTSSSMLV.[R]	4034.991
7984	[I].PGSVIDATMFNPCGYSMNGMKSDGTYWTIHITPEPE.[F]	4035.75439
7985	[V].DVAVPDVDVHGPDWHLKMPKMKMPKFSMPGFKGEGP.[E]	4036.95805
7986	[R].KDEPSPLNLSHHNPELQGGGRGPARSTAEMAEKNQARG.[V]	4036.97544
7987	[R].RASDSIFQSKNLNFPKSSPWESEFGQPALGNKNIQD.[S]	4036.97878

7988	[K].KADIGVAMGIAGSDAAKNAADMILLDDNFASIVTGVEQGR.[L]	4036.99542
7989	[E].FQPVECAIVVNAAGAWSGQIAELAGVGNGPPGTMQGTRLP.[V]	4037.00078
7990	[S].QRKEFAEKLESLLHRAYHLQEEFGSTFPADSMLL.[D]	4037.02256
7991	[I].TPYSLDIKNTCTSVYTKDKAAKCRIPALELLIKLL.[Q]	4037.21776
7992	[G].QPSTNTMGLFGVTQASQPGGLFGTATNTSTGTAFGTGASLFG.[Q]	4037.91855
7993	[V].HGLAMAPASVAPAPAGSGAPPGSLGPSEQLGQAGPTVGPQQQPP.[A]	4038.01379
7994	[P].GPQGQPGLPGTPGHAVEGPKGDRGPQGQPGLPGHPGPMGPPGL.[P]	4038.01512
7995	[V].QQQIAMGAQPPVAQVMPGAQPIAWGQPGLFPATQQPWP.[A]	4038.01531
7996	[P].AAPAPFPPGPPMMPPPFMPPPGIPPPFPPMGLPPMSQRP.[P]	4038.01636
7997	[L].GLGDAAHSSFSANADLSFSAVPCPRPSPAVDPPTPGPGHPT.[H]	4038.9039
7998	[A].AAGPAPSQAGAGSAPSSVESAASSGPSPSPGSAPSAASPATPSPPAR.[L]	4039.92278
7999	[P].DMLRTALGESTASLDSTVRDEGGQKLSVSSGPARGGHGEP.[D]	4041.95304
8000	[S].RVGPHNMDLKAEESPFVLTDDLTDQSIREEGVSMLP.[G]	4041.95322
8001	[G].PAGPPGPQGPPGEQGPEGIGKPGAPGTPGQPGIPGMKGHSGAPGP.[A]	4042.98282
8002	[L].APNVTTPSSPPPTTTTVPVSPTTRTPSPVTTPAPDICGSR.[N]	4043.039
8003	[G].PCMLELRPSLPGSEEPQPQGQRGYGREGSSRTSLEGT.[S]	4043.9298
8004	[M].SRGPQDVSRTSPASVEETSSPSSPVTLSAMTSPSPESPTL.[P]	4043.93499
8005	[P].GINQTYRSPLGSTTSAPAPSAPPAPPAFHGMLERAPAEPS.[Y]	4044.98724
8006	[G].VPSSQPMQLSQQQQGVQPTAPSPQAAQYPLPQASAPSEA.[A]	4045.956
8007	[G].TKFSRSGHTSFFDKGAVNGFDPAPPPPGLGSSRPSSAPGM.[L]	4045.96136
8008	[C].LERTDVCHEVEPVRTLAEHSYAVSAVSSVAEAYRSQ.[P]	4045.96723
8009	[T].KNMHLHKASDMEVLSQEIVRLSKECVGSPDPDLEPG.[E]	4045.978
8010	[L].QPTAHSPAGNQVQAGKQSHIPYSQQRPSGPGPVTQGPQQ.[P]	4045.99756
8011	[A].VSAHMGVLESGVWGKSRDEGLPLATNGGGPAAGGRGGRGQEA.[W]	4046.00093
8012	[Q].HPRPRMTPQNRGSQEPRPEGAADGPAVPAAERRTEDP.[N]	4046.97103
8013	[E].AGLDGAKGEKGVQGEKGDRGPLGLPGASGLDGRPGPPGTPGPIG.[V]	4047.10063
8014	[P].AAAAPLSQDGPQAEPQAPGRPPASGLAAAAEESEPPRELEN.[R]	4047.96425
8015	[T].FTASGSLEKMLSTHSFGWQLKICTIHETSLTMNFL.[H]	4047.96531
8016	[G].PEGPAGFPGPPGIQGNPGPVGDPGERGPPGRAGLPGSDGAPGPPG.[T]	4047.96961
8017	[E].GVDQRGSSPTSHPRPGAPPSASSAASRAPPESSAHRLSGGPS.[T]	4047.9728
8018	[G].GPSSIVGFGSGLSTDAGFNGGPSSSAGYGNGLNNAAGFGGGATSLG.[A]	4048.85437
8019	[D].ARLSSVADMTAADQSPLAPLLETLEDPSASVGEQTDAYL.[T]	4048.95433
8020	[F].GKGLSNWRPSGSSGPSQPGQPGAGTVLAGASGLQQVQMAGAPS.[Q]	4048.98936
8021	[F].FGTILFMYLRPSGSYSMGQDKAVSVFYTAVIPMLNP.[L]	4048.98973

8022	[G].PAAHCLREASRAYAQAGQPQASALALGAAAGCMLKSGQHG.[V]	4049.96033
8023	[L].DNLDQICLPSRQHVVYGPWNLPQTGFSHLSRQGET.[L]	4049.96751
8024	[A].QPSLAQWGKESPAPDTAPSWAAAGNREAIQELESSLKE.[V]	4049.98393
8025	[F].QVGMKLEAVDRMNPSLVCVASVTDVVDGRFLVHFDN.[W]	4049.98817
8026	[K].IDFASLDPLNIKQNNKQEGARPDPSPAPGLPAASAPQQP.[S]	4050.06793
8027	[P].AAPAGATLSTAPQQPLPPVPQQYQVPGNLSAAQVAAQNAVE.[A]	4051.08833
8028	[A].EPGPPGPPGPPGPMGLQGMQGPKGLDGAKGEKGSSGERGPSGL.[P]	4051.96004
8029	[F].CVLNADARAAWTPACLGRKAAPEEARPAPGTGPGAYNNT.[A]	4051.96137
8030	[M].ASSVLMAQQKQPVVYGDPFQSRLDFGQGAGSPVCLAQV.[K]	4052.00045
8031	[I].KDGKFGAYMQVHIQNDGPVTIELESPAPGAAASDPKQVS.[P]	4053.00222
8032	[G].KMAPGEHQAQWVPKEKLDKMVQRSGPSGPPGPKGDDGI.[P]	4053.04331
8033	[T].TQPSALQAPGGAAPAPSSALPGTAQLPTPGPTPAPAAPSQGSPLA.[S]	4054.088
8034	[F].PVSEELEADLASGDQSLPMGVLGAQSERFPSNLEVEASP.[Q]	4055.93901
8035	[S].SILYEFSTLQALEMEHKGPTSEKVATDSWSTLDVGSV.[L]	4055.97942
8036	[F].FAAPQPGPGATAASKPNSTVPKGEAKPKRRKKVRRPFQ.[R]	4056.28423
8037	[E].EKSSKAESGEKSKKRKKRKRKKNKSSAPADSERGPK.[P]	4056.28608
8038	[D].AAEALDLSERLYMDMSKTLGSLMNIKDMSGHVSMKH.[L]	4057.91599
8039	[G].PGSHRMEMLNRLPFPPGAPEWQGGSQGAPGAMGKTAGPGE.[K]	4057.92182
8040	[H].GAPDADTGQTAVVTSERDLGGGGQPPPWASASAAGAQEQPPR.[D]	4057.92345
8041	[G].PTSALGTTPTPASASPEGLKEESGDLAASPASPGSPPNSDLVP.[L]	4057.97242
8042	[P].KGEMGPVGPAGNPGAKGERGSSGLDGKPGYPGEPGLNGPKGNP.[G]	4057.97846
8043	[H].PSAAGGPGGPLSVYPGAGAGGGGGGSSVASLTPTAAHSGAHLFGFPP.[T]	4057.97912
8044	[F].PPPSPPPTPMPRPADGETGGPRKLLQMDSGYASIEGRGAG.[D]	4057.98586
8045	[G].PGGQPIDANHLNKGIGMGNLGPAGMEGPFGGGMENMGRFGS.[G]	4058.83643
8046	[V].PQSGQSLPGAGVMPTVGQLRAQLQGVLAKNPQLRHLSPQ.[Q]	4059.20326
8047	[Q].QRTTMILLCEDGSLRIYMANVENTSYWLQPSLQP.[S]	4059.96128
8048	[K].PASSGPLSHPAPLSASSSSLSSKSSVTPSASGRAAQGSPSPVPS.[M]	4060.02177
8049	[L].HLSSFTPQSNSPVKVECAAPSAHARLCSVGHPVFQMGA.[K]	4061.95312
8050	[T].IDTTMRGGRLGVFCFSQENIIWSNLKYRCNDTIP.[E]	4061.97827
8051	[D].SHATMKSLLASKPEDATHGRRSRGMVQSSSPVMPTAVGP.[S]	4062.043
8052	[H].KLYEKCEVVMGNLEIVLTGHNADLSFLQWIREVTG.[Y]	4062.08272
8053	[E].AADAIDAEGASAPLMELLHSRNEGTATYAAAVLFRISED.[K]	4062.97131
8054	[D].KLADPVKICSSATETYKVLQEHMVLQNEVVAQASIY.[S]	4063.08786
8055	[D].TAEGKDKDLEPEDRGLEHRDTAPEQKDQALEGKDKD.[L]	4063.94391

8056	[S].TMVTSITTGTTATQVMANSAGLNFINVVGSVCGAQALMSGS.[N]	4063.94431
8057	[E].ESSLLGKDSPPTPTMYKYRPGHSSSASAAMPHSSSAKLS.[R]	4063.9488
8058	[L].PQTAEEIQNTLTSPQFQQALGMFSAALASGQLGPLMCQ.[F]	4063.9562
8059	[Q].QQTPVASIQQVASASQQASPQTVTLTQATAAGQQVQMIPA.[V]	4064.0717
8060	[E].QRAPAAESQAPFPSGSPTLPAQAQAGQMMPLSSARPTSGSV.[G]	4064.99167
8061	[V].AGLQQAPFSEPPAPAYVLPEPGFPPTDPSQVQSPSLPPGP.[A]	4065.02802
8062	[L].GAAAGSGGAAGFAATTGLSNFSMGQSGTMRTRHSTGGTNKDFG.[E]	4065.85261
8063	[L].QETGAIVAMTGDGVNDAVALKSADIGIAMGQTGTDVSKEAAS.[M]	4065.95909
8064	[P].QVQQLMSGMISGGHNPLGTPGTSPSQNDLASLIQAGQQFA.[Q]	4065.97569
8065	[G].VAHFSHEEKHVGSGKLSEMQNQRRSHAFFQDVQKA.[S]	4065.98489
8066	[K].NPQKYSDKELQYIDAIGNKQGELESYVSDGYKTALT.[E]	4065.99276
8067	[P].TSETTTLTSSIAPPVAATPDSNSVPAGQATPSKGSGVSAASPAP.[A]	4066.00987
8068	[A].AGVAADWAAAGLADGARAAGHAGHGPAHGGLAGHGAAAAGVAVETGL.[E]	4066.01388
8069	[R].PGRTNVQQTTDLEIPPPGTPHSELLEEVECTPSPRLA.[L]	4066.0186
8070	[N].MTSPRSNAGITTVGNTIYAVGGFDGNEFLNTVEVYNLE.[S]	4066.93387
8071	[T].PPAMPASPPGSTNTWTGVTTRASSSAHEGRLSDPQLGGPGH.[M]	4066.94241
8072	[P].RNTTSVDSGAPGGAAPGGPGFRAFLCPLICHNGGVCVKPD.[R]	4066.94328
8073	[P].KGETGPQGYKGMVGSIGAAGSPGEEGPRGPPGRAGEKGDVGSQ.[G]	4066.96354
8074	[G].ESRSQPPRFKTGGPGDELGAPDPPGAAPPGPGQTAEGRRGD.[A]	4066.9714
8075	[I].VSCLIDLDEGTLSFSLNGVSLGTAFENLSRGLGMAYFP.[A]	4066.97764
8076	[L].QTGAAQICARPDPFQQALIVCPPGFQGLQASPSKHAGY.[S]	4067.00145
8077	[N].QGVAPMVGTPAPGGSPYGQQVGVLGPPGQQAPPPYPGPHPAGP.[P]	4067.02323
8078	[T].STEPSTAKPSSPEPAPPEAMDTERPGTPVPPVEVPELLD.[T]	4067.96416
8079	[P].GPPGGPGMPPGGRGRGRGQGNWGPPGGEMTFSIPTHKCGLV.[I]	4067.96502
8080	[Q].PATTPTGSQPPSQHAAPSPVQHQAGQAPHLGSGQPQQNLY.[H]	4067.97068
8081	[P].QSPPTCRVSPPESRGTQSLLPSDSPQPLAASPSPWGPEA.[V]	4068.97198
8082	[G].KLGCDLDMFLDLDEIGKFTAQKTSGNFLMEFQVKN.[V]	4068.97554
8083	[I].FFASSRSARPFSGFDPDDMDVDEDDDPFGAFGRFSF.[N]	4069.7246
8084	[A].QPATVVSSIDSAHSDVASGMSDGNENVPSSSGRHEGRTTK.[R]	4070.87043
8085	[S].ASASAGPSASASAGPPASARPGTSAAARAATSASARADMSATARPG.[P]	4070.96567
8086	[G].QPGTGKTAIAMGMAQALGPDTPFTAIAGSEIFSLEMSKTE.[A]	4070.97593
8087	[I].ASPSTAPLVFQGHLVPWAQKASLDGMGQRGAKDLGTLEH.[K]	4071.08689
8088	[G].QKGAAHPDPSQSSVDTGPARRPEDPGGPESPRMPESEDS.[T]	4071.83332
8089	[T].VLPAAGRSGESEGPREPDAEADAAAAGLAGWPGAGPSPASFPQ.[T]	4071.94312

8090	[P].EQSATPAGAVPTPEQSATLAGAVSTPEEPATPAGAVSTPEEPA.[T]	4071.95168
8091	[Y].RQLICYPQEVIPTFDMAVNEIFFDRYPDSILEH.[Q]	4071.96193
8092	[T].QRCAAHSSAFLSTTLSALDEALHGGVACGSLTEFHLEK.[M]	4071.96512
8093	[A].ATTMEATSREAAPAKSSASGPSAPPALFELCGRAVSAHMGV.[L]	4071.96849
8094	[R].AAPWAAATSSPPPSADEEGMLLAGGAMDADSSAEACASPEPP.[Q]	4072.73688
8095	[W].NGVAPKHQSSLPPQAKNLKKPRPTPASRPDEASASSNLP.[K]	4073.1639
8096	[V].AAATTMEATSREAAPAKSSASGPSAPPALFELCGRAVSAHM.[G]	4073.94776
8097	[F].MSQNITPVNESVVSASRTVNMFSGTGANVSLGSVSVTSTSA.[S]	4073.97541
8098	[E].AEALEGPVAMVAQDTSDIVFLGLCILAGVLMVIAIVVLM.[L]	4074.16492
8099	[P].GPSPGPGPSPGAMLGPSPGPSPGSAHSIMGPSPGPPSAGHPIPTQ.[G]	4074.94366
8100	[S].FISLLGTMTTCSRQYTSSSSIKGSGGIGGGSSRMSSVLAGG.[S]	4074.9529
8101	[A].VFSFGSSSLKSSGSVPGEAPPSSSGSDGAKAALASGASPFSFAP.[P]	4074.95671
8102	[I].PAMVGDYIAAFEAISPEVLRHVINMADGNGNTALHYSV.[S]	4074.96881
8103	[P].GRALACGSNRFNKLGLDHLSLGEEPAPHQQVEEALNF.[T]	4075.02027
8104	[P].QPASGSPTPAPRRPGAAGPAPHPQWAGQPSVLDSINPDRH.[F]	4075.03937
8105	[S].ASGLPSEDLDSSGLTSTVGSGLPVESGLPSGEEERITWTSA.[P]	4075.9466
8106	[L].AAPSSPSLSHRQGMGPLGTGFHGNTVSSPQSSAATTPGSPSLG.[R]	4075.95264
8107	[G].GKPPNSAQTAEIFQALQQECMRQLQVPAGSLVPSPSPGG.[D]	4076.03281
8108	[S].GFGTPRLTSRSSSMVSLEDGEKGPAPRGSTTDSLGSQLLP.[E]	4076.03531
8109	[L].QGQKGERGPPGESVVGAPGAPGTPGERGEQGRPGPTGPRGEK.[G]	4076.04049
8110	[M].APGCEPLAVRRMMDVLAPHVHGQSLAGAGGGGFLCLLTK.[E]	4076.04491
8111	[S].QAGMLAPGTRRDIYDQKLTLQPVDNSTISLQMGTNKV.[A]	4076.09032
8112	[R].ETSFIHAISSAGVMYTITKNCSMGDFENCGCDESKN.[G]	4076.70751
8113	[H].LPSSPAPSGTAPSGPAASRAHLTPPCSPGAPAEATPHNPRPL.[R]	4077.03592
8114	[I].SSYAKVSICLPMDTETPLALAYIILVLLLNIVAFIIV.[C]	4077.30338
8115	[E].PSVIGHMSPITTSPHSPGASGNMERITSPVLMGEENNVV.[H]	4077.93144
8116	[-].MVRTQTESSTAPGVPSGGSRQGPAMDGTAAQPRASASALQHT.[A]	4078.95952
8117	[V].SVGPAVSSGVNVNILSGMGNGTIASSAALNSAASAAAGMTVGSVSS.[Q]	4079.00196
8118	[V].SPSLEGRGNGSEDEMRFREVVMERMSNMEKRIQY.[L]	4079.87903
8119	[P].SGPENSSQWLDDLLASPPPSARRGAPSELKDAQTPSACS.[E]	4079.93632
8120	[A].AAAAQMHAKNGGGGSGSHRSSPVPGAPAVCEPLAVPAASPMAAA.[A]	4079.95966
8121	[Q].VEPEGQEKPSPATVRSTDPVTTKETKAVSEMSTEIGTM.[I]	4079.96351
8122	[S].AAAEEPRSQTHGPMVDHVTLEVALSSQVLKELTVLLPGG.[S]	4080.1434
8123	[A].AADAMSVLGEYERHCDSLNSDFGSESGGGGDSGPGPSAGPAP.[R]	4081.70467

8124	[A].QAQDASRPSSPQATTPNPVPSSTEAQGVAGPAAEIPASGGHGT.[E]	4081.94458
8125	[T].APSLFGQQTGSNVSTAAAAPQVSSSGFGSPAFGASTPGVFGQPG.[F]	4081.95263
8126	[A].AYPAGPPPTQSATAAANNNFVNFGVADLNAVQSPGIPQGNSG.[V]	4081.96386
8127	[N].GLLPSAPSAASNNSNSLNVNNGVPGGAAAAAAATVAAASATTAASSS.[L]	4082.0021
8128	[P].PGPSGPLGHPGLPGPMGPPGDPGIQGYHGRKGERGMPGMPGK.[H]	4082.00582
8129	[S].PNMLITYDDVVKISDFGTSKELSDKSTKMSFAGTVAW.[M]	4082.01369
8130	[N].GLLSTPSVNGGGGSVLGSAGSGGGPVGSVENGKPGADVVDLTLDSS.[S]	4082.01601
8131	[N].KVSEHVGSRTQDECILHFLRLPIEDPYLEDSQASL.[G]	4082.02877
8132	[T].IFTFGMKIPSGLFIPSMAVGAMAGRMVGIGVEQLAYHH.[H]	4082.05229
8133	[R].PGWFDIGKQDGVITVNSPLDREQLLEEDEEVQVQVT.[A]	4083.01931
8134	[L].LHGGRKMPDSDAPPNVTVSTSTIPLSMAATLQHSQPPDL.[S]	4083.02739
8135	[A].SKGTEPHPTPAFPPGPYATPPGYGAAFSAAPVGALAAAGANYS.[Q]	4083.98756
8136	[R].GPAGPPGPQGPPGEQGPEGIGKPGAPGTPGQPGIPGMKGHSGAPGP.[A]	4084.00937
8137	[T].TSAHQGVTTPPKFPLENMINTATSQPATSIGDITSSVTPA.[T]	4084.01793
8138	[A].MAPASVAPAPAGSGAPPGSLGPSEQLGQAGPTVGPQQQPPAGAPQ.[P]	4084.01927
8139	[R].DMIGIAKTGSGKTAAFIWPMLIHIMDQKELEPGDGPIA.[V]	4084.05921
8140	[H].LPANFEQVCVPLIQSTISIDSNVSPQGSSSRVATTPGLN.[P]	4084.06555
8141	[S].PPQGLMTQQNFMLMKQRGVGGEVYSQPPHMLSPQGSL.[L]	4084.98639
8142	[T].GPSQHLQAAGSGIQNQNGHPTLPSNSVTQGAALNHLSSHTA.[T]	4084.9932
8143	[L].GGYVAPGYPLQLQPCTAYVPVYPVGTPYAGATPGGTGVTST.[L]	4085.00009
8144	[S].QPGTPSPLDGPRPFPMAQGRHSSSLSNVLEDGSLPEPTI.[S]	4085.00328
8145	[G].NGLLWGWESHKRGDGSPAGLVSPPQPSLPQGSETVLMPS.[L]	4085.01854
8146	[G].PGSRTPPSAPSQSRVTSERAPSPASRMVQAPSQSALPPAQ.[D]	4085.05811
8147	[P].GGSPGDPSSSTSSLSPASPPSSPRTKDPPTGSPPASPGPQSPST.[K]	4085.91703
8148	[L].SQQKPNQWLNQFVPPQGSPGMGSSVMGTQVNVLGQSAFG.[M]	4087.97529
8149	[S].DSPGLLAAHSPHVMIGPNGSSLGAPSPGPPGPGVSPVQLAFSD.[F]	4088.0182
8150	[L].GQPGTLLGDDQIYNVIVTTHAFVMIFFMVMPIMIGGF.[G]	4089.03966
8151	[G].SLASSRGSLSSVSFTDIYGLPQYDKPDTEGGPLLRFDL.[V]	4089.04513
8152	[E].KATGLSLAAKIIKVKSAKDREDVKNEINIMNQLSHVN.[L]	4092.25977
8153	[N].TSTLGQPSTNTMGLFGVTQASQPGGLFGTATNTSTGTAFGTGA.[S]	4092.94549
8154	[A].RPPASSAGSNAGSAPEAPTSSPALPVTSSPAGASSAQQQLMQQ.[M]	4092.9527
8155	[P].QGPPGKPGPAGMKGEDGLPGSPGEKGEKGETGQPGPPGLDGPTG.[E]	4092.95673
8156	[S].APDPTVTPVGSSGDHLTPMAHPLDQPPPDHLSLGPAPSPSS.[G]	4092.96075
8157	[S].GAATTTAAAAASAPAPGPASSPEASPAPGFPFPPPWMGMPLPPP.[F]	4093.98267

8158	[G].PQGQPGLPGTPGHAVEGPKGDRGPQGQPGLPGHPGPMGPPGLP.[G]	4094.04134
8159	[P].IIPSEEMAKIACSLETKELWDKFHELGTEMIITKS.[G]	4094.05345
8160	[P].QGSVRPPPAEGYSEVGPSYGPGEGAPEQEKSRGGYASGFR.[D]	4094.92272
8161	[E].TQKDEAAQAKGTTVSMDTQDQGTEKGAKNKASEATERPA.[S]	4094.9531
8162	[E].PGERSPGQGSLRFCHKPPTELRGPDGIHVMHGSTGTLL.[A]	4095.03996
8163	[A].PWGSSCILPISWAYIKTLSSCSGEAVAASVPGIVSAHHT.[Q]	4097.02593
8164	[M].VPNSDPSRAVASPAGSRASSTRAARDGTEGARHPEARPSAP.[E]	4097.0368
8165	[W].TLAGCFLGSTNTSKLVIKPKHKVKKLGKKRTSAESRQ.[V]	4097.3492
8166	[E].GKGGPTSHPSPAPTPSSAPSPLGGSALCGGKPEAGESPPPAPGTP.[K]	4098.98255
8167	[E].GPAGAAGAQAGAREGVDRNSVPRRGDAMPEAEAGGVAGPSGGLR.[E]	4100.01871
8168	[G].QVGTGDLDGFQADTEEEDDEEGDCVLMDISDVGDIQAP.[C]	4100.68668
8169	[S].KMSYTLDSLGNPSAYRRVTETRSSFSRISGSPSSGFR.[S]	4101.02066
8170	[G].QHNQLGGNLRFEGPHGQPGVGIRFEGPLVQQGGGMRFE.[G]	4101.03726
8171	[A].SPDRIPSLPQETVDQEPKDQKRKSFEQATSASFPEK.[K]	4101.05234
8172	[V].EVKMEAGGERFHQQRQVLILFLLLGVTFAGWESRR.[Y]	4101.19672
8173	[P].RGEPSIDAPGHHTGEPPLGPIQAPKKKKVRFSVAVPSSE.[E]	4101.19922
8174	[E].HCIIDITPEGQVMLTPQKNTRLNLPKKKKKADRDD.[E]	4101.20596
8175	[L].DPGKAFVEVKMEAGGERFHQQRQVLILFLLLGVTFAG.[W]	4101.21064
8176	[G].SRRPDSSGPGAGAAPEPPASLPEPSREINKALPKPLLVKN.[S]	4101.22035
8177	[G].DRVHLALLGSPVPLGLLVKGGNQVLMGPQGVQAQWGLEGP.[L]	4101.243
8178	[F].PGSPGEKGEKGSTGIPGMPGSPGPKGSPGSVGYPGSPGLPGEKGD.[K]	4101.98221
8179	[T].SIPSAYGGQVISNGFKVCSSGGKGSVELYTHNKSVTWEA.[S]	4101.99747
8180	[V].KVEDSAFGKPAGGGGGQTPSTTAATAAAMGADEEGAKPKVSPSL.[L]	4102.00334
8181	[P].SAPLYLDLGSKTDKDTPGITVSSHPEQNLQETSDTQKT.[A]	4102.00987
8182	[P].SMTGPLLPGQSFGGPPVSQPNHVSSPPPQALPPGTQMTGPPG.[P]	4102.0161
8183	[L].AASVDRPGSNGVLYAVFSTDGRGGGGPRTGLCLFPLDEVH.[R]	4102.01994
8184	[L].GASSVVNNTAASKIDGSQSAVGAEVEKPGTADNVPKHQFPH.[L]	4102.02244
8185	[N].VDPDTYRLNVAGPPGGQSLCLSLDDLYQFPKHEVTVT.[L]	4102.02262
8186	[P].EVSMVRSSHNPTNNFSSVKVPIDVPHEGASLTNLLDPN.[S]	4102.02983
8187	[P].AVPGDAVSRGVPGGSGDQANPRGPSAAGESGGAAGAIPQILGAPHA.[P]	4102.0449
8188	[G].ETVKHETSYRSRHLEQTPVRDPSPEADVQVLGSPEK.[E]	4102.05882
8189	[H].NPQPQPVDGVFVYPLAEAEVVSGFEAEAAGRRVSFQLQ.[S]	4102.06687
8190	[P].GLAPPTQPGAPSMAGTVAPGGVSGPSPAQLGAPALGGQQSVSNKH.[L]	4102.07745
8191	[R].SPTSSPTPQRRAPAVPPARPGSRGPAPGPPPAGSALGGAPPVPS.[R]	4102.16932

8192	[T].PEASAVSLVPEGAARESLVALVSTSDRDSGANGQVRCALY.[G]	4103.04621
8193	[L].AKDPASGICTLLYDSAPSGRFGTMTYLSKAAATYVQEF.[L]	4103.97289
8194	[Y].FLTQSTASMLLMMAVPRATEAATKYFLTQSTASMLLM.[M]	4104.02341
8195	[N].MLGPQASQLPCPPVTQPPLHHTPPPASTAAGMPSLQHPAA.[P]	4104.02522
8196	[E].LNDSPVYKTVLERMQRFFCTLYENCFHILGKAGP.[S]	4104.02924
8197	[S].GGASLRSLGHSVDPDILSFSGLRDSARPAPNGTRCLTEH.[S]	4104.0428
8198	[C].VVHCMVSRPGSPGTRGRGGLQAPGSDRLSGLFRGSSLSHA.[P]	4104.08389
8199	[H].GPDWHLKMPKMKMPKISMPGFKGEGPEVDVNLPKADV.[D]	4104.09415
8200	[A].AVPQMFGVGGLLNAPMATTCASAVPAPLSSTTKGGASAAEKTA.[E]	4105.04026
8201	[W].FIVAAAIHDHRFPLNETACVAATFFVHFFYLSVFF.[W]	4105.06218
8202	[G].LGPSGASGPALASAGTGPGEPDEVDKFKAKFLTAWNNVKYG.[W]	4105.06653
8203	[P].APASASAPAPVPAPAPAPAPSPAPASSSDPAAAATAAPGQTPASAPAP.[A]	4106.04653
8204	[P].KSARCQASASPEVLASHSGHPTADLQTFQAKRHMHQH.[R]	4106.98965
8205	[P].VPETSLNLSGSLLFDSFSEDYLVKEQRPDGQEKEHL.[P]	4107.01931
8206	[E].KPRDQSPWNTSVAQVPAEEMPERPEDTLGPALHGPKAA.[H]	4107.03525
8207	[K].KPVSPESAPSAVSQPSSPHSPPSLGRSSEVSPVPTPSRGGAD.[G]	4107.03775
8208	[Q].LGGDLNSTPLHWATRQGHLSMVVQLMKYGADPSLIDGE.[G]	4107.04264
8209	[G].GQQLSISDAFIKANHNHMMALLQQLLHSESLSTSWR.[D]	4108.04913
8210	[A].QPGTPQDSPLPAHTPPSHSAKLLAEPSPARTMHDTLLPD.[G]	4108.05565
8211	[G].EKPGASVASVHSEAGPKGAEKPAAATGKGWPEAKGQGPSPQR.[P]	4108.09588
8212	[R].PQGGLPSSKQDGELSKTSFASSLYHSPSDSKEATVTDAAK.[D]	4108.99455
8213	[P].SPAVDPPTPGPGHPTHPAPPLLGSVTNRRGDDNAQAGAAGML.[T]	4110.021
8214	[P].AAPAGEGGPPAPPPNLTSNRRLQQTQAQVDEVVDIMRVN.[V]	4110.07851
8215	[H].HPSTSPASESSLSPAPSMDILSTENFKPATSQSPQVTSPP.[T]	4110.94482
8216	[G].AVSTPEQSATPAGAVSTPEQSATPAGAVSIPEEPDAPAGAVPTP.[E]	4110.99897
8217	[Q].QLLGDSTHPQGAGRSGQNLLGQASATSHIYQGPESSLPGPP.[S]	4111.02277
8218	[F].KGHPDLIMGFNTFLPPGYKIEVQTNDMVNVTTPGQVH.[Q]	4111.04158
8219	[S].AMTKVMTGTGVPQSIQAQGPSSPSSPPVEARTAAEQIPAHP.[L]	4112.05394
8220	[M].DGIAGQIGAADKPRDVSSVEVLMNYHQGLKTELEARMP.[E]	4112.05394
8221	[V].PQQPPTAPPSGLKKYEEPLQSMISQTQSLGGPPLEHEV.[P]	4112.06449
8222	[E].DMLRLQQEVSGLREEFRRQEARWAATHRELWAQ.[M]	4112.07437
8223	[D].YRLEDGGISSTSDDDDEEEEGKQVAWVKWMDSSTTF.[T]	4112.78257
8224	[D].SPPARSPNLPSMNNMPGMGINTQNPRISGPNPVVPMPTL.[S]	4113.01367
8225	[V].VGGPLPGDLQPGPSPESEDGSILSGVGPTHPSPRVGGFPGGGPP.[E]	4113.03121

8226	[R].GEVGVPGSRGEDGPEGPKGRTGPTGDPGPPGLMGEKVTGERGS.[C]	4114.98467
8227	[T].REASGQRDTGPKARLPGWDPAAGSASARGSTPAATNPPAPTS.[S]	4115.04015
8228	[D].KPSPAFSCFSKNATKSKEFMSPSYSQFSLPVGEKVMS.[T]	4115.99152
8229	[C].GQPADKASASGSGAPVGGSISSGSSASSVTVTRSYRSVGGSGGGSF.[G]	4118.96096
8230	[S].LAGAWTHKMGTAHVSVLGEDGSAVAATSTINTPFGAMVYSP.[R]	4118.99503
8231	[Q].PNQPSSLAMLDLLHVARDIACGCQYLEENHFIHRD.[I]	4119.9586
8232	[P].AGAAREEPLSTAPPAAAKEVGAAPALGAGQKPQGEATPGGGSGPLG.[R]	4120.10577
8233	[A].PPGGSLEPAPSSQPAKPTASSVTPKRPPPAPPAAMASPPSPLP.[V]	4120.15357
8234	[D].KEKGSSWRNWPGEAKARPLEQESGHPPGPARPQSLQQ.[G]	4121.08123
8235	[S].AAAAAAANPGEQFSVPDRVTGRAGLKELHAASPSRTWNFV.[E]	4121.10638
8236	[Q].QPDEQRRRSGAMVKMAAAGGGGGGGRYYGGGSEGGRAPKRL.[K]	4122.03292
8237	[G].PKGDDGIPGQPGLSGPPGPKGEPGHPGTDGAAGQRGPPGLKGEQ.[G]	4122.03876
8238	[N].KTSKEHVAKMMKDLESLQTAEQSLMDLQERLEKAQ.[E]	4122.05155
8239	[L].ERSASGDALPGAQPPLPPPSYDSLIFAAGGISGEAAPGAACSL.[A]	4123.0077
8240	[Q].GQVPSTTATTPGNSGAPQLQANQNVQHAGGQGAGPPQNQMQV.[S]	4123.95985
8241	[S].KGFPGSPGADLHGDLGFPGPAGDRGDPGEANTLPGPTGAPGQKG.[E]	4123.98566
8242	[C].SRQYTSSSSIKGSGGIGGGSSRMSSVLAGGSCRAPSAYGGLSV.[S]	4123.98838
8243	[S].RQYTSSSSIKGSGGIGGGSSRMSSVLAGGSCRAPSAYGGLSVS.[S]	4123.98838
8244	[D].QGLSGFPGSPGEKGEKGSTGIPGMPGSPGPKGSPGSVGYPGSPGL.[P]	4124.00295
8245	[L].PGPPGEGKVGEPGVAGPTGPPGVPGSPGLTGPPGPPGPPGPPGAPGAF.[D]	4124.0876
8246	[E].EEPVDPQLMRLDNMLLAEGVAGPEKGGGSAAAAAAAAASGGGVS.[P]	4124.98631
8247	[D].PAGPGQPLHVPGSPAAAGQEEAGGGGGPGQTPRPLEDTPGEAQK.[P]	4125.00204
8248	[G].PEPPSPAPEPAPSRAQAAEGPHLTPEASPDPVPEPPPPSVE.[A]	4126.00399
8249	[G].RNHGINAHAATTQYANGVVPSGQTANAVAHRAQEMLQNQ.[F]	4126.01323
8250	[L].KAVPTTIMNCRRMHTVTAHSNCIEVFPEVMQLPEI.[K]	4126.01631
8251	[Q].MGFPVITVDTNTGTISQNHFLLDPNSTVTRPSEFNYL.[W]	4126.02262
8252	[G].ALQGGTLWGLCPDPHPRGAPNILLACQSAPGQAGLGPGSHP.[T]	4126.0498
8253	[G].EGVAGPREEPLPPAPPLANGSQPPQGLPPNPADPTRTFAW.[L]	4126.0781
8254	[Q].LGQDPFFDMHMMVSRPEQWVKPMAVAGANQYTFHL.[E]	4126.90708
8255	[G].NREYSARVPVTTNTTDSVSDEEKVSGGKDGNGSTSSIQGP.[P]	4126.93955
8256	[A].TPAGAVSTPEEPATPAVSTPEEPATPEEPATPAGAVSTPEQSA.[T]	4126.94626
8257	[G].PGPSPGPAPPNYSRPHGMGGPNMPPPGPSGVPPGMPGQPPGGPP.[K]	4126.9473
8258	[M].QGPPPQGSMLGPPQELRGPPGSQGQQGPPQGSLGPPPQGGMQ.[G]	4126.98217
8259	[D].NREALYGVFDGDRNVEVPSLLQCTMSDILAEELQKT.[K]	4127.00598

8260	[G].ILDGCAHGRCVRVPEGFTCDCFDGYRLDMTRMSC.[V]	4127.75298
8261	[P].FPQAFATSPGHPPHFSELLDPRIYAFESGHCQAMER.[L]	4127.92795
8262	[G].HVTFGSTSATTGGLMSSRGSTPGRVTFQSPNSSSYLGSTGY.[V]	4127.93632
8263	[G].TAGSSGGSAPLGSPSSKPPTAPTNPTPPTERGPEPTLDLSGEQ.[F]	4128.00036
8264	[R].GQQSREQWRPGGNLHGSLTEAAPPHADGWLPLLSSGPHS.[S]	4128.0183
8265	[S].PTRSVNSKYNSTDTNVIKGTAPMGTLMGSPVHLEPSNQV.[G]	4128.04885
8266	[L].KNCFALLTTPETKTFDSQMKEKIFPQGVQRGSLQDS.[V]	4130.06852
8267	[T].PAAEGVGAAANAAATSSTGTGGVAASGMAASGVVPGGGFVASAAAEVQT.[G]	4131.98875
8268	[K].CKLEVPGWNGVTHYSMNECEDVEMCCDQEVGESW.[E]	4134.63772
8269	[S].ATSTAATSATTIASTGQTFQITGNPVTMAGKVITKLPLPANS.[K]	4135.15911
8270	[G].AARGEAIETDDTMGGIPAVNGRGERLLLHIGIIDILQSY.[R]	4135.16045
8271	[I].RSPSASSVGSRGSSGSSSKPAGADSTHKVPVVMLEPIRIKQ.[E]	4135.16766
8272	[G].NTSTLGQPSTNTMGLFGVTQASQPGGLFGTATNTSTGTAFGTG.[A]	4135.95131
8273	[P].PAAWSAPFSAPKDEWAAAYGPGPTAPTASPAPLAFGPPPDFG.[A]	4135.98649
8274	[D].ADAVAPGPQRRACSLDSAPVAPPPRQPCSSLGPAASEARPA.[V]	4136.05125
8275	[L].AGVPGMPGTKGGPGDKGEPGRQGFPGVSGPPGKEGDHGERGPVG.[Q]	4136.99551
8276	[V].GPAGAKGMPGHNGEAGPRGVPGIPGTRGPIGPPGIPGFPGAKGDAG.[T]	4138.11517
8277	[S].PGQVGQGGSALASPAPRPPASRASKMWDAVLYRMSVAASRG.[G]	4138.11854
8278	[F].DRSNLYGKEGREGMVIPFCCRVCVMAGTATASLSQEG.[K]	4138.88715
8279	[G].PKGEMGPVGPAGNPGAKGERGSSGLDGKPGYPGEPGLNGPKGNP.[G]	4139.03631
8280	[A].QLNITEQNWSPGQPSFLQSRDLRGMPNHIHMGAGPPP.[Q]	4139.99268
8281	[A].NGIAGSMATLLHDAVMNPAEVVKQRMQMYNSPHRSALS.[C]	4141.01982
8282	[Y].APSSAASYFEAPGGAQVTVAGSSPPAVPSHSMVGITMDVGGSPI.[V]	4141.98452
8283	[K].PGPNPEEAPGAGKGSRRSWAEAADHIFFQVYVVGVACSQ.[F]	4141.99372
8284	[A].APFSQPQWVPAPQDSGPPAALAPQPPGSFPSGPCTEALLSS.[P]	4141.99641
8285	[A].GPSASASAGPSASASAGPPASARPGTSAAARAATSASARADMSATAR.[P]	4142.00278
8286	[A].NNNVGGEASAWPQQPQPRQPPPPAPQPLNGRGADEEVEL.[E]	4142.00746
8287	[S].GSPASVMTSIRAPSTTGSLGINSVTGTNTMNNVNITAVGSFN.[P]	4142.01286
8288	[G].PFYSPRDPEPPEPPTYRAQVVGGPGPHEEQRPYPQGL.[P]	4142.0155
8289	[S].GSSVTSPSGTRFNFSQLASPTTVTQMSLSNPTMLRTHSL.[S]	4142.02812
8290	[V].TPPPGSAPGPGPLSGSQGPGQQCLGQAGLPGSVPASTHSLTHSL.[T]	4142.03598
8291	[-].MQVGQGGSPPMLEGAGGLGISVWTPGSPHQTAVLPDGLRWSE.[G]	4142.04
8292	[M].QVGQGGSPPMLEGAGGLGISVWTPGSPHQTAVLPDGLRWSE.[G]	4142.04
8293	[G].PPSSPGSRKLSAAGSSDGVMPVAPTSAVSSSGSPASVMTSIRAP.[S]	4142.04925

8294	[D].NPSQSRTVEIFVNYYGNLFPGGALGSVKPQDPDVLDTF.[H]	4142.05055
8295	[R].GCLSPRAGPPAARERGGGGLEEEPVDGLAGSAAGPGAESRAGGAA.[V]	4143.00205
8296	[V].SFRSGGSRSFSTASAITPSVSRTSFTSVSRSGGGGGGGFGRVS.[L]	4143.03507
8297	[P].GDAVSRGVPGGSGDQANPRGPSAAGESGGAAGAIPQILGAPHAPGPG.[G]	4143.03507
8298	[T].VEGGSSVDQLPYLDAKPPTPGASFSQVEISVDPEPNSTQT.[L]	4143.98807
8299	[G].RPLEGEHPQDTHNPWLPPWPGQNGLWEASLGGPHTVH.[A]	4143.9961
8300	[T].TAAEPQPTAPPTVCVTGPPTARPSEGPTTGPTGPPAAGPTGPPT.[A]	4144.02916
8301	[P].SPGVEPVASMTSVASHPALGASSSSLPPLGPAAMNMVGSLGVPP.[S]	4144.03993
8302	[G].PSPGVEPVASMTSVASHPALGASSSSLPPLGPAAMNMVGSLGVP.[P]	4144.03993
8303	[V].EGGATLFKLDYFGEEAYLTQSSQLYLETCIPALGDVF.[C]	4145.99401
8304	[D].EAAAAQASEVALATTSGANWVATTSFVEALAALQESGQLSSH.[I]	4146.02619
8305	[R].INDAVANEDGPQVLTGRFMYGPLDMVTLTGEKVDVHIM.[M]	4146.03445
8306	[P].PGSTASLSTASLTPSSPRVPNVSAQGPTVQAPMPTPRTVDDA.[S]	4146.07717
8307	[Y].FFTYHILMRGGDGTSMWADLCKNGQVRASAIAQDADQ.[N]	4146.92188
8308	[D].PTYTSSLVQEAAGRETGGGAGQAGARGWGPAPRHRVKGVCP.[P]	4147.0751
8309	[G].RDTSGPRKEWHGPPSQGPGYHDTRRMGDGRTGAGMITQ.[H]	4149.95908
8310	[G].LHTDMDLPGRGLANPAPSCYLLGSEPSSGLGPPPEAHLPE.[G]	4150.00084
8311	[L].NAAEQRGAREAAGSASRSGPGGSGSSGRGGAGVPGPGSGGPGGSAGRM.[S]	4151.94806
8312	[Q].SIVTTDSQKHRMNGRAFEDFEERFAAATPNRNLPMD.[F]	4151.97742
8313	[G].APSPGEQVLASAFPLAKPPVSSELGDNSCSSDMTDSSTASGT.[S]	4152.88599
8314	[V].KQDFKMESPSNSALMLPSTPQAGANPPSPHSSSSRKQPM.[S]	4152.98996
8315	[T].APSSPISVTMEPPEPHLIADGPQHHHHLHHSQQPPPAAA.[P]	4153.02094
8316	[A].KDVAPCQEIEVTLGKDTVSPPETEMALGRNVSLPPETE.[V]	4153.03153
8317	[S].PSSPVPTPSPSTPAPPTSSPSSPAPPNPSPSNPTPASFAPVPAP.[E]	4153.04004
8318	[Q].SPHYFRSGRGEGPGEKKKEDSRGDDGKGIGPPSQNSSVGP.[G]	4154.00343
8319	[V].KVEPADSVESSPPSITHSPQNELKGTNHSNEKKNTPAAQ.[K]	4154.03848
8320	[G].AAANAAATSSTGTGGVAASGMAASGVVPGGGFVASAAAEVQTGRNNF.[V]	4154.97959
8321	[V].EEDRLGRAHSGHSGSPRGSLSRHPSSQLAGSGVEGGEGTQK.[P]	4155.00589
8322	[G].PKGEMGPVGPAGNPGAKGERGSSGLDGKPGYPGEPGLNGPKGNP.[G]	4155.03123
8323	[A].VGGFGGLGGFGGGISGAVGGFGGLGGFGGAVGGGDAGILPADEKTTMQ.[E]	4157.00328
8324	[S].PRKEFSACAIGCKVYITGGRGSENGVSKDVWVYDTLH.[E]	4157.03314
8325	[V].RASAQGKPGNRNSNSYGVPEPAHAYAQPQTMAPPPAAGAPGA.[T]	4157.99585
8326	[A].AAAAGATPPSLAGHPLYPYGFMLPNDPLPHICNWVSANGP.[C]	4158.00005
8327	[L].SSDTIPVAPETSRPPTTSSAVSSAAPGPATETPVFGIVTSDSS.[V]	4159.0201

8328	[H].KGTSSGATMAPASKATPSSVPSSETAPSAASHITRTAASSTSPQ.[Q]	4159.02078
8329	[S].GLLSCTLPNGFGGPPGPEGERSLAPPDASILISNVCSIGDH.[V]	4159.0223
8330	[M].AVNNCGLQAGLPPYSPVFKSWIHCWKYLSVQDAPGAQ.[E]	4159.03169
8331	[V].SSAVTTTSAHQGVTTPPKFPLENMINTATSQPATSIGDITS.[S]	4159.04996
8332	[F].NEVDFIPGHLAPRPPPPSSPKPASMGNAIQEEGRELGPPG.[P]	4159.06655
8333	[G].PGSPAQDTYQVPPSAGVGQGVYQVPPSMDARHWEGTKPPA.[K]	4159.99305
8334	[R].LCKTNLSSNTAFRGFGGPQALFIAENWMSEVAVTCGLP.[A]	4160.00382
8335	[H].RSQPGGAAPPGHQMFAIQPGAAEGGQFLGGPPPGVCPPELQP.[D]	4160.02291
8336	[T].QPPEAQSGLPEPMNENCTIALPECGKARRPGAWPWEA.[Q]	4160.93753
8337	[G].KDLDACGQLSTGPHRGHLHPGAGGRERCASPLGAGAAGSQGP.[A]	4160.9962
8338	[L].TPAPGVPGGDTGESQVLHVHVEDRGIRMEQQQEPWVPK.[H]	4161.05705
8339	[L].PQDPAIVQSSLGSGSASSFQPHVPYSPFRGMPPYSQLAAS.[S]	4161.99747
8340	[R].FSAKMDHLVCFLPGTLALGAHHGLPAEHMELAQALMDT.[C]	4162.00171
8341	[G].PGPRCRGGGSGRASRPESRRMERSGRGPAGSGGGGGPGPYPL.[T]	4164.02956
8342	[I].PGNPGLPGPKGEMGPVGPAGNPGAKGERGSSGLDGKPGYPGEPGL.[N]	4164.05671
8343	[T].FPAGSTNIDVKQRSPPGVQNDGNYLALKTTDGEYLLNGN.[N]	4164.06324
8344	[V].QREAAEVSEHAVMLAKNSGEELTLDANIREVGLEGALLT.[L]	4164.12412
8345	[D].DPPALSEAGVMLPEQPAHDDMDEDDNVSMGGPDSPDSVDP.[V]	4164.71145
8346	[S].GPYPSIPAAAADPSMVSAYMYPAGAAGAQAAPQGPAGPTTSPAY.[S]	4164.93176
8347	[G].VEEKMFSLLKRAKVQLIKIDQQQQQKVASLMPPSPGG.[Q]	4166.28282
8348	[A].AAAAYGRSPMVGFDPHPPMRATGLPSSLASIPGGKPAYSFH.[V]	4167.06913
8349	[E].AAEEAGVRGGAEEEEEEEEEEEEEEEEEEQQPATTTATS.[T]	4167.67637
8350	[S].PAPSASPTTLLHSQGLGMDLWEEWFGPGSSCLNSVPNDP.[I]	4167.90627
8351	[I].QRGSLIMDMVSDKGNLVYSDNRSFQGKDSIFGDNMNE.[L]	4167.91685
8352	[G].IPSGPSRGGSSSSTMFMQGPPGPPGPPGPPGSLSSSGLEIQQY.[I]	4167.97502
8353	[T].PGAAGGATAASAAASVLGGSAAPATAGDTTKSENVAPADRSATPATDG.[S]	4168.00249
8354	[L].VLMGGELADGTLTSDIWAFNPMGGGRWELLAPPASSPSGPP.[G]	4168.01543
8355	[P].RTLPPAGMGSQSRPQSTLQGFGYSKERGHTGSAGEAFLST.[I]	4168.02648
8356	[T].KMLNVCFSTLPGYTRSTAHVSRESSVVMMSLQATFSL.[A]	4168.0334
8357	[P].QVGASHLMGYPTAYPAAAPAYNPSLYPTNSPSYAPATLLM.[K]	4169.99871
8358	[E].IQVSEQEPSPTGPPGPPTSPETAGTTRPSSSTTSEVPRPPE.[P]	4170.01093
8359	[L].TAVPNGFHEDGPLGPRGDEEDEDEEEEEEEEDTDKLL.[N]	4170.75416
8360	[A].MMAIMIMKVHSIYRGAGGSFQKAQTEWSTGTWRNPPS.[R]	4171.01328
8361	[F].GIVTSDSSVFTQPPAASSSSAFSQLTSHTATAPSATPMFGQV.[A]	4171.97646

8362	[T].GISQDTTSLPTGATAPSETATDLTPGFGSAPVSMLTTVTMLD.[P]	4171.97849
8363	[P].VYASRSEPPASMGPRYNTYVAPGRSVSGHHPKPCSRAE.[Y]	4171.99374
8364	[R].PGPSPGPGPSPGAMLGPSPGPSPGSAHSIMGPSPGPPSAGHPIPTQ.[G]	4171.99642
8365	[G].NLHAASSPSGALRAPSPASFVPTPPPSSHGISIGPGASFASPHG.[T]	4173.09006
8366	[I].KMSSFNLTRIVTLTPFYTIENKSSLELEVGEIASDGAL.[P]	4174.1628
8367	[D].SASSSAWHRLRPTDGSSGQNSKAGTGMSKSASFAFEFPKD.[R]	4174.96354
8368	[A].NREKMTQIMFETFNVPAMYVAIQATTYNSIMKCDI.[D]	4174.97786
8369	[E].FGPGPGTGQLVFGGHHRTPVYTTEEQRGGSPATTFAMAEL.[Q]	4175.00395
8370	[S].GEGHLEISASGVEDLSRLPSRGEDHLETSASGVGDLSGLPSG.[R]	4175.01233
8371	[P].GVPGSPGFPGVPGSPGIMGFQGFTGSRGDKGAPGTAGLFGEVGPT.[G]	4175.0291
8372	[W].RSLCTSTVAQASSRTQGEDVRVEGAFPVTMLPGDGVGPEL.[M]	4175.04958
8373	[L].EAELELEMKTLSPQAPSPLGSPFLWPGVEGPDSPSSPKPG.[A]	4175.05292
8374	[A].QPFVAAANIDDKRQVVSASYNSPIGLYSTSNIQDALHGQ.[L]	4175.07922
8375	[C].GQPADKASASGSGAPVGGSISSGSSASSVTVTRSYRSVGGSGGGSFG.[D]	4175.98242
8376	[S].QIGGPSSTEAYVRYHDAGVAEVSRFGSRFGGDKGLSSHHG.[L]	4176.00304
8377	[S].GNLSEPVPAAPSSDLDVNTDIYSKVLVTIVYLALFVVGTV.[G]	4176.23662
8378	[A].PGASGHGGTVGGVKAAAEPVEAGEIPGPIASCQELKGSADSFVD.[P]	4177.01424
8379	[C].CHLTAGQSQVKERNEDYREITQKLSFPMGRNIFSH.[D]	4177.04544
8380	[G].GLHQYKWHHPPGHPALEARSTGCVLAGGVAGAGAMLLCSL.[T]	4177.07932
8381	[H].PALEARSTGCVLAGGVAGAGAMLLCSLTLTLLWMVASGLEC.[D]	4177.09865
8382	[G].PVMGGKPPAPHPPPFPPRAFQSHDPHSGVFPRYRPHQG.[M]	4177.09907
8383	[G].FIQAMSALRGMKLMFKGEDGKAVACNIKVSFDSTKHL.[S]	4177.10651
8384	[G].QASGDASAFSQKLLNVPILGSLASCSLSFATVYWMLRTS.[L]	4177.10966
8385	[G].PSGKWSLGGRKGLGGSEGEPASGSPKGSTPKSQAPLDLSLSPD.[I]	4177.116
8386	[I].NPLKSPTMRQVQSPMLGSPSGNLKSPQTPSQLAGMLAGPAA.[A]	4177.12024
8387	[E].CAEVRGRDPGVGGTGLEQGPSAGAASAGPQVSLYQGAPPAAEQG.[V]	4177.99557
8388	[L].VAQGTPGMAPPPAPATASASSGTTNTATTAGPAPGGPAQPPPPQASA.[S]	4178.00949
8389	[P].QGPAGPPGPPGPMGPPGLPGPMGIPGSPGHMGPPGPTGPKGTSGHPG.[E]	4178.01572
8390	[Y].TTFTTVGDWLDAIKMGRYKESFVSAGFASFDLVAQMTA.[E]	4178.02493
8391	[P].TPNPTVFFESTKAGPALGQGAGPGAREADTGVSQGPASAPRPE.[V]	4178.05374
8392	[G].SVLAQDPDLGQNGTVSYSILPSHIGDVSIYTYVSVNPTNG.[A]	4178.05642
8393	[N].PQVQQLMSGMISGGHNPLGTPGTSPSQNDLASLIQAGQQFA.[Q]	4179.02337
8394	[I].PMLTELCEAMKTNTHVRSFSLVATRSGDPIANAVADML.[R]	4179.03413
8395	[E].PDAAATRAAPNPASLPNTLGSGYSPRLCPLSFGEGVELDPL.[P]	4179.08153

8396	[R].KSSGGPWTYWGQSLGAVLGPDPPVAFPQVYTSGKGSSAAGLT.[A]	4179.08218
8397	[N].LPMFTGNAYFVASRAFVQHVLENPKSQRLIEWAKDT.[Y]	4180.14368
8398	[S].PFRPASGTPEAGRLEEPPAAGPAEAERASSPCGASPPTPSAGP.[Q]	4181.99451
8399	[D].PGPGEVPAAATALPQFVERLCRQAPIPAMETSAATALTEF.[V]	4182.09983
8400	[G].KTTSSPSVTFPSMGSKTLVAITSVVASDIASNLGQLSQTSSP.[A]	4182.14861
8401	[L].PGRDGMTGAPGLTGERGEKGEPGERGPPGFPAYLDEELQGT.[L]	4182.97852
8402	[K].ATPSSVPSSETAPSAASHITRTAASSTSPQQLPVRVFLFSL.[S]	4184.16223
8403	[P].GIMGFQGFTGSRGDKGAPGTAGLFGEVGPTGDFGDIGDTIDLP.[G]	4184.98696
8404	[L].RDTGKEGNAEQRKDAPPREAEAPGGDQGGGGGGLSPGPSAKPE.[H]	4184.99399
8405	[T].GAEEAAVAPGAFERAHPSPRANADPGPTGGTAPDSPRAFLAAM.[E]	4186.01591
8406	[-].MMGLRAGGALGRAGAGRGAPEGPGPSGGAQGGSIHSGCITAVHNV.[P]	4186.01997
8407	[M].GHLLASVAGSGGGGGGGGPGTATGLDAGGLGPAGNAASTAGPFPFHLSQ.[H]	4186.03367
8408	[P].QGPPGTPGMQGPPGPRGMQGPPHPHGIQGGPGSQGIQGPVSQGP.[L]	4187.00464
8409	[L].AQLDSHPHPTPVVNWTSYASGVKAHSMGNQEFTGIIFF.[D]	4187.00797
8410	[T].TAAAAASAPAPGPASSPEASPAPGFPFPPPWMGMPLPPPFAFP.[P]	4187.00816
8411	[P].LPRMDQSSSRASPGLAQKGCGESAWARSTTTRESPVHTA.[M]	4187.01051
8412	[E].IPQSLGTYLQGMASTLSPSQEPPPPPGSQVPPASPSSQEPE.[S]	4187.01251
8413	[P].LPLSAPHTHHGCEHYPGIRGHHRQAPYPSAYVHRNH.[S]	4187.02909
8414	[S].PAVWGQPGEGQPVCPLQAQVQGGGSAATSSPTPSHVHVRGPT.[V]	4187.04755
8415	[T].GPVPPGRRHDLYDIYDLPDRGFEDHEPGPKRRRGGC.[C]	4187.04888
8416	[E].ATPRTNFHSSVAFMFRNPPAVAMASPASAPAPAPSPAPAPAP.[A]	4187.05896
8417	[K].GEKGSTGIPGMPGSPGPKGSPGSVGYPGSPGLPGEKGDKGLPGLDG.[I]	4187.07136
8418	[S].PAGNQVQAGKQSHIPYSQQRPSGPGPVTQGPQQPQPPSQQ.[P]	4187.07654
8419	[Y].NIGSLSSGTGAGAITMAAAQAVQATAQMKEGRRTSSLKASYE.[A]	4187.08194
8420	[M].APSSAPAPNAGVLSSLASDGGPGSASTFTNPLLPLMSEQFKAK.[F]	4187.09651
8421	[F].RSPLGSVSQCFSANFPPTRCAHGVTARGRVSENGAGGQASG.[E]	4187.99586
8422	[A].QAAEGPHLTPEASPDPVPEPPPPSVEAPDKPTGSPDQPPSPA.[Q]	4188.00439
8423	[P].AANTASSAFLQLPSEADELATFSAKPEASDEKPKQADPPSA.[Q]	4188.02552
8424	[Q].PSPALGGPETGPGHRATSEEGFPQLSPQVQKPPAGPGMKALP.[W]	4189.1135
8425	[G].PLQGKEDRHPPTLHPADKGDEKNAKELEGLQGKQDGQK.[E]	4189.13847
8426	[G].PQGLPGMKGEPGLPGPPGEGKVGEPGVAGPTGPPGVPGSPGLTGPPG.[P]	4189.13865
8427	[S].SAKAVDHVLGPLNSNTNLGQNTPTSSSLQRTAVTVGTKTSH.[L]	4189.1596
8428	[G].PPGIDGKDGTPGMPGVKGSAGQAGRPGNPGHQGLAGVPGMPGTKGG.[P]	4190.06182
8429	[A].RQPGAPSLLDGACGPGIQAAKLKSITMQGSGSHLDFGLSVAP.[N]	4190.14851

8430	[P].GRPEPRDRGSTLPRRQPPRDYPGATAGRFGSRDALDRG.[A]	4191.15277
8431	[H].KSPHQFPSSLVQILRHFEASCRQWPPARRPPAEPSP.[A]	4191.15698
8432	[Q].PADGGAGPAGAGGAAAVATGPQALFSGGADLLGLQAPPTVLTHQALV.[P]	4191.18329
8433	[A].INTFFAKNGYRLMDSSMYSQPIQTQAQYASPVFMQP.[V]	4191.96128
8434	[P].VQEAGGQADALREAPGPGGEVGGQVETGDAGGRAEELLGETLGS.[K]	4192.00249
8435	[G].PAGNAASTAGPFPFHLSQHMLASQGIPMPTFGGLFPYPYT.[Y]	4192.00955
8436	[E].NPPEPVSTGVSHYGAEHTAVAPTSSTKSTSVNFGSLSMTPF.[G]	4192.97679
8437	[Q].EGHAVTLQYPRSPDGYLQIGSFYKGVAQGEVDPAFGPLE.[A]	4193.06145
8438	[E].DVKMTGYDLRADAIGIQHAKASRDIASDYLYKTAYEK.[Q]	4193.09718
8439	[T].TAAFVTESLNRRLEDSGSAHLEGIDLNNRTSNTQNHLL.[S]	4194.09225
8440	[D].LIMGFNTFLPPGYKIEVQTNDMVNVTTPGQVHQIPTHG.[I]	4194.11508
8441	[E].KGGPEQQKEVEEEEDEEDEDEDEDERQLLGEFEKE.[L]	4194.77529
8442	[G].PPAPGPRPQPEPEPAAGEPGRAATAPTAGGEPLSPPPPQEPAPG.[A]	4195.08431
8443	[Q].VSQLDNGLRVASEQSSQPTCTVGVWIDAGSRYETEKNN.[G]	4195.9949
8444	[D].GKAAAPAAGHHHHGNPGGGGGGGGGYMLSKSLYPDRVTQNPFI.[P]	4196.03798
8445	[D].PASSEKQGSMSVGMTKTMSAGQVEPTSLKNMDPVSAGEVGSV.[S]	4196.98182
8446	[N].PAEWALYAKFDQYRYTRNLVDQGNGKFNLMILCW.[G]	4197.04734
8447	[G].GAGGGGFGGLGIGFGGGSGGGSLGILSGIDGGLISGSEKETMKNLND.[R]	4198.0357
8448	[V].PTPSMSPPRPGAPAALTDTGASDLGSPGPGSRRGGSPQTAVSPAS.[E]	4198.05817
8449	[P].QGQLGGGCPLRFEGPPGPVGTPLRFEGPIGQAGGGGFRFEGS.[P]	4198.06755
8450	[G].RGAGMPYPTPAMQGATSSVLAETLTQVSPQMASHAGLNTAQA.[G]	4199.03182
8451	[P].PVCKPDVISHLERGEEPWQAARDGPAGPGPEPGWEPLPA.[A]	4199.04034
8452	[L].VTPEAGAYDSIRQAHSLSAEAERRANTSALTVPSPVSSSAD.[T]	4199.05994
8453	[T].SSLTQPIEMPTLSSSPTEERPTVGPGQQDNPLLKTFSNV.[F]	4199.08126
8454	[A].GIWGSGLGGAEGGAPASRAGLPSGPGPGCRVTRSPAEPRGARASG.[L]	4199.10238
8455	[T].FSGEFVDIHPGGASKLMLAAGGPLEPFWAHYAVHNQPHV.[Q]	4200.05486
8456	[Q].PEAAAAATTPVTPAGHGHLEANSNEKQPPQQDARPAEQSLD.[M]	4202.01333
8457	[Q].VATSTAPSLFGQQTGSNVSTAAAAPQVSSSGFGSPAFGASTPGVF.[G]	4202.03127
8458	[S].QQNVLSGHSQQTSLPSQTQSALTAPLYNTMVISQPTAGSM.[V]	4202.04925
8459	[L].GPAASEARPAVCERWVLVMGAGSRQVCGTRSVPGSFLGSAA.[T]	4202.08044
8460	[V].GLPGSPGAKGEQGPAGHPGEAGLPGPSGNMGPQGPKGIPGNPGLPGP.[K]	4202.0836
8461	[G].GPLSVYPGAGAGGGGGGSSVASLTPTAAHSGAHLFGFPPTPPKEVS.[P]	4202.09415
8462	[S].LGGSGAPPPPSMPPPPLGSPFPVISSSMGSPGLPPPAPPGFSGPV.[S]	4202.0977
8463	[L].CPPRQLEGLPRTPMRPAQVEKASQEALPMDGKRSLEP.[S]	4202.12673

8464	[A].VTDRPVMDVAFVQFLASVSGKVSCLGKTEVVRSAHMGAH.[V]	4202.13075
8465	[Y].NIGSLSSGTGAGAITMAAAQAVQATAQMKEGRRTSSLKASYE.[A]	4203.07686
8466	[L].APGGLAAVGRSAGGEPGPEAGRAADSGERPLAASPPGAVKAEEHQ.[R]	4203.09258
8467	[L].PGRPGPPGPPGPPGENGFPGQMGLRGLPGMKGPPGALGVMQEK.[E]	4203.10487
8468	[G].PPEHGEARPPPSAVPEGPAALASEATRPAPSEDPPSQPAELP.[S]	4204.05815
8469	[-].MIDNPGDNILVNEPVYSGTIHAIRSDLQAWSPLTSESPTP.[L]	4204.08331
8470	[M].IDNPGDNILVNEPVYSGTIHAIRSDLQAWSPLTSESPTP.[L]	4204.08331
8471	[A].KFYRSVDSTLPRSPVELDEDFDVIFDPYAPKLTSSVA.[E]	4204.11248
8472	[D].LETGKITDFIKFDTGNLCMVTGGANLGRIGVITNRERH.[P]	4204.17539
8473	[V].YIEHRLMMEQRSRDPGAARSPQNQYPPELMRRFE.[L]	4205.03383
8474	[I].GLDGNIGCLVNGAGLAMATMDIIKLHGGTPANFLDVGGGATVH.[Q]	4205.09403
8475	[P].QGLPGMKGEPGLPGPPGEGKVGEPGVAGPTGPPGVPGSPGLTGPPGP.[P]	4205.13357
8476	[L].PGPLASTSAPPGPPAAASPCLGPAAAAGSGLRRGAEGILAPQPPPP.[Q]	4205.19242
8477	[L].APVVALVLPGYSFSPVTPALPPAFFPSQPNFASEIPPASQP.[E]	4205.19978
8478	[G].VNPEIDIRSFVFTENMICAGNDKGVDSCEGDSGGAFAVQ.[D]	4205.88488
8479	[D].ETRQILDTEDELQELRSDAVPSEVRDWLASTFTQQT.[R]	4207.04256
8480	[C].PGLGRQGPEPGGCPAALTSLSFSSLQPGPTPSGTNVGSSGRSPS.[K]	4207.04727
8481	[L].SGYLLVCFSAGYFIHDTVDIVISHQSRASWEYLVHH.[V]	4207.04944
8482	[P].PAAALGATCAAAFPSAASVTSAGATSASSVHLPVSAPHGAGLMAAAS.[A]	4207.05467
8483	[K].AEKPRFMFNIADGGFTELHTLWQNEERAAISSGKLNE.[I]	4207.06655
8484	[S].QPFLFGTPPTSGTSTPTFGQNTPAPGVGAAGGSLSFGASSTPAQG.[F]	4208.02071
8485	[Q].GVPGPPGFGGEPGPQGEPGPPGDRGLKGENGVGQPGLPGAPGQGGAP.[G]	4208.05441
8486	[L].STSAPGSGSTTTSPVTTTVPSVQPIVKLVSTATTTPPSTAPSGPG.[S]	4210.16128
8487	[K].QAGCQLLARGHGDGIRFILDVLLPEAIICAISAVDAVDY.[K]	4210.17874
8488	[H].SASPSPVSMGRSLTPLSLKRPPPYDAVHSGSLSRSSPAVPH.[S]	4210.18258
8489	[S].SGPPGPPGSIGPKGPEGLQGQKGERGPPGESVVGAPGAPGTPGERG.[E]	4211.12282
8490	[T].APSPAHPAKQRAQTTVPASAPGPPQPQPQAPSHPAGQSALPQG.[L]	4211.14931
8491	[G].PITDAAKSPSAETSAKSQALSTPPPSPKQSILFGMLSTPAAN.[P]	4211.15403
8492	[T].AAPAVCNGVLQGILTFADGCVLRADVGIYTRIFNYIPW.[I]	4211.15689
8493	[R].DANPALYVTAGSNVTLSCTAASRPPADITWSLADPAEAAVPA.[G]	4212.05538
8494	[E].EGPYKVDITYDGHPVPGSPFTVEGVLPPDPSKVCAYGPGL.[K]	4212.06342
8495	[S].APASPSSASKEVGIGFAQGPGASASTAATPGPAGLPRGYMAPTSPA.[A]	4212.06661
8496	[P].HSTPRPVSDGGKMVNAAVNTYGSAPSGSRSRTPTSPLEELT.[S]	4212.07382
8497	[A].EQRGAREAAGSASRSGPGGSGSSGRGGAGVPGPGSGGPGGSAGRMSLT.[P]	4212.98959

8498	[V].PGKDGQAGHPGQPGPKGDPGVSGIPGAPGLPGPKGSTGGMGLPGMPG.[P]	4213.05533
8499	[G].ASPSPPAPSPRNGAAGQREGGGAGGPAGPGLVGVVAEEAARQPSSH.[S]	4213.08817
8500	[Q].RADMAIGSLTINEERSEIVDFSVPFVETGISVMVARSNG.[T]	4213.09038
8501	[L].FFPPQLNGTANDTAGPELPDPLGGLLDEAMLDEISLMDLA.[I]	4214.01957
8502	[K].SRGKLIGAQGAQGDSQMSHSLQMSKLQSGLEYKKGFEDT.[K]	4214.06048
8503	[R].PWRAEPDVRDGFSATFEKIVESELLRGTQYSSLDSLD.[V]	4214.06766
8504	[P].GGPEMTKTRSASTSSPLQHPRPRMTPQNRGSQEPRPEGA.[A]	4214.06903
8505	[E].GPSTAPPHFGQTGPVFPAVPPALSSAPGAPAAAAAASMSAPAPSHP.[L]	4214.07639
8506	[T].VAAAMSMRSPVNVSSAVNITSPMNIGHPVTITSPLSMTSPL.[T]	4214.0964
8507	[P].RNSVQHQFQDTFPGPYAVLTKDTMPQTYKRKRSWS.[R]	4214.09885
8508	[E].VQEVDVGARDRVFQEVSSFQGPPDATVVVNLQSPSAEEK.[D]	4214.10002
8509	[S].KMAQIETGPTSSLTAGLRDTSTHQEERLSRDPSKNTAVT.[N]	4214.1106
8510	[S].PGEAAGGQAEARREFLEPVQEAGGQADALREAPGPGGEVGGQV.[E]	4215.04496
8511	[R].LLEAASVSSKGLPSPYNMSSAPGSRSGSRSGSRSGSRSGSRSG.[S]	4215.05555
8512	[T].SSAKAGGPAAEPPASSSRDQRKALPGEGGRTQMTKSDSLPSF.[R]	4215.08472
8513	[I].QLESCQLNLGPDMEIVELIQGQEAKSSCVSLLSPSPSGD.[I]	4216.00941
8514	[L].PAGSHSAAPPPPFHTAPPSASQASSSPDPKLCLLPPTSDGRQ.[E]	4216.05163
8515	[G].KDIEKACQSIYPLHDVFVRKVKMLKKPKFELGKLM.[E]	4217.35275
8516	[A].PPEQFKEEVHMEGVLQAACGRARGQPDAGARAEQDLGGR.[P]	4218.03158
8517	[Y].RLHDESNQRMLALSSSRDTNILSEQGGFREGLEGSAKE.[A]	4218.05923
8518	[L].KPEHSELPQLDSQPTPGPPDRMQAEAEAEAPAPRKPSRG.[F]	4218.06326
8519	[E].PVASMTSVASHPALGASSSSLPPLGPAAMNMVGSLGVPPSATQAT.[G]	4218.08794
8520	[Q].LFCGLLAGLACILCQGSGLLWTHGFRGWAPRQLERMG.[F]	4218.11327
8521	[G].YHQGPSLVPSHQASFLEPQQGTMGAAGSSFGLMQPRPPPE.[P]	4219.01241
8522	[P].SPSPAGIPHGPQTVGNHFQRTPITNQSSSLTATQMSFPVQ.[G]	4219.06253
8523	[A].GAAGGAAGGGPAAGPADHGLAGRGAAGDGPAAALLQAAGVAADWAAAGLAD.[G]	4219.06637
8524	[T].SRGRTPGERPSCRESSKAGGERGPAAPSQPVPTAPQTTGPF.[N]	4219.08097
8525	[L].LGSAGGGGGGGIGLGSLGGGGGLSSSLGGTATIGHLRGSSEHHFSNTL.[G]	4219.0875
8526	[R].QGQYAPPPGASNILGLEASGHVAELGAGYQGPWKVGDPAMVL.[L]	4219.0917
8527	[P].GPRGKEGGAAESGPFANKRQLDGLGPKGEGGLPTCGPPDKAST.[A]	4219.09489
8528	[K].GDPISVGNTKTSPPGKVNPESSGEIDSASSGPVGPPSSEKAEAV.[T]	4220.04771
8529	[I].EGSVFRPGSKTFVVNSNMGQNYKIGDYVALDLDTGRPST.[T]	4220.0717
8530	[F].QVGMKLEAVDRMNPSLVCVASVTDVVDGRFLVHFDNW.[D]	4220.07256
8531	[M].PSRPMAPMSSAAVAGPMLPAGNAQQRTSGPAPAPPQGAPQPGLS.[G]	4220.07978

8532	[L].LKSGGSSPPSSQNATLPSSSAWPLAASGYSRSFSSLAPAPSVAG.[K]	4220.08945
8533	[P].RQPLTYMAQRQPSESGRHLLSEPNTPLSPPGPGDVFFP.[S]	4220.09819
8534	[A].HGRYPHRFGNGRGPRLHPSSSPPSTASPRVHPWVDSHP.[A]	4220.1047
8535	[L].ERGAAPAAAEGPAPGSIFLAGAAPPAPCPASSSILVNGSFLAAGSS.[P]	4220.10808
8536	[S].PQSSAATTPGSPSLGRHPGAHQVSNLHGNVATTPGSPSLGRHP.[G]	4220.10924
8537	[S].GPPGPKGEPGHPGTDGAAGQRGPPGLKGEQGDTVVIDYDGRIL.[D]	4220.11192
8538	[E].KAQDADVGSNSISSYRLSSNEHFALDVKKRSDGSLVPEL.[L]	4220.12182
8539	[S].SARSPESVQPPGNSQRGERKPEEAPRGADPGGLPSVWHGGS.[S]	4221.05687
8540	[P].GPRGMQGPPHPHGIQGGPGSQGIQGPVSQGPLMGLNPRGMQGP.[P]	4221.07636
8541	[L].LPWSMLPDVIDDFHLKQPHIHGTEPIFFSFYVFFT.[K]	4221.09829
8542	[S].GTSAVVCVWELSMTKGRATGLRLKQALYGHTQAVTCLAA.[S]	4221.17295
8543	[G].FVFGNLPDLSMCAQKRVAWHLFGMGNEVDVHTVFFH.[G]	4222.02483
8544	[S].TTGGLEQDVAQLNITEQNWSPGQPSFLQSRDLRGMPNH.[I]	4222.03704
8545	[L].PGGSIPQMLNGEVYPPSVEEAPVLMRYPEGIPPQSQMAVG.[Q]	4222.06575
8546	[P].GTPGATPPPPQFPAGIWGSGLGGAEGGAPASRAGLPSGPGPGCRVT.[R]	4222.08868
8547	[S].VGTGSRSSSRRGSMNNELLSPEAGPVRDPLAEGAEGLGRASP.[E]	4222.10177
8548	[Q].SLGNAAPHAKSKELNGSCMRPGLVSEPLPAPSGSPPPSAAPTS.[T]	4222.10195
8549	[G].HKGERGYPGNAGPVGTAGAPGPQGPVGPTGKHGNRGEPGPAGAVGP.[A]	4222.10376
8550	[S].TIGAESIRDEEAAPGQAAVTVRGGADGKTVTMSVPGAAMTIVH.[L]	4222.12308
8551	[A].QGPQMQGSAQKAIPPGASPVSSLSQASSQALAVAQASSGASGQSL.[N]	4223.0997
8552	[C].SIFPDLSFVTFDGSHVALFKEAIYILSQRPEEMVTVH.[V]	4223.15218
8553	[V].GPPPPSTARDSTQAGVLRSLSRGHFINLNHHKTMSNRGL.[R]	4223.17515
8554	[E].AMVEAGRGPEALQTGSLGLQEGSGGAAPGAGAPRSSPDGDGQGVFG.[G]	4224.00105
8555	[P].GPQGQFRPPGPQGQMGPQGPPLHQGGGGPQGFMGPQGPQGPPQ.[G]	4224.99916
8556	[G].LHREGEAWAPADCRLPQGIYRVNGVKTRVEKLCQAF.[E]	4225.16583
8557	[Q].SPKQEAGGAAPGQHRGQATGAASRPSGRGQGLAEKQEEARKL.[T]	4225.16815
8558	[V].TVAGSSPPAVPSHSMVGITMDVGGSPIVSSAGAYLIHGGMDGSR.[H]	4226.03149
8559	[L].WLSMLPSGPGALWLETQMVGLGPCVLQQHFLHSFMEP.[A]	4226.03703
8560	[S].KPEASPRSWAMAAGRPLCRSLRRELGPPESQQPREQP.[E]	4226.15705
8561	[V].MEEAALVMLEKAEAVEVMDRVMETRAVAMVGEVAMVGEA.[A]	4227.01841
8562	[A].KETRTAENTSTKEASTSTTTVEPPTTPKSPAESTPQPPIE.[S]	4227.07867
8563	[A].VAWPSQTAEPGPAQSSLPAVVVETFSATVNGTVESGSGAGRLD.[L]	4227.08403
8564	[L].ANGIAGSMATLLHDAVMNPAEVVKQRMQMYNSPHRSALS.[C]	4228.05185
8565	[D].GRVWAEQTGSSSLPPQVAQHTFTSLPSAGPQSGKIQDQYS.[S]	4228.06939

8566	[P].RHRSQPGGAAPPGHQMFAIQPGAAEGGQFLGGPPPGVCPPEL.[Q]	4228.07159
8567	[M].DSPILGDYRAFLDEMKQCFGWDDDEDDEDEDEEDD.[Y]	4228.61899
8568	[A].PTVNSSSRSSSPSKVMDEGKVSMAAKGPPPFPGAPLMSSPVGG.[P]	4229.06754
8569	[W].NSAVAFSQELQRSLSPLMLPQPSSLTPGPMSGASGGAALCLT.[G]	4229.10392
8570	[H].VSPNHSSSAPVTAVPPSRLSPPMLEEMAYSAYVPQDGTML.[T]	4230.01919
8571	[M].TTLHKMGAPGSGIAEYLFDKHTVGHSGGSHQLPGQHASVGF.[L]	4230.05738
8572	[G].QPQAQQVPGGQWETERVKEEGALAVARGQWAMPGAVEGPS.[W]	4230.07851
8573	[Q].QSQQGLSPSHVAGSSSQGQALQQPPQGSTVQHTYLPNTWN.[S]	4231.01875
8574	[S].PGLSQPSGVYASSSVQDFRHPPPQLLSTSNRAYMRNPSS.[S]	4232.05778
8575	[G].PSASASAGPPASARPGTSAAARAATSASARADMSATARPGPSASAHA.[G]	4232.06096
8576	[I].PSDQQVINEMVRELDGHVLKCVKDQNGNHVVQKCIE.[C]	4232.06452
8577	[V].PPAAWSAPFSAPKDEWAAAYGPGPTAPTASPAPLAFGPPPDFG.[A]	4233.03926
8578	[P].SESTLDLPDGGGGRRPGASVVSSASMSALHTSSLRDYTPASR.[S]	4233.0589
8579	[G].RQPQPSGSNRRGWNTTSQRYSNVIQPSSFSKPTPWGGS.[R]	4233.07212
8580	[A].YGPGIEPQGNTVLQPAHFTVQTVDAGVGEVLVYIEDPEGH.[T]	4233.07749
8581	[L].SSPGSVMATDKLGKTDAAEALDLSERLYMDMSKTLGSLMN.[I]	4234.03861
8582	[W].PQGPAGPPGPPGPMGPPGLPGPMGIPGSPGHMGPPGPTGPKGTSGHP.[G]	4234.04193
8583	[E].KGEFTNLQGVSAASSGRIVVADSNNQCIQVFSNEGQFKF.[R]	4234.06219
8584	[L].QFMVSVATMLRADLNLPENNDLVQEDMAQVLELETQL.[A]	4234.07162
8585	[T].GLEQGPSAGAASAGPQVSLYQGAPPAAEQGVVSRDLRVWEVE.[T]	4234.11634
8586	[P].PGPSGPLGHPGLPGPMGPPGDPGIQGYHGRKGERGMPGMPGKH.[G]	4235.05965
8587	[A].QGVHDGVNQAGKEAEKLGHGVNHAAGQAAKEAEKLGQGVHHA.[A]	4235.12013
8588	[S].APAGTQGTRSTRCSINHFIFREGAQITFTVSFHVAPTAT.[L]	4235.12032
8589	[L].PGQVEREPPVSIFWQWDRLALTNASSTTLMPNGSLHLA.[A]	4235.13424
8590	[S].QQHKQGSGGGSSGGGTGSSNGGGQAQCASGPGTLGSCFESRVVVV.[C]	4235.91773
8591	[E].DSTLLMQTLMEAIQISEAPPTNQATAAASAPNASPQSSQPPA.[V]	4236.04349
8592	[A].ARRQGSLSASECLYGAPPAPLGPGMAFGSPAFPPHTVMLHA.[G]	4236.05758
8593	[L].VSGVLGSALTTGGPSLSAMGNGRSSSPTSSLTQPIEMPTLSSSP.[T]	4236.06462
8594	[E].KEDRFLTTLSSQSSTSSPHLQLPTSPEGVPEQAMGGPPEL.[D]	4236.07651
8595	[A].ASHQLPGYAATPQPTGLSGVFDTSVKSASTNTKEPSVMNFL.[S]	4236.09176
8596	[G].GAGGGPGKPGMGGTQGRAEKPLAAGPPMAPGTTGSRTTSARCRGV.[P]	4236.09313
8597	[G].EKGPRGKPGLPGMPGSDGPPGHPGKEGPPGTKGNQGPSGPQGPLG.[Y]	4236.10031
8598	[E].VGDEGLGGGVVWRSGEPQRPTGQRVGGLSMTSVSHPFQSQV.[E]	4236.10031
8599	[P].AQAQAGQMMPLSSARPTSGSVGVMLAAGRNEALQVPAPRGEGA.[E]	4236.10705

8600	[M].PAFSLPYAQARAHHTPQSLGLQAMPAAGGVLYQPSGPASFAG.[T]	4236.10836
8601	[A].GPSVTSVPQLSQELSGLPAPSMGLEPPQEVPEPPVMAQELP.[G]	4236.10905
8602	[R].NSPTDTILFIRTACRAHSGTYQVTLRVEDMEDKAQLV.[L]	4236.1176
8603	[G].DGQPLLLGRRSGVSECAELEAGLHAGGAWSSPSKLSGVRGSS.[R]	4236.12144
8604	[A].QRMMPVNRTAKPFPGSGNQPAAPFSPSRNVTSPIADFPAP.[P]	4236.12296
8605	[-].MQAPGGVPGVEATLYWVEAVEEGAVLEEGEVSGIGQDVQLLV.[L]	4236.12344
8606	[S].KYSPKDGDLEFSAQSLGSKVSSEAHAGTSFPLQAGEPLAVM.[V]	4237.07578
8607	[A].EGALHWDLPRVQGGSQLSGLFQMDVPGLPGPPGQGPSASAPL.[G]	4237.1135
8608	[L].QRNRKMAMGRKKFNMDPKKGIQFLVENELLQNTPE.[E]	4237.1791
8609	[S].PGAGGGVNERRRHAHSAPSAHPGMAAAQGPVAPSSPEQNGAVPS.[E]	4238.0405
8610	[F].GIVPHAGMNGELTSPGAAYAGLHNISPQMSAAAAAAAAAAAYGRS.[P]	4238.05058
8611	[P].NPNPIPTSGSGSSPIPDSVLSSDPKPGRNANSDLLASPDHRS.[G]	4238.07084
8612	[P].CPLQGAAKGLVPTELPEPADLVEADGAPEPGSWGPVPSTAGSL.[G]	4238.09618
8613	[P].ASASAPAPVPAPAPAPAPSPAPASSSDPAAAATAAPGQTPASAPAPAQT.[P]	4238.10002
8614	[L].VTATTMFFQYLLQPFRDMRELAASCKLAILKSLDED.[D]	4238.13343
8615	[T].PAGAVSTPEQSATPAGAVSTPEQSATPAGAVSIPEEPDAPAGAVPT.[P]	4239.05754
8616	[P].QQLLGDSTHPQGAGRSGQNLLGQASATSHIYQGPESSLPGPP.[S]	4239.08135
8617	[L].AVDATRARCTVGEITDAMKKVFGEHKANDRMVSGAYRQ.[E]	4239.09682
8618	[A].GFPGPPGIQGNPGPVGDPGERGPPGRAGLPGSDGAPGPPGTSLMLP.[F]	4239.104
8619	[F].ANVSTVADHFDHIRAVMGSEFIGISGSYDGSGRFPEGLED.[V]	4239.96762
8620	[R].KPLPRMDQSSSRASPGLAQKGCGESAWARSTTTRESPVH.[T]	4240.07344
8621	[A].GVSVSQYEEQFVTLFTLTMMQLKQALHYMLLVSEVE.[E]	4240.09023
8622	[C].GGTVAQPMSPAPGPADPGPGPEGRAPHSAIEEKVMKGIEENM.[L]	4241.99002
8623	[Q].DMAPSASPKLEPATGPAMQAGGPGTPQGPASEHKTPWPLMET.[L]	4241.99404
8624	[G].QLNSSANSLFILYYTAQGEPFPNNLDKLCGPNVTDFPP.[F]	4242.04884
8625	[V].PQGHFDSFTVQYKNGDGQPQVVRVPGDEDEVTVSGLEPD.[H]	4242.98505
8626	[P].EPVSWREGPSGHSTLPRSPRDAQGSATSELSGPSTPLHTSS.[P]	4243.03988
8627	[P].SPQQSQQGLSPSHVAGSSSQGQALQQPPQGSTVQHTYLPNT.[W]	4243.03988
8628	[P].LFHTIEDFWRMIWEWKSCSIVMLTELEERGQEK.[C]	4243.04495
8629	[S].GSNLGTKPQMFQGANSLGGPNRNVTVNQAPSSGDWGLPSSKA.[S]	4243.05851
8630	[A].AQNGQSPMAALILVADNAGGSHASKDANQVHSTTRRNSSSPP.[S]	4243.06572
8631	[G].KHLMAEASGKGVTYLPSEERAPGLPNHGATFKELHPQTE.[G]	4244.11931
8632	[K].ERGEKGEPGARGATGAKGESGVDGLMGPPGPQGPPGDPGPPGTPGL.[D]	4246.05817
8633	[P].SSAAMAAPGHPLLLDNSPRNGSVMGPPFAEPPTAEMGVKGSSI.[P]	4246.07296

8634	[Y].SPVASQVTHHPQPTLWGYSLMGQPQQPGFFLQSQPVTPG.[G]	4246.08147
8635	[A].GAPAPFVWTRCGVLGWSGPWRSAPTQAGSSTTVCTGKTPAS.[S]	4247.05494
8636	[P].PAGPTSPSGAHPGEKPLVDLPGEAPTGPTDAAGKNMALTSPREA.[A]	4247.10372
8637	[P].PQLLSTSNRAYMRNPSSSIPPPSAGSAKTTAPSPTPRSHSP.[A]	4247.12619
8638	[T].LNVNDPSPVPSSSSTYHAIIGGIVAFIVFLLLILLIFLGH.[Y]	4247.38825
8639	[R].GSPPVPSGAPMEEDGLRWTPKSPLGPDSGLLSCTLPNGFGGP.[P]	4248.03762
8640	[L].FPMHSVAPPVFGNGFRADSFSSLASSYAPFVSGAGPGLPGGAH.[K]	4248.03961
8641	[G].GGGTRGANGGRVPGNGAGLGPGRLEREAAAAATTTPAPTAGALYSGS.[E]	4250.14093
8642	[S].SSPNPLPQGAAPPSPPGPPLPPSAAASLGGSGAPPPPSMPPPPLGSP.[F]	4250.15905
8643	[P].TLQPADGGAGPAGAGGAAAVATGPQALFSGGADLLGLQAPPTVLTHQ.[A]	4250.18402
8644	[D].LFDSGDIFSKGIASQSAGRRKAKVKAANSLANLAEGSKDRS.[P]	4251.25925
8645	[L].ATAEPLCLDPSIAVACTANRLLYNRQKMNTRPMKRC.[F]	4252.10284
8646	[W].GPPAPGPRPQPEPEPAAGEPGRAATAPTAGGEPLSPPPPQEPAPG.[A]	4252.10577
8647	[E].AELGEWTSVKRSFDQAQPQPQPQPQPQPQPPSSNKRPS.[N]	4253.11226
8648	[A].AIADQPEGVLFMSPEPAPHAATKDAGPASSLPSLPAALASVAAE.[Q]	4253.14346
8649	[V].PQAAQAPTPGGGASSAGTFPSAPASLAPASAVGSAVASAAQLPPVPSQ.[P]	4253.1473
8650	[W].QPNLNSGATPTSYIIEAFSHASGSSWQTVAENVKTETFAI.[K]	4254.06257
8651	[L].NGTANDTAGPELPDPLGGLLDEAMLDEISLMDLAIEEGFNP.[V]	4254.99448
8652	[T].PEKDEEVLEAGPEGDEGQPAEPHEVALAREEVNMLLAEQ.[R]	4254.99832
8653	[A].VPTGGAPGSPGGLEEIFGSLDSLREEIEQMRRPTGTQDSPA.[R]	4255.05717
8654	[H].AVAGDPQSVVAAIDRYSSEKEWAMHVGDKKGGWRGGSGAGPA.[G]	4255.07376
8655	[V].KGTTSHRSFTHSRSAAVTSEFHLVPSRSMNGQPLTCVVS.[H]	4257.10402
8656	[T].QQLQPQQNLQMQPPSMPPQPNLQPPPPQPHLGVGSAASGH.[L]	4257.10804
8657	[G].RGGNWPGSPQVSGPSPATRMPGMSPANPSLHSPVPDASHSPR.[A]	4258.04175
8658	[V].VEFHPNEYLLASGSSDRTIRFWDLEKFQVVSCIEGE.[P]	4258.05498
8659	[T].GERGSCLRALGHGGGSDARSCRRDPLVSEVRQDPGDPLAP.[Q]	4258.07009
8660	[E].GAAGRAPAAPPGGADPEPWAAPAEPREGRQGAVPEAEGAGVQER.[V]	4258.07727
8661	[S].RVDDLHTLFPGGDRNGLSGDIRMRNTTSSESVLTDLSEP.[E]	4258.0793
8662	[A].AAAAAATTPATPSGSAAFQPPRFPPPAPLYPAGSAGPTQNGFAAH.[Q]	4258.11046
8663	[L].WPSPARPSPAGGLSQDVASGRLDEKMPGLAGQAAGSGDRPRSA.[S]	4258.11702
8664	[M].MKKSADHKNLEIIVTNGYDKDSFVQGVQNSIHASPSLNG.[R]	4258.11971
8665	[V].SPSAASPLAAAPTAPAPEAPQAKQEAPGTKGPDPEPTQPGASKSP.[P]	4258.12623
8666	[N].AGPVGTAGAPGPQGPVGPTGKHGNRGEPGPAGAVGPAGAVGPRGPSGPQ.[G]	4258.16127
8667	[S].WGTGVLGAVWGSWSPGASGAAFGNRGAGVARATWGARVAWAAC.[G]	4260.08454

8668	[Q].VQTQSPTQPSPGPGPGLQSVRAGAPGPGLGLCSSSPTGGFVDASV.[L]	4260.09897
8669	[G].PPGPQGHLGPQGPPGTPGMQGPPGPRGMQGPPHPHGIQGGPGSQG.[I]	4261.03152
8670	[D].LMLETYGKMVSGGAGISNARPDLTDPAGYGAELAGPHLQASE.[K]	4261.054
8671	[E].SFLYQSGESYRADVVDLFPGTFEVVEMVASNPGTWLLH.[C]	4261.05867
8672	[E].ELGNDKEPLGSPALPPVCEGEAMPAPRTEDGNGDLLPAPPPG.[D]	4262.03801
8673	[G].PEVSASGVEDISGLPSGGEVHLEISASGDLSRLPSGEGPEVSAS.[G]	4262.05827
8674	[M].AMGVAGPASLYHSGLTVGMISGGGVVCVQEARAGYVGSLGCLH.[L]	4262.06135
8675	[-].MAMGVAGPASLYHSGLTVGMISGGGVVCVQEARAGYVGSLGCLH.[L]	4262.06135
8676	[G].EGRTGGGRGEAEKASTSGLGFKDEGDIKQAKKEDPDDRNR.[M]	4262.07805
8677	[G].QDALTLSSEKASQDSVTPSVVEENGEAKELHPCKYCKK.[V]	4263.05439
8678	[-].MLVPGACAVTSHPHPSPHPHRAPALSPGFAAAAGIGHPGAGGHA.[R]	4263.09881
8679	[S].ELLDSNDGLGSSIPSPSPPDPARVPSHSLSCRRKGILKHS.[S]	4264.18913
8680	[T].QPAPDQSSTVQLHPATSPAVSPTASSAVSLAVSPAASPEISPQV.[S]	4265.1572
8681	[N].TSRSGGFLSNQGSFEEDDDDDWDDWDDGCTVVEEPRAG.[G]	4265.70209
8682	[T].GATWRTSGLLEELNAEAGHLDPGFLASEKTSAGNAPLNEEI.[N]	4266.09493
8683	[P].PGGSPGRSSPAGGSPGKPGSTPHVSGLGSPGRYSPANGGHLRRMA.[R]	4266.10819
8684	[T].PATVVPTGEGPRGPGQVGGEGMGRGGCPRPPLTLCSLTGPELP.[V]	4266.12164
8685	[F].GPSPGVEPVASMTSVASHPALGASSSSLPPLGPAAMNMVGSLGVPP.[S]	4266.12432
8686	[A].PGPPGTSLMLPFRFGSGGGDKGPVMALRGPPGPMGYTGRPGPL.[G]	4266.14092
8687	[G].APAAAAAASMSAPAPSHPLSDVATSVIQAEVTVAPGTGVMPPSGLP.[V]	4266.14208
8688	[L].GPAVKQESSLARCQLSRQPPGPEPPFSPSLTAPAASHADAR.[A]	4266.14726
8689	[E].PGAQTLGRAGVGSSKGASEEFPVLPGPQDTAREVTGQGASGRSV.[P]	4266.14976
8690	[C].QKQQAAPVEVAAPEERVVALYDFQARYSREVTMKKDD.[V]	4266.16118
8691	[S].GPESPRLDSLEAGSPRHPQRPETQSPAAPGPPLRPPETLGS.[P]	4266.16502
8692	[H].QFFKTSGKMGPGFTKALGHGVDLGHIYGDNLERQYQLR.[L]	4266.16654
8693	[G].AALPSASPSGSLSPPPPPPGLYFSPSAAAVAAVGRDARLACTPH.[Q]	4266.17644
8694	[Y].PAPGDGALLLASYTWSDSAAPFAGRSLAESLRLALDDVAALH.[G]	4266.18296
8695	[A].IAATPVSAIPAALGVNGYSPVPTQPTGQPAPDALYPNGVHPYP.[A]	4266.18698
8696	[W].SPAMKEPLAKILCHRGGVRAVAVDPTGTHMATSGLDHQLK.[L]	4266.20564
8697	[M].DAEPVGEPEPVLSAASFPGGGTRVPERHVGDGGGGARGDGAERP.[A]	4267.05111
8698	[A].RAAASAGPSASASAGPSASASAGPSASASAGPPASARPGTSAAARAATSAS.[A]	4267.06822
8699	[A].FDAVSPVHKDGDKALPEEEPQPRAPAQEAGYSTLAQSHPP.[D]	4267.06905
8700	[A].AEPPQPQPQPQPQPQPQPAAPEGPAQPRPEPSPWGPLDEV.[R]	4267.08431
8701	[G].MPYQAPPQQLPAAQPPQPANPPHGAHPLSSGPQPGTAPATQH.[S]	4267.08902

8702	[Q].QPYISGQQPVYQQMAPSSGPPQQQPPVAQQPPAQGPPAQGS.[E]	4268.04654
8703	[L].KGKVMASMFYEVSTRTSSSFAAAMARLGGAVLSFSEATSSV.[Q]	4268.0672
8704	[P].APGRQEGPAGAAGAQAGAREGVDRNSVPRRGDAMPEAEAGGVAGP.[S]	4268.0722
8705	[T].AAEPQPTAPPTVCVTGPPTARPSEGPTTGPTGPPAAGPTGPPTAGP.[S]	4268.09283
8706	[C].FQPCQAPLPGTLERGGHGEDRGALLATGSLTGCLVWLFAD.[H]	4268.10156
8707	[S].LGNTPSSPPASPTSPRASSEASPDPQPPEKPGRAKENTDSLN.[P]	4269.06542
8708	[P].GPKGEMGPVGPAGNPGAKGERGSSGLDGKPGYPGEPGLNGPKGNPG.[L]	4269.07416
8709	[-].MNRVRQQGRRNQVPPGPSPLPMVSVGLGCWHQGTHATF.[A]	4269.12398
8710	[R].PGARGEHQAHTQAGVGGAPPLPSSGIGGRCHSRKSCPSPAEGP.[P]	4270.04896
8711	[R].RAAAQEAEGEAQQRAPGGKAAAGREPDPVAADGSHLYAEAAPGA.[P]	4270.06201
8712	[A].AAHLENGIALSGLESCVMSAPPGSGPLEVTTDSLPNGPALADGP.[A]	4270.06423
8713	[Q].VGQGIGIPSRTNSMSSSGLGSPSRSSPSIICMPKQQPSRQP.[F]	4270.11253
8714	[P].GLPPAERGGGGGGRSKSSSYRVLENSAPHLLDVEADSGLLYT.[K]	4270.1487
8715	[A].QQQAIEEPRAFHPPNVSPRLLHPAAHPPQQNAVMVDIH.[D]	4270.18392
8716	[T].SGARSKTESKAMVGARPKTESQTMAGARPRTESQPLAGARP.[K]	4270.18914
8717	[E].GPLGEGGASEAPRELAGQSQRRGGGAESGWVPGTSAPRGTAPQE.[P]	4273.07291
8718	[A].GPPPNSMGLSNSLAGSNGAGLQSHLYQPAFPGMVPASLPGPSNV.[S]	4273.08049
8719	[S].QGPSVSDVRMPQYSTFAYFPTEVTPHAFTPSSGQHHSVP.[T]	4274.00362
8720	[L].ASPGSQSKEGSPVSKMSVSRSSSLRSSSSLSSQGSVASSIGSQT.[S]	4274.08009
8721	[G].AGAGPPNPAINGSAPRDLFDMKPFEDALRMPPPPQSIAMAE.[Q]	4274.08313
8722	[P].LLEAEVGEAASHLASYALGSSSANVEALPQESLDRMMANLL.[K]	4274.09553
8723	[A].AAGGGGGERTPAPGALEPDAAATRAAPNPASLPNTLGSGYSPRLC.[P]	4274.1007
8724	[P].GPPGPPGPPGVPGSDGIDGDKGPPGKAGPPGPKGEPGKAGTDGPDGKP.[G]	4274.11125
8725	[D].VHYAAGQAGKEGEKVVQGVHHGVNQAGKEAEKFGQGVHHAAG.[Q]	4274.13506
8726	[P].APAPASASAPAPVPAPAPAPAPSPAPASSSDPAAAATAAPGQTPASAPAP.[A]	4274.1364
8727	[H].QRESNSDIPKNSFTKSLDSCRSQVLPQEGQVKESHSTA.[T]	4275.06946
8728	[V].TGVLNRTAERMTPETPSPITPTRGTWGGDHTSPAVTAGTTH.[R]	4275.08472
8729	[P].PDHVDPPPDTDALIPRAPEHHPSVQNPDPPPRLDPSSAPP.[P]	4275.08537
8730	[S].LFPALVQTNCYKYLMFFACTILVPKCDEDAHQRIP.[P]	4275.0898
8731	[A].TDFSPKQISLSWFRDGRRMVTGVSEGQVETVQSSPVTF.[R]	4275.1139
8732	[K].EREGEPAALRAGEHQPGPADDLAKRSDKEAAPVASGPGRAPN.[P]	4275.12495
8733	[K].TEPRRSQNFYFSERDSVIPSSNLVMPAFQDVSLSFPQ.[Y]	4276.07678
8734	[H].EGRQKDKGSAEDLPVVLECARLLERMYSHVAARAEEF.[R]	4276.12375
8735	[L].TLSPGPEAHQGFSRQLSSTSPLNPYPASQMVSSDRSPLSF.[L]	4277.05677

8736	[R].GAEAAAAFLGMGFSSLDMSLCVLLYVASSLFLMVMYFFF.[R]	4277.05801
8737	[P].GPSPQFQSPPAKQTSAFSKQMPHHPFPSPAPDLCQVPPGP.[G]	4277.06953
8738	[E].LQPSSLETDQPLQEIMEMASDKSKKSAGDGEDPKTETQP.[P]	4277.99118
8739	[I].PGGYNVLRAMYTDIMDPMLNAVQEQFGGNPFATTTTANAT.[S]	4277.99404
8740	[G].GRGPHGGPSQPAAPRAQGSFEYQDTPDRDYGGVAQPVAEGTP.[A]	4277.99835
8741	[D].STQKEQYWDLMLETYGKMVSGGAGISNARPDLTDPAGYGA.[E]	4278.0118
8742	[P].SAHCRAPSATGLHTDMDLPGRGLANPAPSCYLLGSEPSSGLG.[P]	4278.01248
8743	[A].RPAMQQQPQPQPQMLQMRPGEIPMGMGVSPYGQAAPSNQ.[P]	4278.01335
8744	[T].LQHPEGAESGRGAEPGGPQPPGERDPGSLQHPEGAEPGSPQAL.[G]	4278.01948
8745	[E].PGDVSAGPRSGGGRNATTAMPPPVPNGNLHPHDPQDLRHNGN.[V]	4278.03542
8746	[M].AMGVAGPASLYHSGLTVGMISGGGVVCVQEARAGYVGSLGCLH.[L]	4278.05626
8747	[-].MAMGVAGPASLYHSGLTVGMISGGGVVCVQEARAGYVGSLGCLH.[L]	4278.05626
8748	[P].PAAALGATCAAAFPSAASVTSAGATSASSVHLPVSAPHGAGLMAAASA.[Q]	4278.09178
8749	[Q].KMREAGAGPEEMLKLRAAGADMLPAQQKMVPLPFGEHPQ.[Q]	4278.12903
8750	[L].PEVTPANVSGGGGSKSELVITWETVPEELQNGRGFGYVVAF.[R]	4278.13534
8751	[L].FLAMHYTSDTTTAFSSVTHICRDVNYGWIIRYMHAN.[G]	4278.99465
8752	[Q].SSPGLNPGQPSSMLSPRHRMSPGVAGSPRIPPSQFSPAGSLH.[S]	4279.12475
8753	[E].APDSGRPAPYSAAFLELQPGPAGSGYPAAAPPASFASHFLQGGP.[F]	4280.08358
8754	[N].AGPQGHLGPQGPPGPQGHIGPQGPPGPQGHLGPQGPPGTPGMQGPP.[G]	4280.0955
8755	[P].AGPKGDPGSRGPMGMRGPPGLQGPPGSPGQAGAVGIPGERGPPGPPG.[P]	4280.12
8756	[R].GPSGARARARFYTDPVKAVRDITDGSRIMIGGFGLCGIPE.[N]	4280.18154
8757	[Q].VSSVNEEDFVRLKQQQISDHISQPYGSGKMKMFQEVP.[A]	4282.09072
8758	[T].PATVVPTGEGPRGPGQVGGEGMGRGGCPRPPLTLCSLTGPELP.[V]	4282.11656
8759	[S].GPRDGNRQPEATHTVLEMVRAMGFTPVDMGSLASAREVEA.[M]	4283.07542
8760	[G].QGPGGGQPRTGMPAIESLPPERPQPPTLASTPMQNGGLRDSS.[Q]	4283.09318
8761	[L].QPRPSVCPVSSFSLDSVARDRRELRRFLTQNLSLPDS.[A]	4286.22109
8762	[K].FPLENMINTATSQPATSIGDITSSVTPATIVSSPKVTLSGRP.[D]	4286.22244
8763	[F].PGSPGEKGEKGSTGIPGMPGSPGPKGSPGSVGYPGSPGLPGEKGDKG.[L]	4287.09864
8764	[Y].GPLDMVTLTGEKVDVHIMMQPPSGEWLYLDTLVTNSSGR.[V]	4287.11343
8765	[L].EMKTLSPQAPSPLGSPFLWPGVEGPDSPSSPKPGAPHATPHT.[G]	4287.11792
8766	[G].PGAAGADAAVPSTPAGQDLLSSGEPQPLPSSPGAEPRTPSRAPPAG.[E]	4287.12763
8767	[P].AGPGTGGPGVASPTITVAMPGVPAFLQGMTDFLQATQTAAPPPPP.[P]	4287.14644
8768	[F].APQPGVLGQPSPALHTQLYPGPSQDPPPPHSGALPFPSAGPPQ.[P]	4287.16217
8769	[-].KNNCVLPEDLKNFYLMTNGFHMTWNVKLDEHTIPLG.[S]	4289.09805

8770	[G].AGAGPPNPAINGSAPRDLFDMKPFEDALRMPPPPQSIAMAE.[Q]	4290.07804
8771	[A].SNTEAVLESWASIPQKPDAAAAAPTVEDAVCAAVPVPEGAAAAS.[V]	4290.08707
8772	[T].TSPGGGVAVAGVGAGSVGGAVSLAATRSTSSSQMSATLAGAAAKSPGESG.[S]	4290.12664
8773	[L].WFPTSTISTQKSGQIIYGCAFLLVLLLIILSVILAQWP.[S]	4290.40146
8774	[T].VGQQGLGSVKDPSNCGMPLTPPTSPEQAVVGESGGMQSAVSHL.[G]	4291.04278
8775	[Y].YSVTPPPGSAPGPGPLSGSQGPGQQCLGQAGLPGSVPASTHSLTH.[S]	4291.08366
8776	[P].RQSHSGSTSPCPKAKSQTPPGHNLLGSKSPCSQEKSKDSL.[A]	4291.09424
8777	[P].PAGQAPFQAQPAPPASRMLTGSHSFAASGMAGVPVVPLRGCGQ.[V]	4291.09576
8778	[P].YLCRRTSQLDPDKPSSLQCPRPLPTVLDDPHEVEKSG.[L]	4291.12341
8779	[G].QPEISGEASGILSGLGPPFGITDLSGEAPGIPDLSGQPSGLPEF.[S]	4291.12925
8780	[V].WGVTPALHSPLMSVTNAISGLTAVGGLVLMGGHLYPSTTSQGL.[A]	4291.22536
8781	[P].GLPGQQGTPGQPGFPGPKGEMGVMGTPGQPGSPGPAGVPGLPGAKGD.[H]	4292.0863
8782	[G].KQMVDMLVESSNNVEMILKFFDMFLKLKDLTSSDTF.[K]	4292.08852
8783	[L].RGGSGLSHAGWPGATPSMSELERRRLEEALEAAQGEARGAQ.[L]	4292.09735
8784	[A].ADIRPHGVHMVLNQQGRPSGDAFIQMTSAERALAAAQRC.[H]	4292.09822
8785	[I].SGSSGLLGSAGGGGGGGIGLGSLGGGGGLSSSLGGTATIGHLRGSSEHHF.[S]	4292.10388
8786	[L].LTPGQMGGNREAMYVVGLPQAPLWSSAERVSVGPCPPAAPQ.[P]	4292.10493
8787	[M].AANPRAHPDRPACSAAAAPGHALGRDEAAPLNPGMYSQKAAR.[P]	4292.10608
8788	[T].EQWLQAETERLQEELESLAGQLQAQVKDNESLSHLNQ.[E]	4292.10656
8789	[S].GQAKSSSKESKDSKTSSKDDKGSTISASGSSGSSTKNIWVSGL.[S]	4292.11243
8790	[M].TQATRQGSTTFVSDRSLETHPTAPSVEAVTVDGFPTVSMAL.[P]	4292.11395
8791	[S].SPCLSTVSQISSVSMASGSVKMTSFAERKLQRLNSCETK.[S]	4292.11417
8792	[-].QIAAFAGCKTTTSAVLVHCLRQKTEDELMEITLKMSH.[P]	4292.1182
8793	[N].TPAPGVGAAGGSLSFGASSTPAQGFVGVGPFGSAAPSFSIGAGSKTPGA.[R]	4292.12584
8794	[E].GSVAASLSCLPARAVEGSTTATCDLSEEELEIKRLVTELE.[S]	4292.12722
8795	[G].NMGPQGPKGIPGNPGLPGPKGEMGPVGPAGNPGAKGERGSSGLDGK.[P]	4292.1299
8796	[P].TASSLSPGPDAPLAPASSAGPGPGLSVAPGPGVSFSPGPTPTPAPTAGS.[F]	4292.13574
8797	[N].PPSSAAFPTASAGSGSVKSQPGLLGMPLNQILNQHNAASFPASS.[L]	4292.14044
8798	[A].AAADTITIATPESLTEQVAMTLASAISEGTVLTARSGTSGAEQA.[T]	4292.14498
8799	[I].YFSDIVGFTALSAESTPMQVVTLLNDLYTCFDAVIDNF.[D]	4293.02941
8800	[I].GVAMGIAGSDAAKNAADMILLDDNFASIVTGVEQGRLIFDNL.[K]	4293.15298
8801	[D].AAIDLFTRSCAGYCVATFILGIGDRHNSNIMVKDDGQHA.[N]	4294.05904
8802	[R].PSGAATTTAAAAASAPAPGPASSPEASPAPGFPFPPPWMGMPLPPP.[F]	4294.06238
8803	[A].PAAAAAASMSAPAPSHPLSDVATSVIQAEVTVAPGTGVMPPSGLPV.[P]	4294.17338

8804	[K].QSTATRNTKYTPNTGPNADRSGSKEGFYMYIETSRPRL.[E]	4295.08981
8805	[I].FYVTYGANQLNAPARLGIMSSEEMGGGRGGPVAYGAIFPGFG.[G]	4296.06411
8806	[N].QTQQQMGPRPPQNNPLPQGFQQPVSSPGRNPMVQQGNVP.[P]	4296.07853
8807	[G].LPGRPGTSIGDEDEKRGLPGEMGPKGYTGERGFPAVYPGPPG.[T]	4296.11423
8808	[L].PGPAGPKGDPGSRGPMGMRGPPGLQGPPGSPGQAGAVGIPGERGPPG.[P]	4296.11492
8809	[S].EENKTDVAKGHTAVADEPNKRSNDGKSTKTKNSFPEKHT.[L]	4296.12394
8810	[R].QREGQFSSLQLVAMQRGVAAAMQYLSSFAFVHRALSAHS.[V]	4296.15532
8811	[K].HVADRQATEKQEKHEAMAGKAKGSRASGKSTAGTTDAGEATK.[I]	4297.1338
8812	[R].QYTSSSSIKGSGGIGGGSSRMSSVLAGGSCRAPSAYGGLSVSSSR.[Y]	4298.05243
8813	[G].QRTIAAADSNVGHSAPMLGGCPLAYGAPSSLNKNTMPVQWN.[E]	4298.05395
8814	[L].PGSLGFGSSNFQSVGQVFPSLGFGTGGFQSVSPNIFSSLSCR.[S]	4298.06113
8815	[I].ALAVYMGTGICGFLTFGAAVDPDVLLSYPSEDMAVAVARAF.[I]	4298.08582
8816	[Q].VFEGFSLKCQTQEKTGVAQVGTTEKPLRKPPARLKKLK.[I]	4298.41694
8817	[L].FPLTHCCGPGLRPTSQEDKATQTLSPASPSQGVMLPCGVT.[E]	4299.03011
8818	[S].GPRDGNRQPEATHTVLEMVRAMGFTPVDMGSLASAREVEA.[M]	4299.07033
8819	[E].GPPGPEGPAGFPGPPGIQGNPGPVGDPGERGPPGRAGLPGSDGAPGPP.[G]	4299.09661
8820	[T].LPFTLRTAASIHGAEVEVQSWLRMSAGFSSNWDPLTQVP.[C]	4299.16554
8821	[P].ASAAGTVPASQPAKSWASLFHDSKPSSSSSPVVSVETKYSPPA.[T]	4301.13607
8822	[P].PGPPGPPGPMGLQGMQGPKGLDGAKGEKGSSGERGPSGLPGPAGPPG.[L]	4302.10301
8823	[V].KEPFQTSAESTAQDGKDLVPQESTVVPHGDAPGLWGGPEQI.[P]	4303.07895
8824	[A].PGTPGTPASLSANSSLSSSGELVEPSVDQTPQASPGLAPNTRGSP.[G]	4303.09606
8825	[S].PGEPKSSVPDTGTPTPASTPQAVKTASSSMPLYMVTSFVSAPP.[A]	4303.11487
8826	[P].SKGGACPSRAKMSMTGAGKSPPSVQSLAMRLLSMPGAQGAPAAG.[P]	4305.1029
8827	[L].RIASGRPYNPSMSKPDAWGVTKGTAELMQQKEATTEQQL.[R]	4305.13906
8828	[P].NSQRSGYGAAAGAFPSTVPGLYNVNSPLYQNPFASGYGLGADA.[Y]	4307.04284
8829	[T].SHTATAPSATPMFGQVATSTAPSLFGQQTGSNVSTAAAAPQVSSS.[G]	4307.05208
8830	[Q].GTPGMAPPPAPATASASSGTTNTATTAGPAPGGPAQPPPPQASASDLQ.[F]	4307.05208
8831	[A].AATAAPAGFFGSTQNKGFGFGTGFGTTTGTSTGLGTGLGTGLGFGGF.[N]	4307.06799
8832	[D].GGSPALSTTASVSVEVADVNDNAPAFPQPEYTVFEAKHGTFV.[G]	4307.07789
8833	[V].QGVQNSIHASPSLNGRCTEEVKSVDENLEQTGKTVVCVH.[Q]	4307.07792
8834	[D].RDVEFLIYYSAVHTPSVVVEMGEPTTKPDGVLGDPAAMVT.[F]	4307.12504
8835	[A].PGVGTPFSYAVPGQANELVLIEWGNNPMEILINDKVAKLP.[F]	4307.24206
8836	[M].AANPRAHPDRPACSAAAAPGHALGRDEAAPLNPGMYSQKAAR.[P]	4308.101
8837	[K].SAESQGRYEIMLSLQNILNGLGAAATPCHRDVYKAARSC.[L]	4308.10705

8838	[T].KEEYRPCSRYVLQQGYRAGCLLDVQGDGPLYFSIRN.[G]	4308.1077
8839	[D].QLGGDLNSTPLHWATRQGHLSMVVQLMKYGADPSLIDGEG.[C]	4308.1176
8840	[R].FTDNLRNITKQNTETKSGTDQTKTVTDSHKALGDSEDVI.[F]	4308.1226
8841	[-].MGTERIAPNEESALKREGSAGKEKGGAHGQDLPPASELGPPP.[T]	4308.13134
8842	[I].KASEAEMVPAIKPPREDFLNSRMLMPQDIMAYRGREV.[V]	4308.1396
8843	[L].HPKSQMGLSAAAPLWGAPGLLLTIALHLALSLSPAQAWAPVH.[R]	4308.3478
8844	[-].MQLGPLSAMASGRGLRPLLLLLLLLSPSPAASASDRPRGSDPV.[N]	4308.38603
8845	[A].MAPASVAPAPAGSGAPPGSLGPSEQLGQAGPTVGPQQQPPAGAPQPGA.[V]	4309.13061
8846	[K].TAMPSPGVPQNKQPPLNMSSSTTLGNMEQGALPPSGPRSSSSA.[P]	4310.04859
8847	[Q].TNPWGAPAAPASTADPWPSFGAKPAASVDPWGAPTGAGTHSAPKG.[S]	4310.06899
8848	[P].WAGAPSSLGNPPLYRSSLSHLASQHQSSGLSATSTASASLPSGS.[A]	4310.10723
8849	[S].TQDPLLGMLDGREDLEREEKPEPDSVYETDCRWDGC.[S]	4310.89109
8850	[C].LCPRPLWCHWHLCLQVGAGGEGGRWTGPGSPACLGAGVSP.[C]	4311.03681
8851	[K].GPLFVSSFNCVFAVYLPEQQAHNRADGSMIVTSDRRNQ.[C]	4311.08222
8852	[E].QSATPAGAVPTPEQSATLAGAVSTPEEPATPAGAVSTPEEPATPAV.[S]	4311.11506
8853	[V].SRTVPSTTTSTNYLAKAMVSQISTQGFKSPFSMAASPKLAS.[S]	4311.16355
8854	[E].GPSTAPPHFGQTGPVFPAVPPALSSAPGAPAAAAAASMSAPAPSHPL.[S]	4311.16554
8855	[A].AQSAPGTDTATPGAAGGATAASAAASVLGGSAAPATAGDTTKSENVAPAD.[R]	4312.04475
8856	[P].LGGGPEGRGAAAAASSCQWGLYGGVAGVAYMLYHVSQSPLFAGA.[R]	4312.07026
8857	[P].GAPGEAGRPGLPGPMGLPGFCEPAACLGASAYASARLTEPGSIK.[G]	4312.09476
8858	[M].PGSGIGTGPGVIQDRFSPTMGRHRSNQLFNGHGGHIMPPTQ.[S]	4312.09993
8859	[S].GGSRSFSTASAITPSVSRTSFTSVSRSGGGGGGGFGRVSLGGAYGAG.[G]	4312.10896
8860	[G].LPGRPGTSIGDEDEKRGLPGEMGPKGYTGERGFPAVYPGPPG.[T]	4312.10914
8861	[R].AGKSGTSTPTTPGSTAITPGTPPSYSSRTPGTPGTPSYPRTPHT.[P]	4312.11165
8862	[R].GSTIRSTFHGGQVRDRRAGGGGGGGVQNGPPASPTMAHEATPLP.[T]	4312.11367
8863	[V].LHSHVGGSRLGSDLEILSNSSATDKEAAIDKKMYSAHQKD.[V]	4312.12625
8864	[E].GPSGHSTLPRSPRDAQGSATSELSGPSTPLHTSSPVQGKESAR.[R]	4312.13009
8865	[G].GPRPPGQHYWPGPEGAPQIPGPHASSVTHFPPPSSLHQRPG.[H]	4312.1336
8866	[T].KGPEQRDSDKKMMKKSADHKNLEIIVTNGYDKDSFVQ.[G]	4312.13364
8867	[P].GPQGQFRPPGPQGQMGPQGPPLHQGGGGPQGFMGPQGPQGPPQG.[L]	4314.01045
8868	[A].TPAVSTPEEPATPEEPATPAGAVSTPEQSATPAGAVSTPEQSATP.[A]	4314.04195
8869	[N].FPNTVLTEDSSTSLFKDLSSALAGMPEVNLSVDTPFPLEE.[E]	4314.08976
8870	[A].QRSPSLESGVGPAPPEPGLMEGGQDQKDRSATVLPTGWPEVG.[T]	4314.10954
8871	[S].STDLELQALTGSMAPRTELPRPDPGGPGGGRQADDLRSPNPA.[K]	4314.11675

8872	[E].KGQTGPTGDKGSRGDPGTPGVPGKDGQAGHPGQPGPKGDPGVSGIPG.[A]	4314.12461
8873	[P].APYSAAFLELQPGPAGSGYPAAAPPASFASHFLQGGPFPLPYP.[G]	4314.13349
8874	[P].KDGSAPGPGEGALLSNGGSGGTSRKRALDEGSNGHAKFRLKKR.[R]	4321.26205
8875	[G].VGQPWHFGAGMTGVSYCNQGCANSWLADKFCDQACNVL.[S]	4321.84053
8876	[L].ARDEFNLQKMMVMVTASGKLFGIESSSGTILWKQYLPN.[V]	4322.1658
8877	[T].SVSRSGGGGGGGFGRVSLGGAYGAGGFGSRSLYNLGGSKRISISASG.[G]	4322.17732
8878	[D].NILLDEHGHVHITDFNIAAMLPGEMRITTVAGTKPYMGAG.[A]	4323.13589
8879	[A].FAREARFPGQNALPGDGLFPLNNQLPPPSSTFPRIHYNS.[H]	4323.18463
8880	[S].SLSESSPRPSFWERSHIALDRFRFRGRPYRGGSRWS.[R]	4323.19318
8881	[V].QPGAGQAGVVQPGAGQAGVVQPGAGQPVVMQPRMYPRGLVQPGM.[Y]	4323.20598
8882	[G].QPGVVQPGAGQAGVVQPGAGQAGVVQPGAGQPVVMQPRMYPRGL.[V]	4323.22374
8883	[S].GVGGSLAEAVGSPPPAAAPTPTPPTRKTPESFLGPNAALVDLDSL.[V]	4323.25071
8884	[G].PSGPGGGARSGGGRPAAANAARERSRVQTLRHAFLELQRTLP.[S]	4323.31542
8885	[E].AGGPDLGVGRNSGSLWPGDQAPEDRRLAPNQRYNQLDFLS.[Q]	4324.12422
8886	[S].ADSPAEPTTSPSFPTKPALRSSTSLGQAQAQAAAATTATGTQPPG.[K]	4324.13278
8887	[I].QSTISIDSNVSPQGSSSRVATTPGLNPMTPVHKGASPYGTPVT.[P]	4324.1514
8888	[F].PFLHPSETSVLNRLCRLGTDYIRFTEFIEQYTGHVQ.[Q]	4324.16079
8889	[D].GVPCPRGSQPWQVALLKGSQLHCGGVLINEQWVLTAAHC.[M]	4324.16887
8890	[L].ALMHGADLVPIFSFGENDIYDQVENSPGSWLRWFQDR.[L]	4326.03492
8891	[L].DRVLMGYQTYPQALTLVFDDVQGHDLMGNVTLDHFGEV.[P]	4326.04818
8892	[P].FYRDNPKKWQNSIRHNLTLNDCFLKIPREAGRPGKG.[N]	4326.25775
8893	[S].PGTPSATPAADPALPTLSSSSATPSQRQWVSAATSANDSFEIR.[P]	4328.10656
8894	[L].RQHGVESGLLWDSAIMGQRQGASEQQPGDRGPGGHPLAPGPP.[V]	4328.11261
8895	[P].KPERDQMLEPPSIALRDSPGSGWGRGTDEYFIRKPPND.[F]	4328.11529
8896	[N].GAGVEPGEEGRRGPGCPTCQEELRGLVLDWVHGRISNFH.[Y]	4329.07886
8897	[P].GERGPAGPPGPQGPPGEQGPEGIGKPGAPGTPGQPGIPGMKGHSGAPG.[P]	4329.12177
8898	[R].FCGHLAAVGGAVGAGLMGLAGGVVGAGMAAAALAAEAGMVAAGAAVGATGA.[A]	4329.12468
8899	[A].GKEAEKFGQGVHHAAGQAGKEAEKFGQGAGKEAEKFGQGVHH.[A]	4329.12964
8900	[L].RAGEHQPGPADDLAKRSDKEAAPVASGPGRAPNPFGDGPLPEP.[E]	4329.13953
8901	[Y].KPSTGAMGDRLTAMKAAFQSQYKSHFVAASLSNQKAGSSAAGA.[S]	4329.1503
8902	[Q].PGQPGAGTVLAGASGLQQVQMAGAPSQQQPMLSGVQMAQAGQPGK.[M]	4331.13293
8903	[I].GGVGQGLPPGSVTGGMGQGLPQGSVIDSVAGAVDSRIPPCPVNSSV.[D]	4331.13946
8904	[T].PKHSPASPSETDNIRGVFEMIVNPGDNILVNEPVYSGTIH.[A]	4331.14011
8905	[H].LQSGQGWTNPLPDEGLHPGPVWGQIGSRAHWDLAGGALPME.[Q]	4332.10433

8906	[-].MSESKRDLSTSTSREGTALNNSNSSLLLMNGPGSLFASENFL.[G]	4332.10485
8907	[R].TSPAVMQPPPGMSLPPADIGPPPYEPPGHPTPQPGFIPPHVN.[A]	4332.12565
8908	[S].VFEVQCSNSVLPFSFETLNLGNENTDSSANMLGKTQSRL.[L]	4334.07038
8909	[L].HLLIDSGERADITDVMDAYGQTPLMLAIMNGHVDCVHLL.[L]	4334.10763
8910	[L].EDKDGKSRGIGTVTFEQSIEAVQAIYGLGGIGMGLGPGGQPID.[A]	4334.1609
8911	[L].QPSWQGPPAALQGQPGAPLAGANFPMGSAKSLMTPSGESRASSI.[D]	4335.1285
8912	[V].QPGAGQPGMVQPDIGQPDLVQSGAGQAGVIQAGAGQAGVIEPGAGQA.[G]	4335.14223
8913	[V].QPPQPQPQHRWVAPRNRGAGFNQNNGAGSENFGLGVVPVSA.[S]	4336.16194
8914	[I].GAPDLGLPSLPWPPTSISMEMPAAPGSQNQVLGAEDSQSLPPP.[P]	4337.11045
8915	[L].QQTASSGNLNTLSSLHPMGGLNAMQLQNLAALAAAASAAQNTPS.[G]	4337.12487
8916	[V].RGESPQSLGATNSSPTPVGRGAQVGPGQGPVPQDGGAAPSEPRGTA.[A]	4337.12534
8917	[R].GQLNGSAGPGHSELTLLWDRTGVPGGSEISFFFLEPYRSSA.[C]	4337.12617
8918	[G].RWQDVSVGSWNQPPRLGRQMSDGVGEKLFQDLYPFML.[G]	4337.13828
8919	[T].INTDDKHSNTEVAVTAVVSSTPSVVMSTVAQGVSTSAIKMAST.[R]	4337.14869
8920	[S].LVLMGGELADGTLTSDIWAFNPMGGGRWELLAPPASSPSGPPG.[L]	4338.12095
8921	[P].APGVFAGLHCPQDLARPLFSSSGATHPAANPFGPSAHPGSFLP.[T]	4338.13015
8922	[E].WDAVMLHSFTLRQQLQTTRQELSHALYQHDAACRVI.[A]	4339.16114
8923	[E].TQSTPFANTTHKPLAKAKNFQETAASSSGQPPRDPELETR.[M]	4339.17016
8924	[E].KPSESLEKQPRMEEVTLPEDVRVGTPPSDVSTRNLLSDS.[E]	4339.1722
8925	[Q].TGGQPLNFKAEPESETSILLSWTPPRSDTIASYELVYKD.[G]	4340.16089
8926	[L].KMPQQARDAESIMLNLAGQLIMMQRDRSGPQIREKDGN.[P]	4342.16352
8927	[W].SAVSLTAGSMIGSGIFMAPQGVLVYMGSPGASLVVWAVCGLLAT.[L]	4342.1994
8928	[D].EGIRPGTTMEGLAKLKPAFKNGGSTTAGNSSQVSDGAAAILLAR.[R]	4343.27761
8929	[S].SPGELQAAIDSNRREGRRQPYTAAPLFGLAGQPPRGTSGPAP.[E]	4344.23444
8930	[A].CLNISAAILEILNAWENGVLAFESIQKITDNIKGKVCSL.[A]	4345.29344
8931	[C].QDQIHGAGFVPSVLSEENKTDVAKGHTAVADEPNKRSNDGK.[S]	4346.13959
8932	[L].GAFPVLCSPTVPGPGSSAPSPLPNAGPVNFGLPGLGSTAHLLIGPA.[A]	4346.26419
8933	[S].QPAAAGPAATTVLQGVTLPPSAVAMLNTPDGLVQPATPAAGGEAAPV.[L]	4346.27006
8934	[G].AGPAGLQMAYFLQRAGRDYMVFERAPGPGSFFMRYPRH.[R]	4348.12661
8935	[N].PLEFFQAELQVIDINDHTPVFMDGDMLLKISESSLPGTT.[F]	4348.14035
8936	[P].AASEARPAVCERWVLVMGAGSRQVCGTRSVPGSFLGSAATAGA.[G]	4348.14959
8937	[N].LAETFNTPALKDGSSTFDPLAKPPVSTETKEGLECTQALPS.[G]	4348.15409
8938	[R].PGISVDSARSSSSQTGRMGHGLPRPTLKAGPGGMASRQSQRPG.[A]	4349.1698
8939	[S].LSPGPDAPLAPASSAGPGPGLSVAPGPGVSFSPGPTPTPAPTAGSFAGGA.[G]	4349.17246

8940	[E].ALEACLRGIPLSGSLPPQPPASFWSRSPQPGDPGSQRPELP.[R]	4350.2088
8941	[A].SQIDPQKDVEPRTTYQIENFAQAFGSQFKSGSRVPMTF.[I]	4351.10881
8942	[A].QREQRVDMTVIEPYKKVLSHGGYHGDGLNAVILFASCY.[L]	4351.17506
8943	[V].NNIAGIEEVNMIKDDGTVIHFNNPKVQASLSANTFAITGHA.[E]	4351.17756
8944	[A].SPAASPAAVFPTASPADKDISSVPETTADLEDTIVEGVPPSGSGD.[V]	4353.07801
8945	[Y].PNGAPVVQIYTDSEFSSPEVQGPVISFNVLDDNGNIVGYSQ.[V]	4353.08337
8946	[V].AQDKQNKHTSYISGPWFDMYLTARDSVVLNFNPFLSF.[N]	4353.10735
8947	[V].TQVGPGVAAVPVGEETTATSGFTIEPENKTEWELAYTPAGTF.[P]	4353.10852
8948	[I].FPGTGEKGEKGEKGEPAVVEQGQQFEGPPGAPGPRGVVGPSGPPG.[P]	4353.15345
8949	[S].WRNRNNPSFIMGSITPMDYALAKCYLPREDMILTTY.[C]	4354.09159
8950	[E].TESQAKLVCQHGLCVGSSAITTLGDPAKGVYISKYSDYLH.[A]	4354.14823
8951	[P].TSSKTTPTPGSTTGLPLPSTGPTSSPTAPPASATSTGAPTSTGRPST.[T]	4354.15324
8952	[D].KDAAKADLNYIGLDGNIGCLVNGAGLAMATMDIIKLHGGTPAN.[F]	4354.19922
8953	[V].NLQEMVLHVPTGGGGGSGGGGGGGSYHCLACESALCGEEALSQH.[L]	4354.89686
8954	[F].VANPAHLQMREDMAKYRRMSGVRPQSFRDLETPAYW.[A]	4355.10191
8955	[S].QGSLNSSASLDLGYLAFASSKSESHRKSLGSSEGENESRPGK.[Y]	4355.11344
8956	[A].QVTTGAFHGTPMPTCPGLPPASRSRSPLMVTIVHQMPNHV.[D]	4355.13043
8957	[I].SQEPAMGIPSAVVPGSMAGRMTTTVAPGSIAGGMAPSLPPGSMIR.[G]	4355.13246
8958	[S].RAQLMPQGQMMVNPQSQNLGPSPQRMTPPKQMLPQQGPQ.[M]	4355.14503
8959	[G].QEAGKGRNAHLSSPSSLSLNSSHPGNLSSPEAGIRNPTVCLQ.[T]	4355.15453
8960	[A].VGDWVLSIDGENAGGLTHIEAQNKIRACGERLSLSLSSSAGS.[E]	4355.16845
8961	[Q].ATPSHAPGVPTLASSPALEAEEVWLSTMAPSPSSVEASTVVGTH.[A]	4356.13402
8962	[N].VTAQAMQSNNGRGEDEEEDDDDWDEEVLEETALEGFSTP.[L]	4357.79571
8963	[K].GPREPEPPADLYTKGRYVMVSGDASFIDPGFCVFSAPVRG.[G]	4358.10089
8964	[G].RQRRCGGGGAGSAAGGKMADEEKLPPGWEKRMSRSSGAAGVGAG.[T]	4358.11599
8965	[P].AGRCSFSLRSGIPGTEGWMAPELLQVPPPDSPTSAVDIFSAG.[C]	4358.12202
8966	[P].AVPTEAPGSTEPPARHPDTKGKGGDSATTGHERPESKEPPPKG.[S]	4358.13959
8967	[V].TGERGSCLRALGHGGGSDARSCRRDPLVSEVRQDPGDPLAP.[Q]	4359.11777
8968	[M].QPSGSVPVMVSLQGPASVPPSPDKQRMPMPVNTPLGSASRKM.[M]	4359.20801
8969	[R].AGKSSPGQGLTARREHVYGMFRGGDRSGSLSSTAGGRSGGGHTL.[H]	4360.14603
8970	[P].PAAAPPSAVGSPAAAPRQPGLMAQMATTAAGVAVGSAVGHTLGHALTG.[G]	4360.22888
8971	[L].QPAASTTQAILPHLMTQGSFIAGDSQPSPLALLAATCSKIGPP.[A]	4360.23157
8972	[A].VPSAAGYALMAGAHGLWMLLLGRMLTGFAGGLTAACIPPGPGHV.[L]	4360.2378
8973	[E].LSWTPMGYVVRQTLSTELSPAPKNVTSMINLKMIASPADP.[K]	4360.23896

8974	[Q].QPPAPAPTTTAATQQHSRQAAPQMLQQQPPRLISVQAMQR.[G]	4360.25135
8975	[S].ESKKGSRQAHPGPAALQPKLESQPQPQGRQAAPGPQPSQPPP.[S]	4360.26574
8976	[Y].GAGRDEVRPEEIADQELAEALQKSVEDAEKSGKETTSLGMS.[S]	4361.10491
8977	[L].ASPGSQSKEGSPVSKMSVSRSSSLRSSSSLSSQGSVASSIGSQTS.[F]	4361.11212
8978	[I].SGQDLLSVPMSQAALGEIVPPGEDQVGHPSPTVHQDFVREH.[H]	4361.12552
8979	[Q].SQQLGYQLNPNPTPSPLDASPRRPPGPATSPTSSSISSSISSP.[G]	4361.16441
8980	[R].RSTLAPCAMVTHSKFPAAGMSRPLDTSLRLKTFSSKSEY.[Q]	4361.18391
8981	[K].TSQSEAGKVKNTVSAAAFPQPIASPTTSSTVQPQLSATTLNGST.[N]	4361.21069
8982	[P].TVTMVEGQGEKNVTFWGKPFVQCDVLQGQKMMVEEKR.[P]	4362.10254
8983	[S].GALMTGGALGHRSPPRPPEAGPGTEPTARASAGETSALQPAETPP.[V]	4362.15313
8984	[R].GAPVRAGAAPSELDQMNPGQAQAAGAKRGWPPAAWRDSTFVR.[M]	4362.16973
8985	[I].QLTSSVSSAPSVMETNTSVLGPMGSGLTLATGLNPNLPASQSLF.[P]	4362.18434
8986	[G].AVGPAGPRGPAGPSGPAGKDGRTGQPGAVGPAGIRGSQGSQGPAGPPGPP.[G]	4362.21985
8987	[M].GPSSVTLVMAEPSPGQSGVGLARAHFEKQPPSNLRKSNFFH.[F]	4362.22003
8988	[-].MGPSSVTLVMAEPSPGQSGVGLARAHFEKQPPSNLRKSNFFH.[F]	4362.22003
8989	[G].AKGMPGHNGEAGPRGVPGIPGTRGPIGPPGIPGFPGAKGDAGTPGPPG.[P]	4362.23126
8990	[T].QPSALQAPGGAAPAPSSALPGTAQLPTPGPTPAPAAPSQGSPLASQPP.[T]	4362.23645
8991	[M].RWAWAQALLSSQPHVPGAVGGASALGMWPGGGPRGGGAGRVWVP.[R]	4362.23663
8992	[P].LSTAPPAAAKEVGAAPALGAGQKPQGEATPGGGSGPLGRPGLEREDA.[L]	4362.24366
8993	[L].RMAPAYLPRCLAILSLAENEIRDLNEVSFLASLTELEQ.[L]	4362.24722
8994	[S].SVPSSETVICRPQPAPASPPQKSVLVSPPAVSAAGVPPMPVIC.[Q]	4362.26584
8995	[A].SPHSNRTTPPEAAQNGQSPMAALILVADNAGGSHASKDANQVH.[S]	4363.08685
8996	[S].PTHATSHSHITTSHSHTTVSPIHITTSPTHTTTGPTHTTASP.[T]	4363.10863
8997	[V].STRDQEVAASFLTAGSQGLGPFKDMALAFPEEEWRHVTPA.[Q]	4363.10881
8998	[Q].PGAGQPGMVQPDIGQPDLVQSGAGQAGVIQAGAGQAGVIEPGAGQAGV.[I]	4363.17353
8999	[G].GFRSDTSWVEPRVLDGLEAQSSALRLSAWSHAGPPTLGPQS.[H]	4363.18542
9000	[H].QGSLPLLGQCLTSYWEQKKLMAPGCEPLAVRRMMDVLA.[P]	4363.18919
9001	[G].QELQDFLLSQMSQHQVHAVQQLAKVMGWQVLSFSNHVG.[L]	4364.1703
9002	[P].GVPGSLMVSGLTEAFVMVQSRVEELVERLSWDFRLGPSPG.[A]	4364.20535
9003	[Y].HSGLTVGMISGGGVVCVQEARAGYVGSLGCLHLGSLGNSARGAH.[A]	4365.13975
9004	[G].PFTDGPLDRHFLLRGGGQAATLSRTCLGGGGGFPEEPRDVPG.[Y]	4365.15816
9005	[G].APAAAAAASMSAPAPSHPLSDVATSVIQAEVTVAPGTGVMPPSGLPV.[P]	4365.2105
9006	[Q].GSLEPQQGLRRQVPGNRGLNQGGEGHYAIPGAPARIEAMPW.[A]	4365.21701
9007	[L].AGSLGSCSSLPTIGWARRRRRLELRWVGGSGPDPGGGVRSSG.[W]	4365.26061

9008	[A].DKVQGSHSLSRGTVCRRTGVQSLEPKGQGWDYGIGGGWDH.[L]	4368.10752
9009	[D].PSGPGIGSSGTCEAQVAVVTVTPAPEPAENSQDLGSMSSLGPGISG.[P]	4369.04461
9010	[G].LSSGAELSGQASGSPDISGETSGLFGVSGQPSGFPDISGETSGILE.[I]	4369.04777
9011	[I].PGGPPSSPGSRKLSAAGSSDGVMPVAPTSAVSSSGSPASVMTSIRAP.[S]	4369.13985
9012	[-].MSKSPSAMQQDGLDRSELLPLSPLAPTMEEEPLMIFMSD.[E]	4370.02566
9013	[S].APASPSSASKEVGIGFAQGPGASASTAATPGPAGLPRGYMAPTSPAAS.[E]	4370.13575
9014	[P].GQAGAPGKEGLIGPKGDRGFDGQPGPKGDQGEKGERGPPGVGGFPG.[P]	4370.16608
9015	[P].GPAPADAMRLSLTVSRFMGPASGMNMSGMGGLGSLGDVSKNMAP.[L]	4372.0321
9016	[T].GQPNITPSSSPSPVPAATNQVPTAMSSSSTPQPQGPPPTVSQML.[S]	4372.10715
9017	[P].PPPGMLMPPMPGPGPGPGPGPGPGPGPGHSMRLPVPQGHGQPPPSV.[V]	4372.13987
9018	[S].FSTASAITPSVSRTSFTSVSRSGGGGGGGFGRVSLGGAYGAGGFGSR.[S]	4372.14535
9019	[Q].LHLLLRLSVAFIYTSIFLAILLCHCVDSLPFADVAIME.[G]	4374.34666
9020	[A].GPATVICFLVAGLSCVLSGLCYAEFGARVPGSSSAYLYSYI.[T]	4376.144
9021	[L].AEEKTEAAKEESQQMVLDIEDLDNIQTPESVLLSAVSGED.[T]	4377.06612
9022	[L].QGPAAAVAEPVPGQSDTATGLGPCLPPAAASPSEAGSTGPSRPGSPGP.[P]	4377.10518
9023	[L].PCSSPSILLCLTAHHHCLFLHPQDSPSTPSDHRMLELS.[L]	4378.06241
9024	[P].GAPGMPPGIPPLMPGVPPLMPGMPPVMPGMPPGLHHQRKYTQ.[S]	4379.19686
9025	[I].YGKNAGVQELESRFLVPGDLLILMGNKVQMPCDAILVDGS.[C]	4379.20839
9026	[I].AVNGGGLHASGALNFSHLPDVFALAINEQISNAAHVAVVEQGSP.[H]	4379.21672
9027	[Y].PQGVHPAFLGAQYPYSVTPPSLAATAVSFPVPSMAPITVHPY.[H]	4379.22093
9028	[M].GAQPPVAQVMPGAQPIAWGQPGLFPATQQPWPAVAGQFPPAAF.[M]	4379.22226
9029	[A].PVFLFSQYSGSLSEAAPVNSIVRSVDNSPLVIRATDADSNR.[N]	4379.22662
9030	[R].QREGQFSSLQLVAMQRGVAAAMQYLSSFAFVHRALSAHSV.[L]	4379.22882
9031	[V].GSPAVPAEKTHISPLESTDNRPERKSPGIKNPGDKANVQEQ.[S]	4379.23383
9032	[T].GGAAPVTAAPPAATGAAEARSHPEGSSRKQQRAQSPARLRDNSV.[R]	4379.24237
9033	[G].QLQAHAASVPGPNPRAHGRGQARQGSSAGSKYRPARGRSRSN.[P]	4379.26618
9034	[L].SVSSFAVGGMIASFFGGLLGDKLGRIKALLVANILSLVGALLMG.[F]	4379.45972
9035	[C].MPKQQPSRQPFTVNSMSGFGMNRNQAFGMNNSLSSNIFN.[G]	4380.01652
9036	[P].GAPPSASSAASRAPPESSAHRLSGGPSTSRPSSSTDNDSVGLEEE.[E]	4380.02066
9037	[F].NASYLPVMPDGSVLLVDNVCALEEHNFLFQLRGGEQPPPG.[A]	4380.14275
9038	[V].PGAPGMPPGIPPLMPGVPPLMPGMPPVMPGMPPGLHHQRKYT.[Q]	4380.18088
9039	[L].APTRSQCTGPQALPVRAQGPVSGTMLETLGTNRTDPVCTALA.[R]	4380.1857
9040	[H].PGSLGLTSTNTNFKGPLICNMNYVVKVEVGAPPSAKMLTCN.[P]	4380.18588
9041	[P].LVNIHTGDTFYFPNFRASGAQLPGLPSLSYPRRDNVCSL.[P]	4380.19823

9042	[N].TGESEGKKRTEALYTPAGGEKPGASVASVHSEAGPKGAEKPAAAT.[G]	4380.20661
9043	[A].QDKCVVIGLQSTGEARTREVLGEKEGQLDGFVSAAEGVFLS.[L]	4380.214
9044	[R].YVVAIGSLCALSTSLLGSMFPMPRVIYAMAEDGLLFRDLA.[R]	4380.21506
9045	[L].KSVATYKGVEPSAPLIQWFWEVMESFSNTERSLFLRF.[V]	4380.21618
9046	[Y].AHGYLKGAKGPGITGTKGDPAGAGPETSLEPGVDSVSLQAFSRAQ.[P]	4380.22187
9047	[Y].RHHFARQDLTQSLIMIQPILYSYSFHGPPEPVLLDSSS.[I]	4380.22339
9048	[G].GAVGAGLMGLAGGVVGAGMAAAALAAEAGMVAAGAAVGATGAAVVGGGVGAGLAA.[T]	4380.24723
9049	[R].PAGDPISIPFPPPLHWARAASPHHLCDIMKQALAAQLAPST.[D]	4380.25325
9050	[S].LGPTFTNTGASTLVIGLTSTQAQSVPVINSMGSSLTTLQPVQF.[S]	4380.26431
9051	[K].QPDELSLQVADVVLIYQRVSDGWYEGERLRDGERGWF.[P]	4381.16362
9052	[R].AAQSRKPNPVPGSYPTQSGHPHLTPNHPAAQMQHASGQKLAS.[W]	4381.17554
9053	[T].EKHASQKDYSRGFGGKYGIDKDKVDKSAVGFEYQGKTEK.[H]	4381.18475
9054	[I].IISSYTANLAAFLTVQRMDVPIESVDDLADQTAIEYGTIH.[G]	4381.19081
9055	[V].RVAPDTGVHTVHIQPPPPPPPPPPPADGGPRGPGDPGGPGAGGGPVT.[P]	4381.22247
9056	[H].LVCPEWPLPRGDGAAEKQQSELMERERRASNAGGPPAPPAG.[G]	4382.15169
9057	[E].PGGPTSALGTTPTPASASPEGLKEESGDLAASPASPGSPPNSDLVPL.[D]	4382.15217
9058	[G].SIPQMLNGEVYPPSVEEAPVLMRYPEGIPPQSQMAVGQEV.[F]	4383.13456
9059	[G].EPSLSTTATLTVSVTEESPEALAEFPAGSAPREQNKNLTFY.[L]	4383.15145
9060	[L].RSGSHVLQNQIWAGQRPRPQNESRDHPRCRGQRSGFQ.[V]	4383.17459
9061	[K].AALSAAAAHHHNPWTVSPFSKTPLHPSAAGGPGGPLSVYPGAGAGGG.[G]	4383.18061
9062	[K].KGATFQSFAKFGNFGDDLYSGWLAEALGNTLQVQFWQRS.[S]	4385.14143
9063	[P].KDVFTFSSRPRSAPHGKTQTMSPEACLFTLDLKEDTSVT.[R]	4385.15405
9064	[A].AAAAAAAANASASTSASSTVSGTVPVVPEPEVTSIVATVVDNENTVT.[I]	4385.1842
9065	[R].APTGPSGGYTQKEATPSFPSKSRPQIPPATLVAFSEHGHASPP.[S]	4385.19492
9066	[A].PKEAVWEALSRMGVYVGREHCVFGEPRELLTQVWVQE.[G]	4385.19579
9067	[L].QEPGVFPQEVADRLLQTMAFHGLLNDATVGIFRGNQMRL.[K]	4385.22815
9068	[A].PGAAEVPGQPRLPGGVVFHYRPGSSRYSLTFEEAKQACLR.[T]	4385.23602
9069	[P].APVLGADHPDVAKQLNNLALLCQNQGKFEEVERHYARAL.[S]	4385.25715
9070	[R].EGLDPHSLSAPSGVPEVSLMPKVTSRSGPWAATDGGITVDPRD.[S]	4386.16705
9071	[P].GVEPVASMTSVASHPALGASSSSLPPLGPAAMNMVGSLGVPPSATQA.[T]	4386.17782
9072	[R].PPATNSGVFAATTGPIQAAFDASVSVPSEGLPQGTSSAPQAPAHPT.[G]	4387.1477
9073	[Y].TSSVLEKGICMLLTILEEQAMDSLLLGSDKQNDFMQSIL.[H]	4387.17912
9074	[P].GPPGSTASLSTASLTPSSPRVPNVSAQGPTVQAPMPTPRTVDDAS.[Q]	4387.18343
9075	[V].GLPGSPGAKGEQGPAGHPGEAGLPGPSGNMGPQGPKGIPGNPGLPGPKG.[E]	4387.20002

9076	[G].SPATLSPSAGVPQPVGMEALDQAEGPAASQRAMPPPPPASPPSEP.[A]	4388.11733
9077	[G].VSAAGGGPAGAAGGAAGGGPAAGPADHGLAGRGAAGDGPAAALLQAAGVAADWA.[A]	4388.15149
9078	[Q].GAPQPGLSGLSPAGPELGAFSQSPAPAMGGRAGLHCAQAYPVRTT.[G]	4388.16628
9079	[V].GEYRRLANSEKYAGGGGVGGGADTVPTEPACVPRVGLSTLGSQP.[Q]	4388.16878
9080	[M].DFIQYIASAGDTMVFPSPRPFLASPASPPPSLGRLEAAEEVG.[A]	4388.19075
9081	[H].PSTACAQLQVPALPVNPSSAAMAAPGHPLLLDNSPRNGSVMGPP.[F]	4388.1948
9082	[R].LTPGGMALPGQPGGPFLNTTLAQQQQQQHSGGAGALGGPSGGFFPG.[N]	4389.14693
9083	[F].EAPGGAQVTVAGSSPPAVPSHSMVGITMDVGGSPIVSSAGAYLIHGG.[M]	4389.14896
9084	[K].HQQLQQMRMQQPPAPAPTTTAATQQHSRQAAPQMLQQQP.[P]	4389.15098
9085	[P].SGPPGPKGDDGIPGQPGLSGPPGPKGEPGHPGTDGAAGQRGPPGLKGE.[Q]	4389.16066
9086	[P].KGDRGPQGQPGLPGHPGPMGPPGLPGLDGLKGDKGNPGWPGTPGAP.[G]	4389.19455
9087	[G].MQGPPHPHGIQGGPGSQGIQGPVSQGPLMGLNPRGMQGPPGPRE.[N]	4390.15025
9088	[D].SALHTSVMNPSPQDTYPGPAPPSVLPSRRGGCLDGDMDSKVP.[A]	4391.08531
9089	[C].GPITLPGEKNCSYFRHFNPGESSEIFEVTTQKEYSISAA.[A]	4391.09249
9090	[H].PAVTQSFTHSEISTLMSRGPQDVSRTSPASVEETSSPSSPVT.[L]	4391.09434
9091	[Y].QSFPTHFTATTMMPRLPTISAQGSQPPGNAHFSVYNQLSQ.[A]	4391.0972
9092	[A].RTAPLYMESGTSWGSPARGQGLPEDVTVHTQMAATSVLSQSS.[L]	4391.10307
9093	[L].KPLQDEGQSAVPPLMTSPEAVMAMGQKHSLPADEDSVLEEL.[E]	4391.10913
9094	[R].VAYEVSVQEAAALYGGHTPAGMQTKYMDVGWGLGSVTHELAP.[G]	4391.11112
9095	[N].KDFSFFVPENFPGYGEIGVISVTDADTGQNGWVALSVVNQS.[D]	4391.11427
9096	[M].NNPQVQQLMSGMISGGHNPLGTPGTSPSQNDLASLIQAGQQFA.[Q]	4391.11431
9097	[P].QGPPGKPGPAGMKGEDGLPGSPGEKGEKGETGQPGPPGLDGPTGEKG.[E]	4391.12083
9098	[D].QGLSGFPGSPGEKGEKGSTGIPGMPGSPGPKGSPGSVGYPGSPGLPGE.[K]	4391.12485
9099	[L].RIAPPEAPITGYMFGKGIYFADMSSKSANYCFATRLKDT.[G]	4391.12975
9100	[E].QKTENGKDKEQKQTNTDKEKMKEKGSFSDAGLGDTKMKS.[D]	4391.14533
9101	[P].PAGPTSPSGAHPGEKPLVDLPGEAPTGPTDAAGKNMALTSPREAAG.[D]	4391.15722
9102	[F].VHAISSAGVMYTLTRNCSLGDFDNCGCDDSRNGQLGGQGW.[L]	4391.89211
9103	[L].SARTYSVDGPNASRPQSARPSVSEIPERTMSVSDFSYSRT.[S]	4392.09093
9104	[I].PSDQQVINEMVRELDGHVLKCVKDQNGNHVVQKCIEC.[V]	4392.09517
9105	[T].RAPDGSMVTQRIRMDMHKVDAVFVGTGDLFAAMLLAWTH.[K]	4392.15083
9106	[S].SRVSPDRQRLQAAGGMRCTVMAAAAQISGHPEAGLGVWQGAGS.[W]	4392.16188
9107	[A].AAAPASVLPASQAAPTSTFSLPSAVFSFGSSSLKSSGSVPGEAPPSS.[S]	4392.18816
9108	[Y].GPEIEANVTTGPGEGAPGPPGVPIIVRYSSAIAIHWSSGDPGKGP.[I]	4392.22589
9109	[K].GPPYSPLDASIFPQSESRMKNSRSGVDSGIGESVHVSSGTRT.[V]	4393.11133

9110	[T].GPLGLAGEKGDQGETGKKGPMGPEGEKGEVGPPGPPGPKGDRGEQG.[D]	4393.14771
9111	[S].SHWPQGPAGPPGPPGPMGPPGLPGPMGIPGSPGHMGPPGPTGPKGTSG.[H]	4394.1056
9112	[V].VSQQTPQNHALQPHHHIPMVPAQQPVVPQQPMMPVPGQH.[S]	4394.14919
9113	[S].PASSPTVNQTQQQMGPRPPQNNPLPQGFQQPVSSPGRNPMV.[Q]	4394.15169
9114	[P].SNELPGMSGKGAQLFAKRQSRMEKYVVDSDTVQAHAARAQ.[S]	4394.17282
9115	[V].SASVSPSAVSSANGTVLKSTGSGPVSSGGLMQLPTSFTLMPGGAVAQ.[Q]	4394.1854
9116	[I].YIDPSNLRRSGTISTSAAAAAAALEASNASSYLTSASSLARAYS.[I]	4394.18587
9117	[S].ASQTQSTPTVQAAAQAQVTPQPQTPVQPPSVATPQSSQQQPTP.[V]	4394.18587
9118	[F].ANLNELGRPASGEKSPFYVSFLAGCVAGSAAAVAVNPCDVVKT.[R]	4394.19077
9119	[E].PTGPSNGAPSSRTVPDLAGTTASNNDLASLFECPVCFDYVLP.[P]	4395.05439
9120	[E].KHSDLREVENEITKMLNVCFSTLPGYTRSTAHVSRESS.[V]	4395.14561
9121	[E].SPQSLGATNSSPTPVGRGAQVGPGQGPVPQDGGAAPSEPRGTAALSE.[G]	4395.15597
9122	[L].RSGGAMILTFSVLGARAVISFCTLSAMPGYMVVPPDSTGGAGGH.[G]	4396.1709
9123	[L].KQQQQQQWQQQQQGSAQPTPVPPSPPQPVTMGAVPAPQAPP.[P]	4396.18913
9124	[T].GPSHFQDVRLIEFDISDSGIRCGPAPGPRGVRAARGSHGPGL.[S]	4396.22283
9125	[D].PGERGPPGKEVCAGAPSAFLENVDQREIAETLALRGHLAWP.[W]	4396.22551
9126	[P].PGPSTAALEVMQDLSVNPPPPFIQGPKGDPGRPGKPGPRGPPGE.[P]	4396.25066
9127	[W].PCPRSPSLSSQLQVHPGARILDPFAMRVVRGLEPLLCLL.[L]	4396.35667
9128	[A].GAGPGPGPGAPPGLEAALQKLALRRKKVLSAEEMELFELAQAAG.[G]	4396.38095
9129	[R].RGSGGGSASALGAAGTGVGSSAPSAEDFPPPSLLQPPPPAASSLSGPQP.[P]	4397.16441
9130	[P].VTTLSTGQATSSTGVSTRTSLSPPGPEPSTSSLGTSLGPTTTTTTS.[S]	4397.16895
9131	[R].PAIPPKMSRSSPGGSPVSPSTSPLYDLSEGNSGVPGPQPPSRGPA.[D]	4397.18304
9132	[P].PVSRGKENELQAGPWNTGRPLEYRPRGNTASMTAVPSMLP.[S]	4397.18775
9133	[N].AYRPAASRSRSASGEVLAPWGSPEGVMPIAQAVPQGAETPRPP.[L]	4399.23641
9134	[S].SPGKGSEPRPLAPPGSGHAAIARAPAKSLASHHTSPDQPAPPASAA.[D]	4400.26065
9135	[P].AAAPFTSSSAANGLESSVATDSSKLATITTPMALNTSGIDVKSSGS.[M]	4401.16136
9136	[T].APNKTRAPTLQPQTDEVTDHTVTLNPSTATSSVPPGNATRET.[T]	4401.19169
9137	[K].NRDLQESISSAISALDDPPLAGPKDASTPDGPPLAAEAAVPGPPP.[L]	4402.20488
9138	[Q].PGGTVEKESPDKLSVGDGQVPTDWGPGGTLPHPDTSHPTENLP.[E]	4403.10623
9139	[S].AAGEERPPEADGKKGNSPGSEPPPPKTAWAETSRPPETEPGPP.[A]	4403.11746
9140	[P].AAMFAPQVSARGVPVAEFNMETTPATNRFRYGVPRAGGGWH.[-]	4404.16656
9141	[S].SPGRAPACPPSVPTPHPERGGLAAGASAQAPPLTWPLCLADYL.[V]	4404.2016
9142	[T].QPVPLANKAVPSQSTFPSKTGGMEGGTAVATSSSLTADNDFKPL.[G]	4404.20277
9143	[P].RQPLTYMAQRQPSESGRHLLSEPNTPLSPPGPGDVFFPSL.[D]	4404.21936

9144	[G].GLSSKCAVLVEVLDTNDNAPELIISSLSNHVAENSPETVLAV.[F]	4405.2443
9145	[E].NSQKTSTAAVPMVTTVEPATLQSMATGSGRSSLPHSPTGFVTAA.[K]	4406.16025
9146	[L].SYLVASYIVSTGDETPGPAHTGREAVRPDGGLDIQGALPGHSGT.[Y]	4406.16474
9147	[S].KAGPQGWEERPPEPPPGPPSKSSTQTHGSLEEQLLQELSSL.[I]	4406.1899
9148	[A].KGLASSSGGPSPAGSPSQPMQSTAGFMNALHSMTPSYESPIHST.[T]	4406.99622
9149	[L].APLSRDQEQVPPASMGPEPALAASGLNRAMTSEKQPPQPPHS.[S]	4407.14561
9150	[A].ADLPYGLQSCGIFPALGGKRNEAASPGSGGGSGGLGPGAHGYAPAPI.[D]	4407.15749
9151	[A].LLEESMKEHPVVNVEALAEVSMEAPPKVSVEVLPEVNMEG.[P]	4407.16558
9152	[S].SPGSRKLSAAGSSDGVMPVAPTSAVSSSGSPASVMTSIRAPSTTGSL.[G]	4407.17663
9153	[D].LEPTTPQEYILKGVVNAALGQEMCSRDHMKIAQQFFQL.[V]	4407.19341
9154	[L].SRLPSRGEDHLETSASGVGDLSGLPSGREGLEISASGAGDLSGLI.[S]	4407.20225
9155	[P].LTWSSTASPAPAFGPWTFTNMTSSLEATGSPTAISAMGTTSSLA.[S]	4408.06356
9156	[L].TSHTATAPSATPMFGQVATSTAPSLFGQQTGSNVSTAAAAPQVSSS.[G]	4408.09976
9157	[P].PGKPGPAGMKGEDGLPGSPGEKGEKGETGQPGPPGLDGPTGEKGEPG.[D]	4408.09976
9158	[T].STPSTVGQFSKPFSFSSSGTGFNFGIVTPTPSSNFPATQGSAPP.[T]	4408.10444
9159	[G].PSGPGGTMPIRIESSSSHVAEGQTLDLNCVVPGQAHAQVTWH.[R]	4408.11973
9160	[L].GREVLASSTCGRPATRACDASDPRRAHPAALLTSAGGTASPVC.[W]	4408.14154
9161	[P].KPDASPSVSSAPATRDAPEGKQEVEPQQAAGTMSPKTGKKEAGA.[T]	4408.16851
9162	[V].GDVVTGGYAMILEKETMLNKKTKQIQDMAEEKGTQAGEIH.[D]	4408.1833
9163	[P].ADKASASGSGAPVGGSISSGSSASSVTVTRSYRSVGGSGGGSFGDSLVT.[R]	4409.10875
9164	[A].GPSASASAGPSASASAGPPASARPGTSAAARAATSASARADMSATARPGP.[S]	4409.12469
9165	[G].SSVTLSTEAFLYVGTMLDGGDLSRFMFRFAGNYDLVYLH.[C]	4409.12571
9166	[S].KGFPGSPGADLHGDLGFPGPAGDRGDPGEANTLPGPTGAPGQKGER.[G]	4409.12936
9167	[P].GRDGRDGIKGDPGPPGPMGPPGGMPGLPGRDGMTGAPGLTGERGEK.[G]	4409.12958
9168	[K].MVQRSGPSGPPGPKGDDGIPGQPGLSGPPGPKGEPGHPGTDGAAGQR.[G]	4409.14397
9169	[Q].PASASSPQSARDTGKPKPVSDNSSRSVSEDEKPKGVSSDSPRP.[V]	4409.15636
9170	[S].RGVPGGSGDQANPRGPSAAGESGGAAGAIPQILGAPHAPGPGGDAAPGAGV.[L]	4409.17296
9171	[I].PCTLEPGLDPRIHYLNGLYGDRNTPWAGGRGCAWVPSWA.[V]	4410.10837
9172	[F].GQSPFSQPPAAPHQNTFPPRSSGCFPNIAAKVNGNPHSAPVSG.[P]	4410.12211
9173	[G].AVPTPEQSATLAGAVSTPEEPATPAGAVSTPEEPATPAVSTPEEPA.[T]	4410.13585
9174	[P].QRALGDQGWGPLGGVGVTGNPGTPHPPMADSGSWGAPMLELSLAA.[R]	4410.1394
9175	[Q].QGSAQPTPVPPSPPQPVTMGAVPAPQAPPPPPKALYPGALGRPPP.[M]	4410.34311
9176	[I].PGADAATLQGSRASRPGGSHGDSGSPPPALSSSIRSVMQKYLED.[R]	4411.13313
9177	[T].QPMTAQAASYRAQPSVSLGAPYRGQLASPSSQSAAASSLGPYGGA.[Q]	4411.13715

9178	[P].PAAATMTTTIRHFSSGSIKGASGLAGGSSRSCRVSGSLGGGSCRL.[G]	4411.17759
9179	[Q].QRKQVSDCGAIKNKSSGGSNKKECLSYLSTHQKMKSDGLG.[A]	4412.18676
9180	[Q].ADSERLMQLWVSAVQSSIATAFSQARLDDSPRGPGQGSGHLA.[I]	4413.16403
9181	[E].VEPGTSRGSFLITESAPTYAHLSISQQSPVRWGQESWGAPV.[R]	4413.18984
9182	[L].PGQPGLKGDQGVPGERGPAGPPGPQGPPGEQGPEGIGKPGAPGTPGQP.[G]	4413.19705
9183	[T].GETPIAVMGEFGDLNAVSPGNLDKDEGSEVESEMDEELDDSS.[E]	4413.88683
9184	[P].QPSVPEQTVDNGLEQAGTSKRGSILQLCEEIAGEIESDTVE.[V]	4414.12022
9185	[L].PVNPSSAAMAAPGHPLLLDNSPRNGSVMGPPFAEPPTAEMGVKG.[S]	4414.12645
9186	[G].YMLYRTYLTYTVSEPTQLWVPNNGVHDRAYVMVDGVF.[Q]	4414.13113
9187	[S].DVKAETQEVYHEPLSSITVSTGSFLSYENIDLSLTEPESV.[P]	4414.13479
9188	[H].MSLQQGKSSSTGNLLDKEDLALPPPDYGTSSRAFPTQTAGTF.[K]	4414.15073
9189	[Q].AATSHHLGQNFSKMFEIVFEDPKTAGEKQFAYQNSWGLT.[T]	4415.11898
9190	[E].KQMAMSRALDFIRMKGYPSSTNVETVNDGAESAMFKQLF.[Q]	4415.12909
9191	[A].KSHDLMSGFWNACYDMLMSSGQRRQRERAHSRRAFQ.[E]	4416.06146
9192	[G].PPPMGMPPRGPPFGSPMGHPGPMPPHGMRGPPPLMPPHGYTGP.[P]	4416.07306
9193	[P].PAGDGKKPSIAAVVGSMDAHPNRYCATVRVQQHRQEIIQD.[L]	4416.20479
9194	[L].SGLPSGQPEISGEASGILSGLGPPFGITDLSGEAPGIPDLSGQPSGL.[P]	4416.20929
9195	[H].TSEDGKVQVTRPDQARMDIRLAKTLVLILVVLIICWGPL.[L]	4417.48258
9196	[R].KSTPSTGSPSQSSRSGSPSFRPTVGFTTLATAYPSPPPGPTGPAD.[S]	4418.15351
9197	[I].SPDRRGYMGLDDILLLSYPCAKAPHFSRLGDVEVNAGQNA.[S]	4419.16086
9198	[E].KSGAPPTPGRTSPAVMQPPPGMSLPPADIGPPPYEPPGHPTPQP.[G]	4419.19004
9199	[G].RGSGDSGKEAMNFEFEREIGFINSQPSLAECLTSFPAVLE.[T]	4420.08603
9200	[D].LDAPYLDLAPYMPDYYKPQYLLDFEDRLPSSVHGSDSL.[S]	4420.1006
9201	[G].QPGFPGPKGEMGVMGTPGQPGSPGPAGVPGLPGAKGDHGFPGSSGPRG.[D]	4420.13498
9202	[G].GLGAGFGGGFGGGLGGGFGGGFGGGDGLLAGSEKVTMQNLNDRLASYL.[D]	4420.14151
9203	[S].QPGSPSPKTPTPSTPGDTQPNTPAPAPPAEDGIKVEENNLKEE.[E]	4420.14267
9204	[S].GPSAPPALFELCGRAVSAHMGVLESGVWGKSRDEGLPLATNGGG.[P]	4421.17651
9205	[A].GPATSPARPLPQPMASLTVVCVPLPPQDIINSMSNSPATSKPP.[V]	4421.26657
9206	[R].SGPPGPPGGPGMPPGGRGRGRGQGNWGPPGGEMTFSIPTHKCGLV.[I]	4422.11895
9207	[L].QPHLPCSPQYLTHPAHPAHPMPHMPRPAVFPVASSAYPQG.[V]	4422.127
9208	[C].MLLGCLNSGTSFVSGFAIFSILGFMAQEQGVDIADVAESGPGL.[A]	4422.13422
9209	[S].PAMLASVESGGPPPPTASQSASVSIPGSLPSSTPYTMPPEPTLSQ.[N]	4422.13672
9210	[V].KDTLWHSKQNPQVDPVNFPSQRFPRSESTEGHSYAKHS.[A]	4422.13987
9211	[E].RGDRAEGAENKTGDKAGSHRGLGSSSGDGSRSPSKPAGQALEEGV.[R]	4423.13294

9212	[N].GNHNPPATDVNQNLSAAVTPQSLPLSSVQQNSSEAQVPPNDTV.[P]	4423.13965
9213	[D].KGDQGLSGFPGSPGEKGEKGSTGIPGMPGSPGPKGSPGSVGYPGSPGL.[P]	4424.14632
9214	[S].QPAVSIEGQVSNPPSTSSTEVNSQTIPEKQPSQEVKMEPKM.[E]	4424.15958
9215	[R].RSEMRLKLPDTSLPEVAPPCGPSTSLSSSSPEAGPFSPPKVD.[V]	4424.17484
9216	[T].PEKTDLSNGEHAGSGSAAIAENGHAPRERGPSRSGTEDGAQEVV.[K]	4428.07951
9217	[S].IGPQEAQGNSKTHHDPLQPVGGERPPEMGPSGEARGAPALPCT.[V]	4428.12079
9218	[Y].SPGQPQGLLGPQAYGQGFGGGQAQDLSKGPSYSGGPQQPPSGPPPP.[G]	4428.12796
9219	[R].QQAMAQSLSQCSLAAGPPPNSMGLSNSLAGSNGAGLQSHLYQPA.[F]	4429.06056
9220	[K].DNINRRSWKSFMPPNFPEFAERMEASLSEVSEAGVSNPS.[L]	4429.06121
9221	[S].QWAASAPSLWSSSPMATAAAAASATPSAQQQYGFQYPLAMAAKG.[P]	4429.10162
9222	[G].MVQEGALTSPIFSFYLSSQQGSQDGGAVIFGGVDSSLYTGQIS.[W]	4429.11804
9223	[H].NGLEKQNNKDCSSPHPAPQQAAGIELERGAQDISEELNRQ.[L]	4429.11854
9224	[L].NPDKIKEYTCRYKLDDSQVYNKEPASVTNEKITCQGH.[I]	4429.11872
9225	[Q].DYFSLVRVHCFQGNIQRAAEIASETGNWAASYHLARQY.[E]	4429.13195
9226	[P].TLQEPPPGPSGSPAIHTLEAQSLRCLQPFSTQDMRGQPFH.[T]	4429.14521
9227	[G].KPGPQGYPGIGKPGMPGMPGKPGAMGMPGAKGEIGPKGEIGPMGIP.[G]	4429.19976
9228	[F].QTVVLDPEGDAQIDPNWVVLNQGMEIVQTMNSDPGLAVGYT.[A]	4430.11666
9229	[F].AQMLVQDEEPLADITQIVDVFMEYNLIQQCTAFLLDAL.[K]	4430.1492
9230	[V].QRSGPSGPPGPKGDDGIPGQPGLSGPPGPKGEPGHPGTDGAAGQRGPP.[G]	4430.16206
9231	[E].AGLRGPEEACGPRRPARTPGEGPGEQLPCRQAHPGPTGRTQA.[P]	4430.18137
9232	[P].GEGALLSNGGSGGTSRKRALDEGSNGHAKFRLKKRRRAPGPAL.[P]	4430.41005
9233	[S].AGMYMQSGSDFNCGVMRGCGLAPSLSKRDEGSSPNLALNTY.[P]	4430.95669
9234	[S].LDKETFAYDLSAVVMHHGKGFGSGHYTAYCYNTEGGFWV.[H]	4430.991
9235	[A].PPAAAQAQVPPGSAGPLASNPGSAAPSAGSAFNPTSNGSSLNPAASSSAS.[G]	4431.10835
9236	[G].QTEAAKTAPAPAGGSASGTSTPLIPSPKPEPEKTSPKPEYPTEK.[P]	4431.25658
9237	[N].FLDGLANSFSGSALFYLGLTMVGKIKKLKKSAFVVLILLIT.[A]	4432.56958
9238	[L].GVEEPLSSMTEDTSSPLSTGYNTRSSSEEVVTEPGASLRGSGE.[L]	4433.00565
9239	[A].QFPDTLPGPGGLAEVAGTCVPHAHVSPGPSGAPRMHCSPDGEW.[L]	4433.02847
9240	[A].GPPPNSMGLSNSLAGSNGAGLQSHLYQPAFPGMVPASLPGPSNVSG.[S]	4433.12889
9241	[E].PGPAKMAVTSSSSSIPSAEKVPTTKSTLWQEEMRAKDQPDGS.[S]	4433.15992
9242	[Q].PSPGTTLGPPAASTPAGPPSGGASPTPPAASPSGGSATRPSSGPTSEAPR.[P]	4433.16039
9243	[T].SAQVTAQLAGMQISGAAAPAPAPSGLGYGPPTSLASASGSFPNSGLYG.[S]	4433.1718
9244	[A].AAAAANLDSAQSPGPSWPAAYGAPLREDWNGYAPGGAAATANAVAH.[G]	4434.09225
9245	[P].APNTSVWSSVPMMSPPLASPSRAASQATTPPASSLCPPGAAGTPAG.[S]	4434.11628

9246	[P].GASAGGIPSSIFGMAGQVPTLQSATTGGGGSPGLAFGAFNPFTTPAAH.[A]	4434.14592
9247	[F].LGPQGIRGYPGMAGPKGETGPQGYKGMVGSIGAAGSPGEEGPRGPPG.[R]	4434.17176
9248	[L].AGRSMVWWPVGASLFASNIGSGHFVGLAGTGAASGLAVAGFEWNA.[L]	4434.18767
9249	[L].DPLSRHHAFCFRGVSVAPSPGEEEGSTPTAGPDVEDWIVTQ.[V]	4435.0684
9250	[V].PGLSSSLSKWADGPCGNSGFPGSNIQGFVCKFSFKGMCRPL.[T]	4435.0879
9251	[M].GLERMGANSLERMGPAMGPALGAGIERMGLAMGGGGGASFDRAIE.[M]	4435.11961
9252	[V].QGVQNSIHASPSLNGRCTEEVKSVDENLEQTGKTVVCVHQ.[D]	4435.1365
9253	[P].SDGNKRDQATSMSQLHLICGGPLEPAPSGDPEAPPPGPLHSAF.[S]	4436.10341
9254	[G].ELSSTTGPQGQGEGRGSSLSIHSLPSGPSSPFPTEEQPVASWGL.[S]	4436.12769
9255	[R].GGGERYTRGWKNYSGKMASPKSMPKDAQMMAQILKDMGIT.[E]	4436.14403
9256	[G].SGPGSSAFGSIPPAGQAPFQAQPAPPASRMLTGSHSFAASGMAGVPV.[V]	4436.15505
9257	[H].IATGHGLAPQEMADAHGLLSAEADREDLLSLLQQDEGPPSLPG.[P]	4436.16745
9258	[I].SGPPGGSPGRSSPAGGSPGKPGSTPHVSGLGSPGRYSPANGGHLRRM.[A]	4436.17733
9259	[L].QGPASVPPSPDKQRMPMPVNTPLGSASRKMMYQENPQNPAS.[S]	4437.12065
9260	[G].GPSGKPQCLIPNVVSCLCVLWVQEVGENFMTQRNFIPM.[K]	4437.13207
9261	[T].IQGLEGSADFLGPQGIRGYPGMAGPKGETGPQGYKGMVGSIGAAGS.[P]	4437.16019
9262	[Q].GPPQAVGDRQWLIQGSEPHPALGASTFWGRWAPPAMTANPGQ.[A]	4437.17342
9263	[L].VGSVLYRDSELGGEGRSPGPAGSSPPALPTDPHSHPRSLLQVC.[S]	4437.20042
9264	[L].LEGPTAQEGADLRMSQDSNNSLGQENHPIKGNKTAVGSNFLL.[K]	4438.16918
9265	[A].QMPAFSLPYAQARAHHTPQSLGLQAMPAAGGVLYQPSGPASFA.[G]	4438.18595
9266	[G].AGAVGAEEAEQAAEEMVVDIMQFLGIVDQKNHTSGGQRKGDH.[R]	4439.09905
9267	[I].KTEFSPAAFEQEQLGSPQVRAGSAGQTFMGPSSGPVTTDSPSLG.[G]	4439.1096
9268	[L].GAGQPLFLHPGQFAMGPGAFSAMGMGHLLASVAGSGGGGGGGGPGTATGL.[D]	4439.1118
9269	[L].GINPFADGMGAFKLNPSSHELASAGQTAFTSQAPGYAAAAALGHH.[H]	4439.12619
9270	[Y].APGGAAATANAVAHGLNGGSPAAAMGYSGPADYHPHHHPHHHPHH.[P]	4440.02619
9271	[P].GAPAVCEPLAVPAASPMAAAAEGPQQSAEGSASGGGMQAAAPPSSQPH.[P]	4440.02892
9272	[D].LMLKNSPNSGPSSALATLTVEQLSSRVSFTSLSDDTSIAGSAEA.[S]	4440.20864
9273	[Q].KQLSETANGYPRSVCVNTRQNGFSGGVGALGIMNNRLAETSA.[S]	4441.17355
9274	[T].AATPMPTPKSSPHLDAAPGPLDSRRGSSSSMDPQLGDQKSLTH.[D]	4442.14633
9275	[P].QVGASHLMGYPTAYPAAAPAYNPSLYPTNSPSYAPATLLMKQ.[A]	4442.14717
9276	[L].LDAEKCKYGMEIPTNIPGLGAAGPTGMFFGSAPSPMGGISPAMT.[P]	4443.08378
9277	[R].AESLSSMSSRLHAGSKDSTMPRTGRSPGRSAGASPTNPGPTQSAG.[A]	4443.11241
9278	[T].KGDPAGAGPETSLEPGVDSVSLQAFSRAQPGATPGVYQQSAAEASG.[S]	4443.1335
9279	[S].EVASLAMDSLCRGVFAGNSRELSIRSCFPSLFQAEQTHR.[S]	4443.13908

9280	[W].RSLCTSTVAQASSRTQGEDVRVEGAFPVTMLPGDGVGPELMH.[A]	4443.14898
9281	[T].APVACGAVMVPSAMLMGQVVTAYPTFAPQHPQSQTLSVTQQQ.[S]	4443.16039
9282	[G].FWEDVKLDGAVPAPDPRSLQQEGAVWGTSGRERGPAPADPSP.[P]	4443.17525
9283	[T].PSGSSSSSTPGAGGLGSPRPIKTADKLRPAEEGSPGAPGREPSPTTG.[S]	4443.21349
9284	[P].PYPGGPTAPLLEEKSGAPPTPGRTSPAVMQPPPGMSLPPADIGPP.[P]	4443.23632
9285	[P].AGPRGPAGPSGPAGKDGRTGQPGAVGPAGIRGSQGSQGPAGPPGPPGPPGP.[P]	4443.24131
9286	[S].SSRLMNKSGSEWSDSSPTEGPQGTFVPDILHGNFQEGGHLAS.[A]	4444.05348
9287	[E].NALQAAGSRCLSLASSEGSDARLSERAHAVGSVGISYRDGPSPS.[G]	4444.16582
9288	[P].GTDRSVPHTNGLLSPQQAEDPGAPSPQRWFVTPANNRLDFA.[A]	4444.18173
9289	[D].QGVPGERGPAGPPGPQGPPGEQGPEGIGKPGAPGTPGQPGIPGMKGHS.[G]	4444.1851
9290	[P].GPPGPPGPPGVPGSDGIDGDKGPPGKAGPPGPKGEPGKAGTDGPDGKPGI.[D]	4444.21678
9291	[S].PGDPTPGSRTEEETMTSASLITMFWSLSVSSFAVGGMIASFF.[G]	4445.0662
9292	[A].SPAAASAVPGSGAAAGALASGGSKEEFVATFKGNEFFCYDLSHNP.[I]	4445.0779
9293	[A].STAQQTVSASAPFEGLQDGGSMDGRHSLSVHSFQTTGLHNSKA.[K]	4445.08109
9294	[P].GASASTAATPGPAGLPRGYMAPTSPAASERSPSPSFAGHGYGQSPAT.[A]	4445.08511
9295	[R].HTQPATPTPMQNRTSIVQAAAGGGHGGGGGGDGKTPVCHQCHRV.[I]	4445.09051
9296	[T].APATPAMTSPRGSGSSTSLSTVGSEGDPAPGPTPACSASRPEPLPGP.[P]	4445.09838
9297	[E].STVVPHGDAPGLWGGPEQIPTPPTSPSSGSRSGTYTKVSEPQQA.[L]	4445.16441
9298	[S].GVSAAGGGPAGAAGGAAGGGPAAGPADHGLAGRGAAGDGPAAALLQAAGVAADWA.[A]	4445.17296
9299	[K].RQEPEQAEIAKDTQSVAPDIPLHKDGEAKTDAAGVGSPHPPGT.[S]	4445.19677
9300	[S].RTSKLETSGRYPDASATRAGVVSPEAPLSPTIEEKVMLCIQ.[E]	4445.28031
9301	[S].TGLGSPEAPHPVPGGGEGPPKTGTAPSPGPPCPPVDGTSEGKGARHP.[K]	4446.15313
9302	[G].APGDGPRERTATTVTDSRGAGGGGSGALPAGTANSGTARHWPPFQV.[L]	4446.16821
9303	[P].QMPLQSPTSIASYSQGTGSVDGGAVATAGPGRESTEGPPPLYNTN.[H]	4448.09468
9304	[S].VPDSSKPGVCGGRDVASGALDFTFGRTLENPAGMYSPAHADGLA.[S]	4448.10341
9305	[S].AMGMGHLLASVAGSGGGGGGGGPGTATGLDAGGLGPAGNAASTAGPFPFHLS.[Q]	4448.11464
9306	[L].QGPPGSPGQAGAVGIPGERGPPGPPGPPGPPGPPAPVGPPYTRISQHG.[D]	4448.26467
9307	[E].PTAPSGGLVEKPGAGSEKMGLGPVKLDGRGTQALDAGRISTSSAPN.[R]	4448.28381
9308	[T].SATHGGGPPSGTRGPGASVHDRNANSYVMVGTFNLPSDGSAVDVH.[I]	4449.06611
9309	[A].GPGAVMLQPVTHDNEWEMLSPQNIIPETELEEETEFLEP.[G]	4449.07887
9310	[S].KMIQMAGEIADGMAYLNANKFVHRDLAARNCMVAEDFTV.[K]	4449.09166
9311	[T].AAVPMVTTVEPATLQSMATGSGRSSLPHSPTGFVTAAKPPAKGRA.[A]	4449.30171
9312	[P].KEEDGKKGNKPENQEKGTRAASKDPHLPWGPQGTRSQDHI.[Q]	4451.21991
9313	[F].QPSQGYRPPPFSEKFFLVVIEKDSNNCSILHMWHLHL.[K]	4451.22161

9314	[P].GPSLGAVLCPMPTLPHTGEAEAGVCRGSHWCPARTRAVTSHA.[A]	4452.13289
9315	[P].QPSPHHVSPQTGSPHPGLAVTMASSIDQGHLGNPEQSAMLPQL.[N]	4452.14594
9316	[V].AMVTEEDGAPQVALITQDGAQQVSLSPEDLQALGSAISMVTQH.[G]	4452.1545
9317	[A].GGPDLGVGRNSGSLWPGDQAPEDRRLAPNQRYNQLDFLSQA.[Q]	4452.18279
9318	[L].PGPPGPQGPPGYGKMGATGPMGQQGIPGIPGPPGPMGPPGKTGHCNP.[S]	4453.1097
9319	[S].QGPLMGLNPRGMQGPPGPRENQGPAPQGMMLGHPPQEMRGPH.[P]	4453.11038
9320	[L].NAAEQRGAREAAGSASRSGPGGSGSSGRGGAGVPGPGSGGPGGSAGRMSLT.[P]	4453.11183
9321	[P].PRSAGDSRVASSSKGADSASVTMVVTPSVPGGGMTTMPVSTLSSNP.[M]	4453.12797
9322	[P].VRSMGPLSMEQLSVNQGNGQSAGYILYETVVTGGGVLNSDGHV.[K]	4453.13985
9323	[P].GSPGAKGEQGPAGHPGEAGLPGPSGNMGPQGPKGIPGNPGLPGPKGEMG.[P]	4453.14119
9324	[V].PGIERMGPGIDRIGGAGMERMGAGLGHGMDRVGSEIERMGLVM.[D]	4453.16003
9325	[H].LGPQGPPGTPGMQGPPGPRGMQGPPHPHGIQGGPGSQGIQGPVSQGP.[L]	4454.16293
9326	[G].RGEVWGAGYRSHREPGPGAKEEAAGVSGPAGGRGGGYGSQATLEK.[C]	4454.17329
9327	[G].QGHPGAQPPPRSVPPQASSFTPSVSGGPRMPSMTGPLLPGQSFGG.[P]	4454.17685
9328	[G].DKGSQGLPGLTGQSGLPGLPGQQGTPGQPGFPGPKGEMGVMGTPGQP.[G]	4454.18674
9329	[Q].TVTTSDLVGSVLYRDSELGGEGRSPGPAGSSPPALPTDPHSHPR.[S]	4454.19711
9330	[V].KASGALMTGGALGHRSPPRPPEAGPGTEPTARASAGETSALQPAET.[P]	4454.21171
9331	[T].TSEVPRPPEPSQGSSTTSSGGDAGLQPSPGTTLGPPAASTPAGPPSGGA.[S]	4455.11825
9332	[I].AKSVNGSTQAPTSGKYCRLCDIQFNNLSNFITHKKFYC.[S]	4455.1431
9333	[I].FEFSQSPKIWVSSLLEVGMAETVSCEVARVFPAEEVMIH.[M]	4455.17094
9334	[C].QPSAASMAAVAQRSMPLQTGAAQICARPDPFQQALIVCPPGF.[Q]	4455.18286
9335	[G].HKKSDFSASHPICVSRVGMLSQEDIPILKNEGSSVLAETGD.[V]	4455.19189
9336	[S].SSSLIGGSGAGWEGTALLHHGSYIKLGCLQFVFSITEFATKQ.[P]	4455.24418
9337	[D].FYIGLGLAMSSSIFIGGSFILKKKGLLRLARKGSMRAGQGGH.[A]	4455.46318
9338	[I].NMKPPAMSQYQTPLFVWSVLVTAVLLLLSLPVLAAGITMLL.[T]	4455.48718
9339	[C].PTSQGLTPPNVAQSSMDSCLEDQSSLLHSHLSTLASAQSMLN.[K]	4456.07012
9340	[S].LQGMDMASLPPRKRPWHDGPGTSEHREMDAPGGPSEDRGGK.[G]	4456.07279
9341	[D].APSPTGGVGSFDIAGLLNNPSFMSMASNLMNNPQVQQLMSGMI.[S]	4456.07501
9342	[P].GPPGVPGSDGIDGDKGPPGKAGPPGPKGEPGKAGTDGPDGKPGIDGLTGA.[K]	4456.20152
9343	[R].SIATGEITEADVSSRKGDEIPLTAVKTEASPESMLSPSHVVSN.[P]	4456.20356
9344	[H].PGPGPGPTTGPGSHFNGLNQTVLNRADALAKDPKMLRSQSQLD.[L]	4456.24262
9345	[D].QRLGEEGAIDETDQRSTILPTAEAESTEASTKEGEVKENHT.[V]	4457.11864
9346	[L].SLTDLQTSTSSGLSTTAPPPPWPVGSMIETSGGPDLSDSPSSGGVV.[A]	4457.11882
9347	[R].PSGAATTTAAAAASAPAPGPASSPEASPAPGFPFPPPWMGMPLPPPF.[A]	4457.12571

9348	[V].SSPVHSEKQTPLGAAWEVADIQPEPVEAQPRVMSQEEAESL.[H]	4457.15655
9349	[F].GWEPFIRLFSEYRNQTNLPTDNVDKMNLWVKMFSHQ.[V]	4457.15941
9350	[A].PGTPGTPASLSANSSLSSSGELVEPSVDQTPQASPGLAPNTRGSPGP.[P]	4457.17028
9351	[S].LSPAPSMDILSTENFKPATSQSPQVTSPPTQTPHSAPDPTVTP.[V]	4457.1817
9352	[N].MPNAAAVAMAATLTQQQQPATGPQPSLGVSFGAPFGSGIGTGLQSSG.[L]	4457.18641
9353	[G].PAGPSGPAGKDGRTGQPGAVGPAGIRGSQGSQGPAGPPGPPGPPGPPGPSGG.[G]	4457.20934
9354	[T].QFPSLDIEVDGGVGPDTIHKCAEAGANMIVSGSAIMRSEDPR.[S]	4459.09627
9355	[M].GPPGPQGQPGLPGTPGHAVEGPKGDRGPQGQPGLPGHPGPMGPPGLPG.[L]	4459.21126
9356	[M].AAQQLGGTLLSMDSTAQTGSWTLATAGLWQASWWLLTARGPVG.[E]	4459.25033
9357	[-].MAAQQLGGTLLSMDSTAQTGSWTLATAGLWQASWWLLTARGPVG.[E]	4459.25033
9358	[A].TTAGPAPGGPAQPPPPQASASDLQFSQLLGNLLGPAGPGTGGPGVASPT.[I]	4459.25283
9359	[T].TKSDQIEPSPLPSSLSDTNKDSTGSLPGPGSTHGTSLKEKHKI.[L]	4459.2587
9360	[A].GIGGEPAAAGAGCSPRPKYQAVLPIQTGSLVAAAKEPTPWAGDKGG.[A]	4459.28269
9361	[G].YPGPRGVKGEDGFPGFKGDAGLKGDRGQPGPPGPRGEDGPEGLKG.[Q]	4460.21303
9362	[Q].PGGPFLNTTLAQQQQQQHSGGAGALGGPSGGFFPGNLALRGLGPDS.[R]	4460.21303
9363	[S].GKEREGEPAALRAGEHQPGPADDLAKRSDKEAAPVASGPGRAPN.[P]	4460.24137
9364	[S].VQFLNQYGFDYNKFLKNGIPYMNEEQEKKIKHNILTG.[N]	4461.27
9365	[D].AGGLGPAGNAASTAGPFPFHLSQHMLASQGIPMPTFGGLFPYPY.[T]	4462.14236
9366	[P].GPQGHLGPQGPPGTPGMQGPPGPRGMQGPPHPHGIQGGPGSQGIQGP.[V]	4462.14286
9367	[I].QGLEGSADFLGPQGIRGYPGMAGPKGETGPQGYKGMVGSIGAAGSPG.[E]	4462.15544
9368	[P].PSSPGSRKLSAAGSSDGVMPVAPTSAVSSSGSPASVMTSIRAPSTTGS.[L]	4462.18244
9369	[M].QGPPGPRGMQGPPHPHGIQGGPGSQGIQGPVSQGPLMGLNPRGMQ.[G]	4462.18262
9370	[P].QGLLPPFPPGMFPLWPPMGPFPPVPPPPGSGEAAAPPSTSAAALS.[R]	4462.22905
9371	[E].GPPKTDLMAFSPSLPPFPAWETGSGQVRYSVMEEAKHGTFV.[G]	4465.16315
9372	[I].PTVNASVGTGACAGPAALAHMQLRHVDPKTFKCRAIELSWF.[Q]	4465.24784
9373	[L].PMQNLQPTGQLEYKEQSLLQPPTLQLLNGMGPLGRRASDGG.[A]	4465.26024
9374	[E].NAPGGKPGINQTYRSPLGSTTSAPAPSAPPAPPAFHGMLERAPAE.[P]	4466.23099
9375	[S].QRSHDLKFSANMDRERDSKKSLATLKSEDLGKSSRSKTE.[R]	4466.28046
9376	[G].NLGAGNGNLQGPRHMQKGRVETSRVVHIMDFQRGKNLRY.[Q]	4466.29053
9377	[P].QGPAGPPGPPGPMGPPGLPGPMGIPGSPGHMGPPGPTGPKGTSGHPGEK.[G]	4467.1431
9378	[P].SGPGGTMPIRIESSSSHVAEGQTLDLNCVVPGQAHAQVTWHR.[R]	4467.16807
9379	[Q].ISDFYLSVVTRDSGVPQMSSTGTVHITVIDQNDNPSQSRTV.[E]	4467.17326
9380	[D].NIAHLKDPLDDGPPEEAARALSGSATLDSMEDLKAQLQRDY.[T]	4467.17326
9381	[V].TPPPGSAPGPGPLSGSQGPGQQCLGQAGLPGSVPASTHSLTHSLTHS.[F]	4467.1746

9382	[P].ATSQSPQVTSPPTQTPHSAPDPTVTPVGSSGDHLTPMAHPLDQP.[P]	4469.1314
9383	[A].AVGGNAFGGLGNPSVTPNSVFGHKDGPSVQSFSNPHEPWNRLH.[R]	4469.15585
9384	[I].DVHVHLREPGGTHKEDFASGTAAALAGGVTMVCAMPNTRPPIT.[D]	4469.19112
9385	[S].SPSPSAVSTSHSIPTVSSAPHSPSPSAPLTVSQTASLSTSPPSPATS.[T]	4469.19544
9386	[R].MGLALPGMGGPGPVGTPDIPLGTAPSMPGHNPMRPPAFLQQGMMG.[P]	4470.13578
9387	[R].QLQACSLVPESPGDLTRSPEMDKLKSVAKCYAYIETSSNP.[A]	4470.16256
9388	[Q].PYGPPPTSAQVTAQLAGMQISGAAAPAPAPSGLGYGPPTSLASASGSF.[P]	4470.1922
9389	[M].SNPVTVAAAMSMRSPVNVSSAVNITSPMNIGHPVTITSPLSMTS.[P]	4470.21355
9390	[R].KLEGSHWRCRGPFSYCFLNRGQDEDDDEDEEEGEATH.[Q]	4470.86469
9391	[G].SASEPGPQRLDVEPEVPASSSTSITMATRKELEEMIENLEP.[H]	4471.14908
9392	[S].PSSAMKEELTGTLQSAGTGSEEGARGLGGGIKSSGGGQELAPEDGPL.[V]	4471.15292
9393	[I].NGSAHPHPLHHHHPIHGHHLHHGPHHPSHAGVASTSIPGGPPS.[S]	4471.16026
9394	[I].FEFSQSPKIWVSSLLEVGMAETVSCEVARVFPAEEVMIH.[M]	4471.16586
9395	[A].DGSKTSRASVDTTPSVIQHRAMMRFSELEMKEREGGHPPT.[K]	4472.15037
9396	[A].GGLGAGYGGAGGGGFGGLGIGFGGGSGGGSLGILSGIDGGLISGSEKETMKN.[L]	4472.17868
9397	[P].GPRGMQGPPHPHGIQGGPGSQGIQGPVSQGPLMGLNPRGMQGPPGP.[R]	4472.20335
9398	[C].NTLKGTSDTQELQSPEGPVKESMDLKSESCNQGGEGPPQDKG.[P]	4476.04132
9399	[A].QPLQLQAWMDKEVNYHGEPISVNVSINNSTNKVIKKIKI.[S]	4476.40716
9400	[I].QGLEGSADFLGPQGIRGYPGMAGPKGETGPQGYKGMVGSIGAAGSPG.[E]	4478.15036
9401	[A].GAGDCLSRKDWLAAQLVEGNGRCSGRVEVYFEGVGVHGVCL.[P]	4478.15507
9402	[L].APGRMPIYYQMSRLPAGYTLHETAPAARRESEALDSPSSKG.[E]	4478.19798
9403	[M].QLAHHGPHGLGHPHAGPPGSGGQPPPRPPPGMPHPGPPPMGMPPR.[G]	4478.19815
9404	[W].KPEDSKDPEKNTPKFLYTVPNGNNPAGNSVIDFYRKQRD.[A]	4479.23276
9405	[A].GQAPFQAQPAPPASRMLTGSHSFAASGMAGVPVVPLRGCGQVGFG.[S]	4483.18564
9406	[E].GPEVSASGVEDLGVLPSGEGHLEISASGVEDLSRLPSRGEDHLE.[T]	4483.18593
9407	[S].QPPSQHAAPSPVQHQAGQAPHLGSGQPQQNLYHPGALTGTPPSL.[P]	4484.22427
9408	[P].GGGLTPTAPPYGAGKHAPPQAFPPFPEGHPAVLPGEDPPPYSPLT.[S]	4484.235
9409	[P].VKFCRCYKEKCLVCRAVSEPFITHISEANIKHVPVPL.[F]	4485.31784
9410	[T].LQDLASFQPEVADALAMPLGDYTLYSPPPPAGGAILSFILNVL.[K]	4485.3302
9411	[F].HFILPFIIAALAMVDLLFLHETGSNNPTGIPSDADKIPFHP.[Y]	4485.33154
9412	[L].PGLAGHHGDQGAPGAVGPAGPRGPAGPSGPAGKDGRTGQPGAVGPAGIRGS.[Q]	4486.25836
9413	[L].AGPLGDPLGGDHLAAGGDVPPAPLAPAGPAPYSPPGPGPAPPAAMALRN.[D]	4486.26123
9414	[G].RGGNWPGSPQVSGPSPATRMPGMSPANPSLHSPVPDASHSPRAGT.[S]	4487.14801
9415	[I].NWTVMILYNYFNAMFVGPGFVPLGWKPENSQDSVYLQY.[C]	4488.1508

9416	[A].QREASGQQSIVEQPPGMMPNGQDMSTMESGPNNHGNFQGDSN.[F]	4488.89323
9417	[R].LAHTQSPMLQQSQANPAYQASSDMNGWAQGSMGANSMFSQQS.[P]	4488.93364
9418	[I].SMDMKKKGTMVKGIKADCIAVDWIGRNLYWTDGTAGQILA.[I]	4490.23389
9419	[T].HFSNSLPLMLRELRTAFSRVKNFFQMKDQLDSMLLTQ.[S]	4490.27814
9420	[M].SGARYGAPGSIASMNPGPGVQSPSSYQNSTYGLGVSSPPHGSPGLGS.[S]	4491.09059
9421	[A].ASAPAPGPASSPEASPAPGFPFPPPWMGMPLPPPFAFPPMPVPPA.[G]	4491.16909
9422	[F].SQLTSHTATAPSATPMFGQVATSTAPSLFGQQTGSNVSTAAAAPQV.[S]	4491.17326
9423	[P].PGPSGPLGHPGLPGPMGPPGDPGIQGYHGRKGERGMPGMPGKHGAK.[G]	4491.21319
9424	[A].RQPLAQRLPESAVFMPGDMGPPKQGGTRYGSISSPPSPGPQQA.[P]	4491.22961
9425	[G].SVPENLNLFPEPGSKSEETSKKQLSKDSILSLYGSQTPQMP.[T]	4491.25995
9426	[S].VSGVPGPYSIPSVPVGGAPSSGMLMDKPHPPPLAPSDSTGGSHSVR.[K]	4495.20206
9427	[V].PGKDGQAGHPGQPGPKGDPGVSGIPGAPGLPGPKGSTGGMGLPGMPGPKG.[V]	4495.22453
9428	[L].LTHTFNREFSQVHGSISDCKLSDLSPIGRDPSVSSFSSSTL.[T]	4496.17868
9429	[Y].SMRSCEAIPLQVPPEAVNMSLGLSMAATTNPFQLLACGPTVH.[H]	4496.19031
9430	[A].EPGPPGPPGPPGPMGLQGMQGPKGLDGAKGEKGSSGERGPSGLPGPAGP.[P]	4496.20854
9431	[I].PQDLWSAAGSPAAQPLAQAWMQLLDPARESVHVASFYWSLT.[G]	4496.21321
9432	[R].NGIGGASVSPGPGAPLTHLSTPSGGSELRQREGQRFGAAHVWENG.[T]	4496.22024
9433	[P].SQQQPTLPTPPALGEIPQELQSPIGEGGSSTQLLMPVEPEELG.[P]	4496.23888
9434	[F].NGKDFRIKQCTSVNMEDLVVAHHEMGHIQYFMQYKDL.[P]	4498.11993
9435	[V].IPDTLTDMVNQMTEKVGLVHGLPYVADRQGFAATLEQVYF.[G]	4499.22615
9436	[-].MQNVQPPKSSPVVSTVVSGASTARMPPAANRPVEPVASVTQPSE.[L]	4501.28137
9437	[G].RSDPGVPGAAGGEGPVELAHLARPGSPEAEEWSGPRGGLQEVGTPA.[G]	4502.20834
9438	[A].GQAGRPGNPGHQGLAGVPGMPGTKGGPGDKGEPGRQGFPGVSGPPGKE.[G]	4502.21305
9439	[E].KGEPGQGLRGSQGPPGKMGPPGNIGNPGLPGPRGHKGDRGDSSVQT.[F]	4502.24541
9440	[L].QRHHREQKSGAGPGPPPEPPPPPSQRGSGQSSGAKAAPQPATWV.[E]	4502.2573
9441	[S].VVVDLAAEAGGNFETTKPGELYVHKGITHIGYTDLPSRMATQ.[A]	4502.26604
9442	[R].GPFPGSGLGVPPHSELTNPASNLSTVAVLPVCAEVPMVAFMLEL.[Q]	4502.28083
9443	[S].RQSSPSSSAALPGPPAPLVDGSAVPGTALGTEPRHGGHCLNSSLLV.[T]	4502.28448
9444	[S].PPNSDLVPLDLSLGGASSSGPQESTCESPVTQLEGHEQPLGSTE.[P]	4504.0944
9445	[A].GPRSHPRTPPKALYGTTGVKENQALGSETSPGPEPSLPGAGVTAH.[P]	4504.29676
9446	[K].RGNGNGCATAQQRTKNATANLAKTQGSQGSPNSVKSSVSSRQSD.[D]	4506.18466
9447	[G].IDGKDGTPGMPGVKGSAGQAGRPGNPGHQGLAGVPGMPGTKGGPGDKGE.[P]	4506.2001
9448	[I].SDFGTSKRLAGITPCTETFTGTLQYMAPEIIDQGPRGYGKAA.[D]	4506.20681
9449	[R].SPSGRMRGEVSWAQMTASLLSVPPPSSSCRGPGVPAGPQASALQG.[Q]	4506.20749

9450	[A].ATAAASPSPPMAPVPAVIAESTTVDSPPSSPPPPPPPPQATAPSSPAP.[V]	4506.23849
9451	[M].AVPSRNTSKQMNLNPMDSPHSPISPLPPTLSPQPRGQEAESL.[D]	4506.2504
9452	[S].GALPGDDLSSRAKEFAFYPSFASSYQAMPGYLDVSVVPGISGH.[P]	4507.15509
9453	[A].AASAPAPGPASSPEASPAPGFPFPPPWMGMPLPPPFAFPPMPVPP.[A]	4507.16401
9454	[P].GEKGLTGETGPQGQKGEKGDVGPMGPEGPEGNTGPLGPTGLPGPTGPI.[G]	4507.20456
9455	[H].SATSPTLSTTGPAPSAHLGSANKTINSSSPELPTHTTTGSTSSAISP.[T]	4507.20706
9456	[Y].TLLNQAPDMLHRFYGKNSSYVHGGLDSNGKPADAVYGQKEI.[H]	4507.20992
9457	[Q].ASGQPSLQSQWQPQLQQLRDMGIQDDELSLRALQATGGDIQ.[A]	4507.22703
9458	[A].GKEAEETTFEAGVKVQIHSQSEPPFIQELGFGVAPGFQTFVA.[T]	4507.24562
9459	[R].ITGPEEDGVRVVCTTSGWFPKPQVQWRDLSGEKFLEFSE.[A]	4511.19762
9460	[Y].VVSLCVANSAGNKSSSPLTFEGYELLQPDPPVNITVRPVDGSP.[R]	4511.27627
9461	[V].PNVSVSVSTSHTSISGGGGRGGGGFSSGGGGYGSGGGGGYGSGGGGGYSSRGGG.[S]	4511.98159
9462	[A].LGGRGRTLRTGDCEAMRPPKLPGPALAPLLLLLLRPPPGSAGGP.[A]	4512.51701
9463	[S].QTPAAPRPRISEGFTRSSTRPALMPNQDPFLQAHTRGAALPG.[P]	4514.32221
9464	[W].DPLKDIAQFEQDGVLHTLQRGTMASQTAVLGSPGPGLQGSAPNP.[R]	4515.25726
9465	[A].WAGLGPGQGEQTVTVAVVFGSSGSPQAQAHTHLTPQSFLDLPLE.[I]	4515.25792
9466	[Q].GLAGVPGMPGTKGGPGDKGEPGRQGFPGVSGPPGKEGDHGERGPVGQP.[G]	4516.21747
9467	[V].QRFWPDSIASGAAQPAASELTVSEGAVVTITAPVSMNVDSLQSL.[S]	4517.25045
9468	[P].RAAASGGAAGAAGYPPVQYVQPMHKGPVGPPFREGKGQYLGEHR.[L]	4517.27961
9469	[Q].GPPAGPAAPSSAPASSSPAAPAGALDRHQDSPVTSLRNWVDGVFHD.[P]	4518.18213
9470	[V].GPAVSSGVNVNILSGMGNGTIASSAALNSAASAAAGMTVGSVSSQQQQP.[A]	4518.18351
9471	[T].AMDSLVKNGINFKVYDHVRVEPTNTSFMEAIEFAKKGAFD.[A]	4519.24247
9472	[A].GQVTSGHGAAAATSASTGQAPEDPSGPGIGSSGTCEAQVAVVTVTPAPE.[P]	4520.11178
9473	[R].EEEVEIKVSEHATPGSRFPLPNARDPDVGMNSLQRYQLNP.[N]	4520.2263
9474	[F].GENLDPAILEEVDKELALCDLCLVVGTSSVVYPAAMFAPQVS.[A]	4520.22852
9475	[P].APDGSLPYLSHGASQRAGITSPVEKREDPGAGTGSSLAAPELSGTQ.[D]	4520.2288
9476	[A].APSPTHATSHSHITTSHSHTTVSPIHITTSPTHTTTGPTHTTAS.[P]	4521.17777
9477	[L].HQPQGEQGQAGIQGPPGPPGPPGPSGPLGHPGLPGPMGPPGDPGIQGY.[H]	4521.17929
9478	[A].LSSSVPSSSTPSGPHTTATPSVTASALGPSTPRSATSHSISELSPDS.[E]	4521.18633
9479	[S].SPVPTPSPSTPAPPTSSPSSPAPPNPSPSNPTPASFAPVPAPEASPPA.[P]	4521.20963
9480	[A].AATAAPAGFFGSTQNKGFGFGTGFGTTTGTSTGLGTGLGTGLGFGGFNT.[Q]	4522.1586
9481	[G].RQASQTEGADGPRTRCPVYIYSCSLEALREQMVGVQPPQA.[P]	4522.16602
9482	[G].APVTSAGMFGGTSLRFGLNFSGAYGAAAVASTTASTTTTTTVTTTTT.[T]	4522.19299
9483	[K].KPEVNWGSSVQAGSYNLALSYSVGLNEVEDHIKNYRPQCL.[V]	4522.20959

9484	[V].KFVRMAGDETQPTRFAFVEFADQNSVPRALAFNGVMFGDR.[P]	4522.21832
9485	[D].GPQVLTGRFMYGPLDMVTLTGEKVDVHIMMQPPSGEWLYL.[D]	4522.23177
9486	[P].QGPIGTPGEKGPPGNPGIPGLPGSEGPPGPAGSAGPPGYPGPRGVKGEDG.[F]	4522.23858
9487	[D].HAPVFEQAQYRETLRENVEEGYPILQLRATDGDAPPNANL.[R]	4522.23858
9488	[S].VHDIMKAFQSGRDPSKELAGLFEHKSAVSPDVHKSAAETSAQ.[H]	4522.24195
9489	[Y].QGVHELLCVVSRACAPQMQTAYEIKTQRCAAHSSAFLSTT.[L]	4523.16867
9490	[P].GPSLGAVLCPMPTLPHTGEAEAGVCRGSHWCPARTRAVTSHAA.[P]	4523.17
9491	[G].GLGGFGGGISGAVGGFGGLGGFGGAVGGGDAGILPADEKTTMQELNSRLA.[S]	4523.22596
9492	[V].GEPGVAGPTGPPGVPGSPGLTGPPGPPGPPGPPGAPGAFDETGIAGLHLPN.[G]	4523.263
9493	[S].PGHMGPPGPTGPKGTSGHPGEKGERGLQGEPGPQGSMGQRGEPGPKG.[D]	4524.16438
9494	[L].RAGYALTLFAFNNRLQQYLILENGKMSISIFEPFLESTI.[E]	4524.36357
9495	[A].ATSAAAPTAAASPSPSPSSSSTGVFGNLPLTSAGSSGLFSFGGLFFSSS.[K]	4525.16816
9496	[F].TSLTGLLEVEPLHFSCVSTSDGTRIERDDAMSSFGVTPAVGGL.[S]	4525.18613
9497	[P].DNILLDEHGHVHITDFNIAAMLPGEMRITTVAGTKPYMGAGA.[E]	4525.19487
9498	[M].RLDPSADSAAGPLDWLEGGLHWQCSPPDLELELKAKGGASDPA.[A]	4527.18852
9499	[-].MRLDPSADSAAGPLDWLEGGLHWQCSPPDLELELKAKGGASDPA.[A]	4527.18852
9500	[Q].NNLAPSSSTLSHGMATTSTAYGVKKNMPQSPTVVSTGVSTSAASTT.[N]	4527.19776
9501	[R].PSGAATTTAAAAASAPAPGPASSPEASPAPGFPFPPPWMGMPLPPPFA.[F]	4528.16282
9502	[M].AEPPEALGPPSSQAFLSFSTAPMAGGGLPAGEDPGALLANSHGAAQAP.[S]	4528.17253
9503	[V].APKALTETSVVGCDSQALNMLADLALSAATSSTPSPEPRNLPCS.[L]	4528.2004
9504	[L].HLEVSSTYLSLSVYFEGSETALNGVGRFFRALAKEKQEGAH.[L]	4528.28955
9505	[R].QAGAGAGPPNPAINGSAPRDLFDMKPFEDALRMPPPPQSIAMAE.[Q]	4530.20028
9506	[R].PASASPAPNATADGSKTSRASVDTTPSVIQHRAMMRFSELEMK.[E]	4531.21264
9507	[I].EGYISIVMDAETQKKFPSDLLLTSSSGELWRMVPEDGISSV.[I]	4531.22587
9508	[W].VGSEDSVIYIINIHSMSCNKQLTDHRSSVTGLAVQDGVQATS.[T]	4533.19843
9509	[P].AATEGPETKPVLMALGEGPGAEGPRLASPSGSTSSGLEVVAPEGTSAP.[A]	4533.23011
9510	[D].VQGHDLMGNVTLDHFGEVPGGLAGGGQGREVQWQVFVPSAESR.[E]	4534.19567
9511	[L].PGSPGAKGEQGPAGHPGEAGLPGPSGNMGPQGPKGIPGNPGLPGPKGEMG.[P]	4534.19904
9512	[H].PEAGRLGGGPALYPPPEGQVCNPLDSLDLDNTQLDFVAILDEA.[Q]	4534.20825
9513	[L].ALPSEHLGGGLGMGAASRELSSQASGSLAHPAPINLEDSLDGDLVH.[N]	4534.22669
9514	[Q].GQPGLPGHPGPMGPPGLPGLDGLKGDKGNPGWPGTPGAPGPKGDPGFQ.[G]	4534.23608
9515	[P].GPGLLAQPLGGPGTPSSPGAQRRTPYEWMRRSVAAGGGGGSGKTRT.[K]	4534.32327
9516	[N].SRFHPGSMSVIDDRPADTGSGAGLRVNTSEGVVLLSYSGQKTEG.[P]	4535.22194
9517	[A].AAPAVEEVLPVGAPFPGDAPHADSVPVSEGTSILEEASPAGMPIQQ.[E]	4535.22865

9518	[P].GTPGVPGKDGQAGHPGQPGPKGDPGVSGIPGAPGLPGPKGSTGGMGLPGMP.[G]	4535.25582
9519	[G].KGGEDDILTSERCSSLVPLGDGGTARIPGPAGRVTNECLHTED.[W]	4537.16819
9520	[P].DIVLYNNADGEFVVTHMTKAHLFSSGMVHWVPPAIYKSSC.[S]	4537.17776
9521	[S].AAGYDAKAFGSPVIDLSSPVGGPYNLPSLPDIDCSSTIMLDNIV.[R]	4537.21531
9522	[N].KDVSPVMHFSSKFAAAEITEELFFQSANGSERRPGLLAESPG.[Q]	4537.24564
9523	[G].GPAPLSSAASSPLSSSLGTVGHRANSPSLFGTEGKPKMEPVASSQAA.[M]	4537.26274
9524	[S].TAGDGRGVTLCSGAKAEKESFEKVYQVGAVLGSGGFGTVYAGSRI.[A]	4537.278
9525	[A].PGAGLLPPTINSREQIPGDRQVCMEGVAGPAGYLRRASVAQMT.[Q]	4537.28608
9526	[N].NIPGEYLGSVLAQDPDLGQNGTVSYSILPSHIGDVSIYTYVSV.[N]	4538.26133
9527	[M].KGLSHVAMGQFYEGIKAQTKVMLNDPLPGQKASPEIADPDQP.[V]	4538.26941
9528	[A].KDTPQEEVVKVVTSTDMAEYPLVASTGQRCCLQDILAGGCP.[V]	4539.15101
9529	[N].PGAMLELGPPHGVSAEEAGLGPQMAGQPLEAEEDRFVAPQQALQ.[G]	4539.19189
9530	[A].EKSGKETTSLGMSSLPTSDGFNHQAHPPGLSPEIGNPPSRAHSV.[S]	4539.19573
9531	[T].GEVAQDTQRERRVFQLHMCEYLLKAGESQMKQGPDFLQ.[S]	4539.1966
9532	[Q].ASNTPSHIPISSSFSTSVYQPIPQPTTPVSSFTSGSMLGRTDTA.[L]	4539.19841
9533	[G].IPGWGVCGWIATISFFGTNIGSAVVMLIPTVMFTVMAVFSFV.[A]	4539.30275
9534	[E].PGWQSLGGSVFPSPEEAPSATSPGTEPTGTSEPLRTGTVSAELSSP.[W]	4540.1638
9535	[D].QARPSQNLEAPLGSPRGSLDPPVPRASRGQMLLSGGPRGPVPQP.[G]	4541.39062
9536	[M].QKIYQTFVALAAQLQSIHENVKVLKEQYLGYRKMFLGDA.[V]	4541.43774
9537	[Q].QAHLKMSSPQFSQAMPSRPMAPMSSAAVAGPMLPAGNAQQRTSG.[P]	4542.15672
9538	[V].QAKTTQGFGPEREGIITIDWAATRGSSSTQCQGNTAASPPTPAP.[L]	4543.19064
9539	[L].GPSLEDLFNFCSRKFTMKTILMLADQMISRLEYVHTHN.[L]	4543.23931
9540	[D].QVGVPRSIAANMTFAEIVTPFNIDRLQELVRRGNSQYPGAK.[Y]	4543.39906
9541	[M].YHRNESLQPSLQGPQTELRSDFQCVVGFGGIHSTPSTVLSD.[Q]	4544.18991
9542	[Y].KSCTVSINFGPCFKYPPKDLAYRPMSDMGWGAVVEHTLAD.[V]	4545.14983
9543	[S].PSTVTSGGGHHDPAGPGQPLHVPGSPAAAGQEEAGGGGGPGQTPRPLED.[T]	4546.13663
9544	[Q].KQIDPETFKDFYNCWKEAEAEAQEVSLPLSVMEQLDKN.[E]	4546.14287
9545	[S].PGAKGEQGPAGHPGEAGLPGPSGNMGPQGPKGIPGNPGLPGPKGEMGPVG.[P]	4546.23542
9546	[P].VDIVAKFEDAEEISSSTYFPGSVIDYPEDISIPLDQTTIQD.[G]	4547.17632
9547	[P].GPQGVPGPPGFGGEPGPQGEPGPPGDRGLKGENGVGQPGLPGAPGQGGAPG.[P]	4547.20867
9548	[P].ATTPTGSQPPSQHAAPSPVQHQAGQAPHLGSGQPQQNLYHPGALT.[G]	4547.21991
9549	[G].EAAADIRPHGVHMVLNQQGRPSGDAFIQMTSAERALAAAQRC.[H]	4547.22013
9550	[P].HSVPGGPQAQATMTPPPNLTPPPMNLPPPLLQRNMAASNIGISH.[S]	4547.31084
9551	[L].YICLGTLGYMKFGSNTQASITLNLPNCWLYQSVKLMYSI.[G]	4548.24742

9552	[D].GQPAAAEATATPTLEGRVGESAPTPLQAPRAGQASCKVPRHWEP.[L]	4548.28007
9553	[A].GAPGAGASKDQCCSGKGDLSDEDDENEFFDAPEIITVPENLGH.[K]	4548.96782
9554	[L].WVSQPPEIRTQEGSPAILPCSFNASQGSMAIGSVTWYRDKV.[A]	4550.22313
9555	[G].DPLTGLGGVMAQAGFNGGNLTNFFSLPGSRTPDIVNIQETTNVN.[V]	4550.22563
9556	[H].VDAAAGAVPPSPPAAALGATCAAAFPSAASVTSAGATSASSVHLPVSAPHG.[A]	4551.25726
9557	[A].NTGESEGKKRTEALYTPAGGEKPGASVASVHSEAGPKGAEKPAAATG.[K]	4551.271
9558	[T].EGRPGYVEISTFRNIEDVRSTMATFLLLRIPTLKIKTASK.[K]	4551.51196
9559	[A].AEGVGAAANAAATSSTGTGGVAASGMAASGVVPGGGFVASAAAEVQTGRNNF.[V]	4552.17572
9560	[I].SGAAAPAPAPSGLGYGPPTSLASASGSFPNSGLYGSYPQGQAPPLGQGH.[P]	4552.18039
9561	[R].REEGSPGAVSRDGSGRKGEGLWGERPSSRGVREAGSWPREEAA.[P]	4552.21728
9562	[R].PGRAHGGDPSHLHAMSVAQPVRFGSKLQACPGADPGRTPRHPE.[T]	4552.23341
9563	[R].APAAESQAPFPSGSPTLPAQAQAGQMMPLSSARPTSGSVGVMLAAGR.[N]	4552.23813
9564	[H].ANGLLPSAPSAASNNSNSLNVNNGVPGGAAAAAAATVAAASATTAASSSLAT.[P]	4552.25099
9565	[F].PVYSASQTVLGDGLLQPAPGAAREPSAPLTDTSSLGAAGEGPPGAPSH.[A]	4552.25904
9566	[S].MAPRTELPRPDPGGPGGGRQADDLRSPNPAKHRALAWPGPWPG.[H]	4552.29157
9567	[S].EGQVPSESLGGRWPLAGPCPAVSLDATGGDRLWQRLEPGSHRG.[S]	4553.2491
9568	[T].TTKRDEYGPGVQGKEPPPAHVDAAAGAVPPSPPAAALGATCAAAFPS.[A]	4553.25179
9569	[S].QTSPPLAQSLSFTRQDKHTSEKPVMWQAQALPAACSLSVGSP.[R]	4553.25516
9570	[Y].LTTSITLTTDTVTNPEHCQKQPPRNLVCAEQAGGRGLHSPPA.[I]	4553.26237
9571	[L].PRDDRVGAPGLPGPAGPKGDPGSRGPMGMRGPPGLQGPPGSPGQAGAVG.[I]	4553.2637
9572	[G].LGDPSARSTSPGRADLPGSSTTLTKSFISSSPSSPSRAKGLNGHPS.[H]	4553.29788
9573	[K].WGPSKKGKEQSDRALDMSGAARGHGHFLGIVGGSPAGGGGLAFYPG.[V]	4554.24837
9574	[R].PGPPRDEPRSNGRREEKAEKPRFMFNIADGGFTELHTLW.[Q]	4554.24837
9575	[V].AGPTGPPGVPGSPGLTGPPGPPGPPGPPGAPGAFDETGIAGLHLPNGGVEGA.[V]	4554.26882
9576	[P].GAPGREPSPTTGSQGSSKSCRNLKRGSPGAGAAGISPGHSPLQGLIN.[C]	4554.29784
9577	[L].GPAGGGNSGSSLPTPAGGLLFPIDAGIAGESSSRSRHSPYAWMRKT.[V]	4555.25352
9578	[Q].ASLAPTPVASPMTPSAASFSFGSSGFKPTLESTPMPSVSAPNVGMK.[P]	4556.20336
9579	[F].GRPPFDPNMPPMPPPGGIPPPMGPPHLQRPPFMPPPMGTMPPP.[P]	4556.21832
9580	[-].MAAQAQAQARDGGAQLAGPAAEADPLGRFTCPVCLEVYEKPVQ.[V]	4558.19118
9581	[Y].KYRPGHSSSASAAMPHSSSAKLSRGDSLKEPTSIAESSRHPSY.[R]	4558.21277
9582	[V].VYLEGDLVSTANTHSNNSERSLHLNIGSSMSEHLKFKNEGQ.[N]	4559.18556
9583	[W].HRTATTQETDGFQVKRPGDVNVRCTVLLMLDYQPPQFKL.[D]	4559.3286
9584	[L].PQTPGAPRQETSGRMPPVLQKGPSLLYPGASEQDTRIQGPLTS.[P]	4559.3311
9585	[A].GPRPPSATPRPRLAPLSRDQEQVPPASMGPEPALAASGLNRAMT.[S]	4561.35146

9586	[Q].GARSTKTRSPGPEGMNYPVRKMLTPPIPALESKAPETKEGGQP.[K]	4561.36538
9587	[D].NAPEVIFQSLPDFIMEDTKLGTHIALLKIRDKDSGHNGEVI.[C]	4561.37592
9588	[P].GTPGATPPPPQFPAGIWGSGLGGAEGGAPASRAGLPSGPGPGCRVTRSP.[A]	4562.27459
9589	[K].PASSGPLSHPAPLSASSSSLSSKSSVTPSASGRAAQGSPSPVPSMVQK.[S]	4562.27912
9590	[S].FANNSGFILELAAQQGALVVFAEHVGTGQAGGHGGVALGAQSYYGK.[S]	4562.28513
9591	[I].APAAAEPPSEPKEVRVGARRRDVPKVPHDSSKDGVTVFVSNLP.[Y]	4562.42263
9592	[P].NGPMPLPVNRMQVSQGMNSFNPISLGNVQLPQAPMGPRAASPM.[N]	4563.21859
9593	[Q].VWEPLQDTEHLIMDLRQNPGGPSSSVPLLLSYFQNPDASPV.[R]	4563.25005
9594	[E].AVEPASDHSLGQSPQIQILEEMKPLESLAVGEASDLGSQSRRS.[K]	4563.26314
9595	[R].HLAYLKWISQIEELSDNIQQYLMTNNVPEAASTLVSMAEL.[D]	4563.27858
9596	[L].TPVPTQMPWLVASPEPPQSSPTPAFPLAVSYDINGPPQPPLPE.[K]	4563.27923
9597	[V].PGAPGMPPGIPPLMPGVPPLMPGMPPVMPGMPPGLHHQRKYTQS.[F]	4563.28165
9598	[Q].ASQGTGSPIPKIHGSSFVTSAVKQEDSLFASMPPLCPIGSHPKV.[Q]	4563.30104
9599	[V].RDSVTGTPEGSISVSSSSLSGTCGKSETNGTDVDSFQEADEQVR.[V]	4564.04997
9600	[S].SGATRAVEVMPKAGALNSNDAFVLKTPSAAYLWVGAGASEAEKTGA.[Q]	4564.31405
9601	[T].TLQNISEDVLAVMDNKNPTIKQQTSLFIARSFRHCTASTL.[P]	4564.32866
9602	[M].KEELTGTLQSAGTGSEEGARGLGGGIKSSGGGQELAPEDGPLVQDPQ.[K]	4565.22377
9603	[N].PSKQNQSAPAAAPFTSSSAANGLESSVATDSSKLATITTPMALNTSG.[I]	4565.23117
9604	[P].PGMFPLWPPMGPFPPVPPPPGSGEAAAPPSTSAAALSRPSGAATTTAA.[A]	4565.2632
9605	[K].GDKGSPGKTGPRGGVGDPGVAGLPGEKGEKGESGEPGPKGQQGVRGEPG.[Y]	4565.27273
9606	[Q].QHRSGGRGTKTRNSNNNNTAAATVAAAGPAAAAVGMGVRPVSGDLAY.[L]	4565.28867
9607	[A].ATGKGWPEAKGQGPSPQRPLSEASKPSGMKRSPSATVQSSLRCA.[T]	4565.30999
9608	[S].AEARTDGLTTEHFRQAEVPVHLMWQTPGVSAQGTMVETKEQ.[G]	4566.21402
9609	[R].GSPVRQSFRKDSGSSSVFAESPGGKTRSTGGSSTAGAPPSELFPGPG.[E]	4566.22438
9610	[P].QRALGDQGWGPLGGVGVTGNPGTPHPPMADSGSWGAPMLELSLAAR.[Q]	4566.24051
9611	[A].PDSGRPAPYSAAFLELQPGPAGSGYPAAAPPASFASHFLQGGPFPL.[P]	4566.25171
9612	[R].GPAGPPGPQGPPGEQGPEGIGKPGAPGTPGQPGIPGMKGHSGAPGPAGLPGA.[P]	4566.25827
9613	[A].LDGRATTAQSMAARGSALETGGVPWIKMVGTVCVKWVGVGQAAM.[L]	4566.2724
9614	[-].MEKFQAAMLLGAVGDALGFGHTARESSGSGARVQEELGKGGGLDHL.[V]	4566.2794
9615	[S].GGFLSNQGSFEEDDDDDWDDWDDGCTVVEEPRAGGLGTNGHP.[P]	4567.83998
9616	[V].RAELEALPSPEGRCSETPGKLQDSSVGGQGAQGSQPKPAAAGGPHT.[L]	4568.21825
9617	[M].KEVCLGSPGTPGTPGSHGLPGRDGRDGIKGDPGPPGPMGPPGGMPGLP.[G]	4568.22314
9618	[L].QSLSASTLTMSSGSSRGSLASSRGSLASSRGSLSSVSFTDIYGLPQ.[Y]	4568.2533
9619	[G].IGACCLALENSPPGIYIHSLAPGSVAKMESNLSRGDQILEVNS.[V]	4568.25819

9620	[L].APGTRRDIYDQKLTLQPVDNSTISLQMGTNKVASQKGMSVYG.[L]	4568.32357
9621	[L].GPDPPVAFPQVYTSGKGSSAAGLTASVMRDPSSRNFIMEGGAMVL.[A]	4569.22108
9622	[E].EGGVGRMVDARHFPMSGFRLTFVDLVHSVLNRFYYNNHV.[L]	4569.24554
9623	[Y].EFFVLMNSVQNLPAPFSRENVLTFRQTCPEHSRTTLGVM.[M]	4569.24757
9624	[G].PGAFSAMGMGHLLASVAGSGGGGGGGGPGTATGLDAGGLGPAGNAASTAGPFPF.[H]	4570.15142
9625	[P].QYLMELARYRRWGDSVLFVDLEHEDMPQNVVAATSGLKT.[F]	4570.24934
9626	[R].GPEVQQRPDGSLARPALSPGASPAGGGYYLAVGGAAAQHSWSHISAA.[L]	4570.26104
9627	[H].RHYPAPEGPAPAPPGPLPPAPNSGTGPSGVAGGRRLGKCEAAGENSD.[G]	4571.22328
9628	[E].SQATPSHAPGVPTLASSPALEAEEVWLSTMAPSPSSVEASTVVGTH.[A]	4571.22463
9629	[E].VAAATTMEATSREAAPAKSSASGPSAPPALFELCGRAVSAHMGVLE.[S]	4571.23271
9630	[G].ADSYLVQQPVDADALGLHQQVPGGPGECGPLELGAGLPELFLGEG.[A]	4571.23988
9631	[H].AALENGSALATTASPAPEEQKAQAAPEEEAATVAVPEKGVGNGRAPD.[V]	4571.2496
9632	[V].KTDPSPSENRNPMFLENMDSKSSKQLDSISIGKEDAGSLRM.[A]	4572.16508
9633	[V].ARSGGAGGGAYGFRSGAGGFGSRSLYNLGGNKSISISMAGGSRAGGFGGG.[R]	4572.19338
9634	[T].IAGTSGNKVSPDVMDKWQALSTLLEAFGNSPTVMNGNATRFSQ.[I]	4572.21335
9635	[G].LEAIGLQDPGPGAMTMKAAEPQGGPGGGERVGSSMWPEPRVPLDL.[G]	4572.23198
9636	[T].VGQSLTQPVMSQSANLPVPQGMSQFQFSAQLGAMQHLKDQLE.[Q]	4572.23198
9637	[G].GPAGAAGGAAGGGPAAGPADHGLAGRGAAGDGPAAALLQAAGVAADWAAAGLADGA.[R]	4572.23629
9638	[L].TLSPGPEAHQGFSRQLSSTSPLNPYPASQMVSSDRSPLSFLPT.[E]	4572.24637
9639	[L].PARRAPHSEDREEGSRASAPPSRLSPSPGGSSRLLITEPQPGSP.[L]	4574.32068
9640	[S].AQNGESSPSSSSSAGDLAHANGLLPSAPSAASNNSNSLNVNNGVPGGAAA.[A]	4576.11668
9641	[Q].SHLYQPAFPGMVPASLPGPSNVSGSPQLCSSPDSSDFPGRTAPY.[R]	4576.11839
9642	[D].QHSQGYPSPLGGSEHASSPMSTPDPALGGTPRPGPSPGPGPSPGAMLG.[P]	4576.1256
9643	[A].PSAGFGGNLHPGARAGGASSPSPVVFTVGSPPSGTTPPQGPRTTMFSV.[G]	4577.26302
9644	[D].KGDQGLSGFPGSPGEKGEKGSTGIPGMPGSPGPKGSPGSVGYPGSPGLPG.[E]	4578.22054
9645	[E].ALGPPSSQAFLSFSTAPMAGGGLPAGEDPGALLANSHGAAQAPSNSLTA.[A]	4578.22054
9646	[R].CPEIVHGLAQFPDTLPGPGGLAEVAGTCVPHAHVSPGPSGAPRMH.[C]	4578.22275
9647	[S].PGAKGEQGPAGHPGEAGLPGPSGNMGPQGPKGIPGNPGLPGPKGEMGPVG.[P]	4578.22525
9648	[P].PQSPVFEGVYNNSRMLHFLTAVVGSTCDVKVKNGTTYEGIF.[K]	4578.24319
9649	[Q].GDPVPGQQESPRTPYERHQVSKGRPGPVAGHAQMPRAPAQQY.[Y]	4578.25558
9650	[P].GKPGPAGMKGEDGLPGSPGEKGEKGETGQPGPPGLDGPTGEKGEPGDPG.[R]	4580.14817
9651	[L].QGLHDQLLNSNLDPGEVEKAKDGQKADFPTGIPECGTDALRF.[G]	4581.23144
9652	[P].ATSQSPQVTSPPTQTPHSAPDPTVTPVGSSGDHLTPMAHPLDQPP.[P]	4582.17907
9653	[K].PATSQSPQVTSPPTQTPHSAPDPTVTPVGSSGDHLTPMAHPLDQP.[P]	4582.17907

9654	[T].APSLFGQQTGSNVSTAAAAPQVSSSGFGSPAFGASTPGVFGQPGFGQAP.[A]	4582.19096
9655	[T].SAHKGTSSGATMAPASKATPSSVPSSETAPSAASHITRTAASSTSPQQ.[L]	4582.20741
9656	[P].PGLPPPPPPGMLMPPMPGPGPGPGPGPGPGPGHSMRLPVPQGHGQP.[P]	4582.24032
9657	[A].QVGTTEKPLRKPPARLKKLKIKKEGKDFTMKDIEEKMRA.[V]	4582.60515
9658	[S].EPQDVGMQPLLAKSPSRQEAHEALSPGEAAGGQAEARREFLEP.[V]	4584.25358
9659	[N].KEPIPDDGIYWQANLDRFHQHFRDQAIVSAVANRMDQTS.[S]	4585.20657
9660	[S].TAPSLFGQQTGSNVSTAAAAPQVSSSGFGSPAFGASTPGVFGQPGFGQA.[P]	4586.18587
9661	[W].RDSSVGDKPAVSSWAAGGDPGENVPLSGMPAGSLLCSPLPNHLAQS.[P]	4586.20385
9662	[A].VLFPEHSYSASGVYHQIQPTYDLNGYLSYIKSLPLNDMPE.[I]	4586.22244
9663	[K].GRALYDFRSENKEEISIWQDEDLVIFSETSLDGWLQGQN.[S]	4588.19029
9664	[P].GAREPPPPAPAPAHHHPEYQGQPVVSHPHHIMPPQQHYAPPP.[P]	4588.23808
9665	[L].FYKMVQQLGETEAAALTERAKQVSLLWGVVIKVHLQGSPFT.[-]	4589.45887
9666	[R].REGLHSADALQAVTFDMELVNLDGAFDLASGRFLCTAPGVYF.[L]	4590.20681
9667	[F].FDILLCMARNVQREDFLVGRPPEMGQGDADVAGYLHGARAE.[L]	4590.20749
9668	[E].KSELKMEIDDMASNIETVSKSKSNVERMCRTVEDQFNEI.[K]	4591.17829
9669	[G].HLDPGFLASEKTSAGNAPLNEEINIASSDSEVEIVGVQEHARC.[V]	4591.20054
9670	[Q].GQPVVSHPHHIMPPQQHYAPPPPPPPPISHPMPHPPQAAGTPH.[L]	4592.27678
9671	[P].KTYGFGSVEELLGAIPQVVWIKGHGHKRIVVLKNDMKSRVS.[S]	4592.52862
9672	[G].PSGDRRGDVYRCLVGGSHSAPCAKGHLGDHPLGNSSRPAVNMH.[L]	4595.16982
9673	[I].GSGVERMGAGMGFGLERMAAPIDRVGQTIERMGSGVERMGPAIE.[R]	4595.2044
9674	[G].PQGNAGPQGHLGPQGPPGPQGHIGPQGPPGPQGHLGPQGPPGTPGMQGP.[P]	4595.21338
9675	[P].GPSLAETGSGTGDLAPPGTGGSGALGDLHLTTLYSAFMELEPTPPAAP.[A]	4595.21339
9676	[L].GQPGTLFGDDQIYNVIVTAHAFVMIFFMVMPIMIGGFGNWL.[V]	4595.23104
9677	[V].TESGLTPIHVAAFMGHVNIVSQLMHHGASPNTTNVRGETALHM.[A]	4595.23404
9678	[G].GNLEEAQKTSTQTGRWQEPSLNSSLVPSPRLVAGPFPLHLGPV.[C]	4595.4005
9679	[P].GLMGEKVTGERGSCLRALGHGGGSDARSCRRDPLVSEVRQDPG.[D]	4596.23248
9680	[D].GAGATGSTVPAALGPAQPFFKEEKEGGVEEAGGPPASLCKLEGGEEL.[E]	4597.24028
9681	[T].NVGFDPKDPYRTPTAREVLKDMGQRGMSYAKNFAIVGAMFS.[C]	4597.24248
9682	[E].GIDAQVVNGYVIHDQESKTEVQIPFPLSENNHVQSGRAFHH.[G]	4597.26071
9683	[F].RVTSMQGVINETPLPIDLYQFDDISGKVEQFGKFGVIYYD.[I]	4598.27996
9684	[L].QGASQLPANASLAAMAAAAGLNPGLMAPSQFAAGKDRSPFCPASSPK.[S]	4599.25411
9685	[L].LDESLKDGDRQVIDSSIDGPVVNLVATEVPAGALGTHLSQLELE.[E]	4600.36283
9686	[P].LGTSPASSQPGTVTSYGPTSSVALGFTSLGPSGPAFVQPLLSGQAPLL.[A]	4600.38211
9687	[P].AGPQPPPQPQPPLPSQPQAQKRRFTEELPDERESGLLGYQH.[G]	4601.3284

9688	[T].GPKGGPPGIDGKDGTPGMPGVKGSAGQAGRPGNPGHQGLAGVPGMPGTKGG.[P]	4602.26885
9689	[S].QKSAIPAMVGDYIAAFEAISPEVLRHVINMADGNGNTALHYSV.[S]	4602.27556
9690	[A].AGDGPAAALLQAAGVAADWAAAGLADGARAAGHAGHGPAHGGLAGHGAAAAGVA.[V]	4602.28457
9691	[E].SQAEIQATSTISPSSETLDSQGEVFENTLVQNEPPAATELNVGN.[V]	4603.18057
9692	[V].NDLWPSSPPKASPSSLRSGPCSRPSSASGSRSPSRSSVSSRSWS.[R]	4603.20909
9693	[G].SADFLGPQGIRGYPGMAGPKGETGPQGYKGMVGSIGAAGSPGEEGPRG.[P]	4603.20927
9694	[G].SIPQMLNGEVYPPSVEEAPVLMRYPEGIPPQSQMAVGQEVFG.[L]	4603.21935
9695	[D].WLAMLREASDEVVAEEGAEVKLAEGSRCVEDVSSRPRVPEM.[D]	4603.22254
9696	[P].GLPGRDGMTGAPGLTGERGEKGEPGERGPPGFPAYLDEELQGTLH.[E]	4603.22703
9697	[R].GPVGPQGRRGPPGAQGEMGPQGPPGEPGFRGAQGKAGPQGRGGMSANPG.[F]	4603.22905
9698	[G].EAGLPGPSGNMGPQGPKGIPGNPGLPGPKGEMGPVGPAGNPGAKGERGSS.[G]	4603.24163
9699	[T].QASLEPALSLSDRPGEEVCLSLGEGTSLAALSGPSHSLDTSSGHLA.[L]	4604.24207
9700	[R].QKSAYTAYPKAEPTPVASSAPPASSLYSSPVNSSAPLAEDIDPEL.[A]	4604.25664
9701	[P].SPPPDGSPAATPEIRVNHEPEPAGAATPGAALPKSPSQAAPSPAADPQ.[L]	4604.26519
9702	[W].QDVSVGSWNQPPRLGRQMSDGVGEKLFQDLYPFMLGEHGLT.[S]	4605.22894
9703	[G].QQAFGEGGANKGYVPQGVYGRGGYPGGPGFTTGYAGGPGGPGGLGLPSH.[S]	4606.1923
9704	[K].KESFAPGTMYKPFGKEAAGTMTLSQFQTLHEKDQETASLRE.[L]	4606.22285
9705	[Q].LGPLSAMASGRGLRPLLLLLLLLSPSPAASASDRPRGSDPVNPEK.[L]	4606.55014
9706	[A].AGPPPNSMGLSNSLAGSNGAGLQSHLYQPAFPGMVPASLPGPSNVSGS.[P]	4607.19295
9707	[C].YKSISGRYMSIAKFGTCYVLTKSIPSAEVEKWTQSSSSAFL.[E]	4608.3153
9708	[R].CWSSDTYNPVPGVMDGVPSANNYQGGFGTTLMAKDLGLAQDSAT.[S]	4609.05922
9709	[P].GPPEGPVQVTGVTAEKCTLTWSPPLQDGGSDISHYVVEKRETS.[R]	4609.25151
9710	[L].PGGSIPQMLNGEVYPPSVEEAPVLMRYPEGIPPQSQMAVGQEV.[F]	4610.22516
9711	[G].APTQYPPGRAGPPPPMGRGAPPPGMMGPPPGMRPPMGPPMGIPPGR.[G]	4610.23208
9712	[W].AQASVTHGAHGDGGRASSLLSRFSREEFPTLQAAGDQDKAAKER.[E]	4610.2843
9713	[P].GDVGPPGPQGPPGKPGPAGMKGEDGLPGSPGEKGEKGETGQPGPPGLDGP.[T]	4611.20562
9714	[R].KQFETENEVKSSFRQEASRLTMEKRPRGVVVNMIPGLPAH.[I]	4612.38751
9715	[T].QMAATSVLSQSSLPAVEGEEETALPKGRNSSGQEPSWPPLSRTS.[A]	4613.2424
9716	[K].IEPELDGSSPKQENGLTAEDWCEGADDWGSDSEEASPPQPVAD.[F]	4614.94852
9717	[D].GTPCGEEGYCFNGSCTDRNVHCKEIFGRGAFNAPNSCYIL.[N]	4614.97407
9718	[L].KNQGAISEPIFAFYLSKGNPEGSVVMFGGVDTSYYRGTLNWV.[P]	4615.26022
9719	[P].PSLGQDALTLSSEKASQDSVTPSVVEENGEAKELHPCKYCKK.[V]	4617.24471
9720	[G].SGGAFRAPSIHGGSGGRGVSVSSARFVSSSSGGYGGGYAGALATSDGLLAG.[N]	4617.24652
9721	[D].MKPNLDTPTTDTVQLKEAEPQNTDFSEEFKKTENVFSETL.[F]	4618.20289

9722	[Y].SLTSGSMGQLPHTVSWPSPPLYPLSPSCGYRQHFPAPTAAPGAP.[Y]	4618.22821
9723	[A].MGVAGPASLYHSGLTVGMISGGGVVCVQEARAGYVGSLGCLHLGSLG.[N]	4618.26732
9724	[P].SPTPQPTSPQRSPSPLLGHSLGNSKMAQAFPSKMHSPPTIVRH.[V]	4618.34056
9725	[V].RQYYTLLNQAPDMLHRFYGKNSSYVHGGLDSNGKPADAVYG.[Q]	4619.21607
9726	[R].IRDPNQGGKDITEEIMSGARTASTPTPPQTGGGLEPQANGETPQV.[A]	4619.22781
9727	[M].LAAALRTGDDYIAIGADEEELGSQIEEAIYQEIRNTDMKYK.[N]	4619.24576
9728	[Q].SSPGLNPGQPSSMLSPRHRMSPGVAGSPRIPPSQFSPAGSLHSPVG.[V]	4619.29942
9729	[Q].SLGGPAAAYATGKASGAGGAGGQAYSPGQPQGLLGPQAYGQGFGGGQAQDL.[S]	4620.21382
9730	[E].GLGGGGVGKWGGLSWMGEGSGVAALSSPLSLPSSSTTQEICLSDSPAV.[M]	4620.22325
9731	[R].PGARGEHQAHTQAGVGGAPPLPSSGIGGRCHSRKSCPSPAEGPPGVP.[A]	4620.24437
9732	[M].GGGSVRFGSGGAFRAPSIHGGSGGRGVSVSSARFVSSSSGGYGGGYAGALA.[T]	4620.24752
9733	[K].APSPATLPATSSSLPSPATPSHGSPSSHGPSAPHPTSPTPPVTAGGATAAA.[N]	4620.2601
9734	[K].QGVQKPEVEKAAQSHRASENRFPSSDKPDSRGTQGPLNMEQV.[Q]	4620.26079
9735	[P].AAGGYFVPAVPQAQGRPPCYTPNQLAQMRPNPRWQQGGRPQG.[F]	4620.26365
9736	[S].KHIFEMESVRGQLQSMLQTSRDAAYPQAFEELFPRYTSL.[R]	4620.26499
9737	[A].LRGPPGPMGYTGRPGPLGQPGSPGMKGESGDLGPQGPRGPQGLMGPPG.[K]	4620.26568
9738	[L].PQGASWARPNLSIMPPSAPASASDDIPEAADVPPPVPAPPTPPPQE.[G]	4620.27152
9739	[L].QLASPGTDGVQGLQTLTMTNSGSAQQGTTILQYAQTSDGQQILVP.[S]	4620.27337
9740	[R].RQGSLSASECLYGAPPAPLGPGMAFGSPAFPPHTVMLHAGPPYTP.[Q]	4621.21012
9741	[L].GPEAKAKARTYHYLQVPQDDWGGYPTGGKDGEIPCRRMRSG.[S]	4621.22117
9742	[G].TSWGSPARGQGLPEDVTVHTQMAATSVLSQSSLPAVEGEEETALP.[K]	4621.23625
9743	[E].QSATPAGAVSIPEEPDAPAGAVPTPEELAAPAAELSAPEESDSPAVRA.[F]	4621.24278
9744	[A].GPGRWQDVSVGSWNQPPRLGRQMSDGVGEKLFQDLYPFMLG.[E]	4621.25035
9745	[A].GPGGQPSAFPPARSPGGSDPQIPLAEMEALSLTSEIVSELSCSLAL.[T]	4621.28003
9746	[S].PGFGLDPKTPMEMLYHHVHRLNMSGPFGGAVSAAGLTQMPAGNV.[F]	4622.21997
9747	[M].AMKVELARMSYQRAAWEVDSGHRNTYYASIAKAYAGDIANQ.[L]	4622.23034
9748	[G].TGASLFGQTNTGFGAVGSTLFGNNKLTTFGTSTTSAPSFGTTSGGLFG.[N]	4622.23215
9749	[G].LQPSGPPQFPPYRGMMPPFMYPPYLPFPPPYGPQGPYRYP.[T]	4622.23269
9750	[S].LGNVQLPQAPMGPRAASPMNHSVPMNSMGSVPGMAISPSRMPQPP.[N]	4622.23795
9751	[E].KTFDNSTVPHPGSITMGGSLLQSSAPVNIPGSLGSSASFHSASPSPP.[V]	4622.24676
9752	[G].QRVDGLAFVNEDVVASKGSGPGTICLWSWSQTWQGRGSQSTVA.[V]	4622.2481
9753	[G].EAQGPVEVPLLEAEVGEAASHLASYALGSSSANVEALPQESLDRM.[M]	4622.25666
9754	[Q].QSLGGGWAGGGRAGPGPPEHSVPPPLQDKDFGKHALRMEHVNPPA.[E]	4622.28582
9755	[A].TPAGAVSTPEEPATPAVSTPEEPATPEEPATPAGAVSTPEQSATPAGAV.[S]	4623.21081

9756	[S].HVPQQNGFTGVSETAEAQRMMPVNRTAKPFPGSGNQPAAPFSPS.[R]	4623.22559
9757	[H].LKMSSPQFSQAMPSRPMAPMSSAAVAGPMLPAGNAQQRTSGPAPAP.[P]	4623.23972
9758	[L].AAYHPHGIMGIGTVTNLCTEATGFSSIFPGIRPHLMTLNMFF.[Q]	4623.2444
9759	[L].QAAGVAADWAAAGLADGARAAGHAGHGPAHGGLAGHGAAAAGVAVETGLEAAS.[A]	4623.25842
9760	[A].LGSIAGLGMQNLNSVRQNGNPSMFGVGNTAAQPRGMQQPPAQPLS.[S]	4623.26132
9761	[S].GQQSRGAASHHSPLLAWTSYAGPTHPVLELCSTGAPSSLPGAAWL.[V]	4624.279
9762	[K].SLSSPSSRPSGEASVPPPPAVGRMYPPRSPKSAAPAPISASCPEPP.[I]	4624.29227
9763	[V].TPGSHIHSQTAGHHLPPPPPPPGPAPHHHPPPHPSSGLQGLQAQH.[Q]	4624.29944
9764	[A].APGGAAIESRARAIESKVIPEPSFFEEESAVRDAAGGPGAPADTSR.[P]	4624.30263
9765	[Y].YNPAAVNPMKFAEQEKKRKMLWQGKKEGDKSQSAEIWEK.[L]	4624.34391
9766	[H].APGTPGTPASLSANSSLSSSGELVEPSVDQTPQASPGLAPNTRGSPGPP.[P]	4625.26016
9767	[P].WREPGEAGPVPPESSPKDALKSMSLPSSQPQVSRDLWENAPPA.[T]	4625.27291
9768	[P].PQAVGDRQWLIQGSEPHPALGASTFWGRWAPPAMTANPGQALSA.[L]	4625.28951
9769	[A].AAAGKEGVPSGVCDLATSRPEVPEGPLWSWRSRTLGSAAGKAGAMA.[L]	4625.29875
9770	[S].NPVTVAAAMSMRSPVNVSSAVNITSPMNIGHPVTITSPLSMTSPL.[T]	4625.30818
9771	[S].QPPVTSGGVAPSLAQTSMTGGVVRSLGQASKVSGVEPSLAQSPMTTGV.[A]	4625.35493
9772	[T].LSSKGGSEGRPESSLANSSVAPPPPGGSGRGSPSGGSTAEASDTVSIRSG.[G]	4626.22623
9773	[N].PGWPGTPGAPGPKGDPGFQGMPGIGGSPGITGAKGDMGLPGVPGFQGQKG.[L]	4626.26566
9774	[I].FGTVVFAGYPPDIPQKLNCETYDLKEIICTWNPGRPTSLE.[G]	4626.26835
9775	[P].EQLPPASPATPEASDPSVSGHPALEQQPGQKTLSPDPDPLSRLLA.[T]	4626.3322
9776	[Q].GLMVASPAQTLNDTLDDIMAAVSGRASAMSNTPTHSIAASISQPQT.[P]	4627.24366
9777	[G].AAGRGRGAGMPYPTPAMQGATSSVLAETLTQVSPQMASHAGLNTAQA.[G]	4627.245
9778	[Q].GPSQNLPRDPSKSGSHGIFFKQQNPSSSFHGDQRPQNPHSFP.[P]	4629.21549
9779	[G].PGSPATLSPSAGVPQPVGMEALDQAEGPAASQRAMPPPPPASPPSEPA.[Q]	4629.22358
9780	[P].KHKQHEAMFDWLDNTVIKLCTMPPVGTDLNTVKDQLNEM.[K]	4629.22445
9781	[G].RQRRCGGGGAGSAAGGKMADEEKLPPGWEKRMSRSSGAAGVGAGTGP.[R]	4629.23282
9782	[G].RGGEAEPLDLSLRAGPGGEAGPGGALHRCLFCPFATGAPELMALH.[L]	4629.25478
9783	[L].SVPMSQAALGEIVPPGEDQVGHPSPTVHQDFVREHHLVMQSVA.[N]	4629.2613
9784	[Q].QGTLNPQNPMILSRAQLMPQGQMMVNPQSQNLGPSPQRMTPP.[K]	4629.26152
9785	[T].FLSRGPDDPKHQSSGTVWLKHQHDRVCGDTTLQLQENVKD.[K]	4629.26514
9786	[P].PGPKGQQGVTGSVGLPGPPGEPGFDGAPGQKGETGPFGPPGPRGFPGPPG.[P]	4629.29095
9787	[S].NAPSGSTRPSAASSRPSSRTQASEGAGDSDGDGESDAEEGGRPAAATAP.[T]	4630.05045
9788	[S].KNDERLHGSDLFWRGVGSNISRNSWEIRTNNLRMDCRK.[K]	4633.276
9789	[N].GQLQCRVLGGGGPGGGGGLGGPGGSVPFKLEENYDNFFTVVTDRPL.[D]	4633.28923

9790	[S].GPSLGPHFPLPGRGEVWGAGYRSHREPGPGAKEEAAGVSGPAGGRGGG.[Y]	4633.29441
9791	[S].QAPSYLPGEHLGVFPCNQPALVQGILERVVDGPDPHQPVCLE.[T]	4633.29663
9792	[G].PQPASGSPTPAPRRPGAAGPAPHPQWAGQPSVLDSINPDRHFTVN.[K]	4633.31956
9793	[R].AGRDTLCSLGRVAHRGMEKSLFVFNQDEVQASEVREGDLQL.[G]	4634.28383
9794	[D].GPSGDRRGDVYRCLVGGSHSAPCAKGHLGDHPLGNSSRPAVNMH.[L]	4636.19637
9795	[P].GAMLELGPPHGVSAEEAGLGPQMAGQPLEAEEDRFVAPQQALQGH.[V]	4636.2195
9796	[P].PRGPPFGSPMGHPGPMPPHGMRGPPPLMPPHGYTGPPRPPPYGY.[Q]	4636.21985
9797	[D].ATISIDSSNQKLDDLQQSNEQTSSQITGSMMQLLLNNPSLAAQ.[M]	4636.23527
9798	[G].GPTSHPSPAPTPSSAPSPLGGSALCGGKPEAGESPPPAPGTPKANGSQPP.[G]	4636.23726
9799	[Q].NKAAGSGPGKGVSATSSTSGLPDMTGSVYNKTQTFDKQGFHAGTPPP.[F]	4636.23726
9800	[W].PQSSAQPSSPASISTQWPKTKVSSGPESSATTGLEEGHLGSRSPAE.[G]	4636.23976
9801	[P].AEPLNLGSSDQDRAGSVQSSGDLKDVPEDPVLESKEILMNSRH.[E]	4636.24313
9802	[S].PLGDGPEPPSPAPEPAPSRAQAAEGPHLTPEASPDPVPEPPPPSVEA.[P]	4636.2478
9803	[R].SLACGKSSIIVAADESTISWGPSPTFGELGYGDHKPKSSTAAQEV.[K]	4636.25118
9804	[L].DLLPKLERSAARPSGEPGCSCAQPAAEAAAPGWAQARGHPGGELAA.[A]	4636.2532
9805	[V].SDKAPSPATLPATSSSLPSPATPSHGSPSSHGPSAPHPTSPTPPVTAGGA.[T]	4636.25502
9806	[F].QILTQEDWNKVLYNGMASTSSWAALYFIALMTFGNYVLFN.[L]	4636.25672
9807	[T].TAAVSSLPSRITGEQTGCTQLGDKAWLQPSGAEARPTDNTTTVY.[E]	4636.25839
9808	[L].GQVSQPEAEMSVRPHAPSHGQRKSLRERWLMDGAAEEPERP.[E]	4636.26443
9809	[P].GRDGQPGHKGERGYPGNAGPVGTAGAPGPQGPVGPTGKHGNRGEPGPAGA.[V]	4636.2649
9810	[T].GPGSLADLRALEDPPPGADPSALAAPSAGGLQLRKHMSAMHRFEQ.[L]	4636.31474
9811	[D].TISQSQMALEEVPEPLASSQGQSLPGSSREHMAQWEVRNQTL.[L]	4637.23588
9812	[N].SGVFAATTGPIQAAFDASVSVPSEGLPQGTSSAPQAPAHPTGASESIVS.[Q]	4637.26418
9813	[-].MLVPGACAVTSHPHPSPHPHRAPALSPGFAAAAGIGHPGAGGHARAM.[A]	4637.27244
9814	[Y].LEYRMVPNSDPSRAVASPAGSRASSTRAARDGTEGARHPEARPS.[A]	4637.27342
9815	[I].SIVGQSNDRGDGGIYIGSIMKGGAVAADGRIEPGDMLLQVNDVNF.[E]	4638.25628
9816	[Q].QRPSGYVHQQAPTYGHGLTSTQRFSHQTLQQTPMIGTMTPLG.[P]	4638.27287
9817	[Q].AHLASSPPSSQAPGALQECPPTLASGMTLAPVQGTAHVVKGGATTSSP.[V]	4638.29266
9818	[S].TGFMKAPASGAKSTPRMRAPASGAMSIPSSTAPISETVSVLQMTTP.[A]	4638.30343
9819	[G].QQDRSVAQFTGSQSMPQSSLYGMTSGITQIVAQPPPQATNGHAH.[I]	4639.20525
9820	[F].SPMSLPGASTASPGAAAYPSLTNRGSNFAPETGQTAGQFQTRTAEGV.[G]	4639.21177
9821	[D].PAPSLGPMAAGSQKHSHDSGVPPVVDERTGSEGATASPSLGHHSEIP.[R]	4639.223
9822	[F].PGTNLTGFLPFVDNQMRNLSQDHLYDLDINIFDEINLMSL.[A]	4639.24834
9823	[R].PGGSAGPGSPATLSPSAGVPQPVGMEALDQAEGPAASQRAMPPPPPASPP.[S]	4639.25555

9824	[P].PSPSSMNQRRLGPREVGGQGAGSAGGLEPVHPASLPDSSLAASAPLC.[C]	4639.27399
9825	[E].VRGYGEEGERKTLEGALPSEDLTDLKELTEESDLLSDIFGLD.[L]	4639.2785
9826	[P].AAGTATISQDTSHLTTGPVSGLASGSSVLNVVSMQTTTAPTSSASVPGH.[V]	4639.27918
9827	[P].APINLEDSLDGDLVHNSASSLEAMSKELAVLNSRASGSSEFPLPA.[P]	4639.2832
9828	[V].RASAQGKPGNRNSNSYGVPEPAHAYAQPQTMAPPPAAGAPGATVSPL.[P]	4639.28588
9829	[S].PREGRDYEGMLRSGGVAKVATPGFEEAGPRERPTSVFYQRAD.[M]	4639.28588
9830	[S].KANSSMLIGTTQMTLNGTREEKQQTWENGKSEKHVLFDSKS.[V]	4639.28791
9831	[V].AIRDGAYSLFDNSKLVEGFYPAPGLKTFLNMFVDSNQDARR.[R]	4639.30382
9832	[K].SHLIHGSQGVMGTSMSTPASKIIPQGADSTMLATKTVKHGAPGPSH.[P]	4640.30179
9833	[K].TSLQPMVSALNISMGGTGTFTVRMALFQSPAYTQPYQGSSVTLS.[T]	4641.26736
9834	[M].NGQYLYNFPITVEGHKKDSKEPLSQADHPHQLFADAPPPPSA.[P]	4641.27971
9835	[-].MNGQYLYNFPITVEGHKKDSKEPLSQADHPHQLFADAPPPPSA.[P]	4641.27971
9836	[A].AAEAAAQAAATEEAQALAIQAVLQAAQQAVMGTGEPMDTSEAAAAVTQ.[A]	4642.2247
9837	[A].SPNPMLATAAPAAPVHAQHALSFSNLHEMQPLAHGANTVLPSVSQ.[L]	4642.29294
9838	[A].FRGFGGPQALFIAENWMSEVAVTCGLPAEEVRRKNLYKEGD.[L]	4642.29696
9839	[S].QLGQPSIFDTQKGQTAGVRDPERPESAKAFGREGSGAQGEAEVR.[H]	4642.29928
9840	[H].APWQGLRDPPDSPDGSPLTPVPTQMPWLVASPEPPQSSPTPAFP.[L]	4645.27079
9841	[R].PSFSTRNAGIEAQDRRESLPTSPPWTPGASRPPSSLDGWVSPGP.[W]	4645.28184
9842	[R].HEAKRMIEGVIYEMRVYAVNAVGMSRPSPASQPFMPIGPPSE.[P]	4645.28224
9843	[A].QAGRQWGGALQGGTLWGLCPDPHPRGAPNILLACQSAPGQAGLGPG.[S]	4645.30518
9844	[E].PGRQGFPGVSGPPGKEGDHGERGPVGQPGPQGRQGPKGEQGPPGIPGP.[Q]	4645.31554
9845	[R].AVASVGTSALGRAGTSVRDTAGISVLVWDGPSAVAQAEHPASAMPGISA.[A]	4646.36313
9846	[C].RGGGVGGFLPAMKQIGNVAALPGIVHRSIGLPDVHSGYGFAIGNMAA.[F]	4646.4235
9847	[S].IKKVRLQTPSPKMVPALLLLPALAGLFGAAEGQAFHLGKCPNPP.[V]	4646.61935
9848	[T].KDVTTPGHSTPVPEGKNAMSLFSSTKTDVRQDNAAGRAGSSSLTQ.[V]	4647.27035
9849	[N].AGPQGHLGPQGPPGPQGHIGPQGPPGPQGHLGPQGPPGTPGMQGPPGPRG.[M]	4647.2923
9850	[S].YSSKSQRGLPGAARPHDSGTFIQQEVGKPEDESPGRFFKQID.[S]	4647.29749
9851	[G].EDVRVEGAFPVTMLPGDGVGPELMHAVKEVFKAASVPVEFQEH.[H]	4648.32144
9852	[V].SALLHGQQTLLQDLQEMRDAAAHMASRAQVFYLPVGTKHHF.[S]	4648.32999
9853	[V].SPGRSGSVGQRSVASALGPSQSQMSRGVAPLTSHASVAAAPLTRESAS.[R]	4648.36083
9854	[G].SPRTVGSLDPVEIAVRSSEQVASASLGPAGSLGQEGLVETVLAMEPG.[A]	4648.37744
9855	[G].TTATSSLPSFGQAPTSVSIPAGFNPSTGKNLKFTLSQPSCSGNLGH.[T]	4649.29404
9856	[F].RSRGGGGGGFHRRGGGGGRGGLHDFRSPPPGMGLNQNRGPMGPVPGQ.[D]	4649.29472
9857	[G].VTGESGAGKSSLINALRGLGAEDPDAALTGVVETTIEPSPYPHPQF.[P]	4649.33695

9858	[C].APGLGAAPQAADPADPLAEAWGAQHSPRPDPSPAGPPAQAWGLGWGW.[G]	4650.23377
9859	[S].LSSPSSRPSGEASVPPPPAVGRMYPPRSPKSAAPAPISASCPEPPI.[G]	4650.34431
9860	[R].SIATGEITEADVSSRKGDEIPLTAVKTEASPESMLSPSHVVSNPI.[E]	4650.34547
9861	[R].AFYELSQTHRAFGDVFSVIGVREPQPAASEAFVKFADAHRSI.[E]	4651.34807
9862	[G].PQQAIGEMAVPPSVPSSQKDYLSNKPFKSHRNGSPGYKLLGAAV.[P]	4651.40895
9863	[F].RCNLPPTTGNVTNDVGTKTNLVTLNPSIIKLRYGNLKEKKAI.[F]	4651.5716
9864	[R].DPLAEGAEGLGRASPEPSALPTQRTPSDMAGPEPEGSQNSLQAAPPA.[T]	4652.21692
9865	[K].FRIPVDTMVTYMLTLEDHYHPDVAYHNSLHAADVLQSTHV.[L]	4652.23369
9866	[P].GPQGNAGPQGHLGPQGPPGPQGHIGPQGPPGPQGHLGPQGPPGTPGMQGP.[P]	4652.23485
9867	[E].PGPRGGFAGGGGEGTTNVNSVRERWPSRGGGERYTRGWKNYSGK.[M]	4652.27507
9868	[L].QTLQTEAPGLVPSLGSFGMARPPASSAGSNAGSAPEAPTSSPALPVTSS.[P]	4652.27845
9869	[R].IPDPGGPEMTKTRSASTSSPLQHPRPRMTPQNRGSQEPRPEGA.[A]	4652.28048
9870	[P].PGMFPLWPPMGPFPPVPPPPGSGEAAAPPSTSAAALSRPSGAATTTAAA.[A]	4652.29523
9871	[A].EELVKTMDLPKGLQGVGPGCTDETLLSAIASALHTSTTPITGQLS.[A]	4653.36376
9872	[L].GVSADVAQTPGWPGDGQGDPSPPGKEPGFARGGPFFGLHASPGPKAAQ.[A]	4654.24981
9873	[A].VSTPEQSATPAGAVPTPEQSATLAGAVSTPEEPATPAGAVSTPEEPATP.[A]	4654.25301
9874	[Q].QQPPNQGPQSLHPGLGGMPKRLPPGFSAGQANPNFMQGQVPSTTA.[T]	4654.26779
9875	[T].TQTSQPTSYTVAPASQPGMAPSQPGAYQPRPGFTPPPGSTMSPLPS.[G]	4655.2181
9876	[Q].GPGKDRDAAGGGASGGRDREEPRGRGLEGRGLEGQAWRFGLTEGC.[G]	4655.2377
9877	[T].WELPGSPEGAGKSPAARLEEEEALQRSGPHTQDAPGKDVPLSCT.[I]	4655.24307
9878	[S].RSYTSGPGSRISSSAFSRVGSGSSFRGGLGTGMGVAGSYGGAPGLGGITA.[V]	4655.26554
9879	[P].VPLSSTCISPSSKSVPAHGTTLNAQPAASGAVDPVSSMQSRQVSSSS.[S]	4655.26757
9880	[S].APASPSSASKEVGIGFAQGPGASASTAATPGPAGLPRGYMAPTSPAASER.[S]	4655.27946
9881	[E].STVVPHGDAPGLWGGPEQIPTPPTSPSSGSRSGTYTKVSEPQQALP.[P]	4655.30124
9882	[P].REKPPNKSSMRIVVDSESRKRTIGSGEPGVPTKKTWFDKPN.[F]	4655.44747
9883	[R].PGLAAMGTQPATHHPIPTYPSYVPAAAHDYGSGLFHPGGFLGGPASS.[F]	4657.23574
9884	[E].QKQTAAVALCQLAACSPVNVEPAAQEPTCRPDAPSPRAPGSQEV.[Q]	4657.25557
9885	[L].SSPSGEISATGVGVSAEGEMSGVVTVRVYSPHGRLSEPVSPDAALGSS.[S]	4657.26862
9886	[T].GLGSPEAPHPVPGGGEGPPKTGTAPSPGPPCPPVDGTSEGKGARHPKPS.[T]	4657.28521
9887	[A].AIYTARTAMDATEVRGPGREASCPSMSHSSSSGPFPLTGPGDAFV.[R]	4658.13445
9888	[E].MPRSELGSVDGPLPRSRWASEASGKPSASDPESGAAPTVNSSSRSS.[S]	4658.21356
9889	[A].NAVPAAVYHHHHHHPYVHPQAPVAAAAPDGRYMRSWLEPTPG.[A]	4658.25479
9890	[E].VAAATTMEATSREAAPAKSSASGPSAPPALFELCGRAVSAHMGVLES.[G]	4658.26473
9891	[R].SLNPLGNNPMNIPAGGITTDQQPPNLISESALPTSLGATNPLMNDG.[S]	4658.27126

9892	[P].QGSSSRVATTPGLNPMTPVHKGASPYGTPVTPRMNLNSNFGMATI.[P]	4658.29122
9893	[T].ETQQGLMVASPAQTLNDTLDDIMAAVSGRASAMSNTPTHSIAASIS.[Q]	4659.23349
9894	[K].ERGEKGEPGARGATGAKGESGVDGLMGPPGPQGPPGDPGPPGTPGLDGKP.[P]	4659.24922
9895	[P].GSGEAAAPPSTSAAALSRPSGAATTTAAAAASAPAPGPASSPEASPAPGFPFP.[P]	4659.25977
9896	[D].AAGVRGLQSGDGASSGQQEGRPFQGGRWKEPRDGAQGGGPGGAGTPPR.[T]	4659.26563
9897	[I].VPGARYAMAGSFLQDQFVSNYAKARFHPGAGAGPGPGTDRSVPHT.[N]	4659.26984
9898	[M].SSFQPKRKEEEAGFTGRRMNSKMQVYSGSKCAYLPKMMTL.[H]	4659.27611
9899	[P].KQPNAHRGTRPDRPDTQTLLYQGSEAEAAAMTVATCVKCKSV.[H]	4659.28245
9900	[-].MAQKHPGEGGLCGAHHSGGASLRSLGHSVDPDILSFSGLRDSARPAP.[N]	4659.29817
9901	[M].AQKHPGEGGLCGAHHSGGASLRSLGHSVDPDILSFSGLRDSARPAP.[N]	4659.29818
9902	[E].ARSPKVGAQEEEVEFPGQGKAPSELPTAPGENVGRANGGQGVESAVA.[E]	4659.30336
9903	[T].GWPLGLLEERGDGAMLGGVAPVGVVGEEPWEASLDRDFHLDALL.[S]	4659.3188
9904	[A].PGFVSFAGRGDIQPQLDSALQDVNDKYLLLEETEKQAVRKAL.[I]	4659.44169
9905	[I].STPIMATSGVGLGEPGMLSPTSTLAPALMPAPAPALAPIPIPPQSPKL.[L]	4659.485
9906	[L].CLIGTATIGMIYTLCVYVLSGETPGEVVKKALDVITIAVPPALP.[A]	4659.51016
9907	[L].PSGSEMRIGLTEEFCVGKSELRGYGPGIRVDEVSFHSSVYLD.[E]	4660.24465
9908	[P].AVEESTVPTTQSCATPAAKAVATPEPALAQPDSTAPGGATGQAPPSSKG.[E]	4660.26828
9909	[Q].GPAGHPGEAGLPGPSGNMGPQGPKGIPGNPGLPGPKGEMGPVGPAGNPGAKG.[E]	4660.27835
9910	[V].SNPNASPSLPESLSSLAATPVGGSSPGSVDVASSPGVERPGSGVSTAPLE.[I]	4660.28604
9911	[K].GPNGRDPSLDVYDVPPSVEKGLPPASHHAVYDVPPSVSKDVPDGP.[L]	4660.29542
9912	[G].AGLMGLAGGVVGAGMAAAALAAEAGMVAAGAAVGATGAAVVGGGVGAGLAATVGCM.[E]	4660.30238
9913	[S].PGLPTSGLPNKPSSASLSSPTPAQATMAMAPQPAPQPQQQPQVQP.[P]	4660.3134
9914	[E].PEVPGAASAELGTSEGSVQQPLLELGPGEYRVVLCVDVGETKGAGH.[R]	4660.31992
9915	[L].QLGYVDIVFANRLDPNSPMEEIVRAMTYVINQGLALYWGTS.[R]	4660.32144
9916	[I].RQFHFHGWPEVGIPSDGKGMISIIAAVQKQQQSGNHPITVH.[C]	4660.37424
9917	[E].GPMANAAAPTSTPQKLIPPQPTGRPSPAPPAVPPAASPVMPPQTQSPG.[Q]	4660.40143
9918	[A].AVGNINGLGSLGLFPRLYPLYGGLLGGGGLPPPMLAPASLPSSLPSEG.[A]	4660.48475
9919	[P].GPPGPSGPLGHPGLPGPMGPPGDPGIQGYHGRKGERGMPGMPGKHGAK.[G]	4661.28233
9920	[P].LPMPDSKSTSTAPDGAALTPPSPSFAATGASSANRFVSIGPRDGNFL.[N]	4661.29404
9921	[S].DFDRLRTALPASGSSTGELELLAGEVPARSPGAFDMSGVGGSLAEAV.[G]	4661.31517
9922	[Q].KAKEAILEMDSYYPNRYRHSPERLKKGSQDDVFTHMHQG.[R]	4662.27287
9923	[K].RQQAGPPAGGMPPAPQAAQLAGQKQSQQQYDPSTGPPVQNAASLHT.[P]	4662.28661
9924	[S].HRDELLAPSMEGALPPDLGIPLDATEDQQATAAIIPPEPSFLEP.[L]	4662.32836
9925	[S].LEPSSSTEPAQSNLSVTAKIKAIEAKLKMMAENPDAEYPAAPVH.[S]	4664.35862

9926	[H].REEQAGKDVTLRDGPRSTPGAQAAPSTARSPQDPAPPELGSVPPSS.[V]	4665.32516
9927	[T].ADPGGSLGKSTNTMKETSKIETYIAKPALPGTSTNSNVAPLCQIT.[V]	4665.33861
9928	[V].GPEAGMQKVRAWGPGLHGGIVGRSADFVVESIGSEVGSLGFAIEGPS.[Q]	4665.35183
9929	[S].ASTVGAVTPSSVAGEPMETSPVIHLVTDAKGTVIHEVHVQMQELP.[L]	4665.35387
9930	[Q].RNHSPVPATLLTNSPTPASPRSIPGSSSQHSSSQLAQSPSLRSSR.[E]	4665.38401
9931	[D].TWNRLDFFIVMAGYGPSPEGQPGATPVPAGLGAWHRQHASSKC.[S]	4667.24593
9932	[S].PGAGGGVNERRRHAHSAPSAHPGMAAAQGPVAPSSPEQNGAVPSEATK.[K]	4667.26285
9933	[S].EKAALDSQEPSSSVKEDGDSIFLSLGENNCEEVTLMPSESNPI.[E]	4669.12913
9934	[V].LSAASLLQMADIAASCQELLDARSLGPPGPGAVALAQPAAGCTPAAPP.[Y]	4669.34226
9935	[G].AGGVDNLLVLDLQKYKASTHTVAASGGGGVNMGSHQKWKVGEIEF.[E]	4669.38313
9936	[Q].PPTLASTPMQNGGLRDSSQVPRTLEGNPGASAEPMLGAGGRGPSPGPA.[R]	4670.26857
9937	[E].GPLGEGGASEAPRELAGQSQRRGGGAESGWVPGTSAPRGTAPQEPSRG.[P]	4670.28028
9938	[P].ASPSSASKEVGIGFAQGPGASASTAATPGPAGLPRGYMAPTSPAASERSP.[S]	4671.27437
9939	[L].QMAVQGKRPMPGMQPQMPALPPPSVSATGPGPSPGPAPPNYSRPHG.[M]	4671.28397
9940	[S].GPSGPPGPKGDDGIPGQPGLSGPPGPKGEPGHPGTDGAAGQRGPPGLKGEQ.[G]	4671.29347
9941	[K].IHRSATDADVVNSGWLVVGKDDIDNSKPGPGPSQPGPSPLVNQYS.[L]	4671.30739
9942	[S].PQNHALQPHHHIPMVPAQQPMVPQQPMVPVPGQHSMTPIQPN.[L]	4671.31046
9943	[P].AVLPGEDPPPYSPLTSPDSGSAPMITCRVCQSLINVEGKMHQH.[V]	4673.22551
9944	[A].AKERESAEQSSGPGPSLRPQNSTTWRDGGGRGPDELEGPDSKLH.[H]	4673.23232
9945	[P].GPPGPMGPPGLPGPMGIPGSPGHMGPPGPTGPKGTSGHPGEKGERGLQGE.[P]	4673.24461
9946	[L].TRLGLEACGLTSEGCKALSAALTCSQHLASLNLMHNDLGLRAM.[T]	4673.27248
9947	[V].TAGPLDREAKSSYDIMVTASDAGSPPLSTHRTIFLNISDVNDNP.[P]	4673.27879
9948	[T].LQEGTGANLTCRVSREAGGPANFTWLRDGALWAQGPLETLTLM.[P]	4674.31915
9949	[W].DGHLTPPEVASLADRASRARDSNMVRAAAELALSCLPHAHALNP.[N]	4674.3376
9950	[V].FLQMPTTLTAGGPGERALPSQAAERWLPRPEQGLCPVEEDRL.[E]	4674.35554
9951	[K].MGKNQTAVREEMISLANYLDSIWTNENLISMAGGAGDVLSNFV.[Q]	4675.24769
9952	[A].SVSVPSEGLPQGTSSAPQAPAHPTGASESIVSQAEEKAVATPTPTMMG.[R]	4675.25019
9953	[N].WARDAMSLSGRRGSKGHGPGMARKFSAPGQLCVSMTSNLGGSAPGS.[A]	4675.26094
9954	[M].HVSTPMQVEIESEALQPSAEAVAANPGAMLELGPPHGVSAEEAGLGP.[Q]	4675.26545
9955	[V].PTTNLNHSVGKGSSCGLPLMNSFNLKDMAPGLGSETRLDRSKGD.[T]	4675.26613
9956	[A].VQGQAGSPDKGKHGKQRAEYMRIQAQQQATKPSKEMSGSNETS.[S]	4675.26997
9957	[P].KEVSPVFTQFLECVWHLTEQFPQAFEFNEAFLLQIHEH.[I]	4675.27548
9958	[S].PGAKGEQGPAGHPGEAGLPGPSGNMGPQGPKGIPGNPGLPGPKGEMGPVGP.[A]	4675.27802
9959	[I].NLHNFSNSVLETLNEQRNRGHFCDVTVRIHGSMLRAHRC.[V]	4675.28004

9960	[M].TPFGGSSAVTPFGGASSSFSVVPSSYPAGLTGGVTIFVALYDYEART.[T]	4675.28788
9961	[P].TQSAPAGGWRGAARAGPTPGQAALEIQECHHCLALLHPFGHLFS.[M]	4675.29461
9962	[S].VQGKNVNVTEAQLNEMNILDINCVVNEFNTAQVTVLRDVEE.[K]	4675.29781
9963	[E].QGGPGMRRGRSSGTGVGGGVEATGPILMSPHLHPSEYMAQLERQL.[Q]	4675.30385
9964	[H].GPAHPAFSIGSPSRYMAHHPVITNGAYNSLLRMFPFLSFNISG.[L]	4675.31255
9965	[R].LSKRTPVFYNYMYAPEDAEVSRAPGEVAGLLHAPCSRQAVPQ.[V]	4675.31843
9966	[P].AAASSPATAPAPAPAPASASAPAPVPAPAPAPAPSPAPASSSDPAAAATAAPGQT.[P]	4675.32745
9967	[S].GLGAGPGPSVGMGVVPDPFVGREVTSAKGDDAYLYILLIMVFYAC.[L]	4675.3285
9968	[V].GAPGLPGPAGPKGDPGSRGPMGMRGPPGLQGPPGSPGQAGAVGIPGERGPPG.[P]	4675.33687
9969	[P].PQPSLLAPAAPYTPMLPATFPPQPDSVYLVPTPSKTQQGLYQAP.[G]	4675.41566
9970	[R].PASASPAPNATADGSKTSRASVDTTPSVIQHRAMMRFSELEMKE.[R]	4676.25015
9971	[L].IGNGTQPSTSCSLGGPMPLLPNRSDLSGVDITMLNMLNRRDSST.[S]	4676.25352
9972	[G].PQGNAGPQGHLGPQGPPGPQGHIGPQGPPGPQGHLGPQGPPGTPGMQGPP.[G]	4676.27123
9973	[E].KPEDVEAFKNYTLDSSAAPAPQAAPAPTPAAAAPTPAPSAQAPGSSYP.[T]	4676.27911
9974	[L].LRDTGKEGNAEQRKDAPPREAEAPGGDQGGGGGGLSPGPSAKPEHLQ.[E]	4676.27961
9975	[Y].GKAWAAGDIVSCLIDLDEGTLSFSLNGVSLGTAFENLSRGLGMAY.[F]	4676.3011
9976	[S].APLSPSPMGSSPRLPGPPPRPSCPASTPPTKDSLGPSYPAGSPNVAAA.[C]	4676.32357
9977	[I].PSDADKIPFHPNYTIKDILGILLLVLFLMLLVLFAPDLLGDP.[D]	4676.60676
9978	[V].GPSTSLPAGHSDIHSLLGPSHNAPIGSLNSNYGGSSLVTSSRSASMVG.[T]	4677.25978
9979	[P].PGSLENQTFTDVPKTESPEPTLSPTPMPMVITTAVTFGSNVTIL.[E]	4677.32017
9980	[Q].QPVRSMGPLSMEQLSVNQGNGQSAGYILYETVVTGGGVLNSDGHV.[K]	4678.25119
9981	[H].LGPGDRQMFLTVYLSNNEQHFTEVPVTPETICRDVVDLCK.[E]	4678.27384
9982	[Y].GPPPTSAQVTAQLAGMQISGAAAPAPAPSGLGYGPPTSLASASGSFPNSGL.[Y]	4678.30936
9983	[A].AVQQSTSQQATQGPSGQTPQLFHSQTLTTAPLPGTTPLYPSPMTP.[M]	4678.30936
9984	[A].ALVAAHSAYLSPMATMAAVQMQHMAAINANGLIATPITPSSGTSTPP.[A]	4678.3136
9985	[G].KPGPQGYPGIGKPGMPGMPGKPGAMGMPGAKGEIGPKGEIGPMGIPGPQ.[G]	4679.34273
9986	[R].LPSRGEDHLETSASGVGDLSGLPSGREGLEISASGAGDLSGLISGKED.[L]	4680.2871
9987	[L].FRFRLDGTSGGYWEQVMPAGGRPPAATGHSMVFHAPSRALLVH.[G]	4680.32518
9988	[Q].GPAGPPGPPGPMGPPGLPGPMGIPGSPGHMGPPGPTGPKGTSGHPGEKGER.[G]	4681.24969
9989	[Q].KCVSGGGGRAGRARGTAMATTGALGNYYVDSFLLGADAADELGAGRY.[A]	4681.26343
9990	[I].QGPPGPPGPPGPSGPLGHPGLPGPMGPPGDPGIQGYHGRKGERGMPGMP.[G]	4681.27618
9991	[P].QREPQRNFYPAASTQQATSGALFTQTPSGQSSATYSQFNQTSL.[S]	4682.21421
9992	[G].PGLGAGVALPGTGVGPLLSGHDGGPGLPGYPSPGETAAGKERAQETTAFL.[D]	4682.38491
9993	[G].KEGQDPRGPDDGPSEKRCPAVLACIPDNTQQPKAAPAACEPSAP.[R]	4683.19845

9994	[L].KAQGGPSRSTASSSMATTANPSKQNQSAPAAAPFTSSSAANGLESSVAT.[D]	4683.21871
9995	[D].SPGDRPPNSLAPGAEDSLDSPTARPLSTECPALDAALVQHLYHC.[S]	4683.22023
9996	[E].AGLNYHGHRFGQLFQGPGRDSAMDLSSLKHSYSLGFADGRYLG.[Q]	4683.26984
9997	[Q].NKGFGFGTGFGTTTGTSTGLGTGLGTGLGFGGFNTQQQQQQTTLGGL.[F]	4683.271
9998	[P].MQRPSTLPASAAGYQLRVGQFGQHYQSSATAAAASFPSPQRFSQ.[S]	4683.29096
9999	[G].PQGEPGPPGQQGTPGTQGLPGPQGAIGPHGEKGPRGKPGLPGMPGSDGPP.[G]	4683.31209
10000	[A].PASAAGTVPASQPAKSWASLFHDSKPSSSSSPVVSVETKYSPPATSP.[L]	4683.3213
10001	[L].ELFSIDPQSGLIRTEAALDRESMDRHYLRVTAQDHGSPRLS.[A]	4683.33322
10002	[V].PDSRFPFLGVHFTPRMDGNIWLGPNAVLAFKREGYRPFDF.[S]	4683.35065
10003	[P].NRPTPSDLAIVMYTSGSTGRPKGVMMHHSNLIAGMTGQCERIP.[G]	4684.26733
10004	[L].ESYIYQKMKTKEAERHPVQASGYVRQSQEQKLFVGFQEH.[H]	4684.33652
10005	[A].ASEPLANPDYAFHVIFLGDANVGKTSFLHLLHQNSFATGLTATV.[G]	4684.38345
10006	[P].QPAPANSVFPSPLPNIGTTAEDLNPLSALAQQRKSKPPNVTAFEA.[K]	4684.43694
10007	[P].DLQRTAMPGTMVKQTSPVMSDGTPGQAENPAISDCSCSKSDGSGP.[T]	4685.05283
10008	[E].RTMSNSIHCEDSFTLDETSMEFRSPEPFAKNISFCADRR.[Y]	4685.0912
10009	[F].GPSPGVEPVASMTSVASHPALGASSSSLPPLGPAAMNMVGSLGVPPSATQ.[A]	4685.28955
10010	[E].AGHTTALQLSSDYLRSSSPQSGPGPSGALNPRSETRSRTDTSRQA.[S]	4685.30107
10011	[L].RSDSISPFSASTVRPEDDVTTGVKNEKTTLGVEDYLSTPAASKE.[P]	4685.30644
10012	[G].KDDYINASRVEGLSPYCPPLVATQAPLPGTAADFWLMVHEQK.[V]	4685.31669
10013	[Q].QNVLSGHSQQTSLPSQTQSALTAPLYNTMVISQPTAGSMVQIPSS.[M]	4685.31854
10014	[R].GALWGAEDISRLDEAGGLQSLRVSMATHPDGFRLEGPLATMRST.[G]	4685.31988
10015	[S].KSASGTASPESPLEIQNPYLPVEPEKEVVPLVPEPASPEEAGSAGT.[E]	4685.33562
10016	[A].VGAGLMGLAGGVVGAGMAAAALAAEAGMVAAGAAVGATGAAVVGGGVGAGLAATVGC.[M]	4685.35178
10017	[T].QGVDLRGPPGPPGPPGPPGEGLPGPPGPPGSLLTSSGPPGPPGPKGDQGPPG.[P]	4685.37468
10018	[S].PLAESYLALVTEGSGSEVAGNRSSAVVSSSKLQGSSLTSISERYDP.[D]	4686.33808
10019	[G].QGRAGAMGSRTPGSPLHAVQLRRGARRGPRLLPLLPLLLLLLLP.[P]	4688.80897
10020	[R].RGSFVNSSGVMNQGVAPMVGTPAPGGSPYGQQVGVLGPPGQQAPPPYP.[G]	4689.29769
10021	[S].NPLTGMIEGSQEYNEGLVKRLHKVLRPFLLRRVKVDVEKQ.[M]	4689.61374
10022	[T].PPSNISGSPGDVGAPGIFGLEGYRGPPGPPGPAALPGSKGDEGSPGTPGNP.[G]	4690.28084
10023	[S].GETRGPAEEQPRPHTAAPSPGGPARAPPYREPPWGGPTTAPYSLE.[T]	4690.28217
10024	[D].SVAKNRDLQESISSAISALDDPPLAGPKDASTPDGPPLAAEAAVPGPP.[P]	4690.38463
10025	[A].TSGDSQLKPLQDEGQSAVPPLMTSPEAVMAMGQKHSLPADEDSVL.[E]	4692.24775
10026	[V].VMEALAAGADGTGPEVSLGLAEDDLMLPDDVVQYIKARTSGNLEE.[S]	4692.25427
10027	[L].VQNEPPAATELNVGNVQTTSVQTTSSPQDIMGTTEVSSARSLPSY.[P]	4692.25811

10028	[E].LNEGLASPAQRSQTSDSKTPRGPFQGSIEQLASPAQNEMSDFAK.[F]	4692.25945
10029	[P].QPSGSNRRGWNTTSQRYSNVIQPSSFSKPTPWGGSRDQEKPS.[F]	4692.26865
10030	[G].ARTTEFNSAFPMAPPAEPSAVPVSQNSDVRKHGGPRDSKDVKDV.[R]	4692.32232
10031	[F].NIAAMLPGEMRITTVAGTKPYMGAGAEAELIKLADAQDSDVVREG.[A]	4692.33175
10032	[A].RAASGSSDPFLCPPRQLEGLPRTPMRPAQVEKASQEALPMDGK.[R]	4692.34432
10033	[I].QGQEAKSSCVSLLSPSPSGDIRFSFLLRGYMVPMPTTGILSCS.[V]	4693.27688
10034	[Y].LESPKESSEPTGSPAPVIQHSSATAPSNGLSVRSAAEAVATSVLTQM.[A]	4693.32613
10035	[P].YYTGVPGLPSTFQYGPAVFPVAPTSSKQHGVNVSVNASATPFQQP.[S]	4693.33617
10036	[F].ANSVAAAPSARDKPASSMSDDEMPVLVRMTLSPPHSPHGATPNPPA.[A]	4694.23958
10037	[N].ESDVQPAKEQPVQAMFDHSPVGVGSKGVIPMNAKDLEEALEMGI.[D]	4694.27865
10038	[S].ASLPVGAPGLMGAMSSGTAGSATPDTPTLAASGAGDSAVVGAASVPVPPASIM.[E]	4694.29978
10039	[V].VDQLETMRRENKNLQEEISDLTMQIAETNKNLQEMEKTK.[K]	4694.30699
10040	[Q].QASAQGPQMQGSAQKAIPPGASPVSSLSQASSQALAVAQASSGASGQSLN.[L]	4694.30746
10041	[R].NHLEASAPSTVSPDALSPGPVPSPPPPEKESPQEVAEEVRPELEP.[Q]	4694.3108
10042	[L].GPTAKFFKEHDFFHLDPNNVIMFEQRMLPAVTFDGRAILE.[R]	4694.33228
10043	[P].SPATSSPVPPMASGGFLGFLEANMFSVIIPICLVLLLLALIVPLL.[F]	4694.60294
10044	[G].SEGPSVSGGPNNMPSHLVVSQNQLMMTGPKPGPSPLSATQGATPQQP.[P]	4695.25998
10045	[L].LHGYEEEGIVVHGVGGQQDTLAAGEAVAGVVDVAGGQVGDGDPALDAVP.[L]	4695.2809
10046	[A].QAASYRAQPSVSLGAPYRGQLASPSSQSAAASSLGPYGGAQPSASALSS.[Y]	4695.30336
10047	[S].PGQGLTARREHVYGMFRGGDRSGSLSSTAGGRSGGGHTLHAGSEGVK.[L]	4695.30539
10048	[E].FVEVEPEAKQEILENKDVVVQHVHFDGLGRTKDDIIMCEI.[K]	4695.3433
10049	[M].QEYTQGVRPFGVSLLICGWNEGRPYLFQSDPSGAYFAWKAT.[A]	4696.27179
10050	[S].HDLQISSGVTQDVWLNSPVGNSTLSHTGGTVSHQTGFGTNIPNVH.[A]	4696.27748
10051	[A].TSPSSTTMTTLFPVKPDTSTSFLETHPTNQTAAPGSISTGTTPIST.[V]	4696.2822
10052	[V].PNKSGSNVMVGTLCRKRMCGGRGVGPGQTLRQEEDRHGGGQKQ.[G]	4696.28962
10053	[E].RLTSGFGYSLAVADLNNDGWTDLVVGAPYFFERQEELGGAVYV.[Y]	4696.29945
10054	[G].PRMPSMTGPLLPGQSFGGPPVSQPNHVSSPPPQALPPGTQMTGPPGP.[P]	4696.3109
10055	[G].RHSPFGSQQFYAQREVIFQAPVSGVGKAGDSSQAEGSAGTQTSIK.[H]	4696.31387
10056	[Y].SSLPNGLGGPSEHLATLFRGPADTGLPNQGDIWSSPREVSSHAQR.[I]	4696.3251
10057	[S].PAVTSMPPTSSGVREASLTSAMTKVMTGTGVPQSIQAQGPSSPSSPPV.[E]	4696.32667
10058	[N].SAPVPGIKIKKKKKVLSPTAAKPSPFEGKTSTEPSTAKPSSPEPAP.[P]	4696.62877
10059	[V].LSPPGEYTALEPGFCAAVPEVACEEEANADDADLGADFLSPGMDV.[A]	4697.0528
10060	[K].AVGGEDVSVTCTVFQTQPVIPQPQPDGAEAGAPSAVPEAATPAPSAAGP.[P]	4697.26755
10061	[W].ESENFFFDLEPLPGAVEAVKQMANLERVHGGPELGWGIWSEP.[R]	4698.26095

10062	[H].SPVPCEAVASSDTSSRVSPDRQRLQAAGGMRCTVMAAAAQISGHP.[E]	4699.2192
10063	[R].RVSADELGGSAALMSLFVLSSKDGWVNIMYDGLDAVGVDQQTRL.[P]	4700.33347
10064	[A].RGCVLSQELFQQEAEKAAATQLGLNEPPSHCLVHLHKSAAQF.[L]	4700.34604
10065	[F].GPSPGVEPVASMTSVASHPALGASSSSLPPLGPAAMNMVGSLGVPPSATQ.[A]	4701.28447
10066	[L].NIHTMANMSRVFKKTCSNGKLSMYLGKRDFVDHVDMVEPI.[V]	4701.28667
10067	[S].AKGSCGDLPGLRAAVPPHEPGGPGSPVGLGGGPSAWEACPPALRGLHH.[D]	4701.33139
10068	[V].DEGVLASPGSQSKEGSPVSKMSVSRSSSLRSSSSLSSQGSVASSIGSQ.[T]	4702.2708
10069	[S].VAGYYPLDVSVTWTREEQGGSPAPVSGASLSSLRQSPAGTYSISSS.[L]	4702.29073
10070	[R].GEGTQEVPVAPSQIGAVAEGREEAGLPVSADVGVDSGTSPSSSLPSQVP.[F]	4702.29661
10071	[K].TQIFMNGACSPSLLPALPTPMPFPLPAVPDYSSSTYLFQLMAV.[V]	4702.29918
10072	[H].KILDMCAAPGSKTTQLIEMLHADMNVPFPEGFVIANDVDNKR.[C]	4702.3136
10073	[P].APASASAPAPVPAPAPAPAPSPAPASSSDPAAAATAAPGQTPASAPAPAQTPAQ.[A]	4702.33835
10074	[K].DGKRSSFKSPGQDQSWMVLGRSEVSDPSSETMDSGPGWSGEAVE.[P]	4703.08967
10075	[S].QPGQPGAGTVLAGASGLQQVQMAGAPSQQQPMLSGVQMAQAGQPGKMP.[S]	4703.27967
10076	[V].GVSAEGEMSGVVTVRVYSPHGRLSEPVSPDAALGSSSVAQVMPSSVT.[A]	4703.29272
10077	[A].TSTVTSVSTASTSTDSAPKPAFSFGVSSVTSTLSSVTSTTASTSQPFL.[F]	4703.29454
10078	[Q].QAQLSSSQGELPSMQLQPSWQGPPAALQGQPGAPLAGANFPMGSAKS.[L]	4703.29808
10079	[I].TGPKGGPPGIDGKDGTPGMPGVKGSAGQAGRPGNPGHQGLAGVPGMPGTKGG.[P]	4703.31653
10080	[D].LPRNTSTEPSTSGTSVAPENSGRTTLNSRPASQDPHEGSTVLPALS.[L]	4704.30957
10081	[V].QKEELSPAVTQVLWERATEKVPCSPLERCSSIMLLGMMARG.[K]	4704.34385
10082	[V].RSLSTYSAAALQSDLEDSLYKAAGGGPLYGDGYGFRLPPSSPQKL.[A]	4704.35802
10083	[T].GAGTHSAPKGSDPWAAPQQPAPSAGKAADPWAAASAAKPVSSSGSFDLF.[S]	4705.27061
10084	[R].AVISAMGQDIDFNLHLPSSPAPSGTAPSGPAASRAHLTPPCSPGAPAE.[A]	4705.27735
10085	[S].PSSPVPTPSPSTPAPPTSSPSSPAPPNPSPSNPTPASFAPVPAPEASPPA.[P]	4705.29442
10086	[S].RKESCNRESKMDPKVDSPKVDKMDSKTDKTPDGFAVPEPPK.[R]	4706.28586
10087	[T].PGVAHPAFSFTPTYMFARTAHTVSAHPAMQGNTGAASGLLSTTHLP.[R]	4706.30311
10088	[I].MTILLEELNASGRCTLPIDESNTIHLKVIEQRPDPPVAQEY.[D]	4706.38042
10089	[R].CGRPPSPSGLEGGGGPAIPPAIGPPRPPGSEQTAPAVAVTTLEVPPESP.[G]	4706.38828
10090	[H].AAAAAATAAAHSFPLSFAGAFPMLPPNAAAAVAAATAISPPLSVSATSSPQ.[Q]	4706.3923
10091	[E].KDPGVQVEGLTVQYLGRAPELLGGAEPGTYLTGTINGDPESVASLH.[W]	4706.3948
10092	[P].GLAAMGTQPATHHPIPTYPSYVPAAAHDYGSGLFHPGGFLGGPASSF.[T]	4707.25139
10093	[D].QGLSGFPGSPGEKGEKGSTGIPGMPGSPGPKGSPGSVGYPGSPGLPGEKGD.[K]	4707.26314
10094	[T].PAGAVSTPEQSATPAGAVSTPEQSATPAGAVSIPEEPDAPAGAVPTPEEL.[A]	4707.27956
10095	[E].PGNKEPLADTSSNQQKNFKMQSATFPIAADVKDAKAAQSNENLS.[D]	4707.2955

10096	[A].GPNLLKNMTQLLCVEAFEGEEPWTPSALDGSFPSLLPPDPSPGV.[Y]	4707.29971
10097	[N].LDCLLDTMSHRLGPGSPPGVWVCSTDMLLSVPPSPGISWGGFRG.[A]	4709.27715
10098	[G].MPGLPGRDGMTGAPGLTGERGEKGEPGERGPPGFPAYLDEELQGTL.[H]	4710.25628
10099	[Y].TGIKDKEEKEASNGEDKGANSREEEKGANREGEKEKEAYKEI.[N]	4710.27252
10100	[P].TRPRGDSTNSHGPEPLDGLRRGPGTEPWRQSWIDCAPGAAAGQV.[T]	4710.27269
10101	[N].KSGSNVMVGTLCRKRMCGGRGVGPGQTLRQEEDRHGGGQKQGPA.[K]	4710.30527
10102	[Q].QPDLLPSAAQRLTALYLLWEMYRTEPLAANPFAASFAHLLNP.[A]	4710.45411
10103	[V].IVGALMASGKEVAGKIPKGKLVDFEAMTAPGSEAFSKIAKSWMNL.[K]	4710.47075
10104	[A].LSAVTPLPEHPAQLDPFPSVQQSVPQTIALSNLPQAFVGHPPVSV.[L]	4710.493
10105	[I].PVNSAGSPVGDVPFIRSGLLGFVGPGSLVFVVGKMDGLLMVSGRRH.[N]	4710.50108
10106	[L].IDVYAGPCSQKADAIFRLNWATYLASTENIVVASFDGRGSGYQ.[G]	4711.28856
10107	[A].GGAAGGGPAAGPADHGLAGRGAAGDGPAAALLQAAGVAADWAAAGLADGARAAGHA.[G]	4711.32208
10108	[L].SPLEESYKKVGMEGGGLGAPLAAYRQGQAAPPAAAMQQHAVGHHGAV.[T]	4711.32564
10109	[S].VRLEDTLADGAESPRQPPGELGAPAESSSPLRSSPEPPPLSPEPPP.[D]	4711.34858
10110	[D].PGAAGGRGGRGGSPGGTRRATCPRPLRPGAVADSAASFGGPVAEPASSPR.[G]	4711.38431
10111	[A].PSVMGAGPAGPSSQAPGTVIAAFIRTSSATAAPGVKEGPLRPSSYVQGM.[M]	4711.39707
10112	[R].RPTKSKGSKSSRSSSLGNKSPGPGTLSGQSAASVLHPQQTLPAPGSVP.[E]	4711.48742
10113	[A].LEGARRPGPLMQGVLQTCRDLPALRDELFLQLAKQTSGPAGPPG.[P]	4711.49231
10114	[Q].ARPSQNLEAPLGSPRGSLDPPVPRASRGQMLLSGGPRGPVPQPGLQ.[P]	4711.49615
10115	[G].MLAGPAAAASIKSPPVLGSAAASPVHLKSPSLPAPSPGWTSSPKPPLQS.[P]	4711.52801
10116	[A].SYHDEEEDEYEAEDDEEEEDEGGKDSDAESSDLFTNLNLGR.[T]	4711.84701
10117	[Q].EQQQQQQQQQQSILFSNQNAMAPMASQKQPPPNMIFNPSQN.[P]	4712.20387
10118	[A].DAAYEELMKRQQMQLTPGSSPTQPLIRDDMTESTVDFDRVP.[D]	4712.22768
10119	[P].SPSPAGIPHGPQTVGNHFQRTPITNQSSSLTATQMSFPVQGVHTV.[A]	4712.32741
10120	[P].PPSMQNHIPQVSSPAPLPRPMENRTSPSKSPFLHSGMKMQKAG.[P]	4712.33165
10121	[P].PGEEVKSTLSMLPMVLPGMAAVPQMFGVGGLLNAPMATTCASAVPAP.[L]	4712.33387
10122	[R].VGGNFNRGSLKAPWAMGSRFWLQQSPKSFHPWPFQNTRAHS.[P]	4712.33813
10123	[F].EGPPGPVGTPLRFEGPIGQAGGGGFRFEGSPGLRFEGSAGGLRFEGPG.[G]	4712.33929
10124	[S].PSRSGTPNLPGTPGATPPPPQFPAGIWGSGLGGAEGGAPASRAGLPSGPGPG.[C]	4712.36042
10125	[P].THTLWIAGSESSAITSVMYTVITPMLSPFIYSLTLGVFLSSGTT.[Y]	4712.37656
10126	[L].VTLEDINDNGPMLTISEGEVMENKRPGTLVMTLQSTDPDLPPN.[Q]	4713.25798
10127	[G].EFMTSRVNWVVQSSAVDYLHLMLVAMKWLFEEFSIDGRF.[C]	4713.29787
10128	[P].PQMSAAAAAAAAAYGRSPMVGFDPHPPMRATGLPSSLASIPGGKPAYS.[F]	4713.30106
10129	[Y].VADSGSNQLLGTIVSAGDTSVLHLGHVDHLVACQGTPEPTELPHPS.[E]	4713.32132

10130	[L].QGGALSAMLDFFQALVVTGTNNLGYMDLLRMLTGPVYSQSTALT.[H]	4713.32488
10131	[D].APGPAAPQEDSVDLLGLHVEAGPAPAPHAPGGPPSNADLLSCLLGAPDP.[A]	4713.32534
10132	[P].GGQGPPGSSGPPGVKGEKGFPGFPGLDMPGPKGDKGSQGLPGLTGQSGLPG.[L]	4713.33658
10133	[L].TLPSAGASTHPLPSTPHAPTPLSSSPPPSSVSLTASAPTSPSDSQTPLP.[P]	4713.353
10134	[D].FTTTKRDEYGPGVQGKEPPPAHVDAAAGAVPPSPPAAALGATCAAAFP.[S]	4714.33585
10135	[A].PGYPAGPGLAQPSSHREAGGGRGVAPPRERRAGPGVFEAQSRPPPW.[Q]	4714.39988
10136	[E].ELPSEGRGMVLTVEVMEAMIIIMAMDLGQINLEETSITVFQE.[R]	4715.28842
10137	[R].GPQRSVGCWGPLRGIQEQMGPVWDGQPPVSAPGRRRPPGGPHGM.[G]	4715.31536
10138	[S].GVEDISGLPSGGEVHLEISASGDLSRLPSGEGPEVSASGVEDLGVLPSG.[E]	4715.31701
10139	[T].FAAVHCLSGKCQGSVMLYRANKYPLEMLGPVTFMWKARGAPG.[D]	4715.33284
10140	[F].TSGPQQLLGDPFQGMRKPMSPVTAQQMSQLELQQAALEGLAVPH.[D]	4715.37423
10141	[T].PLQAMMLRMAGQEIPEEGREVEEFSEDDDEESDDSEAEKQS.[Q]	4717.98221
10142	[G].RAGLHCAQAYPVRTTGQELPFAYSGQPGSGGLASMAGDADLIDSLL.[K]	4719.293
10143	[P].ASASPAPNATADGSKTSRASVDTTPSVIQHRAMMRFSELEMKER.[E]	4719.30358
10144	[R].GRAGDPRAAQGAPGPRQPQAALPRVPYRSCPGGRRSPGAQAQASGAL.[S]	4719.43701
10145	[G].PMGQQGIPGIPGPPGPMGPPGKTGHCNPSDCFGAMPMEEQLKHGH.[R]	4720.10806
10146	[I].PENNPRGISIFSVNAHDPDSGNNAQVTYALAEDKFQGTPLSSYV.[S]	4720.25502
10147	[W].YELHAREWPFKTQPAEAIIWQMGTGMKPNLSQIGMGKEISD.[I]	4720.29966
10148	[F].YKLLRDYEPQLEDFVDQFVELQGEEIIAYKPPGFSLMYH.[L]	4720.33199
10149	[R].GPAGPPGPQGPPGEQGPEGIGKPGAPGTPGQPGIPGMKGHSGAPGPAGLPGAPG.[F]	4720.3325
10150	[H].PGPHGPLGPQGPPGPQGNAGPQGHLGPQGPPGPQGHIGPQGPPGPQGHLGP.[Q]	4720.34169
10151	[G].GLSPSHEDGTSGAKKPEARAALSHVAGRPFTPQDTLLVAVDDQMF.[M]	4720.34239
10152	[P].WPPTSISMEMPAAPGSQNQVLGAEDSQSLPPPPRWERTNEVSD.[D]	4721.18826
10153	[V].EIESEALQPSAEAVAANPGAMLELGPPHGVSAEEAGLGPQMAGQPLEA.[E]	4721.27092
10154	[A].VISAMGQDIDFNLHLPSSPAPSGTAPSGPAASRAHLTPPCSPGAPAEA.[T]	4721.27226
10155	[P].TPSPSTPAPPTSSPSSPAPPNPSPSNPTPASFAPVPAPEASPPAPDSPAI.[S]	4721.28934
10156	[A].SPKLEPATGPAMQAGGPGTPQGPASEHKTPWPLMETLDAQLAAQSPG.[M]	4721.29741
10157	[P].GPQGYPGIGKPGMPGMPGKPGAMGMPGAKGEIGPKGEIGPMGIPGPQGPP.[G]	4721.31691
10158	[S].ANPVGSSTFKPDLSSTVTLSSSTVTLSSSTLPTTDIIPSSPTTHNTS.[T]	4721.35272
10159	[A].ASSAVLMDVSRREHGEVLPLKIVTYAAVSLSLAALLVAFVLLALV.[R]	4721.70418
10160	[V].AGPGIDEPNAFGNTALHIACYLGQDAVAIELVNAGANVNQPNDKGF.[T]	4723.28454
10161	[A].LNPFSGPAYPTGPSAASSSGPAAASGPLATSSPAYSPGLSSPGQAYSAASAS.[S]	4725.22271
10162	[E].PSSRGGSLRRGGEAGGTGDGGPPPSRGSSGGAPNLRAHPGLHPYGPPPGM.[A]	4727.31047
10163	[N].DVDAAQPRETPRETPRPPMPPAKPSPAPETSSAEVAGASGLDQRF.[G]	4727.31182

10164	[V].AGQAGKEAEKFGQGVHHAAGQAGKEAEKFGQGAGKEAEKFGQGVHHA.[V]	4727.32102
10165	[E].KFGQGAHHVAGQAGKEAEKFGQGVHHAAGQAGKEAEKFGQGAGKEAE.[K]	4727.32102
10166	[E].AEKFGQGAHHVAGQAGKEAEKFGQGVHHAAGQAGKEAEKFGQGAGKE.[A]	4727.32102
10167	[S].AASMAAVAQRSMPLQTGAAQICARPDPFQQALIVCPPGFQGLQAS.[P]	4727.33132
10168	[K].WCNKDRKKSSSEAKPTSLGLAGGHKEPRERSMSETGTAAAPGVSA.[E]	4727.33766
10169	[P].SVAGKLSDIKSTWSSGSASHTQASLSHELWKVPRNTTAPTRPPPG.[L]	4727.42884
10170	[L].PGQPGAKGERGPKGPPGPPGLQGPKGEKGFGMPGLPGLKGPPGMHGPPGP.[V]	4727.44496
10171	[L].IQAEDRHLIIRDLLQDVHDKILDDEAAQELMPVVAGAVFTLT.[A]	4727.47128
10172	[S].PVSGEPPVSLPFLGKMGFLQRLLESQPHHARLYQAKADPPTTT.[M]	4727.47664
10173	[E].AALAAPAAEAASAAPDPPAAGAAPAAPAAPAAPAAPAAPAASVAGAAPAALVTRGAP.[V]	4727.49038
10174	[S].IQPEKPVGGSEAAVPGTVSVQTLKPLAGPGGANTGVVTLHSVGPAAVPGGT.[T]	4727.53666
10175	[A].RSGGAGGGAYGFRSGAGGFGSRSLYNLGGNKSISISMAGGSRAGGFGGGRS.[S]	4728.29449
10176	[T].ASLKGYEGHVPGVAFSFGSPYGTTTLKYFQDQRNAALGRSSTDF.[S]	4728.31174
10177	[S].GPSGPPGPKGDDGIPGQPGLSGPPGPKGEPGHPGTDGAAGQRGPPGLKGEQG.[D]	4728.31493
10178	[G].QPGPKGDQGEKGERGPPGVGGFPGPRGSDGSSGPPGPPGSIGPKGPEGLQG.[Q]	4728.31493
10179	[L].STVSQISSVSMASGSVKMTSFAERKLQRLNSCETKSSTSSSQKT.[T]	4728.32371
10180	[L].QREAAGGVTMSTIGAESIRDEEAAPGQAAVTVRGGADGKTVTMSVPGAA.[M]	4728.33157
10181	[E].EGPGLGGWQGPGESSRGAARAPEGMWRGPPRESEESWSLLGGLSGH.[R]	4729.23491
10182	[A].GPPPNSMGLSNSLAGSNGAGLQSHLYQPAFPGMVPASLPGPSNVSGSPQ.[L]	4729.27735
10183	[-].MVTGSTEVGQTLEASPDAPRAQVSSPEPLVPEAVAGAEMSVAQAPGADP.[P]	4729.27851
10184	[G].VGESLTDPSVIKAEEKRSCDMVFGPANLGEDAIKNFRAKHHC.[N]	4729.29195
10185	[S].IQRSGSSMKEEPLGSGMNAVRTWMQGAGVLDANTAAQSGVGLARAH.[F]	4729.29916
10186	[M].QGPPGPRGMQGPPHPHGIQGGPGSQGIQGPVSQGPLMGLNPRGMQGPP.[G]	4729.30452
10187	[Y].YSVTPPPGSAPGPGPLSGSQGPGQQCLGQAGLPGSVPASTHSLTHSLTH.[S]	4729.30634
10188	[K].AAEAEAVDRSFGEKRYDPLQATPPPPPLPTQYSQGAHPSPNTALG.[L]	4729.32812
10189	[S].SGHHRNLIVMPALSPPDLSAPHCRMSGGSRGQLGPSRADLASLGH.[P]	4729.33689
10190	[I].VGGACIYKYFMPKSTMYHGEMCFFDSEAPTNALQGGEPYFL.[P]	4730.08451
10191	[L].HHTPPPASTAAGMPSLQHPAAPGVTPPQPAAPTQPSTPASSSGQTPTPT.[P]	4730.29036
10192	[S].LNNGQPKSARCQASASPEVLASHSGHPTADLQTFQAKRHMHQH.[R]	4730.29238
10193	[G].TTLGPPAASTPAGPPSGGASPTPPAASPSGGSATRPSSGPTSEAPRPPEPSQ.[G]	4730.29286
10194	[W].HFVVSPSFEYTIMAMIALNTVVLMMKDVYFSSLALDDHLSF.[Y]	4730.29409
10195	[E].ELQNQANFSFAPLVLDMLNFLMDAIQTNFQQASAVGSSSRAQQ.[A]	4730.29775
10196	[P].ASTAAGMPSLQHPAAPGVTPPQPAAPTQPSTPASSSGQTPTPTPGSVPSAS.[Q]	4730.30025
10197	[E].QSGQSGAESSLGPLNLSTGLPPTVWGHGWPGAPTNCSALPRTLGVMS.[E]	4730.30898

10198	[A].TEGPETKPVLMALGEGPGAEGPRLASPSGSTSSGLEVVAPEGTSAPAGGPG.[S]	4730.31015
10199	[D].SRVASSSKGADSASVTMVVTPSVPGGGMTTMPVSTLSSNPMDSGWTLS.[C]	4731.22563
10200	[E].GCKALSAALTCSQHLASLNLMHNDLGLRAMTTLCSAFMHPTSK.[L]	4731.27546
10201	[H].HTPPPASTAAGMPSLQHPAAPGVTPPQPAAPTQPSTPASSSGQTPTPTPG.[S]	4731.31076
10202	[G].HLAPAGPLASAESPEPVSWREGPSGHSTLPRSPRDAQGSATSELSGP.[S]	4731.3146
10203	[P].GPEASLKQSAKSIYEQRKRYSTEVMSEVSTNAVNHLVTFCLG.[E]	4731.35051
10204	[G].SVGRPPRPSAQPQSNPGAAWAGPWGGRRPGPPSYEAHLLLRGAAGM.[A]	4731.39744
10205	[R].KGGGSAASYRTPSKGAGAAFGSRSLYSLCRGDLCIPLKVAGSSVRTG.[G]	4731.4206
10206	[E].QGVDIADVAESGPGLAFIAYPKAVTMMPLPTFWSILFFIMLLL.[L]	4731.45667
10207	[Y].PGMAGPKGETGPQGYKGMVGSIGAAGSPGEEGPRGPPGRAGEKGDVGSQGV.[R]	4733.27947
10208	[R].MLAVLPANGKMHSAVDCNGVVSLVGGPSVPTSPVGQLLPEVIIDKPA.[T]	4734.50311
10209	[P].GPPPHKSLRCGEEEAAGPPGPPPPHAQRGLGLAAGAGGPAGPGGPGGGAGVR.[S]	4735.37709
10210	[C].LYDAEVAQIQTHTSETSVILSMDNNRDPDLDGIIAEVRAQYE.[D]	4736.2632
10211	[L].RAPGAQLVVGHMVELHCEAQRGSPPILYQFYHENVTLGNSSAS.[F]	4736.30965
10212	[P].GASPGPEARSAGDIPVEKLNLGAETDSPPQKSPLGPPSSPSSLPSEEV.[T]	4736.35373
10213	[Q].SAFYPQKSFPEKAPANGVEQTQKMVTPAYNRFTPKPYTSSAR.[P]	4736.35658
10214	[P].GPSTQVTAGSNHTAALLMDGQVFTFGSFSKGQLGRPILDVPYWNA.[K]	4736.35658
10215	[G].ADGGPAGFPARGGNESLPGSARRNQHAGGDSSQPGAGAEATPETPSAQPP.[S]	4737.20209
10216	[S].LGNTPSSPPASPTSPRASSEASPDPQPPEKPGRAKENTDSLNPEEL.[C]	4737.28744
10217	[D].KALGYHTQELEVYQELSDLPGECRAHGHLAAVYMALGKYTMA.[F]	4737.28983
10218	[P].ETHPRGLCKNRDLGTTEGSGRAATKDSVSVSQTNNAERQVEPSP.[S]	4737.29936
10219	[A].PGPASSPEASPAPGFPFPPPWMGMPLPPPFAFPPMPVPPAGFAGLTP.[E]	4737.30592
10220	[L].QALSEGHGVSLGSSLASPDLKMGNLQNSPVNMNPPQLSKMGSLDSK.[D]	4737.32806
10221	[S].SSGALSVSSVGMGQPAAQAGVPQGQVPGTALPNPLNMLGPQASQLPCPP.[V]	4737.34332
10222	[S].PSLATSPMYFDYQTRLPLSSPRSEVMYLKPASNNLTVPQGHAG.[C]	4737.34397
10223	[R].PPHHAPPGPAAGAPPPGCATLPRMPPDPYLQETRFEGPLPPPPPAA.[A]	4737.34933
10224	[I].EAVEVEVGRFRDQQYEMLKRWRQQQHAGLGAVYAALERMG.[L]	4737.35252
10225	[C].SPASQPWSPRSGPPLNNNPPAVVVNSPQGWAGEPWNRAQHSLPR.[A]	4737.35701
10226	[R].TPSSPGTRAPGNSSSSRPATAIPPATPTLATSAQPAAATPASVSSPAGSPGP.[P]	4737.37144
10227	[H].PSPRANADPGPTGGTAPDSPRAFLAAMEDGVYEPPDLTPEERMEL.[E]	4738.20357
10228	[A].SSPMSTPDPALGGTPRPGPSPGPGPSPGAMLGPSPGPSPGSAHSIMGPSPGP.[P]	4738.23343
10229	[M].GPVMDRMATGLERMGANNLERMGLERMGANSLERMGLERMGAN.[S]	4738.23434
10230	[P].AAEGVGAAANAAATSSTGTGGVAASGMAASGVVPGGGFVASAAAEVQTGRNNFV.[I]	4738.27616
10231	[N].PYSGDLTKFGRGDASSPAPATTLAQPQQNQTQTHHTTQQTFLNP.[A]	4738.28805

10232	[T].SPEGIGGYIHSHRPLGPGEFESFIDVYAIRSAEGAPQKEVYFM.[G]	4738.30349
10233	[R].GPQGITGPKGGPPGIDGKDGTPGMPGVKGSAGQAGRPGNPGHQGLAGVPGMP.[G]	4739.35291
10234	[P].WLIAESFSEELVDEALGAVAAELQDMCEDYAEAVFTSEFLEP.[A]	4740.14193
10235	[G].TGTCDKVNNIHPAVHTKTDNSVASSPSSAISTATPSPKSTEQTTTN.[S]	4740.26532
10236	[L].DPRSEGGGDGRPPLLGRAHPCVGTPGPFTMQGLLGCELGPDNVSVP.[V]	4740.27155
10237	[P].ASSPTVNQTQQQMGPRPPQNNPLPQGFQQPVSSPGRNPMVQQGN.[V]	4740.27539
10238	[V].PGIERMGPGIDRIGGAGMERMGAGLGHGMDRVGSEIERMGLVMDR.[M]	4740.283
10239	[N].HVSSPPPQALPPGTQMTGPPGPPPPMHSAQQPGYQLQQNGSFGPAR.[G]	4740.28344
10240	[F].QQALGMFSAALASGQLGPLMCQFGLPAEAVEAANKGDVEAFAKAMQ.[N]	4740.29286
10241	[L].QPGPTPSGTNVGSSGRSPSKAVAARAAGSTVRQRKNASCGTRSAGRTT.[S]	4740.41673
10242	[P].GPQGVPGPPGFGGEPGPQGEPGPPGDRGLKGENGVGQPGLPGAPGQGGAPGPP.[G]	4741.3142
10243	[K].QRISSFETFGSAHPPDRGAQRLSLQASSSSGEAAELPGKQEGGRAS.[G]	4742.32656
10244	[R].QGPPAGPAAPSSAPASSSPAAPAGALDRHQDSPVTSLRNWVDGVFHDP.[I]	4743.29347
10245	[G].KLWIEQYGTVEILNHSSPGPSLVPSALGFQTGRTGREAPHVVQS.[D]	4743.46416
10246	[D].EPSGAHLPGKQTQAENGTASGATKAEKGPACSRGGGYRLFSGNSRAQ.[R]	4744.2993
10247	[L].KGDQGVPGERGPAGPPGPQGPPGEQGPEGIGKPGAPGTPGQPGIPGMKGHS.[G]	4744.32847
10248	[-].MAADGERSPLLSEPIDGGAGGNGLVGPGGSGAGPGGGLTPTAPPYGAGKHAPPQ.[A]	4744.335
10249	[M].AADGERSPLLSEPIDGGAGGNGLVGPGGSGAGPGGGLTPTAPPYGAGKHAPPQ.[A]	4744.335
10250	[M].GPPGPQGQPGLPGTPGHAVEGPKGDRGPQGQPGLPGHPGPMGPPGLPGLDG.[L]	4744.34373
10251	[P].PRARIVGYRLTVGLTRGGQPKQYNVGPSASQYPLRNLQPGSEY.[A]	4744.51826
10252	[Q].QQQLCAPPAPAAPTEQTPALPPANGNPLLPFPGPPVRPNVKNRLL.[V]	4744.55081
10253	[N].FLDGLANSFSGSALFYLGLTMVGKIKKLKKSAFVVLILLITAKL.[L]	4744.78573
10254	[A].LEQGEEERPWPQDLQPPPPPGPHEDGQEEKSSTDERPSTEPL.[F]	4745.18739
10255	[A].AAAAAQMHAKNGGGGSGSHRSSPVPGAPAVCEPLAVPAASPMAAAAEGPQQ.[S]	4745.27295
10256	[E].LAMQQICANVMEYCQSLLLQSSPTFQHAVCLFTPSLSETIN.[R]	4746.23805
10257	[E].SSMGGLLREEGGGQAAPGQGLLSAESRAQEPSPEEEAPGETLPAATPE.[E]	4746.24352
10258	[P].GQPGFPGPKGEMGVMGTPGQPGSPGPAGVPGLPGAKGDHGFPGSSGPRGDPG.[F]	4746.25762
10259	[V].SLTAGSMIGSGIFMAPQGVLVYMGSPGASLVVWAVCGLLATLGALCY.[A]	4746.38762
10260	[D].ENNLPLAVSTLNCNVSGRHLETNSERDPRHVNKAILKLLVPK.[E]	4746.55841
10261	[L].PSRFHQYQQHRPSLEGGRSPATGHCGAQQVPDQAQAAAWVPAPA.[T]	4747.28319
10262	[L].QRMTVIVQEMGGDQLVFMKGAPERVASFCQPETVPTSFVSEL.[Q]	4747.28744
10263	[S].SQQSQQNVLSGHSQQTSLPSQTQSALTAPLYNTMVISQPTAGSMV.[Q]	4747.29379
10264	[R].STSPSVVENMTVLDVDKQNTKEEIGLTEEVKESTTSSRTTTFE.[V]	4747.29897
10265	[L].IQGSLDGELKLMDELAGPGGQPSAFPPARSPGGSDPQIPLAEMEALS.[L]	4747.32296

10266	[E].KTEAAKEESQQMVLDIEDLDNIQTPESVLLSAVSGEDTQDRTD.[R]	4748.25784
10267	[G].EAAELPGKQEGGRASGPSGRGAPPTMEQQRPEPEQLPPASPATPEAS.[D]	4748.2969
10268	[D].GKDGTPGMPGVKGSAGQAGRPGNPGHQGLAGVPGMPGTKGGPGDKGEPGRQ.[G]	4748.31284
10269	[P].PAAAPDAAAGGAQTLADGFTSPTPPVVSSTPPTGHPVQFYSMNRPAAR.[H]	4748.31618
10270	[F].INRNAAGTGRMSAPRNYSRSGGFREGRTGFRPVEAGGQHAGRSGE.[T]	4748.31802
10271	[H].LQQYSREHALKTQNANQAASERLAAMARLQENGQKDMGSFQL.[S]	4748.33799
10272	[T].KTKQGDNEHKPMNNPSAQIYQALASELKTGFTEAMQELSRIQ.[H]	4748.34068
10273	[V].AAVAFGKGLSNWRPSGSSGPSQPGQPGAGTVLAGASGLQQVQMAGAPSQQ.[Q]	4748.35977
10274	[P].GTIQGLEGSADFLGPQGIRGYPGMAGPKGETGPQGYKGMVGSIGAAGSPG.[E]	4749.30356
10275	[P].RGPDGASYLGGPPGGRPGATYPSLPTSFAGLGAPFEDAGSYSVNLSLAP.[A]	4749.32197
10276	[P].APRGPDGASYLGGPPGGRPGATYPSLPTSFAGLGAPFEDAGSYSVNLSL.[A]	4749.32197
10277	[L].WSIMKNCIGRELSKIPMPVNFNEPLSMLQRLTEDLEYHQ.[L]	4749.32958
10278	[Q].EFQKDAKQAEAILSNQEYTLAHLEPPDSLEAAEAGIRKFEDF.[L]	4749.33187
10279	[F].PLASDIDPPDLDLGWPEVPHATGFSPTQAVVAIVMDIFTDMELL.[C]	4749.33542
10280	[D].PTGVDRDDGPRIGVSYIFSNDDEDMEPQPPPQGPNGGGVGLPDGGDG.[P]	4750.12341
10281	[A].GTHGQAEKPRVQAEAGHLQPGDRGRPGRLDSCTTASADPETTPAGL.[V]	4750.30986
10282	[S].PQNHALQPHHHIPMVPAQQPMVPQQPMVPVPGQHSMTPIQHH.[Q]	4750.32751
10283	[L].TALDSGDPPQSGTAQIQVLVVDANDNPPVFSQDVYKASLREDVPP.[G]	4750.34825
10284	[S].AASVTSAGATSASSVHLPVSAPHGAGLMAAASAQEETYGPRAVTSAQQKA.[G]	4750.34893
10285	[A].PAYGAEALERMFLSFPTTKTYFPHFDLSHGSAQVKAHGEKVAN.[A]	4750.35111
10286	[G].GPGEPGPPRGPYAGYRTYGAELPATPAFSAFSRALGAGHFSVPADYA.[P]	4751.3066
10287	[Q].QRPSGYVHQQAPTYGHGLTSTQRFSHQTLQQTPMIGTMTPLGP.[Q]	4751.32055
10288	[G].GPSPATSSPVPPMASGGFLGFLEANMFSVIIPICLVLLLLALIVPL.[L]	4751.58801
10289	[P].MAHYPSQPVFAPMLQSNPRMLTSGSHPQAIVSSSTPQYPSAEQP.[T]	4752.26434
10290	[G].PSASASAGPPASARPGTSAAARAATSASARADMSATARPGPSASAHAGHPASA.[R]	4752.30036
10291	[A].PAAGTATISQDTSHLTTGPVSGLASGSSVLNVVSMQTTTAPTSSASVPGH.[V]	4752.32686
10292	[H].RSLSSSSQGPALSMSSSIYRRGVTPSVYGGAGGHGTRISTSRHMVN.[Y]	4752.34414
10293	[S].KYGDLANWMIPGRKVKGMGGAMDLVSSSKTRVVVTMEHCNKAN.[E]	4752.36632
10294	[A].AADTITIATPESLTEQVAMTLASAISEGTVLTARSGTSGAEQATVTMV.[S]	4752.38053
10295	[M].GPASPAARGLGPLLRLLPLLLLLLRVQLAEGNLAGGSPSAAEAPGSAQV.[A]	4752.72507
10296	[-].MGPASPAARGLGPLLRLLPLLLLLLRVQLAEGNLAGGSPSAAEAPGSAQV.[A]	4752.72507
10297	[H].LETPQPSPQEAGTVDVWRIPEAGAAHSGLTPESGAEEPFLSHASSL.[L]	4753.30163
10298	[G].SGKDLDACGQLSTGPHRGHLHPGAGGRERCASPLGAGAAGSQGPAGGPAP.[D]	4755.27237
10299	[P].SQASLAPTPVASPMTPSAASFSFGSSGFKPTLESTPMPSVSAPNVGMK.[P]	4755.29905

10300	[V].PGNIVYSSLCGLGSEKGREAATTTLGGLGFSSERNPEMQFKPNTP.[E]	4755.31413
10301	[F].PLDYYSIPFPTPTTPLTGRDGSLASNPYSGDLTKFGRGDASSPAPA.[T]	4755.3213
10302	[E].PQGQAFPGSAGPALQYPPPAYPGAKGGFQVPMIPDYLFPQQQGDLG.[L]	4755.33406
10303	[T].SRPPATNSGVFAATTGPIQAAFDASVSVPSEGLPQGTSSAPQAPAHPTGA.[S]	4758.33941
10304	[F].PGTGEKGEKGEKGEPAVVEQGQQFEGPPGAPGPRGVVGPSGPPGPPGFPG.[D]	4758.35467
10305	[W].VAQEGRGEKNSHPHFLWKHDEMPLLGLNEGKTLNDGIMFPSL.[A]	4758.35554
10306	[S].VYANIGDVKNFEAAEGIEEPLLHDICYAENTDDAEDEDEVSC.[E]	4759.0458
10307	[V].APTSSTKSTSVNFGSLSMTPFGGSSAVTPFGGASSSFSVVPSSYPAGLTG.[G]	4759.27197
10308	[E].GTALNNSNSSLLLMNGPGSLFASENFLGISSHPRNDFGNFFGSAVT.[K]	4759.28454
10309	[K].NMHVVDVELSGPPGPTGRSFTVHTHRENPAEPGAVTGSATVTTFW.[R]	4759.29578
10310	[P].KDVFTFSSRPRSAPHGKTQTMSPEACLFTLDLKEDTSVTRSD.[T]	4759.30904
10311	[G].LSNWRPSGSSGPSQPGQPGAGTVLAGASGLQQVQMAGAPSQQQPMLSGV.[Q]	4759.33151
10312	[F].LCENQGSRKRRQPPGNEAEARVRPEEEQEPLMEMRLRDAP.[H]	4759.33219
10313	[W].VMMFVMCLTVVAVTVFIFEYLSPVGYNRSLATGKRPGGSTFTI.[G]	4759.45226
10314	[P].GPQGPKGDKGDPGVPGAPGIPSGPSRGGSSSSTMFMQGPPGPPGPPGPPGSL.[S]	4760.28316
10315	[G].AAAPAPAPSGLGYGPPTSLASASGSFPNSGLYGSYPQGQAPPLGQGHPGAQ.[P]	4761.29682
10316	[E].GSIPDTPKSADASQDSLSSSPLPLEMSSIAALENQMKMINAGLAEQ.[L]	4762.27436
10317	[A].SPGSPPGAEVEALPEAAALEVAEPPAEALGEASESCPLRPGEVGAGPGQG.[A]	4762.27885
10318	[G].RSYAPYHHHQPAAPKDLPGAAAAQAASWYLNHSGDLNHLSGHTF.[A]	4762.28351
10319	[T].TPFNITSSVSSGTMSNPVTVAAAMSMRSPVNVSSAVNITSPMNIGHP.[V]	4762.29432
10320	[G].SPVSKMSVSRSSSLRSSSSLSSQGSVASSIGSQTSFRSTDFATPGHP.[K]	4762.29729
10321	[T].STLSSVTSTTASTSQPFLFGTPPTSGTSTPTFGQNTPAPGVGAAGGSLSF.[G]	4762.30063
10322	[A].GGASSPSPVVFTVGSPPSGTTPPQGPRTTMFSVGSSSSLSSAGSSSARHL.[A]	4762.30131
10323	[E].ALGPPSSQAFLSFSTAPMAGGGLPAGEDPGALLANSHGAAQAPSNSLTAAE.[A]	4762.30534
10324	[R].PGTSAAARAATSASARADMSATARPGPSASAHAGHPASARPGPSASARPGPS.[A]	4762.33233
10325	[H].TASPWNLSPFSKTSIHHGSPGPLSVYPPASSSSLSAGHSSPHLFTF.[P]	4762.33248
10326	[V].APEVFTHVLYYVYSNHTEVRPARPGQGCPGAALAAEAPSTLPGGPG.[R]	4762.34708
10327	[V].GTAVIAMFFWLLLVIVLRTVKRANGGELKTGYLSIVMDPDELP.[L]	4762.60784
10328	[P].GPQGHIGPQGPPGPQGHLGPQGPPGTPGMQGPPGPRGMQGPPHPHGIQGG.[P]	4763.29674
10329	[P].QGHIGPQGPPGPQGHLGPQGPPGTPGMQGPPGPRGMQGPPHPHGIQGGPG.[S]	4763.29674
10330	[D].HYSLAGAWTHKMGTAHVSVLGEDGSAVAATSTINTPFGAMVYSPRT.[G]	4763.29808
10331	[R].PGRGIMAEMKTPLYPAAYPPLPAGPPSSSSSSSSSPPQQLSAATPHG.[I]	4763.30798
10332	[S].PIGESTQVSSGGLQQNPSQVSAELAQSYSSAIPSSGYPPPAKVKSCS.[T]	4763.31048
10333	[S].GGRLPYGVRMTAMGSSPNIASSGVASDTIAFGEHHLPPVSMASTVPH.[S]	4763.31269

10334	[M].AAGGGGPAPLSSAASSPLSSSLGTVGHRANSPSLFGTEGKPKMEPVASSQA.[A]	4763.36933
10335	[A].AGGGGPAPLSSAASSPLSSSLGTVGHRANSPSLFGTEGKPKMEPVASSQAA.[M]	4763.36933
10336	[-].MAAGGGGPAPLSSAASSPLSSSLGTVGHRANSPSLFGTEGKPKMEPVASSQA.[A]	4763.36933
10337	[E].PGASWSKSPGSPTPSMSADQAASSPLLPLNSPGLSQGDVSRQDEAVSP.[G]	4764.26935
10338	[P].TQPGPVLYMPSAAGDSVPVSPSSPHAPDLSALLCRNSSLGSPSNLCG.[S]	4764.27021
10339	[G].TLQSAGTGSEEGARGLGGGIKSSGGGQELAPEDGPLVQDPQKVADEEGQ.[E]	4764.28308
10340	[P].RALEGQFDPEVGSQAEGAEDELEIRVGPSAVGGQVGQAGEGVHPHTP.[T]	4764.28844
10341	[H].GGPPQGAVPQSGVPALSASTPSPYPYIGHPQGEQPGQAPGFPGGADDRI.[R]	4764.30772
10342	[M].QVEIESEALQPSAEAVAANPGAMLELGPPHGVSAEEAGLGPQMAGQPL.[E]	4764.31312
10343	[Y].TYPPSSLGGTIVDGQTGFHGDTLNKAPGMNSLEQGMVGLKIGDVTTS.[G]	4764.31312
10344	[S].VPTNSAQQGHSSPDSPIASATKGIPGFGSTGNLSSAPVTYPSAAAPGVNN.[T]	4764.31359
10345	[R].FGSGGAFRAPSIHGGSGGRGVSVSSARFVSSSSGGYGGGYAGALATSDGLLA.[G]	4764.31493
10346	[Q].APIPAMETSAATALTEFVEPPCRQAPIPAMETSGQRPQNSTSARP.[P]	4764.31783
10347	[F].TLQGHSGAITTVYIDQTMVLASGGQDGAICLWDVLTGSRVSHMFA.[H]	4764.32186
10348	[D].GFGTKDVTTPGHSTPVPEGKNAMSLFSSTKTDVRQDNAAGRAGSSSL.[T]	4764.3282
10349	[R].AGVGSSKGASEEFPVLPGPQDTAREVTGQGASGRSVPLPMEGGPRDTK.[L]	4764.36458
10350	[G].ERGPAGPPGPQGPPGEQGPEGIGKPGAPGTPGQPGIPGMKGHSGAPGPAGLPG.[A]	4764.36994
10351	[Q].PAAEAAAPGWAQARGHPGGELAAAASAAGDAGWPNKHTLRILQDFSSD.[P]	4765.32544
10352	[Q].AAKPPDLNPETEESIPSRSSPEGPDPPVLTEVSKQEDQQPLDLE.[G]	4766.31667
10353	[D].GKSSGFGTPRLTSRSSSMVSLEDGEKGPAPRGSTTDSLGSQLLPEAD.[L]	4766.31736
10354	[T].HLTTTQTSGATSTGFMKAPASGAKSTPRMRAPASGAMSIPSSTAPISE.[T]	4766.31823
10355	[S].PQFQQALGMFSAALASGQLGPLMCQFGLPAEAVEAANKGDVEAFAK.[A]	4766.34153
10356	[N].GIHVTGGSMTVTGNIYIYNGPVLGGARGPGDPPAPPEPPYPIPEEGAP.[R]	4766.35592
10357	[S].GAGPGGGLTPTAPPYGAGKHAPPQAFPPFPEGHPAVLPGEDPPPYSPLT.[S]	4766.3678
10358	[L].QAMPAAGGVLYQPSGPASFAGTFSPAGSVEGSPMHTMYMSQPAPAASGP.[Y]	4767.16224
10359	[Q].PRAVGSCAPGPRARRDMAVAAQCPQPPSASSQTGLSQANLSAGPSHN.[C]	4768.29614
10360	[G].MVPPMMPGMLMPAVPVTAATAPGADTASSAVAGTGPPRALWSEHVAPDG.[R]	4768.3064
10361	[K].TSFFLGEVGNAAKMMLIVNMVQGSFMATIAEGLTLAQVTGQSQQT.[L]	4768.33406
10362	[P].GEPKSSVPDTGTPTPASTPQAVKTASSSMPLYMVTSFVSAPPAPEPPA.[P]	4768.33721
10363	[A].GPPGYPGPRGVKGEDGFPGFKGDAGLKGDRGQPGPPGPRGEDGPEGLKG.[Q]	4768.36149
10364	[E].RSSLGANQTDCLKPAEAGESGRIILPSVNSDSAHIKSEKNFQAVS.[Q]	4769.39113
10365	[L].AGPVRGVGGPSQQVMTPQGRGTVAAAAVAATASIAGAPTQYPPGRGTAPTP.[V]	4769.45401
10366	[E].KESPTPEAKEPSKDGQRDTEGTKMSPRAGSAATGSPGRPSPTNFTQ.[T]	4771.29763
10367	[Q].PVLSSLEYGTELSSGQPQVLSSAQSSPADASQPEATTEVVDRGDALP.[N]	4771.30684

10368	[P].GAREPRLMGSCHRASRVSIYDNVPGSHLYASTGDLLDLEKDDL.[F]	4771.33151
10369	[T].GQPSSQLQPITYGPSHSGTATTASPAPSHPLANSPLSGPPSPQLQPMP.[Y]	4771.34206
10370	[P].QPATTPTGSQPPSQHAAPSPVQHQAGQAPHLGSGQPQQNLYHPGALT.[G]	4772.33125
10371	[S].ASKEVGIGFAQGPGASASTAATPGPAGLPRGYMAPTSPAASERSPSPSFAG.[H]	4772.33731
10372	[W].PPMGPFPPVPPPPGSGEAAAPPSTSAAALSRPSGAATTTAAAAASAPAPGPASS.[P]	4772.36246
10373	[G].PSGTLQTAGWRAGQGWGQEGTRQGGHPLQDGGSGGRHSSAGRRGGRF.[Q]	4773.30989
10374	[P].GPAGVPGLPGAKGDHGFPGSSGPRGDPGFKGDKGDVGLPGKPGSMDKVDM.[G]	4773.3148
10375	[E].KMKMGQEFVESKEKPSRYPPTLVYQNGSIGSVENVDAGSYPPP.[P]	4773.31748
10376	[W].RSLCTSTVAQASSRTQGEDVRVEGAFPVTMLPGDGVGPELMHAVK.[E]	4773.3393
10377	[P].AGAAGGAAGGGPAAGPADHGLAGRGAAGDGPAAALLQAAGVAADWAAAGLADGARAAG.[H]	4773.35886
10378	[G].PGATQASEGPMKVQTLPNMRSGASSQAKGSKIHLLGPDWTPPNADL.[F]	4773.37231
10379	[G].AGAMLLCSLTLTLLWMVASGLECDMKEVCLGSPGTPGTPGSHGLPG.[R]	4774.27272
10380	[S].KSATVNPESKTEAKTLDDIFESSTLSDGQAIADQSEVISTVGYLE.[R]	4774.33165
10381	[V].TEAPEPPGVPVTTEATPRTNFHSSVAFMFRNPPAVAMASPASAPAPA.[P]	4774.33922
10382	[W].VAQEGRGEKNSHPHFLWKHDEMPLLGLNEGKTLNDGIMFPSL.[A]	4774.35045
10383	[P].GPADRSHRSSVSSVGARAADVLVYLADDSMVPLAVENLPSLSAHEL.[H]	4775.40572
10384	[P].AGPGREVGAPESGKQTLKIFDGSDAMQRNHFRTVTVPHLARSQE.[V]	4775.41829
10385	[S].LPTGATAPSETATDLTPGFGSAPVSMLTTVTMLDPGSSAPGGTTPISSKT.[S]	4776.34817
10386	[H].HNPWTVSPFSKTPLHPSAAGGPGGPLSVYPGAGAGGGGGGSSVASLTPTAAH.[S]	4776.35534
10387	[V].LSPGPSSGLDPDPGSALGSLPNPNPIPTSGSGSSPIPDSVLSSDPKPGRN.[A]	4776.35987
10388	[A].FLGSFVAGGMGPAASSHGSPVPLPSDLSFRSPTPSNLPMVQLWATHA.[H]	4776.37013
10389	[Q].SVGGTTAPAAGTATISQDTSHLTTGPVSGLASGSSVLNVVSMQTTTAPTSS.[A]	4777.33201
10390	[A].PDTVSHQRSKTEHDLKSLSGLPEVMEILKEGSGSVDSKGPLMEL.[E]	4777.40227
10391	[Q].KQAHYLANFMYTKEFMSIFAVVSGFGLMPSKSKESVASEGSNQ.[T]	4778.29391
10392	[S].TFSLPSAVFSFGSSSLKSSGSVPGEAPPSSSGSDGAKAALASGASPFSFAP.[P]	4778.3108
10393	[T].PAGAVSTPEQSATPAGAVSTPEQSATPAGAVSIPEEPDAPAGAVPTPEELA.[A]	4778.31667
10394	[S].MARKNEWPLDKMCLSVEVTKKNREDMTAPPREGSYVYGLF.[M]	4778.31975
10395	[R].PGTSAAARAATSASARADMSATARPGPSASAHAGHPASARPGPSASARPGPS.[A]	4778.32724
10396	[D].PEQAPVPEEAVRAPAPPCSGPSAEAYLLHPAAFHGAPSHLPTRNPS.[F]	4778.35323
10397	[Q].PNHVSSPPPQALPPGTQMTGPPGPPPPMHSAQQPGYQLQQNGSFGPA.[R]	4779.2831
10398	[R].SPSGRMRGEVSWAQMTASLLSVPPPSSSCRGPGVPAGPQASALQGQE.[G]	4779.30358
10399	[P].SAYAGLHNIPPQMSAAAAAAAAAYGRSPMVGFDPHPPMRATGLPSSLA.[S]	4779.32286
10400	[C].EVERGDRAEGAENKTGDKAGSHRGLGSSSGDGSRSPSKPAGQALEEGV.[R]	4780.28654
10401	[A].YFSASPPALCPHGRTRDLDALLAVMGAAQEFLYASVMEYFPTT.[R]	4780.28843

10402	[A].GHLTPPSSSPPWTGWSHCLISSRSRPGGKQTTTSSSGGTQGSLASSL.[V]	4780.31322
10403	[T].TTSAAPVPTTEIPTTMSTMAAAEAAAAVVAAAAAAAAAAAAAANASASTSASSTV.[S]	4780.31391
10404	[P].QRGDVYTCRVEHPSLQSPVSVDWRSQSESAQSKMLSGVGGFVL.[G]	4780.32061
10405	[P].PGPPGPPGPPGPPSGGYDLSFLPQPPQEKAHDGGRYYRADDANVVR.[D]	4780.32912
10406	[S].PNRPTPSDLAIVMYTSGSTGRPKGVMMHHSNLIAGMTGQCERIP.[G]	4781.3201
10407	[T].PGLNFGSVQLSSGNSSNIQQLAPINMQSQVVQTNQIQSGMNTGHIG.[T]	4781.33699
10408	[D].TGLATGTGPEKQKGSWSQAPGENSRNSILASSGFGASLPGSSQALTFGS.[G]	4781.34014
10409	[L].ATMATFPWVPAHSPVGDAPCPRRAAWLWWGGAGPRGQASQAAPALG.[S]	4781.35173
10410	[W].LDGGKALGSHHTASPWNLSPFSKTSIHHGSPGPLSVYPPASSSSLSAG.[H]	4781.37065
10411	[Y].TITHPSGSTSSPASRSSGTSILSSSPGLYAPASSPQAVPTSSSRQRPPG.[T]	4781.3725
10412	[G].HAQMPRAPAQQYYPHGENPPPPGFVMHGNVSPNVAATQLPTSPGH.[M]	4782.28411
10413	[S].SPVELDLFGDHTPSVKQNGTKEPDAFDLDGLGEALAESSRDTPAR.[R]	4782.31292
10414	[T].HLTTTQTSGATSTGFMKAPASGAKSTPRMRAPASGAMSIPSSTAPISE.[T]	4782.31314
10415	[P].APAHHHPEYQGQPVVSHPHHIMPPQQHYAPPPPPPPPISHPMP.[H]	4782.31462
10416	[E].AAQEEKGPGAGSASRDPGPPAPLPPGLGCDGARPRGPGLCASSCSPCR.[V]	4783.23043
10417	[M].PGASGAEVKGVCTEAGMYALRERRVHVTQEDFEMAVAKVMQKD.[S]	4783.30589
10418	[P].AANPFGPSAHPGSFLPTGHLTDPFSRSSTFGGLGSLGSNAFGGLGSHTL.[T]	4783.32879
10419	[E].QGVPEKEETPPPEEETEDAGLDDWEAMASDEERETEENTVHI.[E]	4784.04355
10420	[L].PGSPTPPMTPGSSIPYMSTSQEVKSPFLPDLKPSVSNLHPSPPGSGP.[C]	4784.35862
10421	[P].YGFPTAFGLCPKKDDPVLGAGEPKGGPGPGSGGGAGTGAGAGGPGTGHLPPGAG.[T]	4785.31143
10422	[S].YPHHATQLHAHQPQPATTPTGSQPPSQHAAPSPVQHQAGQAPHLGS.[G]	4785.3166
10423	[T].KTKSMTVATQWMKEHVGNRDTIQISLCLFIYWEDPQEGRG.[H]	4785.32219
10424	[P].GTATGLDAGGLGPAGNAASTAGPFPFHLSQHMLASQGIPMPTFGGLFPY.[P]	4785.32284
10425	[V].SRDGSGRKGEGLWGERPSSRGVREAGSWPREEAAPEEAPGGHVGKG.[P]	4785.33371
10426	[V].DEVALPSMIPFPPPPPGLPPPPPPGMLMPPMPGPGPGPGPGPGPGPGPGH.[S]	4785.33763
10427	[K].PEEVLVVENDQGEVVREFMKDTDSINLYKNMRETLVYLTH.[L]	4785.33861
10428	[I].TVSSNRDANPALYVTAGSNVTLSCTAASRPPADITWSLADPAEAAVP.[A]	4785.34245
10429	[H].RLNMSGPFGGAVSAAGLTQMPAGNVFTTAEGLFSTLPFPVYSNGIHA.[A]	4785.34397
10430	[S].PGWTGPISQDMAGTTPKASAPHPDLPRLGPEDHLVLLSPDAQGSPSS.[N]	4785.35771
10431	[E].MLQNTVTIRFENVSPEDFVGLHMHGFRRTLRNAVLTQKQDS.[L]	4785.44642
10432	[I].KQTEGEGTDVRDHTVLEMKAGFSLPDAGDLLEEVLFTEPRREA.[A]	4786.36285
10433	[P].QQRHGQLTSPTASEQLACKPPAFSFVSPTNQKTPPAPGDLAGASVL.[E]	4787.42098
10434	[Y].TEFIARLQEAIHRQGINAEATDVILQLLAYDNANTDCKKVMN.[P]	4787.42435
10435	[I].QHSCQDIGAKSLTQVRAMMYSGELKFEKRTSSAQVEGGVHSLH.[S]	4788.3403

10436	[E].EPEVPGAASAELGTSEGSVQQPLLELGPGEYRVVLCVDVGETKGAGH.[R]	4789.36252
10437	[G].REREPDVKNGHNLFMAAAAAPPAGLLSGPGLAPAASSAGGAAPSVQTHR.[P]	4789.43394
10438	[S].PSPSLMPASAQAQNAQRALSSVVLPSQGPGGGSELSSAHQLQHIAAKQ.[K]	4789.44384
10439	[D].GTPGMPGVKGSAGQAGRPGNPGHQGLAGVPGMPGTKGGPGDKGEPGRQGFPG.[V]	4790.33866
10440	[Y].VEAMKEDLAEWLNALYGLGLPSGGDGFLTGLATGTTLCQHANAVTE.[A]	4791.32805
10441	[S].YNHSQVSVQPVTTTGPEHSKPLEKSESLFAQDRDPRFSEIYS.[N]	4791.32851
10442	[E].AAGGVTMSTIGAESIRDEEAAPGQAAVTVRGGADGKTVTMSVPGAAMTIV.[H]	4791.35976
10443	[S].PGLNPGQPSSMLSPRHRMSPGVAGSPRIPPSQFSPAGSLHSPVGVCS.[S]	4791.36646
10444	[A].RPAYEAVDGTREANNRLVTYVGTSERAASDGKATGMAPDIAGLNLP.[G]	4791.37548
10445	[F].PGVPGSPGIMGFQGFTGSRGDKGAPGTAGLFGEVGPTGDFGDIGDTIDLP.[G]	4792.28354
10446	[G].GPGTATGLDAGGLGPAGNAASTAGPFPFHLSQHMLASQGIPMPTFGGLFP.[Y]	4792.32866
10447	[A].VSTPEQSATPAGAVSTPEQSATPAGAVSIPEEPDAPAGAVPTPEELAAPAA.[E]	4792.33232
10448	[A].ANTAPIFPSNMSAVSLPEDLPLGAEAFWLVARDQENDELKYGIS.[G]	4792.34508
10449	[F].SVPPEDLVAPPPYASPTAPSPSSPVPTPSPSTPAPPTSSPSSPAPPNPSP.[S]	4792.3516
10450	[L].GISIVGQSNDRGDGGIYIGSIMKGGAVAADGRIEPGDMLLQVNDVNF.[E]	4792.36689
10451	[L].QPHHHIPMVPAQQPMVPQQPMVPVPGQHSMTPIQHHQPNLHL.[P]	4792.37446
10452	[E].KAWSLSGYLLVCFSAGYFIHDTVDIVISHQSRASWEYLVHH.[V]	4792.37693
10453	[A].PLSQAPYAFQHGSPLHSTGHPHLAPAPAHLPSQPHLYTYAAPTSAA.[A]	4792.38077
10454	[P].GRPGDKGDQGLSGFPGSPGEKGEKGSTGIPGMPGSPGPKGSPGSVGYPGSPG.[L]	4793.286
10455	[K].PGDVGPPGPQGPPGKPGPAGMKGEDGLPGSPGEKGEKGETGQPGPPGLDGPT.[G]	4793.31115
10456	[S].IRSSQEEEPVDPQLMRLDNMLLAEGVAGPEKGGGSAAAAAAAAASGGGVS.[P]	4793.34688
10457	[E].SPEILEGSQPVETAQTQQLQETVGEDEQSQLVETVPKENASLQV.[S]	4793.3487
10458	[E].AGGPDLGVGRNSGSLWPGDQAPEDRRLAPNQRYNQLDFLSQAQAA.[R]	4793.35271
10459	[T].ANGVQFLPEQPLAKEAADPPGSTEETQPLEGLKGSEPPQPGGKDGAPG.[A]	4793.35406
10460	[A].ITTQPGSIFSTTMRDLSGVHTNDAVTSLSALHQSQPMPRSYFLT.[T]	4793.36616
10461	[V].EQLREAPLPRVGLQEMAEEISVPEEVPSIENRDVSSAANTCVK.[P]	4793.37204
10462	[S].SQLNTIVTMNHHHPHPHHAAVGGVSGVVAGTGGDLVGFRHHENGGVV.[L]	4793.37268
10463	[C].RNPQEPPNVPGTNGIHVTGGSMTVTGNIYIYNGPVLGGARGPGDPPAP.[P]	4793.38526
10464	[L].AVAAGRNRLSAEEARWGPGKRSAGRGAASRFACIQSGEAGTGARPGPA.[R]	4793.4374
10465	[K].LEPATGPAMQAGGPGTPQGPASEHKTPWPLMETLDAQLAAQSPGMGRG.[P]	4794.30727
10466	[I].PGSLGSGSSSPISGLTGNLEKLQSSEPSAPLAGLEKMASSENGTNFRF.[T]	4794.35268
10467	[H].LEISASGVEDLSRLPSRGEDHLETSASGVGDLSGLPSGREGLEISASG.[A]	4794.36642
10468	[D].VNWVNGGQENATPLIQATAANSLLACEFLLQNGANVNQVDNRGRG.[P]	4794.37649
10469	[S].SPGTRAPGNSSSSRPATAIPPATPTLATSAQPAAATPASVSSPAGSPGPPGST.[A]	4794.39291

10470	[G].PPATMLEQAQELFLLCDKEAKGFITRHDLQGLQSDLPLTPEQ.[L]	4794.41172
10471	[L].KQTTSFYALLTCSVIIGGFWLGVDQEGAEGTLSWTGTLFGVLASL.[C]	4794.43752
10472	[P].GWPQTVGSREEPLARQGELEAGKQQKRTKDPRAASGFILCTGPS.[T]	4794.44926
10473	[A].AGVAADWAAAGLADGARAAGHAGHGPAHGGLAGHGAAAAGVAVETGLEAASATAQ.[G]	4795.34321
10474	[E].EEHENKGQKEIQERKEAPSQTPEKSGGIEKVSPDQGLMTPVPH.[S]	4795.35916
10475	[Q].PGAGTVLAGASGLQQVQMAGAPSQQQPMLSGVQMAQAGQPGKMPSGIKT.[N]	4795.3634
10476	[P].PGPSPTLENGGAPSPGLPAEALGSGPESPRLDSLEAGSPRHPQRPETQ.[S]	4795.36703
10477	[P].AAAPDLDSLSSDLPATPEASLLGPDAVSVLSPGPSSGLDPDPGSALGSLPN.[P]	4795.36837
10478	[G].KTSFFLGEVGNAAKMMLIVNMVQGSFMATIAEGLTLAQVTGQSQQ.[T]	4795.38135
10479	[A].WPGRLTSEVDLKTAYPESGHTVFSAAGSVLSGGAQLPSEQSLSPPGK.[F]	4796.4166
10480	[A].QVNGNKSAPQGQDKKLLQDECKLQAAPHLQTPVDESHTVSSLSL.[K]	4796.42718
10481	[V].QPGAGQAGVVQPGAGQAGVVQPGAGQPVVMQPRMYPRGLVQPGMYPRG.[L]	4796.44465
10482	[T].KQMKFAASGSFLHHMAGVSSSKLSMSKALPLTKVVQNDAYTAPAL.[S]	4796.45723
10483	[Q].QSPVPVTPKSIRCTHQETFFKTPGSLGDPVLRGKERNQSRSSS.[S]	4796.46491
10484	[P].QASPAASKDQSPPPSPPPPPPPPPPPPTKKPEVAEGALSALETAEEPL.[Q]	4796.46691
10485	[V].EFLIYYSAVHTPSVVVEMGEPTTKPDGVLGDPAAMVTFYPNIPE.[A]	4797.33542
10486	[M].KEVCLGSPGTPGTPGSHGLPGRDGRDGIKGDPGPPGPMGPPGGMPGLPGR.[D]	4797.34063
10487	[G].SGRASMIRGTSASWDGQVGTVSALTYPIAHWHLVMSNLMILTSY.[L]	4797.35858
10488	[G].QGYLPQQLLGDSTHPQGAGRSGQNLLGQASATSHIYQGPESSLPGPP.[S]	4797.36155
10489	[I].LDNTVPGSPEERGLIQWKAGAHADSEMSSSLKSYDFPIGMSMVK.[R]	4798.27972
10490	[Q].DVAQLNITEQNWSPGQPSFLQSRDLRGMPNHIHMGAGPPPQFN.[R]	4798.30015
10491	[G].GSAGPGSPATLSPSAGVPQPVGMEALDQAEGPAASQRAMPPPPPASPPSEP.[A]	4798.30871
10492	[E].ASPDPVPEPPPPSVEAPDKPTGSPDQPPSPAQSPAPRPDAQAEEVAPP.[P]	4798.31186
10493	[F].VSEGAGPWGQPSHLLSPDWQFDITHLVADFMKLEEPHVATLQD.[S]	4798.32462
10494	[H].ITTSHSHTTVSPIHITTSPTHTTTGPTHTTASPTHTTTGPTHTTASP.[T]	4798.33031
10495	[P].GAAARGDAGAPGLGEQRGRRGAEDGMSQAPGAQPSPPSVYHERQRLE.[L]	4798.33233
10496	[A].PALSQVMSYQALPSTRLASQPHLVPTQQLQPQQNLQMQPPSMP.[P]	4798.41134
10497	[R].HVSAYGPGLSHGMVNKPATFTIVTKDAGEGGLSLAVEGPSKAEITCK.[D]	4798.41786
10498	[L].STPAASSIWSPASISPGSAPASVSMPEPLAAPSNASCMQRSVAAGAASAAA.[S]	4799.27094
10499	[D].LSSSPGREAKANSSTEETRQELASKPEVSKEATADPESNLESAAPL.[E]	4799.34535
10500	[E].ILDLNDNSPSFATPEREIRISESAALGARFPLDSAQDPDVGTNTV.[S]	4799.37586
10501	[D].RNSVPRRGDAMPEAEAGGVAGPSGGLREAAMEGWSAEEDSDIGPAEE.[E]	4800.14964
10502	[Y].AVMSRNSLQQGDVDGAQRLGRVAKLLSIVALVGGILIIIASCVINL.[G]	4801.72706
10503	[C].SPGPGAPAASMGAQVFSTVTAVSVLLLLVLSLLRLLRGRGCLLAPSKG.[P]	4801.73108

10504	[P].PGPRENQGPAPQGMMLGHPPQEMRGPHPPSGLLGHGPQEMRGPQE.[M]	4803.25077
10505	[Y].SKSLSDSLNTSLPMTSAVQNSTYTTSVITSSSLTSSSLSSTSPVATSS.[S]	4803.30993
10506	[V].QRPKEESSEDENEVSHILRSGRSKQFYNQTFGGRKYKSDW.[G]	4803.32583
10507	[V].PGGGEGPPKTGTAPSPGPPCPPVDGTSEGKGARHPKPSTSVTMETVPLE.[T]	4803.33526
10508	[P].GLPNGEKEDRFLTTLSSQSSTSSPHLQLPTSPEGVPEQAMGGPPEL.[D]	4803.34178
10509	[L].HQEILDIENAPRASMDLAKNAEDFVNSLFSNFPSVTTVWHLF.[S]	4803.35116
10510	[H].QSPQNHALQPHHHIPMVPAQQPMVPQQPMVPVPGQHSMTPIQP.[N]	4804.34797
10511	[M].PNLARMPSTTTVSSNGSSPVTVRNSQSFDSSLHGAANGISRIQSCI.[P]	4805.33297
10512	[D].GKDGTPGMPGVKGSAGQAGRPGNPGHQGLAGVPGMPGTKGGPGDKGEPGRQG.[F]	4805.3343
10513	[K].GERGPPGESVVGAPGAPGTPGERGEQGRPGPTGPRGEKGEAALTEDDIR.[G]	4805.35859
10514	[R].WNPVDFGNITSLRVPSEMIWIPDIVLYNNADGEFVVTHMTKA.[H]	4805.37421
10515	[S].ATVIFAGDTNLRDQEVTKCGGLPNNILDVWEFLGKPKHCQYT.[W]	4805.38142
10516	[P].PGTPTQQPSTPQTPQPPAQPQPSPVSMSPAGFPRVARTQPPTTVSTG.[K]	4805.39516
10517	[C].SRNPSGGTNLDFQIEQYKRKSDGIHIIRTWLLLLEPLLLVTA.[S]	4805.64648
10518	[G].PSAASSSGPAAASGPLATSSPAYSPGLSSPGQAYSAASASSCPTSSSSSSEW.[Q]	4806.12313
10519	[A].GPARTGSPCSRALAAPGTQNPLPLQSIPGAENRCEPGAPAPASSTTTH.[A]	4806.34347
10520	[V].NGVGSSGPSEYMEVPLGSLELPSEGTLSPNHAGVSNDTSSLETERGF.[E]	4808.21156
10521	[R].GPAAEEALNVFYYCTYEGAVDLDQVADERERKALEGIISNFGQ.[T]	4808.27845
10522	[S].QPQEQILQLCSPTTNTEQTFPGTNLTGFLPFVDNQMRNLSQD.[H]	4808.29306
10523	[L].MDLADVFTAPAAAPATDPWGAPVSMAAALPTAAPASDPWGGPPVPQAADP.[W]	4808.3011
10524	[D].PGVGIGRSQDPEAERGRNHDPEAGKENVGSDLVPAQDQDIGIEVEA.[G]	4808.31063
10525	[P].STPSFPVSPAYYPGLSSPATSPSPDSAAFRQGSPTPALPEKRRMSM.[G]	4808.3447
10526	[L].KLCSHTMMLPTRGQLEGRMIVTAYEHGLDNVTEEAVSAVVYAV.[E]	4808.35144
10527	[T].PSSLSSSQEILKDVAEMKEDLMRMTAILQTDVPEEKPFQPEL.[P]	4808.35663
10528	[M].GSFQLSKGMSGHLNGQARTSANKVMASKSTAFQNPMGIVPSSPKNAG.[Y]	4808.36651
10529	[T].HQQMRSLNPLGNNPMNIPAGGITTDQQPPNLISESALPTSLGATNP.[L]	4808.37304
10530	[A].AAPALCKPASKSMQEGWGSGGDDANLSASQWEDEDGGVWNNTASQE.[S]	4809.06999
10531	[P].STSSVATFSKEQQQMVQAFSTQSGMNCQWSQKCLQDNGWNY.[T]	4809.07086
10532	[L].QHQLDTSVSIEECVSKKESFAPGTMYKPFGKEAAGTMTLSQFQ.[T]	4809.28447
10533	[P].QGPAGPPGPPGPMGPPGLPGPMGIPGSPGHMGPPGPTGPKGTSGHPGEKGER.[G]	4809.30827
10534	[S].HGPSAPHPTSPTPPVTAGGATAAANGGTSNCLQTPSSTSRGRKMTVNGAP.[V]	4809.31798
10535	[L].QQGKPYHWRDWSIIRGRDCLYIWSDAAALELSNGSNGWFR.[F]	4809.32802
10536	[G].PGVSFSPGPTPTPAPTAGSFAGGAGGPSPPLFARPEAAHEPPFWDTPLN.[H]	4809.33723
10537	[P].AGPPGPPGPMGPPGLPGPMGIPGSPGHMGPPGPTGPKGTSGHPGEKGERGLQ.[G]	4809.34466

10538	[T].LQAAEGEAAAAAGAGAGETAVKVEGPGSPGVPGSPPEAAAEPPTGLRFSPEQ.[V]	4809.36021
10539	[C].RNPQEPPNVPGTNGIHVTGGSMTVTGNIYIYNGPVLGGARGPGDPPAP.[P]	4809.38017
10540	[V].ISQEPAMGIPSAVVPGSMAGRMTTTVAPGSIAGGMAPSLPPGSMIRGVGQ.[S]	4809.38645
10541	[M].PPGEEVKSTLSMLPMVLPGMAAVPQMFGVGGLLNAPMATTCASAVPAP.[L]	4809.38663
10542	[I].GTVTFEQSIEAVQAIYGLGGIGMGLGPGGQPIDANHLNKGIGMGNLGPA.[G]	4809.39746
10543	[Q].PAAATPASVSSPAGSPGPPGSTASLSTASLTPSSPRVPNVSAQGPTVQAPMP.[T]	4809.39997
10544	[Q].GAIGPHGEKGPRGKPGLPGMPGSDGPPGHPGKEGPPGTKGNQGPSGPQGPLG.[Y]	4809.40264
10545	[S].ADREMLKLSSQEKLNGAPWSGGSVADTPMSPLLKGSLSQELSKSF.[L]	4809.40736
10546	[F].WRYFAGNLASGGAAGATSLCFVYPLDFARTRLAADVGKGAAQREF.[T]	4809.41069
10547	[Q].APPSPPPASSLHDPPSLRAQPPQRGLPSLASSPLQAPTSPPASPPLQD.[S]	4809.49585
10548	[A].NPPSGLVNPSLPFTSSPDPTPSQNPLSLMMSQMSKYAMPSSTPLY.[H]	4810.27588
10549	[L].GINPFADGMGAFKLNPSSHELASAGQTAFTSQAPGYAAAAALGHHHHP.[G]	4810.29678
10550	[G].GPGSQGIQGPVSQGPLMGLNPRGMQGPPGPRENQGPAPQGMMLGHPPQ.[E]	4810.31812
10551	[Q].QPGFTSGTGHFTAMVWKNTKKMGVGKASASDGSSFVVARYFPAGNV.[V]	4810.35045
10552	[R].AAEAGGGCKDALQLLIEHSWERGEKLDMQALKQSSTELLFGGHE.[T]	4810.35633
10553	[R].RNSSSPPSPSSMNQRRLGPREVGGQGAGSAGGLEPVHPASLPDSSLAAS.[A]	4810.36738
10554	[T].YGLGVSSPPHGSPGLGSSQQSIMISPRHRGSPKMASHQFSPVAGVHS.[P]	4810.3689
10555	[N].PGPVGDPGERGPPGRAGLPGSDGAPGPPGTSLMLPFRFGSGGGDKGPVMAL.[R]	4810.38282
10556	[A].AAQAAINGVINTPELNCKGNGTSQATDLRIRRQHSSDSVSSINSAT.[S]	4810.38851
10557	[L].AAPSRRPCSRKTSCPLMRSNGDLSRSLSPSPLGSSAPSSIPERPS.[F]	4810.38938
10558	[D].HGLAGRGAAGDGPAAALLQAAGVAADWAAAGLADGARAAGHAGHGPAHGGLAGHG.[A]	4810.39183
10559	[I].LGQDVLLNEASLEVGESHQPYQTSLVIEETLVNGSSDLTTGSLAVP.[H]	4810.41566
10560	[T].RAPGNSSSSRPATAIPPATPTLATSAQPAAATPASVSSPAGSPGPPGSTASLS.[T]	4810.42421
10561	[S].QGRLSVGSNRDREISMSVGLGRSQLDSKGGVVGGTIDVNPLEMVAH.[I]	4810.43229
10562	[D].GLPGAPGDKGDTGPPGVPGPRGEPGALGPKGPPGMDGVGAPGLAGLPGPQGPPGA.[K]	4810.43696
10563	[P].APHSAFSLPGAAASLKDKAGTPTSGSPTAGTAATAEHVVQPKATSAAAAAPGG.[E]	4810.43946
10564	[S].KPDGDVAGTAQSLTEQMNKIALESGEQMESDNCSGGDDDWTHLSS.[K]	4811.06252
10565	[V].QMQVQATAVATAEAEARAEARAQMGIGEEAVAGPWNWDDMDIDC.[L]	4811.10764
10566	[S].RAGGEGPRGAEAAAAFLGMGFSSLDMSLCVLLYVASSLFLMVMYF.[F]	4811.30501
10567	[P].GPPGPMGPPGLPGPMGIPGSPGHMGPPGPTGPKGTSGHPGEKGERGLQGEPG.[P]	4811.32392
10568	[R].AAYLNLSQEPAHPAMALHARFAEAECLAESHQHLSKESLAGNKP.[A]	4811.34168
10569	[S].VASAHHGARTTEFNSAFPMAPPAEPSAVPVSQNSDVRKHGGPRDSK.[D]	4811.34552
10570	[A].VSSSQLNTIVTMNHHHPHPHHAAVGGVSGVVAGTGGDLVGFRHHENG.[G]	4811.34686
10571	[E].KWQEAQRPPAEVTALLSVLSGCGRGCDDRTAKKLMTHFSHLGS.[A]	4811.39267

10572	[D].KDIESDLYKMKHSVPQSLPQSNYFTTLSNSVVNEPPRSYPSK.[E]	4811.39851
10573	[V].ARAGEGPGGGQAALRATGNRMTLPAAHCLSPEGTSGASAVLDTLHRALA.[G]	4811.41764
10574	[A].EAEKSQLIMQAEAEAESVRMRGEAEAFAIEARARAEAEQMAKK.[A]	4812.33494
10575	[L].NPMTKHTDLPVSVFESVIDIINGEATMLFAELTYTLATEEAER.[I]	4812.36343
10576	[G].VKAAAPDCASTTGLGRQTVAAAAASAAVASEKQVCTQPVLNDLMPDIA.[M]	4812.39648
10577	[P].GNAGPVGTAGAPGPQGPVGPTGKHGNRGEPGPAGAVGPAGAVGPRGPSGPQGIRG.[D]	4812.45376
10578	[P].KGGPPGIDGKDGTPGMPGVKGSAGQAGRPGNPGHQGLAGVPGMPGTKGGPGDK.[G]	4813.40093
10579	[Y].QTILPAPPKPAGEALGSAGGPPTRSLSTTNSSSGSGAPGPSGLVRQNSTSL.[T]	4813.47149
10580	[G].PGMLGLDSEAQPQQQWGTVALAVERETWDPSAQPRDGPALGGTEAP.[P]	4814.31148
10581	[V].GTGDWRPPRPSTAAEPPTGTPVPPTEPPGTRDEGALGDGSPAPWPSQA.[G]	4815.30336
10582	[S].AGRPASASPAPNATADGSKTSRASVDTTPSVIQHRAMMRFSELEMK.[E]	4815.37233
10583	[G].ATSTGFMKAPASGAKSTPRMRAPASGAMSIPSSTAPISETVSVLQMTT.[P]	4816.3624
10584	[D].VAEMKEDLMRMTAILQTDVPEEKPFQPELPKEGRIDDEEPF.[K]	4817.33583
10585	[K].ADLNMTVTQASCLTLVPGSNDVVSLHSSTRGPSSTVSPPSCMTLIL.[G]	4817.34642
10586	[S].KETSSKAEEFPLPHVPSNPGSHSPQTRDEPANRRENLGHAQER.[K]	4817.34869
10587	[S].GLNLSPEQSIAAAFSAASWWPLGFTGAAHPLVWPSMRMAGRMWL.[Q]	4817.35779
10588	[A].TPPPGIMAPPPGMRPPWAHHRVFLPPTPETSTSMPPPGMRTPFT.[R]	4817.36116
10589	[Q].APNKTVQNDSGPAAPQPTTGPPASSPASESQNGNGLSTPPGPGGGPHPPHT.[P]	4818.27385
10590	[N].AASTAGPFPFHLSQHMLASQGIPMPTFGGLFPYPYTYMAAAAAAASA.[L]	4818.28295
10591	[Q].APGQQRARPSTTSSGPSQGPSGSTPRPSTTSSQGPALGQSPSGTTTPTQP.[P]	4818.32735
10592	[T].PQEQTPPPPQQQQQQQLQVTCSAQTVQVAEVEPQSQPQPSPEL.[L]	4818.32753
10593	[G].KDGQAGHPGQPGPKGDPGVSGIPGAPGLPGPKGSTGGMGLPGMPGPKGVAGIPG.[P]	4819.44067
10594	[V].ANALGNRCVRCEPTFINTNRSCACSEPNILTGGLCFSSTGNFP.[L]	4820.18546
10595	[R].WFEVVHMSQKMKGTIESSNKPDERGVSVAGTGEAEGLELHGHIQ.[L]	4821.33593
10596	[I].DGTGEGLRCKTRTLDSEPKCVEELPEWNFDGSSTFQSEGSNSD.[M]	4822.11152
10597	[K].FHTSTGLGLKNSVSCLNSENNVMENGCDSDSGDTDEIIAMKKSSG.[K]	4822.11827
10598	[P].HRNFQEEIEFLNAIFPNGAAYCMGRMNSDCWYLYTLDFP.[E]	4822.1258
10599	[A].QPSASLGVGYRTQPMTAQAASYRAQPSVSLGAPYRGQLASPSSQSAAA.[S]	4822.39655
10600	[D].QGVPGERGPAGPPGPQGPPGEQGPEGIGKPGAPGTPGQPGIPGMKGHSGAPGP.[A]	4823.37067
10601	[P].GKPGPTGPAGQKGEPGSDGIPGSVGEKGEPGLPGRGLPGFPGSKGEKGSKGD.[V]	4823.43471
10602	[V].AAGSPLMPEVGSPQDPGKSLPPPPPLGLPPPQEFGRGNPFMLFLCL.[A]	4823.43979
10603	[P].ALDRPAAASRPETYVATEYVALVPDGARAEAPGQLERMVEVLRAGG.[A]	4823.48972
10604	[S].PGSAALSTYTPENLLNKCRPGDLVEFVSQAQYPHWAVYVGNFQ.[V]	4824.3515
10605	[V].SETAEAQRMMPVNRTAKPFPGSGNQPAAPFSPSRNVTSPIADFPAP.[P]	4824.36208

10606	[V].GPGQTLRQEEDRHGGGQKQGPAKPGMAKHQGEVQSLKLDDDSVIE.[G]	4824.37179
10607	[D].VFLGPARCPAPYTFSFEMLVTGPCLLAGLESPSHALRADAPPHAS.[S]	4824.3735
10608	[P].QGPPGPQGHLGPQGPPGTPGMQGPPGPRGMQGPPHPHGIQGGPGSQGIQGP.[V]	4825.33351
10609	[P].AGPPGPPGPMGPPGLPGPMGIPGSPGHMGPPGPTGPKGTSGHPGEKGERGLQ.[G]	4825.33957
10610	[Q].FPSLDIEVDGGVGPDTIHKCAEAGANMIVSGSAIMRSEDPRSVINL.[L]	4825.35936
10611	[L].FNATKSWTSLGQLQSSSWNTPMQKQINQHLAFNINSKTSTVDS.[E]	4825.36385
10612	[P].KDVVGGPLPGDLQPGPSPESEDGSILSGVGPTHPSPRVGGFPGGGPPEPGS.[E]	4825.37038
10613	[P].GGRGPMGSPGLQGFPGITPPSNISGSPGDVGAPGIFGLEGYRGPPGPPGPAA.[L]	4825.37911
10614	[P].FPPGMFPLWPPMGPFPPVPPPPGSGEAAAPPSTSAAALSRPSGAATTTAA.[A]	4825.37929
10615	[M].PPQQHYAPPPPPPPPISHPMPHPPQAAGTPHLVYSQAPPPPMTSAP.[P]	4825.38063
10616	[R].VGGNFNRGSLKAPWAMGSRFWLQQSPKSFHPWPFQNTRAHSP.[G]	4825.38581
10617	[L].PGPSGNMGPQGPKGIPGNPGLPGPKGEMGPVGPAGNPGAKGERGSSGLDGKPG.[Y]	4825.38969
10618	[S].DILSPLEESYKKVGMEGGGLGAPLAAYRQGQAAPPAAAMQQHAVGHH.[G]	4825.39372
10619	[S].SMFVFGERRLPSGQAPGRAGPASLCPVLQRLWVGCGTWARGRAP.[D]	4825.4613
10620	[V].NALSFHPSGNYLVTASSDSTLKILDLMEGRLLYTLHGHQGPATTV.[A]	4825.46178
10621	[S].ICKAMETWLSADPQHVVVLYCKGSKGKLGVIVSAYMHYSKISA.[G]	4825.47004
10622	[P].SAPSASPAFGANQTPTFGQSQGASQPHPPGFGSISSSGALFSAGSQPAPPT.[F]	4826.27173
10623	[D].EPETSLPTQLKDNFNRAPSNQNWLTVNGGKLSTVCGAVASGMALH.[F]	4826.36248
10624	[P].DSGRPAPYSAAFLELQPGPAGSGYPAAAPPASFASHFLQGGPFPLPYP.[G]	4826.3678
10625	[D].SELSPFQAQRENGGLRVAPSMPVAPPATPAPRTCDGSPRGTPTSPAP.[E]	4826.37371
10626	[E].QGAGTPGQAQVRTVNTWAQKTASGWGQDREGLDVAPQMAATITVMAA.[H]	4826.37371
10627	[G].PTDLSQHQPLPAGGLGGSQPRLCGHPPPGDGGALTWGKPGWVRGSSW.[S]	4826.3757
10628	[T].SGGHNLSAQQTSARSASVSSAATTGLTTQQRTAIENATVAFFLQCIS.[C]	4826.37621
10629	[A].VDVMMTSSGKFLDHVLIEMGYGVKPKGQNSKKQSTDSGDLEDVR.[T]	4826.37976
10630	[S].EGQVPSESLGGRWPLAGPCPAVSLDATGGDRLWQRLEPGSHRGSVS.[S]	4826.38157
10631	[A].VGEGPSSPPHEVFVGEAVPTAAPRNVAVHGPTATQLDVTWEPPPLES.[Q]	4826.40604
10632	[R].TAAEQIPAHPLVTESMETVPPPEKVQPGPSQPTGLPAAPLPSPFPMA.[P]	4826.44195
10633	[H].PPASSSISIPGMGSRTSGPHGLGSPLVASPSLEKGLGGQSPQLGSRVSML.[S]	4826.49276
10634	[Y].PAAYPPLPAGPPSSSSSSSSSPPQQLSAATPHGINDILSRPSMPVASGA.[A]	4827.35302
10635	[Q].HMLASQGIPMPTFGGLFPYPYTYMAAAAAAASALPATSAAAAAAAAAGSLS.[R]	4827.36193
10636	[E].PSLREEEEVVTPDVQEPLVSSGEEESLILAEKQESQATPSHAPGV.[P]	4827.36944
10637	[G].GGGLTSLSNNPPLAQPTPENTAGAGDQPLPPGPAWGPRPSLSSSGDGRPP.[P]	4827.37211
10638	[G].GGAPPPLSEASSPLACLLQSLQIPPEQPEAPCLPPESPTSALEPEPA.[R]	4827.37433
10639	[-].MKNAMACDLVGIMNLYSAAGRLAKPFSIHFAYAGISEVNQPAELM.[P]	4827.37653

10640	[V].EPQQMGSDVRDLNALLPAVPSLGGGGGCALPVSGAAQWAPVLDFAPPGA.[S]	4827.3869
10641	[V].QNPKTVWFRQEDADVTFLVDITFFKNQSQLGQVMDYRSFL.[E]	4827.38755
10642	[Q].PGQGRAPEAPRKEGESRSQPPRFKTGGPGDELGAPDPPGAAPPGPGQTA.[E]	4827.39458
10643	[R].DLPGGAPAPLVPSSPPVTAVSQDMAPSASPKLEPATGPAMQAGGPGTPQGPA.[S]	4827.39679
10644	[L].VSPGDMKKSPVTADLAPDPDLGTLAALTPQQERPQPTGSQLDVSEPG.[T]	4827.3993
10645	[L].TVLSMASAKMESVSAALDGRATTAQSMAARGSALETGGVPWIKMVGTV.[C]	4827.41477
10646	[T].AVPSATREDSGSVPAPGPGQGMPVSLKRKSAGSMCITQFMKKRRH.[D]	4827.43857
10647	[R].DAVLYFSESLVPTARKALCDPLEEVREAAAKTFEQLHSTIGHQ.[A]	4827.44104
10648	[R].MVSVDRGSALSQLSFEVGAPMSPKMSTMGSPPPPQPPPPDLSTELY.[Y]	4829.31808
10649	[Y].EFFVLMNSVQNLPAPFSRENVLTFRQTCPEHSRTTLGVMML.[H]	4829.36703
10650	[T].EGPETKPVLMALGEGPGAEGPRLASPSGSTSSGLEVVAPEGTSAPAGGPGSL.[D]	4829.37856
10651	[V].WITDTTLRKSPFVPQMGDEVIYFRQGHEAYIEAVRRNNIY.[E]	4829.42567
10652	[E].AAEASRSNGASPEVRDARSPLSPNGSLENGVKAEGKEAKTANGHGGEVA.[E]	4830.37496
10653	[D].GEAETAQAREAAAPARAGWTLAGSGPVERASQPAVSGTCRPLSPAPLC.[S]	4830.37986
10654	[R].QGVRKAACAATSQLVSELTKEDAMTCVPAKMPKKSEEVPTILEE.[T]	4831.43484
10655	[S].MAVPSPGWVASPKTAMPSPGVPQNKQPPLNMSSSTTLGNMEQGALPP.[S]	4833.33546
10656	[S].PAMLASVESGGPPPPTASQSASVSIPGSLPSSTPYTMPPEPTLSQNPLS.[I]	4833.34851
10657	[Q].KGDPGTPGYPGKNGPMGTPGIPGTPGTMGPPGEPGVEGRYKQKHQSVF.[S]	4833.35118
10658	[P].VSMAAALPTAAPASDPWGGPPVPQAADPWGGPAPTPASGDPWRPAAPAGPP.[A]	4833.35183
10659	[E].SSLLGKDSPPTPTMYKYRPGHSSSASAAMPHSSSAKLSRGDSLKEP.[T]	4833.35706
10660	[E].AATKYFLTQSTASMLLMMAVIINLMFSGQWTVMKLFNPVASMF.[M]	4833.44229
10661	[V].QKKDPKDWAVQYQEAVQMQVQATAVATAEAEARAEARAQMGIGE.[E]	4834.3523
10662	[G].GASPEEPLAPEVSTPFPPLQPEPGGSGELNAVGSEDSQAAPKEAPGSRP.[R]	4835.32824
10663	[E].TVPYQSFPTHFTATTMMPRLPTISAQGSQPPGNAHFSVYNQLSQ.[A]	4835.33447
10664	[S].DGVMPVAPTSAVSSSGSPASVMTSIRAPSTTGSLGINSVTGTNTMNNVNI.[T]	4835.34959
10665	[L].PPMGAPPPLTQIPGMVPPMMPGMLMPAVPVTAATAPGADTASSAVAGTGPP.[R]	4835.36589
10666	[S].SESLIFSGSQGRGHQRPAPPSAAPSSHPPASSSISIPGMGSRTSGPHGL.[G]	4835.36665
10667	[I].QSYADQNKISILDFFRSLNPSGELMMPVGEFRKAMIQYFTF.[G]	4835.36701
10668	[G].GAENKEAGKTLDQVGQCMVASAAVVTTASSTPTTVRISDTGLATGTGPE.[K]	4835.36734
10669	[S].GSSGLLGSAGGGGGGGIGLGSLGGGGGLSSSLGGTATIGHLRGSSEHHFSNTLGSA.[S]	4835.36915
10670	[R].ATEKVPCSPLERCSSIMLLGMMARGKPEIVGSNLETLVNTGLDE.[K]	4835.37561
10671	[Q].LYSRTQEASPQVLEAFQNFYPTVGLSADMVAMLPKSGTPASPAHQ.[S]	4835.38075
10672	[Q].PRGSFYSVVPANQGWGDGPLSSRVAADASFTVQSAFLGSSVLGHLEN.[V]	4835.38122
10673	[L].KLPGGSIPQMLNGEVYPPSVEEAPVLMRYPEGIPPQSQMAVGQEV.[F]	4835.40927

10674	[T].APDLTAFGDPRQFPALPSISDPRMHYPGAFTYSPTPVTSGIGIGMS.[A]	4836.34364
10675	[G].MYKLNNMLSLAGMKVRKPTQEAYQNELKIESVERSFILSASS.[A]	4836.47327
10676	[S].VDAMVQVHLGAVFMPHGLGHFLGLDVHDVGGYPEGVDRIDEPGLR.[R]	4837.39774
10677	[L].QPWSGGHVLDGESALTTRHCRQGHGPLSCLARDSWAREDRPSV.[A]	4839.30876
10678	[L].PESLYPHTYNPKMSLDISAVQDGRLGNNRLLSAGSAMKDDEPDH.[M]	4839.3101
10679	[G].TPPTSGTSTPTFGQNTPAPGVGAAGGSLSFGASSTPAQGFVGVGPFGSAAPSF.[S]	4839.31728
10680	[Q].QRMKYYELIVNGSYTPQTVPTGGKALSEKFQGSGAATETTDSRR.[R]	4839.40063
10681	[S].QELSSPEQKAELAEGALHWDLPRVQGGSQLSGLFQMDVPGLPGPPG.[Q]	4839.40466
10682	[S].GPAVGAPLSMCSFRVGAEENLAPVPGPDLLNQGFLQSSWRGRECL.[L]	4840.37562
10683	[A].GKSPAARLEEEEALQRSGPHTQDAPGKDVPLSCTISGEKRPSEAPG.[E]	4840.39186
10684	[P].PTSGTSTPTFGQNTPAPGVGAAGGSLSFGASSTPAQGFVGVGPFGSAAPSFSI.[G]	4841.33293
10685	[P].ITMDLTPAEGTVPPTDQEMAPVKGVSSLSEIEAPLDEDIVSSTEIP.[S]	4841.337
10686	[D].PKEDTRSTAAGSLPFSASTPFPSSTLHTTDLSVSSPSHWITSSPATP.[S]	4841.35406
10687	[Y].VMIDDISELTKDSTSSTASESQRLEPLGPSSSGRPAKEPGEPGVLEG.[S]	4841.3633
10688	[L].PGTTSTIQTAPSTSTTMQVSSGPSFPITNYLAPVSASVSPSAVSSANGTV.[L]	4841.36733
10689	[S].GATSTGFMKAPASGAKSTPRMRAPASGAMSIPSSTAPISETVSVLQMTT.[P]	4841.39403
10690	[F].GKMYFPDVEFDIKSSKFKAEASLPSPKMGGEIQAPDLDISSPGIN.[V]	4841.40523
10691	[P].PGSQGQQGPPQGSLGPPPQGGMQGPPGPQGQQNPARGPHPSQGPIPFQQ.[Q]	4842.3302
10692	[R].HTQASLEPALSLSDRPGEEVCLSLGEGTSLAALSGPSHSLDTSSGHLA.[L]	4842.34866
10693	[D].VANAVLDGADCIMLSGETAKGNFPVEAVKMQHAIAREAEAAVYHR.[Q]	4842.37602
10694	[L].WFAHGRMTVRSSGSFILLYTDFGLQVRYDGYHLVEVTAPSSY.[A]	4842.37732
10695	[G].VYSNTVMVAWPASSWVVVMEAAMVARAAWVDMTKFYMKIQQF.[F]	4842.37772
10696	[M].KGDVSLNVSMGSNSQMIPQKMREAGAGPEEMLKLRAAGADMLPAQQ.[K]	4842.38276
10697	[S].SASANPAAAAAAGLGGNPAVAVPSSLSTPAASSIWSPASISPGSAPASVSMPEP.[L]	4842.38907
10698	[L].SVAFPEGIMARGVTNPIMPGGYALAGAAAFSGAVTHSISTALLAFEMTG.[Q]	4842.39396
10699	[Q].KNAPPSETSASSVSTSALDQPSSVPRSPLRNPAFSPVSSATSNGTKDK.[H]	4842.41404
10700	[L].QHALQASGQPSLQSQWQPQLQQLRDMGIQDDELSLRALQATGGD.[I]	4843.38163
10701	[I].GALPTAAGSPDSPGGRPGATMIAHEALKNHMKPMATGMRGAFGKPQGTV.[A]	4843.40113
10702	[M].APGCEPLAVRRMMDVLAPHVHGQSLAGAGGGGFLCLLTKEPRQKE.[A]	4843.43751
10703	[R].GEWLAVPCRDAQLTVGWLGREAVRRYIKNKPDNGGFASVDDAR.[F]	4843.45976
10704	[H].APSFNGNGLEGSMTLTKAPGPRPNSLVSSAGSEYAERPEVARRDSAP.[A]	4844.36565
10705	[G].QSGAESSLGPLNLSTGLPPTVWGHGWPGAPTNCSALPRTLGVMSELGS.[E]	4844.37706
10706	[G].PPGPEGPAGFPGPPGIQGNPGPVGDPGERGPPGRAGLPGSDGAPGPPGTSLML.[P]	4844.38492
10707	[S].KFTDTVSTQALSPAVSTLSQGNIKEHPLLASCESEDNICQLIEI.[K]	4844.39685

10708	[Q].GGSHRPPAPTRIADSCALTSGKQEPALNQAVNSYVHPQAPHLYPGP.[S]	4844.40739
10709	[V].QPGAGQAGVVQPGAGQAGVVQPGAGQPVVMQPRMYPRGLVQPGMYPRG.[L]	4844.42939
10710	[L].LRWFEQNLEKMLPQPPKISEGWSDERTDAPLGPEAPGPASEIK.[P]	4844.43523
10711	[A].PPPSGSAVSTAPQPKPADKMSKNKKKKLKKKQKRQAELLEKRM.[Q]	4844.74411
10712	[D].PSTQVPVVEGSGEQDFTFDVSGENAAGAAVEPGSRNGAPGDPEATGATGAS.[Q]	4845.19941
10713	[G].RAGMGPVQGAGWDAPLGPVWGQHPQLLTASAFLPPKGEASRYIFLT.[K]	4845.50861
10714	[I].GGVGQGLPPGSVIGGVGQGLPPGSVIGGVGQGLPPGSVTGGMGQGLPQGSVIDSV.[A]	4845.52036
10715	[I].ERGFLSDTPVGVAHFILERKGLSRQMIGEFLGNRQKQFNRDV.[L]	4845.54818
10716	[Q].PPTWAPTQPRGPAAIMEVSTLEAGETQDGGGAADDDAATVQQHLELM.[-]	4846.25707
10717	[S].VAPTPTAVSGGGSTSSTSSGSFEASAVEPQVPSKEGPEPPEEVPSPATPPA.[P]	4846.3065
10718	[G].QLEVLECLWPRGASEPEYAEILSFPNSLRSQASAQPCAHLRH.[T]	4847.37807
10719	[G].RAGLHCAQAYPVRTTGQELPFAYSGQPGSGGLASMAGDADLIDSLLK.[N]	4847.38796
10720	[E].FGFLLDHVQTARSLNRSSFTYYPDPSFEPLGPSGVLDIKPGSHV.[V]	4848.44203
10721	[N].AAQLAAAAQAMQTININGVQVQGVPVTITNTGGQQQLTVQNVSGNNLT.[I]	4848.49085
10722	[G].GGPGTATGLDAGGLGPAGNAASTAGPFPFHLSQHMLASQGIPMPTFGGLFP.[Y]	4849.35012
10723	[V].MIDFKPAFSKDDILPMDLGTFYREFQNPPQLSSLSVDIGAQSM.[A]	4849.35617
10724	[T].SAASDRRDSELSRKEKADDTTPSTGLVGGSYEALVSAAVPEHGPPHT.[S]	4849.36233
10725	[Q].KDMVVMLLSMLEGNVVNGTIGKQMVDMLVESSNNVEMILKFFD.[M]	4850.38669
10726	[D].PWGAPVSMAAALPTAAPASDPWGGPPVPQAADPWGGPAPTPASGDPWRPA.[A]	4851.34127
10727	[M].YSAMSPFGKATLDPSKLYVSSSFANKIPDEGDAATEKSEEPSALSK.[Q]	4851.3557
10728	[D].SGGPVQPKYSQLFCGLPSLHSESLVDTFLGYQGLSMNGSMSKHPL.[K]	4851.35791
10729	[G].AGMGFGLERMAAPIDRVGQTIERMGSGVERMGPAIERMGLGMERM.[V]	4851.35881
10730	[P].KGDPGFQGMPGIGGSPGITGAKGDMGLPGVPGFQGQKGLPGLQGVKGDQGD.[Q]	4851.38288
10731	[D].QGTLSPPFTQPGGMSPGIWPAPRGPPPPPRMQGPPSQTPLPGPHHPD.[Q]	4851.40349
10732	[P].QTLSSFYSSSRPATASQRSPSKHGGPSAPGALQPLTSGSAGPAQPGSVAGA.[G]	4851.40447
10733	[E].KLEQSEAQLGRGSFMLGLETHDRKSEDKLAKATRDSCKTTIEA.[I]	4851.43637
10734	[S].IFPFGEGFERRLESMQGVSELSGSAQLLCPTVVDGFLSDLDVHP.[L]	4852.35968
10735	[A].AGVAADWAAAGLADGARAAGHAGHGPAHGGLAGHGAAAAGVAVETGLEAASATAQG.[G]	4852.36467
10736	[T].PGTPASLSANSSLSSSGELVEPSVDQTPQASPGLAPNTRGSPGPPPAKPC.[S]	4852.36939
10737	[R].PPTTTAAEPQPTAPPTVCVTGPPTARPSEGPTTGPTGPPAAGPTGPPTAGPS.[A]	4852.37342
10738	[H].MPSGKTATPEIVDNKDGTVTVRYAPTEVGLHEMHIKYMGSHIPE.[S]	4852.37428
10739	[L].ALDGQTPGTGRLGQAMFTSLLCSKLDPLFTEFKEEPWAPEAEIT.[V]	4852.38483
10740	[G].RPAIPPKMSRSSPGGSPVSPSTSPLYDLSEGNSGVPGPQPPSRGPADPA.[S]	4852.39588
10741	[A].PAANTASSAFLQLPSEADELATFSAKPEASDEKPKQADPPSAQPNQT.[Q]	4853.3388

10742	[P].PTGEGKGGPTSHPSPAPTPSSAPSPLGGSALCGGKPEAGESPPPAPGTPKANG.[S]	4853.34351
10743	[K].AQLSPGIYEDASARRTPGSYASTVSRGSPMLSRASEVTISSGKSANH.[E]	4853.38711
10744	[G].KSSGSSSQKTTPEGSELNIPHVVAWAPAPEDAGLPHGRDTTQLLASE.[V]	4853.39766
10745	[G].GPLSVYPGAGAGGGGGGSSVASLTPTAAHSGAHLFGFPPTPPKEVSPDPSTTG.[A]	4857.37547
10746	[G].EISATGVGVSAEGEMSGVVTVRVYSPHGRLSEPVSPDAALGSSSVAQVM.[P]	4857.40334
10747	[P].GPRGPPGISGALATYAAENSDSFRSELISYLTSPDVRSFIVGPPGPPG.[P]	4857.44824
10748	[S].RSRGRSRSSSCSRSRSKRRSRSTTAHSWQRSRSYSRDRSR.[S]	4857.48044
10749	[R].RQRRRRLEQEDASSRMAEVTVRVTPAAGGGGTWGRERFARFGP.[S]	4857.51632
10750	[P].QQGMVPHGLHQGVMSPPQGLMTQQNFMLMKQRGVGGEVYSQPPH.[M]	4858.32552
10751	[A].QAASYRAQPSVSLGAPYRGQLASPSSQSAAASSLGPYGGAQPSASALSSY.[G]	4858.36669
10752	[G].PSLESTSNGRHSASSPKAPDPEGLARPVSPDSPEIISELQQYADVAA.[A]	4858.37659
10753	[L].AAPSSPSLSHRQGMGPLGTGFHGNTVSSPQSSAATTPGSPSLGRHPGAHQ.[V]	4859.3415
10754	[-].MNREAAAKSQGKGSSGSEIYERLTPGQPGNQLYVVGTSSMSLGQQK.[T]	4859.36868
10755	[L].QREAAGGVTMSTIGAESIRDEEAAPGQAAVTVRGGADGKTVTMSVPGAAM.[T]	4859.37205
10756	[Q].RSFRHDVRGNARAMMKLMNGADTAKHSLSTLGSANCFLDSLYE.[G]	4860.31852
10757	[G].ALIGSSGAILSYIMCVAMNRSLANVILGGYGTTSTAGGKPMEISGTHT.[E]	4860.40387
10758	[D].DVVPDNKQVQLCHVGAAVEPKYVSFCALMNATVDVLDNVCKNK.[T]	4861.37799
10759	[P].RPGTPQSPPTCRVSPPESRGTQSLLPSDSPQPLAASPSPWGPEAVAGG.[D]	4861.39622
10760	[S].SPSSLSSGSVFTIAPQLQAFLVPKSSASSSSAFPSVAGTTATSSLPSFGQ.[A]	4861.44181
10761	[E].PRGDVGEPGAGSEVPPVGSQADPVSAETLISEELAAMTLEKHDSESAE.[D]	4862.27964
10762	[R].ERGAAGAGSDDTDSGSSVVLVGGAAGSESPGRVEANVPMVPAPAEEPGARNG.[V]	4862.28818
10763	[E].EGQGLTLAASCTAEGSPAPSVTWDTEVKGTTSHRSFTHSRSAAVTSE.[F]	4862.29221
10764	[A].SVDAALAEASVEAADLESLVRGPSPPACPDPTHGEHPAPVPPALDAGHS.[A]	4862.33261
10765	[P].RSDPVSPTMARSRDPEKDYRSKEEMAVAADAAALVDGKGGAGVGQAE.[L]	4862.3432
10766	[E].FLDLDNHNAAARRQGSLSASECLYGAPPAPLGPGMAFGSPAFPPHTV.[M]	4862.3566
10767	[L].PGVGTFYNLGTAVYYAVQNCPDVAKARGRDGVIDLGYDLLTAMAGP.[S]	4862.39165
10768	[Y].TAQNSDELNISAGDILEVILEGEDGWWTVQRNGQRGFVPGSYLE.[K]	4863.34964
10769	[V].PSVGSLADPDYLNTPQMNTPVTLNSAAPASNSGAGVLPSPATPRFSVPT.[P]	4864.4098
10770	[S].QPGILEESSTVPGSPVSLFREQGSGEAAVDPETTTVPSLSLNLEPEI.[L]	4864.42622
10771	[A].WVSAPPTWIPLCRLYPPWLFADPDVVSLEAADRPNFFLHVTA.[N]	4864.47485
10772	[S].GAVGNYSGGVGVKEWLLAHEGSPVGKAARGGGSRATGSWRGALGGTASSQP.[T]	4865.45785
10773	[G].LGNVAMGPRQHYPYGGPYDRVRTEPGLGPEGNMGTGAPQPNLMPSN.[P]	4866.29335
10774	[S].SSINPGIGNVSASSPAQQGLGGQAQGQPSSANMASLGAMGKSPLNQGDSSAP.[S]	4866.30172
10775	[P].SPGQQVHTPQSMPPPPQPSPQPGPPSSQPNSNVSSGPAPSPSSFLPSPS.[P]	4866.3064

10776	[P].QGPAGPPGPPGPMGPPGLPGPMGIPGSPGHMGPPGPTGPKGTSGHPGEKGERG.[L]	4866.32974
10777	[M].HQASSRELAFAEDKKKEKQFLNAESAYMDPMKQNGGPLTPGTSP.[T]	4866.34616
10778	[A].IDMWSLGCVIAELFLGWPLYPGASEYDQIRYISQTQGLPAEY.[L]	4866.34699
10779	[A].QGVLASQALSQGSEPSSENANDTIILRNLNPHSTMDSILGALAPYAV.[L]	4866.42143
10780	[S].GPARSIPGYPSSPLPGSPTPPMTPGSSIPYMSTSQEVKSPFLPDLKP.[S]	4866.43687
10781	[G].QGPPGSSGPPGVKGEKGFPGFPGLDMPGPKGDKGSQGLPGLTGQSGLPGLPG.[Q]	4866.45194
10782	[E].GPKGDRGPQGQPGLPGHPGPMGPPGLPGLDGLKGDKGNPGWPGTPGAPGPKG.[D]	4866.46451
10783	[E].RGLPGLSGSGSSSLGLNLQGPPGPPGPQGPKGDKGDPGVPGAPGIPSGPSRGGS.[S]	4866.48814
10784	[A].VCEPLAVPAASPMAAAAEGPQQSAEGSASGGGMQAAAPPSSQPHPQQLQE.[Q]	4867.23562
10785	[L].KAAQADVQEKLSCTSKHLAECQAAMLRKDEEGAALHQDLDRTQ.[K]	4867.35199
10786	[S].PQTSPMLGSSIQTFAPSSQEVGSGIHPDEAAEKELTSVVAENGAGLVGS.[L]	4867.35783
10787	[H].QRIQDEKYSQSLRRGSEDFEKRSSFQRRYPEDHDFRKY.[G]	4867.3796
10788	[H].REDLPTGESTTAKSTPAQSTTAKTTVHTHVPATSHSQGEKNSHPSGL.[S]	4867.38413
10789	[L].PGSLPLGGCGSTPPTPTGLAAASDKREGSSSSEGRGDTDKYLKKLHTQ.[E]	4869.40718
10790	[G].PAGAAGGAAGGGPAAGPADHGLAGRGAAGDGPAAALLQAAGVAADWAAAGLADGARAAG.[H]	4870.41162
10791	[F].SNRPPGYPSQPVEQRPLQQLPPQLVQHVAPPAQPPPQPPPPPPSQ.[P]	4870.55398
10792	[G].EPGDPGPPGVTGSPGLKVHVKVVLKGQQGPAGSVGPRGPPGDIGLDGNPGAP.[G]	4870.55985
10793	[S].VSGRLDMSSSKFTVTSVGPSISQVSVDLPISLDHRCPSVSTTSSSY.[Q]	4874.38227
10794	[A].PSVMGAGPAGPSSQAPGTVIAAFIRTSSATAAPGVKEGPLRPSSYVQGMM.[T]	4874.42738
10795	[S].SPSTPGVSKMTLGAPSTLPNFSAVSVSPAGGKQTQQRPTDMSALNNLF.[G]	4874.44514
10796	[S].QQAQLQEFGQSSRKGEFPGGLMGPVRMISSGHELTTDYDEKALH.[E]	4876.34174
10797	[Q].QRAPGGKAAAGREPDPVAADGSHLYAEAAPGAPPHREPAPGAGPPREAGSG.[P]	4876.40106
10798	[H].GTPFQREPVGPSSAPPAPPKDHGGIFSRDAPTHLPSVDLSNPFAKEA.[A]	4876.44415
10799	[P].QTYTQHAIQVQHIQVTEPPAPAVSTSQVTGQPLSPSPQQSQQGLSP.[S]	4876.45003
10800	[D].PADGPPDLTGWLHSLAGSTLPGLLRQADPQHVMEYSLALIAVLNEP.[P]	4876.49783
10801	[L].LNSVVYGSERTMLSQQVGSVKWPNSVMAPGRGPERGGGGGVSDSGWQ.[Q]	4877.34822
10802	[G].QPGVDSPSPSLMPASAQAQNAQRALSSVVLPSQGPGGGSELSSAHQLQH.[I]	4877.38711
10803	[N].SPQFAGQQPQFLAKVGPTQPYIPQRTYTRSYYPGSGGFGAISSAGN.[C]	4877.40704
10804	[G].DSKNNVRSTALATVNAWAEQTGMKEWLEGEDLSEELKKENPFL.[R]	4878.38907
10805	[T].VCFKMKHDFKNLATPIRPMEETDQGAEVIWLTQHVELSLGPL.[L]	4878.47796
10806	[T].IEHGDRTGSLFAFMPEFYLSVAINSYSALKNYFGPVHSMEELP.[G]	4880.33749
10807	[A].RGQWPWQVSINHHGTHVCGGSLVSEQWVLSAAHCFPRDNKIE.[E]	4880.34335
10808	[S].GPGHGPSTEQLDILSSILASFNSSALSSSVPSSSTPSGPHTTATPSVTASA.[L]	4880.37083
10809	[G].TPAGSQPSSPRYRPYTITHPSGSTSSPASRSSGTSILSSSPGLYAPASS.[P]	4880.37217

10810	[R].GEEARAPPTGEPGSALFPGPAMGTAAAVLLSRAWPCASSPSCRARLVS.[R]	4880.4029
10811	[C].DRSSQGTPFGASPLAPASQPNSLADVGSLLGPGASAGGIPSSIFGMAGQVPT.[L]	4880.41595
10812	[G].GEGVYHVPHGSTTSLKSTEGGAAGTTSGLAIAGTTTSPGGGVAVAGVGAGSVGGAV.[S]	4880.42969
10813	[Q].PGAPSMAGTVAPGGVSGPSPAQLGAPALGGQQSVSNKHLAWSGVLEWQEK.[P]	4880.44244
10814	[S].ALHPQAQGQSQGQPSSSSLTGVPSSQPMQLSQQQQGVQPTAPSPQAAQ.[Y]	4881.34564
10815	[R].RSQNFYFSERDSVIPSSNLVMPAFQDVSLSFPQYSLPQQENL.[M]	4881.34647
10816	[A].QPASDGESDTTLTRSRVPAAAPAASWRHCPPRSAQSSGDARLAASPH.[P]	4881.35821
10817	[A].PADGPAGPGPEALCFPAPQAQPDGGGLLAFSVQDGSPPGLDLDSSPVLQD.[W]	4882.31523
10818	[F].KADPNSSSFKRPDGEMNIASGCPRFVAHSTLENAKNTYIKDDTL.[F]	4882.3523
10819	[T].PGQGGSIDSGPMPPESAGPVVAGVFSAIFVLVALMLMYRCCKQNNK.[L]	4882.38572
10820	[T].EAATKYFLTQSTASMLLMMAVPRATEAATKYFLTQSTASMLLMM.[A]	4882.39177
10821	[S].ESSKLSDPEKTSSEHIPYLSPYHSSSYRYANIPAHARHYQSY.[M]	4883.30845
10822	[G].AAAMKQEHSNVAANPRQHQRSHEDPAALRFNFYEVITTGHWAP.[S]	4883.37201
10823	[P].LIPDSVRQYMSSSSRNPSFLWPFLERKLKRLRTQGVVFESL.[E]	4883.61414
10824	[E].VGLENRGEMKNGREAGAEFLTEGDRLGSGSSDASEEEEEEGEKEGP.[P]	4884.16204
10825	[R].QRWAWGAEAARQAFPFSENVWCGPRPCSLHRGHGECPSGQSC.[V]	4884.16844
10826	[S].ALTTGGPSLSAMGNGRSSSPTSSLTQPIEMPTLSSSPTEERPTVGPGQQ.[D]	4884.36259
10827	[S].APKNMATSTPVARGGGMPAPFPKNTPSKTFPPECENQKDPSVNTVV.[V]	4884.37535
10828	[G].SLGAAGRGRGAGMPYPTPAMQGATSSVLAETLTQVSPQMASHAGLNTAQA.[G]	4884.38256
10829	[V].VTNVSGSVSSAGRPASASPAPNATADGSKTSRASVDTTPSVIQHRAMMR.[F]	4884.40753
10830	[A].WTQERTMKWLVLLGLMALSECIMAYISNINIGTPPQEFWVV.[F]	4886.44282
10831	[Q].RMPSVPTTSRQPALLHYLQQPTPPPASSATASSTATATLQLQQQPD.[L]	4886.47413
10832	[E].APTGQEAALRAGRGTSPSRPDPRPSVEGMRLTPAPPAQAKAAHSGGETP.[P]	4886.48268
10833	[M].RDLPSQLFEPSSNLGPNQNSAVIPANSLASLIKTEVNGFVHVHMGT.[H]	4886.48939
10834	[L].GGPPGEPGLPGIPGPMGPPGAIGFPGPKGEGGVVGPQGPPGPKGEPGLQGFPGK.[P]	4886.50867
10835	[L].QSKMTLDQPHVEVPGQNKASKVTSSVVGPGHEVQEQSSGPFKKQSA.[T]	4889.44865
10836	[N].FPANVMDVIARQNFTEPTAIQAQGWPVALSGLDMVGVAQTGSGKTLS.[Y]	4889.46006
10837	[F].EKVENKMSGSSRKGCLWALNLARIDKMEEEMHKWKRKDLAA.[I]	4889.47938
10838	[A].PGFSGPKGEPGDILTFPGMKGDKGDLGSPGVPGLPGLPGTPGQDGLPGLPGP.[K]	4889.48184
10839	[G].SVVVEHELIMEANYTSGYLELFENLTKIVRAKIMNETGQLHGN.[S]	4890.48046
10840	[G].GAPQAVPLSHYTFRKTRRMRDRCIMATLPGIDQAVFQWETGL.[P]	4890.48651
10841	[S].APVSMLTTVTMLDPGSSAPGGTTPISSKTSSKLPGKPDSKEGTSGVLTVS.[N]	4891.48025
10842	[G].VVPPLQPAFVTAQSYPVANGMTYPAPNVPVVGITPSQMVANVFGTAGH.[P]	4891.49499
10843	[F].KHPGDFYVQLYSSEVLEYMNQLSASLKETYANKAHEEDYVPV.[K]	4892.33999

10844	[C].KVVNATGFCSVPTATPTPTNSTAKTTTLPSTTTTSTTATTSGTTNTTLT.[P]	4892.42049
10845	[K].KPMFGKMYFPDVEFDIKSSKFKAEASLPSPKMGGEIQAPDLDIS.[S]	4892.42353
10846	[E].EQAGKDVTLRDGPRSTPGAQAAPSTARSPQDPAPPELGSVPPSSVEIGN.[L]	4892.44092
10847	[S].SSPPSPSSMNQRRLGPREVGGQGAGSAGGLEPVHPASLPDSSLAASAPLC.[C]	4894.3959
10848	[Y].SPQVQTPDQQPNRTNGGDKLRRSMPNLARMPSTTTVSSNGSSPVTV.[R]	4894.42826
10849	[G].KQQGPTEAEAEEVAELEEAELEEEEEEQDWGSTPDNSQLPGELP.[G]	4896.15012
10850	[R].PEGGYKAVWFGEDIGAEADVVVLNTPASEAGGAGDSGSEGSGDEAADAQDA.[P]	4896.15146
10851	[K].QPDELSLQVADVVLIYQRVSDGWYEGERLRDGERGWFPMEC.[A]	4898.33011
10852	[C].PSPPTIANGHHTGESVASFAPGLTVTYSCEPGYLIFGKNTIHCLSL.[G]	4898.39165
10853	[R].DRFRSRGGGGGGFHRRGGGGGRGGLHDFRSPPPGMGLNQNRGPMGPVP.[G]	4898.40606
10854	[E].PQITPSYYTTSDAVISTETVFIVEISLTCKNRVQNMALYADVSG.[K]	4898.41144
10855	[P].LFSYNNGVVMTSCRELDSNRSALSAASAFAIATAGANEGTPNKEKY.[R]	4899.33123
10856	[-].MGPASPAARGLGPLLRLLPLLLLLLRVQLAEGNLAGGSPSAAEAPGSAQV.[A]	4899.76047
10857	[A].EQKEAEFQQMLEQLKKQQEEAQAAAAAASAESRDFSGAGGIGVFSE.[S]	4900.33301
10858	[H].RYSGEDRVLSAPELLEFLEDQGEPKQHELMTLDGFMMYLLSP.[E]	4900.35182
10859	[S].QSSPVDSSTVDGKNAEEKPKTQEGNLNLKDLSMQKRIQFFETC.[A]	4900.37276
10860	[P].RDAPAAGSPGSASRGRQRFHPNQYLPSFYAVDLSEPQKAGAGDGSAR.[R]	4900.40106
10861	[N].GRDYRSGEPVGSGDPCSHCHCANGSVQCEPRPCPPTPCRHPGR.[T]	4901.08855
10862	[V].PVGGAPSSGMLMDKPHPPPLAPSDSTGGSHSVRKGYRIQADKERDSM.[K]	4901.38798
10863	[Q].GPASEHKTPWPLMETLDAQLAAQSPGMGRGPCAPPLEAPLHPMGLGD.[P]	4902.34702
10864	[S].VENPMALLGGDALKFSEMFQKDLAARAMNVDPSFWNQYAAAITNG.[L]	4902.36881
10865	[M].PNLARMPSTTTVSSNGSSPVTVRNSQSFDSSLHGAANGISRIQSCIP.[S]	4902.38573
10866	[H].TACQRSKGMLVMDCHRTHLSEEVLAMLSASSTLPAVVPAGCSSKI.[Q]	4902.38613
10867	[T].WDTEVKGTTSHRSFTHSRSAAVTSEFHLVPSRSMNGQPLTCVVS.[H]	4903.36387
10868	[R].NSTLSFRNALEGFDKADGTLDSQVMSFHNLVHSFLNGTSALPHSAA.[N]	4904.36967
10869	[A].PSGPGSVQKYIVVSLPPTGEGKGGPTSHPSPAPTPSSAPSPLGGSALCGGKP.[E]	4904.48872
10870	[A].GAPSSLGNPPLYRSSLSHLASQHQSSGLSATSTASASLPSGSASSPRSVPA.[T]	4905.43617
10871	[W].SALQYEEKEVRKHLWECVTKKMAPFLETTGDRLPEDCPVKS.[K]	4905.43722
10872	[D].SLSSDLPATPEASLLGPDAVSVLSPGPSSGLDPDPGSALGSLPNPNPIPTSG.[S]	4905.45277
10873	[T].AVPMTTSSIFSRGPETTPSLVPSPGVETSTAVPMTTSSIFSRGPETIP.[S]	4905.45364
10874	[N].FPANVMDVIARQNFTEPTAIQAQGWPVALSGLDMVGVAQTGSGKTLS.[Y]	4905.45498
10875	[N].MAHVTQAHVQTGITAAPPPHPGAPHPPQVMLLHPPQSHGGPPQGAVPQ.[S]	4905.45765
10876	[M].GKTSFFLGEVGNAAKMMLIVNMVQGSFMATIAEGLTLAQVTGQSQQT.[L]	4905.46574
10877	[P].GAGGLSPSSLPASSFALGGGLAADLSLHSFSDGASLSHKAPEAAGLGAPLSFP.[G]	4905.46937

10878	[P].MPGKAQMQAPPLPAQPQTVAPTRPPVDPTQSCLRPPPSTSTTSTVAP.[A]	4905.46958
10879	[I].PMPGKAQMQAPPLPAQPQTVAPTRPPVDPTQSCLRPPPSTSTTSTVA.[P]	4905.46958
10880	[A].PGFSGPKGEPGDILTFPGMKGDKGDLGSPGVPGLPGLPGTPGQDGLPGLPGP.[K]	4905.47676
10881	[E].QRGGDKILLNACCPGLVRTNTAGPKVPKSPEEGAETLVYLALLPSD.[A]	4905.56011
10882	[V].DAASEDLELYVSRNTDVLTPTPDSSPRSTSSPSQSKNGSFTPRTAH.[I]	4906.33618
10883	[R].AFEGRLQEHEHRVEALQEEKLSAGSEGSEAVQRLEQQLEMKEA.[S]	4906.40242
10884	[G].ASQASGPSLGPHFPLPGRGEVWGAGYRSHREPGPGAKEEAAGVSGPAGGR.[G]	4906.42688
10885	[S].SPSTPGVSKMTLGAPSTLPNFSAVSVSPAGGKQTQQRPTDMSALNNLF.[G]	4906.43497
10886	[P].PQQHYAPPPPPPPPISHPMPHPPQAAGTPHLVYSQAPPPPMTSAPPP.[I]	4906.43848
10887	[I].SVSSIPSAASVSVGPAVSSGVNVNILSGMGNGTIASSAALNSAASAAAGMTVGSV.[S]	4906.44084
10888	[F].CGHLAAVGGAVGAGLMGLAGGVVGAGMAAAALAAEAGMVAAGAAVGATGAAVVGGGVGAG.[L]	4906.44305
10889	[F].LYDVIESLAVGDNDFRFALVQFNGNPHTEFLFNTYRSKQEVL.[S]	4906.44751
10890	[V].QKTVDETSFQKVGKKSGSRSFKKEKKRSPSSASESPTSTYEQLL.[E]	4906.55449
10891	[S].PGVTASWIEANLPDDSKDTWKKRGSVDYVVLLDWFSSAKDLQLG.[T]	4907.48904
10892	[T].GTVGKNSLSGIAVNVPASRGSNLNSSGANRTSLSGGTGSGTQGATKPLSTPH.[R]	4907.49541
10893	[T].QEKPRDVSSVELLMKYHQGIRAEIETRSKNFSTCLELGESLL.[Q]	4907.50299
10894	[N].NPYNTLLGEPAVCNNPSVSMYNTQEGLLNNARDTSVMDTLPLNGN.[H]	4908.28732
10895	[P].KTPMEMLYHHVHRLNMSGPFGGAVSAAGLTQMPAGNVFTTAEGLFS.[T]	4908.33646
10896	[V].PAGAVSTPEEPDTPNVRMLTPEEPAAPAGAGSTPEELATPAGAVPTPEQS.[A]	4908.33676
10897	[F].PGMQPLEMVKPQSGSPYQPMSGSQALVYEAPLSQAAGLGASQMLNSQ.[L]	4909.31512
10898	[A].ISEGRGTQREENPLEGNILAGEAASRAGNSGNEAASKGDGSDVPSQTPQ.[T]	4909.3179
10899	[S].KPEEDLQDPGEAQGPIEVPLLEAEVGESASHLASYYPASSSAPMEAL.[P]	4909.32479
10900	[A].SVPAASGYPLGPEAPQEAGRSAGRAQGSRLTCTFDAASKQLGLEDPTH.[S]	4909.39219
10901	[S].SRTSHPETDILHRQAYAAPHPLQSYATNHHPAGLSGLFDTGLHHA.[G]	4909.40542
10902	[A].VSRGVPGGSGDQANPRGPSAAGESGGAAGAIPQILGAPHAPGPGGDAAPGAGVLSN.[R]	4909.43242
10903	[G].RGEPAPGASSLLEAELLGAWWCHCLYGRQGASVPPPGEPPQALCPA.[Y]	4910.35997
10904	[K].PLFPSAGQMGTPVTSSSTASSNSESLSASSKALFPSTAQAQAAVQGPVGTD.[F]	4910.36364
10905	[A].ARPTMPSGEWAPQSPAVRVTCAATSNAMNRPIQGGMIRNPTASIPM.[R]	4910.37393
10906	[R].MSAPRNYSRSGGFREGRTGFRPVEAGGQHAGRSGETVKHETSYRS.[R]	4910.37486
10907	[S].PGAGGGVNERRRHAHSAPSAHPGMAAAQGPVAPSSPEQNGAVPSEATKKD.[Q]	4910.38476
10908	[P].AFFSPATAAPEPGASPRLAVDLTLPEELPLVSSHEDTDGEPEEAVGPP.[Q]	4910.38944
10909	[M].GPSPTQHRSPSGRMRGEVSWAQMTASLLSVPPPSSSCRGPGVPAGPQA.[S]	4910.39955
10910	[H].SAPASPSSASKEVGIGFAQGPGASASTAATPGPAGLPRGYMAPTSPAASERSP.[S]	4910.40136
10911	[S].APASPSSASKEVGIGFAQGPGASASTAATPGPAGLPRGYMAPTSPAASERSPS.[P]	4910.40136

10912	[L].QTLQAAEGEAAAAAGAGAGETAVKVEGPGSPGVPGSPPEAAAEPPTGLRFSPE.[Q]	4910.40789
10913	[G].VSTSLSLSSHGGGSPPAPSQAAIDTQAGASPNSPGVDFGEMRGASPTELSK.[A]	4911.33374
10914	[G].PGMLGLDSEAQPQQQWGTVALAVERETWDPSAQPRDGPALGGTEAPP.[L]	4911.36425
10915	[P].CAFIPRDIQTPGSPGQGEPIPSEVRENSLPSCSLHTSTPKSPEPGP.[I]	4911.36762
10916	[A].VSTPEQSATPAGAVPTPEQSATLAGAVSTPEEPATPAGAVSTPEEPATPAVS.[T]	4911.39057
10917	[Q].SFLTAIMQSMARKNEWPLDKMCLSVEVTKKNREDMTAPPREG.[S]	4911.40824
10918	[A].PRVTTGPTPFSNMPNAAAVAMAATLTQQQQPATGPQPSLGVSFGAPFGSG.[I]	4911.41926
10919	[A].GQVEPTSLKNMDPVSAGEVGSVSLAKVDPVSSGKPEPLSPMQAELMSV.[G]	4911.43119
10920	[M].GFIGFFVKLIHIPINNIIVLIKHSRVAIPRGSVQLEQSALGNFVG.[V]	4911.82399
10921	[S].EGTKLPNSMMTSTLKAQGGPSRSTASSSMATTANPSKQNQSAPAAAPFT.[S]	4912.35098
10922	[Q].APGPSTGRTTSSEPVGQAEATGRLQSLANGPSNGSSSRQRTSGSGFHRE.[G]	4912.36652
10923	[D].QFHRMVELTMAARQAYRTMLESARQEPLGELGPCTPASPPSQGP.[S]	4912.37497
10924	[L].KMVMHARSGGNLEVMGLMLGKVDGETMIIMDSFALPVEGTETRVN.[A]	4912.3844
10925	[Q].VETGVAKMNTINAAEPHIVTVTMNGVAGRNHGINAHAATTQYANGVVP.[S]	4912.45811
10926	[L].APETGDLWAALGQRAPGGSPGSAGLVQRLEEYAATLARNMELTYLNP.[V]	4912.46865
10927	[P].PGLNSEQQPPQPPPPPPPPALPPSSPTNPGGGVPAKKAKGGPNASGSSATIS.[K]	4912.5228
10928	[T].APTPVGRATPPPGIMAPPPGMRPPMGPPIGLPPTRGTPIGMPPPGMRPP.[P]	4912.54316
10929	[G].ALSPAFTFPHPINPVAYQQILSQQRGLGSAFGHTPPLIQPSPTFLA.[Q]	4912.59372
10930	[W].ERERLSDRWYPSDVDRHSPMAEHMPSHHSSEMMGSDANLDSD.[Q]	4913.07914
10931	[T].LAEGQAFAAGESLGEGQGLAEGQVFAEDEPLQEDESSDDDMSVNEEL.[V]	4913.12252
10932	[T].MIVSHELAHQWFGNLVTMEWWNDLWLNEGFAKFMEFVSVR.[V]	4913.34655
10933	[S].RMEPGGPTSALGTTPTPASASPEGLKEESGDLAASPASPGSPPNSDLVPLD.[L]	4913.3633
10934	[G].GLGAGYGGAGGGGFGGLGIGFGGGSGGGSLGILSGIDGGLISGSEKETMKNLNDR.[L]	4913.41226
10935	[R].RDASMLNDELSHINARLNMGILGSYDPQQIFKCKGTFVGHQGPV.[W]	4914.42364
10936	[W].NAVTQTRESGKAIIITSHSMEECDALCTKLAIMVKGKFMCLGSP.[Q]	4915.3953
10937	[L].PGPPSISVQLPSVAAVRGYQPCAGYGGGRRQAGPRGGLALQPGQLSDQS.[Q]	4915.51325
10938	[L].HLLDQVFCTRLTEAGIPSEVTTGIFSNISSIYRFHGHFLLPEL.[Q]	4915.52398
10939	[Y].LSKPPKDWQPLISPAGTEPAYIEYKTSKEGSVMGVTVSRLAMLSH.[C]	4915.53725
10940	[A].DPTRATTASTTKDEAPLPDGPGPVAGQTLVAKKAVVIKQEVPVAPAEPP.[S]	4915.64152
10941	[L].PGSPGAKGEQGPAGHPGEAGLPGPSGNMGPQGPKGIPGNPGLPGPKGEMGPVGP.[A]	4916.38427
10942	[G].KPELQNSASQQMLSFPDKGKEKPADMQNFGLRTDMYTKKNVPS.[K]	4916.40156
10943	[A].AVGGNSGEARGAPTPHEKALTSPSWGKGAELLLGDQPDLMASLDGGAKSD.[G]	4916.41193
10944	[L].QGPPDSPSLGIARHLRGDASASSSSSSSSDNELAPFARAKSLPSSPVTH.[S]	4916.41577
10945	[S].ISPETRYTLENSQKTSTAAVPMVTTVEPATLQSMATGSGRSSLPHSP.[T]	4916.44045

10946	[R].SPVVGFDPHHHMRVPAIPPNLTGIPGGKPAYSFHVTADGQMQPVPF.[P]	4916.45519
10947	[V].GGAAGLNGQCEWLSRLQNGLVPNQYNPLRGGVTPGTLSTQAAGLQHGT.[V]	4916.46088
10948	[S].APAAAPFTSSSAANGLESSVATDSSKLATITTPMALNTSGIDVKSSGSMTT.[T]	4918.38199
10949	[-].MAMGVAGPASLYHSGLTVGMISGGGVVCVQEARAGYVGSLGCLHLGSLGN.[S]	4918.39293
10950	[P].PGTFRTEPGASSQDDCELCPPGHYCPEAEQSGHANVFATPCRAGT.[E]	4919.09372
10951	[L].PGELQSPTGQMIADESGHGQKGTLGGFSVQMNEPASYGVEILLSPMSS.[H]	4919.31722
10952	[E].NGIDIIMADRTFHLIAESPEDASQWFSVLSQVHASTDQEIREM.[H]	4919.32509
10953	[D].PQFRTCLDTEAGWPLPALAKGYTPAGQWDRPTEDWSLLGLTSCG.[N]	4919.3556
10954	[H].ATLSQQGNLESPSGSGVLSSGSSSPLYSKTVDSTQSPLASSPSSAHSGPSN.[S]	4920.32534
10955	[L].GEVGAEEEEDGQDEEEEEAGAEDEAAEDSRPGTRGSSSPSSQPPGPHP.[H]	4921.01827
10956	[D].MLAHVEELARDGEAPLDRAGADEEDDEEEEEEEPDQDPEMEHV.[-]	4921.03306
10957	[T].ANGVQFLPEQPLAKEAADPPGSTEETQPLEGLKGSEPPQPGGKDGAPGAG.[G]	4921.41264
10958	[P].RPPTTTAAEPQPTAPPTVCVTGPPTARPSEGPTTGPTGPPAAGPTGPPTAGP.[S]	4921.4425
10959	[E].AGPAGPKGEAGEMGLSGLPGANGPKGEKGESASDKLQESLAQIIAEPGPPGP.[P]	4922.44764
10960	[A].KYESKIQMYDEQVTSLEKTTKESGEKMAHMENELQKMTSIAN.[E]	4924.33591
10961	[T].FVANRHKDNLNNYVFQGQHPLTLNESNPNTVEVAVSTESKGNSR.[P]	4925.43136
10962	[L].FFNRGAPVRAGAAPSELDQMNPGQAQAAGAKRGWPPAAWRDSTFVR.[M]	4926.45059
10963	[P].RGPLNPDSQRMPMQPSGSVPVMVSLQGPASVPPSPDKQRMPMPVNT.[P]	4927.41439
10964	[D].GAPGQKGETGPFGPPGPRGFPGPPGPDGLPGSMGPPGTPSVDHGFLVTRHS.[Q]	4928.39616
10965	[V].EDLGVLPSGEGHLEISASGVEDLSRLPSRGEDHLETSASGVGDLSGLPS.[G]	4928.4032
10966	[L].EGSMTLTKAPGPRPNSLVSSAGSEYAERPEVARRDSAPAESRPAKSD.[V]	4928.41914
10967	[G].PGESRHEPLGLPMESYQPWALPNGWNGQMYCPKEQAQPPHLW.[K]	4929.27591
10968	[T].RWLNSPNTYMKVNVPEESRNGETSPRTKITWMKAEDSSKVSSG.[T]	4929.38942
10969	[Q].AAQREPFCGAKKTAVEEQCLLGAGVLGDKKQASAVRPRRVLRPPGG.[G]	4929.65266
10970	[P].KGGPGPGSGGGAGTGAGAGGPGTGHLPPGAGTGPGGGAMFWGHQPSGAAKDAAATAGPP.[S]	4930.30986
10971	[L].PGKQEGGRASGPSGRGAPPTMEQQRPEPEQLPPASPATPEASDPSVSGH.[P]	4930.34089
10972	[A].NPGAMLELGPPHGVSAEEAGLGPQMAGQPLEAEEDRFVAPQQALQGHV.[D]	4930.38869
10973	[A].SRGSNLNSSGANRTSLSGGTGSGTQGATKPLSTPHRPSSASGSSVVTASVQS.[T]	4930.42337
10974	[E].GPAGFPGPPGIQGNPGPVGDPGERGPPGRAGLPGSDGAPGPPGTSLMLPFRF.[G]	4930.44819
10975	[R].NGTSSNWKEVKQDFKMESPSNSALMLPSTPQAGANPPSPHSSSSRK.[Q]	4931.3323
10976	[V].GMPSPVSPKLSPGNSGNYSSGASSASASGSSVTIPQRIHHMAASYVQVTS.[N]	4931.36868
10977	[T].VSPEERMQRIEGRGMEKTREEAELETDSIFRQKVEVCYQR.[M]	4931.38329
10978	[A].SRRGSFVNSSGVMNQGVAPMVGTPAPGGSPYGQQVGVLGPPGQQAPPPYP.[G]	4932.43083
10979	[G].PQSLGILMTSIPDSQAATRRQAVGLLAFLGFLFCLGVAMFAYQKL.[Q]	4932.59768

10980	[V].AGPMLPAGNAQQRTSGPAPAPPQGAPQPGLSGLSPAGPELGAFSQSPAPAMGG.[R]	4933.39959
10981	[T].LENGGAPSPGLPAEALGSGPESPRLDSLEAGSPRHPQRPETQSPAAPGPP.[L]	4933.44634
10982	[Y].LEYRMVPNSDPSRAVASPAGSRASSTRAARDGTEGARHPEARPSAPE.[Q]	4934.40589
10983	[I].KMGISASTMTLKKEGPGEVTDKTDAVMTSGQGLENEPITVISNTAGSH.[M]	4934.40677
10984	[W].GNTRPSTLLPPSDQALNGEEKAEASKGVPAPPHSSSPAQGRAERQENA.[P]	4934.43756
10985	[T].PGYPGKNGPMGTPGIPGTPGTMGPPGEPGVEGRYKQKHQSVFSVTRQT.[V]	4934.44648
10986	[S].ANVPRQEDELEPETPALALPVTPQKEWLHMDTVELEKLHWTQ.[D]	4934.46692
10987	[V].GARSAAPGGGSVAAASAAMGAALASMAGLMTYGRRQFEHLDVTMRRLIP.[P]	4934.47569
10988	[V].LSPAVTCGPTGLLLCRPVILTVPHCAEVSAGNWIFQLKTQAHQGH.[W]	4934.53788
10989	[R].ATTHTHFLPPTTSKGTTQVTKLPIRPSETTTRPPVTTLSTGQATSST.[G]	4934.58579
10990	[-].MSVPESGPRPPAAPAPFPPGPPMMPPPFMPPPGIPPPFPPMGLPPMSQ.[R]	4935.40658
10991	[G].LMGEKVTGERGSCLRALGHGGGSDARSCRRDPLVSEVRQDPGDPLA.[P]	4935.4119
10992	[S].GLNPQGYHPAFPVAQGAAEALGHSFLDRASPAQGLPLDTAGGGHERGGVS.[W]	4935.43096
10993	[T].GRAHSQSTQGRGPQAPRMTYSQAIRRSGQQSTHEAQATEGAQLQAT.[K]	4937.39163
10994	[S].GGGDKGPVMALRGPPGPMGYTGRPGPLGQPGSPGMKGESGDLGPQGPRGPQG.[L]	4937.39921
10995	[G].PQRGLEEGSPVSSVEDVVIDLSSSTRQETERVQQGAGAQGRPDSEGL.[E]	4937.41074
10996	[A].APTFPTPNTDIFPEPQGQAFPGSAGPALQYPPPAYPGAKGGFQVPMIP.[D]	4937.42835
10997	[A].QPAAATPASVSSPAGSPGPPGSTASLSTASLTPSSPRVPNVSAQGPTVQAPMP.[T]	4937.45854
10998	[S].EQTTGRDLTLGGSPGTTDTLPKTSIEPTILATLTSESQAMTSLTNASE.[G]	4938.42596
10999	[M].NVYLMMLITSGAWALFGVWNAIGSIIYFGYGIRHSLAGNNYQQP.[P]	4938.45346
11000	[K].SGAPPTPGRTSPAVMQPPPGMSLPPADIGPPPYEPPGHPTPQPGFIPPH.[V]	4939.43345
11001	[V].SKMSVSRSSSLRSSSSLSSQGSVASSIGSQTSFRSTDFATPGHPKYW.[S]	4940.38677
11002	[S].PGRGGRGTQEVASTPAASLPSSFCPHPASSSSSPPDPAISPALSAPPPAYA.[T]	4940.3908
11003	[I].PGGPPSSPGSRKLSAAGSSDGVMPVAPTSAVSSSGSPASVMTSIRAPSTTGSLG.[I]	4940.43643
11004	[A].GEAAHPLPHTFHRLLQTISDLMMSLPSGSSLQQMALRCWSLKF.[K]	4940.44668
11005	[G].QRGPVGLPGSPGAKGEQGPAGHPGEAGLPGPSGNMGPQGPKGIPGNPGLPGPKG.[E]	4940.49727
11006	[Q].NNPLPQGFQQPVSSPGRNPMVQQGNVPPNFMVMQQQPPNQGPQSL.[H]	4941.36176
11007	[P].VACGAVMVPSAMLMGQVVTAYPTFAPQHPQSQTLSVTQQQSPQEQP.[L]	4941.36782
11008	[G].ASRGPSAVEYNKEDRESFRHSQQRSKSEMLSRKNFAAGVPAVSM.[D]	4941.42312
11009	[V].LQHAVAGDPQSVVAAIDRYSSEKEWAMHVGDKKGGWRGGSGAGPAGGPP.[-]	4941.42377
11010	[I].SDGGTPVLATNISVNVFVTDRNDNAPQVLYPRPGQSSVEMLPRSTAA.[G]	4941.47995
11011	[M].GPSPTQHRSPSGRMRGEVSWAQMTASLLSVPPPSSSCRGPGVPAGPQA.[S]	4942.38938
11012	[Q].AQRENGGLRVAPSMPVAPPATPAPRTCDGSPRGTPTSPAPEGGEGPPSPL.[S]	4942.43229
11013	[D].VLCPVYDLDNNVAFIGMYQTMTKKAAITVQVGNAPGPLGDAASGHHT.[T]	4942.43247

11014	[P].SPAVDPPTPGPGHPTHPAPPLLGSVTNRRGDDNAQAGAAGMLTGLQHPGAA.[M]	4942.44015
11015	[P].TGPHTPFTTQSSATFSVHTTSHTRALPTGTSSRTTTPHSTPSHPETL.[P]	4943.3943
11016	[S].GVSAAGGGPAGAAGGAAGGGPAAGPADHGLAGRGAAGDGPAAALLQAAGVAADWAAAGLAD.[G]	4943.41677
11017	[F].ASTFVGGELYTGLNADFLGHEAMIFRTGGPRPALRSDSDQNLLHDP.[R]	4943.41695
11018	[P].SPHSPALSSQMFFPTPLSLPAYCQRAHFVFSGPHFVAVNNKNEI.[V]	4943.41847
11019	[P].GTPAPSAGPPDRTSPPLFQSRGSSPLQLNLMQLEEAPEGPAAATGATGTV.[G]	4943.44798
11020	[L].KGDKGNPGWPGTPGAPGPKGDPGFQGMPGIGGSPGITGAKGDMGLPGVPGFQG.[Q]	4944.38321
11021	[S].GPSQPGQPGAGTVLAGASGLQQVQMAGAPSQQQPMLSGVQMAQAGQPGKMP.[S]	4944.38593
11022	[S].LHGVDMAANPRAHPDRPACSAAAAPGHALGRDEAAPLNPGMYSQKAAR.[P]	4944.40636
11023	[S].PPPEDLPMSPPPEVSRLSPPPEESPLSPPPEESPTSPPPEASRLSPP.[P]	4944.41355
11024	[E].EYRSLVSGGATQVTGTTGAMVTSDTFRTTRAAGAAREVAGARAGAGAVSEA.[F]	4944.46167
11025	[L].PGMSGKGAQLFAKRQSRMEKYVVDSDTVQAHAARAQSPTPSLPAGW.[K]	4944.46319
11026	[H].PSGPMQGVPRGSSMGVRNSPQALRRTASGGRTEQGMSRSTSVTSQISN.[G]	4945.42861
11027	[E].SVIESALDDLDLNEFGVAALEKTFDNSTVPHPGSITMGGSLLQSSAPV.[N]	4945.42993
11028	[L].PGANGPKGEKGESASDKLQESLAQIIAEPGPPGPPGPPGPMGLQGMQGPKG.[L]	4945.44587
11029	[R].CLVNFNILVEDKMNLYRVEVEIMDINDNAPKFLTEEMNVKI.[M]	4945.44942
11030	[Q].ATATSTVTSVSTASTSTDSAPKPAFSFGVSSVTSTLSSVTSTTASTSQPFL.[F]	4946.41645
11031	[L].RGEGAAGAAGMKRAMSLSMLNTDGSRVPENQFPRQHLSEASRTLSAS.[T]	4946.41665
11032	[L].SARTYSVDGPNASRPQSARPSVSEIPERTMSVSDFSYSRTSPSKR.[P]	4947.40382
11033	[G].RWQDVSVGSWNQPPRLGRQMSDGVGEKLFQDLYPFMLGEHGLT.[S]	4947.40937
11034	[C].GAQGKTSCFLRMLSPFELVQYSLETEEPLRDSHGLCIPARPGEA.[G]	4947.42263
11035	[R].QQPKASDTAAVGGEVPADGGPVGKEDRGTQPLQVLSVPRLQRKLKEAA.[R]	4949.65554
11036	[A].MADTSLYSVPPAAEREAPADAKRLSASSTGSTRSSQSASSLEAAVPGRE.[P]	4950.41338
11037	[I].EIDNPELSLILDGQEWRYFKDIADLHTPLEEDMFHLRGNLAP.[Q]	4950.44071
11038	[E].KEILPSRCLGYSNSETLMEVDIVEQSLVAVLNSGGQNTNVKNIGAS.[D]	4950.48232
11039	[K].NNGICSERWGGFSCDCPVGFGGKDCRLTMAHPHHFRGNGTLSW.[D]	4951.16639
11040	[S].QELSFLNTGVPRSQSSPENVGDTRGALSPVTDTTEVDTGIHVPSEDI.[P]	4952.4032
11041	[P].PGGPPPPHSPYSGPPSRGSPVRQSFRKDSGSSSVFAESPGGKTRSTGGSS.[T]	4952.40587
11042	[S].THLTTTQTSGATSTGFMKAPASGAKSTPRMRAPASGAMSIPSSTAPISET.[V]	4952.41867
11043	[S].QPSRSSSPGPVAVEGCPGGSRMKPVSSSRPAPLPSVFSVPGLSSLNWM.[S]	4953.44443
11044	[E].DRPSSRAVLYADYRAPGPARFDGRPSSRLSHSSGYAQLNTYSRAP.[A]	4953.46399
11045	[K].IRDTSQAGTQLWRQSQDPGPTLQSSASPDSIPAENSLPQGDAQPQPP.[V]	4954.3838
11046	[A].LDAWMLPMGDEVYSRIPQPLFFINSERFQYPSNIIGMKKCF.[L]	4954.42276
11047	[C].PHPVTLAGMLEMGVSYLPVNQNWERYLAEAQSTYEELQREMK.[K]	4956.36411

11048	[L].GAKTEDLPEGNKTISENASATAAPKMPESAPVSAPVPSHEFETTGGHVP.[D]	4957.37962
11049	[Q].KNLESSTSFQIPSQELASQIDPQKDVEPRTTYQIENFAQAFGSQ.[F]	4957.41264
11050	[T].MNTRNVPQQSLPSPTSAPPGTPTQQPSTPQTPQPPAQPQPSPVSMSPA.[G]	4958.40473
11051	[-].MRAGGASEDLHPRDCVTGRGLCILNFAQEPLSPGKHHAGREHKPGM.[V]	4958.42201
11052	[G].PRGMQGPPHPHGIQGGPGSQGIQGPVSQGPLMGLNPRGMQGPPGPRENQ.[G]	4958.42201
11053	[G].PAARSTATPKCQSLGGPAAAYATGKASGAGGAGGQAYSPGQPQGLLGPQAYGQ.[G]	4958.42383
11054	[F].NHSLYRTFVPAMAAIHGPPIQCVPWGLAGGRAGPQPGQGVSMAASSRA.[P]	4958.45119
11055	[L].CILNFAQEPLSPGKHHAGREHKPGMVDTAKKNFGGGNTAWEEKTL.[S]	4958.45771
11056	[D].GAPGPPGTSLMLPFRFGSGGGDKGPVMALRGPPGPMGYTGRPGPLGQPGSPG.[M]	4958.46511
11057	[L].GQAQAQAAAATTATGTQPPGKHRGGLRSQANSSSQTQAPGQQRARPSTTS.[S]	4958.46724
11058	[G].PEPSPGQVGQGGSALASPAPRPPASRASKMWDAVLYRMSVAASRGGQAGP.[G]	4958.49007
11059	[Y].APPPAAFPPREYWSEPYQLPPPTPGLQEPRAPGPGADRGPWGGAGRL.[A]	4958.49138
11060	[Q].IGFMLCSKNPSTNFREPLQPLMQKQVEEMQLKYYNSDVHRA.[A]	4959.40487
11061	[H].QKGDPSQPDGKPESPVGTAHAQLQRQQATDYCQEQQGKLLSPRSL.[G]	4959.44021
11062	[L].RWEPPADLGEREDVRYNVECSQCQGAALDGGPCQPCGAGVHFSAG.[P]	4960.16789
11063	[E].EQQKEERLLRPSRMLTFLFGMRQLPMGPSSPSSLSEFVTHPPA.[H]	4960.49065
11064	[A].PGAPGFSGPKGEPGDILTFPGMKGDKGDLGSPGVPGLPGLPGTPGQDGLPGLP.[G]	4960.51896
11065	[S].ASEPGPQRLDVEPEVPASSSTSITMATRKELEEMIENLEPHIDDP.[D]	4961.36668
11066	[G].RAGLHCAQAYPVRTTGQELPFAYSGQPGSGGLASMAGDADLIDSLLKN.[R]	4961.43089
11067	[S].QAPFPSGSPTLPAQAQAGQMMPLSSARPTSGSVGVMLAAGRNEALQVPAP.[R]	4961.47065
11068	[E].VHTQSRVEPSAPWCLRARDSGSLAPQCGSVLELSRGHSPGAGSPPGPG.[A]	4962.42345
11069	[A].RPAYEAVDGTREANNRLVTYVGTSERAASDGKATGMAPDIAGLNLPGGG.[R]	4962.43987
11070	[S].QRPGAAEWPAEQPRGPPRAALPQPQTPDQGGPGPRSHCGLSQSPERG.[E]	4963.42656
11071	[Q].PGSPGMKGESGDLGPQGPRGPQGLMGPPGKAGRRGRAGADGARGMPGEPGVK.[G]	4963.46969
11072	[G].QPEAGLGETSLKCSPLSMTRSLQGQYLLKTCCSSLASGLPETLPPT.[S]	4964.45122
11073	[M].NRLGIWGEGTPFREFSDFIQAVERRGVGAMEIVAMDMKLRGMY.[I]	4965.44193
11074	[E].ELLAIVRTDMARQGGDPGLMASARSPQRLADTFESKPDSFFLEPL.[M]	4965.48734
11075	[P].LSESPVEEFQYIRAQDSAGAEGLGGGGVGKWGGLSWMGEGSGVAALSSPL.[S]	4967.37923
11076	[I].GPSILNSDLASLGAECLRMLDSGADYLHLDVMDGHFVPNITFGHPV.[V]	4967.3801
11077	[A].ASAERERERERKERERLAAASSDLYLRPGSEQPGRPSSHGYVRSP.[S]	4967.49675
11078	[K].KICMQRFIKIDGKVRTDITYPAGFMDVISIDKTGENFRLIYD.[T]	4967.56203
11079	[N].LPDTPGQSPPNSTTELPFWPTPAPDPTGHVEWHSSLPMRDTPPAPL.[D]	4968.39376
11080	[E].STVVPHGDAPGLWGGPEQIPTPPTSPSSGSRSGTYTKVSEPQQALPPES.[K]	4968.42862
11081	[W].CPRDPLVFSAASFDGWINLYSVMGRSWEVQQMRQADKISSSFS.[K]	4969.34947

11082	[L].GPAGNAASTAGPFPFHLSQHMLASQGIPMPTFGGLFPYPYTYMAAAAAA.[A]	4969.35751
11083	[A].GRTPVRARAPVLPRKEGVDQRGSSPTSHPRPGAPPSASSAASRAPPESS.[A]	4969.59641
11084	[Y].PGAGAGGGGGGSSVASLTPTAAHSGAHLFGFPPTPPKEVSPDPSTTGAASPASSSA.[G]	4970.38274
11085	[R].NPIAKEYDEFPTSYAKADIDEESIEGTSELGDAVSHFTPSRQSQI.[S]	4972.3283
11086	[S].GPRGQAPDTLSYLDSVSLMSGTLESLADDVSSMGSDSEINGLALRKTD.[K]	4972.3674
11087	[L].KMLQATGGKGYFDAHALAMDFMSIGFRECLTEVARYLSSVEGLDS.[S]	4972.37766
11088	[G].DPGEAGPAGPKGEAGEMGLSGLPGANGPKGEKGESASDKLQESLAQIIAEPG.[P]	4972.41165
11089	[A].APGPGPAADWTTPGDPAVSRDEIGSGGAACAPALALYAEPPAGPLTMGGASAL.[A]	4973.37204
11090	[L].PKPTMPPAGPQHDTSLAMAVIRREVSRIHGEDVAQPSPGTEVPQEK.[H]	4973.49964
11091	[T].AELVVIEDAAEPKEPAPPNGSAAEPPATEGSREENQVGPEAPAAEPQDL.[D]	4974.37631
11092	[T].AENALQAAGSRCLSLASSEGSDARLSERAHAVGSVGISYRDGPSPSGLC.[F]	4974.38171
11093	[A].HPTVKGAREPSKTSALGSGEDAIEPVSPPEGMVEPGHPRGTMYPLLY.[R]	4974.44006
11094	[G].PQGEPGPPGQQGTPGTQGLPGPQGAIGPHGEKGPRGKPGLPGMPGSDGPPGHP.[G]	4974.44523
11095	[G].QPLYGPGAAGADAAVPSTPAGQDLLSSGEPQPLPSSPGAEPRTPSRAPPAGE.[L]	4974.45042
11096	[E].RRDSSTSTVSSAYTVSRRSSGISPYFSSRRSSEASPLGAGRAHPASSA.[D]	4974.45495
11097	[L].RQQGHPTPFDFLGRAGSPRGSPLAEGPQAFFPERGPSPRPGTAPYDA.[P]	4974.45712
11098	[S].GIMYFEQAPLLPQSVGGTTAPAAGTATISQDTSHLTTGPVSGLASGSSVLN.[V]	4974.46771
11099	[E].PGSKGDRGEPGQRGQNGIPGLPGERGVAGPEGKPGLQGPRGTPGPAGGHGDPG.[A]	4974.48144
11100	[Q].WENSFVSVYSKDNPNLLFNMCGFECRILPKCRTSYEEFTH.[K]	4975.27353
11101	[T].GPGPSPGPAPPNYSRPHGMGGPNMPPPGPSGVPPGMPGQPPGGPPKPWPEGP.[M]	4975.36539
11102	[D].NLLQEMLLGGSSSETPHAHHPLHPHLMQEHMGTNVIVANTMPAHL.[S]	4975.38587
11103	[R].PGTIQGLEGSADFLGPQGIRGYPGMAGPKGETGPQGYKGMVGSIGAAGSPGE.[E]	4975.39892
11104	[E].SPKATSAMVISSTIRDTSVSTSTPDSSKMAQIETGPTSSLTAGLRDTST.[H]	4975.43582
11105	[D].PGVGGTGLEQGPSAGAASAGPQVSLYQGAPPAAEQGVVSRDLRVWEVETGD.[S]	4975.44567
11106	[G].TPQSPPTCRVSPPESRGTQSLLPSDSPQPLAASPSPWGPEAVAGGDLPV.[P]	4975.45306
11107	[L].DKMVQRSGPSGPPGPKGDDGIPGQPGLSGPPGPKGEPGHPGTDGAAGQRGPPG.[L]	4976.40924
11108	[L].QREAAGGVTMSTIGAESIRDEEAAPGQAAVTVRGGADGKTVTMSVPGAAMT.[I]	4976.41465
11109	[S].SPSGTEERNRLTSTGVASAPRCRSPFPPINVPGMVLYSEDEKEEI.[D]	4976.4153
11110	[L].PGSGLTTTRSGDVVYTGRKESATAKASSEDAGPSPQARATKCPAEEPVT.[A]	4976.42903
11111	[D].REQNQVLARDTQSQESDKKVESASTGRGMETVKVEIETPKETQE.[R]	4976.45016
11112	[P].IADFPAPPPYSAVTPPPETFSRAVSSPTAGPAPPPPWPQPAPWSQPAF.[Y]	4976.47227
11113	[L].GEAHRHEPPVPHDKVVVDEGQDQEGLEKERPSKHVDERALGGKGQ.[M]	4976.47462
11114	[L].GPSGRARSHSTPLPPQGSGQPRGERELPNSHSMICPKAAGAPAAPPAPAA.[L]	4976.48672
11115	[K].IRDTFCSYSVMELCTKGLGNQVEVLKTRGINLSCIRTCVVVAE.[E]	4976.49231

11116	[L].KIPLFKMKDVILILCLLKLSSAVPYSRFGFGKSFNSLWMNGLL.[P]	4976.73708
11117	[V].KEGANKEVLGILVSYRVKVKLVVSRGGDVSVELPFVLMHPKPHDH.[I]	4976.76589
11118	[L].APGDATLKHSTKADPAPSLGPMAAGSQKHSHDSGVPPVVDERTGSEGATAS.[P]	4977.40315
11119	[D].ELPEVNDLTLDGNPFLDPGALQHQDAPMISGVVPACARSALTMGVSGT.[L]	4977.40671
11120	[R].APPSSPFSDVRPDPPWACLAPRRTLPCSPCGLQSTSFRVRGGQDP.[V]	4977.40938
11121	[T].TPLTGRDGSLASNPYSGDLTKFGRGDASSPAPATTLAQPQQNQTQTHH.[T]	4977.41102
11122	[V].YSISEQVSAVEPTKFVRETDTFEWLFSPPLEETTRKDEEKGAT.[G]	4977.43164
11123	[L].TALGMVDVFDRSRANLSGIIAGGGLGVSKIIHKAMLEVNESGSEFTLT.[N]	4977.58124
11124	[S].PGINMICETVDKQNTAENGSLSLGEKSTVPVEQGLSSSKSEITNELS.[V]	4978.41435
11125	[T].QEEGEPRPVGLEMPPARSQPSQVAEFSRTACSLQPAPRDPPRPEP.[V]	4978.43229
11126	[A].GGPPLIQQNASEAEIFYNATGFLRNLSSASQATRQKMRECHGLVD.[A]	4978.43229
11127	[D].AEADAAAAGLAGWPGAGPSPASFPQTAAPRGAEDPSRDALQPGASASLPLAPGG.[T]	4978.43544
11128	[P].SKGGACPSRAKMSMTGAGKSPPSVQSLAMRLLSMPGAQGAPAAGPEPPPAT.[A]	4978.44642
11129	[Y].TLDSSAAPAPQAAPAPTPAAAAPTPAPSAQAPGSSYPTHMQVVLPALSPTMT.[M]	4978.46012
11130	[A].MTHVINQGMAMYWGTSRWSSMEIMEAYSVARQFNLIPPICEQ.[A]	4979.29044
11131	[G].GPGPVGAPDTRKEMASMPGTAITTSSAKKEDLVPSEEETQALTPGAQGPS.[S]	4979.42486
11132	[G].QELSAGDTGVNTAYLATTAAVSSLPSRITGEQTGCTQLGDKAWLQPSGA.[E]	4979.43272
11133	[A].GPPEYGLGDPPPPPGLLQPPTLAPWQPSRADGPPATPTQPSGGRSLGEDG.[P]	4979.45986
11134	[A].QPPGGGRTFPAGLPGSRQDGDADARQPSQHSARMEIPPTHYPAARAAS.[V]	4980.40549
11135	[R].VMFSSFGQIEECRILRGPDGLSRGCAFVTFTTRAMAQTAIKAMH.[Q]	4980.41981
11136	[R].RGSFVNSSGVMNQGVAPMVGTPAPGGSPYGQQVGVLGPPGQQAPPPYPGPH.[P]	4980.43083
11137	[A].VGRAAGADVCGGVLTGLSGVLVSPEYPNNYPNNVECHWVIRASGPATV.[K]	4980.45196
11138	[G].TNGIHVTGGSMTVTGNIYIYNGPVLGGARGPGDPPAPPEPPYPIPEEGAP.[R]	4981.44652
11139	[S].GSEIYERLTPGQPGNQLYVVGTSSMSLGQQKTPGLTGRPNPCPAAPPG.[T]	4981.4571
11140	[A].TQYVPGPGQPPAPSSYPGHRLPLQQGTSPSLSTASPAGPHFKPAEQFN.[G]	4981.46562
11141	[L].MIQQEKAPKPSETPAGCLIMSKGPKENVEQIIEEPGLIMMEQGKA.[P]	4981.48516
11142	[C].AVSTPEQSATPAGAVPTPEQSATLAGAVSTPEEPATPAGAVSTPEEPATPAVS.[T]	4982.42768
11143	[G].PGSPATLSPSAGVPQPVGMEALDQAEGPAASQRAMPPPPPASPPSEPAQKP.[P]	4982.42989
11144	[L].PGQEAGKGRNAHLSSPSSLSLNSSHPGNLSSPEAGIRNPTVCLQTRDT.[L]	4982.45217
11145	[M].KGESGDLGPQGPRGPQGLMGPPGKAGRRGRAGADGARGMPGEPGVKGDTGAQ.[G]	4982.45688
11146	[N].PNVTHLLKKIRLLNEYQKEAPSFWIRHPEKYMEEIVESTLS.[L]	4982.6237
11147	[S].SQTQNKAAGSGPGKGVSATSSTSGLPDMTGSVYNKTQTFDKQGFHAGTPP.[P]	4983.38135
11148	[S].SGSLWQEIPVVRNSTVLLSMTHEDQKLQEVPPGRAGAGAAEGQAGSPL.[F]	4984.52215
11149	[S].FAVGGMIASFFGGLLGDKLGRIKALLVANILSLVGALLMGFSKLGPSHI.[L]	4985.82392

11150	[S].PAGFSPGMSVPGPLIPLCAPFPPTGALEIGQALPIHTGRYTCTARNAAG.[M]	4986.52156
11151	[H].VAATRSIMQPYYQGPVGDPLRYGLPYEDRVRVWQLYGVRESVS.[P]	4987.5312
11152	[G].AKGEQGPAGHPGEAGLPGPSGNMGPQGPKGIPGNPGLPGPKGEMGPVGPAGNPGA.[K]	4988.41664
11153	[A].FPVVHSPYGGGVGPEPVLGGQSAFTVPPVQNFMGAAGVYQAQGLVGSSNGS.[S]	4988.43121
11154	[T].PGMPGVKGSAGQAGRPGNPGHQGLAGVPGMPGTKGGPGDKGEPGRQGFPGVSGP.[P]	4988.4391
11155	[A].EPRPPAGEAPAKSATAVPSGPGAAEPTQEGLTGKLFGLGASLLTQASTLMSA.[Q]	4988.56736
11156	[G].RDGIKGDPGPPGPMGPPGGMPGLPGRDGMTGAPGLTGERGEKGEPGERGPPG.[F]	4989.37887
11157	[S].PGSPTPSMSADQAASSPLLPLNSPGLSQGDVSRQDEAVSPGASCVDGLGPK.[P]	4989.38406
11158	[K].GLGPRDQDGARPPGEGSSTGASPGSPPGAEVEALPEAAALEVAEPPAEALGEA.[S]	4989.39844
11159	[E].PYPGPAAHSQGLPTASPSLSYSTGHSPALSGHGGGWGPSSLGGGGEASPSHII.[R]	4990.37792
11160	[G].PGGHWLCCQLGAAVPGPLPGLSAGPTGSRCAEAPPLEGTPLASAGMQGPQG.[A]	4990.38554
11161	[L].PGRKYTVNVYEISEEGEQNLILSTSQTTAPDAPPDPTVDQVDDTSI.[V]	4990.39638
11162	[K].QGHPGVPGNPGHNGLPGRDGRDGAKGDKGDAGADGHVEAKGVKGDQGSRGPP.[G]	4990.41481
11163	[R].GDPGTPGVPGKDGQAGHPGQPGPKGDPGVSGIPGAPGLPGPKGSTGGMGLPGMPGP.[K]	4990.42105
11164	[L].PQGFSGLTHLLHWPLTPGDGGDLLASTSPQTSTYHLRSPAQTSYALGG.[Y]	4990.47585
11165	[M].ASPAMLASVESGGPPPPTASQSASVSIPGSLPSSTPYTMPPEPTLSQNPLS.[I]	4991.41765
11166	[P].PGPEGPAGFPGPPGIQGNPGPVGDPGERGPPGRAGLPGSDGAPGPPGTSLMLPF.[R]	4991.45334
11167	[H].QASFLEPQQGTMGAAGSSFGLMQPRPPPEPGPAGRHRGVRAGQQLAYA.[R]	4991.45402
11168	[P].PNHGRGPGRACHAAGSTPAVSIPTKLRSFPQQQFAHQGNPAAYSMVH.[M]	4991.45536
11169	[E].VGGAENKEAGKTLDQVGQCMVASAAVVTTASSTPTTVRISDTGLATGTGPE.[K]	4991.45722
11170	[S].PGSRGPPGGYGEKGFPGDPGNPGQSSNIKGQKGSKGEQGRQGRTGQKGTPG.[S]	4991.46037
11171	[G].DAGLGDDLGSPVLSAKQGKNWERSMLVEAKGQKECHQVSVRFQVH.[Y]	4991.50031
11172	[Y].GAGVTKEDLQNMGAKNVCLMTDKNLSQLPPVQTAMDSLVKNGINFK.[V]	4991.50973
11173	[N].TKKDSESAPVKGGTMTDLDEQEDESMETMGKDEDESSPGNKGEQTK.[N]	4992.16744
11174	[V].TMERVQRPAVEEEGGAASYNASSKEQPVVFNHVYNINVPLDSLCS.[S]	4992.38908
11175	[G].ADIDEVTVNVTVLDANDPPVFSLNIYSVQISEGVPTGTHVTFVSAFD.[S]	4992.46769
11176	[E].TGEKTPSSLDIEPGSFSSGRVSVEGSRIQGLDYNQEHFPFRVNGPD.[A]	4993.39872
11177	[R].TEAALDRESMDRHYLRVTAQDHGSPRLSATTMVAVTVADRNDHAP.[V]	4993.40278
11178	[L].PQTAEEIQNTLTSPQFQQALGMFSAALASGQLGPLMCQFGLPAEAVE.[A]	4993.40564
11179	[P].PVPQTEYEATAGTVPFKDGGSVGVMMTQITDAKDGLLWPERAGEVNV.[H]	4993.4234
11180	[M].YDTVNQSKTPFITHVAPSTSTNLTMTFNNQLNTVHNQAPTSTSATL.[S]	4993.42724
11181	[H].HGSPGPLSVYPPASSSSLSAGHSSPHLFTFPPTPPKDVSPDPSLSTPGSAG.[S]	4993.42789
11182	[S].DIKSQERPCHLELHQRESNSDIPKNSFTKSLDSCRSQVLPQE.[G]	4993.42793
11183	[D].KPDLDPQFVREFYSASVAEDPPQGTSVLRVEAMDGDRGINDPVIY.[S]	4993.43127

11184	[E].DGRLGSQGFVYVMANKQPLWNEATQVYQLDFGGRVTQESAKNFQ.[I]	4993.4326
11185	[S].PAAATHQSQIYGRSQTVAMQGPARTLTMQRGMNMSVNLMPSPAYNV.[N]	4994.39505
11186	[R].VAEEGAAAHLENGIALSGLESCVMSAPPGSGPLEVTTDSLPNGPALADGPAP.[V]	4994.4034
11187	[S].SVQCPLGMQLPLPFGELDLDSTTPPAYSLYAPEPPPSYEEVIKMT.[K]	4994.4076
11188	[A].RGALVEETTTYFQTSAHSAPFFAPKGTSSTSQVPQPAQVSGPSTAQQN.[P]	4994.41912
11189	[G].GPTAAHSAGPSNTGTGLPTQEVPIVRMTHLLDSENSGAELQGRAVDPGGAG.[P]	4994.4297
11190	[L].QAAGVAADWAAAGLADGARAAGHAGHGPAHGGLAGHGAAAAGVAVETGLEAASATAQ.[G]	4994.4389
11191	[E].SVSDKAPSPATLPATSSSLPSPATPSHGSPSSHGPSAPHPTSPTPPVTAGGATA.[A]	4994.44025
11192	[C].QELKGSADSFVDPPETPGHGAPRRTCREPGCLSPPHSASQRGDQLL.[P]	4995.3973
11193	[M].AIQAYEDMVTQPPLTGERVSNLEALPVNEYCECIKVQTDKGTEV.[A]	4995.40604
11194	[C].PESISVTIVSASPTTPCIEVGPNTNVTSTPTVPLSVFTSTTEMATSPSS.[T]	4995.42246
11195	[V].KPGECSLLPPFSPRPRWNAGSAAGEVEPGTSRGSFLITESAPTYAHL.[S]	4995.48464
11196	[Q].GPDWHLKMPKMKMPKFSMPGFKGEGPEVDVNLPKADIDVSAPKVD.[I]	4995.49156
11197	[H].GPDWHLKMPKMKMPKFSMPGFKGEGPEVDVNLPKADIDISGPKVD.[I]	4995.49156
11198	[P].ERPQPPTLASTPMQNGGLRDSSQVPRTLEGNPGASAEPMLGAGGRGPSPG.[P]	4996.43883
11199	[S].KKSKPLMRSEQEKSTGESVDGMIALDNSPPGLLNQTECVLDNQVH.[L]	4996.44488
11200	[A].QGPVEVPLLEAEVGEAASHLASYALGSSSANVEALPQESLDRMMANLL.[K]	4996.45543
11201	[P].ADHGLAGRGAAGDGPAAALLQAAGVAADWAAAGLADGARAAGHAGHGPAHGGLAGHG.[A]	4996.45589
11202	[S].SRTTTPHSTPSHPETLPIHVSTSATTSVTPTSHRVITPTESHATYST.[T]	4996.46713
11203	[I].PPMTKSPSSMMLTISRQTPADPRNKGADSDPKRPPGPSGIPAPTATPAS.[P]	4996.50776
11204	[I].PGGVADLNNPRGPGAAGESDGTVGVIPQFPGAPQVPGPGGDAAPGAGILSNRPL.[Q]	4996.53001
11205	[E].KKIEPNISFDSSTQCSGKEAILFKLETAGEIDRKQQQDSDLSVR.[M]	4996.53204
11206	[I].SPSGHPMPSEAKMRLKATLTHQVSSINGGCGMVVGTASTMVTARPEQS.[Q]	4997.41585
11207	[E].LGPQLSLDGSPGDGDRHSTPSLLEAALTQEATAPDSQVWPTAPDITRE.[T]	4997.43992
11208	[S].TGNIQVGLPEPASVSNHVSAPFELSIHKHDITLQFSEAERTGDGSPK.[N]	4997.50279
11209	[S].QAIQNQPTPAQPGVYNNMSITVSMAGGNANVQNMSPMMGQMQMSSLQ.[M]	4998.24396
11210	[Q].KTTGPSANAVLVSETEEEGSMSALKTATMPGMASADQADSSSEDTTSSSD.[E]	4999.16202
